# Synthesis and Evaluation of Peptoid‐Based Compound Libraries as Inhibitors of Parasite Metallo‐Aminopeptidases

**DOI:** 10.1002/cmdc.70433

**Published:** 2026-09-10

**Authors:** Mirta Elisa Aguado, Gerardo M. Ojeda‐Carralero, Mario Antonio Rodríguez, Isabella Rúa, Heisy Guerra, Edoardo Bailo Hernández, Guglielmo Coppola, Susana Alberti, Ariatne Rodríguez, Maikel Izquierdo, Christian Betzel, Filippo Pettazzi, Julieta Coro‐Bermello, Erik V. Van der Eycken, Bernhard Westermann, Jorge González‐Bacerio, Daniel G. Rivera

**Affiliations:** ^1^ Center for Protein Studies Faculty of Biology University of Havana Havana Cuba; ^2^ Laboratory of Synthetic and Biomolecular Chemistry Faculty of Chemistry University of Havana Havana Cuba; ^3^ Laboratory for Organic and Microwave‐assisted Chemistry (LOMAC) Department of Chemistry Leuven Belgium; ^4^ Department of Bioorganic Chemistry Leibniz Institute of Plant Biochemistry Halle (Saale) Germany; ^5^ Institute of Biochemistry and Molecular Biology Laboratory for Structural Biology of Infection and Inflammation University of Hamburg Hamburg Germany; ^6^ Finlay Institute of Vaccines Havana Cuba

**Keywords:** Chagas, enzyme inhibitors, leishmania, malaria, metallo‐aminopeptidases, multicomponent reactions, peptoids

## Abstract

Parasitic diseases are a worldwide health and socio‐economical problem, especially due to increasing parasite resistance and frequent toxicity of commercial drugs. Parasite metallo‐aminopeptidases are potential therapeutic targets, as they perform crucial biological functions during parasite development within the human host. Here, we describe the synthesis and biological evaluation of two libraries consisting of a total of 74 peptoid‐based compounds. The synthetic approaches use a multicomponent reaction to install various diversity elements, and in library one, this is followed by subsequent coupling to α‐amino acids to create further diversity within a peptoid‐peptide hybrid skeleton. The libraries were evaluated against three metallo‐aminopeptidases: PfA‐M1 from *Plasmodium falciparum*, TcLAP from *Trypanosoma cruzi*, and LmLAP from *Leishmania major*, which allowed the discovery of moderate to potent inhibitors of each enzyme. Molecular docking studies were conducted to help understand the binding mode of the inhibitors with the enzymes, predicting the importance of hydrophobic interactions in the inhibition process.

## Graphical abstract

Evaluation of two libraries of peptoid‐based compounds for inhibitory activity against PfA‐M1 from *Plasmodium falciparum*, TcLAP from *Trypanosoma cruzi*, and LmLAP from *Leishmania major* reveals the crucial role of hydrophobic interactions in the inhibition process of these parasite metallo‐aminopeptidases. The use of multicomponent chemistry allows the creation of structural diversity at peptoid and peptoid‐peptide hybrid scaffold.

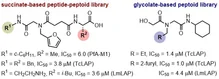

## 1 Introduction

Malaria [1], Chagas [2], and leishmaniasis [3] are parasitic diseases that affect human health and the economy of developing countries. Considered neglected diseases and facing a low priority of the pharma industry, these diseases also feature widespread resistance to traditional drugs, which often show toxicity and low efficacy [4, 5, 6]. As part of the global endeavor to identify and characterize novel parasite targets, a significant interest has been posed in metallo‐aminopeptidases (MAPs) as promising drug targets [7, 8, 9]. Aminopeptidases are proteolytic enzymes involved in crucial physiological processes, and MAPs are a specific subgroup that uses one or two metal ions in the active sites to catalyze the proteolytic reaction [7, 8, 9].

**FIGURE 1:**
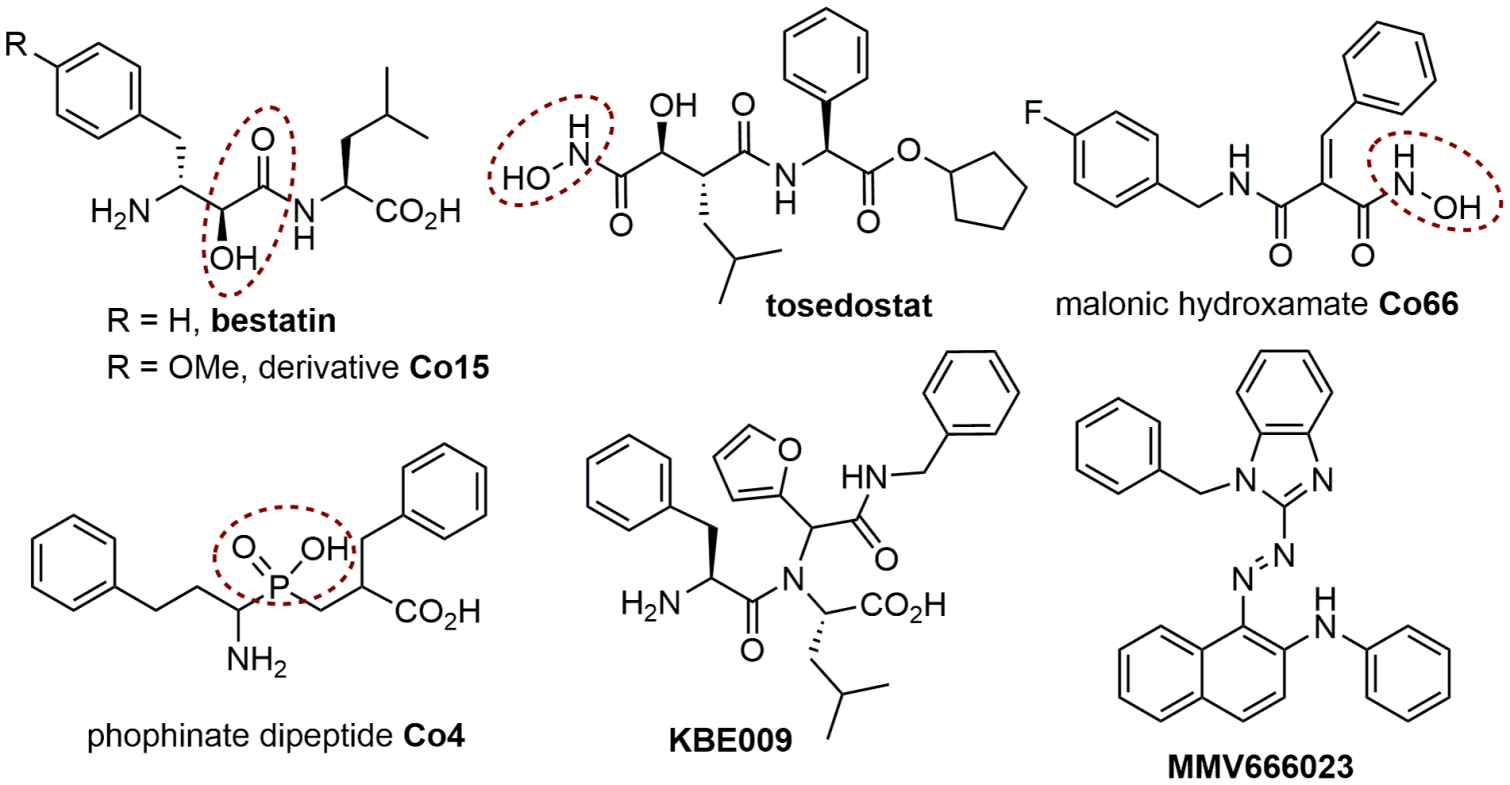
Inhibitors of MAPs including hydrophobic substituents and, in some cases, a metal‐chelating functionality (red dashed circle).

The *Plasmodium falciparum* M1 alanylaminopeptidase (PfA‐M1, EC 3.4.11.2) is an essential MAP involved in host hemoglobin degradation during the parasite’s erythrocytic stages, making it a validated antimalarial target [7, 10, 11]. Most PfA‐M1 inhibitors that have been identified over the last few years are compounds functionalized with a metal‐chelating group and bearing hydrophobic, voluminous substituents that favor stabilizing interactions within the hydrophobic pockets of the enzyme’s active site [9, 12, 13, 14, 15, 16, 17, 18, 19, 20, 21]. As shown in Figure 1, many of these inhibitors have small *pseudo*‐peptide scaffolds incorporating an α‐hydroxy‐carbonyl moiety [12, 13, 14] (e.g., bestatin and its derivatives), a hydroxamate functionality [15, 16, 17, 18] (e.g., tosedostat and malonic derivative Co66) or a phosphinate group like the one found in compound Co4 [19]. Eventually, the strong metal‐chelating capacity of these functionalities can lead to poor selectivity between parasitic and mammalian MAPs, a drawback in the drug development process. Nevertheless, it is a great challenge to discover efficacious PfA‐M1 inhibitors that lack the functionality to chelate the catalytic metal ion. To this end, it is necessary to produce compounds that interact with the enzyme’s catalytic site with high affinity solely through matching hydrophobic interactions. Examples of such compounds include KBE009 [20] and MMV666023 [21] (Figure 1), which bear various aromatic residues that interact favorably with the enzyme’s hydrophobic pockets.

Besides PfA‐M1, we are also interested in other MAPs as targets for the development of antitrypanosomal and antileishmanial compounds. In this context, TcLAP (clan MF, M17 family, EC 3.4.11.1) is the acidic M17 leucyl‐aminopeptidase (LAP) from *Trypanosoma cruzi*, which is expressed in the three parasite stages: epimastigotes, amastigotes and trypomastigotes, and it mediates the major LAP activity in parasite epimastigotes [22]. Like PfA‐M1, TcLAP can be inhibited by bestatin and small molecules containing hydrophobic substituents, several of which have shown in vitro antichagasic activity [23, 24, 25]. Similarly, LmLAP (clan MF, M17 family, EC 3.4.11.1) is the basic M17 LAP from *Leishmania major* [26] and a validated target for this parasite [27]. This enzyme is expressed across all protozoan life cycle stages, including promastigotes and amastigotes, and may participate in nutrient supply. Typical inhibitors of M17 LAPs such as bestatin and analogs like apstatin, as well as *pseudo*‐peptidic hydroxamates like actinonin, among others also inhibit it [26, 27, 28].

As part of our endeavor to discover synthetically accessible inhibitors of microbial MAPs [20, 23, 24, 25, 27, 28, 29], here, we describe the construction of two small peptoid‐based compound libraries and their evaluation against the three parasite MAPs. Our approach is to assemble synthetically accessible scaffolds incorporating hydrophobic substituents that are likely to lead to matching interactions with the catalytic sites of these relevant enzymes. In one of the libraries, we sought to avoid introducing classic metal‐chelating groups like hydroxamates and α‐hydroxycarbonyls (e.g., in bestatin‐like compounds), which typically confer poor selectivity between microbial and human enzymes. Our focus is on the assembly of two types of peptoid‐based skeletons with conformational flexibility and either two or three centers of structural diversification, in which hydrophobic substituents can be readily installed in a combinatorial manner.

## 2 Results and Discussion

### 2.1 Synthesis and Screening of a Succinate‐Based Peptide‐Peptoid Library

Peptide‐peptoid hybrids are compounds incorporating both α‐amino acids and *N*‐substituted glycine residues [30]. In general, small peptoids (oligo *N*‐substituted glycines) are molecules characterized by a high conformational flexibility derived from the amide bond isomerism owing to the few rotational axes and a low energy difference between the *s‐cis* and *s‐trans* rotamers [31, 32]. While in canonical peptides the high energy barrier (~14–20 kcal.mol^−1^) for the C─N bond rotation usually leads to non‐interconvertible amide bond rotamers, the *N*‐substitution in peptoids reduces the energy barrier and allows the interconversion of the two configurational isomers [30, 31, 32]. As a result, small peptoids are typically characterized by the presence of both rotamers [33, 34], which often complicates their NMR spectra.

As shown in Table 1, the synthetic strategy was designed to build the parallel library of succinate‐based peptide‐peptoid hybrids. The first step is an Ugi reaction [35, 36] — i.e., the condensation of a primary amine, a carboxylic acid, an aldehyde, and an isonitrile to form a bisamide scaffold—starting from monomethyl succinate as carboxylic acid component. Here, this multicomponent reaction (MCR) introduces two elements of diversity arising from the amine and isonitrile components, so we employed four different amines and isonitriles in the library construction. As part of the design, the focus was on introducing aliphatic and aromatic substituents, as well as an ionizable amino group, since many MAP inhibitors bear a free terminal amine. Isonitrile‐based MCRs are powerful tools for the discovery of antimicrobial compounds [36, 37, 38], including inhibitors of microbial MAPs [20, 29, 39]. They are also particularly well suited for the rapid assembly of peptidomimetics featuring chimeric peptoid‐based structures [40, 41]. In most cases, the aldehyde can be an additional source of diversity, but due to the poor diastereoselectivity of these reactions, prochiral aldehydes typically lead to the formation of diastereomers. In our experience, the formation of diastereomers, combined with the presence of stable rotamers derived from *s‐cis* and *s‍‐‍trans* isomerization, makes it very difficult to perform chromatographic purification and NMR analysis of the final compounds. As a result, here we sought to use paraformaldehyde as the oxo‐component of the Ugi reaction, while adding a third element of diversity via peptide coupling of the deprotected succinate‐based Ugi adduct with a variety of α‐amino acids (Table 1). The Ugi reactions were conducted for 24–72 h to ensure total consumption of monomethyl succinate, as indicated by TLC. Without further purification, the Ugi products were deprotected and coupled to C‐protected amino acids using HBTU/DIPEA. A library of 64 hybrid compounds was built and purified by column chromatography. The final acidic cleavage of the *t*Bu/Boc protecting groups was almost quantitative and clean, yielding the final compounds. Nevertheless, these latter were submitted to an additional, often challenging chromatography purification to ensure the high purity (>95%) required for biological evaluation. Characterization by NMR spectroscopy and mass spectrometry (MS) confirmed the structure and purity of the library members (see the Supporting Information, SI).

**TABLE 1:** Combinatorial synthesis and preliminary evaluation of the inhibitory activity of a library of succinate‐based peptide‐peptoid hybrids against parasite MAPs.

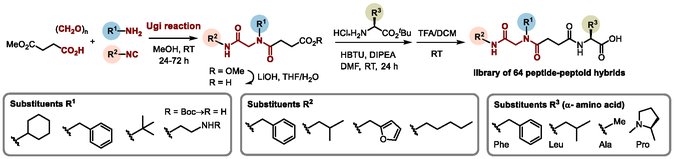							
Number		**Peptide‐peptoid hybrid** a	Code	**Yield, %** b	** Residual enzyme activity (%) at 100** μ**M of compound** c		
					PfA‐M1	TcLAP	LmLAP
**13**	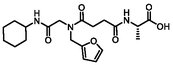		GM513z	17	34.9 ± 2.8	69.4 ± 12.9	96.7 ± 25.7
**18**	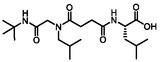		GM518z	40	49.8 ± 0.1	53.4 ± 7.5	91.6 ± 1.2
**19**	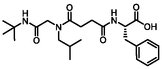		GM519z	42	49.9 ± 0.2	100.7 ± 9.8	109.3 ± 2.5
**25**	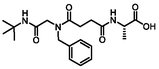		GM525z	29	48.9 ± 12.0	106.1 ± 9.6	103.7 ± 7.7
**36**	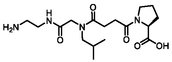		GM536z	37	86.9 ± 16.7	162.9 ± 15.4	21.7 ± 2.1
**39**	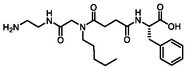		GM539z	32	166.7 ± 0.0	144.8 ± 13.2	44.9 ± 3.5
**41**	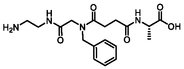		GM541z	27	43.9 ± 8.6	92.1 ± 20.0	20.8 ± 9.6
**43**	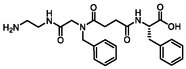		GM543z	36	48.4 ± 2.3	124.2 ± 11.9	15.7 ± 2.1
**46**	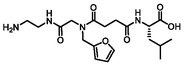		GM546z	30	100.0 ± 0.0	118.6 ± 13.2	0.0 ± 0.0
**50**	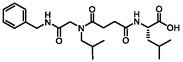		GM550z	32	113.4 ± 0.9	81.9 ± 8.7	7.9 ± 1.2
**51**	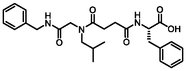		GM551z	42	99.9 ± 1.9	78.8 ± 6.5	10.8 ± 5.3
**59**	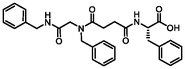		GM559z	27	23.3 ± 12.8	156.0 ± 124.0	93.8 ± 32.7
**60**	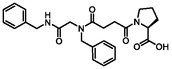		GM560z	46	45.4 ± 3.6	16.0 ± 1.6	114.6 ± 51.9
**63**	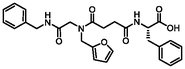		GM563z	17	60.3 ± 0.2	28.0 ± 18.0	265.4 ± 73.9
**64**	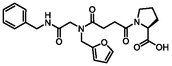		GM564z	27	67.1 ± 1.9	42.1 ± 4.5	116.6 ± 69.1

a A total of 64 compounds were produced in this combinatorial parallel library. Here, only those compounds leading to a residual activity (at 100 μM) lower than 50% in at least one of the three MAPs are shown.

b Overall isolated yields after the four steps: (1) Ugi reaction, (2) methyl ester hydrolysis, (3) coupling to the α‐amino acid, and (4) final deprotection.

c Inhibition assays for recombinant metallo‐aminopeptidases (*n* = 3). DIPEA, *N*,*N*‐diisopropylethylamine; HBTU, hexafluorophosphate benzotriazole tetramethyl uronium; TFA, trifluoroacetic acid; THF, tetrahydrofuran.

The recombinant enzymes PfA‐M1 [42], TcLAP [23], and LmLAP [28] were expressed and purified in our group as reported previously. The aminopeptidase enzymatic activity was assessed using a continuous kinetic method, as described in the SI. The selection criterion for subsequent concentration‐response studies was a reduction in enzymatic activity of more than 50% relative to the control for PfA‐M1 and TcLAP and a reduction to 0% for LmLAP. Thus, of the 64 peptide‐peptoid hybrids produced in the first library and tested against the enzymes, only some were selected for the concentration‐response studies. A full list of compounds and their inhibitory activity is included in Table S1.

**FIGURE 2:**
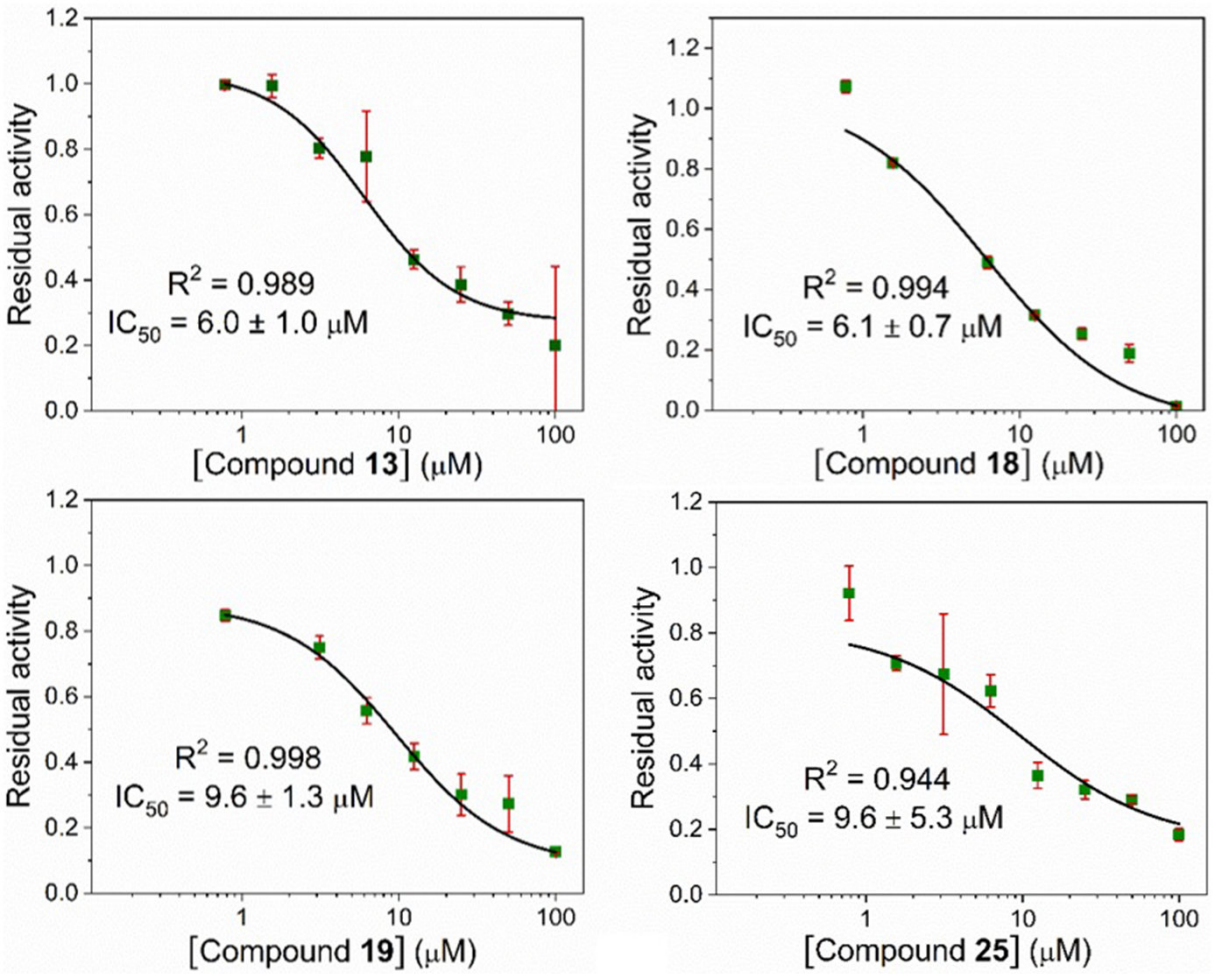
Concentration‐response curves for the calculation of the IC_50_ in the inhibition of PfA‐M1 by four peptide‐peptoid hybrids. The values are means ± SD (*n* = 3).

For PfA‐M1, eight compounds (**13**, **18**, **19**, **25**, **41**, **43**, **59**, and **60**) met our criterion and were selected for determination of IC_50_ values via concentration‐response curves. Four of these compounds resulted in PfA‐M1 inhibition IC_50_ values greater than 100 μM. In contrast, compounds **13**, **18**, **19,** and **25** showed IC_50_ values of 6.0, 6.1, 9.6, and 9.6 μM, respectively (Figure 2), so they can be considered as moderate PfA‐M1 inhibitors. In the literature, several PfA‐M1 inhibitors have exhibited *K*_i_ or IC_50_ values in the 1–10 μM range [21, 43, 44]. However, it is worth‐mentioning that other known compounds—especially those including a metal‐chelating functionality—have been shown to inhibit the enzyme with *K*_i_ or IC_50_ < 1 μM, which is a more relevant result [7, 14, 15, 16, 17, 18, 19, 20, 43, 45].

Although the best inhibitors from this library were those bearing aliphatic substituents at *R* ^2^ (i.e., cyclohexyl and *t*‐butyl), there was no clear rationale for the differences in PfA‐M1 inhibitory activity, especially because we were unsure whether these compounds could coordinate the zinc ion within the active site of PfA‐M1 [14, 19]. As a result, we sought to conduct a molecular docking study that helps us understand the binding mode of this set of compounds.

**FIGURE 3:**
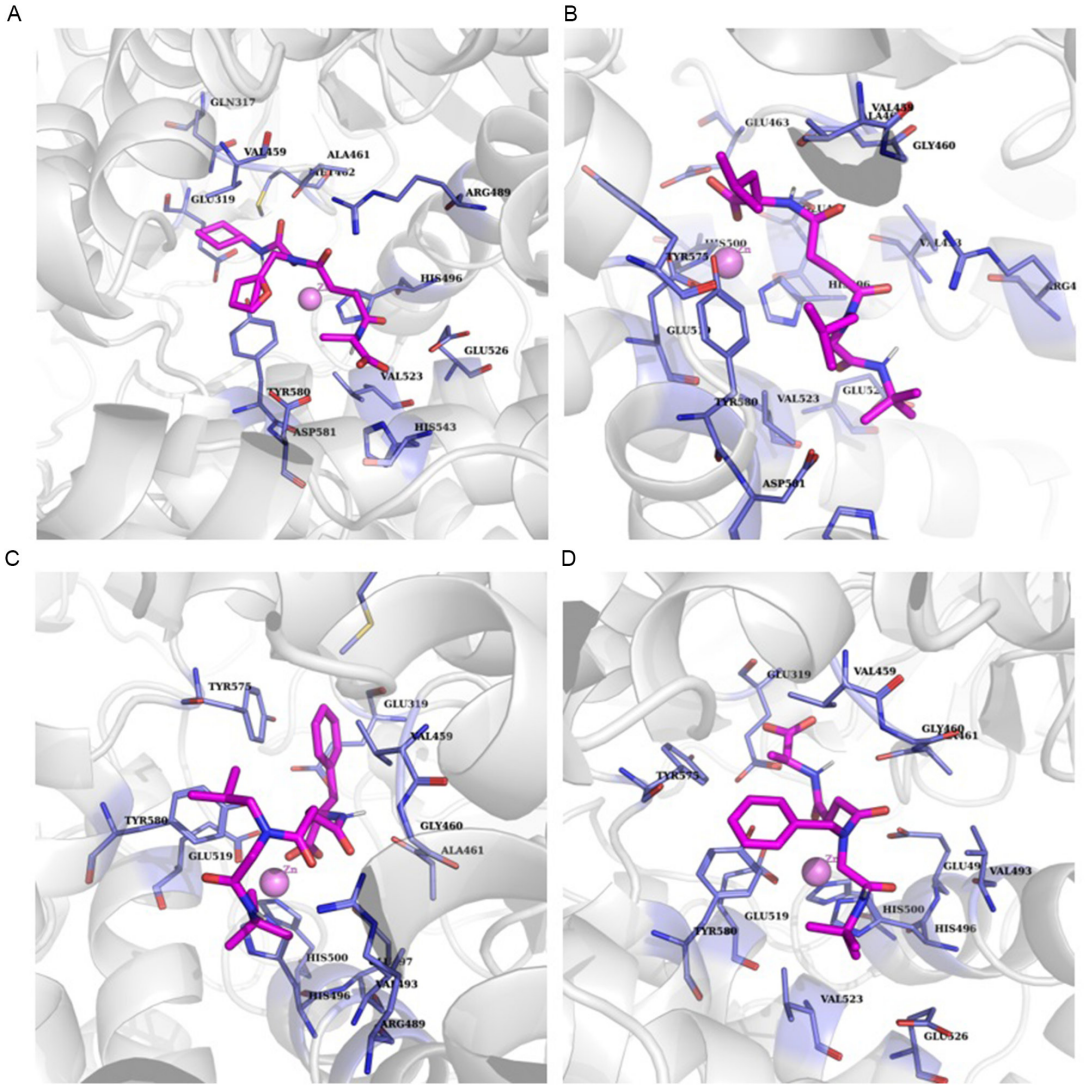
3D representation of molecular docking results between compounds **13** (A), **18** (B), **19** (C), or **25** (D) and PfA‐M1. The best model, in terms of binding affinity, is shown for each inhibitor after 20 runs. Bestatin was used as a control, based on the 3D structure of the bestatin‐PfA‐M1 complex reported [19]. Residues that interact with the inhibitors are represented. Nitrogen atoms are shown in blue and oxygen atoms in red. The zinc ion is represented as a sphere.

The PfA‐M1 inhibition by compounds **13**, **18**, **19** and **25** was modeled using molecular docking (Figure 3), with bestatin as a control. Modeling of the PfA‐M1‐bestatin complex in a way identical to the crystallographic structure determined by McGowan et al. [19] allowed validating the molecular docking performed in this work (not shown). Figure 3A–D shows the best model (in terms of binding affinity) after 20 runs for each inhibitor. Standard deviations for binding affinities were lower than 10% of the corresponding mean values (data not shown).

The predictions suggest that compounds **18**, **19**, and **25** could coordinate the Zn^2+^ cation in the active site (**18** and **19** with the carboxyl terminal group and **25** with the carbonyl group near the methyl function), but compound **13** does not (Figures 3 and S1). Keeping in mind that these are predictions and not crystalline structures of the ligand‐enzyme complexes, it is difficult to assess the relevance of the possible coordinations detected in compounds **18**, **19** and **25**. Certainly, the carboxylate chelation predicted for **18** and **19** is less effective than that of, for example, a hydroxamate, while the single coordination of a carbonyl seen in **25** is not sufficient to contribute significantly to a high binding affinity. This is evident in a comparison of the inhibition capacity of these three compound with **13**, which shows no evident coordination. On the other hand, predictions show that hydrophobic interactions predominate in the stabilization of inhibitor‐enzyme complexes (see Figure S1). In these interactions, the 2‐methylfuryl, cyclohexylamide (**13**; Figure S1A), methyl (**13** and **25**; Figure S1A,D), *tert*‐butyl (**18**, **19** and **25**; Figure S1B–D), isobutyl (**18** and **19**; Figure S1B,C), and benzyl (**19** and **25**; Figure S1C,D) groups are predicted to be involved.

There is substantial evidence for the relevance of hydrophobic interactions to the inhibition potency against PfA‐M1. For example, both bestatin and compound Co4 (Figure 1) chelate PfA‐M1’s Zn^2+^ and establish a similar number of hydrogen bonds; however, Co4 forms a higher number of hydrophobic interactions within the active site and is a more potent inhibitor (*K*_*i*_ 79 vs 478 nM) [19]. Similarly, for the PfA‐M1 inhibitor MMV666023 [21] (Figure 1; *K* ^*i*^ = 4.5 μM), multiple hydrophobic interactions within the active site were predicted by molecular docking, supporting their key role beyond coordination of the zinc ion.

The S1 pocket of the PfA‐M1’s active site is formed by the residues: Q317, E319, A320, V459, M462, E572, Y575, and M1034 [19]. On the other hand, the S1′ subsite includes residues T492, V493, and V523 [19]. As shown in Figures 3A and S1A, the cyclohexylamide group of compound **13** is inserted in the S1 pocket, similarly to the benzyl group of bestatin (Figure 1) [19]. In an opposite orientation, the terminal isobutyl function of compound **18** establishes hydrophobic interactions with the S1 pocket. In contrast, the inhibitor’s *tert*‐butyl substituent interacts with the S1′ subsite (Figure 3B and S1B), like bestatin’s isobutyl group [19]. In the same way, compound **19** is accommodated with the terminal *tert*‐butyl and central isobutyl groups interacting with the S1′ pocket, and the benzyl moiety oriented toward the S1 subsite (Figures 3C and S1C). Similarly, the terminal *tert*‐butyl group of **25** is inserted into the S1′ pocket, and the methyl function is oriented toward the S1 subsite (Figures 3D and S1D). McGowan and coworkers [16] reported that potent PfA‐M1 inhibition is favored when the S1′ subsite is filled and hydrophobic interactions with V493 are present. Such insertion into the S1′ pocket was predicted for compounds **18**, **19**, and **25** (Figures 3B–D and S1B–D).

In addition, docking predicts hydrophobic interactions between the four inhibitors and Y580, which is involved in catalysis (Figure S1) [46]. Similar interactions were also predicted for the binding of inhibitor MMV666023 (Figure 1) to PfA‐M1 using in silico modeling [21]. In addition, this residue establishes hydrogen bonds with bestatin, its derivative compound Co15 and compound Co4 (Figure 1) [14, 25]. Hydrophobic interactions are also predicted between compounds **18**, **19**, or **25** and E497 (catalytic role) [46] (Figure S1B–D). This residue also interacts by hydrogen bonds with the hydroxyl group of bestatin and compound Co15 (Figure 1) [14, 19] and with the hydroxamate function of the inhibitor compound Co66 (Figure 1) [45].

**FIGURE 4:**
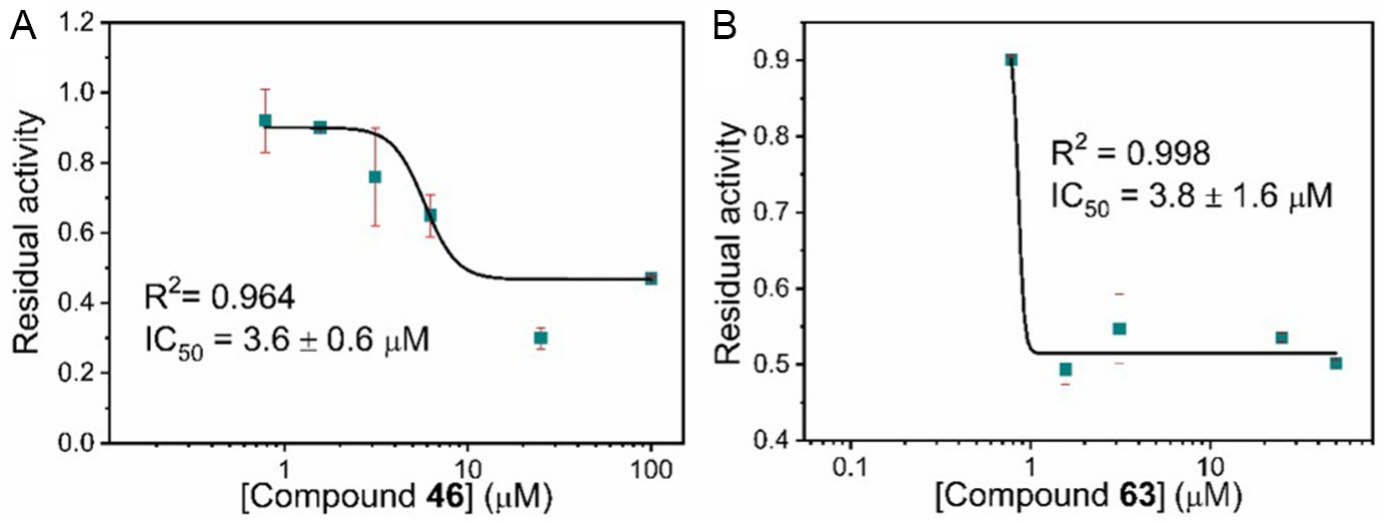
Concentration‐response curves for the calculation of the IC_50_ in the inhibition of (A) LmLAP by compound **46** and (B) TcLAP by compound **63**. The values are means ± SD (*n* = 3).

Screening of the library in Table 1 against the trypanosomal enzyme TcLAP also revealed interesting results. Although TcLAP has not yet been validated as a molecular target in Chagas disease, it is considered relevant because the enzyme is expressed across all life‐cycle stages of *T. cruzi* and is responsible for the main LAP activity in parasite extracts [22]. In this case, three library members in Table 1 (**60**, **63**, and **64**) were selected to determine the IC_50_ values. Unfortunately, compounds **60** and **64** showed IC_50_ values for TcLAP inhibition above 100 μM. However, compound **63** proved to be a moderate inhibitor of TcLAP, showing an IC_50_ of 3.8 μM (Figure 4B). This compound includes three aromatic substituents, two benzyl groups at R^2^ and R^3^, and a furanmethyl residue as *N*‐substituent of the peptoid moiety (R^1^). The molecular docking of compound **63** with TcLAP will be discussed later.

Screening the library in Table 1 against the leishmanial enzyme LmLAP also led to the discovery of a moderate inhibitor. In this case, the selection criterion for the subsequent calculation of the IC_50_ value was a complete depletion of the LmLAP enzymatic activity after incubation with the compounds at 100 μM. Of the 64 library members, only compound **46** fulfilled this criterion. This compound also bears the 2‐methylfuryl group as the *N*‐substituent of the peptoid moiety (R^1^) and an ethanamine residue at R^2^. Interestingly, it showed no inhibition for the PfA‐M1 and TcLAP enzymes. The concentration–response curve for the inhibition of LmLAP by compound **46** resulted in an IC_50_ of 3.6 μM (Figure 4A). As a comparison, we have previously determined that bestatin inhibits this enzyme with a IC_50_ of 6.1 μM [27], thus giving value to this new type of inhibitor lacking a metal‐chelating functionality. The LmLAP inhibition by compound **46** was modeled by molecular docking, and will be discussed later.

As a summary of the synthesis and screening of this library of peptide‐peptoid compounds (Table 1), we may conclude that this type of hybrid scaffold is amenable to further optimization by varying the three substituents R^1^, R^2^, and R^3^. Previously, we have exploited the power of MCRs to optimize peptoid‐based skeletons to produce submicromolar inhibitors [20], which also lack any metal‐chelating group. In this case, many other aromatic and aliphatic substituents can be easily incorporated from available amines (R^1^) and isonitriles (R^2^), while many other hydrophobic α‐amino acids can be installed *via* the coupling step.

### 2.2 Synthesis and Screening of a Glycolate‐Based Peptoid Library

A second library of peptoid‐based compounds was produced for screening against the three parasite MAPs. As shown in Table 2, we used a single multicomponent step starting from glycolic acid, again with variation of five different amines and two isonitrile components. The compounds were isolated in high purity after column chromatography, an characterized by NMR and MS. The presence of an α‐hydroxycarbonyl moiety in this scaffold aims to mimic the metal‐coordination ability of bestatin and its derivatives. However, these compounds cannot be considered bestatin‐like compounds (see Figure 1), as they neither have a free *N*‐terminal amino group nor bear residues resembling the amino acids leucine and phenylalanine.

**TABLE 2:** Combinatorial synthesis and preliminary evaluation of the inhibitory activity of a library of glycolate‐based peptoids against parasite MAPs.

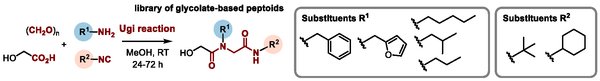						
Number	Glycolate‐based peptoid	Code	**Yield, %** a	**Residual enzyme activity (%) at 100** μ**M of compound** b		
				PfA‐M1	TcLAP	LmLAP
**65**	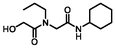	GM566	31	126.5 ± 75.9	21.4 ± 6.9	22.1 ± 1.3
**66**	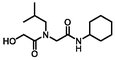	GM567	45	121.2 ± 7.7	47.3 ± 28.2	21.2 ± 3.0
**67**	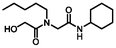	GM568	43	109.2 ± 10.6	156.9 ± 24.4	45.6 ± 4.5
**68**	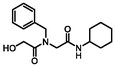	GM569	67	130.3 ± 11.1	119.7 ± 26.4	0.0 ± 0.0
**69**	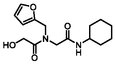	GM570	45	116.5 ± 27.5	49.2 ± 23.5	0.0 ± 0.0
**70**	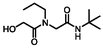	GM571	29	79.5 ± 25.9	8.4 ± 7.9	472.1 ± 67.6
**71**	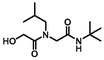	GM572	42	88.2 ± 36.4	0.0 ± 0.0	0.0 ± 0.0
**72**	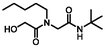	GM573	39	120.0 ± 20.3	33.5 ± 23.3	50.9 ± 27.6
**73**	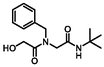	GM574	56	201.4 ± 6.2	62.5 ± 46.2	0.0 ± 0.0
**74**	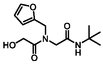	GM575	41	188.7 ± 25.9	256.7 ± 124.9	262.1 ± 70.6

a Isolated yield after column chromatography.

b Inhibition assays for recombinant metallo‐aminopeptidases (*n* = 3).

Screening of the library in Table 2 against the *P. falciparum* enzyme PfA‐M1 revealed that none of the 10 compounds showed a residual activity below 50%; therefore, these compounds were not considered for further evaluation with this enzyme. In contrast, this library proved to be a good source of moderate and potent inhibitors of enzymes TcLAP and LmLAP.

For the trypanosomal enzyme TcLAP, six peptoids were selected for the concentration‐response studies: **65**, **66**, **69**, **70**, **71**, and **72**.

**FIGURE 5:**
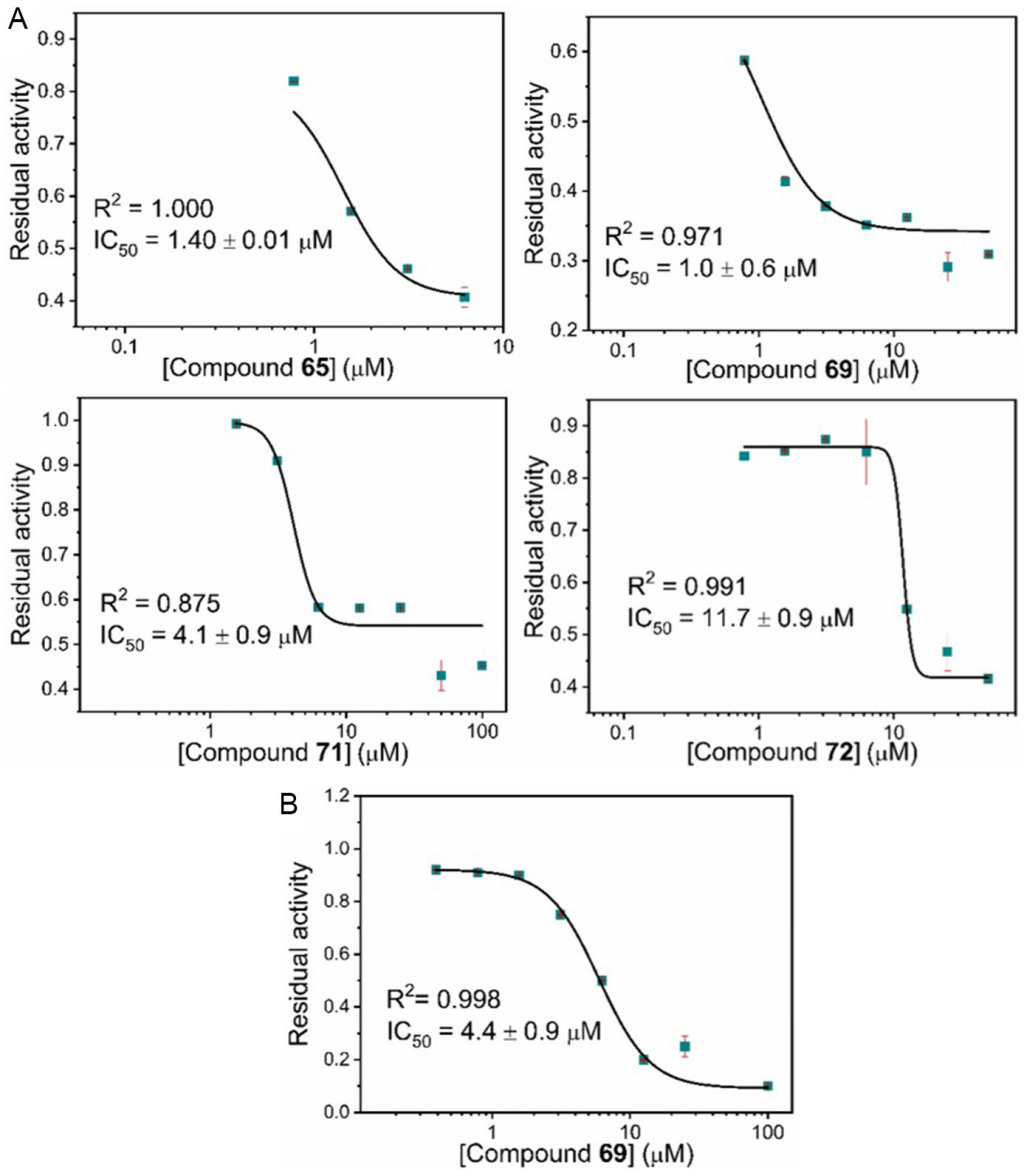
Concentration‐response curves for the calculation of the IC_50_ in the inhibition of (A) enzyme TcLAP by compounds **65**, **69**, **71**, and **72** and (B) enzyme LmLAP by compound **69**. The values are means ± SD (*n* = 3).

The IC_50_ values of **66** and **70** were higher than 100 μM, but compounds **65**, **69**, **71**, and **72** exhibited IC_50_ values of 1.4, 1.0, 4.1, and 11.7 μM, respectively (Figure 5A). These peptoids, obtained in a single step, can be considered moderate to potent TcLAP inhibitors, and their inhibition level is similar to more complex compounds described in the literature [23, 47].

**FIGURE 6:**
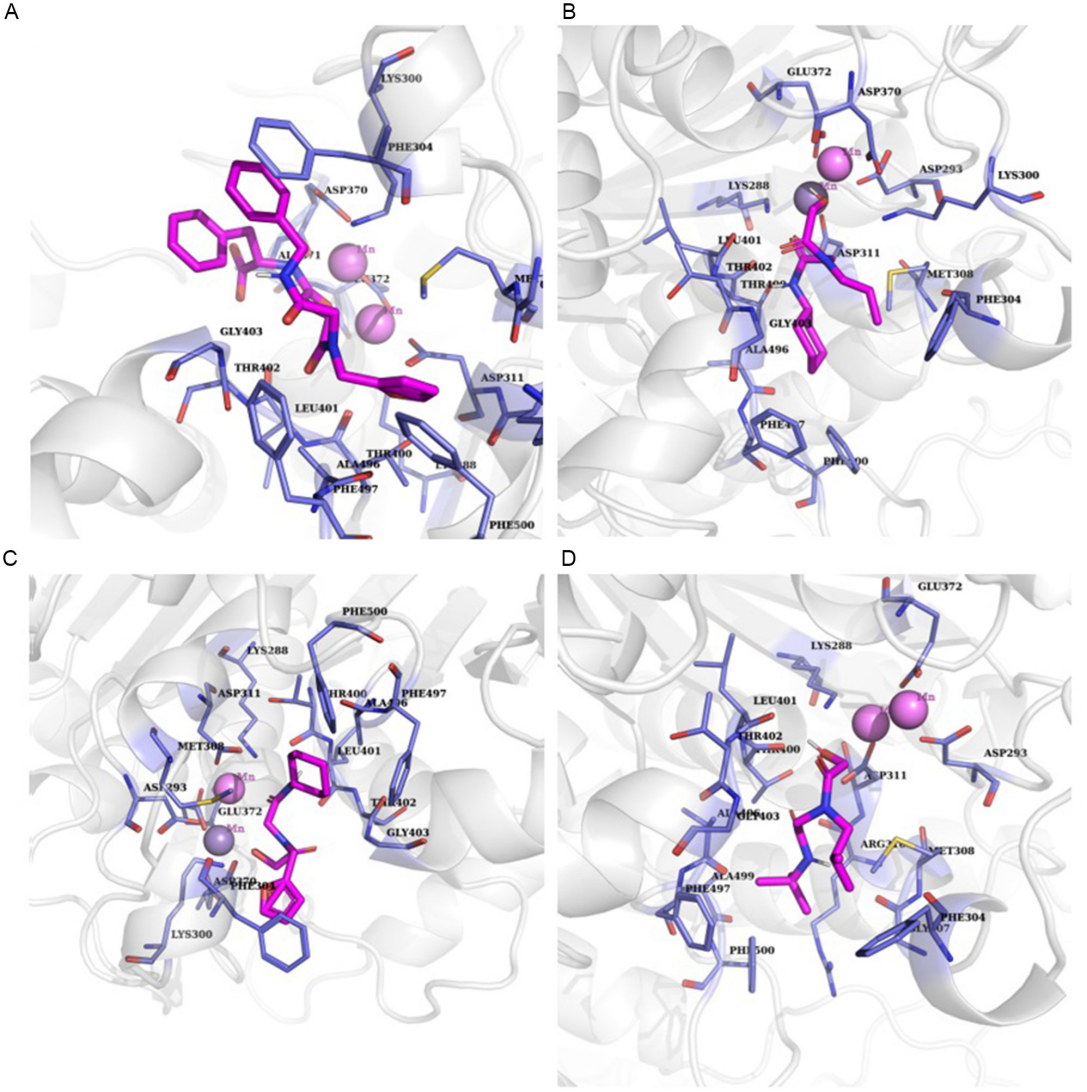
3D representation of molecular docking results between compounds **63** (A), **65** (B), **69** (C), or **71** (D) and TcLAP. The best model, in terms of binding affinity, is shown for each inhibitor after 20 runs. Residues that interact with the inhibitors are represented. Nitrogen atoms are shown in blue and oxygen atoms in red. Manganese ions are represented as spheres.

Together with compound **63**, the TcLAP inhibition by compounds **65**, **69**, and **71** was modeled by molecular docking. Figure 6 shows the best model in terms of binding affinity after 20 runs for each inhibitor. The IC_50_ value for **72** is higher than 10 μM (Figure 5), so its inhibition was not modeled. Predictions suggest that all inhibitors coordinate the Mn^2+^ cations in the TcLAP active site (compound **63** with the carbonyl group more distant to the 2‐methylfuryl function and **65**, **69**, and **71** with the terminal hydroxyl group) (Figures 6 and S2). However, the expected chelating interaction with the metal involving both the hydroxyl and the carbonyl was not detected. As for PfA‐M1, hydrophobic interactions predominate in stabilizing inhibitor‐enzyme TcLAP complexes (Figure S2). In these interactions, the 2‐methylfuryl (**63** and **69**; Figure S2A,C), benzyl (**63**; Figure S2A), *n*‐propyl (**65**; Figure S2B), cyclohexylamide (**65** and **69**; Figure S2B,C), and isobutyl and *tert*‐butyl (**71**; Figure S2D) groups would be involved.

Other previously described TcLAP inhibitors also bear hydrophobic chemical groups responsible for inhibition. S1 and S1′ sub‐sites have not been described for trypanosomatid M17 LAPs. In general, M17 LAPs exhibit an S1 pocket formed by hydrophobic and negative residues, and an S1′ sub‐site mainly formed by hydrophobic and positive amino acids [48]. Several interactions predicted here for the peptoid‐based compounds and TcLAP are coincident with those predicted for other inhibitors. For example, interactions between compounds **63**, **65**, or **69** with K300, D370, and with F304, T402, G403, A496, and F497 are also predicted for the peptidomimetic KBE009 (Figure 1) [24].

Finally, screening of this small peptoid library (Table 2) against the leishmanial enzyme LmLAP provided very relevant results, as four peptoids (**68**, **69**, **71**, and **73**) led to complete depletion of LmLAP enzymatic activity at 100 μM. As a result, they were chosen for the concentration‐dependent inhibition study. The IC_50_ values of peptoids **68**, **71**, and **73** were higher than 50 μM, but compound **69** showed an IC_50_ of 4.4 μM (Figure 5B). Accordingly, this latter peptoid can be considered as a moderate inhibitor, showing an LmLAP inhibition similar to that of bestatin [27].

**FIGURE 7:**
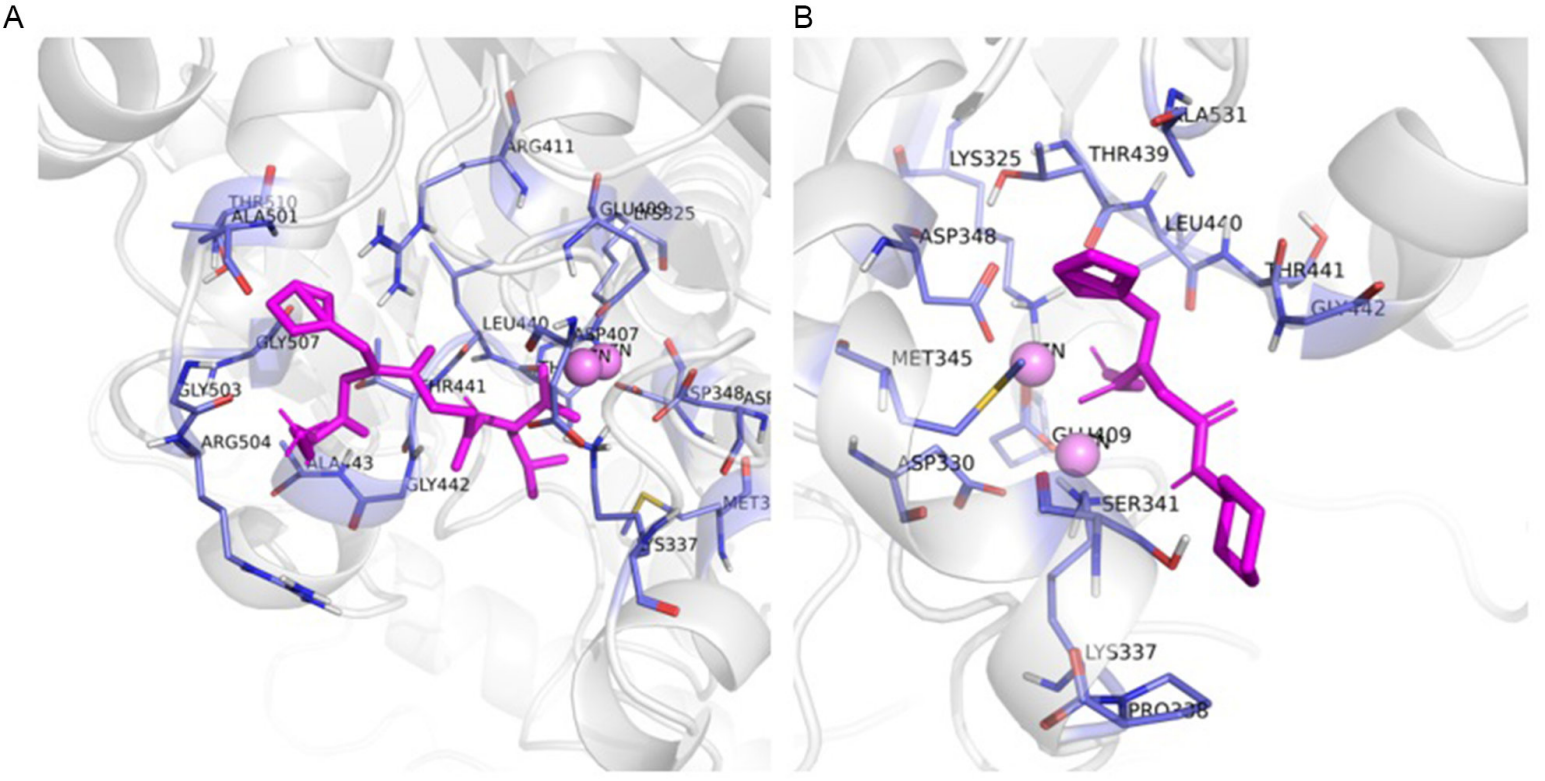
3D representation of molecular docking between compounds **46** (A) and **69** (B) with LmLAP. The best model, in terms of binding affinity, is shown for each inhibitor after 20 runs. Residues that interact with the inhibitors are represented. Nitrogen atoms are shown in blue and oxygen atoms in red. Manganese ions are represented as spheres.

We modeled the LmLAP inhibition by compounds **46** and **69** by molecular docking. Figure 7 shows the best model in terms of binding affinity for each inhibitor. For compound **46,** two best models with the same binding affinity were found, but only one is shown. Figure S3 (in the SI) presents the models depicted in Figure 7 in 2D perspective. Predictions suggest that the two inhibitors coordinate the Mn^2+^ cations in the active site (**46** with the carboxyl group and **69** with the terminal hydroxyl and carbonyl substituents) (Figures 7 and S3). However, like PfA‐M1 and TcLAP, hydrophobic interactions predominate in the stabilization of inhibitor‐enzyme complexes (Figure S3). In these interactions, the ethylamine (**46**; Figure S3A), 2‐methylfuryl (**46** and **69**; Figure S3A,B), isobutyl (**46**; Figure S3A), and cyclohexylamide (**69**; Figure S3B) groups would be involved. Several interactions predicted here between our compounds and LmLAP residues within the active site are consistent with those predicted for potent LmLAP inhibitors reported, like compound DDD00057570 [27] (not shown). For example, interactions between compound **46** and G507, R411; 69 and A531, D407, and E409 are predicted. Similarly, both compound **46** and **69** have interactions with K325, K337, M345, T439, L440, T441, and G442, as previously detected for DDD00057570 too [27].

## 3 Conclusions

We have developed synthetic approaches for the construction of two compound libraries, relying on the diversity‐ and complexity‐generating power of multicomponent reaction for the rapid assembly of peptoid‐based structures bearing hydrophobic substituents. The libraries were evaluated against three metallo‐aminopeptidases: PfA‐M1 from *Plasmodium falciparum*, TcLAP from *Trypanosoma cruzi* and LmLAP from *Leishmania major*, which are considered as potential therapeutic targets owing to their crucial roles in parasite development within the human host. The first library was constructed *via* an initial Ugi reaction to introduce two hydrophobic substituents onto a succinate‐based scaffold, followed by a coupling step with an α‐amino acid, which furnished a peptoid‐peptide hybrid skeleton with three diversity elements. This design of the first library does not focus on the installation of metal‐chelating moieties but rather on multiple hydrophobic substituents capable of effectively interacting with the enzyme binding sites. From this library, four compounds (**13**, **18**, **19**, and **25**) were identified that inhibit PfA‐M1 with IC_50_ values in the range 6.0–9.6 μM. Molecular docking suggests that, although compounds **18, 19**, and **25** could coordinate the Zn^2+^ cation, hydrophobic interactions predominate in stabilizing the inhibitor‐enzyme complexes.

The second library focuses on a glycolate‐based peptoid scaffold and incorporates a α‐hydroxycarbonyl moiety with a potential chelating capacity. Intriguingly, this library did not yield PfA‐M1 inhibitors, but it was successful in the discovery of TcLAP and LmLAP inhibitors. In general, five inhibitors of TcLAP with IC_50_ in the range of 1.0–11.7 μM and two inhibitors of LmLAP with IC_50_ in the range 3.6–4.4 μM were discovered. They can be considered moderate to potent inhibitors, with inhibition levels similar to those of other compounds described in the literature featuring more complex structures. A limitation of the second library is its size, because only a few compounds could be produced for biological screening. However, given the simplicity of this synthesis and the information obtained from this preliminary study, we project the construction of a much larger library for scaffold optimization.

Once more, the docking studies predicted that hydrophobic interactions are crucial for a good binding affinity to the three enzymes, which corresponds with what is reported in the literature. Our results offer new insights into the structure–activity relationship of inhibitors of the three parasite targets and supports the general consideration that, despite these being metallo‐enzymes, the presence of metal‐chelating groups might be favorable but not sufficient for potent activity. Indeed, a proper matching between the inhibitor’s hydrophobic substituents and relevant residues in the enzyme’s binding sites are necessary.

## Funding

This study was supported by the VLIRUOS (CU2018‐TEA458A101), German Academic Exchange Service (57592717), Alexander von Humboldt‐Stiftung, and Deutsche Forschungsgemeinschaft (390715994).

## Conflicts of Interest

The authors declare no conflicts of interest.

## Supporting information

Supplementary Material

## Data Availability

The authors declare that all the data supporting the findings of this study are available within the article and the Supporting Information file and also from the corresponding authors upon reasonable request.
