# Supplementary material for: Synthesis and Evaluation of Peptoid‐Based Compound Libraries as Inhibitors of Parasite Metallo‐Aminopeptidases
Source: ChemMedChem. 2026 Sep 10;21(17):e70433. doi: 10.1002/cmdc.70433 (PMC13562828; doi:10.1002/cmdc.70433)
Supplement: Supplementary file 1 [file CMDC-21-e70433-s001.pdf]

Supporting Information

Table of Content

1. Abbreviations	S3
2. General Information	S4
3. Chemical Synthesis	S4
4. (^{1}H, ^{13}C and ^{19}F) RMN and LC-MS characterization of compounds	S5
5. Biological evaluation	S30
6. Molecular docking	S39
7. NMR spectra of the final compounds	S43
8. References	S116

1. Abbreviations

AA: amino acid

AcOEt: ethyl acetate

Calcd: calculated

$CDCl_3$: deuterated chloroform

DCM: dichloromethane

DIPEA: *N,N*-diisopropilethylamine

DMF: dimethylformamide

DMSO: dimethylsulfoxide

DMSO-d6: hexadeuterated dimethylsulfoxide

ESI: Electrospray Ionization

HBTU: hexafluorophosphate benzotriazole tetramethyluronium salt

HPLC: High Performance Liquid Chromatography

LC-MS: Liquid Chromatography – Mass Spectrometry

M: molar, mol/L

MeOH: methanol

MeOH-d4: tetradeuterated methanol

NMR: Nuclear Magnetic Resonance

M17 LAP: M17-family Leucine Aminopeptidase

PfA-M1: *Plasmodium falciparum* M1-family Aminopeptidase 1

RCSB PDB: Research Collaboratory for Structural Bioinformatics Protein Data Bank

RT: room temperature

tBu: *tert*-butyl

TcLAP: *Trypanosoma cruzi* Leucine Aminopeptidase

TFA: trifluoroacetic acid

THF: tetrahydrofuran

TIS: tris(isopropyl)silane

TLC: Thin Layer Chromatography

TMS: trimethylsilane

2. General information

All starting materials were purchased from commercial sources and used without further purification. ^{1}H NMR,^{13}C NMR and ^{19}F NMR spectra were recorded on Bruker Avance 300 and Bruker Avance III HD 400 spectrometers at ambient temperature using $CDCl_3$, DMSO-d_6 or MeOH-d_4 as solvent. Chemical shifts (δ) are reported in ppm relative to the TMS (^{1}H NMR) or the solvent signal (^{13}C NMR). For thin layer chromatography, analytical TLC plates (Alugram G/UV254 and 70-230 mesh silica gel (Merck) were used). Column chromatography was performed using silica gel (Merck, 60-120 mesh size). Solvents for chromatography were used without previous distillation.

The purity of the compounds was determined by HPLC analysis and confirmed to be more than 95%. This was performed on a Shimadzu prominence-i lc-2030c 3D plus (HPLC system) with a quaternary gradient pump, column thermostat, and diode array detector. The system was equipped with an analytical column Shim-pack GISS-HP, 3 µm, C18, 2.1 x 100 mm. The mobile phase gradient consisted of pure methanol (D) and water (A, with 0,1% formic acid). The samples were measured according to the following method. First, a stabilization with a solvent ratio of 40% methanol (D) was performed for 8 min (DA_stab_40_8min). Then, the sample was injected in the column while the solvent ratio was increased in gradient to 100% methanol (D) during 5 min, followed by 5 min at 100% methanol (DA_40_100_100_5-5 min). Mass spectra were recorded on a Shimadzu LCMS-2020 (MS detector) with electrospray ionization (ESI) and single quadrupole mass analyzer.

3. Chemical synthesis

Construction of the library of succinate-based peptide-peptoid hybrids 1 to 64 following a four-step synthetic route.[1]

1) ***Ugi reaction.*** Paraformaldehyde (10 mmol, 300 mg), amine (10 mmol), monomethyl succinate (10 mmol, 1.32 g) and the isonitrile (10 mmol) were reacted in 20 mL of dry MeOH for a period of 24-72 h. After complete conversion (checked by TLC), the mixture was diluted with 50 mL of AcOEt and washed with aq. sat. $NaHCO_3$ (3 × 50 mL), aq. 5% HCl (3 × 50 mL) and brine (50 mL), then dried over anhydrous Na_2SO_4 and concentrated in vacuum. The crude material was triturated with *n*-hexane (3 × 50 mL) and used in the next step without further purification (Scheme 1).

2) Methyl ester hydrolysis. The crude material (~10 mmol) from the previous step was dissolved in 40 mL of THF/H_2O (3:1, *v/v*). and the solution was cooled with an ice bath. Then, LiOH (960 mg, 40 mmol) was added slowly and the reaction mixture was stirred until full conversion, as checked by TLC. Then, aq. 5 % HCl was added dropwise until pH 3-4 was reached, keeping the temperature low. The mixture was extracted with DCM (3 × 30 mL) followed and the organic phase were dried over anhydrous Na_2SO_4 and concentrated in vacuum. The crude product was used for the next step without further purification (Scheme 1).

3) Coupling to the α-amino acid. The crude material from the previous step (1 equiv.) and the corresponding α-amino acid *t*-butyl ester hydrochloride (HCl·H-AA-O^{t}Bu, 1.2 equiv) were dissolved in dry DMF at a concentration of 0.25 M. The stirring solution was cooled with an

ice bath and then, HBTU (1.2 equiv) and DIPEA (3.0 equiv) were added. The reaction mixture was allowed to reach RT and stirred overnight. After complete conversion (checked by TLC), the mixture was diluted with 50 mL of AcOEt and the organic phase was washed with aq. sat. $NaHCO_3$ (3 × 25 mL), aq. 5 % HCl (3 × 25 mL) and brine (25 mL), and then dried over anhydrous Na_2SO_4 and concentrated in vacuum. Column chromatography on silica gel using gradients of DCM/MeOH was performed to afford the pure peptide-peptide hybrid products.

4) ***Final deprotection.*** The pure product (0.25 mmol) from the previous step was dissolved in DCM (2 mL), water (75 μL) and TIS (75 μL). The solution was cooled with an ice bath and TFA (1 mL) was added dropwise. The reaction was allowed to proceed for 3 h at low temperature, then at RT overnight. After complete conversion (checked by TLC), the mixture was diluted with 25 mL of DCM and concentrated under vacuum. This process was repeated several times until TFA was fully removed. The crude material was purified by column chromatography on silica gel using gradients of DCM/MeOH with 1% of AcOH to afford the pure final products. The final peptide-peptoid hybrids were redissolved in acetonitrile/water 1:2 (*v/v*) and lyophilized.

Construction of the library of glycolate-based peptoids 65 to 74

The synthesis was carried out in a one-pot procedure. Paraformaldehyde (2 mmol, 60 mg), amine (2 mmol), glycolic acid (2 mmol, 102 μL) and isonitrile (2 mmol) were reacted in 5 mL of dry MeOH for a period of 24-48 h. After complete conversion (checked by TLC), the mixture was diluted with 25 mL of AcOEt. The organic phase was washed with aq. sat. $NaHCO_3$ (3 × 25 mL), aq. 5 % HCl (3 × 25 mL) and brine (25 mL), then dried over anhydrous Na_2SO_4 and concentrated under vacuum. Column chromatography on silica gel using gradients of DCM/MeOH was performed to afford the pure glycolate-based peptoids.

4. (^{1}H, ^{13}C and ^{19}F) RMN and LC-MS characterization of compounds

Compound 1. (4-((2-(cyclohexylamino)-2-oxoethyl)(isobutyl)amino)-4-oxobutanoyl)-L-alanine.

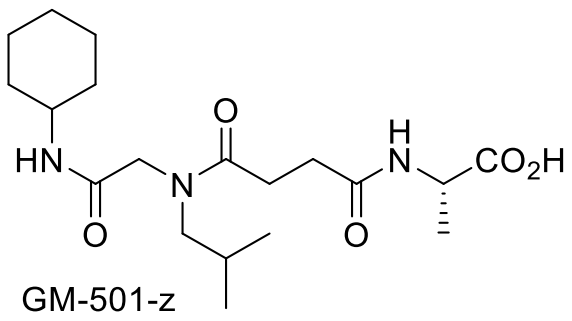

GM-501-z

Obtained as an off white solid, yield 29% (0.268 g). ^{1}H NMR (400 MHz, DMSO) δ 12.40 (s, 1H, CO_2H), [8.21 (d, *J* = 7.3 Hz), 8.13 (d, *J* = 7.3 Hz), 1H, NH], [7.90 (d, *J* = 7.8 Hz), 7.46 (d, *J* = 8.0 Hz), 1H, NH], [4.21 (p, *J* = 7.2 Hz), 4.16 (p, *J* = 7.3 Hz), 1H), [3.92 (s), 3.82 (s), 2H], 3.61-3.46 (m, 1H), 3.17-2.98 (m, 2H), 2.57-2.26 (m, 4H), 1.89-1.76 (m, 1H), 1.76-1.62 (m, 4H), 1.58-1.50 (m, 1H), [1.25 (d, *J* = 7.1 Hz), 1.23 (d, *J* = 7.1 Hz), 3H], 1.33-1.05 (m, 5H), [0.86 (d, *J* = 6.5 Hz), 0.79 (d, *J* = 6.7 Hz), 6H]. ^{13}C NMR (101 MHz, DMSO) δ 174.22, 174.19, 172.20, 172.15, 171.50, 171.37, 167.24, 167.12, 55.58, 53.77, 50.75, 49.32, 47.69, 47.48, 47.41, 32.38, 32.31, 30.49, 30.43, 27.90, 27.36, 27.26, 26.44, 25.17, 25.15, 24.66, 24.51, 20.05, 20.03, 19.80, 17.27, 17.22. Conformers detected. LC-MS (ESI, m/z) calcd for $C_{19}H_{34}N_3O_5^+$ $[M+H]^+$: 384.2493, found: 384.3.

Compound 2 (4-((2-(cyclohexylamino)-2-oxoethyl)(isobutyl)amino)-4-oxobutanoyl)-L-leucine

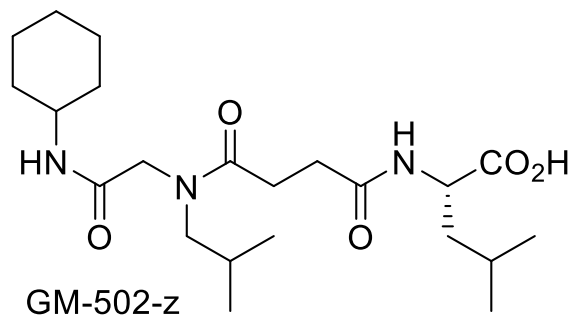
GM-502-z

Obtained as a white solid, yield 34% (0.356 g). ^{1}H NMR (400 MHz, DMSO) δ 12.45 (s, 1H, CO_2H), [8.15 (d, *J* = 7.9 Hz), 8.07 (d, *J* = 8.0 Hz), 1H, NH], [7.90 (d, *J* = 7.9 Hz), 7.47 (d, *J* = 8.1 Hz), 1H, NH], 4.26-4.17 (m, 1H), [3.95 (d, *J* = 17.4 Hz), 3.89 (d, *J* = 17.4 Hz), 3.85 (d, *J* = 16.2 Hz), 3.80 (d, *J* = 16.2 Hz), 2H], 3.61-3.45 (m, 1H), 3.17-2.95 (m, 2H), 2.55-2.27 (m, 4H), 1.92-1.43 (m , 9H), 1.32-1.05 (m, 5H), 0.92-0.74 (m, 12H). Conformers detected.

^{13}C NMR (101 MHz, DMSO) δ 174.20, 174.16, 172.19, 172.17, 171.78, 171.60, 167.26, 167.11, 55.59, 53.79, 50.77, 50.27, 50.12, 49.33, 47.70, 47.50, 32.38, 32.33, 32.30, 30.56, 30.49, 27.95, 27.38, 27.32, 26.45, 25.17, 25.13, 24.69, 24.52, 24.34, 24.24, 22.85, 22.75, 21.40, 21.34, 20.05, 20.02, 19.80. Conformers detected. LC-MS (ESI, m/z) calcd for $C_{22}H_{40}N_3O_5^+$ $[M+H]^+$: 426.2962, found: 426.4.

Compound 3 (4-((2-(cyclohexylamino)-2-oxoethyl)(isobutyl)amino)-4-oxobutanoyl)-L-phenylalanine

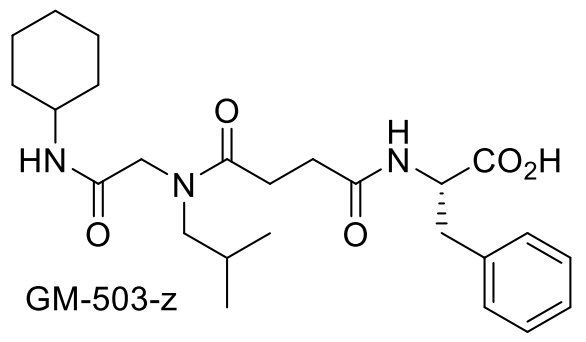
GM-503-z

Obtained as a white solid, yield 18% (0.203 g). ^{1}H NMR (400 MHz, DMSO) δ 12.55 (s, 1H, CO_2H), [8.25 (d, *J* = 8.1 Hz), 8.18 (d, *J* = 8.0 Hz), 1H, NH], [7.88 (d, *J* = 7.9 Hz), 7.46 (d, *J* = 8.0 Hz), 1H, NH], 7.33-7.16 (m, 5H), 4.46-4.35 (m, 1H), [3,89 (s), 3.81 (s), 2H], 3.60-3.44 (m, 1H), [3.12-2.97 (m), 2.90-2.79 (m), 4H], 2.45-2.27 (m, 4H), 1.88-1.75 (m, 1H), 1.75-1.61 (m, 4H), 1.58-1.49 (m, 1H), 1.32-1.04 (m, 5H), [0.85 (d, *J* = 6.6 Hz), 0.78 (d, *J* = 6.7 Hz), 6H]. Conformers detected. ^{13}C NMR (101 MHz, DMSO) δ 173.03, 173.01, 172.13, 172.00, 171.68, 171.54, 167.17, 167.10, 137.69, 137.65, 129.10, 128.12, 126.39, 126.34, 55.54, 53.74, 53.50, 53.46, 50.71, 49.26, 47.69, 47.48, 40.14, 39.94, 39.73, 39.52, 39.31, 39.10, 38.89, 36.92, 36.87, 32.37, 32.32, 30.58, 27.94, 27.41, 27.30, 26.42, 25.16, 24.65, 24.51, 20.04, 19.78. LC-MS (ESI, m/z) calcd for $C_{25}H_{38}N_3O_5^+$ $[M+H]^+$: 460.2806, found: 460.3.

Compound 4 (4-((2-(cyclohexylamino)-2-oxoethyl)(isobutyl)amino)-4-oxobutanoyl)-L-proline

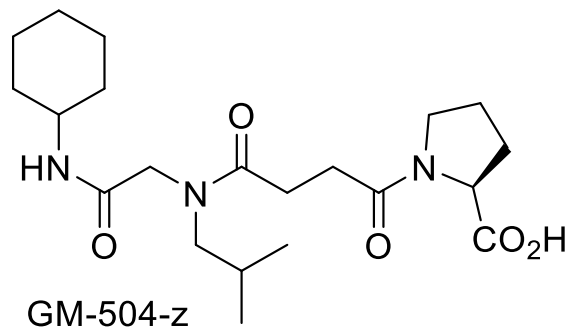
GM-504-z

Obtained as a white solid, yield 39% (0.393 g). ^{1}H NMR (400 MHz, DMSO) δ 12.48 (s, 1H, CO_2H), [7.92 (d, *J* = 7.9 Hz), 7.90 (d, *J* = 7.9 Hz), 7.43 (d, *J* = 8.1 Hz), 7.38 (d, *J* = 8.1 Hz), 1H, NH], [4.54 (dd, *J* = 6.7 Hz, *J* = 2.6 Hz), 4.51 (dd, *J* = 6.9 Hz, *J* = 2.6 Hz), 4.22 (dd, *J* = 8.8, 3.2 Hz), 4.19 (dd, *J* = 8.7, 3.4 Hz), 1H], 3.98-3.68 (m, 2H), 3.60-3.32 (3H), 3.25-2.96 (m, 2H), 2.74-2.00 (m, 6H), 1.96-1.76 (m, 3H), 1.76-1.60 (m, 4H), 1.59 (m, 1H), 1.32-1.04 (m, 5H), [0.88 (d, *J* = 6.3 Hz), 0.87 (d, *J* = 6.2 Hz), 0.79 (d, *J* = 6.6 Hz), 0.78 (d, *J* = 6.7 Hz), 6H]. Conformers detected. ^{13}C NMR (101 MHz, DMSO) δ 173.82, 173.73, 173.46, 173.37, 172.24, 172.20, 170.68, 170.43, 170.31, 170.11, 167.27, 167.23, 167.14, 58.67, 58.36, 58.30, 55.63, 53.83, 50.97, 50.84, 49.44, 47.71, 47.57, 47.50, 46.42, 46.37, 46.04, 32.41, 32.30, 32.27, 32.19, 30.87, 29.56, 29.33, 29.18, 28.89, 28.85, 27.68, 27.46, 27.25, 27.16, 26.74, 26.46, 25.26, 25.15, 25.04, 24.74, 24.71, 24.66, 24.55, 24.52, 24.28, 24.23, 22.24, 21.05, 20.09, 20.07, 20.05, 19.86, 19.84, 19.80. Conformers detected. LC-MS (ESI, m/z) calcd for $C_{21}H_{36}N_3O_5^+$ $[M+H]^+$: 410.2649, found: 410.3.

Compound 5 (4-((2-(cyclohexylamino)-2-oxoethyl)(pentyl)amino)-4-oxobutanoyl)-L-alanine

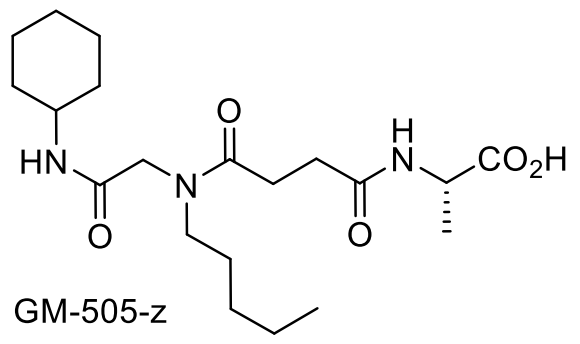

Obtained as a light-yellow solid, yield 28% (0.369 g). ^{1}H NMR (400 MHz, DMSO) δ 12.42 (s, 1H, CO_2H), [8.20 (d, *J* = 7.3 Hz), 8.13 (d, *J* = 7.3 Hz), 1H, NH], [7.91 (d, *J* = 7.8 Hz), 7.46 (d, *J* = 8.0 Hz), 1H, NH], [4.20 (p, *J* = 7.3 Hz), 4.17 (p, *J* = 7.2 Hz), 1H], [3.93 (d, *J* = 17.2 Hz), 3.88 (d, *J* = 17.2 Hz), 3.82 (s), 2H], 3.60-3.46 (m, 1H), 3.29-3.13 (m, 2H), 2.57-2.28 (m, 4H), 1.76-1.62 (m, 4H), 1.58-1.46 (m, 2H), 1.42-1.33 (m, 1H), [1.25 (d, *J* = 7.4 Hz), 1.24 (d, *J* = 7.4 Hz), 3H] (1.32-1.05 (m, 9H), [0.87 (t, *J* = 7.0 Hz), 0.84 (t, *J* = 7.2 Hz), 3H]. Conformers detected. ^{13}C NMR (101 MHz, DMSO) δ 174.23, 174.19, 171.75, 171.66, 171.48, 171.37, 167.32, 167.26, 50.34, 48.76, 48.48, 47.67, 47.48, 47.41, 46.56, 32.36, 32.30, 30.40, 30.32, 28.54, 28.36, 27.84, 27.79, 27.05, 26.72, 25.17, 24.65, 24.51, 21.95, 21.92, 17.27, 17.23, 13.89. Conformers detected. LC-MS (ESI, m/z) calcd for $C_{20}H_{36}N_3O_5^+$ $[M+H]^+$: 398.2649, found: 398.3.

Compound 6 (4-((2-(cyclohexylamino)-2-oxoethyl)(pentyl)amino)-4-oxobutanoyl)-L-leucine

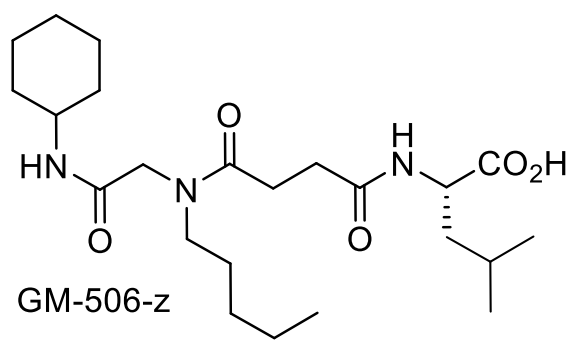

Obtained as an off-white solid, yield 33% (0.451 g). ^{1}H NMR (400 MHz, DMSO) δ 12.44 (s, 1H, CO_2H), [8.15 (d, *J* = 7.9 Hz), 8.07 (d, *J* = 7.9 Hz), 1H, NH], [7.91 (d, *J* = 7.7 Hz), 7.46 (d, *J* = 8.0 Hz), 1H, NH], 4.27-4.16 (m, 1H), [3.93 (d, *J* = 17.2 Hz), 3.93 (d, *J* = 17.2 Hz), 3.82(s), 2H], 3.62-3.43 (m, 1H), 3.30-3.11 (m, 2H), 2.55-2.27 (m, 4H), 1.76-1.59 (m, 5H), 1.58-1.43 (m, 4H), 1.42-1.33 (m, 1H), 1.32-1.06 (m, 9H), 0.92-0.80 (m, 9H). Conformers detected. ^{13}C NMR (101 MHz, DMSO) δ 174.20, 174.16, 171.75, 171.72, 171.67, 171.61, 167.34, 167.24, 50.37, 50.22, 50.13, 48.79, 48.50, 47.68, 47.49, 46.61, 32.36, 32.31, 32.28, 30.47, 30.40, 28.53, 28.36, 27.90, 27.80, 27.15, 26.72, 25.17, 25.14, 24.68, 24.52, 24.33, 24.25, 22.84, 22.76, 21.96, 21.92, 21.37, 21.35, 13.88. Conformers detected. LC-MS (ESI, m/z) calcd for $C_{23}H_{42}N_3O_5^+$ $[M+H]^+$: 440.3119, found: 440.4.

Compound 7 (4-((2-(cyclohexylamino)-2-oxoethyl)(pentyl)amino)-4-oxobutanoyl)-L-phenylalanine

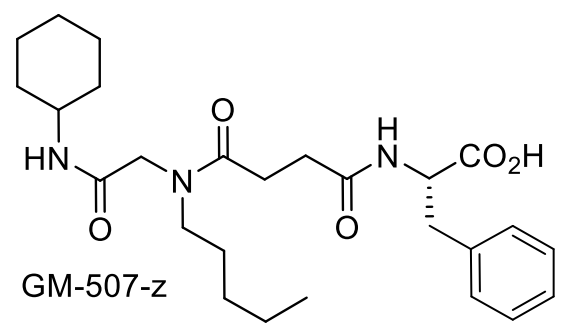

Obtained as a white solid, yield 27% (0.416 g). ^{1}H NMR (400 MHz, DMSO) δ 12.64 (s, 1H, CO_2H), [8.25 (d, *J* = 8.1 Hz), 8.18 (d, *J* = 8.0 Hz), 1H, NH], [7.90 (d, *J* = 7.9 Hz), 7.47 (d, *J* = 8.0 Hz), 1H, NH], 7.31-7.17 (m, 5H), [4.44 (dt, *J* = 8.2, 4.3 Hz), 4.39 (dt, *J* = 9.2, 4.4 Hz), 1H], [3.88 (s), 3.82 (s), 2H], 3.60-3.45 (m, 1H), 3.25-3.14 (m, 2H), [3.08 (m), 2.99 (m), 2H], 2.46-2.27 (m, 4H), 1.76-1.61 (m, 4H), 1.58-1.44 (m, 2H), 1.42-1.33 (m, 1H), 1.32-1.05 (m, 9H), [0.87 (t, *J* = 7.2 Hz), 0.84 (t, *J* = 7.2 Hz), 3H]. Conformers detected. ^{13}C NMR (101 MHz, DMSO) δ 173.03, 173.00, 171.67, 171.55, 171.53, 167.26, 167.23, 137.68, 137.66, 129.10, 128.12, 126.38, 126.35, 53.47, 50.30, 48.71, 48.43, 47.68, 47.48, 46.55, 36.92, 36.88, 32.35, 32.30, 30.49, 28.53, 28.36, 27.90, 27.76, 27.20, 26.70, 25.17, 24.65, 24.51, 21.95, 21.92, 13.89. Conformers detected. LC-MS (ESI, m/z) calcd for $C_{26}H_{40}N_3O_5^+$ $[M+H]^+$: 474.2962, found: 474.3.

Compound 8 (4-((2-(cyclohexylamino)-2-oxoethyl)(pentyl)amino)-4-oxobutanoyl)-L-proline

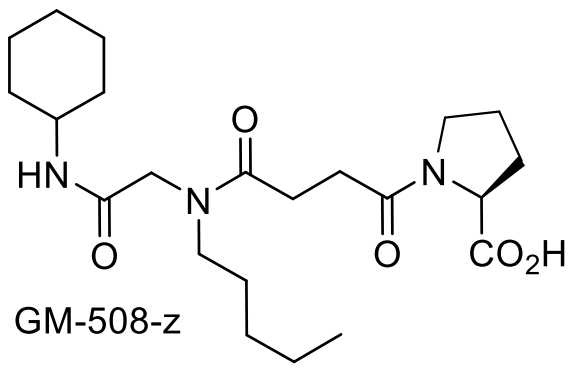

Obtained as a white solid, yield 38% (0.418 g). ^{1}H NMR (400 MHz, DMSO) δ 12.47 (s, 1H, CO_2H), [7.93 (d, *J* = 7.6 Hz), 7.91 (d, *J* = 6.8 Hz), 7.43 (d, *J* = 8.1 Hz), 7.38 (d, *J* = 8.1 Hz), 1H, NH], [4.54 (dd, *J* = 6.3 Hz, *J* = 2.7 Hz), 4.51 (dd, *J* = 6.1 Hz, *J* = 2.8 Hz), 4.21 (dd, *J* = 8.7, 3.4 Hz), 4.19 (dd, *J* = 8.7, 3.3 Hz), 1H], 3.96-3.70 (m, 2H), 3.59-3.46 (m, 1H), 3.41-3.12 (m, 4H), 2.72-2.31 (m, 4H), 2.30-1.77 (m, 4H), 1.76-1.61 (m, 4H), 1.59-1.47 (m, 2H), 1.44-1.33 (m, 1H), 1.32-1.06 (m, 9H), [0.87 (t, *J* = 7.0 Hz), 0.84 (t, *J* = 7.1 Hz), 3H]. Conformers detected. ^{13}C NMR (101 MHz, DMSO) δ 173.75, 173.46, 173.38, 171.74, 170.64, 170.28, 170.12, 167.34, 167.31, 167.28, 58.67, 58.34, 58.30, 50.54, 48.87, 48.54, 47.69, 47.55, 46.61, 46.41, 46.37, 46.00, 32.38, 32.28, 32.24, 32.19, 30.87, 29.40, 29.27, 29.12, 28.89, 28.85, 28.55, 28.37, 27.83, 27.22, 26.72, 26.53, 25.15, 25.05, 24.69, 24.54, 24.28, 24.24, 22.24, 21.95, 21.93, 13.89. Conformers detected. LC-MS (ESI, m/z) calcd for $C_{22}H_{38}N_3O_5^+$ [M+H]$^+$: 424.2806, found: 424.4.

Compound 9 (4-(benzyl(2-(cyclohexylamino)-2-oxoethyl)amino)-4-oxobutanoyl)-L-alanine

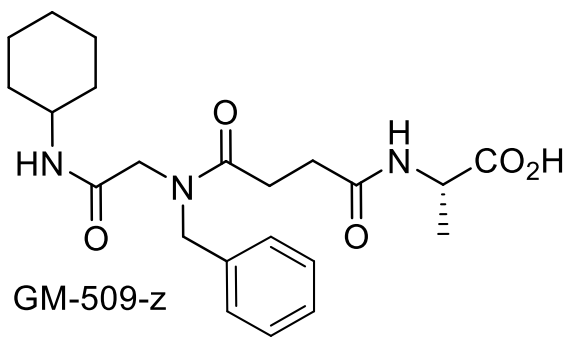

Obtained as a white solid, yield 15% (0.212 g). ^{1}H NMR (400 MHz, DMSO) δ 12.47 (s, 1H, CO_2H), [8.19 (d, *J* = 8.3 Hz), 8.17 (d, *J* = 7.8 Hz), 1H, NH], [7.85 (d, *J* = 7.9 Hz), 7.54 (d, *J* = 8.0 Hz), 1H, NH], 7.40-7.17 (m, 5H), [4.60 (s), 4.48 (d, *J* = 15.2 Hz), 4.42 (d, *J* = 15.2 Hz), 2H], [4.20 (p, *J* = 7.3 Hz), 4.19 (p, *J* = 7.3 Hz), 1H], [3.90 (d, *J* = 17.4 Hz), 3.84 (d, *J* = 17.4 Hz), 3.80 (s), 2H], 3.58-3.46 (m, 1H), 2.63-2.35 (m, 4H), 1.74-1.60 (m, 4H), 1.58-1.48 (m, 1H), 1.25 (d, *J* = 7.2 Hz, 3H), 1.31-1.05 (m, 5H). Conformers detected. ^{13}C NMR (101 MHz, DMSO) δ 174.25, 174.23, 172.43, 172.24, 171.35, 171.30, 166.83, 166.81, 137.68, 137.28, 128.66, 128.32, 127.60, 127.27, 126.94, 126.82, 51.48, 49.73, 49.25, 48.50, 47.69, 47.52, 47.46, 47.44, 32.39, 32.34, 32.28, 30.43, 30.24, 27.69, 27.43, 25.17, 24.63, 24.49, 17.30, 17.27. Conformers detected. LC-MS (ESI, m/z) calcd for $C_{22}H_{32}N_3O_5^+$ [M+H]$^+$: 418.2336, found: 418.3.

Compound 10 (4-(benzyl(2-(cyclohexylamino)-2-oxoethyl)amino)-4-oxobutanoyl)-L-leucine

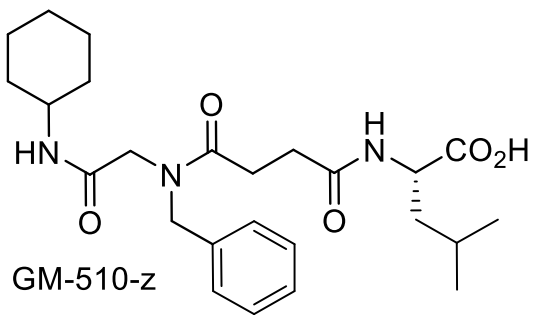

Obtained as a white solid, yield 24% (0.377 g). ^{1}H NMR (400 MHz, DMSO) δ 12.45 (s, 1H, CO_2H), [8.14 (d, *J* = 7.9 Hz, 2H), 8.11 (d, *J* = 8.4 Hz), 1H, NH], [7.85 (d, *J* = 7.8 Hz), 7.54 (d, *J* = 8.0 Hz), 1H, NH], 7.39-7.17 (m, 5H), [4.59 (s), 4.49 (d, *J* = 15.2 Hz), 4.41 (d, *J* = 15.2 Hz), 2H], 4.27-4.18 (m 1H), [3.87 (s), 3.87(s), 3.81 (s), 2H], 3.57-3.46 (m, 1H), 2.61-2.36 (m, 4H), 1.75-1.59 (m, 5H), 1.58-1.43 (m, 3H), 1.32-1.05 (m, 5H), [0.89 (d, *J* = 6.6 Hz, 1H), 0.84 (d, *J* = 6.5 Hz), 0.84 (d, *J* = 6.5 Hz), 6H]. Conformers detected. ^{13}C NMR (101 MHz, DMSO) δ 174.21, 174.18, 172.40, 172.25, 171.62, 171.53, 166.82, 137.69, 137.28, 128.64, 128.31, 127.63, 127.27, 126.94, 126.83, 51.50, 50.24, 50.16, 49.72, 49.27, 48.53, 47.69, 47.52, 32.39, 32.34, 32.29, 32.26, 30.47, 30.30, 27.75, 27.51, 25.16, 24.64, 24.49, 24.31, 24.27, 22.85, 22.78, 21.40, 21.37. Conformers detected. LC-MS (ESI, m/z) calcd for $C_{25}H_{38}N_3O_5^+$ [M+H]$^+$: 460.2806, found: 460.3.

Compound 11 (4-(benzyl(2-(cyclohexylamino)-2-oxoethyl)amino)-4-oxobutanoyl)-L-phenylalanine

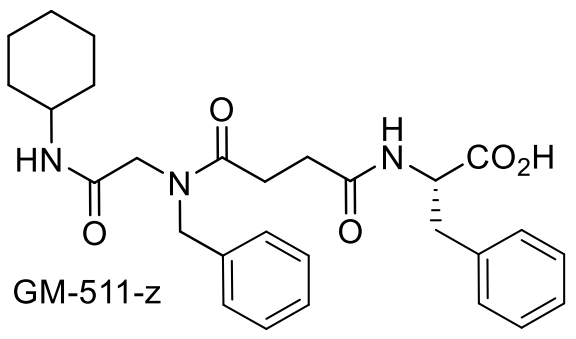
GM-511-z

Obtained as a white solid, yield 13% (0.210 g). ^{1}H NMR (400 MHz, DMSO) δ 12.55 (s, 1H, CO_2H), [8.24 (d, *J* = 8.2 Hz), 8.22 (d, *J* = 8.1 Hz), 1H, NH], [7.84 (d, *J* = 7.8 Hz), 7.54 (d, *J* = 8.0 Hz), 1H, NH], 7.39-7.15 (m, 10H), [4.55 (s), 4.44 (s), 2H], 4.46-4.38 (m, 1H), [3.84 (s), 3.79 (s), 2H], 3.58-3.45 (m, 1H), [3.04 (dd, *J* = 13.7, 5.1 Hz), 3.03 (dd, *J* = 13.8, 5.2 Hz), 2.85 (dd, *J* = 13.8, 9.1 Hz), 2.84 (dd, *J* = 13.8, 9.1 Hz), 2H], 2.50-2.32 (m, 4H), 1.73-1.61 (m, 4H), 1.58-1.49 (m, 1H), 1.31-1.04 (m, 5H). Conformers detected. ^{13}C NMR (101 MHz, DMSO) δ 173.04, 173.01, 172.35, 172.12, 171.53, 171.48, 166.80, 166.74, 137.69, 137.65, 137.63, 137.22, 129.12, 129.10, 128.65, 128.32, 128.12, 127.63, 127.27, 126.95, 126.78, 126.36, 53.50, 51.44, 49.66, 49.21, 48.45, 47.70, 47.53, 36.91, 32.38, 32.35, 32.28, 30.51, 30.39, 27.75, 27.56, 25.17, 24.62, 24.49. Conformers detected. LC-MS (ESI, m/z) calcd for $C_{28}H_{36}N_3O_5^+$ $[M+H]^+$: 494.2649, found: 494.3.

Compound 12 (4-(benzyl(2-(cyclohexylamino)-2-oxoethyl)amino)-4-oxobutanoyl)-L-proline

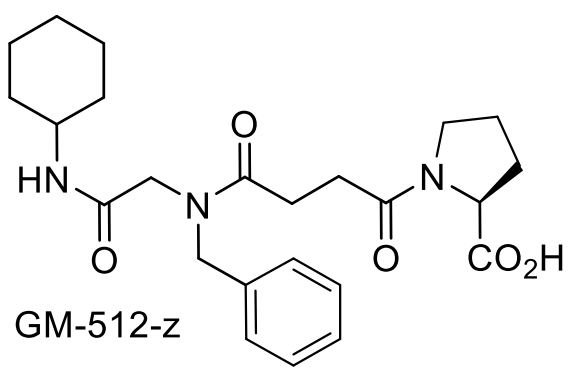
GM-512-z

Obtained as a white solid, yield 25% (0.388 g). ^{1}H NMR (400 MHz, DMSO) δ 12.44 (s, 1H, CO_2H), [7.87 (d, *J* = 7.7 Hz), 7.85 (d, *J* = 7.1 Hz), 7.51 (d, *J* = 7.9 Hz), 7.48 (d, *J* = 8.3 Hz,), 1H, NH], 7.40-7.18 (m, 5H), 4.69-4.19 (m, 3H), 3.95-3.72 (m, 2H), 3.60-3.35 (m, 3H), 2.71-2.41 (m, 4H), 2.30-1.78 (m, 4H), 1.75-1.60 (m, 4H), 1.58-1.48 (m, 1H). Conformers detected. ^{13}C NMR (101 MHz, DMSO) δ 173.82, 173.75, 173.46, 173.41, 172.46, 172.29, 170.38, 170.16, 170.06, 166.84, 166.82, 166.79, 137.65, 137.30, 128.64, 128.32, 127.65, 127.27, 126.93, 126.88, 58.69, 58.33, 51.51, 49.90, 49.27, 48.55, 47.70, 47.57, 46.40, 46.04, 38.25, 32.41, 32.26, 30.87, 29.36, 29.28, 28.88, 27.50, 27.09, 26.94, 25.15, 25.10, 24.66, 24.51, 24.29, 24.23, 22.25, 21.05. Conformers detected. LC-MS (ESI, m/z) calcd for $C_{24}H_{34}N_3O_5^+$ $[M+H]^+$: 444.2493, found: 444.3.

Compound 13 (4-((2-(cyclohexylamino)-2-oxoethyl)(furan-2-ylmethyl)amino)-4-oxobutanoyl)-L-alanine

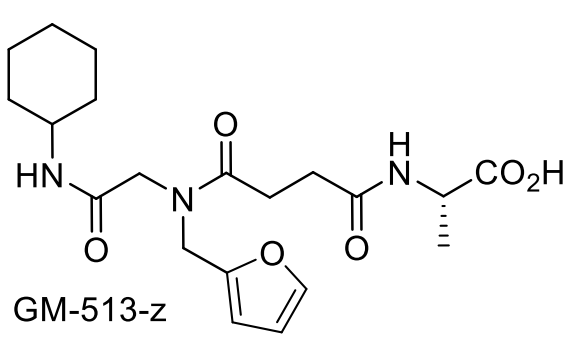
GM-513-z

Obtained as a white solid, yield 17% (0.197 g). ^{1}H NMR (400 MHz, DMSO) δ 12.46 (m, 1H, CO_2H), [8.20 (d, *J* = 7.3 Hz), 8.15 (d, *J* = 7.3 Hz), 1H, NH], [7.84 (d, *J* = 7.9 Hz), 7.51 (d, *J* = 8.0 Hz), 1H, NH], [7.63 (dd, *J* = 1.8, 0.9 Hz), 7.56 (dd, *J* = 1.8, 0.9 Hz), 1H], [6.42 (dd, *J* = 3.2, 1.8 Hz), 6.38 (dd, *J* = 3.2, 1.8 Hz), 1H], [6.40 (d, *J* = 3.2 Hz), 6.27 (d, *J* = 3.2 Hz), 1H], [4.54 (s), 4.48 (d, *J* = 15.5 Hz), 4.41 (d, *J* = 15.5 Hz), 2H], [4.20 (p, *J* = 7.3 Hz), 4.17 (p, *J* = 7.3 Hz), 1H], [3.90 (s), 3.80 (s), 2H], 3.57-3.45 (m, 1H), 2.78-2.61 (m , 1H), 2.50-2.30 (m, 3H), 1.74-1.63 (m, 4H), 1.58-1.50 (m, 1H), [1.25 (d, *J* = 7.3 Hz), 1.24 (d, *J* = 7.4 Hz), 3H], 1.30-1.06 (m, 5H). Conformers detected. ^{13}C NMR (101 MHz, DMSO) δ 174.23, 172.05, 171.90, 171.31, 171.21, 166.77, 166.68, 150.79, 150.52, 143.01, 142.55, 110.52, 110.42, 108.40, 49.38, 48.01, 47.70, 47.50, 47.44, 44.79, 42.25, 32.39, 32.34, 32.32,

30.25, 27.73, 27.49, 25.17, 24.62, 24.51, 17.28, 17.25. Conformers detected. LC-MS (ESI, m/z) calcd for $C_{20}H_{30}N_3O_6^+$ $[M+H]^+$: 408.2129, found: 408.3.

Compound 14 (4-((2-(cyclohexylamino)-2-oxoethyl)(furan-2-ylmethyl)amino)-4-oxobutanoyl)-L-leucine

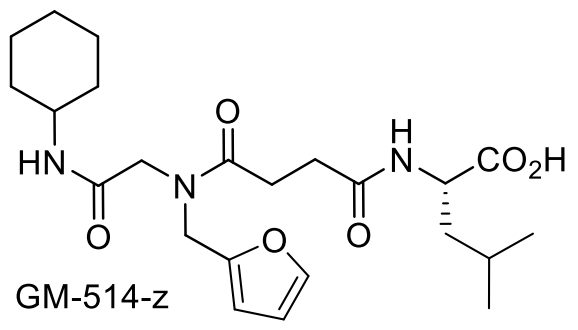

GM-514-z

Obtained as a white solid, yield 14% (0.170 g). ^1H NMR (400 MHz, DMSO) δ 12.47 (s, 1H, CO_2H), [8.14 (d, *J* = 7.9 Hz), 8.09 (d, *J* = 8.0 Hz), 1H, NH], [7.84 (d, *J* = 7.9 Hz), 7.51 (d, *J* = 8.0 Hz), 1H, NH], [7.62 (dd, *J* = 1.8, 0.8 Hz), 7.56 (dd, *J* = 1.9, 0.8 Hz), 1H], [6.39 (d, *J* = 3.4 Hz), 6.27 (d, *J* = 3.1 Hz), 1H], [6.42 (dd, *J* = 3.2, 1.8 Hz), 6.38 (dd, *J* = 3.2, 1.9 Hz), 1H], [4.54 (s), 4.47 (d, *J* = 15.5 Hz), 4.42 (d, *J* = 15.5 Hz), 2H], 4.26-4.18 (m, 1H), [3.90 (s), 3.80 (s), 2H], 3.57- 3.45 (m, 1H), 2.75-2.61 (m, 1H), 2.50-2.32 (m, 3H), 1.75-1.59 (m, 5H), 1.58-1.44 (m, 3H), 1.30-1.05 (m, 5H), [0.89 (d, *J* = 6.7 Hz), 0.88 (d, *J* = 6.6 Hz), 0.84 (d, *J* = 6.5 Hz), 0.83 (d, *J* = 6.6 Hz), 6H].Conformers detected. ^{13}C NMR (101 MHz, DMSO) δ 174.21, 172.03, 171.90, 171.57, 171.45, 166.76, 166.70, 150.80, 150.52, 142.99, 142.53, 110.51, 110.41, 108.41, 108.37, 50.26, 50.18, 49.39, 48.03, 47.70, 47.51, 44.79, 42.28, 32.38, 32.32, 32.28, 30.30, 27.81, 27.56, 25.16, 24.63, 24.51, 24.31, 24.27, 22.84, 22.78, 21.41, 21.38. Conformers detected. LC-MS (ESI, m/z) calcd for $C_{23}H_{36}N_3O_6^+$ $[M+H]^+$: 450.2599, found: 450.3.

Compound 15 (4-((2-(cyclohexylamino)-2-oxoethyl)(furan-2-ylmethyl)amino)-4-oxobutanoyl)-L-phenylalanine

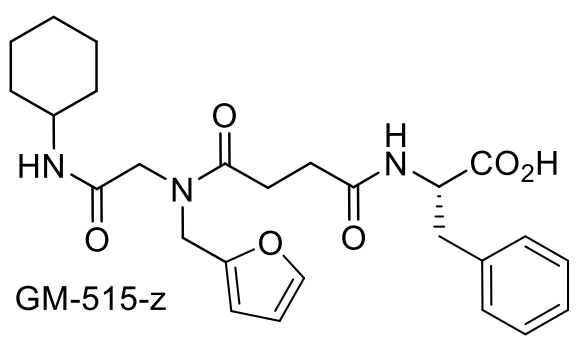

GM-515-z

Obtained as a white solid, yield 25% (0.335 g). ^1H NMR (400 MHz, DMSO) δ 12.68 (s, 1H, CO_2H), [8.25 (d, *J* = 8.1 Hz), 8.20 (d, *J* = 8.1 Hz), 1H, NH], [7.83 (d, *J* = 7.9 Hz), 7.51 (d, *J* = 8.0 Hz), 1H, NH], [7.62 (dd, *J* = 1.8, 0.8 Hz), 7.55 (dd, *J* = 1.9, 0.9 Hz), 1H], 7.29-7.16 (m, 5H), [6.42 (dd, *J* = 3.2, 1.9 Hz), 6.39-6.36 (m), 6.27 (d, *J* = 3.1 Hz), 2H], [4.49 (s), 4.45 (d, *J* = 15.2 Hz), 4.41 (d, *J* = 15.2 Hz), 2H], 4.46-4.37 (m, 1H), [3.87 (s), 3.79 (s), 2H], 3.58-3.45 (m, 1H), [3.04 (dd, *J* = 13.7, 5.1 Hz), 3.03 (dd, *J* = 13.8, 5.1 Hz), 2.84 (dd, *J* = 13.7, 9.2 Hz), 2.84 (dd, *J* = 13.7, 9.2 Hz) , 2H], [2.61 (t, *J* = 7.1 Hz), 2.44-2.30 (m), 4H], 1.75-1.62 (m, 4H), 1.58-1.50 (m, 1H), 1.31-1.05 (m, 5H). Conformers detected. ^{13}C NMR (101 MHz, DMSO) δ 173.03, 171.97, 171.79, 171.50, 171.39, 166.75, 166.61, 150.76, 150.47, 143.00, 142.55, 137.68, 137.65, 129.12, 128.13, 126.38, 126.36, 110.51, 110.42, 108.43, 108.40, 53.50, 53.47, 49.33, 47.98, 47.71, 47.51, 44.77, 42.21, 40.15, 39.94, 39.73, 39.62, 39.52, 39.31, 39.10, 38.89, 36.93, 36.90, 32.38, 32.36, 32.31, 30.40, 30.35, 27.81, 27.62, 25.17, 24.62, 24.51. Conformers detected. LC-MS (ESI, m/z) calcd for $C_{26}H_{34}N_3O_6^+$ $[M+H]^+$: 484.2442, found: 484.3.

Compound 16 (4-((2-(cyclohexylamino)-2-oxoethyl)(furan-2-ylmethyl)amino)-4-oxobutanoyl)-L-proline

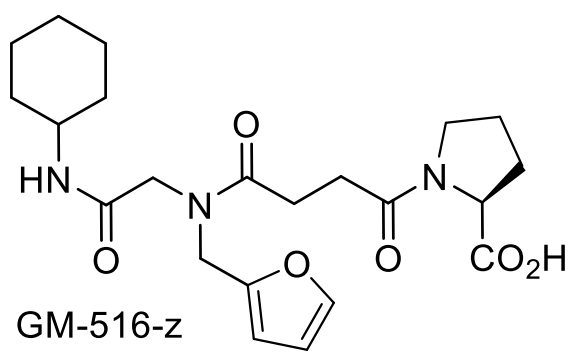

GM-516-z

Obtained as a light brown solid, yield 35% (0.420 g). ^1H NMR (400 MHz, DMSO) δ 12.37 (s, 1H, CO_2H), [7.85 (d, *J* = 7.9 Hz), 7.84 (d, *J* = 7.9 Hz), 7.49 (d, *J* = 7.9 Hz),7.45 (d, *J* = 8.1 Hz), 1H, NH], [7.63 (d, *J* = 1.0 Hz), 7.62 (d, *J* = 1.3 Hz), 7.56 (d, *J* = 0.9 Hz), 7.56 (d, *J* = 0.9 Hz), 1H], 6.43-6.40 (m, 1H), [6.38 (d, *J* = 3.2 Hz), 6.38 (d, *J* = 3.2 Hz),

6.28 (d, *J* = 3.3 Hz), 6.27 (d, *J* = 3.3 Hz), 1H], [4.62-4.37 (m), 4.24-4.19 (m, 3H)], 3.94-3.72 (m, 2H), [3.54-3.45 (m), 4.41-3.34 (m), 3H], 2.85-2.36 (m, 4H), 2.30-2.01 (m, 2H), 1.95-1.79 (m, 2H), 1.76-1.62 (m, 4H), 1.58-1.49 (m, 1H), 1.30-1.06 (m, 5H). Conformers detected. ^{13}C NMR (101 MHz, DMSO) δ 173.82, 173.79, 173.47, 173.43, 172.09, 172.01, 171.99, 170.46, 170.31, 170.10, 169.98, 166.78, 166.69, 150.76, 150.54, 142.99, 142.55, 110.52, 110.43, 108.45, 108.42, 58.69, 58.36, 58.32, 49.55, 48.12, 47.72, 47.55, 46.42, 46.39, 46.04, 44.82, 42.26, 32.40, 32.29, 32.26, 30.87, 29.25, 29.17, 28.89, 28.86, 27.20, 26.97, 25.15, 25.10, 24.65, 24.54, 24.28, 24.26, 22.26, 21.05, 17.85, 12.08. Conformers detected. LC-MS (ESI, m/z) calcd for $C_{22}H_{32}N_3O_6^+$ [M+H]$^+$: 434.2286, found: 434.3.

Compound 17 (4-((2-(tert-butylamino)-2-oxoethyl)(isobutyl)amino)-4-oxobutanoyl)-L-alanine

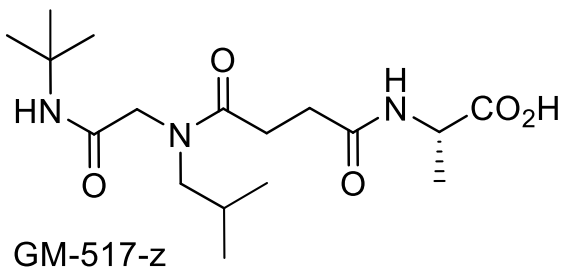

Obtained as a white solid, yield 24% (0.159 g). ^{1}H NMR (400 MHz, DMSO) δ 12.49 (s, 1H, CO_2H), [8.13 (d, *J* = 7.4 Hz), 8.10 (d, *J* = 7.3 Hz), 1H, NH], [7.62 (s), 7.21 (s), 1H, NH], [4.17 (p, *J* = 7.4 Hz), 4.15 (t, *J* = 7.3 Hz), 1H], [3.90 (d, *J* = 17.4 Hz), 3.85 (d, *J* = 17.3 Hz), 3.78 (s), 2H], 3.12-2.97 (m, 2H), 2.59-2.28 (m, 4H), 1.91-1.74 (m, 1H), [1.26 (s), 1.23 (s), 9H], [1.24 (d, *J* ≈ 7 Hz), 1.23 (d, *J* = 7.3 Hz), 3H], [0.87 (d, *J* = 6.6 Hz, 1H), 0.79 (d, *J* = 6.7 Hz), 0.79 (d, *J* = 6.7 Hz), 6H]. Conformers detected. ^{13}C NMR (101 MHz, DMSO) δ 174.47, 172.45, 172.15, 171.53, 171.50, 167.88, 167.82, 55.73, 54.02, 51.21, 50.51, 50.29, 49.76, 47.69, 30.73, 30.52, 28.73, 28.62, 28.13, 27.69, 27.45, 26.68, 20.26, 20.24, 20.04, 17.55, 17.49. Conformers detected. Conformers detected. LC-MS (ESI, m/z) calcd for $C_{17}H_{32}N_3O_5^+$ [M+H]$^+$: 358.2336, found: 358.3.

Compound 18 (4-((2-(tert-butylamino)-2-oxoethyl)(isobutyl)amino)-4-oxobutanoyl)-L-leucine

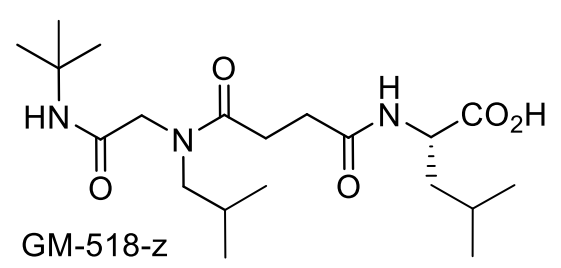

Obtained as a white solid, yield 40% (0.300 g). ^{1}H NMR (400 MHz, DMSO) δ 12.46 (s, 1H, CO_2H), [8.09 (d, *J* = 8.0 Hz), 8.06 (d, *J* = 8.0 Hz), 1H, NH], [7.62 (s), 7.20 (s), 1H, NH], 4.20 (ddd, *J* = 9.5, 8.1, 5.7 Hz, 1H), [3.91 (d, *J* = 17.3 Hz), 3.85 (d, *J* = 17.3 Hz), 3.78 (s), 2H], 3.12-2.96 (m, 2H), 2.58-2.28 (m, 4H), 1.91-1.74 (m, 1H), 1.69-1.57 (m, 1H), 1.54-1.41 (m, 2H), [1.26 (s), 1.23 (s), 9H], 0.90-0.77 (m, 12H). Conformers detected. ^{13}C NMR (101 MHz, DMSO) δ 174.22, 172.22, 171.93, 171.58, 167.68, 167.60, 55.55, 53.85, 51.01, 50.30, 50.22, 50.11, 50.08, 49.58, 30.59, 30.36, 28.52, 28.40, 27.99, 27.56, 27.25, 26.48, 24.31, 24.22, 22.87, 22.79, 21.44, 21.34, 20.05, 20.02, 19.83. Conformers detected. LC-MS (ESI, m/z) calcd for $C_{20}H_{38}N_3O_5^+$ [M+H]$^+$: 400.2806, found: 400.4.

Compound 19 (4-((2-(tert-butylamino)-2-oxoethyl)(isobutyl)amino)-4-oxobutanoyl)-L-phenylalanine

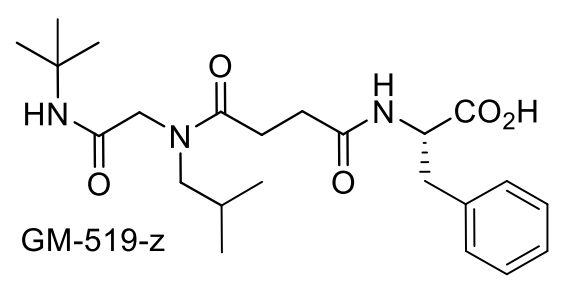

Obtained as a white solid, yield 40% (0.343 g). ^{1}H NMR (400 MHz, DMSO) δ 12.65 (s, 1H, CO_2H), [8.20 (d, *J* = 8.2 Hz), 8.17 (d, *J* = 8.1 Hz), 1H, NH], [7.61 (s, NH), 7.29-7.17 (m), 6H], 4.44-4.35 (m, 1H), [3.85 (s), 3.77 (s), 2H], [3.08-2.99 (m), 2.88-2.80 (m), 4H], 2.45-2.26 (m, 4H), 1.88-1.73 (m, 1H), [1.26 (s), 1.22 (s), 9H], [0.85 (d, *J* = 6.6 Hz), 0.78 (d, *J* = 7.1 Hz),

6H]. Conformers detected. ^{13}C NMR (101 MHz, DMSO) δ 173.04, 172.18, 171.79, 171.53, 171.51, 167.60, 167.57, 137.70, 137.67, 129.13, 129.10, 128.11, 126.36, 126.34, 55.50, 53.80, 53.47, 50.97, 50.30, 50.07, 49.50, 36.92, 36.87, 30.60, 30.48, 28.52, 28.41, 27.96, 27.64, 27.20, 26.46, 20.04, 19.82. Conformers detected. LC-MS (ESI, m/z) calcd for $C_{23}H_{36}N_3O_5^+$ [M+H]$^+$: 434.2649, found: 434.3.

Compound 20 (4-((2-(tert-butylamino)-2-oxoethyl)(isobutyl)amino)-4-oxobutanoyl)-L-proline

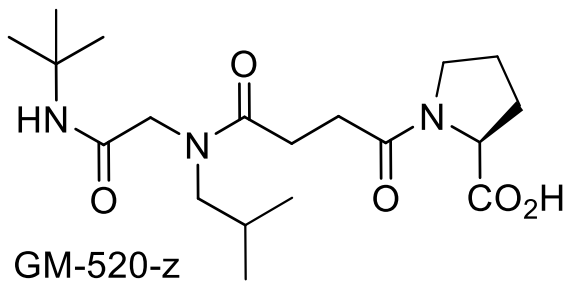

Obtained as a white solid, yield 33% (0.239 g). ^{1}H NMR (400 MHz, DMSO) δ 12.52 (s, 1H, CO_2H), [7.61 (s), 7.16 (s), 7.13 (s), 1H, NH], [4.55-4.49 (m), 4.22-4.17 (m), 1H], 3.95-3.72 (m, 2H), [3.57-3.49 (m), 3.39-3.31 (m), 2H], 3.18-3.96 (m, 2H), 2.68-2.33 (m, 4H), [2.27-2.01 (m), 1.95-1.69 (m), 5H], [1.26 (s), 1.26 (s), 1.24 (s), 1.23 (s), 9H], [0.88 (d, *J* = 6.7 Hz), 0.87 (d, *J* = 6.7 Hz), 0.79 (d, *J* = 6.7 Hz), 0.79 (d, *J* = 6.7 Hz), 6H]. Conformers detected. ^{13}C NMR (101 MHz, DMSO) δ 173.86, 173.49, 173.46, 172.26, 172.07, 171.99, 170.50, 170.41, 170.12, 170.08, 167.68, 167.64, 58.73, 58.30, 55.56, 53.90, 51.19, 51.11, 50.31, 50.13, 50.11, 49.70, 46.38, 46.00, 29.31, 29.25, 29.16, 28.87, 28.85, 28.53, 28.40, 27.69, 27.32, 27.29, 26.97, 26.50, 26.47, 24.29, 24.24, 22.25, 20.09, 20.07, 20.05, 19.89, 19.87, 19.84. Conformers detected. LC-MS (ESI, m/z) calcd for $C_{19}H_{34}N_3O_5^+$ [M+H]$^+$: 384.2493, found: 384.3.

Compound 21 (4-((2-(tert-butylamino)-2-oxoethyl)(pentyl)amino)-4-oxobutanoyl)-L-alanine

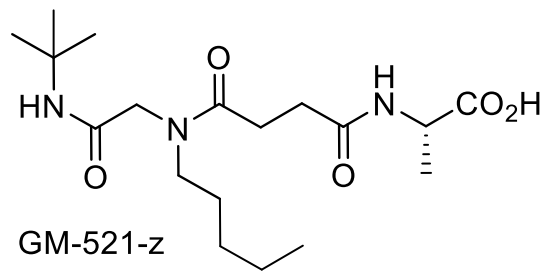

Obtained as a hygroscopic off-white solid, yield 31% (0.252 g). ^{1}H NMR (400 MHz, DMSO) δ 12.49 (s, 1H, CO_2H), [8.14 (d, *J* = 7.5 Hz), 8.11 (d, *J* = 7.3 Hz), 1H, NH], [7.63 (s), 7.21 (s), 1H, NH], [4.17 (p, *J* = 7.3 Hz), 4.16 (p, *J* = 7.3 Hz), 1H], [3.89 (d, *J* = 17.2 Hz), 3.84 (d, *J* = 17.1 Hz), 3.79 (s), 2H], [3.25 (t, *J* = 7.7 Hz), 3.17 (dt, *J* = 13.4, 7.1 Hz), 3.17 (dt, *J* = 13.4, 7.2 Hz), 2H], 2.59-2.27 (m, 4H), [1.51 (p, *J* = 7.7 Hz), 1.38 (p, *J* = 7.4 Hz), 2H], [1.26 (s), 1.23 (s), 9H], [1.32-1.13 (m), 7H], [0.87 (t, *J* = 7.1 Hz), 0.85 (t, *J* = 7.2 Hz), 3H]. Conformers detected. ^{13}C NMR (101 MHz, DMSO) δ 174.25, 171.78, 171.46, 171.34, 171.31, 167.77, 167.73, 50.62, 50.30, 50.09, 48.94, 48.35, 47.45, 46.59, 30.41, 30.23, 28.55, 28.50, 28.39, 27.86, 27.74, 27.21, 26.79, 21.97, 21.93, 17.32, 17.26, 13.89. Conformers detected. LC-MS (ESI, m/z) calcd for $C_{18}H_{34}N_3O_5^+$ [M+H]$^+$: 372.2493, found: 372.3.

Compound 22 (4-((2-(tert-butylamino)-2-oxoethyl)(pentyl)amino)-4-oxobutanoyl)-L-leucine

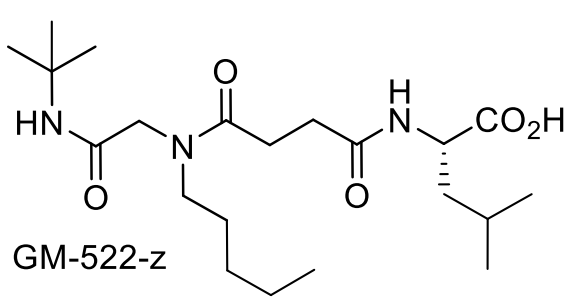

Obtained as a white solid, yield 34% (0.317 g). ^{1}H NMR (400 MHz, DMSO) δ 12.43 (s, 1H, CO_2H), [8.09 (d, *J* = 8.0 Hz), 8.06 (d, *J* = 8.0 Hz), 1H, NH], [7.62 (s), 7.20 (s), 1H, NH], 4.20 (ddd, *J* = 9.0, 7.7, 5.4 Hz, 1H), [3.89 (d, *J* = 17.2 Hz), 3.84 (d, *J* = 17.2 Hz), 3.79 (s), 2H], 3.28-3.12 (m, 2H), 2.58-2.29 (m, 4H), 1.69-1.57 (m, 1H), 1.56-1.44 (m, 2H), [1.26 (s), 1.23 (s), 9H], 1.42-1.13 (m, 6H), 0.91-0.81 (m, 9H). Conformers detected. ^{13}C NMR (101 MHz, DMSO) δ 174.22, 171.75, 171.59, 171.45, 167.78, 167.72, 50.63, 50.29, 50.20, 50.13, 50.09, 48.96, 48.37, 46.64, 30.50, 30.30, 28.54, 28.50, 28.38, 27.94, 27.74, 27.32, 26.78, 24.31, 24.23, 22.86, 22.80, 21.97, 21.92, 21.41, 21.35, 13.88. Conformers detected. LC-MS (ESI, m/z) calcd for $C_{21}H_{40}N_3O_5^+$ [M+H]$^+$: 414.2962, found: 414.4.

Compound 23 (4-((2-(tert-butylamino)-2-oxoethyl)(pentyl)amino)-4-oxobutanoyl)-L-phenylalanine

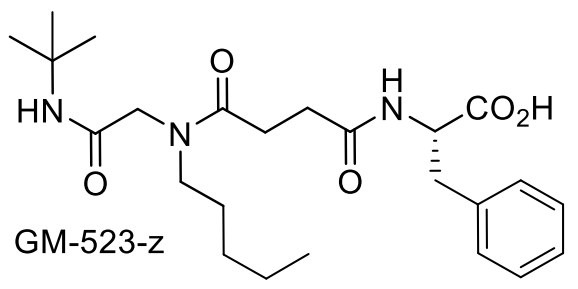

Obtained as a white solid, yield 37% (373 g). ^{1}H NMR (400 MHz, DMSO) δ 12.67 (s, 1H, CO_2H), [8.20 (d, *J* = 8.2 Hz), 8.17 (d, *J* = 8.0 Hz), 1H, NH], [7.61 (s, NH), 7.30-7.17 (m), 6H], [4.41 (td, *J* = 7.8, 4.4 Hz), 4.38 (td, *J* = 7.9, 4.6 Hz), 1H], [3.84 (s), 3.78 (s), 2H], 3.24-3.15 (m, 2H), [3.06-3.00 (m), 2.84 (dd, *J* = 13.7, 9.2 Hz), 2H], (2.45-2.26 (m), 4H), [1.37 (p, *J* = 7.7 Hz), 1.48 (p, *J* = 7.9 Hz), 2H], [1.25 (s), 1.23 (s), 9H], 1.31-1.12 (m, 4H), [0.87 (t, *J* = 7.1 Hz), 0.84 (t, *J* = 7.2 Hz), 3H]. Conformers detected. ^{13}C NMR (101 MHz, DMSO) δ 173.04, 171.70, 171.54, 171.51, 171.32, 167.70, 137.70, 137.67, 129.13, 129.11, 128.11, 126.35, 53.46, 50.57, 50.29, 50.08, 48.89, 48.31, 46.57, 36.92, 36.87, 30.51, 30.40, 28.54, 28.51, 28.40, 28.38, 27.92, 27.72, 27.36, 26.77, 21.96, 21.93, 13.88. Conformers detected. LC-MS (ESI, m/z) calcd for $C_{24}H_{38}N_3O_5^+$ $[M+H]^+$: 448.2806, found: 448.4.

Compound 24 (4-((2-(tert-butylamino)-2-oxoethyl)(pentyl)amino)-4-oxobutanoyl)-L-proline

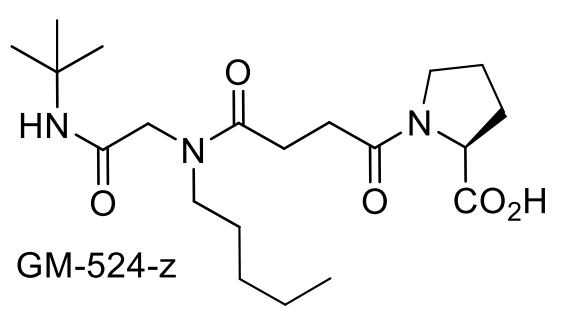

Obtained as a hygroscopic off-white solid, yield 26% (0.236 g). ^{1}H NMR (400 MHz, DMSO) δ 12.53 (s, 1H, CO_2H), [7.62 (s), 7.17 (s), 7.14 (s), 1H, NH], [4.54-4.59 (m), 4.20 (dd, *J* = 7.9, 4.3 Hz), 4.19 (dd, *J* = 7.7, 4.5 Hz), 1H], 3.93-3.73 (m, 2H), [3.57-3.50 (m), 3.39-3.12 (m), 4H], 2.68-2.32 (m , 4H), [2.27-2.01 (m), 1.94-1.69 (m), 4H], [1.52 (p, *J* = 7.7 Hz), 1.38 (h, *J* = 6.4 Hz), 2H], [1.26 (s), 1.26 (s), 1.24 (s), 1.23 (s), 9H], 1.33-1.13 (m, 4H), [0.87 (t, *J* = 7.0 Hz), 0.85 (t, *J* = 7.2 Hz), 3H]. Conformers detected. ^{13}C NMR (101 MHz, DMSO) δ 173.85, 173.49, 173.45, 171.81, 171.58, 171.51, 170.49, 170.42, 170.11, 170.08, 167.76, 167.74, 58.76, 58.30, 50.79, 50.30, 50.12, 49.07, 48.39, 46.65, 46.37, 46.03, 45.99, 30.89, 29.24, 29.15, 28.86, 28.56, 28.51, 28.39, 27.77, 27.27, 27.08, 26.79, 26.69, 24.28, 24.24, 22.25, 21.97, 21.94, 13.89. Conformers detected. LC-MS (ESI, m/z) calcd for $C_{20}H_{36}N_3O_5^+$ $[M+H]^+$: 398.2649, found: 398.4.

Compound 25 (4-(benzyl(2-(tert-butylamino)-2-oxoethyl)amino)-4-oxobutanoyl)-L-alanine

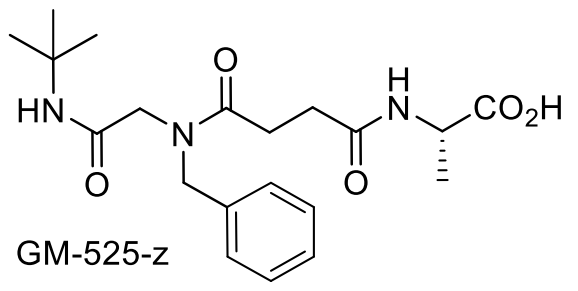

Obtained as a white solid, yield 29% (0.352 g). ^{1}H NMR (400 MHz, DMSO) δ 12.44 (s, 1H, CO_2H), [8.16 (d, *J* = 7.2 Hz), 8.15 (d, *J* = 7.5 Hz), 1H, NH], [7.54 (s, NH), 7.39-7.18 (m), 6H], [4.58 (s), 4.49 (d, *J* = 15.2 Hz), 4.41 (d, *J* = 15.2 Hz), 2H], [4.19 (p, *J* = 7.3 Hz), 4.18 (p, *J* = 7.3 Hz), 1H], [3.87 (d, *J* = 17.5 Hz), 3.84 (d, *J* = 17.2 Hz), 3.77 (s), 2H], 2.65-2.34 (m, 4H), [1.25 (d, *J* = 7.3 Hz), 1.24 (d, *J* = 7.3 Hz), 3H], 1.22 (s, 9H). Conformers detected. ^{13}C NMR (101 MHz, DMSO) δ 174.26, 172.45, 172.03, 171.28, 171.23, 167.23, 167.21, 137.78, 137.27, 128.65, 128.31, 127.62, 127.27, 126.92, 126.87, 51.39, 50.31, 50.13, 49.38, 48.58, 47.48, 47.45, 30.43, 30.15, 28.51, 28.39, 27.69, 27.54, 17.32, 17.28. Conformers detected. LC-MS (ESI, m/z) calcd for $C_{20}H_{30}N_3O_5^+$ $[M+H]^+$: 392.2180, found: 392.3.

Compound 26 (4-(benzyl(2-(tert-butylamino)-2-oxoethyl)amino)-4-oxobutanoyl)-L-leucine

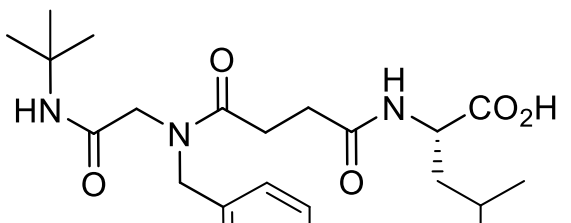

GM-526-z

Obtained as a white solid, yield 37% (0.496 g). ^{1}H NMR (400 MHz, DMSO) δ 12.46 (s, 1H, CO_2H), [8.11 (d, *J* = 7.9 Hz), 8.09 (d, *J* = 8.0 Hz), 1H, NH], [7.53 (s, NH), 7.39-7.18 (m), 6H], [4.58 (s), 4.49 (d, *J* = 15.2 Hz), 4.40 (d, *J* = 15.2 Hz), 2H], [4.23 (ddd, *J* = 9.5, 8.0, 5.6 Hz), 4.21 (ddd, *J* = 10.4, 8.0, 5.8 Hz), 1H], [3.84 (s), 3.77(s), 2H], 2.62-2.35 (m, 4H), 1.70-1.58 (m, 1H), 1.56-1.43 (m, 2H), 1.22 (s, 9H), [0.89 (d, *J* = 6.6 Hz), 0.88 (d, *J* = 6.6 Hz), 0.84 (d, *J* = 6.6 Hz), 0.83 (d, *J* = 6.4 Hz), 6H]. Conformers detected. ^{13}C NMR (101 MHz, DMSO) δ 174.22, 172.43, 172.03, 171.53, 171.50, 167.22, 137.79, 137.28, 128.64, 128.30, 127.65, 127.26, 126.92, 126.88, 51.41, 50.31, 50.21, 50.15, 50.12, 49.41, 48.62, 30.48, 30.20, 28.51, 28.44, 28.37, 27.78, 27.63, 24.29, 24.26, 22.87, 22.81, 21.43, 21.37. Conformers detected. LC-MS (ESI, m/z) calcd for $C_{23}H_{36}N_3O_5^+$ $[M+H]^+$: 434.2649, found: 434.4.

Compound 27 (4-(benzyl(2-(tert-butylamino)-2-oxoethyl)amino)-4-oxobutanoyl)-L-phenylalanine

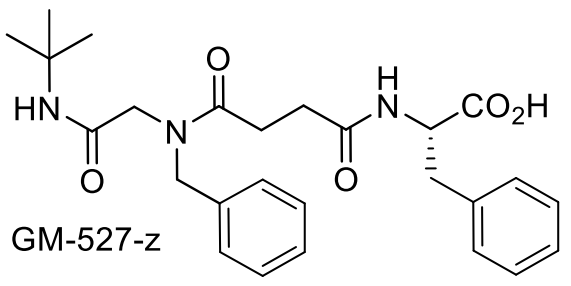

GM-527-z

Obtained as a white solid, yield 36% (0.525 g). ^{1}H NMR (400 MHz, DMSO) δ 12.66 (m, 1H, CO_2H), [8.22 (d, *J* = 8.1 Hz), 8.20 (d, *J* = 8.1 Hz), 1H, NH], [7.52 (s, NH), 7.38-7.15 (m), 11H], [4.54(s), 4.44 (s), 2H], 4.45-4.38 (m, 1H), [3.81 (s), 3.76 (s), 2H], [3.07-3.00 (m), 2.89-2.81 (m), 2H], 2.49-2.30 (m, 4H), 1.21 (s, 9H). Conformers detected. ^{13}C NMR (101 MHz, DMSO) δ 173.04, 172.37, 171.92, 171.48, 171.42, 167.20, 167.14, 137.76, 137.69, 137.63, 137.22, 129.12, 128.65, 128.31, 128.13, 128.11, 127.64, 127.27, 126.93, 126.83, 126.36, 53.49, 53.44, 51.36, 50.31, 50.12, 50.06, 49.34, 48.54, 36.93, 36.89, 30.52, 30.32, 28.51, 28.44, 28.38, 27.75, 27.67. Conformers detected. LC-MS (ESI, m/z) calcd for $C_{26}H_{34}N_3O_5^+$ $[M+H]^+$: 468.2493, found: 468.3.

Compound 28 (4-(benzyl(2-(tert-butylamino)-2-oxoethyl)amino)-4-oxobutanoyl)-L-proline

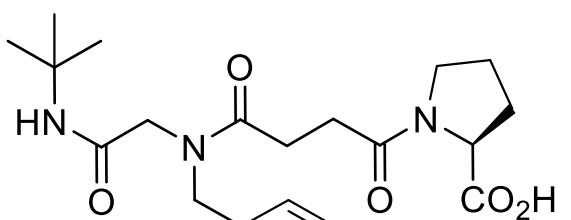

GM-528-z

Obtained as a white solid, yield 31% (0.411 g). ^{1}H NMR (400 MHz, DMSO) δ 12.50 (s, 1H, CO_2H), [7.53 (s, NH), 7.39-7.18 (m), 6H], 4.66-4.9 (m, 3H), 3.92-3.71 (m, 2H), [3.59-3.50 (m), 3.41-3.33 (m), 2H], 2.70-2.42 (m, 2H), [2.30-2.02 (m), 1.95-1.71 (m), 4H], [1.22 (s), 1.21 (s), 9H]. Conformers detected. ^{13}C NMR (101 MHz, DMSO) δ 173.84, 173.49, 173.46, 172.50, 172.06, 170.37, 170.03, 167.25, 167.21, 167.18, 137.76, 137.29, 128.63, 128.31, 127.67, 127.26, 126.92, 58.74, 58.32, 58.29, 51.41, 50.31, 50.13, 49.41, 48.66, 46.38, 46.03, 30.88, 29.33, 29.14, 29.07, 28.87, 28.51, 28.37, 27.50, 27.12, 27.06, 24.29, 24.23, 22.25. Conformers detected. LC-MS (ESI, m/z) calcd for $C_{22}H_{32}N_3O_5^+$ $[M+H]^+$: 418.2336, found: 418.3.

Compound 29 (4-((2-(tert-butylamino)-2-oxoethyl)(furan-2-ylmethyl)amino)-4-oxobutanoyl)-L-alanine

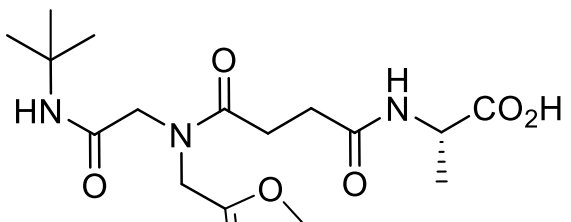

GM-529-z

Obtained as a light-yellow solid, yield 30% (0.312 g). ^{1}H NMR (400 MHz, DMSO) δ 12.43 (s, 1H, CO_2H), [8.16 (d, *J* = 7.3 Hz), 8.14 (d, *J* = 7.3 Hz), 1H, NH], [7.63 (d, *J* = 1.8 Hz), 7.56 (d, *J* = 1.8 Hz), 1H],

[7.52 (s), 7.22 (s), 1H, NH], [6.43 (dd, *J* = 3.2, 1.8 Hz), 6.40-6.37 (m), 6.28 (d, *J* = 3.2 Hz), 2H], [4.53 (s), 4.48 (d, *J* = 15.5 Hz), 4.40 (d, *J* = 15.5 Hz), 2H], 4.24-4.12 (m, 1H), [3.89 (d, *J* = 17.4 Hz), 3.84 (d, *J* = 17.5 Hz), 3.77 (s), 2H], [2.78-2.62 (m), 2.48-2.30 (m), 4H], [1.24 (s), 1.23 (s), 9H], 1.27-1.21 (m, 3H). Conformers detected. ^{13}C NMR (101 MHz, DMSO) δ 174.27, 172.08, 171.71, 171.21, 167.17, 167.08, 150.87, 150.56, 142.99, 142.51, 110.53, 110.43, 108.40, 108.36, 50.33, 50.12, 49.75, 48.20, 47.47, 44.74, 42.35, 30.23, 30.18, 28.51, 28.41, 27.73, 27.61, 17.31, 17.27. Conformers detected. LC-MS (ESI, m/z) calcd for $C_{18}H_{28}N_3O_6^+$ $[M+H]^+$: 382.1973, found: 382.3.

Compound 30 (4-((2-(tert-butylamino)-2-oxoethyl)(furan-2-ylmethyl)amino)-4-oxobutanoyl)-L-leucine

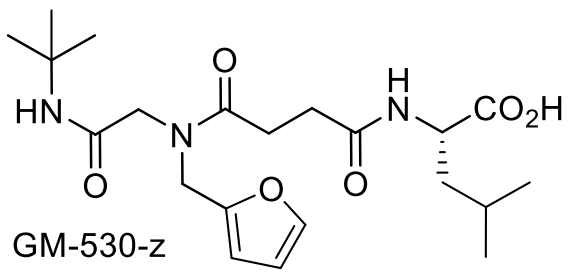

Obtained as a white solid, yield 36% (0.408 g). ^{1}H NMR (400 MHz, DMSO) δ 12.47 (s, 1H, CO_2H), [8.10 (d, *J* = 5.8 Hz), 8.08 (d, *J* = 5.8 Hz), 1H, NH], [7.63 (dd, *J* = 1.8, 0.8 Hz), 7.56 (dd, *J* = 1.8, 0.9 Hz), 1H], [7.52 (s), 7.21 (s), 1H, NH], [6.43 (dd, *J* = 3.2, 1.8 Hz), 6.40- 6.37 (m), 6.28 (d, *J* = 3.1 Hz), 2H], [4.52 (s), 4.47 (d, *J* = 15.5 Hz), 4.41 (d, *J* = 15.5 Hz), 1H], 4.24-4.17 (m, 1H), [3.87 (s), 3.77 (s), 1H], [2.77-2.63 (m), 2.49-2.32 (m), 4H], 1.70-1.58 (m, 1H), 1.55-1.43 (m, 2H), [1.24 (s), 1.22 (s), 9H], [0.89 (d, *J* = 6.5 Hz), 0.88 (d, *J* = 6.6 Hz), 0.84 (d, *J* = 6.6 Hz), 0.83 (d, *J* = 6.6 Hz), 6H]. Conformers detected. ^{13}C NMR (101 MHz, DMSO) δ 174.23, 172.05, 171.69, 171.46, 171.44, 167.15, 167.08, 150.88, 150.56, 142.98, 142.50, 110.52, 110.43, 108.40, 108.33, 50.32, 50.23, 50.17, 50.11, 49.76, 48.22, 44.74, 42.38, 30.30, 30.21, 28.51, 28.39, 27.83, 27.68, 24.30, 24.26, 22.86, 22.81, 21.44, 21.37. Conformers detected. LC-MS (ESI, m/z) calcd for $C_{21}H_{34}N_3O_6^+$ $[M+H]^+$: 424.2442, found: 424.3.

Compound 31 (4-((2-(tert-butylamino)-2-oxoethyl)(furan-2-ylmethyl)amino)-4-oxobutanoyl)-L-phenylalanine

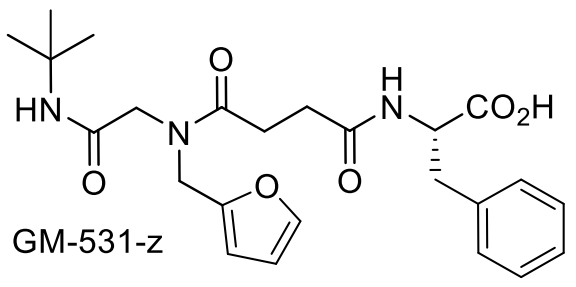

Obtained as a white solid, yield 37% (0.446 g). ^{1}H NMR (400 MHz, DMSO) δ 12.67 (s, CO_2H), [8.21 (d, *J* = 7.7 Hz), 8.19 (d, *J* = 7.8 Hz), 1H, NH], [7.63 (dd, *J* = 1.9, 0.8 Hz), 7.56 (dd, *J* = 1.9, 0.9 Hz), 1H], [7.52 (s, NH), 7.29-7.16 (m), 6H], [6.42 (dd, *J* = 3.3, 1.8 Hz), 6.39-6.36 (m), 6.27 (dd, *J* = 3.2, 0.8 Hz), 2H], [4.48 (s), 4.45 (d, *J* = 15.4 Hz), 4.41 (d, *J* = 15.4 Hz), 2H], 4.45-4.37 (m, 1H), [3.84 (s), 3.76 (s), 2H], [3.03 (dd, *J* = 13.7, 5.2 Hz), 3.03 (dd, *J* = 13.7, 5.1 Hz), 2.85 (dd, *J* = 13.8, 9.1 Hz), 2.84 (dd, *J* = 13.8, 9.2 Hz), 2H], [2.62 (t, *J* = 7.2 Hz), 2.41-2.29 (m), 4H], [1.23 (s), 1.22 (s), 9H]. Conformers detected. ^{13}C NMR (101 MHz, DMSO) δ 173.04, 172.00, 171.59, 171.38, 167.14, 167.00, 150.83, 150.50, 142.99, 142.51, 137.68, 137.64, 129.14, 129.12, 129.04, 128.12, 126.36, 110.52, 110.43, 108.38, 53.47, 50.32, 50.12, 49.69, 48.16, 44.72, 42.30, 36.90, 30.33, 28.51, 28.41, 27.80, 27.73. Conformers detected. LC-MS (ESI, m/z) calcd for $C_{24}H_{32}N_3O_6^+$ $[M+H]^+$: 458.2286, found: 458.3.

Compound 32 (4-((2-(tert-butylamino)-2-oxoethyl)(furan-2-ylmethyl)amino)-4-oxobutanoyl)-L-proline

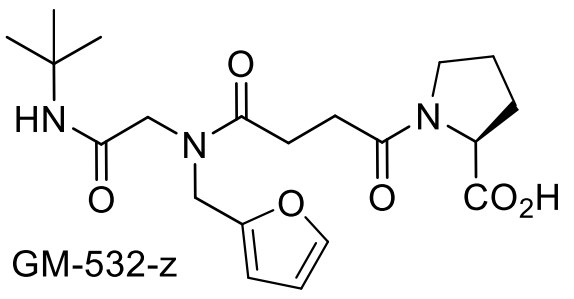

Obtained as a light-yellow solid, yield 30% (0.331 g). ^{1}H NMR (400 MHz, DMSO) δ 12.47 (s, 1H, CO_2H), [7.63 (dd, *J* = 1.9, 0.9 Hz), 7.57 (dd, *J* = 1.9, 0.8 Hz), 1H], [7.51 (s), 7.18 (s), 7.17 (s), 1H, NH], [6.43 (dd, *J* = 3.3, 1.8 Hz), 6.38 (dd, *J* = 3.2, 1.9 Hz), 1H], [6.41 (d, *J* = 3.3 Hz), 6.28 (d, *J* = 3.2 Hz), 1H], [4.57-4.36 (m), 4.23-4.18 (m), 3H], [3.91-3.86(m), 3.81 (d, *J* = 16.3 Hz), 3.75 (d, *J* = 16.3 Hz), 2H], [3.58-3.49(m), 3.40-3.32(m), 2H], [2.84-2.73 (m), 2.68-2.36(m), 4H], [2.28-2.03 (m), 1.95-1.70 (m), 4H], [1.24 (s), 1.24 (s), 1.23 (s), 1.22 (s), 9H]. Conformers detected. ^{13}C NMR (101 MHz, DMSO) δ 173.85, 173.49, 172.13, 172.01, 171.83, 171.78, 170.35, 170.29, 169.99, 169.95, 167.18, 167.07, 150.84, 150.58, 150.56, 142.97, 142.53, 110.54, 110.44, 108.43, 108.41, 58.73, 58.32, 50.33, 50.14, 49.91, 48.31, 46.39, 46.03, 44.76, 42.37, 30.88, 29.14, 29.06, 28.87, 28.51, 28.39, 27.60, 27.50, 27.22, 27.10, 24.29, 24.27, 22.26, 21.06. Conformers detected. LC-MS (ESI, m/z) calcd for $C_{20}H_{30}N_3O_6^+$ $[M+H]^+$: 408.2129, found: 408.3.

Compound 33 (4-((2-((2-aminoethyl)amino)-2-oxoethyl)(isobutyl)amino)-4-oxobutanoyl)-L-alanine

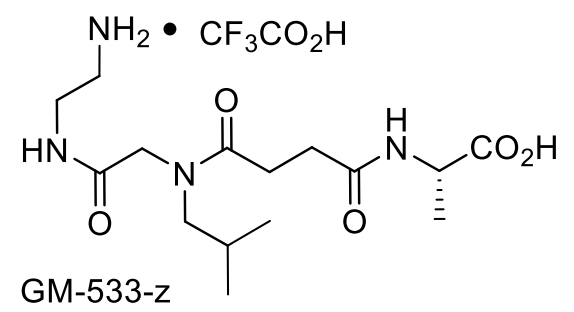

Obtained as an off-white solid, yield 33% (0.278 g). ^{1}H NMR (400 MHz, DMSO) δ [8.57 (t, *J* = 5.6 Hz), 7.99 (t, *J* = 5.7 Hz), 1H, NH], [7.85 (d, *J* = 7.0 Hz), 7.64 (d, *J* = 7.3 Hz), 1H, NH], 6.40-4.31 (s, 3H, NH_3), 4.04-3.68 (m, 3H), 3.35-2.99 (m, 4H), 2.86-2.75 (m, 2H), 2.68-2.31 (m, 4H), 1.91-1.76 (m, 1H), 1.17 (d, *J* = 7.0 Hz, 3H), [0.88 (d, *J* = 6.7 Hz), 0.86 (d, *J* = 6.6 Hz), 0.79 (d, *J* = 6.6 Hz), 0.79 (d, *J* = 6.7 Hz), 6H]. Conformers detected. ^{13}C NMR (101 MHz, DMSO) δ 174.72, 174.70, 172.73, 172.66, 171.97, 171.85, 169.81, 169.75, 158.64 (q, *J* = 30.9 Hz), 117.71 (q, *J* = 300 Hz), 56.05, 54.06, 51.08, 49.95, 47.96, 47.89, 37.00, 36.77, 30.85, 30.78, 28.27, 27.90, 27.83, 26.86, 20.46, 20.44, 20.24, 17.73, 17.69. Conformers detected. ^{19}F NMR (376 MHz, DMSO) δ -73.56. LC-MS (ESI, m/z) calcd for $C_{15}H_{29}N_4O_5^+$ $[M+H]^+$: 345.2132, found: 345.4.

Compound 34 (4-((2-((2-aminoethyl)amino)-2-oxoethyl)(isobutyl)amino)-4-oxobutanoyl)-L-leucine

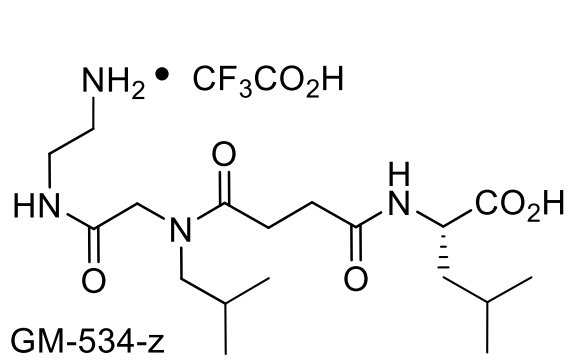

Obtained as a very hygroscopic light-yellow oil, yield 22% (0.194 g). ^{1}H NMR (400 MHz, DMSO) δ [8.42 (t, *J* = 5.7 Hz), 7.99 (t, *J* = 5.7 Hz), 1H, NH], [8.04 (d, *J* = 7.9 Hz), 7.97 (d, *J* = 8.0 Hz), 1H, NH], 9.5-6.5 (s, 3H, NH3), 4.19-4.04 (m, 1H), 4.01-3.71 (m, 2H), 3.38-3.20 (m, 2H), [3.17-3.08 (m), 3.05-3.96 (m), 2.90-2.79 (m), 4H], 2.60-2.33 (m, 4H), 1.92-1.75 (m, 1H), 1.69-1.56 (m, 1H), 1.47 (t, *J* = 7.3 Hz, 2H), 0.93-0.76 (m, 12H). Conformers detected. ^{13}C NMR (101 MHz, DMSO) δ 174.21, 173.31, 172.21, 171.76, 171.62, 169.37, 169.31, 161.29, 158.32, 158.00, 79.19, 55.57, 53.60, 50.63, 50.27, 50.17, 49.44, 40.55, 38.68, 38.58, 38.25, 36.52, 36.32, 30.38, 27.82, 27.59, 27.36, 26.41, 24.41, 24.32, 24.26, 22.85, 22.83, 22.61, 21.61, 21.41, 21.39, 20.01, 19.97, 19.78, 19.24. Conformers detected. ^{19}F NMR (376 MHz, DMSO) δ -73.57. LC-MS (ESI, m/z) calcd for $C_{18}H_{35}N_4O_5^+$ $[M+H]^+$: 387.2602, found: 387.4.

Compound 35 (4-((2-((2-aminoethyl)amino)-2-oxoethyl)(isobutyl)amino)-4-oxobutanoyl)-L-phenylalanine

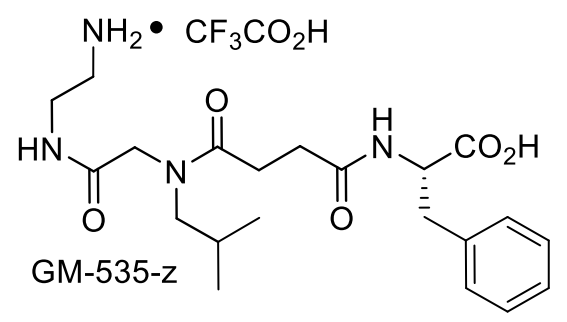

Obtained as an off-white solid, yield 29% (0.276 g). ^{1}H NMR (400 MHz, DMSO) δ [8.46 (t, *J* = 5.6 Hz), 7.92 (t, *J* = 5.9 Hz), 1H, NH] [7.82 (d, *J* = 8.0 Hz), 7.97 (d, *J* = 8.0 Hz), 1H, NH], 9.5-6.5 (s, 3H, NH3), 7.29-7.11 (m,5H), [4.26 (td, *J* = 8.9, 4.9 Hz), 4.19 (td, *J* = 8.2, 4.9 Hz), 1H], [3.97 (s), 3.95 (d, *J* = 11.9 Hz), 3.69 (d, *J* = 16.2 Hz), 2H], [3.40-3.17 (m), 3.14-2.98 (m), 2.89-2.76 (m), 8H], [2.61-2.45 (m), 2.39-2.23 (m), 4H], 1.90-1.75 (m, 1H), [0.86 (d, *J* = 6.6 Hz), 0.84 (d, *J* = 6.6 Hz), 0.79 (d, *J* = 6.6 Hz), 0.78 (d, *J* = 6.6 Hz), 6H]. Conformers detected. ^{13}C NMR (101 MHz, DMSO) δ 172.98, 172.26, 171.65, 171.29, 169.09, 158.01 (q, *J* = 30.8 Hz), 139.07, 138.75, 129.22, 129.17, 127.93, 125.97, 125.93, 117.31 (q, *J* = 300.7 Hz), 55.87, 55.68, 54.89, 53.65, 50.70, 50.20, 38.51, 38.31, 37.36, 36.85, 36.53, 30.93, 30.83, 27.54, 27.35, 26.39, 20.02, 20.00, 19.76, 19.70. Conformers detected. ^{19}F NMR (376 MHz, DMSO) δ -73.54. LC-MS (ESI, m/z) calcd for $C_{21}H_{33}N_4O_5^+$ $[M+H]^+$: 421.2445, found: 421.4.

Compound 36 (4-((2-((2-aminoethyl)amino)-2-oxoethyl)(isobutyl)amino)-4-oxobutanoyl)-L-proline

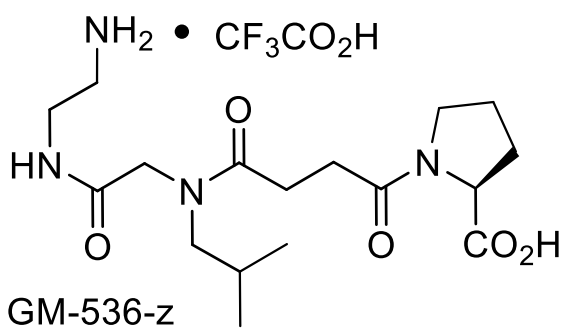

Obtained as an off-white solid, yield 37% (0.318 g). ^{1}H NMR (400 MHz, DMSO) δ 12.63 (s, 1H, CO_2H), [8.23 (t, *J* = 5.7 Hz), 7.88 (t, *J* = 5.9 Hz), 7.84 (t, *J* = 5.7 Hz), 1H, NH], 8.02-7.63 (s, 3H, NH_3), [4.53 (dd, *J* = 8.7, 2.6 Hz), 4.21 (t, *J* = 4.6 Hz), 4.19 (t, *J* = 4.3 Hz), 1H], [4.05 (d, *J* = 17.8 Hz), 4.03 (d, *J* = 17.7 Hz), 3.97 (d, *J* = 17.7 Hz), 3.97 (d, *J* = 17.7 Hz), 3.88 (d, *J* = 16.2 Hz), 3.87 (d, *J* = 16.3 Hz), 3.82 (d, *J* = 16.2 Hz), 3.81 (d, *J* = 16.2 Hz,), 2H], [3.57-3.48 (m), 3.40-3.25 (m), 3.23-3.17(m), 3.15-2.99 (m), 6H], 2.92-2.82 (m, 2H), 2.67-2.33 (m, 4H), [2.27-2.02 (m), 1.95-1.79 (m), 5H], [3.81 (d, *J* = 16.2 Hz), 0.80 (d, *J* = 6.6 Hz), 0.80 (d, *J* = 6.7 Hz), 0.79 (d, *J* = 6.8 Hz), 6H]. Conformers detected. ^{13}C NMR (101 MHz, DMSO) δ 173.71, 173.51, 173.50, 172.41, 172.38, 172.22, 172.20, 170.68, 170.52, 170.32, 170.13, 169.42, 169.33, 169.29, 117.27 (q, *J* = 300.4 Hz), 117.27 (q, *J* = 300.4 Hz), 58.76, 58.39, 58.33, 55.69, 55.64, 53.68, 50.73, 49.71, 46.45, 46.42, 46.04, 38.75, 38.59, 36.54, 36.28, 30.87, 29.36, 29.25, 28.86, 27.49, 27.27, 26.85, 26.42, 24.28, 22.25, 22.22, 20.04, 20.01, 19.83, 19.80. Conformers detected. ^{19}F NMR (376 MHz, DMSO) δ -73.55. LC-MS (ESI, m/z) calcd for $C_{17}H_{31}N_4O_5^+$ $[M+H]^+$: 371.2289, found: 371.3.

Compound 37 (4-((2-((2-aminoethyl)amino)-2-oxoethyl)(pentyl)amino)-4-oxobutanoyl)-L-alanine

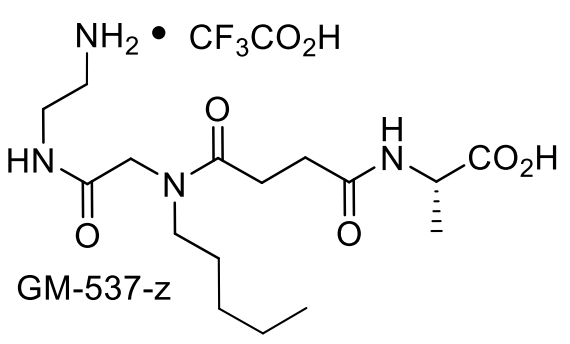

Obtained as an off-white solid, yield 44% (0.392 g). ^{1}H NMR (400 MHz, DMSO) δ 12.49 (s, 1H, CO_2H), [8.23 (t, *J* = 5.7 Hz), 7.92 (t, *J* = 5.8 Hz), 1H, NH], [8.20 (d, *J* = 7.2 Hz), 8.17 (d, *J* = 7.3 Hz), 1H, NH], 7.91-7.65 (s, 3H, NH_3), 4.23-4.14 (m, 1H), [4.00 (d, *J* = 17.7 Hz), 3.94 (d, *J* = 17.7 Hz), 3.87 (d, *J* = 16.2 Hz), 3.82 (d, *J* = 16.2 Hz), 2H], 3.37-3.18 (m, 4H), 2.92-2.81 (m, 2H), 2.62-2.30 (m, 4H), [1.52 (p, *J* = 7.6 Hz), 1.39 (p, *J* = 7.5 Hz), 2H], [1.25 (d, *J* = 2.7 Hz), 1.24 (d, *J* = 2.7 Hz), 3H], 1.33-1.13 (m, 4H), [0.88 (t, *J* = 7.1 Hz), 0.85 (t, *J* = 7.2 Hz),

3H]. Conformers detected. ^{13}C NMR (101 MHz, DMSO) δ 174.24, 171.80, 171.76, 171.47, 171.40, 169.46, 169.36, 158.18 (q, *J* = 30.9 Hz), 117.24 (q, *J* = 300.0 Hz), 50.17, 48.91, 48.47, 47.46, 47.42, 46.45, 38.65, 38.57, 36.53, 36.32, 30.29, 30.20, 28.51, 28.37, 27.76, 27.25, 26.64, 21.96, 21.94, 17.26, 17.24, 13.92. Conformers detected. ^{19}F NMR (376 MHz, DMSO) δ -73.57. LC-MS (ESI, m/z) calcd for $C_{16}H_{31}N_4O_5^+$ [M+H]$^+$: 359.2289, found: 359.3.

Compound 38 (4-((2-((2-aminoethyl)amino)-2-oxoethyl)(pentyl)amino)-4-oxobutanoyl)-L-leucine

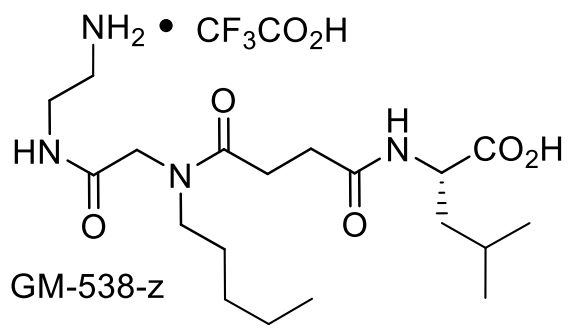

Obtained as an off-white solid, yield 45% (0.438 g). ^{1}H NMR (400 MHz, DMSO) δ [8.22 (t, *J* = 5.7 Hz), 7.92 (t, *J* = 5.8 Hz), 1H, NH], [8.14 (d, *J* = 7.9 Hz), 8.10 (d, *J* = 8.0 Hz), 1H, NH], 7.87-7.74 (s, 3H, NH_3), 4.20 (td, *J* = 8.6, 6.0 Hz, 1H), [4.00 (d, *J* = 17.9 Hz), 3.94 (d, *J* = 17.7 Hz), 3.87 (d, *J* = 16.3 Hz), 3.82 (d, *J* = 16.3 Hz), 2H], 3.36-3.13 (m, 4H), 2.92-2.81 (m, 2H), 2.59-2.30 (m, 4H), 1.71-1.58 (m, 1H), 1.56-1.13 (m, 8H), 0.91-0.81 (m, 9H). Conformers detected. ^{13}C NMR (101 MHz, DMSO) δ 174.21, 171.75, 171.72, 171.63, 169.47, 169.38, 158.24 (q, *J* = 32.8 Hz), 116.70 (q, *J* = 296.7 Hz), 50.24, 50.18, 48.86, 48.45, 46.48, 38.67, 38.58, 36.53, 36.33, 30.33, 30.29, 28.51, 28.51, 28.37, 27.78, 27.74, 27.41, 26.63, 24.32, 24.27, 22.84, 21.97, 21.93, 21.39, 19.24, 13.91, 9.66. Conformers detected. ^{19}F NMR (376 MHz, DMSO) δ -73.57. LC-MS (ESI, m/z) calcd for $C_{19}H_{37}N_4O_5^+$ [M+H]$^+$: 401.2758, found: 401.4.

Compound 39 (4-((2-((2-aminoethyl)amino)-2-oxoethyl)(pentyl)amino)-4-oxobutanoyl)-L-phenylalanine

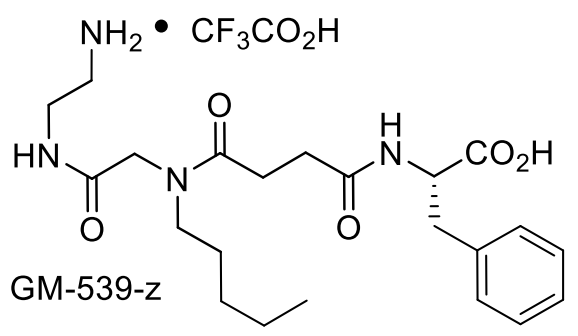

Obtained as a white solid, yield 32% (0.345 g). ^{1}H NMR (400 MHz, DMSO) δ9.5-6.5 (s, 3H, NH3), [8.65 (t, *J* = 5.6 Hz), 7.97 (t, *J* = 5.8 Hz), 1H, NH], [8.65 (t, *J* = 5.6 Hz), 7.97 (t, *J* = 5.8 Hz), 1H, NH], 7.29-7.13 (m, 5H), 4.22-4.12 (m, 1H), [3.97 (d, *J* = 17.8 Hz), 3.95 (d, *J* = 16.3 Hz), 3.91 (d, *J* = 17.8 Hz), 3.68 (d, *J* = 16.3 Hz), 2H], 4.03-3.11 (m, 4H), [3.08-3.01 (m), 2.89-2.75 (m), 4H], [2.61-2.47 (m), 2.37-2.21(m), 4H], 1.56-1.33 (m, 2H), 1.33-1.12 (m, 4H), [0.86 (d, *J* = 7.1 Hz), 0.84 (t, *J* = 7.2 Hz), 3H]. Conformers detected. ^{13}C NMR (101 MHz, DMSO) δ 174.68, 174.54, 172.65, 171.84, 171.54, 171.24, 169.13, 169.08, 139.27, 139.01, 129.21, 129.16, 128.19, 127.87, 125.83, 55.92, 55.30, 50.35, 49.80, 48.84, 46.57, 38.48, 38.21, 37.48, 37.02, 36.66, 31.01, 30.79, 28.53, 28.44, 28.31, 27.92, 27.14, 26.60, 21.95, 21.92, 13.92. Conformers detected. ^{19}F NMR (376 MHz, DMSO) δ -73.51. LC-MS (ESI, m/z) calcd for $C_{22}H_{35}N_4O_5^+$ [M+H]$^+$: 435.2602, found: 435.4.

Compound 40 (4-((2-((2-aminoethyl)amino)-2-oxoethyl)(pentyl)amino)-4-oxobutanoyl)-L-proline

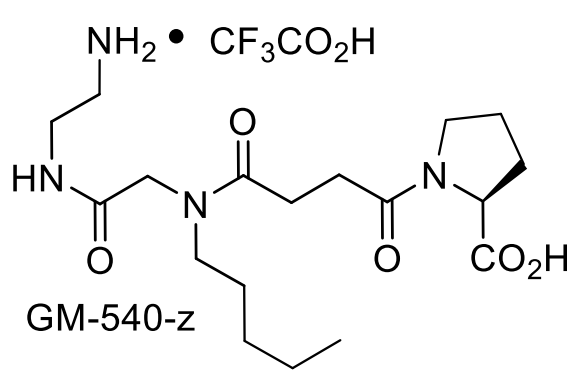

Obtained as an off-white solid, yield 48% (0.458 g). ^{1}H NMR (400 MHz, DMSO) δ9.5-6.5 (s, 3H, NH3),[8.73 (t, *J* = 5.6 Hz), 8.60 (t, *J* = 5.5 Hz), 8.09 (t, *J* = 5.7 Hz), 7.77 (t, *J* = 5.8 Hz), 1H, NH],4.22-3.75 (m, 3H), 3.63-3.12 (m, 6H), 2.94-2.17 (m, 6H), 2.12-1.68 (m, 4H), 1.64-1.12 (m, 6H), 0.90-0.82 (m, 3H). Conformers detected. ^{13}C NMR (101 MHz, DMSO) δ 173.73, 173.50, 171.93, 171.80, 170.63, 170.54, 170.26, 170.15, 169.52, 169.39, 158.19 (q, *J* = 31.5 Hz), 117.22 (q, *J* = 299.7 Hz), 58.74, 58.37, 58.34, 50.28, 49.08,

48.99, 48.55, 46.54, 46.43, 46.04, 38.71, 38.58, 36.54, 36.31, 30.87, 29.18, 28.86, 28.53, 28.37, 27.80, 27.25, 26.66, 24.29, 22.24, 21.95, 13.93. Conformers detected. ^{19}F NMR (376 MHz, DMSO) δ -73.56. LC-MS (ESI, m/z) calcd for $C_{18}H_{33}N_4O_5^+$ $[M+H]^+$: 385.2445, found: 385.4.

Compound 41 (4-((2-((2-aminoethyl)amino)-2-oxoethyl)(benzyl)amino)-4-oxobutanoyl)-L-alanine

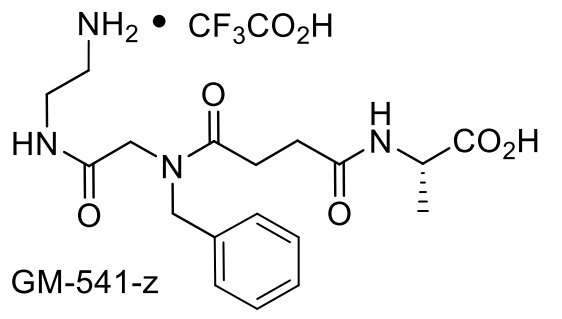

Obtained as a very hygroscopic light-yellow oil, yield 27% (0.311 g). ^{1}H NMR (400 MHz, DMSO) δ 12.45 (m, 1H, CO_2H), [8.23 (t, J = 5.7 Hz), 8.01 (t, J = 5.8 Hz), 1H, NH], [8.21 (d, J = 7.3 Hz), 8.19 (d, J = 7.5 Hz), 1H, NH], 7.92-7.71 (s, 3H, NH_3), 7.40-7.18 (m, 5H), [4.64 (s), 4.52 (d, J = 15.3 Hz), 4.46 (d, J = 15.4 Hz), 2H], [4.20 (p, J = 7.3 Hz), 4.18 (p, J = 7.3 Hz), 1H], [3.97 (d, J = 17.9 Hz), 3.91 (d, J = 17.8 Hz), 3.88 (d, J = 16.3 Hz), 3.83 (d, J = 16.4 Hz), 2H], 3.34-3.27 (m, 2H), 2.90-2.82 (m, 2H), 2.65-2.33 (m, 4H), [1.26 (d, J = 7.3 Hz), 1.24 (d, J = 7.3 Hz), 3H]. Conformers detected. ^{13}C NMR (101 MHz, DMSO) δ 174.24, 172.43, 172.40, 171.35, 171.33, 169.12, 168.96, 158.16 (q, J = 30.9 Hz), 157.69, 137.62, 137.21, 128.71, 128.36, 127.48, 127.30, 126.99, 126.64, 117.25 (q, J = 300.3 Hz), 51.46, 49.71, 49.12, 48.80, 47.46, 38.65, 38.58, 36.54, 36.35, 30.33, 30.10, 27.58, 27.55, 17.29, 17.25. Conformers detected. ^{19}F NMR (376 MHz, DMSO) δ -73.55. LC-MS (ESI, m/z) calcd for $C_{18}H_{27}N_4O_5^+$ $[M+H]^+$: 379.1976, found: 379.3.

Compound 42 (4-((2-((2-aminoethyl)amino)-2-oxoethyl)(benzyl)amino)-4-oxobutanoyl)-L-leucine

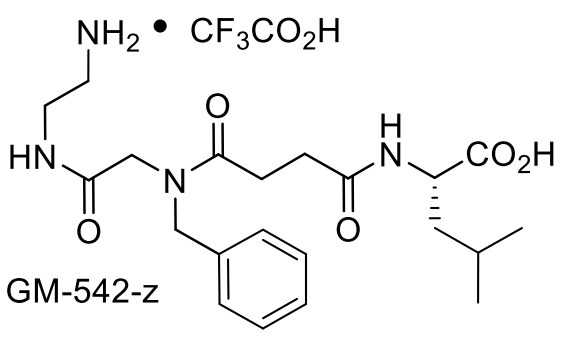

Obtained as a very hygroscopic light-yellow oil, yield 33% (0.434 g). ^{1}H NMR (400 MHz, $CDCl_3$) δ [8.47 (t, J = 6.0 Hz), 8.13 (t, J = 6.0 Hz), 1H, NH], [7.92 (d, J = 7.5 Hz), 7.86 (d, J = 8.1 Hz), 1H, NH], 8.5-6.5 (s, 3H, NH3), 7.40-7.18 (m, 5H), [4.74 (d, J = 17.2 Hz), 4.61 (d, J = 17.6 Hz), 4.57 (d, J = 15.5 Hz), 4.42 (d, J = 15.3 Hz), 2H], 4.17-4.01 (m, 1H), 4.01-3.58 (m, 2H), 3.38-3.22 (m, 2H), 2.90-2.77 (m, 2H), 2.51-2.36 (m, 4H), 1.68-1.56 (m, 1H), 1.54-1.36 (m, 2H), [0.87 (d, J = 6.5 Hz), 0.86 (d, J = 6.6 Hz), 0.84 (d, J = 6.6 Hz), 0.82 (d, J = 6.3 Hz), 6H]. Conformers detected. ^{13}C NMR (101 MHz, DMSO) δ 174.23, 174.21, 172.43, 172.36, 171.58, 169.13, 168.98, 158.24 (q, J = 33.7 Hz), 137.62, 137.21, 128.70, 128.34, 127.50, 127.29, 126.98, 126.63, 116.45 (q, J = 295.3 Hz), 51.44, 50.22, 49.69, 49.11, 38.68, 38.59, 38.26, 36.53, 36.35, 30.34, 30.15, 27.68, 27.60, 24.30, 22.85, 22.83, 21.41, 19.24, 9.67. Conformers detected. ^{19}F NMR (376 MHz, DMSO) δ -73.55. LC-MS (ESI, m/z) calcd for $C_{21}H_{33}N_4O_5^+$ $[M+H]^+$: 421.2445, found: 421.4.

Compound 43 (4-((2-((2-aminoethyl)amino)-2-oxoethyl)(benzyl)amino)-4-oxobutanoyl)-L-phenylalanine

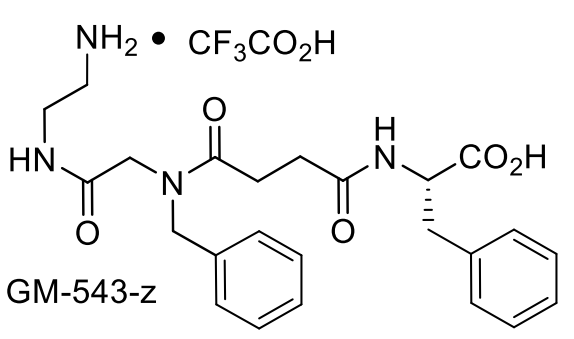

Hygroscopic compound that is rapidly transformed in a light yellow oil, yield 36% (0.495 g). ^{1}H NMR (400 MHz, DMSO) δ9.5-7.5 (s, 3H, NH3), [8.47 (t, J = 5.5 Hz), 8.10 (t, J = 5.9 Hz), 1H, NH], [7.97 (d, J = 8.0 Hz), 7.91 (d, J = 8.0 Hz), 1H, NH], 7.39-7.11 (m, 10H), [4.69 (d, J = 17.2 Hz), 4.58 (d, J = 17.3 Hz), 4.53 (d, J = 15.4 Hz), 4.43 (d, J = 15.3 Hz), 2H], 4.32-4.21 (m, 1H), [3.93 (d, J = 16.9 Hz), 3.88 (J = 17.7 Hz), 3.74 (d, J = 16.3 Hz), 2H], 3.39-3.23 (m,

2H), [3.06 (t, J = 4.5 Hz), 3.03 (t, J = 4.5 Hz), 2.88-2.78 (m), 2.72-2.65 (m), 4H], 2.59-2.28 (m, 4H). Conformers detected. ^{13}C NMR (101 MHz, DMSO) δ 173.04, 172.35, 172.28, 171.53, 171.49, 169.11, 168.92, 158.18 (q, J = 33.4 Hz), 137.66, 137.61, 137.59, 137.14, 129.14, 128.70, 128.35, 128.16, 128.11, 127.49, 127.30, 126.98, 126.59, 126.40, 126.38, 53.52, 53.45, 51.40, 49.66, 49.10, 48.74, 38.68, 38.60, 38.25, 36.87, 36.52, 36.34, 30.38, 30.25, 27.68, 27.59. Conformers detected. ^{19}F NMR (376 MHz, DMSO) δ -73.53. LC-MS (ESI, m/z) calcd for $C_{24}H_{31}N_4O_5^+$ [M+H]$^+$: 455.2289, found: 455.4.

Compound 44 (4-((2-((2-aminoethyl)amino)-2-oxoethyl)(benzyl)amino)-4-oxobutanoyl)-L-proline

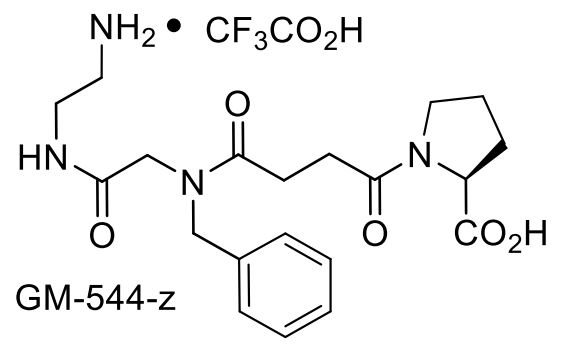

GM-544-z

Obtained as an off-white solid, yield 32% (0.395 g). ^{1}H NMR (400 MHz, DMSO) δ9.0-7.0 (s, 3H, NH3),[8.76 (t, J = 5.6 Hz), 8.64 (s), 8.28 (s), 7.90 (t, J = 5.9 Hz), 1H, NH], 7.40-7.19 (m, 5H), [4.96 (d, J = 17.0 Hz), 4.75 (d, J = 17.1 Hz) 4.60 (d, J = 17.2 Hz), 4.53 (d, J = 16.9 Hz), 4.52 (s), 4.46 (d, J = 15.4 Hz), 2H], 4.25-3.68 (m, 3H), 3.54-3.14 (m, 4H), 2.92-2.26 (m, 6H), 2.18-1.67 (m, 4H). Conformers detected. ^{13}C NMR (101 MHz, DMSO) δ 173.78, 173.50, 172.51, 170.49, 170.12, 169.18, 169.00, 158.39 (d, J = 33.4 Hz),157.73, 137.60, 137.26, 128.69, 128.36, 127.53, 127.29, 126.97, 126.74, 126.69, 116.56 (q, J = 296.0 Hz), 58.74, 58.36, 51.55, 49.81, 49.18, 48.93, 46.44, 46.07, 38.73, 38.61, 36.54, 36.33, 29.31, 29.10, 28.88, 27.09, 24.30, 24.27, 22.25.Conformers detected. ^{19}F NMR (376 MHz, DMSO) δ -73.52. LC-MS (ESI, m/z) calcd for $C_{20}H_{29}N_4O_5^+$ [M+H]$^+$: 405.2132, found: 405.3.

Compound 45 (4-((2-((2-aminoethyl)amino)-2-oxoethyl)(furan-2-ylmethyl)amino)-4-oxobutanoyl)-L-alanine

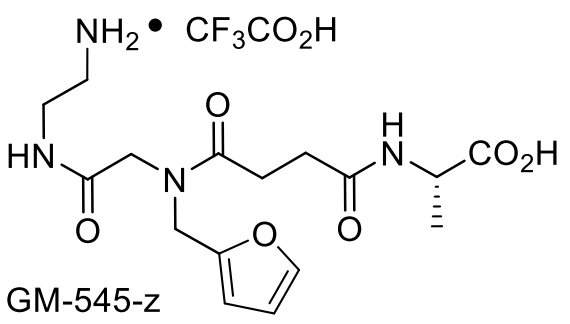

GM-545-z

Obtained as a yellow solid, yield 32% (0.342 g). ^{1}H NMR (400 MHz, DMSO) δ [8.73 (t, J = 5.6 Hz, 1H), 8.28 (t, J = 5.7 Hz), 1H, NH], [7.82 (d, J = 7.1 Hz), 7.65 (d, J = 7.2 Hz), 1H, NH], [7.62 (d, J = 1.8 Hz), 7.55 (d, J = 1.8 Hz), 1H], 7.5-5.5 (s, 3H, NH3), [6.42 (dd, J = 3.3, 1.9 Hz), 6.37 (dd, J = 3.2, 1.8 Hz), 1H], [6.40 (d, J = 3.3 Hz), 6.29 (d, J = 3.2 Hz), 1H], [4.68 (d, J = 16.9 Hz), 4.56 (d, J = 17.0 Hz), 4.53 (d, J = 15.4 Hz), 4.43 (d, J = 15.6 Hz), 2H], [3.99 (d, J = 17.9 Hz), 4.00-3.87 (m), 3.73 (d, J = 16.5 Hz), 3H], 3.37-3.22 (m, 2H), 2.86-2.77 (m, 2H), 2.62-2.33 (m, 4H), 1.16 (d, J = 7.1 Hz, 3H). Conformers detected. ^{13}C NMR (101 MHz, DMSO) δ 174.24, 172.08, 172.01, 171.32, 171.27, 169.04, 168.84, 158.12 (q, J = 31.2 Hz), 150.87, 150.56, 143.05, 142.56, 110.55, 110.44, 108.25, 108.22, 49.46, 48.29, 47.46, 47.43, 44.84, 42.22, 38.66, 38.61, 36.55, 36.36, 30.13, 27.63, 17.28, 17.25. ^{19}F NMR (376 MHz, DMSO) δ -73.49. Conformers detected. LC-MS (ESI, m/z) calcd for $C_{16}H_{25}N_4O_6^+$ [M+H]$^+$: 369.1769, found: 369.3.

Compound 46 (4-((2-((2-aminoethyl)amino)-2-oxoethyl)(furan-2-ylmethyl)amino)-4-oxobutanoyl)-L-leucine

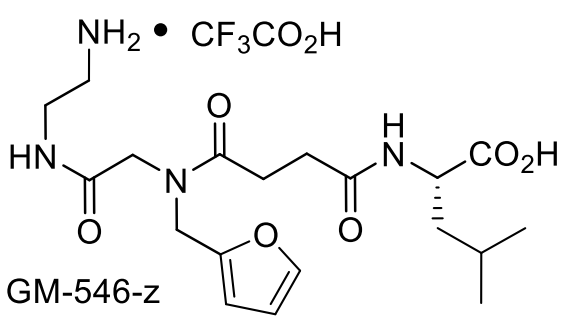

GM-546-z

Obtained as a pale yellow solid, yield 30% (0.331 g). ^{1}H NMR (400 MHz, DMSO) δ [8.73 (t, J = 5.6 Hz), 8.32 (t, J = 5.7 Hz), 1H, NH], [7.84 (d, J = 8.0 Hz), 7.64 (d, J = 8.0 Hz), 1H, NH], [7.62 (d, J = 1.8

Hz), 7.55 (d, J = 1.8 Hz), 1H], [6.42 (dd, J = 3.2, 1.8 Hz), 6.37 (dd, J = 3.2, 1.9 Hz), 1H], [6.40 (d, J = 3.2 Hz), 6.30 (d, J = 3.2 Hz), 1H], 7.0-5.0 (s, 3H, NH3), [4.70 (d, J = 16.9 Hz), 4.56 (d, J = 15.5 Hz), 4.53 (d, J = 17.0 Hz) 4.41 (d, J = 15.5 Hz), 2H], 4.05-3.56 (m, 3H), 3.36-3.10 (m, 2H), 2.90-2.72 (m, 2H), 2.57-2.32 (m, 4H), 1.68-1.56 (m, 1H), 1.53-1.34 (m, 2H), [0.85 (d, J = 6.6 Hz), 0.85 (d, J = 6.4 Hz), 0.82 (d, J = 6.7 Hz), 0.82 (d, J = 6.6 Hz), 6H]. Conformers detected. ^{13}C NMR (101 MHz, DMSO) δ 174.23, 172.04, 172.02, 171.57, 171.52, 169.06, 168.87, 158.45 (q, J = 33.4 Hz), 158.12, 157.80, 150.90, 150.57, 143.04, 142.54, 116.56 (q, J = 296.6 Hz), 110.55, 110.44, 108.25, 108.19, 50.26, 50.23, 49.48, 48.26, 44.82, 42.24, 38.67, 38.62, 36.56, 36.37, 30.18, 27.74, 27.67, 24.32, 24.30, 22.84, 21.42. Conformers detected. ^{19}F NMR (376 MHz, DMSO) δ -73.50. LC-MS (ESI, m/z) calcd for $C_{19}H_{31}N_4O_6^+$ $[M+H]^+$: 411.2238, found: 411.3.

Compound 47 (4-((2-((2-aminoethyl)amino)-2-oxoethyl)(furan-2-ylmethyl)amino)-4-oxobutanoyl)-L-phenylalanine

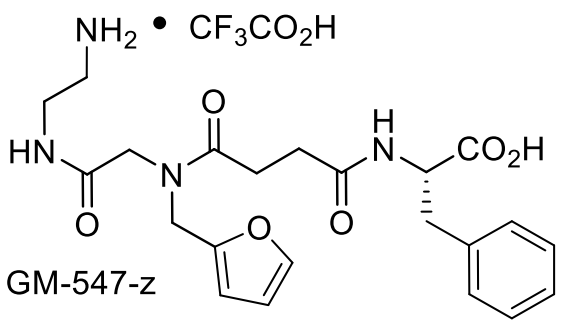

Hygroscopic compound that is rapidly transformed in a pale yellow oil, yield 22% (0.256 g). ^{1}H NMR (400 MHz, DMSO) δ [8.72 (t, J = 5.6 Hz), 8.16 (t, J = 5.8 Hz), 1H, NH], [7.83 (d, J = 7.9 Hz), 7.65 (d, J = 8.1 Hz), 1H, NH], [7.62 (d, J = 1.8 Hz), 7.55 (d, J = 1.8 Hz), 1H], 7.30-7.10 (m, 5H), 6.43-6.28 (m, 2H), [4.68 (d, J = 16.9 Hz), 4.53 (d, J = 15.8 Hz), 4.52 (d, J = 16.7 Hz), 4.40 (d, J = 15.5 Hz), 2H], [4.15 (td, J = 8.0, 4.6 Hz), 4.13 (td, J = 8.1, 4.6 Hz), 1H], 4.00-3.56 (m, 2H), 3.41-3.16 (m, 2H), 3.13-2.71 (m, 4H), 2.55-2.21 (m, 4H). Conformers detected. ^{13}C NMR (101 MHz, DMSO) δ 173.05, 171.97, 171.94, 171.49, 171.46, 169.03, 168.80, 158.22 (q, J = 33.0 Hz), 157.73, 150.86, 150.51, 143.05, 142.55, 137.65, 129.15, 128.44, 128.15, 126.40, 110.56, 110.44, 108.23, 53.49, 49.43, 48.25, 44.80, 42.20, 38.67, 38.62, 36.89, 36.55, 36.36, 30.28, 30.22, 27.74, 27.67, 19.25. Conformers detected. ^{19}F NMR (376 MHz, DMSO) δ -73.48. LC-MS (ESI, m/z) calcd for $C_{22}H_{29}N_4O_6^+$ $[M+H]^+$: 445.2082, found: 445.3.

Compound 48 (4-((2-((2-aminoethyl)amino)-2-oxoethyl)(furan-2-ylmethyl)amino)-4-oxobutanoyl)-L-proline

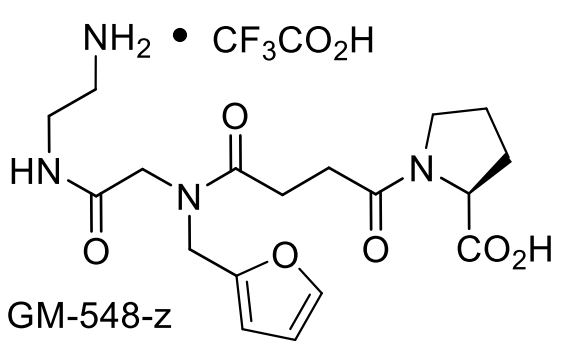

Obtained as a pale yellow solid, yield 38% (0.337 g). ^{1}H NMR (400 MHz, DMSO) δ [8.82 (t, J = 5.6 Hz), 8.72 (t, J = 5.2 Hz), 8.39 (t, J = 5.1 Hz), 7.86 (t, J = 6.0 Hz), 1H, NH], [7.63 (d, J = 1.4 Hz), 7.63 (d, J = 1.4 Hz), 7.56 (d, J = 1.4 Hz), 7.56 (d, J = 1.4 Hz), 1H], 6.42 (d, J = 1.5 Hz, 1H), [6.39-6.35 (m), 6.31-6.29 (m), 1H], [4.93 (d, J = 16.8 Hz), 4.68 (d, J = 16.9 Hz), 4.59-4.44 (m), 2H], 4.20-3.09 (m, 7H), 2.93-2.71 (m, 2H), 2.67-2.19 (m, 4H), 2.11-1.66 (m, 4H). Conformers detected. ^{13}C NMR (101 MHz, DMSO) δ 173.76, 173.51, 172.22, 172.08, 170.46, 170.09, 170.04, 169.09, 168.88, 158.43 (q, J = 33.8 Hz), 157.80, 150.86, 150.61, 150.57, 143.03, 142.56, 116.41 (q, J = 296.0 Hz), 110.57, 110.45, 108.27, 108.22, 58.74, 58.38, 49.57, 48.45, 46.44, 46.07, 44.91, 42.26, 40.15, 39.94, 39.73, 39.62, 39.52, 39.31, 39.10, 38.90, 38.73, 38.64, 36.56, 36.34, 30.88, 29.11, 28.87, 27.19, 27.06, 24.29, 22.25, 19.24. Conformers detected. ^{19}F NMR (376 MHz, DMSO) δ -73.51. LC-MS (ESI, m/z) calcd for $C_{18}H_{27}N_4O_6^+$ $[M+H]^+$: 395.1925, found: 395.3.

Compound 49 (4-((2-(benzylamino)-2-oxoethyl)(isobutyl)amino)-4-oxobutanoyl)-L-alanine

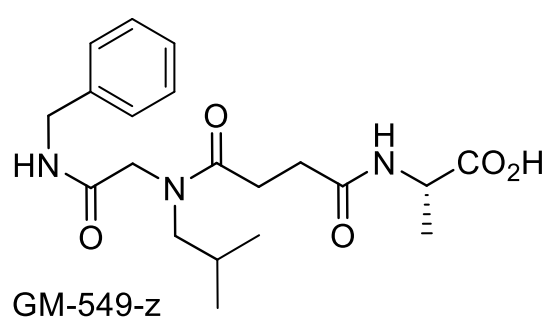
GM-549-z

Obtained as a hygroscopic off-white solid, yield 30% (0.272 g). ^{1}H NMR (400 MHz, DMSO) δ 12.45 (s, 1H, CO_2H), [8.55 (t, *J* = 5.9 Hz), 8.23 (t, *J* = 6.0 Hz), 1H, NH], [8.17 (d, *J* = 7.3 Hz), 8.12 (d, *J* = 7.3 Hz), 1H, NH], 7.34-7.20 (m, 5H), [4.30 (d, *J* = 5.9 Hz), 4.26 (t, *J* = 6.7 Hz), 2H], [4.17 (p, *J* = 7.3 Hz), 4.13 (p, *J* = 7.1 Hz), 1H], [4.06 (d, *J* = 17.5 Hz), 4.01 (d, *J* = 17.4 Hz), 3.91 (s), 2H], 3.21-3.02 (m, 2H), 2.63- 2.29 (m, 4H),1.91-1.76 (m, 1H), [1.24 (d, *J* = 7.1 Hz), 1.22 (d, *J* = 7.4 Hz), 3H], [0.87 (d, *J* = 6.6 Hz), 0.79 (d, *J* = 6.7 Hz), 0.78 (d, *J* = 6.7 Hz), 6H]. Conformers detected. ^{13}C NMR (101 MHz, DMSO) δ 174.26, 174.24, 172.18, 172.15, 171.49, 171.30, 168.45, 168.38, 139.47, 139.16, 128.32, 128.19, 127.29, 127.08, 126.85, 126.65, 55.62, 53.70, 50.64, 49.37, 47.47, 42.27, 41.94, 30.50, 30.41, 27.97, 27.45, 27.35, 26.38, 20.01, 19.99, 19.78, 17.29, 17.24. Conformers detected. LC-MS (ESI, m/z) calcd for $C_{20}H_{30}N_3O_5^+$ [M+H]$^+$: 392.2180, found: 392.3.

Compound 50 (4-((2-(benzylamino)-2-oxoethyl)(isobutyl)amino)-4-oxobutanoyl)-L-leucine

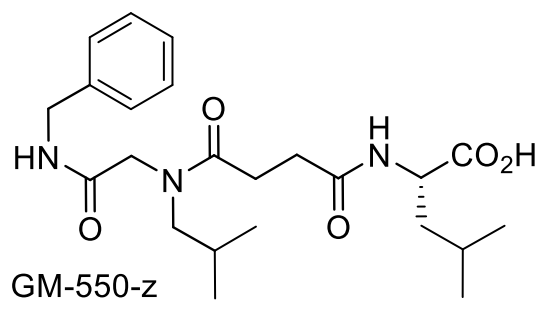
GM-550-z

Obtained as a white solid, yield 32% (0.313 g). ^{1}H NMR (400 MHz, DMSO) δ 12.48 (s, 1H, CO_2H), [8.55 (t, *J* = 5.9 Hz), 8.25 (t, *J* = 6.0 Hz), 1H, NH], [8.13 (d, *J* = 7.9 Hz), 8.07 (d, *J* = 7.9 Hz), 1H, NH], 7.34-7.21 (m, 5H), [4.30 (d, *J* = 5.9 Hz), 4.26 (t, *J* = 5.5 Hz), 2H], 4.23-4.15 (m, 1H), [4.07 (d, *J* = 17.6 Hz), 4.01 (d, *J* = 17.5 Hz), 3.91 (s), 2H], [3.15 (d, *J* = 7.2 Hz), 7.12 (dd, *J* = 13.3, 7.6 Hz), 3.02 (dd, *J* = 13.3, 7.3 Hz), 2H], 2.62-2.30 (m, 4H), 1.93-1.75 (m, 1H), 1.68-1.57 (m, 1H), 1.54-1.43 (m, 2H), [0.88 (d, *J* = 6.7 Hz), 0.87 (d, *J* = 6.6 Hz), 0.87 (d, *J* = 6.7 Hz), 0.83 (d, *J* = 6.6 Hz), 0.82 (d, *J* = 6.6 Hz), 0.79 (d, *J* = 6.7 Hz), 0.78 (d, *J* = 6.7 Hz), 12H]. Conformers detected. ^{13}C NMR (101 MHz, DMSO) δ 174.24, 174.19, 172.17, 172.11, 171.75, 171.54, 168.46, 168.37, 139.48, 139.16, 128.31, 128.18, 127.28, 127.08, 126.84, 126.65, 55.61, 53.69, 50.65, 50.27, 50.18, 49.35, 42.26, 41.94, 30.52, 30.47, 27.97, 27.59, 27.33, 26.38, 24.31, 24.25, 22.86, 22.78, 21.42, 21.39, 20.02, 19.98, 19.78. Conformers detected. LC-MS (ESI, m/z) calcd for $C_{23}H_{36}N_3O_5^+$ [M+H]$^+$: 434.2649, found: 434.4.

Compound 51 (4-((2-(benzylamino)-2-oxoethyl)(isobutyl)amino)-4-oxobutanoyl)-L-phenylalanine

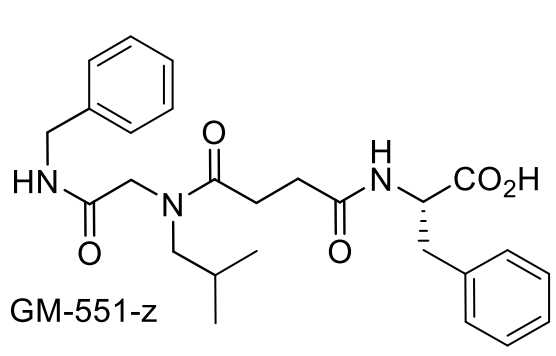
GM-551-z

Obtained as a white solid, yield 42% (0.484 g). ^{1}H NMR (400 MHz, DMSO) δ 12.68 (s, 1H, CO_2H), [8.54 (t, *J* = 5.9 Hz), 8.23 (t, *J* = 6.2 Hz), 1H, NH], [8.24 (d, *J* = 8.4 Hz), 8.18 (d, *J* = 8.1 Hz), 1H, NH], 7.34-7.17 (m, 10H), [4.41 (dt, *J* = 7.8, 4.5 Hz), 4.38 (dt, *J* = 8.0, 3.1 Hz), 1H], [4.30 (d, *J* = 5.8 Hz), 4.26 (d, *J* = 6.0 Hz), 4.25 (d, *J* = 6.0 Hz), 2H], [4.01 (s), 3.90 (s), 2H], [3.14-3.00 (m), 2.87-2.80 (m), 4H], 2.50-2.29 (m, 4H), 1.91-1.75 (m, 1H), [0.85 (d, *J* = 6.6 Hz), 0.78 (d, *J* = 6.7 Hz), 0.78 (d, *J* = 6.7 Hz), 6H]. Conformers detected. ^{13}C NMR (101 MHz, DMSO) δ 173.06, 173.03, 172.12, 171.99, 171.67, 171.50, 168.39, 168.36, 139.47, 139.15, 137.68, 137.65, 129.13, 128.32, 128.20, 128.12, 127.29, 127.08, 126.86, 126.66, 126.37, 59.76, 55.58, 53.69, 53.47, 50.62, 49.32, 42.27, 41.94, 36.89, 30.57, 28.00, 27.64, 27.29, 26.37, 20.77, 20.01, 19.78, 14.09. Conformers detected. LC-MS (ESI, m/z) calcd for $C_{26}H_{34}N_3O_5^+$ [M+H]$^+$: 468.2493, found: 468.3.

Compound 52 (4-((2-(benzylamino)-2-oxoethyl)(isobutyl)amino)-4-oxobutanoyl)-L-proline

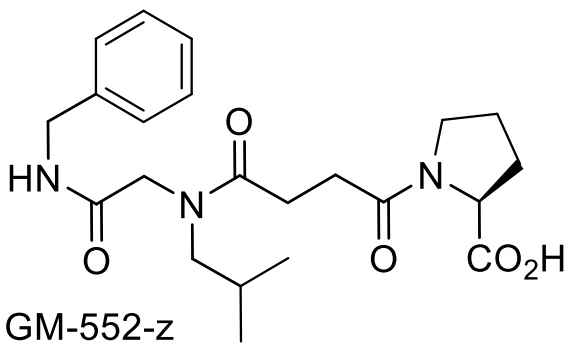

GM-552-z

Obtained as a hygroscopic yellow oil, yield 48% (0.451 g). ^{1}H NMR (400 MHz, DMSO) δ 12.39 (m, 1H, CO_2H), [8.56 (t, *J* = 4.4 Hz), 8.55 (t, *J* = 6.0 Hz), 8.18 (t, *J* = 6.1 Hz), 8.13 (t, *J* = 6.1 Hz), 1H, NH], 1.34-1.20 (m, 5H), 4.54-3.85 (m, 5H), [3.57-3.46 (m), 3.38-3.00 (m), 4H], 2.72-2.37 (m, 4H), [2.27-1.98 (m), 1.93-1.67 (m), 5H], [0.88 (d, *J* = 6.7 Hz), 0.87 (d, *J* = 6.6 Hz), 0.79 (d, *J* = 6.7 Hz), 0.78 (d, *J* = 6.7 Hz), 6H]. Conformers detected. ^{13}C NMR (101 MHz, DMSO) δ 173.84, 173.72, 173.50, 173.41, 172.38, 172.20, 172.01, 170.68, 170.37, 170.05, 168.46, 168.37, 139.52, 139.43, 139.18, 128.31, 128.16, 127.32, 127.31, 127.03, 126.98, 126.85, 126.61, 59.76, 58.68, 58.31, 58.27, 55.78, 55.74, 53.79, 53.75, 50.77, 50.73, 49.69, 49.61, 46.39, 46.02, 45.91, 42.26, 41.86, 28.80, 27.54, 27.48, 27.36, 27.25, 26.82, 26.41, 24.30, 24.23, 22.26, 22.17, 21.05, 20.76, 20.05, 20.02, 19.80, 14.09. Conformers detected. LC-MS (ESI, m/z) calcd for $C_{22}H_{32}N_3O_5^+$ $[M+H]^+$: 418.2336, found: 418.3.

Compound 53 (4-((2-(benzylamino)-2-oxoethyl)(pentyl)amino)-4-oxobutanoyl)-L-alanine

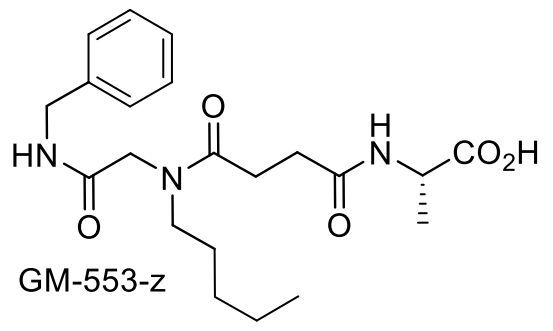

GM-553-z

Obtained as a white solid, yield 34% (0.335 g). ^{1}H NMR (400 MHz, DMSO) δ 12.52 (m, 1H, CO_2H), [8.56 (t, *J* = 5.9 Hz), 8.23 (t, *J* = 6.1 Hz), 1H, NH], [8.16 (d, *J* = 7.3 Hz), 8.12 (d, *J* = 7.3 Hz), 1H, NH], 7.36-7.19 (m, 5H), [4.30 (d, *J* = 5.9 Hz), 4.27 (d, *J* = 5.3 Hz), 4.25 (d, *J* = 5.3 Hz), 2H], [4.17 (p, *J* = 7.3 Hz), 4.14 (p, *J* = 7.3 Hz), 1H], [4.05 (d, *J* = 17.5 Hz), 4.00 (d, *J* = 17.4 Hz), 3.90 (s), 2H], 3.31-3.16 (m, 2H), 2.63-2.29 (m, 4H), [1.52 (p, *J* = 7.7 Hz), 1.38 (p, *J* = 7.5 Hz), 2H], 1.32-1.12 (m, 4H), [1.24 (d, *J* = 7.3 Hz), 1.22 (d, *J* = 7.3 Hz), 3H], [0.86 (t, *J* = 7.1 Hz), 0.84 (t, *J* = 7.2 Hz), 3H]. Conformers detected. ^{13}C NMR (101 MHz, DMSO) δ 174.27, 174.25, 171.74, 171.70, 171.44, 171.29, 168.52, 168.50, 139.48, 139.19, 128.30, 128.18, 127.28, 127.08, 126.84, 126.65, 50.27, 48.88, 48.57, 47.50, 46.59, 42.26, 41.94, 30.41, 30.29, 28.53, 28.37, 27.92, 27.78, 27.27, 26.68, 21.94, 21.92, 17.32, 17.28, 13.91. Conformers detected. LC-MS (ESI, m/z) calcd for $C_{21}H_{32}N_3O_5^+$ $[M+H]^+$: 406.2336 , found: 406.3.

Compound 54 (4-((2-(benzylamino)-2-oxoethyl)(pentyl)amino)-4-oxobutanoyl)-L-leucine

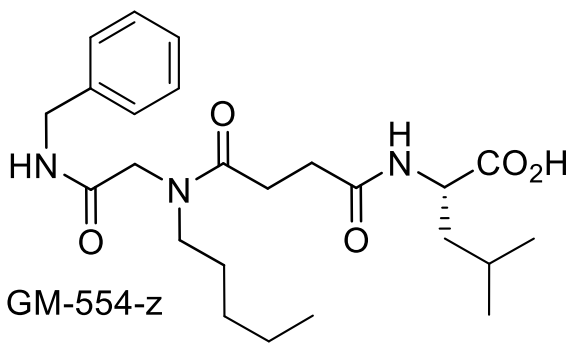

GM-554-z

Obtained as a white solid, yield 14% (0.154 g). ^{1}H NMR (400 MHz, DMSO) δ 12.47 (s, 1H, CO_2H), [8.56 (t, *J* = 5.9 Hz), 8.24 (t, *J* = 6.1 Hz), 1H, NH], [8.13 (d, *J* = 7.9 Hz), 8.07 (d, *J* = 8.0 Hz), 1H, NH], 7.34-7.20 (m, 5H), 4.32-4.16 (m, 3H), [4.05 (d, *J* = 17.7 Hz), 3.99 (d, *J* = 17.6 Hz), 3.90 (s), 2H], 3.31-3.13 (m, 2H), 2.60-2.30 (m, 4H), 1.69-1.34 (m, 5H), 1.31-1.12 (m, 4H), 0.89-0.81 (m, 9H). Conformers detected. ^{13}C NMR (101 MHz, DMSO) δ 174.23, 174.19, 171.72, 171.65, 171.55, 168.53, 168.49, 139.48, 139.18, 128.29, 128.18, 127.27, 127.07, 126.83, 126.65, 50.23, 50.18, 48.86, 48.56, 46.61, 42.26, 41.94, 30.69, 30.43, 30.37, 28.52, 28.36, 27.92, 27.76, 27.42, 26.66, 24.31, 24.26, 22.85, 22.80, 21.93, 21.91, 21.39, 13.90. Conformers detected. LC-MS (ESI, m/z) calcd for $C_{24}H_{38}N_3O_5^+$ $[M+H]^+$: 448.2806, found: 448.4.

Compound 55 (4-((2-(benzylamino)-2-oxoethyl)(pentyl)amino)-4-oxobutanoyl)-L-phenylalanine

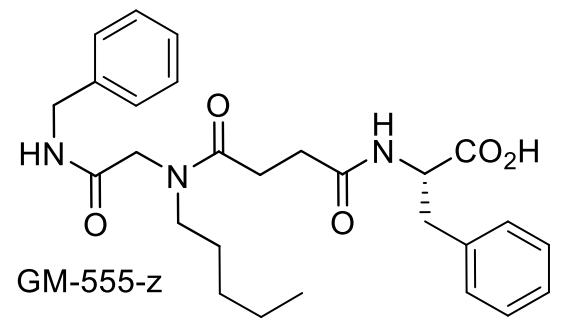

Obtained as a hygroscopic light-yellow solid, yield 21% (0.252 g). ^{1}H NMR (400 MHz, DMSO) δ 12.68 (s, 1H, CO_2H), [8.55 (t, *J* = 5.9 Hz), 8.23 (t, *J* = 5.9 Hz), 1H, NH], [8.23 (d, *J* = 8.1 Hz), 8.18 (d, *J* = 8.1 Hz), 1H, NH], 7.33-7.16 (m, 10H), [4.40 (dt, *J* = 8.2, 4.3 Hz), 4.40 (td, *J* = 8.8, 5.2 Hz), 1H], [4.30 (d, *J* = 5.9 Hz), 4.25 (d, *J* = 6.1 Hz), 2H], [3.99 (s), 3.90 (s), 2H], 3.27-3.17 (m, 2H), [3.03 (dd, *J* = 13.8, 5.1 Hz), 2.88-2.80 (m), 2H], 2.49-2.28 (m, 4H), [1.50 (p, *J* = 7.6 Hz), 1.37 (p, *J* = 7.6 Hz), 2H], 1.31-1.12 (m, 4H), [0.86 (t, *J* = 7.1 Hz), 0.83 (t, *J* = 7.2 Hz), 3H]. Conformers detected. ^{13}C NMR (101 MHz, DMSO) δ 173.05, 173.03, 171.66, 171.64, 171.56, 171.50, 168.48, 139.48, 139.17, 137.67, 129.13, 128.30, 128.18, 128.11, 127.28, 127.07, 126.84, 126.66, 126.35, 53.47, 50.23, 48.83, 48.52, 46.58, 42.26, 41.93, 36.89, 36.88, 30.48, 30.45, 28.52, 28.37, 27.96, 27.75, 27.44, 26.66, 21.93, 13.90. Conformers detected. LC-MS (ESI, m/z) calcd for $C_{27}H_{36}N_3O_5^+$ $[M+H]^+$: 482.2649, found: 482.4.

Compound 56 (4-((2-(benzylamino)-2-oxoethyl)(pentyl)amino)-4-oxobutanoyl)-L-proline

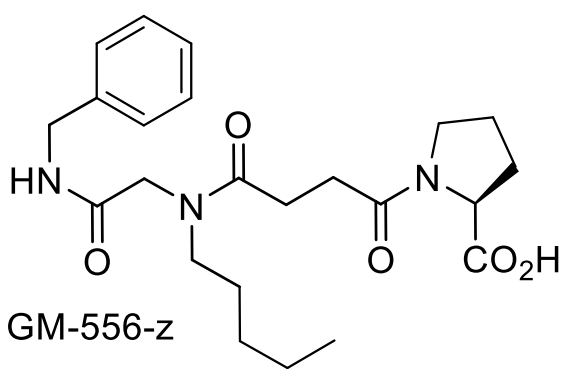

Obtained as a hygroscopic off-white solid, yield 35% (0.361 g). ^{1}H NMR (400 MHz, DMSO) δ 12.50 (s, 1H, CO_2H), [8.57 (t, *J* = 5.9 Hz), 8.18 (t, *J* = 7.4 Hz), 8.15 (t, *J* = 6.1 Hz), 1H, NH], 7.33-7.20 (m, 5H), [4.53-4.49 (m), 4.35-4.17 (m), 4.12-3.98 (m), 3.93 (d, *J* = 16.3 Hz), 3.88 (d, *J* = 16.3 Hz), 5H], 3.58-3.47 (m, 2H), 3.28-3.14 (m, 2H), 2.70-1.66 (m, 8H), [1.53 (p, *J* = 7.6 Hz), 1.38 (p, *J* = 7.6 Hz), 2H], 1.31-1.12 (m, 4H), [0.87 (t, *J* = 7.1 Hz), 0.86 (t, *J* = 7.0 Hz), 0.84 (t, *J* = 7.1 Hz), 3H]. Conformers detected. ^{13}C NMR (101 MHz, DMSO) δ 173.77, 173.51, 173.44, 171.89, 171.77, 170.64, 170.41, 170.32, 170.07, 168.53, 168.49, 139.52, 139.45, 139.21, 128.30, 128.16, 127.32, 127.30, 127.07, 127.01, 126.84, 126.62, 58.78, 58.33, 58.30, 50.40, 49.13, 48.72, 46.68, 46.39, 45.93, 42.27, 41.89, 30.84, 29.39, 29.28, 29.24, 29.14, 28.86, 28.82, 28.54, 27.87, 27.85, 27.33, 27.08, 26.69, 26.66, 24.30, 24.25, 22.26, 22.20, 21.93, 13.91. Conformers detected. LC-MS (ESI, m/z) calcd for $C_{23}H_{34}N_3O_5^+$ $[M+H]^+$: 432.2493, found: 432.3.

Compound 57 (4-(benzyl(2-(benzylamino)-2-oxoethyl)amino)-4-oxobutanoyl)-L-alanine

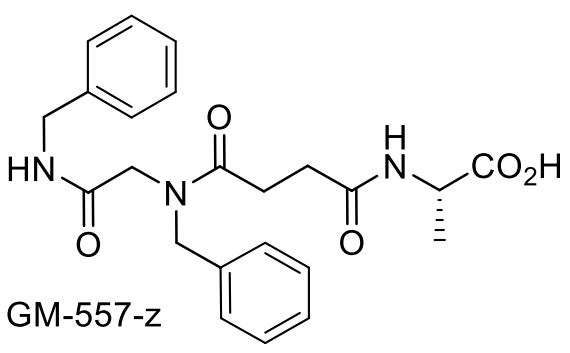

Obtained as a white solid, yield 40% (0.475 g). ^{1}H NMR (400 MHz, DMSO) δ 12.50 (s, 1H, CO_2H), [8.54 (t, *J* = 5.9 Hz), 8.32 (t, *J* = 6.0 Hz), 1H, NH], [8.17 (d, *J* = 7.2 Hz), 8.17 (d, *J* = 7.3 Hz), 1H, NH], 7.39-7.20 (m, 10H), [4.64 (s), 4.53 (d, *J* = 15.3 Hz), 4.45 (d, *J* = 15.3 Hz), 2H], 4.31-4.26 (m, 2H), [4.20 (p, *J* = 7.2 Hz), 4.15 (p, *J* = 7.3 Hz), 1H], [4.02 (d, *J* = 17.7 Hz), 3.96 (d, *J* = 17.9 Hz), 3.89 (s), 2H], 2.67-2.33 (m, 4H), [1.25 (d, *J* = 7.3 Hz), 1.22 (d, *J* = 7.3 Hz), 3H]. Conformers detected. ^{13}C NMR (101 MHz, DMSO) δ 174.26, 174.24, 172.43, 172.32, 171.32, 171.25, 168.14, 168.08, 139.37, 139.08, 137.61, 137.25, 128.68, 128.34, 128.23, 127.58, 127.29, 127.24, 127.08, 126.98, 126.84, 126.78, 126.69, 51.60, 49.65, 49.29, 48.74, 47.48, 47.45, 42.27, 41.97, 30.43, 30.17, 27.75, 27.60, 17.30, 17.26. Conformers detected. LC-MS (ESI, m/z) calcd for $C_{23}H_{28}N_3O_5^+$ $[M+H]^+$: 426.2023, found: 426.3.

Compound 58 (4-(benzyl(2-(benzylamino)-2-oxoethyl)amino)-4-oxobutanoyl)-L-leucine

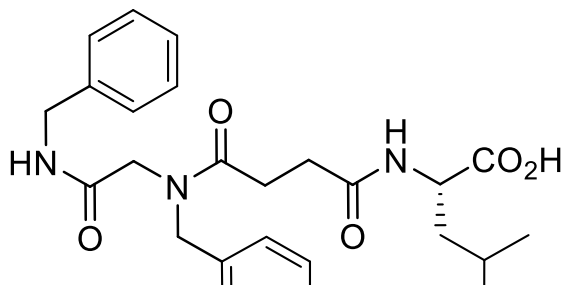

GM-558-z

Obtained as a white solid, yield 12% (0.165 g). ^{1}H NMR (400 MHz, DMSO) δ 12.50 (s, 1H, CO_2H), [8.55 (t, *J* = 5.9 Hz), 8.33 (t, *J* = 6.0 Hz), 1H, NH], 8.11 (d, *J* = 7.9 Hz, 1H, NH), 7.39-7.20 (m, 10H), [4.63 (s), 4.54 (d, *J* = 15.3 Hz), 4.44 (d, *J* = 15.3 Hz), 2H], 4.29 (t, *J* = 6.1 Hz, 2H), [4.22 (td, *J* = 8.2, 5.0 Hz), 4.19 (td, *J* = 8.0, 5.5 Hz), 1H], [4.02 (d, *J* = 17.8 Hz), 3.96 (d, *J* = 17.6 Hz), 3.90 (s), 2H], 2.67-2.32 (m, 4H), 1.70-1.56 (m, 1H), 1.54-1.44 (m, 2H), [0.89 (d, *J* = 6.7 Hz), 0.87 (d, *J* = 6.7 Hz), 0.84 (d, *J* = 6.5 Hz), 0.81 (d, *J* = 6.5 Hz), 6H]. Conformers detected. ^{13}C NMR (101 MHz, DMSO) δ 174.26, 174.22, 172.42, 172.29, 171.55, 171.47, 168.14, 168.09, 139.38, 139.07, 137.61, 137.24, 128.67, 128.33, 128.22, 127.60, 127.28, 127.23, 127.08, 126.97, 126.83, 126.78, 126.68, 51.61, 50.24, 49.64, 49.28, 48.75, 42.27, 41.97, 30.44, 30.24, 27.77, 24.29, 24.00, 22.86, 22.81, 21.42. Conformers detected. LC-MS (ESI, m/z) calcd for $C_{26}H_{34}N_3O_5^+$ [M+H]$^+$: 468.2493, found: 468.4.

Compound 59 (4-(benzyl(2-(benzylamino)-2-oxoethyl)amino)-4-oxobutanoyl)-L-phenylalanine

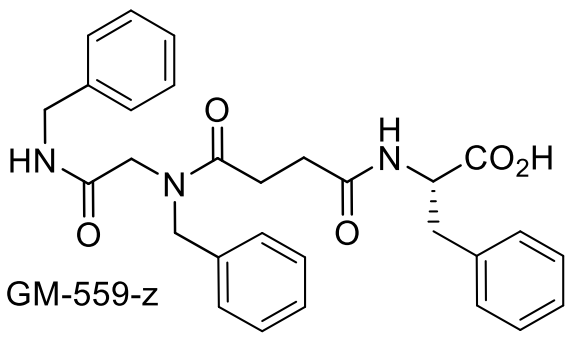

GM-559-z

Obtained as a white solid, yield 27% (0.551 g). ^{1}H NMR (400 MHz, DMSO) δ 12.69 (s, 1H, CO_2H), [8.53 (t, *J* = 5.9 Hz), 8.32 (t, *J* = 6.0 Hz), 1H, NH], 8.22 (d, *J* = 8.0 Hz, 1H, NH), 7.38-7.14 (m, 15H), [4.60 (s), 4.50 (*J* = 15.1 Hz), 4.44 (*J* = 15.1 Hz), 2H], [4.43 (td, *J* = 9.1, 4.6 Hz), 4.39 (dt, *J* = 9.3, 4.6 Hz), 1H], 4.28 (t, *J* = 6.6 Hz, 2H), [3.96 (s), 3.88 (s), 2H], [3.04 (dd, *J* = 9.5, 5.2 Hz), 3.01 (dd, *J* = 9.2, 4.9 Hz), 2.86 (dd, *J* = 13.4, 9.1 Hz), 2.82 (dd, *J* = 13.5, 9.2 Hz), 2H], 2.53-2.32 (m, 4H). Conformers detected. ^{13}C NMR (101 MHz, DMSO) δ 173.06, 173.02, 172.35, 172.19, 171.47, 171.43, 168.11, 168.01, 139.37, 139.05, 137.68, 137.63, 137.58, 137.18, 129.12, 128.68, 128.33, 128.22, 128.13, 128.09, 127.60, 127.28, 127.24, 127.07, 126.98, 126.84, 126.74, 126.69, 126.37, 126.34, 53.51, 53.45, 51.56, 49.60, 49.26, 48.71, 42.27, 41.96, 36.92, 30.50, 30.33, 27.79, 27.75, 0.11. Conformers detected. LC-MS (ESI, m/z) calcd for $C_{29}H_{32}N_3O_5^+$ [M+H]$^+$: 502.2336, found: 502.3.

Compound 60 (4-(benzyl(2-(benzylamino)-2-oxoethyl)amino)-4-oxobutanoyl)-L-proline

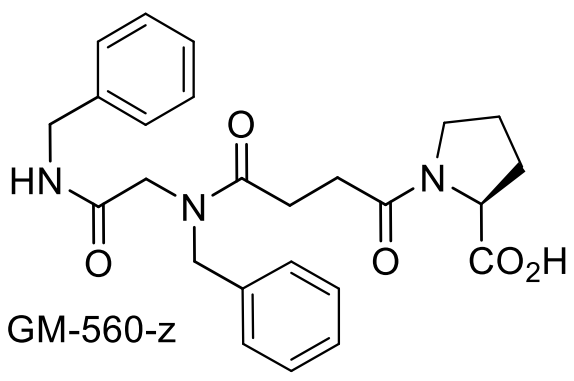

GM-560-z

Obtained as a white solid, yield 46% (0.420 g). ^{1}H NMR (400 MHz, DMSO) δ 12.51 (s, 1H, CO_2H), [8.55 (t, *J* = 5.9 Hz, 1H), 8.26 (t, *J* = 7.0 Hz), 8.27 (t, *J* = 6.9 Hz), 1H, NH], 7.39-7.20 (m, 10H), 4.67-3.82 (m, 7H), 3.63-3.23 (m, 4H), 3.73-3.46 (m, 4H), [2.31-2.01 (m), 1.95-1.68 (m), 4H]. Conformers detected. ^{13}C NMR (101 MHz, DMSO) δ 173.84, 173.76, 173.50, 173.44, 172.49, 172.46, 170.48, 170.35, 170.15, 170.01, 168.14, 168.09, 139.40, 139.34, 139.09, 137.59, 137.32, 137.29, 128.66, 128.33, 128.21, 127.62, 127.28, 127.26, 127.05, 127.02, 126.95, 126.86, 126.83, 126.66, 58.76, 58.34, 58.28, 51.71, 49.75, 49.34, 48.91, 46.40, 42.27, 41.92, 30.84, 29.33, 29.24, 28.87, 28.83, 27.18, 27.05, 24.30, 24.24, 22.26. Conformers detected. LC-MS (ESI, m/z) calcd for $C_{25}H_{30}N_3O_5^+$ [M+H]$^+$: 452.2180, found: 452.3.

Compound 61 (4-((2-(benzylamino)-2-oxoethyl)(furan-2-ylmethyl)amino)-4-oxobutanoyl)-L-alanine

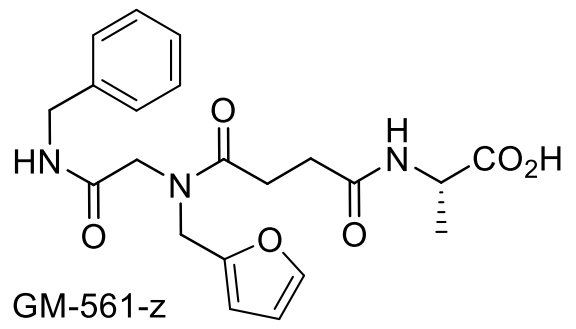

GM-561-z

Obtained as a yellow solid, yield 21% (0.248 g). ^{1}H NMR (400 MHz, DMSO) δ 12.48 (s, 1H, CO_2H), [8.55 (t, *J* = 5.9 Hz), 8.31 (t, *J* = 6.0 Hz), 1H, NH], [8.18 (d, *J* = 7.2 Hz), 8.16 (d, *J* = 7.2 Hz), 1H, NH], [7.63 (dd, *J* = 1.8, 0.9 Hz), 7.55 (d, *J* = 2.2 Hz), 1H], 7.35-7.20 (m, 5H), [6.43 (dd, *J* = 3.2, 1.8 Hz), 6.38 (dd, *J* = 3.2, 1.8 Hz), 1H], [6.41 (d, *J* = 3.2 Hz), 6.28 (d, *J* = 3.2 Hz), 1H], [4.58 (s), 4.51 (d, *J* = 15.5 Hz), 4.44 (d, *J* = 15.5 Hz), 2H], 4.32-4.26 (m, 2H), [4.18 (p, *J* = 7.3 Hz), 4.16 (p, *J* = 7.2 Hz), 1H], [4.05 (d, *J* = 17.8 Hz), 3.99 (d, *J* = 17.7 Hz), 3.89 (s), 2H], [2.81-2.66 (m), 2.57-2.32 (m), 4H], [1.25 (d, *J* = 7.3 Hz), 1.24 (d, *J* = 7.3 Hz), 3H]. Conformers detected. ^{13}C NMR (101 MHz, DMSO) δ 174.26, 172.04, 172.00, 171.31, 171.18, 168.08, 167.96, 150.77, 150.51, 143.04, 142.58, 139.35, 139.08, 128.34, 128.24, 127.27, 127.24, 127.10, 126.85, 126.70, 110.53, 110.42, 108.43, 108.41, 49.31, 48.25, 47.46, 44.92, 42.29, 41.97, 30.24, 30.21, 27.80, 27.67, 17.28, 17.25. Conformers detected. LC-MS (ESI, m/z) calcd for $C_{21}H_{26}N_3O_6^+$ $[M+H]^+$: 416.1816, found: 416.3.

Compound 62 (4-((2-(benzylamino)-2-oxoethyl)(furan-2-ylmethyl)amino)-4-oxobutanoyl)-L-leucine

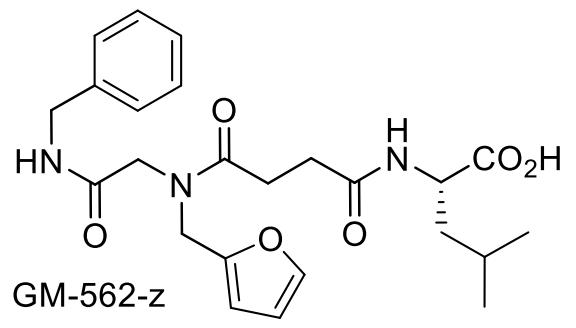

GM-562-z

Obtained as a yellow solid, yield 29% (0.322 g). ^{1}H NMR (400 MHz, DMSO) δ 12.47 (s, 1H, CO_2H), [8.55 (t, *J* = 5.9 Hz), 8.32 (t, *J* = 6.0 Hz), 1H, NH], [8.13 (d, *J* = 8.0 Hz), 8.10 (d, *J* = 8.0 Hz), 1H, NH], [7.62 (dd, *J* = 1.8, 0.9 Hz), 7.55 (dd, *J* = 1.9, 0.8 Hz), 1H], 7.34-7.21 (m, 5H), [6.43 (dd, *J* = 3.3, 1.8 Hz), 6.38 (dd, *J* = 3.2, 1.9 Hz), 1H], [6.41 (d, *J* = 3.4 Hz), 6.28 (d, *J* = 3.2 Hz), 1H], [4.57 (s), 4.51 (d, *J* = 15.5 Hz), 4.44 (d, *J* = 15.6 Hz), 2H], [4.30 (d, *J* = 6.0 Hz), 4.28 (d, *J* = 6.4 Hz), 2H], [4.22 (dt, *J* = 8.4, 5.8 Hz), 4.20 (td, *J* = 8.3, 4.3 Hz), 1H], [4.05 (d, *J* = 17.7 Hz), 4.00 (d, *J* = 17.7 Hz), 3.89 (s), 2H], [2.80-2.67 (m), 2.54-2.32 (m), 4H], 1.68-1.58 (m, 1H), 1.54-1.43 (m, 2H), [0.89 (d, *J* = 6.6 Hz), 0.88 (d, *J* = 6.6 Hz), 0.83 (d, *J* = 6.6 Hz), 0.83 (d, *J* = 6.6 Hz), 6H]. Conformers detected. ^{13}C NMR (101 MHz, DMSO) δ 174.22, 172.02, 171.96, 171.55, 171.42, 168.07, 167.96, 150.77, 150.51, 143.02, 142.56, 139.35, 139.07, 128.32, 128.23, 127.26, 127.09, 126.83, 126.69, 110.52, 110.42, 108.42, 108.37, 50.23, 50.20, 49.32, 48.23, 44.90, 42.28, 41.97, 30.25, 27.81, 24.30, 24.28, 22.84, 22.81, 21.41. Conformers detected. LC-MS (ESI, m/z) calcd for $C_{24}H_{32}N_3O_6^+$ $[M+H]^+$: 458.2286, found: 458.3.

Compound 63 (4-((2-(benzylamino)-2-oxoethyl)(furan-2-ylmethyl)amino)-4-oxobutanoyl)-L-phenylalanine

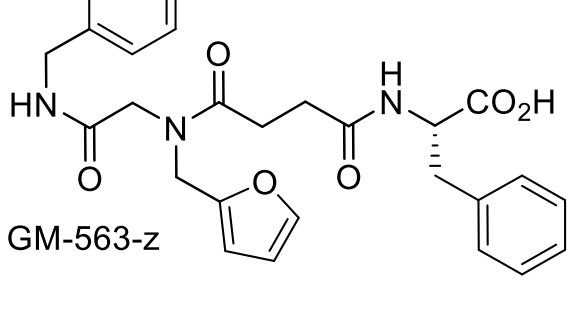

GM-563-z

Obtained as an off-white solid, yield 17% (0.201 g). ^{1}H NMR (400 MHz, DMSO) δ 12.71 (s, 1H, CO_2H), [8.54 (t, *J* = 5.9 Hz), 8.31 (t, *J* = 6.0 Hz), 1H, NH], [8.23 (d, *J* = 8.1 Hz), 8.20 (d, *J* = 8.2 Hz), 1H, NH], [7.62 (d, *J* = 1.8 Hz), 7.55 (d, *J* = 1.7 Hz), 1H], 7.34-7.16 (m, 10H), [6.42 (dd, *J* = 3.2, 1.8 Hz), 6.42 (dd, *J* = 3.1, 1.9 Hz), 1H], [6.39 (d, *J* = 3.0 Hz), 6.28 (d, *J* = 3.2 Hz), 1H], [4.53 (s), 4.49 (d, *J* = 15.7 Hz), 4.44 (d, *J* = 15.7 Hz), 2H], 4.44-4.38 (m, 1H), [4.30 (d, *J* = 5.8 Hz), 4.27 (d, *J* = 6.0 Hz), 2H], [3.99 (s), 3.88 (s), 2H], [3.03 (dd, *J* = 13.8, 5.1

Hz), 2.85 (dd, *J* = 13.8, 9.1 Hz), 2.84 (dd, *J* = 13.8, 9.1 Hz), 2H], [2.65 (t, *J* = 7.3 Hz), 2.44-2.29 (m), 4H].Conformers detected. ^{13}C NMR (101 MHz, DMSO) δ 173.05, 171.95, 171.89, 171.46, 171.35, 168.06, 167.89, 150.73, 150.46, 143.03, 142.57, 139.35, 139.06, 137.68, 137.66, 129.13, 128.33, 128.23, 128.12, 127.27, 127.09, 126.84, 126.70, 126.36, 110.52, 110.42, 108.42, 53.49, 49.27, 48.22, 44.89, 42.28, 42.26, 41.96, 36.92, 36.89, 30.36, 30.33, 27.86, 27.81. Conformers detected. LC-MS (ESI, m/z) calcd for $C_{27}H_{30}N_3O_6^+$ [M+H]$^+$: 492.2129, found: 492.3.

Compound 64 (4-((2-(benzylamino)-2-oxoethyl)(furan-2-ylmethyl)amino)-4-oxobutanoyl)-L-proline

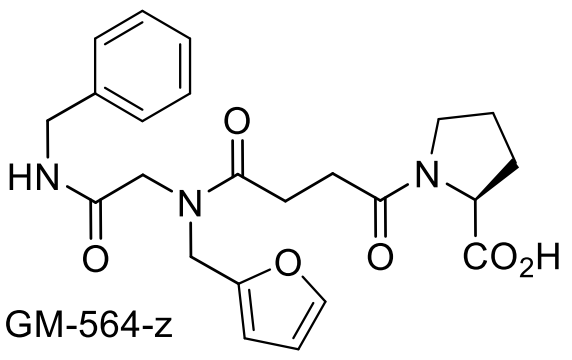

GM-564-z

Obtained as a yellow solid, yield 27% (0.291 g). ^{1}H NMR (400 MHz, DMSO) δ 12.51 (s, 1H, CO_2H), [8.55 (t, *J* = 5.9 Hz), 8.27 (t, *J* = 5.9 Hz), 8.25 (t, *J* = 6.5 Hz), 1H, NH], [7.62 (t, *J* = 1.4 Hz), 7.55-7.54 (m), 1H], 7.35-7.21 (m, 5H), [6.43 (d, *J* = 1.5 Hz), 6.42 (d, *J* = 1.5 Hz), 1H], [6.38 (dd, *J* = 3.2, 1.9 Hz), 6.29 (d, *J* = 3.2 Hz), 1H], 4.66-4.40 (m, 2H), 4.34-4.10 (m, 3H), 4.08-3.86 (m, 2H), [3.58-3.48 (m), 3.38-3.26 (m), 2H], [2.87-2.77 (m), 2.71-2.40 (m), 4H], [2.29-2.01 (m), 1.94-1.68 (m), 4H]. Conformers detected. ^{13}C NMR (101 MHz, DMSO) δ 173.85, 173.81, 173.51, 173.47, 172.19, 172.10, 170.44, 170.11, 169.93, 168.08, 167.96, 150.74, 150.56, 143.02, 142.58, 139.38, 139.32, 139.09, 128.33, 128.22, 127.30, 127.08, 127.05, 126.84, 126.68, 110.53, 110.43, 108.45, 108.41, 58.76, 58.34, 58.32, 49.41, 48.43, 46.40, 45.98, 45.01, 42.35, 42.29, 41.93, 30.87, 29.22, 29.14, 28.86, 28.84, 27.69, 27.29, 27.09, 24.30, 24.27, 22.23. Conformers detected. LC-MS (ESI, m/z) calcd for $C_{23}H_{28}N_3O_6^+$ [M+H]$^+$: 442.1973, found: 442.3.

Compound 65 N-(2-(cyclohexylamino)-2-oxoethyl)-2-hydroxy-N-propylacetamide

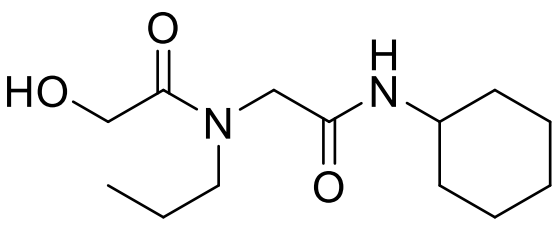

GM-566

Obtained as an off-white solid, yield 31% (0.159 g). Mixture of rotamers. ^{1}H NMR (400 MHz, DMSO) δ [6.24 (d, *J* = 8.6 Hz), 6.14 (d, *J* = 8.1 Hz), 5.68 (d, *J* = 8.5 Hz), 5.63 (d, *J* = 7.9 Hz), 1H, NH], [4.20 (d, *J* = 4.3 Hz), 4.18 (d, *J* = 4.5 Hz), 4.04 (d, *J* = 4.4 Hz), 4.01 (d, *J* = 4.3 Hz), 2H], [3.91 (s), 3.89 (s), 3.70 (s), 3.65 (s), 2H], 3.68-3.61 (m, 1H), [3.52 (t, *J* = 4.4 Hz), 3.42 (dt, *J* = 10.8, 4.5 Hz), 3.37-3.31 (m), 3.23-3.08 (m), 3.96-3.92 (m), 3H], 1.85-1.75 (m, 2H), 1.69-1.47 (m, 5H), 1.35-1.19 (m, 3H), 1.14-1.02 (m, 2H), [0.87 (t, *J* = 7.4 Hz), 0.84 (t, *J* = 7.4 Hz), 3H]. ^{13}C NMR (101 MHz, DMSO) δ 172.79, 172.70, 168.64, 167.71, 60.14, 59.94, 56.29, 51.41, 49.64, 48.37, 48.23, 46.96, 32.96, 32.91, 31.05, 25.75, 25.57, 24.95, 24.78, 21.53, 19.99, 11.20. LC-MS (ESI, m/z) calcd for $C_{13}H_{25}N_2O_3^+$ [M+H]$^+$: 257.1860, found: 257.2.

Compound 66 *N*-(2-(cyclohexylamino)-2-oxoethyl)-2-hydroxy-N-isobutylacetamide

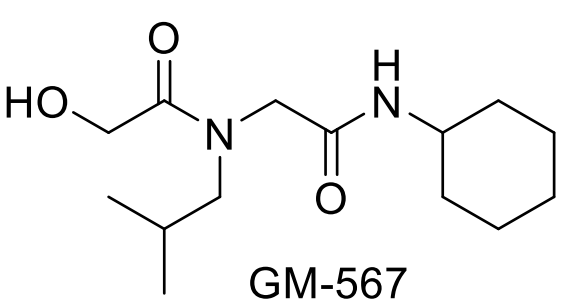

GM-567

Obtained as an off-white solid, yield 45% (0.243 g). Mixture of rotamers, only the major one is given. ^{1}H NMR (400 MHz, DMSO) δ [6.27 (d, *J* = 8.2 Hz), 5.61 (d, *J* = 8.2 Hz), 1H, NH], [4.17 (d, *J* = 4.5 Hz), 4.06 (d, *J* = 4.6 Hz), 2H], [3.94(s), 3.91 (s), 3.71 (s), 2H], 3.73-3.61 (m, 1H), [3.44 (t, *J* = 4.4 Hz), 2.99 (t, *J* = 6.4 Hz), 1H, OH], [3.23 (d, *J* = 7.5 Hz), 2.94 (d, *J* = 7.5 Hz), [2H], 2.00-1.89 (m, 1H), 1.84-1.75 (m, 2H), 1.69-1.47 (m,

3H), 1.35-1.20 (m, 2H), 1.17-1.00 (m, 3H), [0.84 (d, *J* = 6.5 Hz), 6H]. ^{13}C NMR (101 MHz, DMSO) δ 173.19, 167.68, 60.08, 55.55, 51.91, 48.35, 32.95, 27.12, 25.56, 24.78, 19.99. LC-MS (ESI, m/z) calcd for $C_{14}H_{27}N_2O_3^+$ [M+H]$^+$: 271.2016, found: 271.3.

Compound 67 *N*-(2-(cyclohexylamino)-2-oxoethyl)-2-hydroxy-N-pentylacetamide

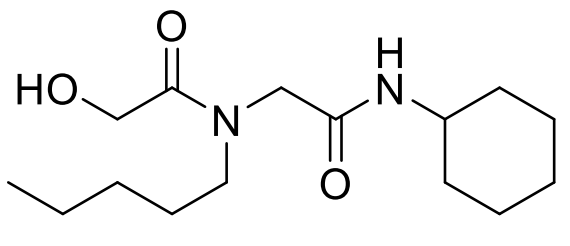

GM-568

Obtained as an off-white solid, yield 43% (0.245 g). Mixture of rotamers, only the major one is given. ^{1}H NMR (400 MHz, DMSO) δ [6.24 (d, *J* = 8.0 Hz), 6.13 (d, J = 8.2 Hz), 5.71 (d, *J* = 8.2 Hz), 5.66 (d, *J* = 8.2 Hz), 1H, NH], [4.20 (d, *J* = 3.9 Hz), 4.17 (d, *J* = 3.8 Hz), 4.04 (d, *J* = 3.5 Hz), 4.01 (d, *J* = 3.6 Hz), 2H], [3.91 (s), 3.89 (s), 3.70 (s), 2H], 3.77-3.61 (m, 1H), [3.52 (t, *J* = 4.4 Hz), 3.41 (t, *J* = 4.5 Hz), 1H, OH], [3.36 (d, *J* = 7.8 Hz), 3.13 (d, *J* = 7.9 Hz), 2H], 1.85-1.73 (m, 2H), 1.67-1.57 (m, 2H), 1.56-1.45 (m, 3H), 1.34-1.14 (m, 6H), 1.13-1.01 (m, 3H), 0.83 (t, *J* = 7.1 Hz, 3H). ^{13}C NMR (101 MHz, DMSO) ^{13}C NMR (101 MHz, $CDCl_3$) δ 172.70, 167.70, 59.92, 51.36, 48.36, 48.07, 32.94, 28.90, 27.98, 25.57, 24.77, 22.42, 14.01. LC-MS (ESI, m/z) calcd for $C_{15}H_{29}N_2O_3^+$ [M+H]$^+$: 285.2173, found: 285.4.

Compound 68 N-benzyl-N-(2-(cyclohexylamino)-2-oxoethyl)-2-hydroxyacetamide

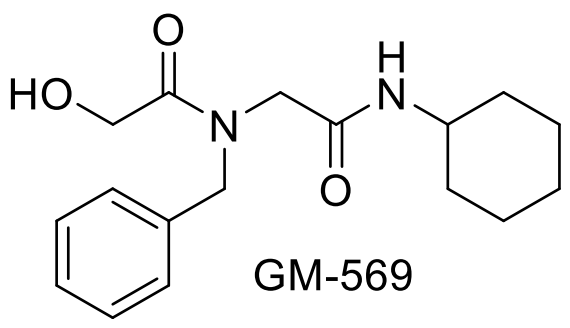

GM-569

Obtained as an off-white solid, yield 67% (0.407 g). Mixture of rotamers, only the major one is given. ^{1}H NMR (400 MHz, DMSO) δ 7.32-7.20 (m, 4H), [7.10 (d, *J* = 8.3 Hz), 7.10 (d, *J* = 7.8 Hz), 1H], [6.23 (d, *J* = 8.1 Hz), 5.82 (d, *J* = 8.2 Hz), 5.69 (d, *J* = 8.5 Hz), 5.34 (d, *J* = 8.2 Hz), 1H, NH], [4.62 (s), 4.43 (s), 2H], [4.25 (d, *J* = 4.1 Hz), 4.20 (d, *J* = 4.1 Hz), 4.10 (d, *J* = 4.0 Hz), 4.01 (d, *J* = 3.9 Hz), 2H], [3.90 (s), 3.89 (s), 3.65 (s), 2H], [3.70-3.55 (m), 3.20-3.11 (m), 1H], [3.51 (t, *J* = 4.4 Hz), 3.44 (t, *J* = 4.5 Hz), 3.41 (t, *J* = 4.5 Hz), 1H, OH], 1.83-1.75 (m, 1H), 1.71-1.48 (m, 4H), 1.33-1.19 (m, 2H), 1.15-1.00 (m, 2H), 0.91-0.81 (m, 1H). ^{13}C NMR (101 MHz, DMSO) δ 173.16, 167.00, 134.80, 129.27, 128.40, 127.07, 60.16, 50.80, 50.16, 48.51, 33.01, 25.56, 24.83. LC-MS (ESI, m/z) calcd for $C_{17}H_{25}N_2O_3^+$ [M+H]$^+$: 305.1860, found: 305.1.

Compound 69 N-(2-(cyclohexylamino)-2-oxoethyl)-N-(furan-2-ylmethyl)-2-hydroxyacetamide

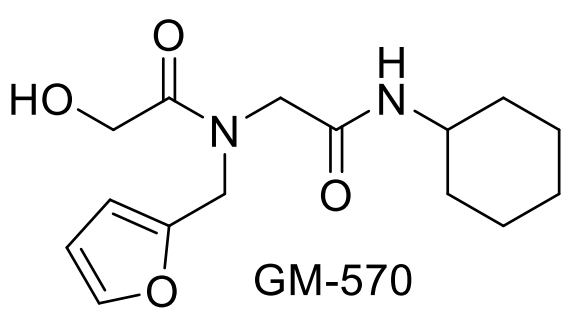

GM-570

Obtained as an off-white solid, yield 45% (0.265 g). Mixture of rotamers, only the major one is given. ^{1}H NMR (400 MHz, DMSO) 7.31 (d, *J* = 1.8 Hz, 1H), 6.30-6.23 (m, 2H), [5.84 (d, *J* = 8.1 Hz), 5.75 (d, *J* = 8.0 Hz), 5.59 (d, *J* = 8.5 Hz), 1H, NH], [4.62 (s), 4.34 (s), 2H], [4.38 (d, *J* = 4.0 Hz), 4.20 (d, *J* = 4.0 Hz), 4.07 (d, *J* = 4.0 Hz), 4.01 (d, *J* = 4.1 Hz), 2H], [3.93 (s), 3.89 (s), 3.75 (s), 2H], [3.68-3.58 (m), 3.21-3.13 (m), 1H], [3.53 (t, *J* = 4.4 Hz), 3.49 (t, *J* = 4.4 Hz), 3.40 (t, *J* = 4.3 Hz), 1H, OH], 1.83-1.71 (m, 2H), 1.66-1.56 (m, 2H), 1.55-1.44 (m, 1H), 1.33-1.17 (m, 2H), 1.15-0.91 (m, 3H). ^{13}C NMR (101 MHz, DMSO) δ 172.97, 167.03, 148.29, 143.38, 110.71, 109.84, 60.11, 50.12, 48.43, 44.07, 32.97, 25.54, 24.81. LC-MS (ESI, m/z) calcd for $C_{15}H_{23}N_2O_4^+$ [M+H]$^+$: 295.1652, found: 295.2.

Compound 70 N-(2-(tert-butylamino)-2-oxoethyl)-2-hydroxy-N-propylacetamide

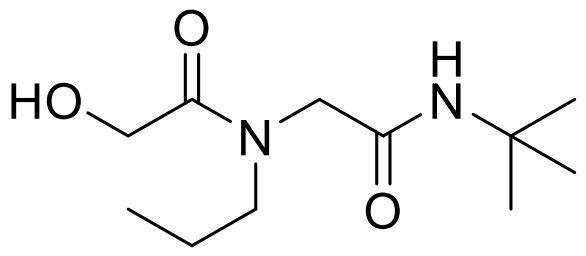

GM-571

Obtained as an off-white solid, yield 29% (0.133 g). Mixture of rotamers, only the major one is given. ^{1}H NMR (400 MHz, DMSO) δ [6.06 (s), 5.49 (s), 1H, NH], [4.18 (d, *J* = 3.5 Hz), 4.04 (d, *J* = 3.2 Hz), 2H], [3.85 (s), 3.63 (s), 2H], 3.48-3.39 (m, 1H, OH), [3.34 (t, *J* = 7.6 Hz), 3.11 (t, *J* = 7.5 Hz), 2H], 1.56 (m, *J* = 7.3 Hz, 2H), [1.29 (s), 1.26 (s), 9H], 0.85 (t, *J* = 7.4 Hz, 3H). ^{13}C NMR (101 MHz, DMSO) δ 172.65, 167.86, 59.91, 52.07, 51.45, 49.63, 27.70, 21.51, 11.21. LC-MS (ESI, m/z) calcd for $C_{11}H_{23}N_2O_3^+$ [M+H]$^+$: 231.1703, found: 231.2.

Compound 71 N-(2-(tert-butylamino)-2-oxoethyl)-2-hydroxy-N-isobutylacetamide

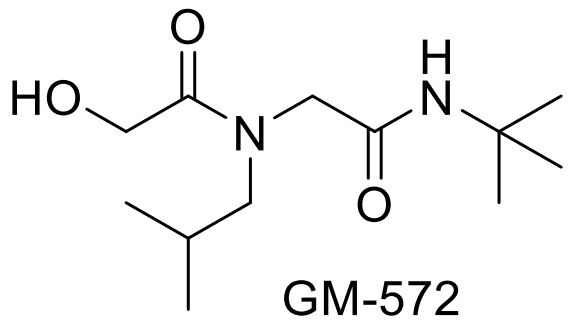

GM-572

Obtained as an off-white solid, yield 42% (0.204 g). Mixture of rotamers, only the major one is given. ^{1}H NMR (400 MHz, DMSO) δ [6.20 (s), 5.43 (s), 1H, NH], [4.17 (d, *J* = 4.4 Hz), 4.05 (d, *J* = 4.3 Hz), 2H], [3.84 (s), 3.64 (s), 2H], 3.45 (t, *J* = 4.4 Hz, 1H, OH), [3.22 (d, *J* = 7.4 Hz), 2.93 (d, *J* = 7.6 Hz), 2H], 2.01-1.83 (m, 1H), [1.29 (s), 1.26 (s), 9H], 0.85 (d, *J* = 6.6 Hz, 6H). ^{13}C NMR (101 MHz, DMSO) δ 173.06, 167.83, 60.07, 55.60, 52.72, 51.42, 28.73, 27.13, 20.01. LC-MS (ESI, m/z) calcd for $C_{12}H_{25}N_2O_3^+$ [M+H]$^+$: 245.1860, found: 245.3.

Compound 72 N-(2-(tert-butylamino)-2-oxoethyl)-2-hydroxy-N-pentylacetamide

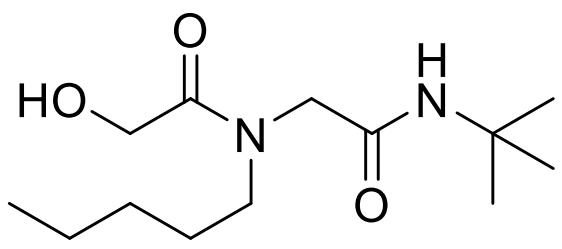

GM-573

Obtained as an off-white solid, yield 39% (0.201 g). Mixture of rotamers, only the major one is given. ^{1}H NMR (400 MHz, DMSO) δ [6.04 (s), 5.48 (s), 1H, NH], [4.17 (d, *J* = 4.4 Hz), 4.04 (d, *J* = 4.1 Hz), 2H], [3.84 (s), 3.62 (s), 2H], [3.45 (t, *J* = 5.4 Hz), 3.42 (t, *J* = 4.4 Hz), 1H, OH], [3.36 (t, *J* = 7.6 Hz), 3.14 (t, *J* = 7.8 Hz), 2H], 1.52 (p, *J* = 7.7 Hz, 2H), 1.31-1.15 (m, 4H), [1.29 (s), 1.26 (s), 9H], 0.83 (t, *J* = 7.2 Hz, 3H). ^{13}C NMR (101 MHz, DMSO) δ 172.57, 167.85, 59.91, 52.08, 51.45, 48.10, 28.92, 28.74, 28.00, 22.42, 14.01. LC-MS (ESI, m/z) calcd for $C_{13}H_{27}N_2O_3^+$ [M+H]$^+$: 259.2016, found: 259.2.

Compound 73 N-benzyl-N-(2-(tert-butylamino)-2-oxoethyl)-2-hydroxyacetamide

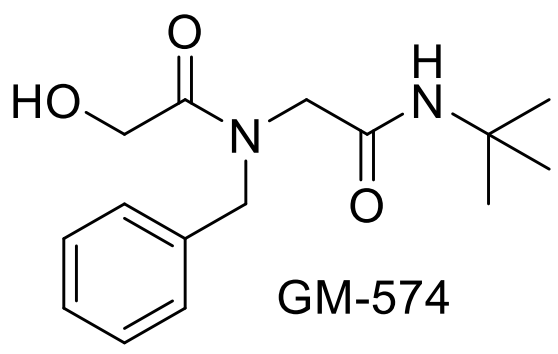

GM-574

Obtained as an off-white solid, yield 56% (0.311 g). Mixture of rotamers, only the major one is given. ^{1}H NMR (400 MHz, DMSO) δ 7.31-7.21 (m, 4H), 7.11-7.09 (m, 1H), [5.69 (s), 5.15 (s), 1H, NH], [4.61 (s), 4.43 (s), 2H], [4.26 (d, *J* = 4.3 Hz), 4.09 (d, *J* = 4.0 Hz), 2H], [3.84 (s), 3.60 (s), 2H], [3.45 (t, *J* = 4.5 Hz), 3.41 (t, *J* = 4.4 Hz), 1H, OH], [1.25 (s), 1.13 (s), 9H]. ^{13}C NMR (101 MHz, DMSO) δ 173.12, 173.05, 167.15, 166.00, 136.25, 134.90, 129.29, 129.27, 128.91, 128.43, 128.37, 127.11, 60.29, 60.15, 51.86, 51.66, 51.57, 50.82, 50.66, 50.14, 28.80, 28.56. LC-MS (ESI, m/z) calcd for $C_{15}H_{23}N_2O_3^+$ [M+H]$^+$: 279.1703, found: 279.3.

Compound 74 N-(2-(tert-butylamino)-2-oxoethyl)-N-(furan-2-ylmethyl)-2-hydroxyacetamide

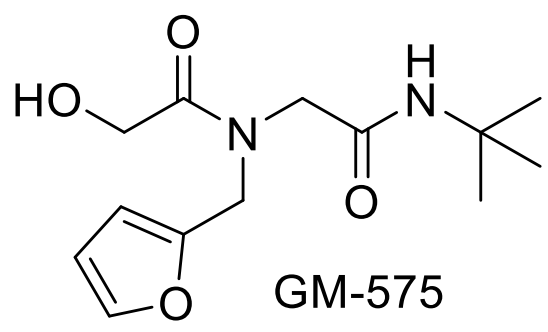

GM-575

Obtained as an off-white solid, yield 41% (0.219 g). Mixture of rotamers. ^{1}H NMR (400 MHz, DMSO) δ 7.33-7.31 (m, 1H), [6.30 (d, *J* = 2.0 Hz), 6.28 (d, *J* = 1.8 Hz) 6.27 (d, *J* = 2.0 Hz), 6.24 (d, *J* = 3.2 Hz), 1H], [5.69 (s), 5.40 (s), 2H], [4.62 (s), 4.34 (s), 2H], [4.38 (d, *J* = 4.1 Hz), 4.06 (d, *J* = 4.1 Hz), 2H], [3.87 (s), 3.68 (s), 2H], 3.39 (t, *J* = 4.5 Hz, 1H), [1.23 (s), 1.20 (s), 9H]. ^{13}C NMR (101 MHz, DMSO) δ 173.01, 172.84, 167.16, 166.06, 149.56, 148.39, 143.35, 143.00, 110.99, 110.71, 110.16, 109.79, 60.21, 60.09, 51.83, 51.54, 50.64, 50.24, 44.08, 43.93, 28.74, 28.60. LC-MS (ESI, m/z) calcd for $C_{13}H_{21}N_2O_4^+$ $[M+H]^+$: 269.1496, found: 269.1.

5. Biological Evaluation

The recombinant production of PfA-M1, TcLAP and LmLAP was reported by González-Bacerio et al.[2], Izquierdo et al.[3] and Aguado et al.[4].

Inhibition assays for recombinant metallo-aminopeptidases

The aminopeptidase enzymatic activity was assessed by a continuous kinetic method, using 100 μM Ala-AMC fluorogenic substrate for PfA-M1,[5] or 1 mM Leu-AMC substrate for TcLAP,[6] or 10 μM Leu-AMC substrate for LmLAP, solubilised in DMSO. These concentrations are approximately one value of apparent Michaelis-Menten's constant -appK_M- for each enzyme (unpublished results for LmLAP).[6,7] The fluorescence due to the release of AMC (excitation: 380 nm, emission: 460 nm) was monitored for 3 min using a microplate spectrofluorometer (CLARIOstarPlus, BMG Labtech, Germany). Kinetic assays were carried out with enzyme concentrations linearly related to the initial velocities, at 25ºC, in 96-well black plates (200 μL final volume). The activity buffer was 50 mM Tris-HCl pH 7.2 (for PfA-M1),[5] or pH 9.0, 4 mM $CoCl_2$ (for TcLAP)[3], or pH 7.0, 4 mM $MnCl_2$ (for LmLAP)[4]. The final concentration of DMSO was < 2 % (v/v). Only the linear portions of the progress curves, corresponding to substrate consumption below 5 %, were used to measure the reaction rates. Slopes with $R^2 < 0.98$ were not considered. All assays were performed in triplicate.

The volume of stock solution of peptoid-like compounds in DMSO used in the inhibition assays was 2 μL, reaching a concentration of 100 μM for the initial screening. The enzyme-compound pre-incubation was performed at 25 ºC for 30 min. To select the active compounds in the inhibition screenings at 100 μM, arbitrary maximal cut-offs of 50 % (for PfA-M1 and TcLAP), or 0 % (for LmLAP), residual activity was established. For the concentration-response studies, eight concentrations of each compound in the 0.78-100 μM range were prepared by serial dilutions in DMSO. Control assays were prepared by pre-incubating the enzyme with 2 μL DMSO. Residual activity was defined as the ratio between the enzymatic activity in the presence of the compound and the activity of the control. The IC_{50} values were calculated by nonlinear fitting of the logistic function to the experimental data, using OriginPro 8 SR0 software (version 8.0724 (B724); OriginLab Corporation [http://www.OriginLab.com]) with default parameters. The logistic function is: $y = 1 / (1 + [I] / IC_{50})$, where y: residual AP activity, and [I]: inhibitor concentration in the assay.[8] IC_{50} values are from independent replicates (n = 3)

Table S1. Evaluation of the inhibitory activity of a library of succinate-based peptide-peptoid hybrids against parasite MAPs

Nr.	Peptide-peptoid hybrid[a]	Code	Yield (%)[a]	Residual enzyme activity (%) at 100 μM of compound[b]		
				PfA-M1	TcLAP	LmLAP
**1**	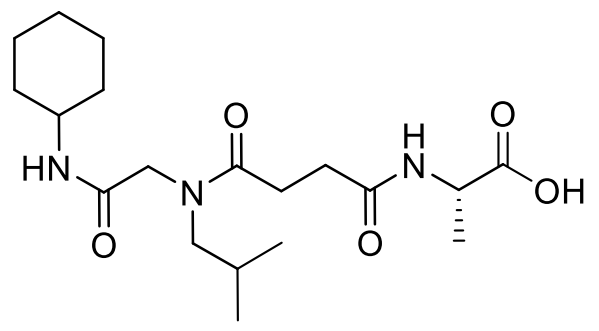	GM501z	29	56.2 ± 21.5	84.3 ± 11.6	131.5 ± 36.6
**2**	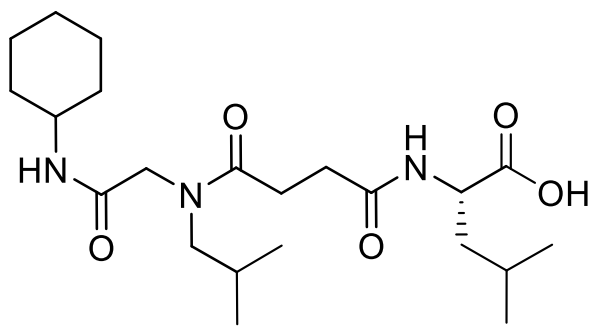	GM502z	34	95.9 ± 7.4	104.5 ± 11.2	106.8 ± 8.9
**3**	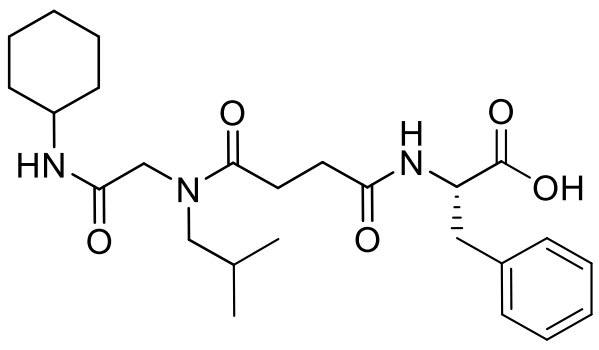	GM503z	18	197.3 ± 43.7	90.9 ± 26.0	111.2 ± 3.8
**4**	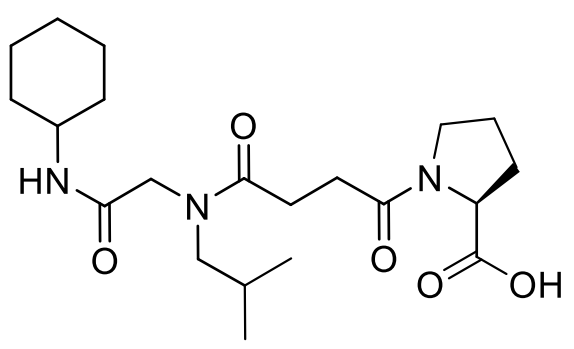	GM504z	39	106.3 ± 14.7	78.1 ± 2.1	121.0 ± 10.3
**5**	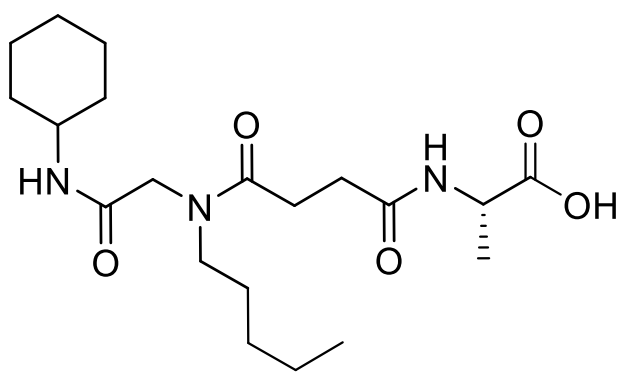	GM505z	28	90.9 ± 7.0	100.7 ± 16.0	82.6 ± 7.9
**6**	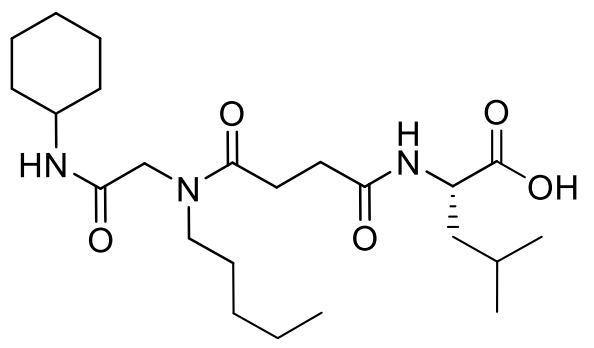	GM506z	33	122.2 ± 19.6	68.1 ± 2.6	110.1 ± 3.3

**7**	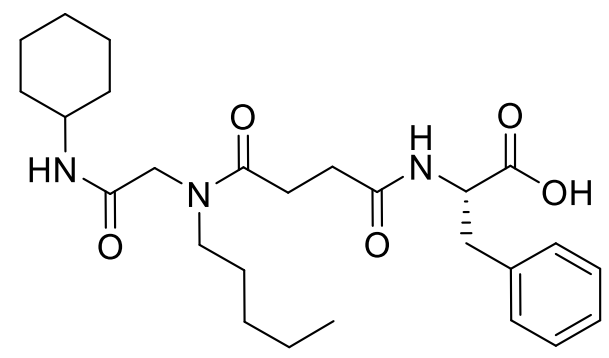	GM507z	27	82.7 ± 19.6	59.1 ± 20.0	109.2 ± 2.2
**8**	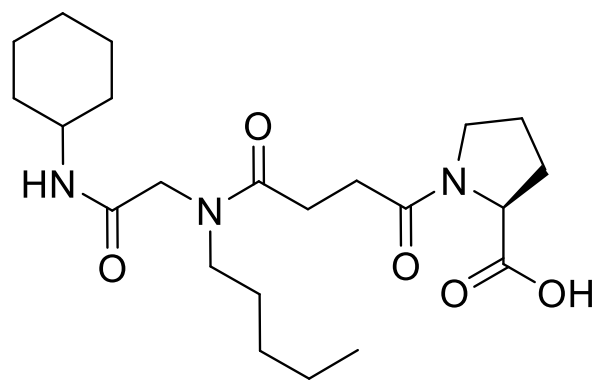	GM508z	38	101.1 ± 9.6	75.9 ± 27.4	114.8 ± 2.9
**9**	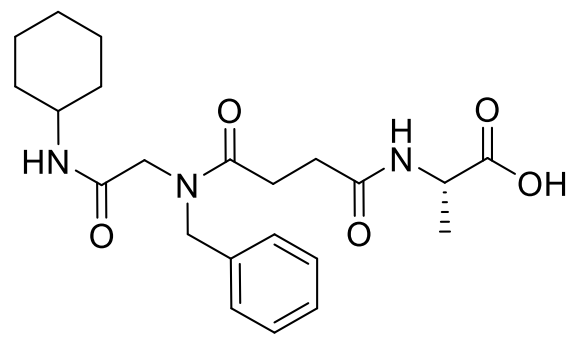	GM509z	15	76.5 ± 13.9	93.9 ± 9.6	107.2 ± 14.9
**10**	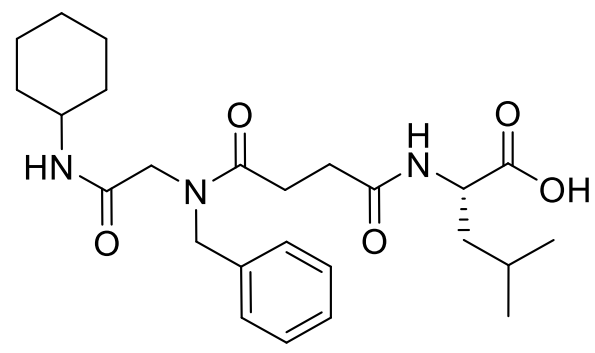	GM510z	24	153.7 ± 40.2	92.0 ± 3.8	109.5 ± 11.2
**11**	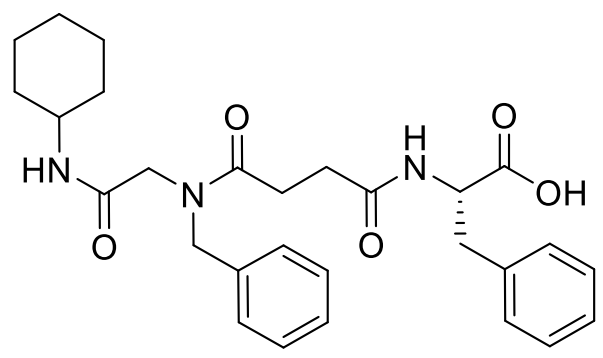	GM511z	13	99.6 ± 14.9	78.4 ± 5.7	108.1 ± 5.6
**12**	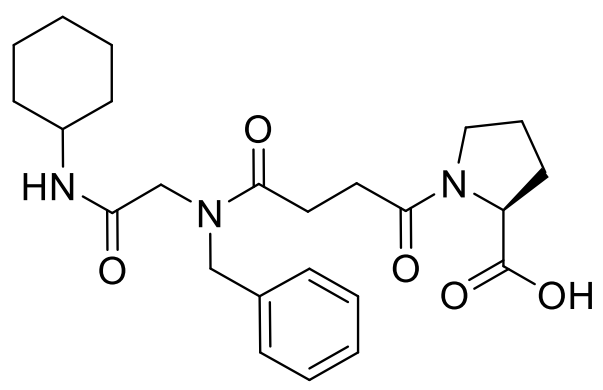	GM512z	25	84.4 ± 34.5	133.4 ± 12.5	98.6 ± 27.9
**13**	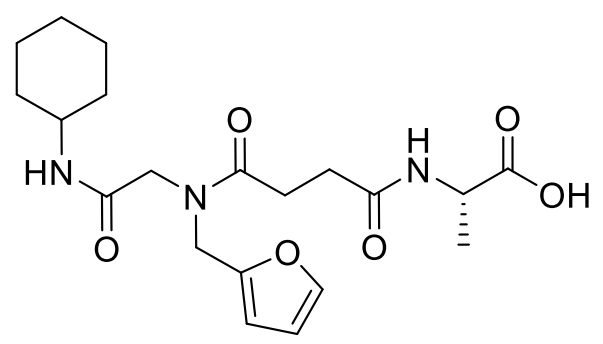	GM513z	17	34.9 ± 2.8	69.4 ± 12.9	96.7 ± 25.7
**14**	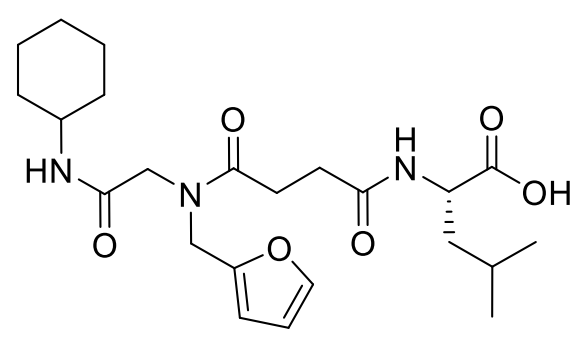	GM514z	14	177.4 ± 143.1	80.0 ± 13.0	120.9 ± 25.8

**15**	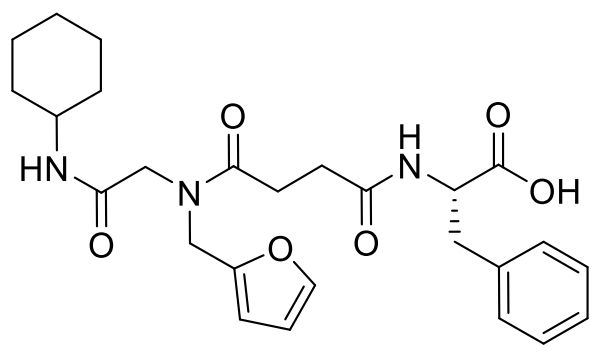	GM515z	25	154.5 ± 15.3	72.3 ± 8.9	101.7 ± 2.5
**16**	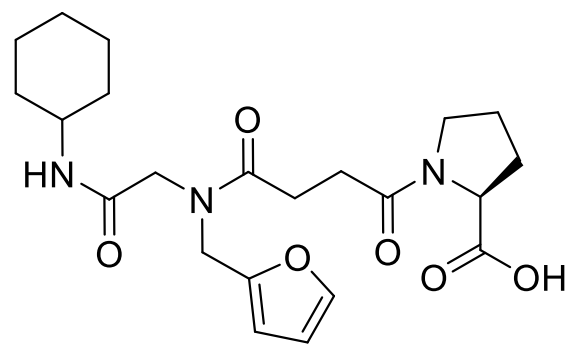	GM516z	35	147.3 ± 138.9	87.1 ± 9.5	65.4 ± 21.5
**17**	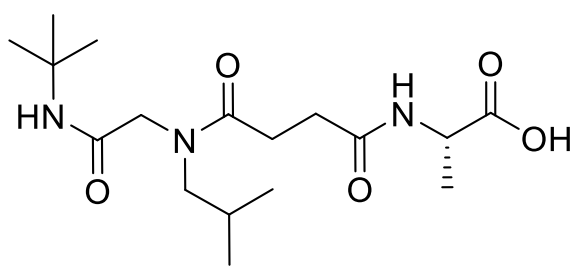	GM517z	24	72.4 ± 0.6	113.1 ± 13.2	108.1 ± 10.3
**18**	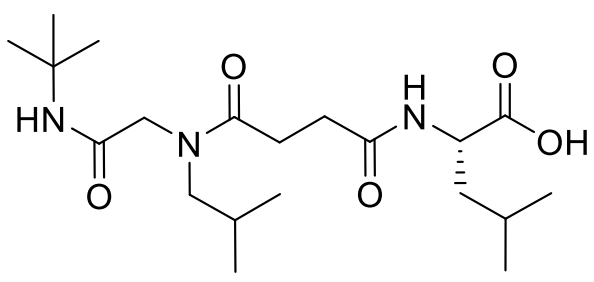	GM518z	40	49.8 ± 0.1	53.4 ± 7.5	91.6 ± 1.2
**19**	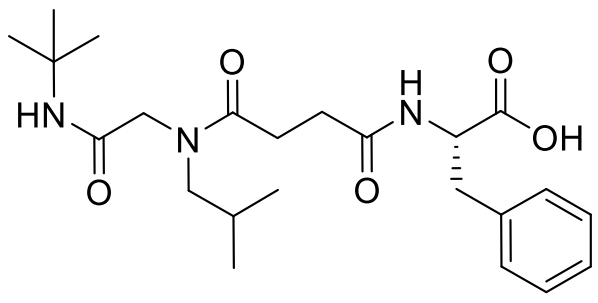	GM519z	42	49.9 ± 0.2	100.7 ± 9.8	109.3 ± 2.5
**20**	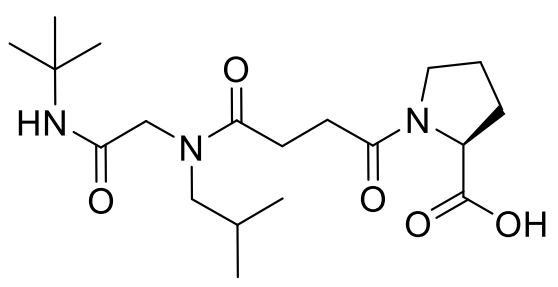	GM520z	33	69.5 ± 13.5	78.2 ± 7.7	102.3 ± 10.6
**21**	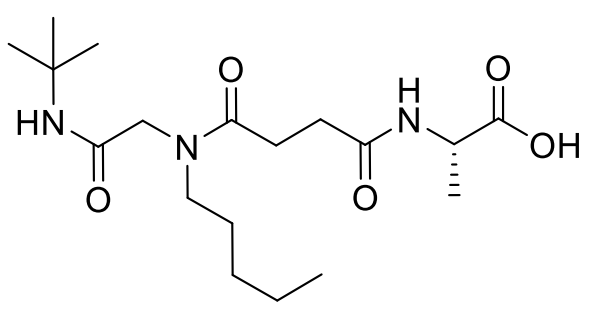	GM521z	31	102.5 ± 2.9	106.3 ± 9.5	105.9 ± 2.4
**22**	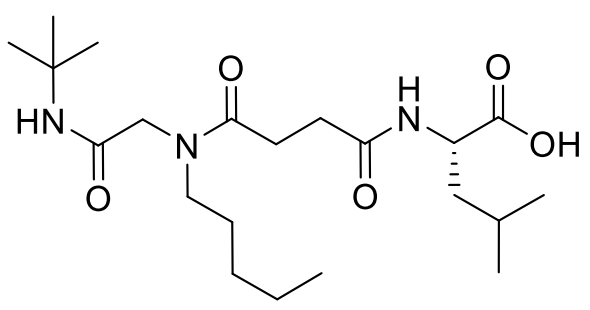	GM522z	34	125.6 ± 16.7	64.0 ± 5.4	103.8 ± 4.0
**23**	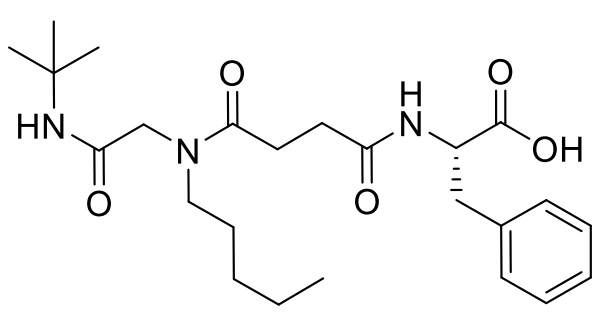	GM523z	37	97.7 ± 15.9	96.8 ± 10.2	94.6 ± 5.4

**24**	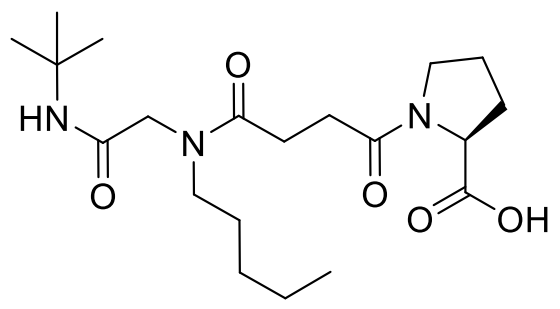	GM524z	26	71.9 ± 3.0	92.1 ± 8.5	75.8 ± 19.2
**25**	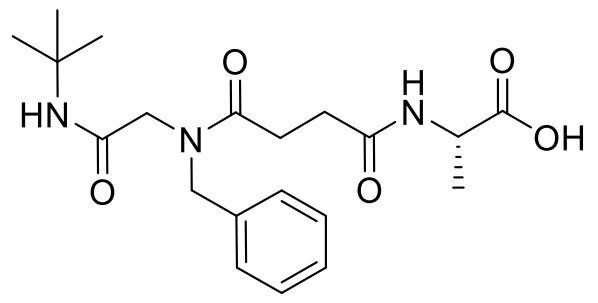	GM525z	29	48.9 ± 12.0	106.1 ± 9.6	103.7 ± 7.7
**26**	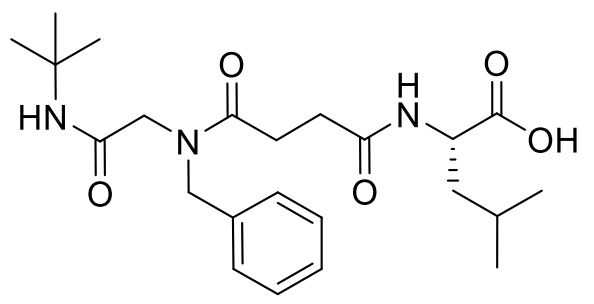	GM526z	37	90.7 ± 7.4	103.5 ± 10.2	87.4 ± 19.9
**27**	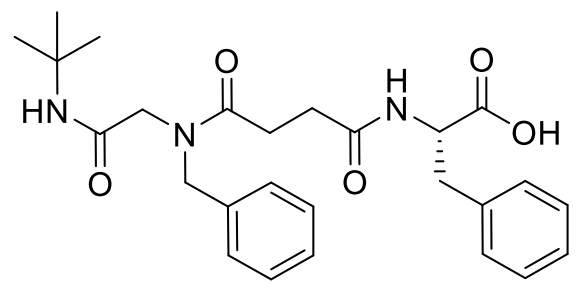	GM527z	36	55.2 ± 4.7	73.1 ± 8.8	112.4 ± 4.1
**28**	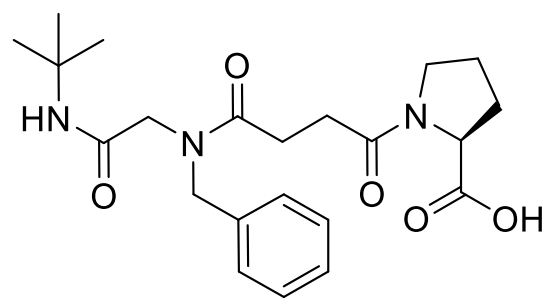	GM528z	31	92.1 ± 2.5	79.5 ± 7.6	120.0 ± 9.7
**29**	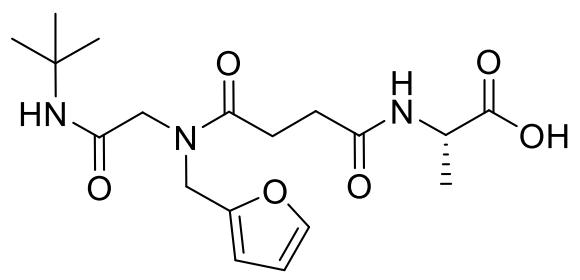	GM529z	30	117.1 ± 8.9	82.9 ± 9.7	114.9 ± 5.4
**30**	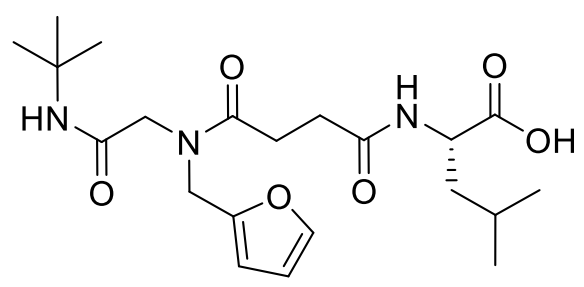	GM530z	36	108.1 ± 10.6	87.7 ± 9.9	130.5 ± 15.9
**31**	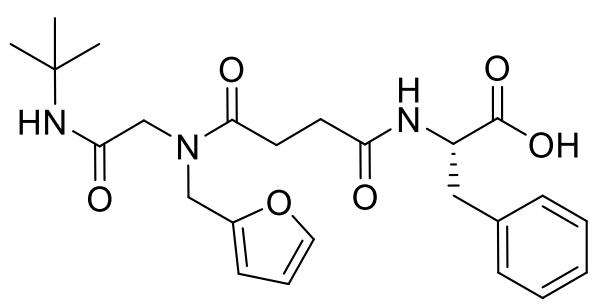	GM531z	37	92.9 ± 2.1	58.4 ± 4.3	100.4 ± 7.1
**32**	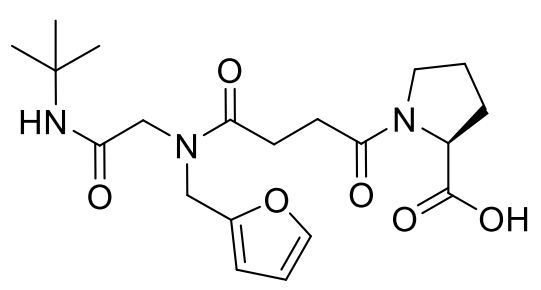	GM532z	30	92.1 ± 3.5	69.8 ± 6.9	151.1 ± 36.4
**33**	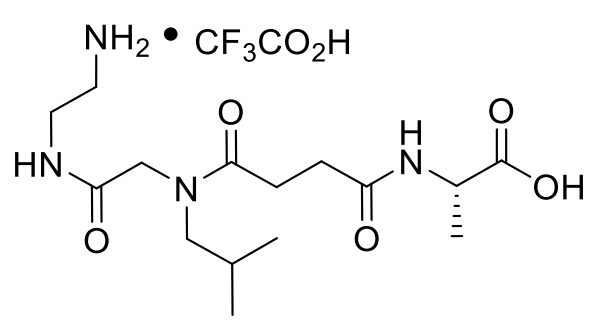	GM533z	33	200.0 ± 0.0	77.6 ± 6.7	66.1 ± 13.7

**34**	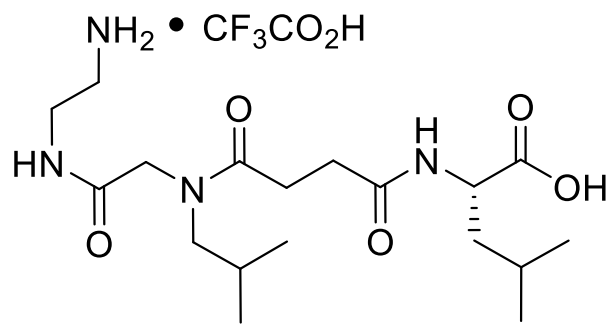	GM534z	22	200.0 ± 0.0	84.5 ± 8.8	65.2 ± 2.7
**35**	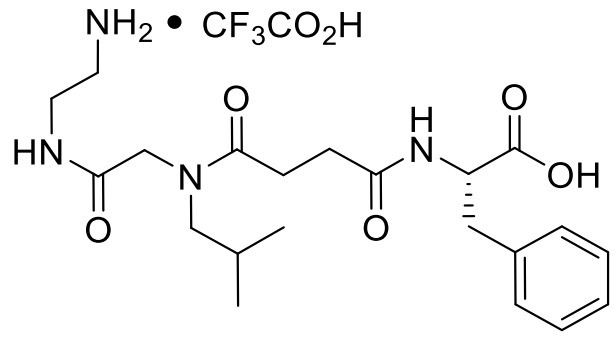	GM535z	29	200.0 ± 100.0	147.3 ± 16.3	56.9 ± 9.6
**36**	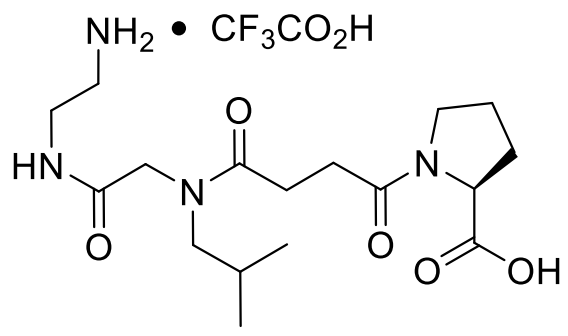	GM536z	37	86.9 ± 16.7	162.9 ± 15.4	21.7 ± 2.1
**37**	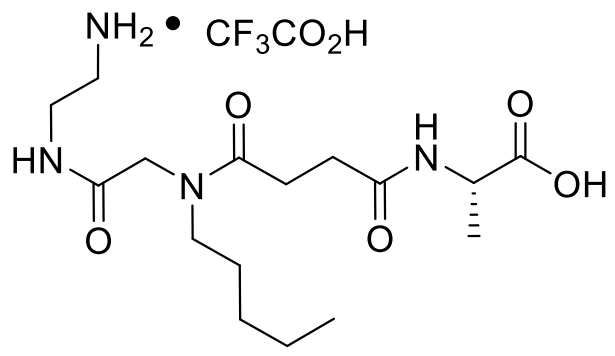	GM537z	44	166.7 ± 0.0	152.6 ± 15.3	114.1 ± 24.7
**38**	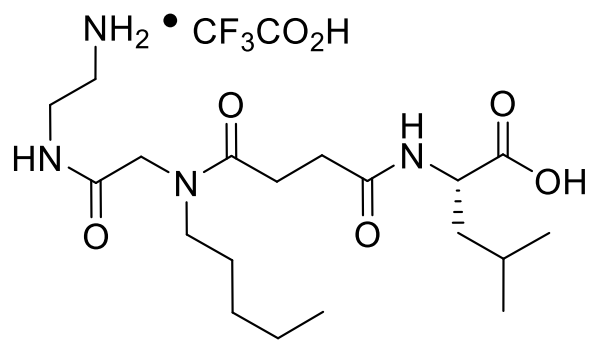	GM538z	45	166.7 ± 0.0	Not tested (availability)	Not tested (availability)
**39**	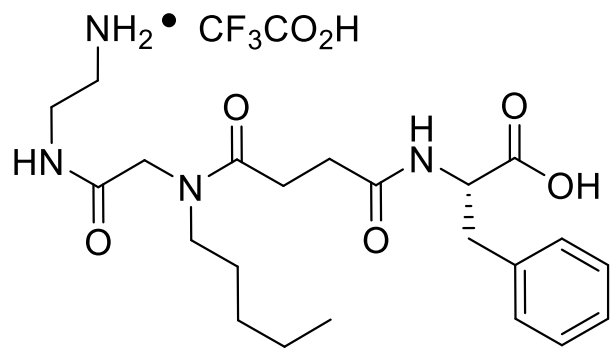	GM539z	32	166.7 ± 0.0	144.8 ± 13.2	44.9 ± 3.5
**40**	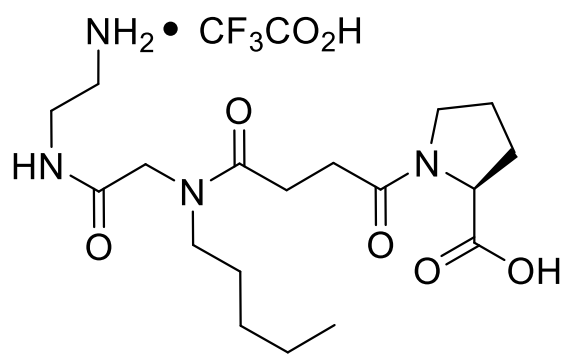	GM540z	48	166.7 ± 0.0	169.7 ± 17.5	75.3 ± 16.3
**41**	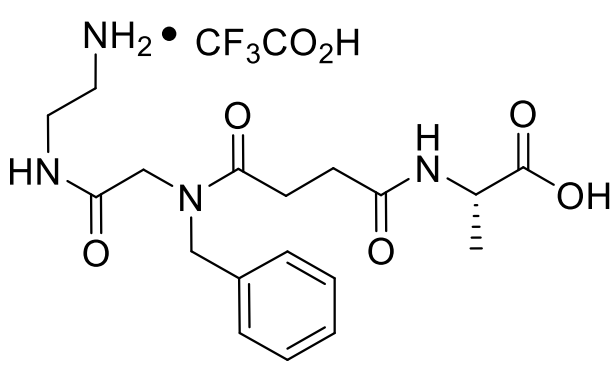	GM541z	27	43.9 ± 8.6	92.1 ± 20.0	20.8 ± 9.6

**42**	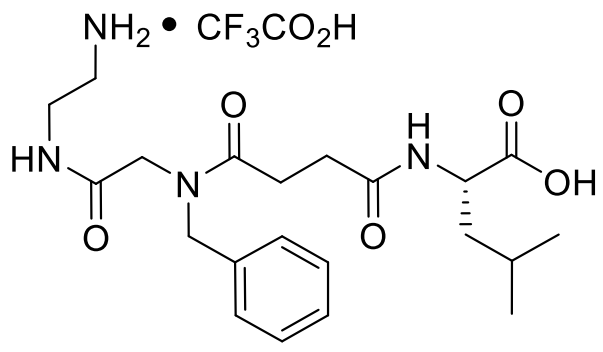	GM542z	33	100.0 ± 0.0	78.8 ± 6.7	114.7 ± 13.6
**43**	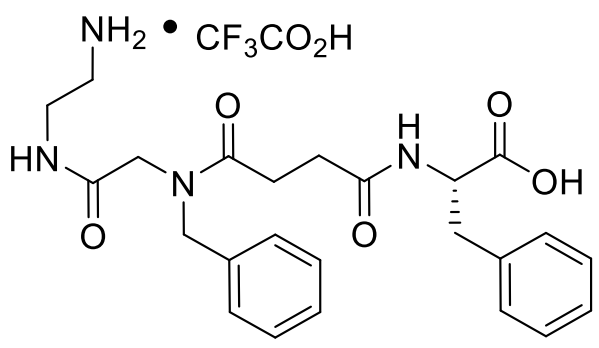	GM543z	36	48.4 ± 2.3	124.2 ± 11.9	15.7 ± 2.1
**44**	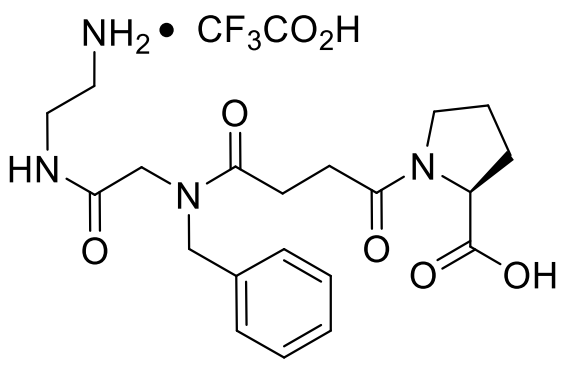	GM544z	32	100.0 ± 0.0	126.4 ± 13.2	124.4 ± 16.9
**45**	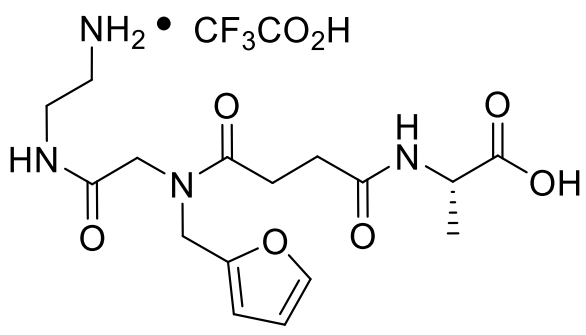	GM545z	32	100.0 ± 0.0	100.9 ± 9.7	34.3 ± 11.3
**46**	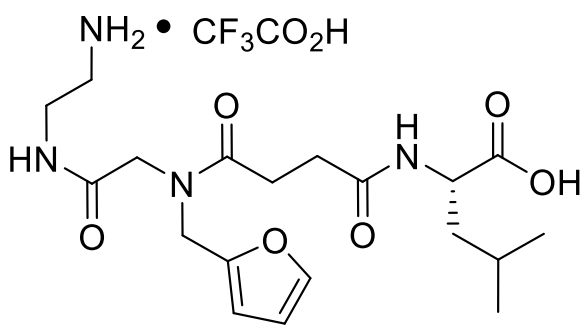	GM546z	30	100.0 ± 0.0	118.6 ± 13.2	0.0 ± 0.0
**47**	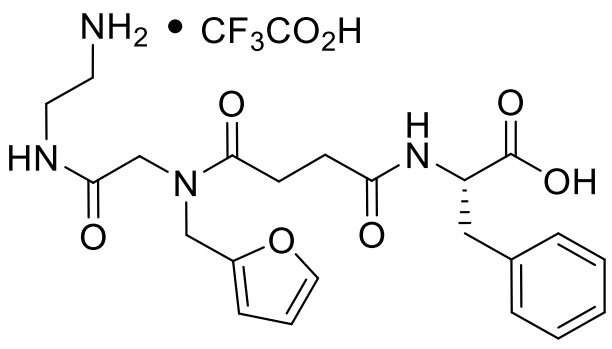	GM547z	22	100.0 ± 0.0	99.5 ± 8.7	58.3 ± 2.4
**48**	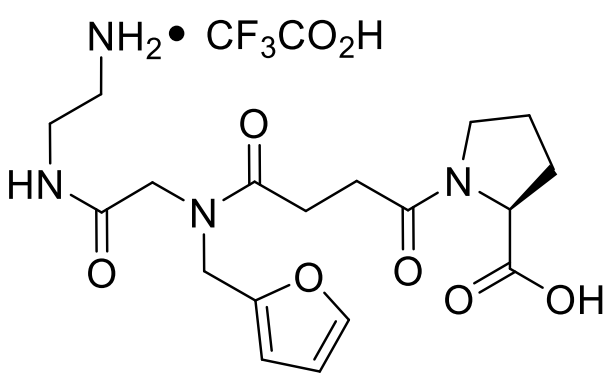	GM548z	38	50.0 ± 0.0	93.8 ± 7.6	115.7 ± 90.4
**49**	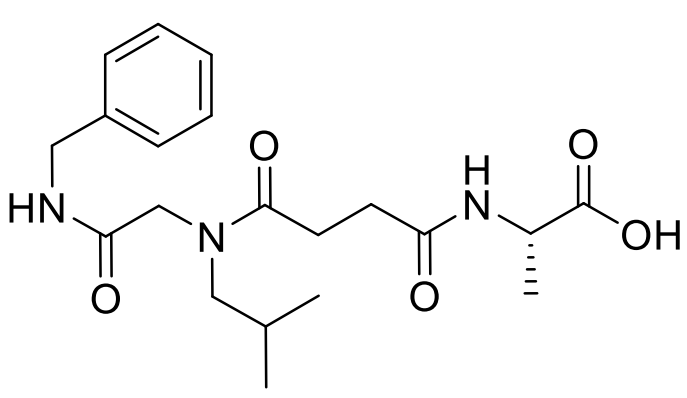	GM549z	30	123.1 ± 3.6	96.0 ± 9.5	87.7 ± 16.4

**50**	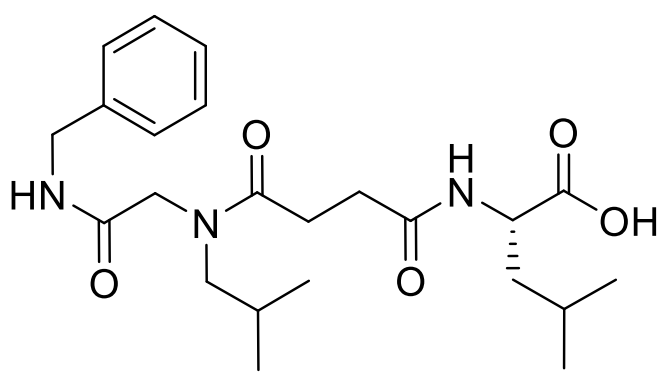	GM550z	32	113.4 ± 0.9	81.9 ± 8.7	7.9 ± 1.2
**51**	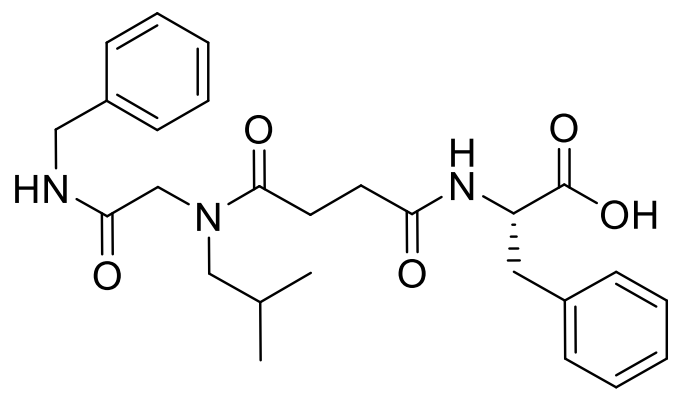	GM551z	42	99.9 ± 1.9	78.8 ± 6.5	10.8 ± 5.3
**52**	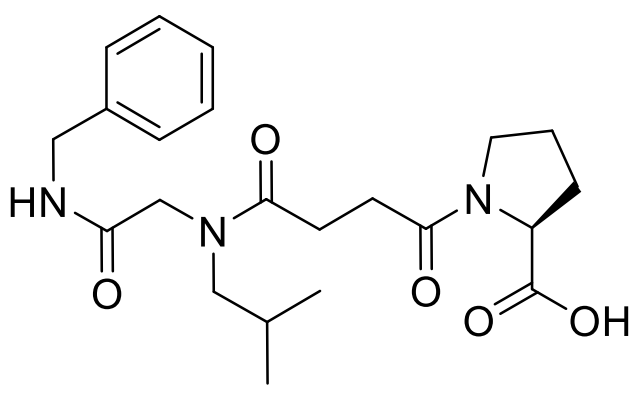	GM552z	48	123.3 ± 8.4	94.1 ± 9.5	94.8 ± 13.9
**53**	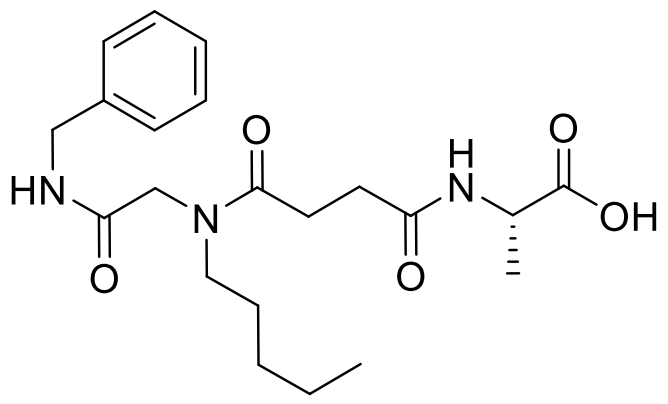	GM553z	34	78.2 ± 7.1	76.2 ± 9.3	162.2 ± 29.3
**54**	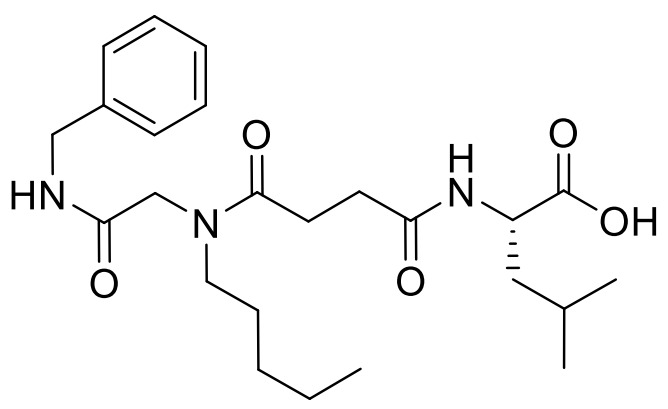	GM554z	14	67.9 ± 7.4	143.0 ± 78.0	185.3 ± 58.0
**55**	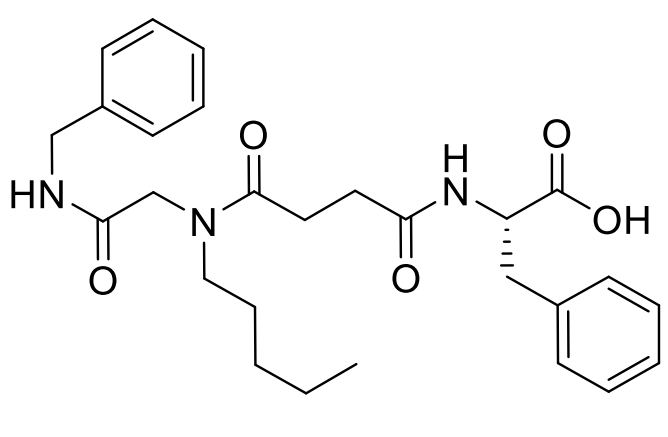	GM555z	21	74.6 ± 1.4	93.4 ± 8.4	132.5 ± 27.8
**56**	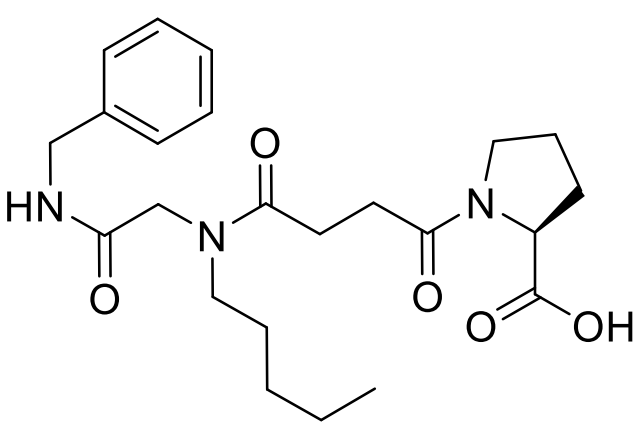	GM556z	35	86.8 ± 3.6	111.1 ± 12.6	78.7 ± 23.9

**57**	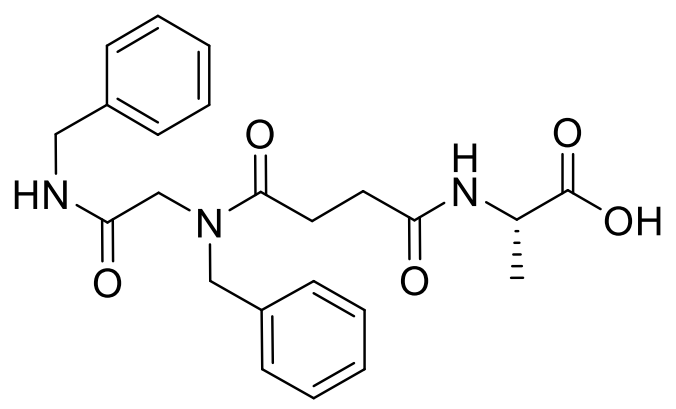	GM557z	40	107.7 ± 31.6	99.0 ± 55.0	117.3 ± 51.4
**58**	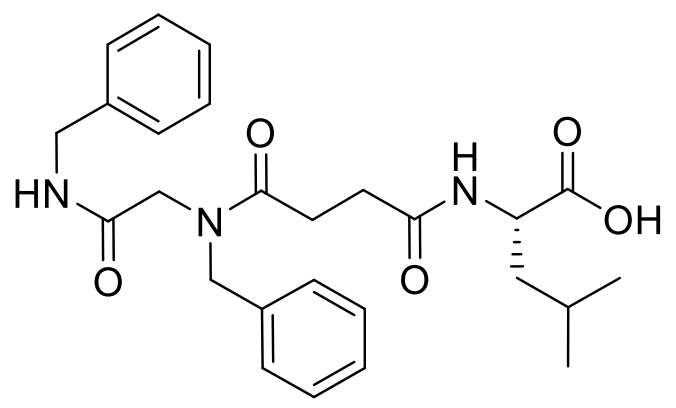	GM558z	12	90.0 ± 5.3	84.6 ± 9.6	89.4 ± 18.2
**59**	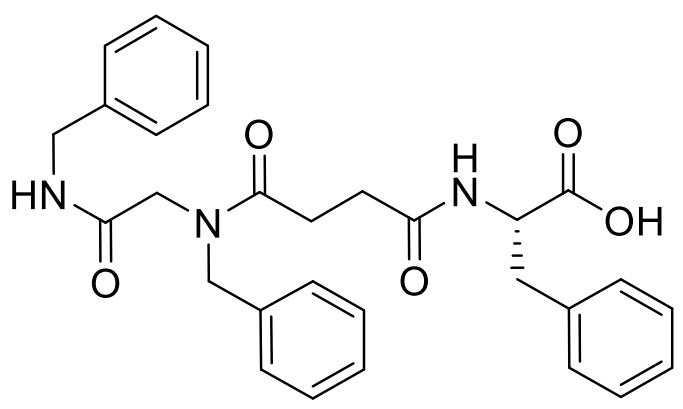	GM559z	27	23.3 ± 12.8	156.0 ± 124.0	93.8 ± 32.7
**60**	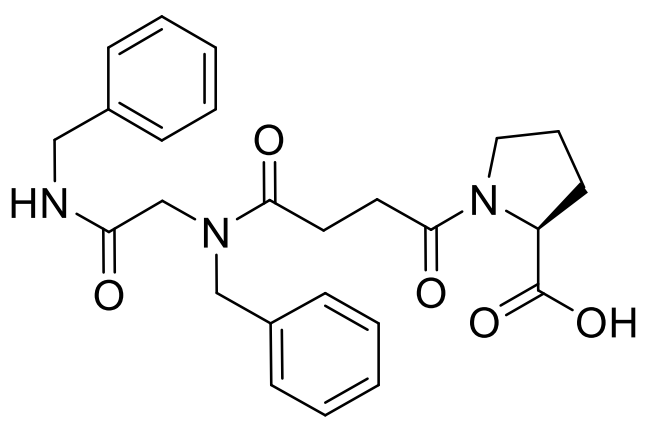	GM560z	46	45.4 ± 3.6	16.0 ± 1.6	114.6 ± 51.9
**61**	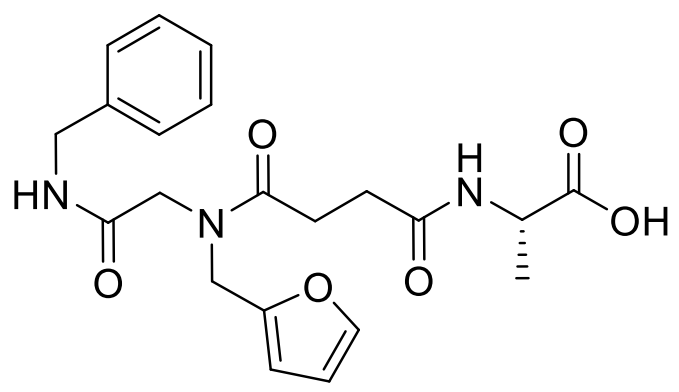	GM561z	21	64.2 ± 11.3	96.0 ± 9.3	229.4 ± 101.0
**62**	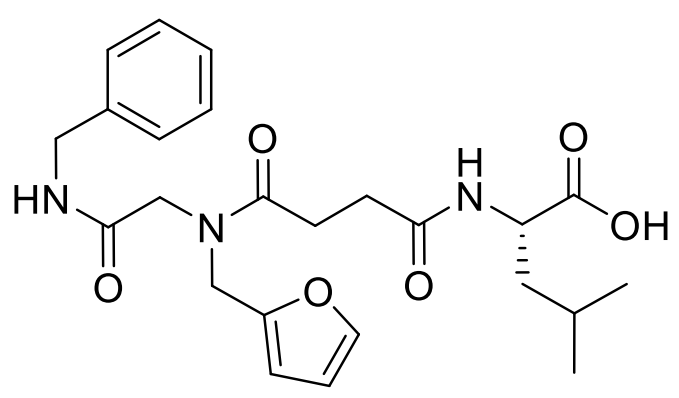	GM562z	29	91.2 ± 2.6	66.9 ± 7.4	167.3 ± 35.4
**63**	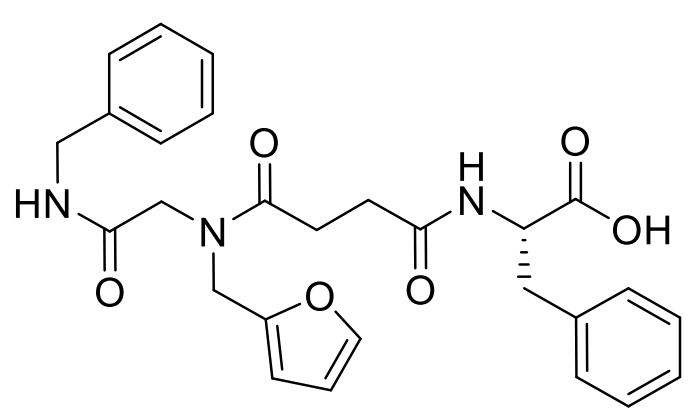	GM563z	17	60.3 ± 0.2	28.0 ± 18.0	265.4 ± 73.9

**64**	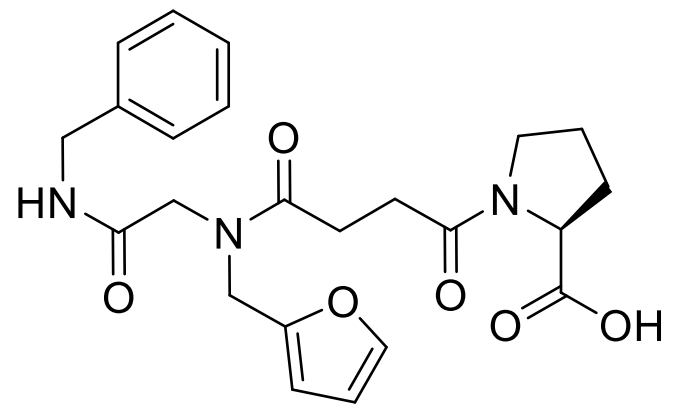	GM564z	27	67.1 ± 1.9	42.1 ± 4.5	116.6 ± 69.1

[a]Overall isolated yields after the four steps: 1) Ugi reaction, 2) methyl ester hydrolysis, c) coupling to the α-amino acid and d) final deprotection. [b]Inhibition assays for recombinant metallo-aminopeptidases (n = 3).

6. Molecular docking

Ligand preparation

The 3D coordinates of the compounds were obtained following a reported method.[9] AutoDock Tools v1.5.6[10] was used for ligand preparation. All hydrogen atoms were added to the molecules, Gasteiger charges were calculated, nonpolar hydrogens were removed, the atom type AD4 was assigned to each atom, and all rotatable torsion angles were defined as flexible. The final structures were exported in pdbqt format, which is a modified pdb file that includes the atomic charges, atom type definitions and topological information (for ligands). Bestatin was used as a positive control for simulations with PfA-M1.

Receptor preparation

The 3D structure of PfA-M1 protein was obtained from the RCSB PDB (https://www.rcsb.org) and used as receptor (PDB: 3EBH)[11]. The receptor coordinate file was modified to retain only the protein and the cofactor Zn^{2+}, in the active site, necessary for its function. On the other hand, there is only one TcLAP structure in the PDB database (PDB: 5NTG)[12]. One of the Mn^{2+} atoms (absent in the original structure) was manually added from the superimposition of structures of TcLAP and the acidic M17 LAP from *T. brucei* (PDB: 5NSM)[12]. The 3D structure of the LmLAP's monomer was obtained from the AlphaFold Protein Structure Databank, AF-Q7KF27-F1-v6 (https://alphafold.ebi.ac.uk/). Dimeric structure was obtained from ClusPro2.0 (https://cluspro.org/login.php). The two Zn^{2+} cofactors were manually added by superimpossing the dimeric model and the acidic M17 LAP from *L. major* (PDB ID: 5NTH)[12] as a template. Finally, all receptors were prepared using AutoDock Tools v1.5.6 software[10] and exported as pdbqt.

The docking simulations of the complexes were performed using AutoDock Vina v1.2.0.[13] The conformational search space was defined using a cubic box, of dimensions 26 Å × 26 Å × 26 Å and centered at coordinates x: 3.737, y: 13.265, z: 40.623 for TcLAP; and x: 11.618, y: 7.318, z: 1.922 for LmLAP; encompassing the inhibition sites described for these enzymes.[12] A grid box of 18 Å × 18 Å × 18 Å and centred at the coordinates x: 11.924, y: 10.930, z: 9.142, was used to cover the entire enzyme active site of PfA-M1.[11]

Molecular docking simulations were carried out by keeping the receptor residues rigid, while all rotatable angles of the ligand were considered flexible. Docking parameters were kept at default values, except the following: energy_range = 4 kcal, num_modes = 1, and

exhaustiveness = 32. For each ligand, a total of 20 models were generated. In each group, the mean and standard deviation of the free energy of binding, according to the AutoDock Vina scoring function, was reported. The conformation with the lowest free energy of binding was selected as the representative conformation. The 3D poses of the complexes were visualized using Pymol software v2.5.8. The 2D interactions of the selected models were obtained using LigPlot+ software v2.2.

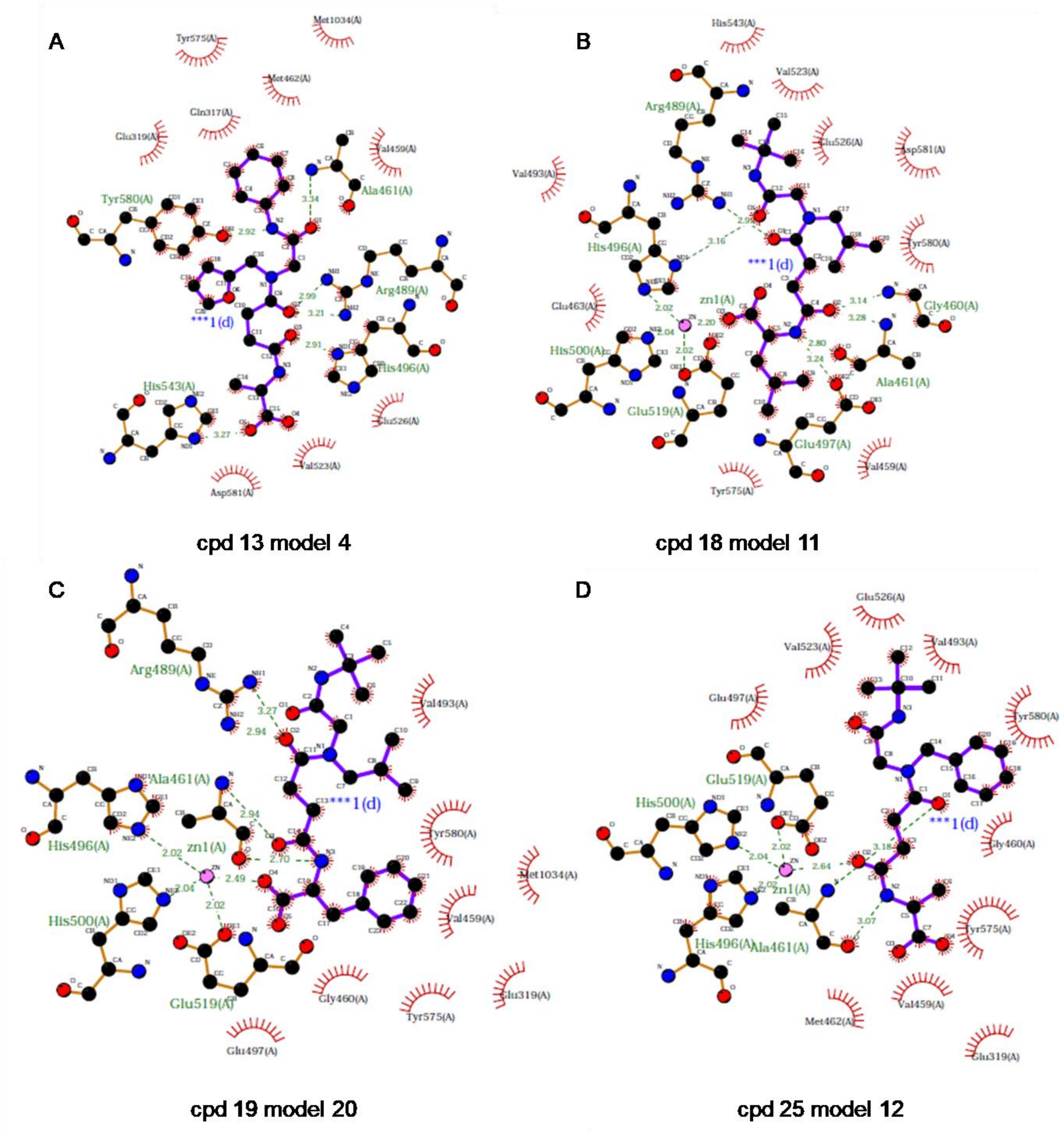

Figure S1. 2D representation of molecular docking results between compounds **13** (A), **18** (B), **19** (C) or **25** (D) and PfA-M1. The best models, in terms of binding affinities, after 20 runs for each inhibitor are shown. Inhibitors and residues that establish polar interactions or coordinate the Zn^{2+} cation (green discontinuous lines) are represented in the ball-stick model. Residues that establish hydrophobic interactions are represented as red arcs. Nitrogens are shown in blue, oxygens in red and carbons in black. The zinc ion is represented as a pink sphere.

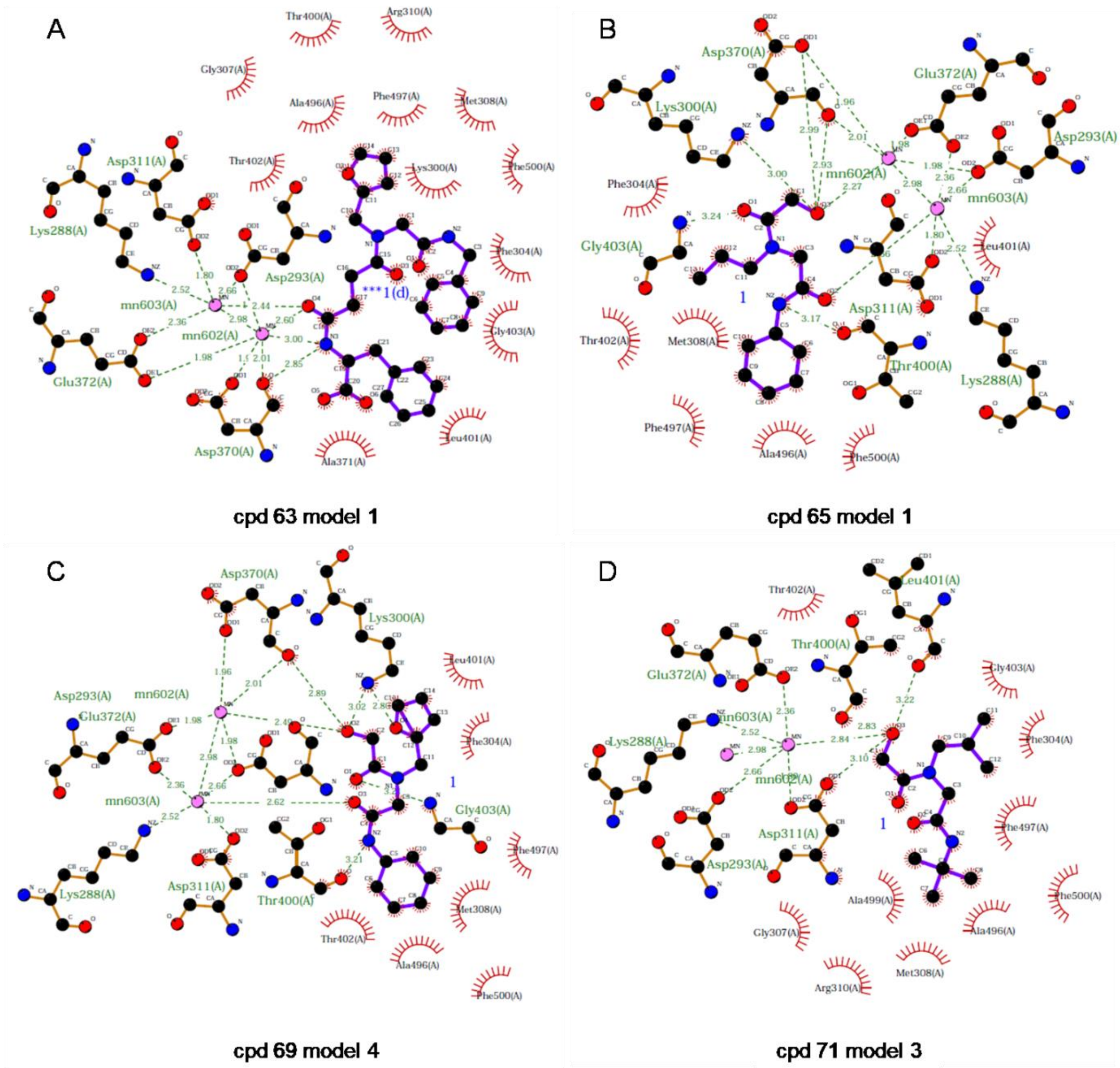

Figure S2. 2D representation of molecular docking results between compounds 63 (A), 65 (B), 69 (C) or 71 (D) and TcLAP. The best models, in terms of binding affinities, after 20 runs for each inhibitor are shown. Inhibitors and residues that establish polar interactions or coordinate the Mn^{2+} cations (green discontinuous lines) are represented in the ball-stick model. Residues that establish hydrophobic interactions are represented as red arcs. Nitrogens are shown in blue, oxygens in red and carbons in black. Manganese ions are represented as pink spheres.

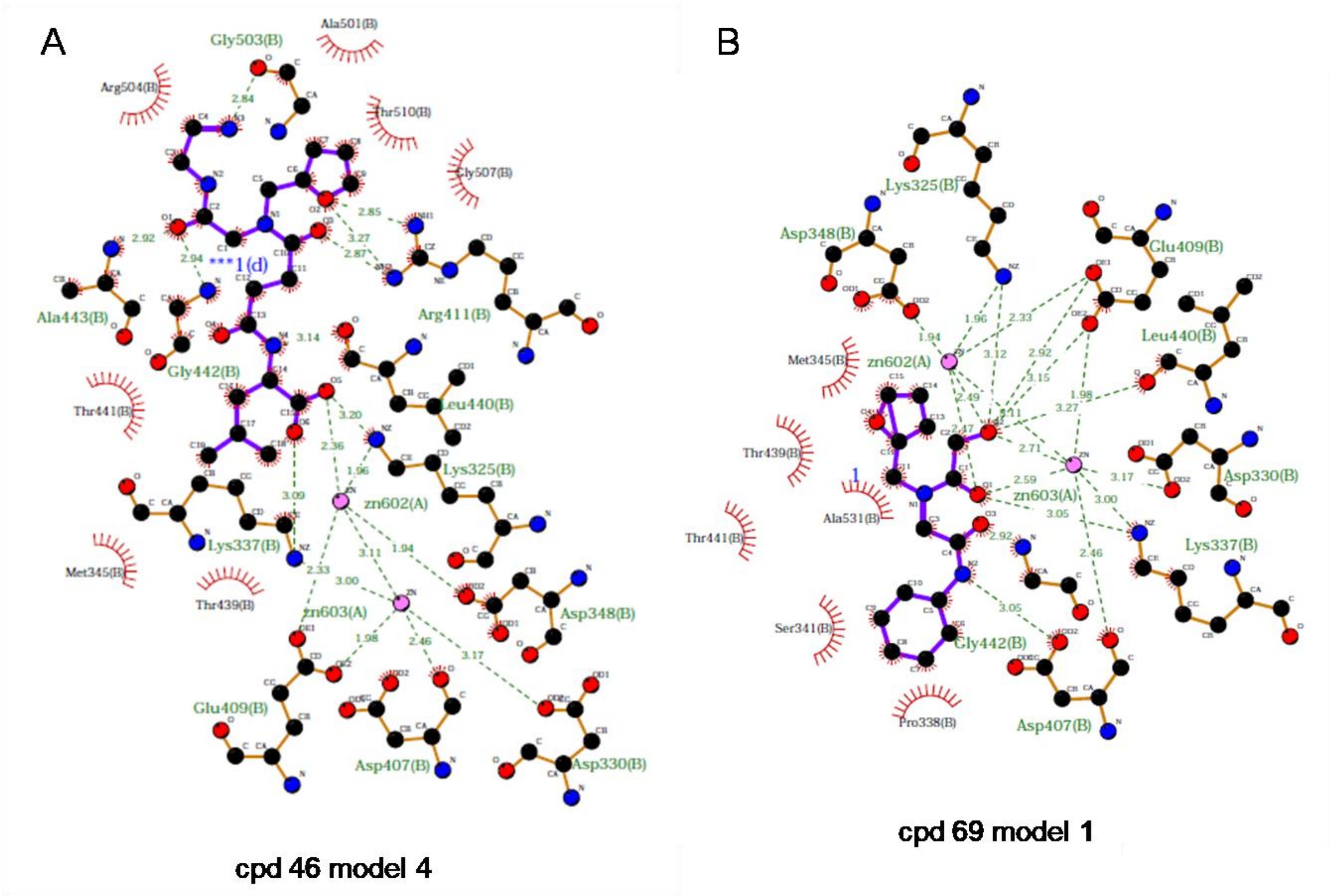

Figure S3. 2D representation of molecular docking between compounds 46 (A) or 69 (B) and LmLAP. One of the two best models, for 46, and the best model, for 69, in terms of binding affinities, after 20 runs for each inhibitor are shown. Inhibitors and residues that establish polar interactions or coordinate the Mn^{2+} cations (green discontinuous lines) are represented in the ball-stick model. Residues that establish hydrophobic interactions are represented as red arcs. Nitrogen atoms are shown in blue, oxygen atoms in red and carbon atoms in black. Manganese ions are represented as pink spheres.

7. NMR spectra of the final compounds

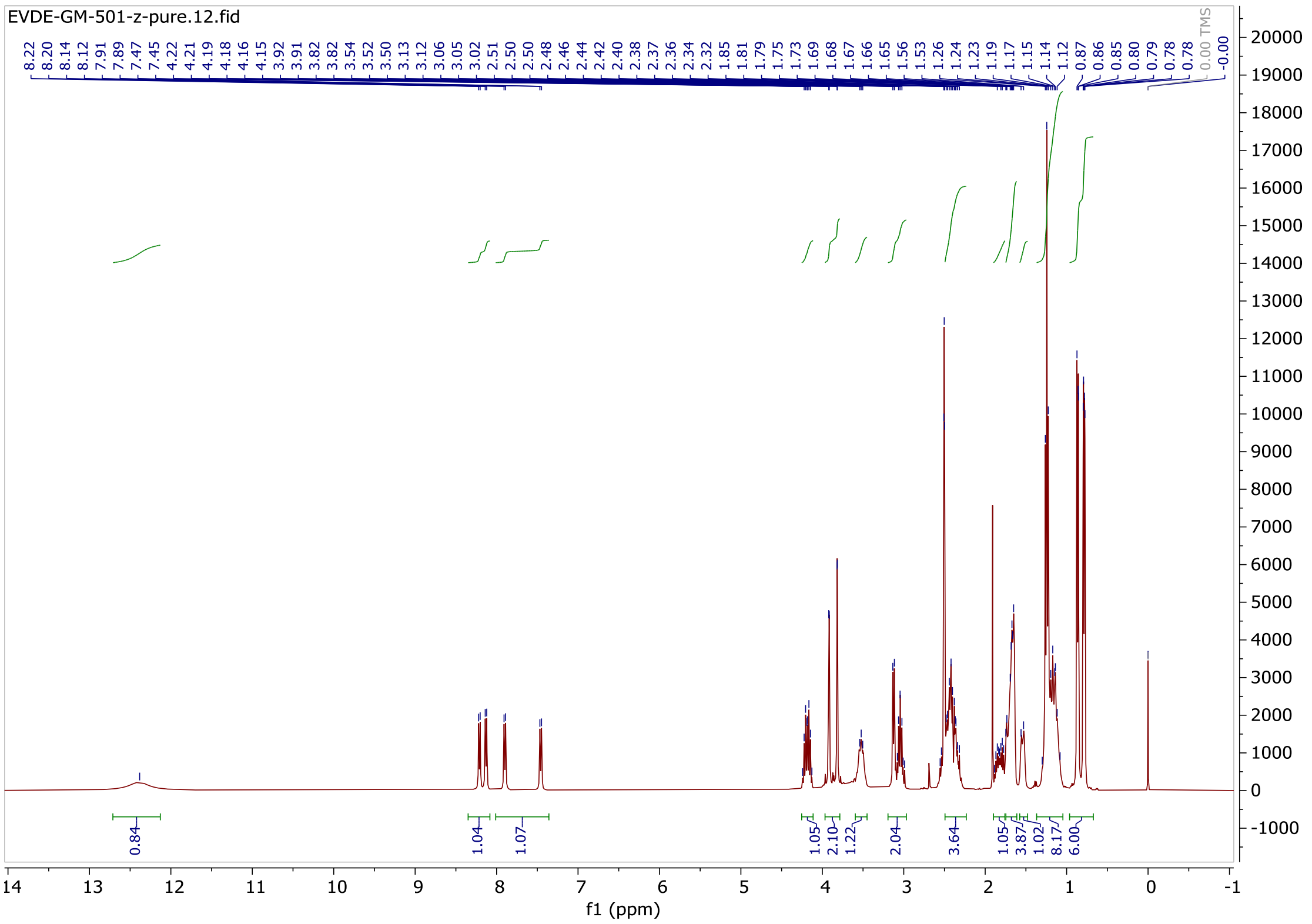

Figure S4. ^{1}H-NMR spectrum of compound **1**.

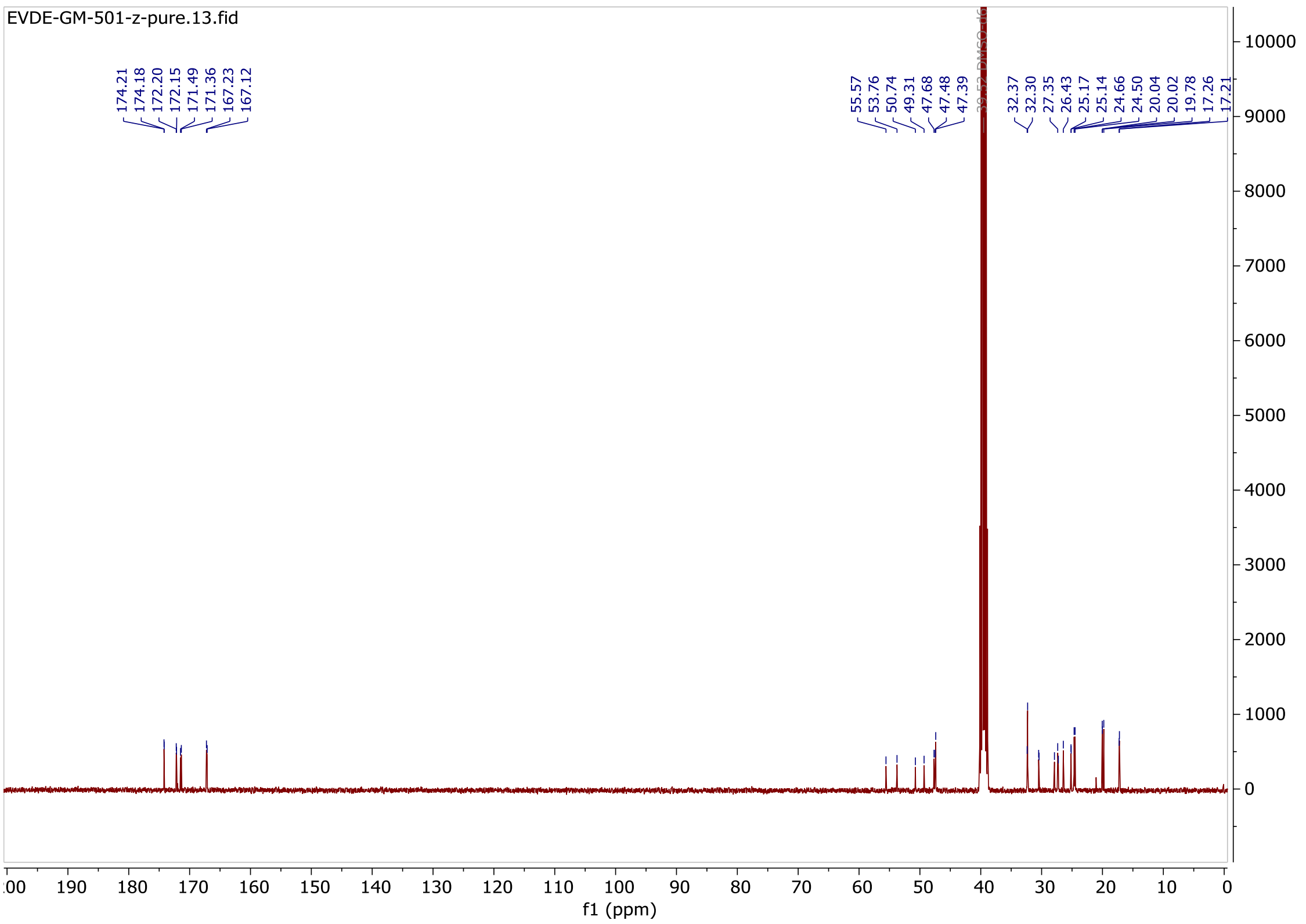

Figure S5. ^{13}C-NMR spectrum of compound **1**.

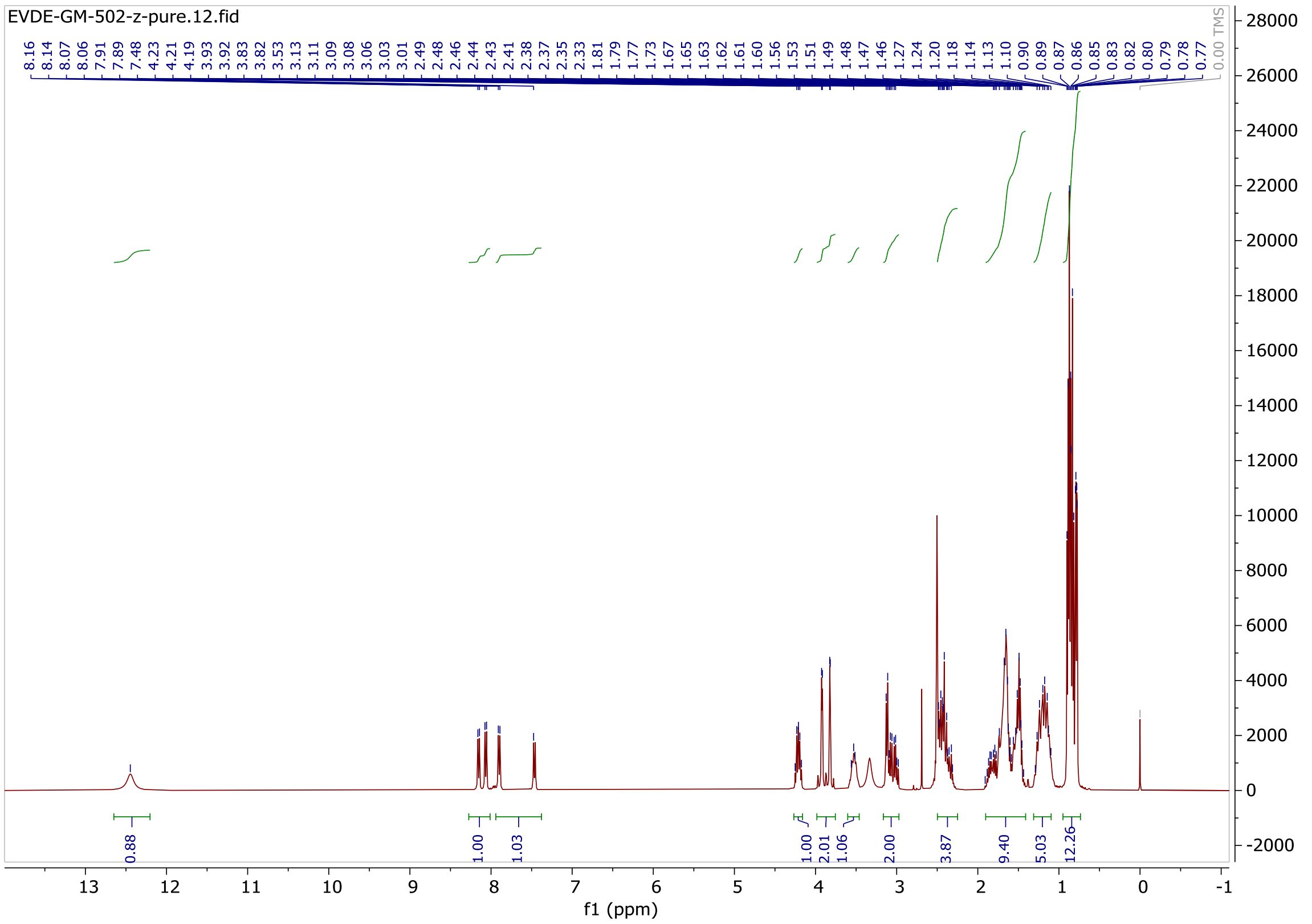

Figure S7. ^{1}H-NMR spectrum of compound **2**.

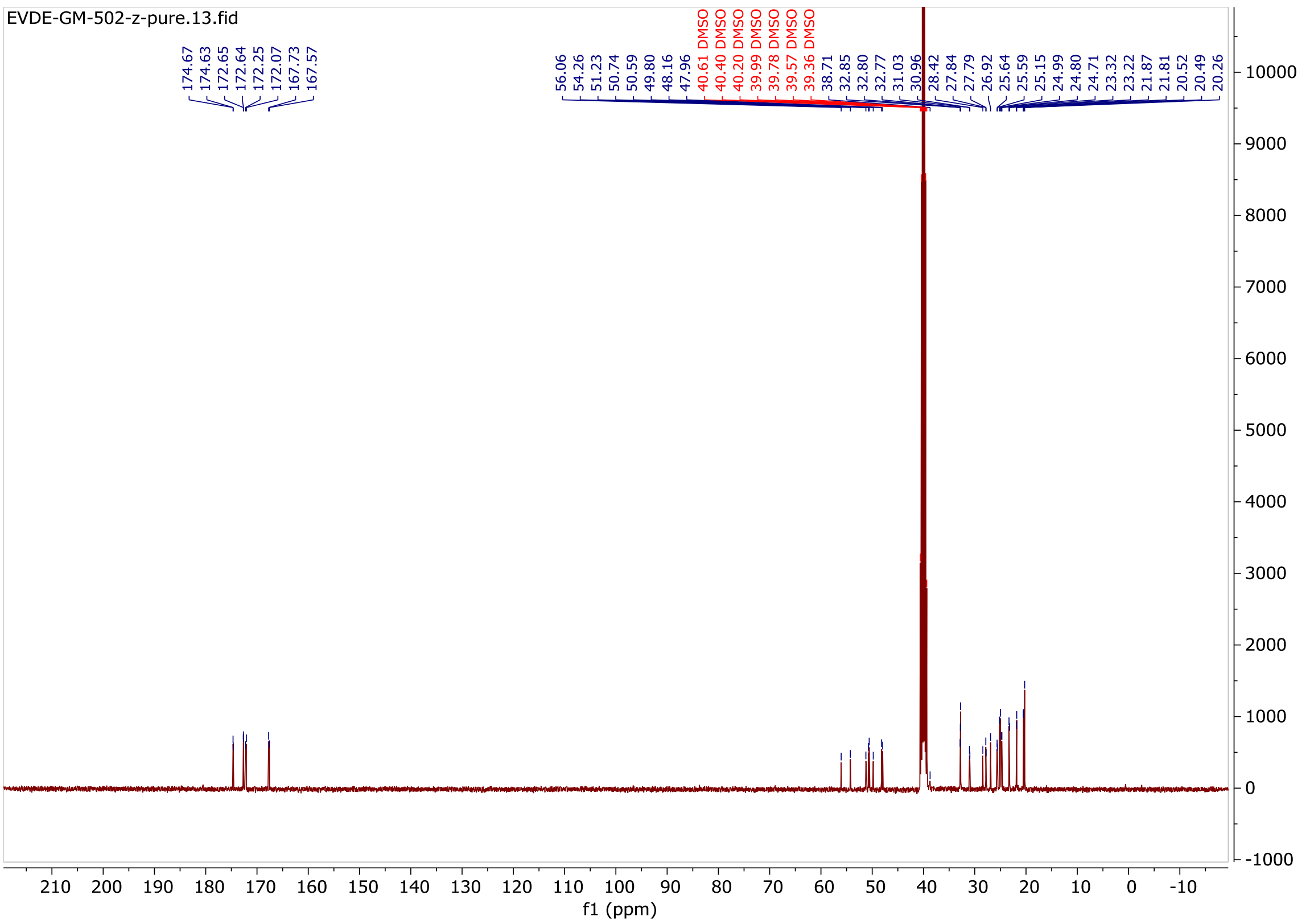

Figure S8. ^{13}C-NMR spectrum of compound **2**.

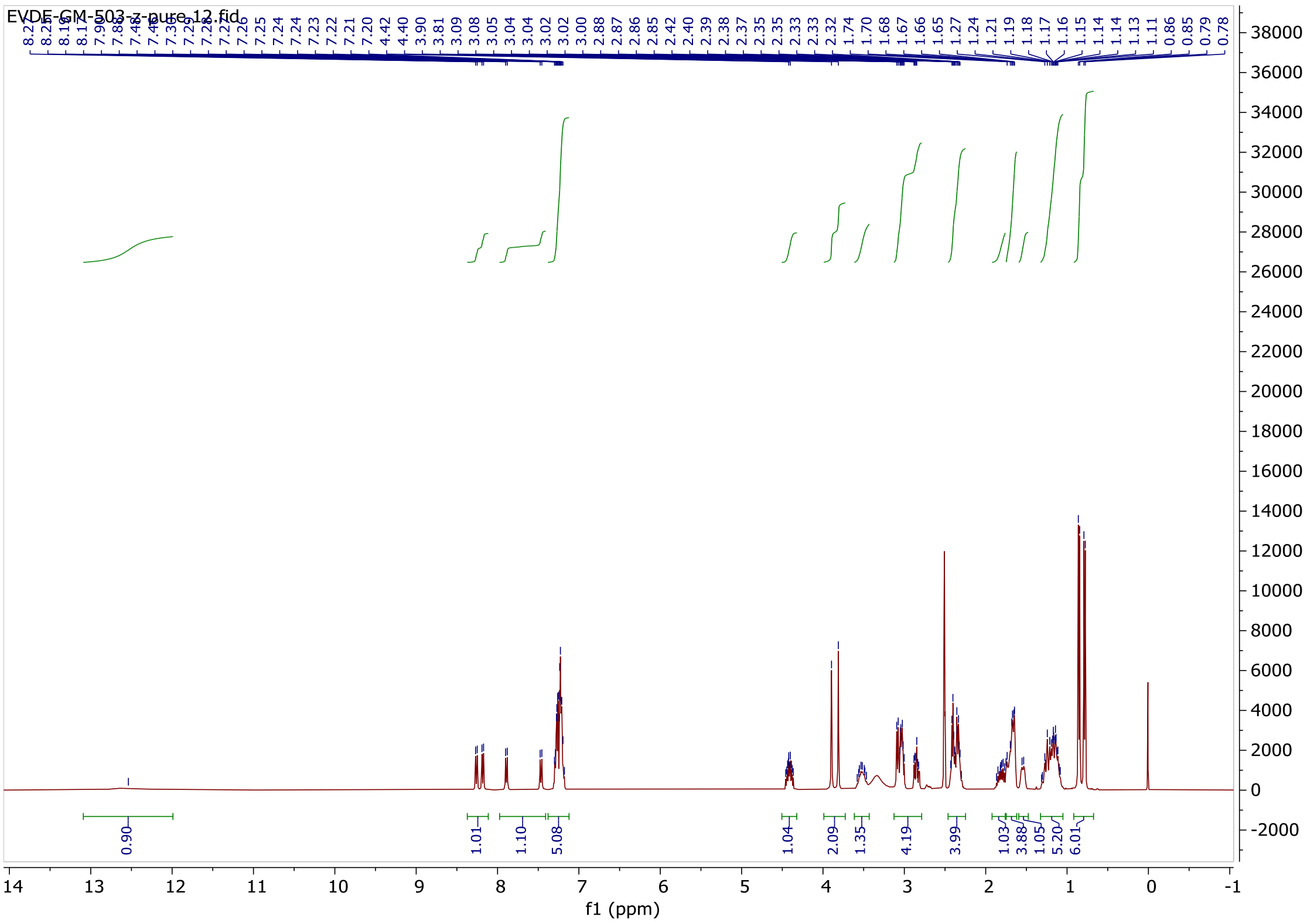

Figure S9. ^{1}H-NMR spectrum of compound **3**.

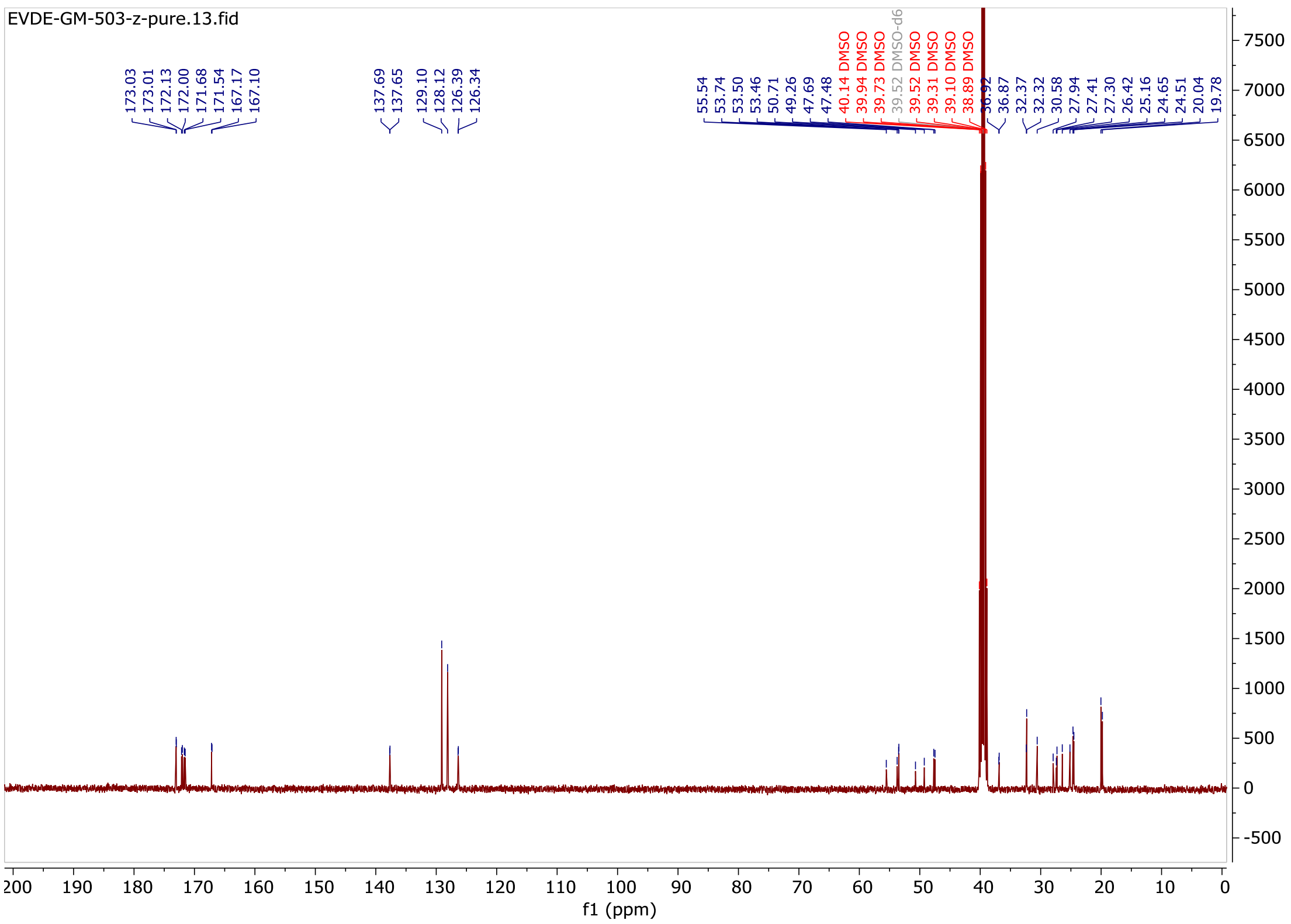

Figure S10. ^{13}C-NMR spectrum of compound **3**.

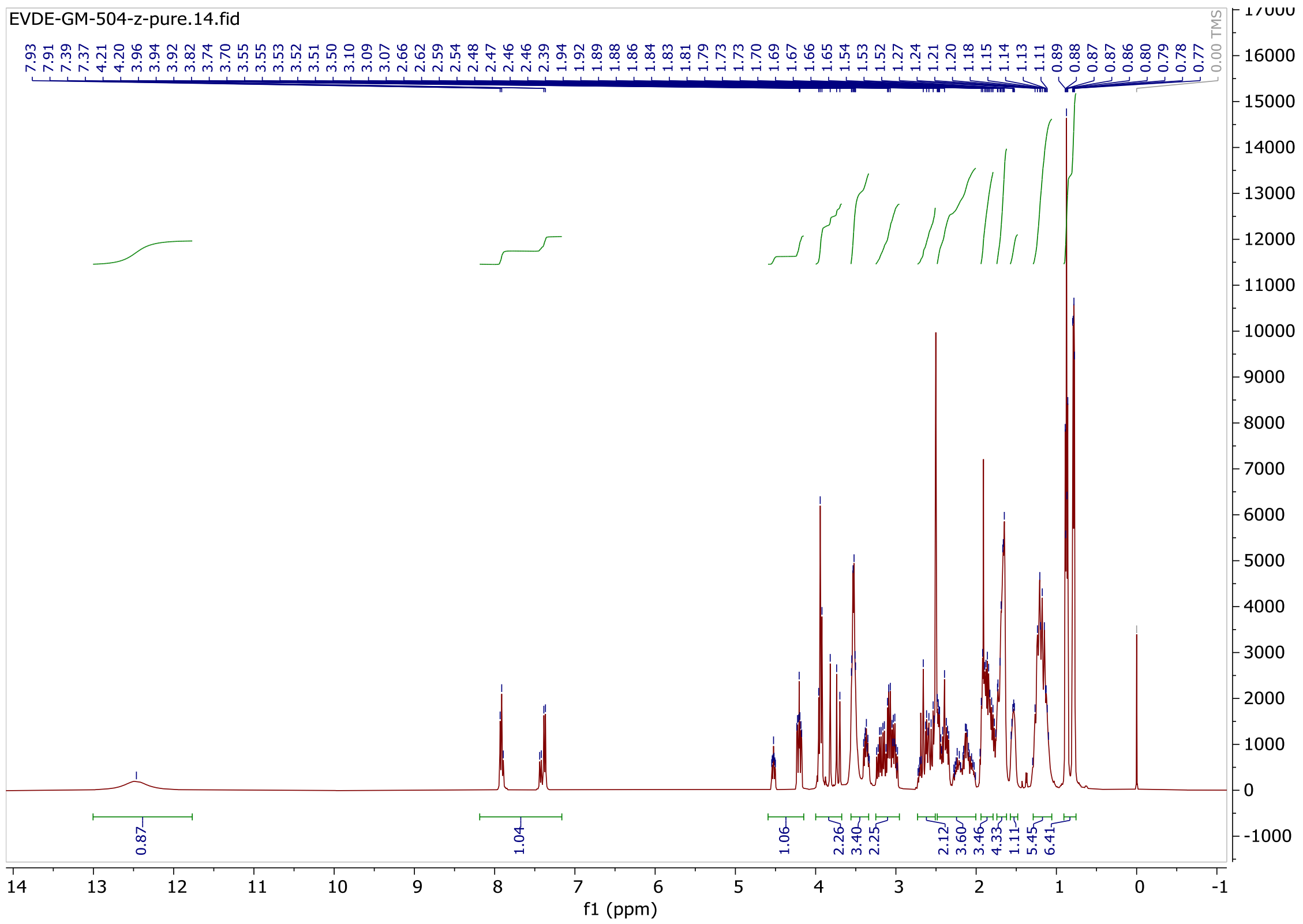

Figure S11. ^{1}H-NMR spectrum of compound **4**.

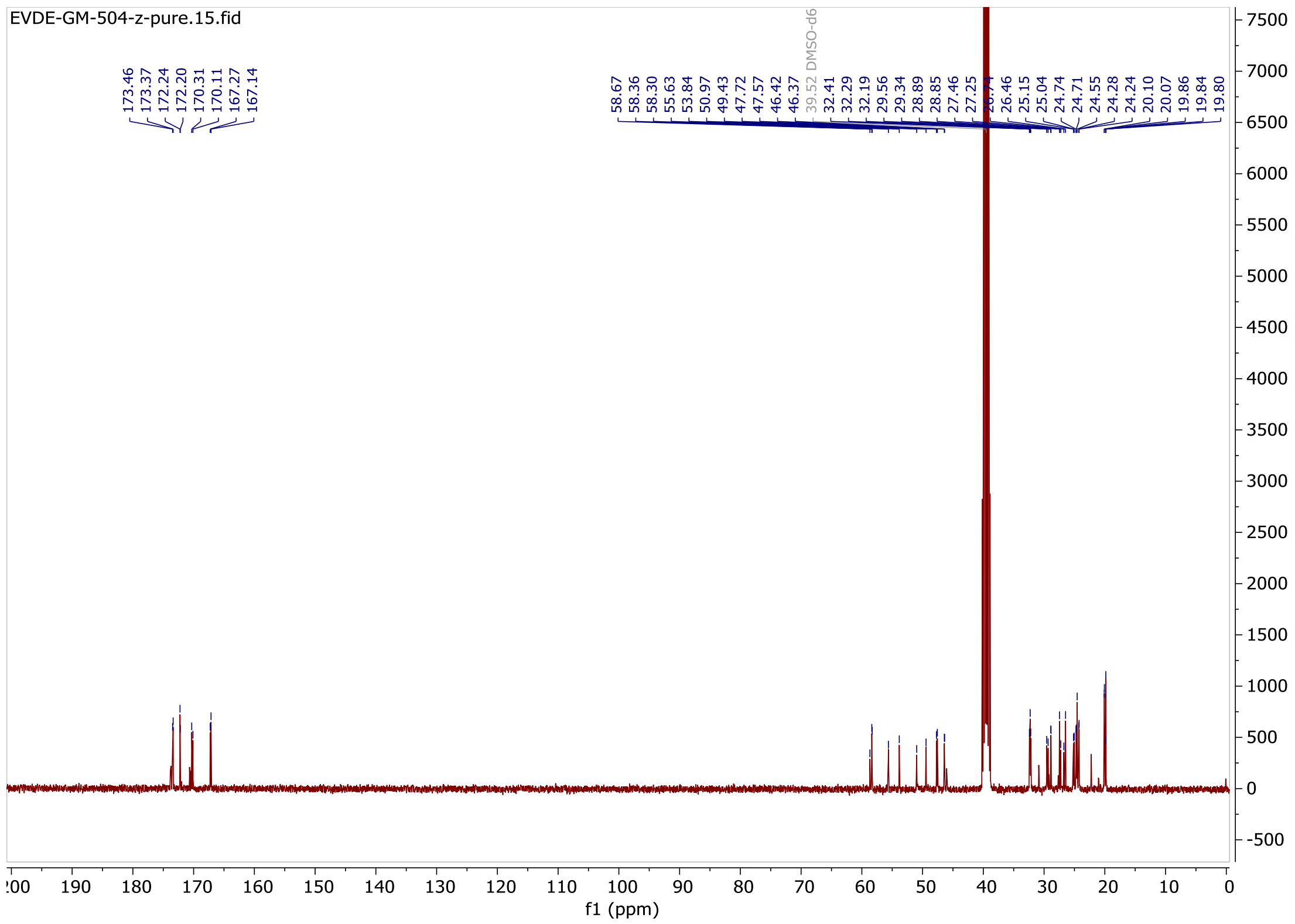

Figure S12. ^{13}C-NMR spectrum of compound **4**.

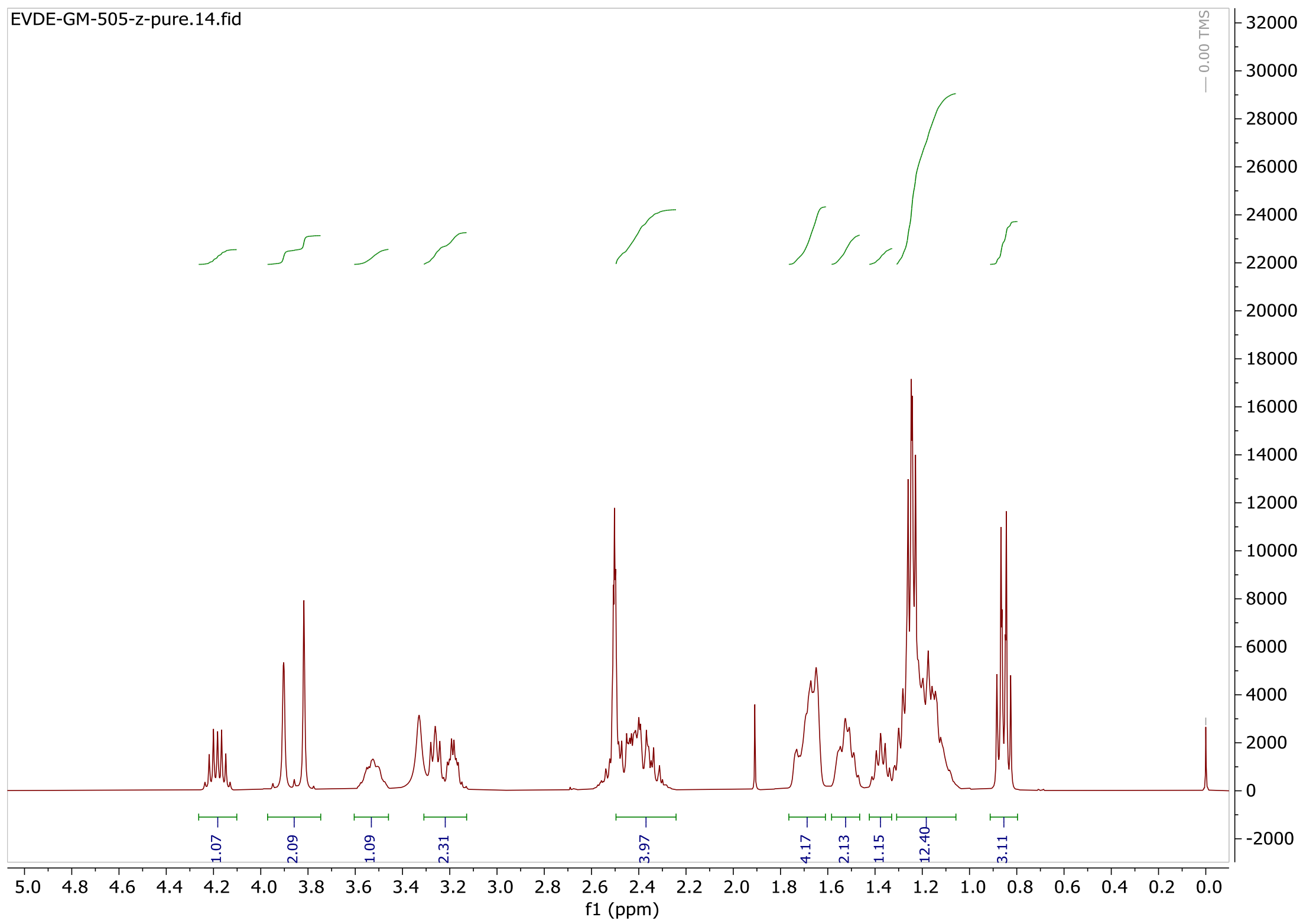

Figure S13. ^{1}H-NMR spectrum of compound **5**.

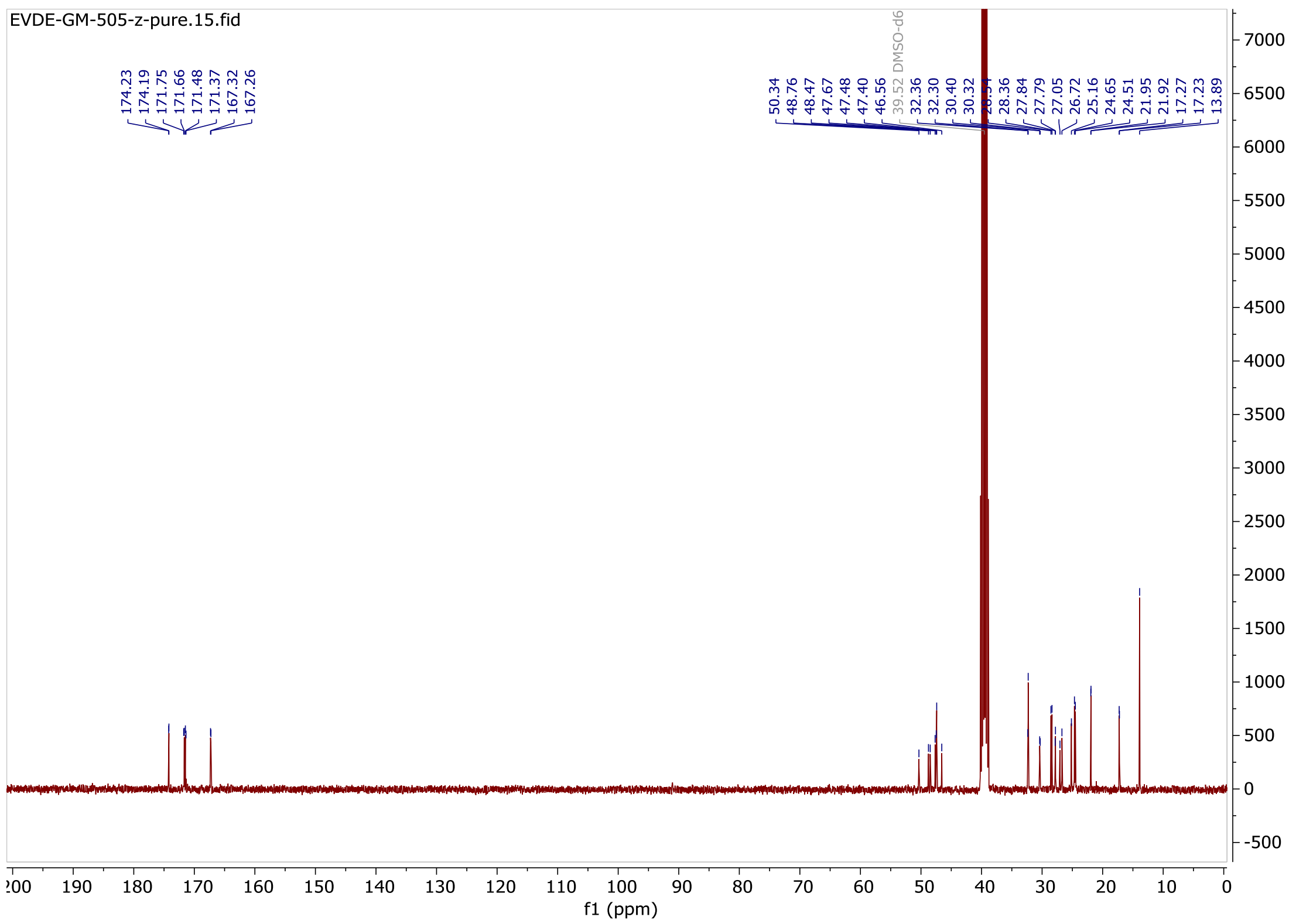

Figure S14. ^{13}C-NMR spectrum of compound **5**.

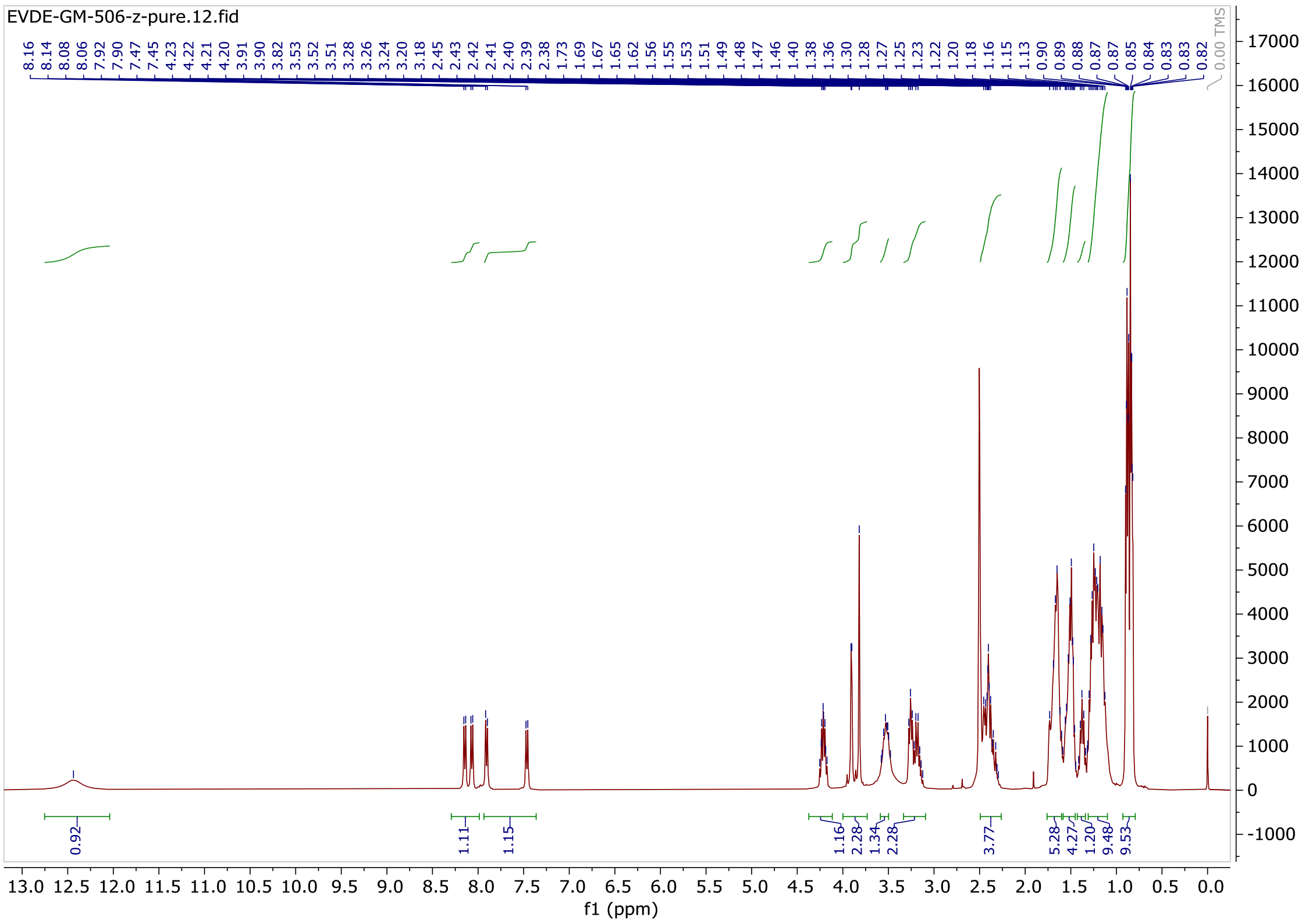

Figure S15. ^{1}H-NMR spectrum of compound **6**.

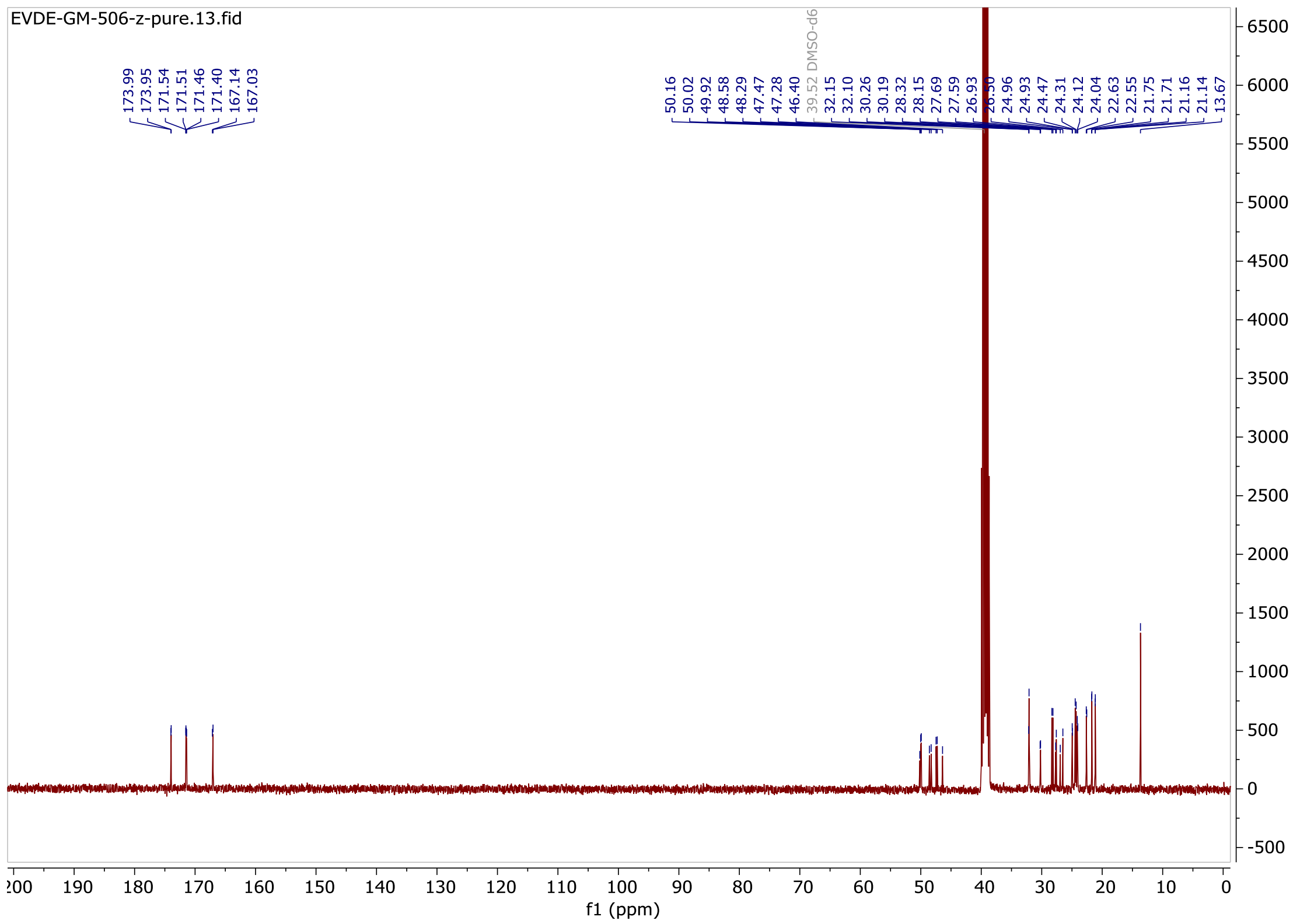

Figure S16. ^{13}C-NMR spectrum of compound **6**.

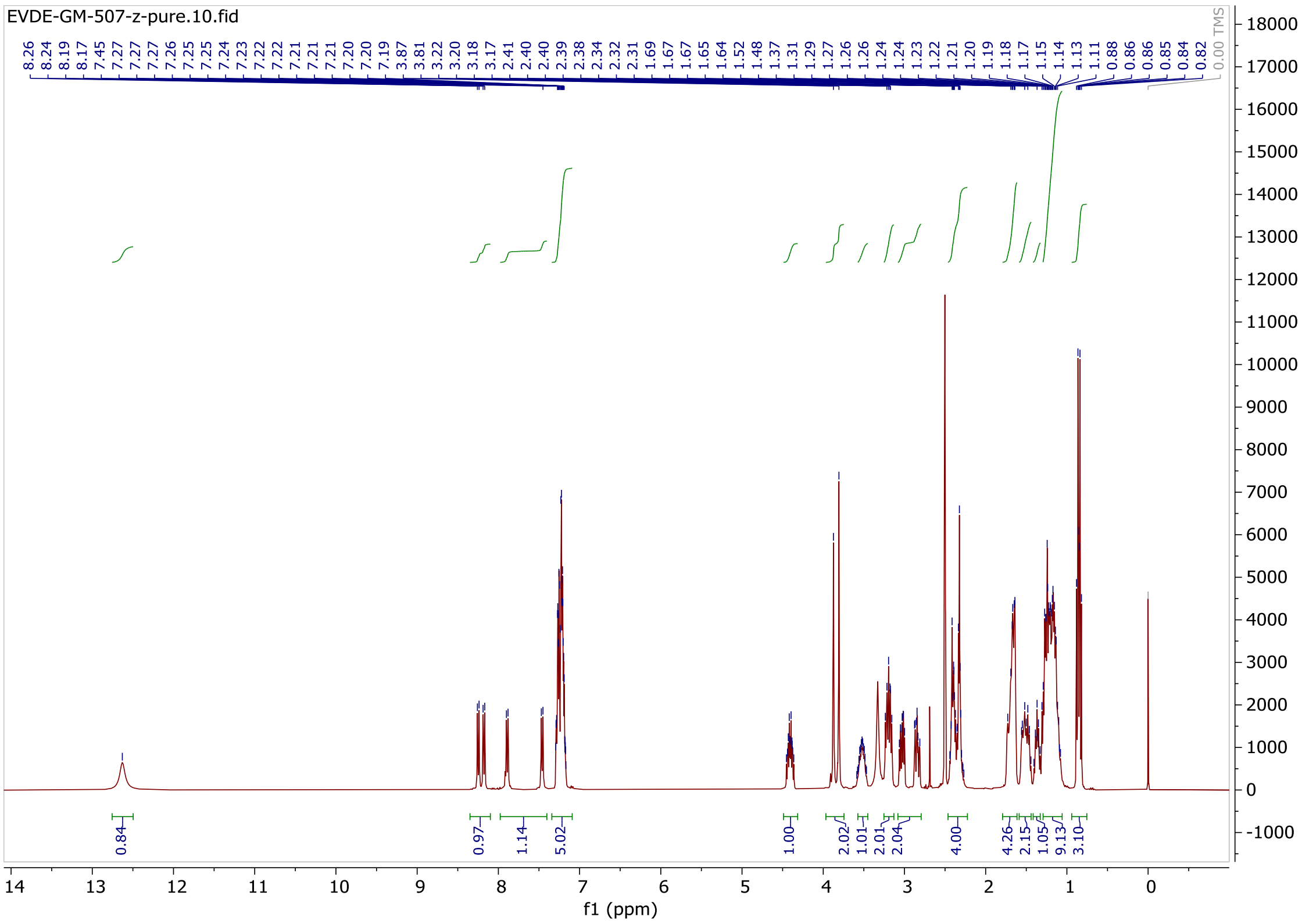

Figure S17. ^{1}H-NMR spectrum of compound **7**.

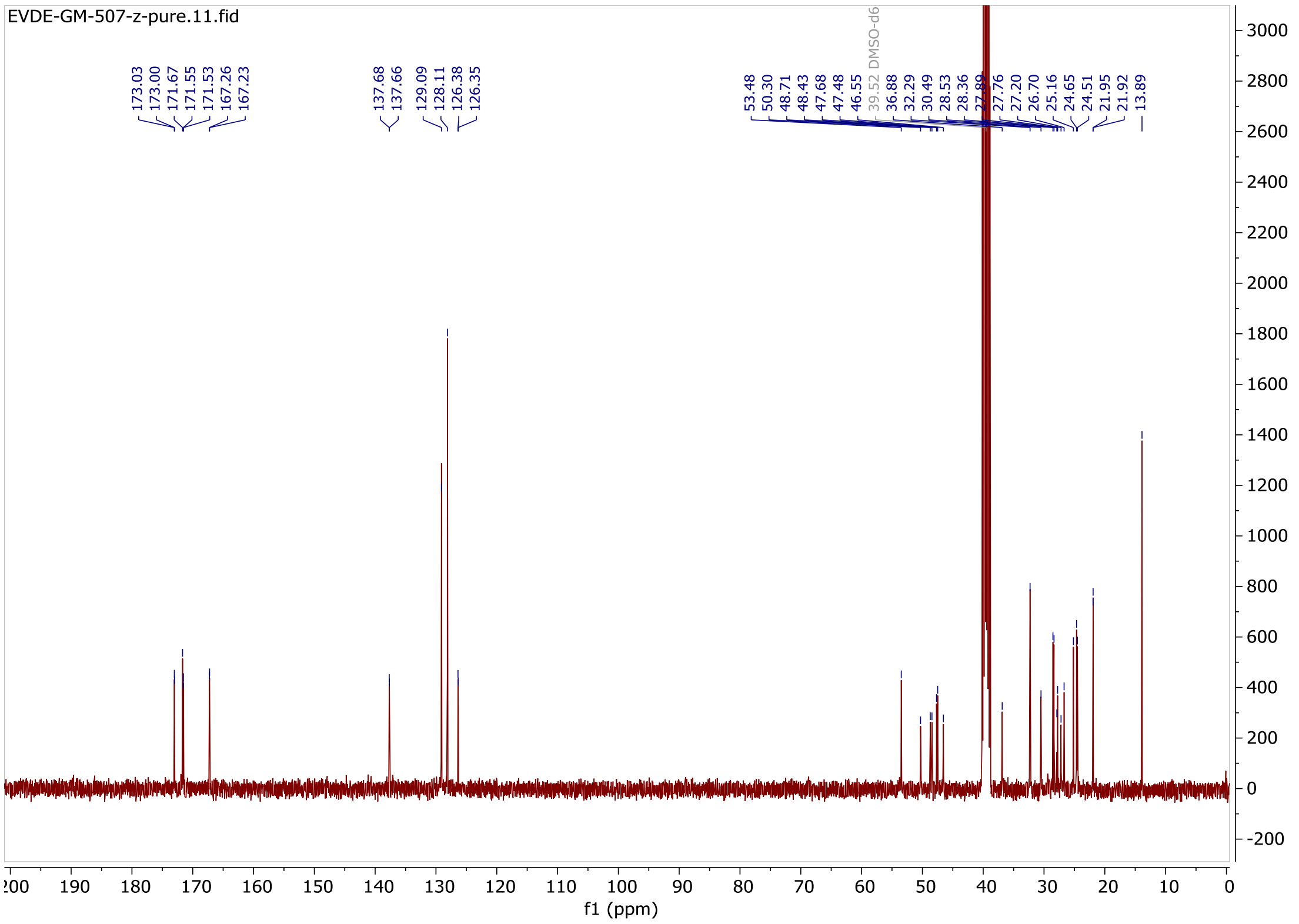

Figure S18. ^{13}C-NMR spectrum of compound **7**.

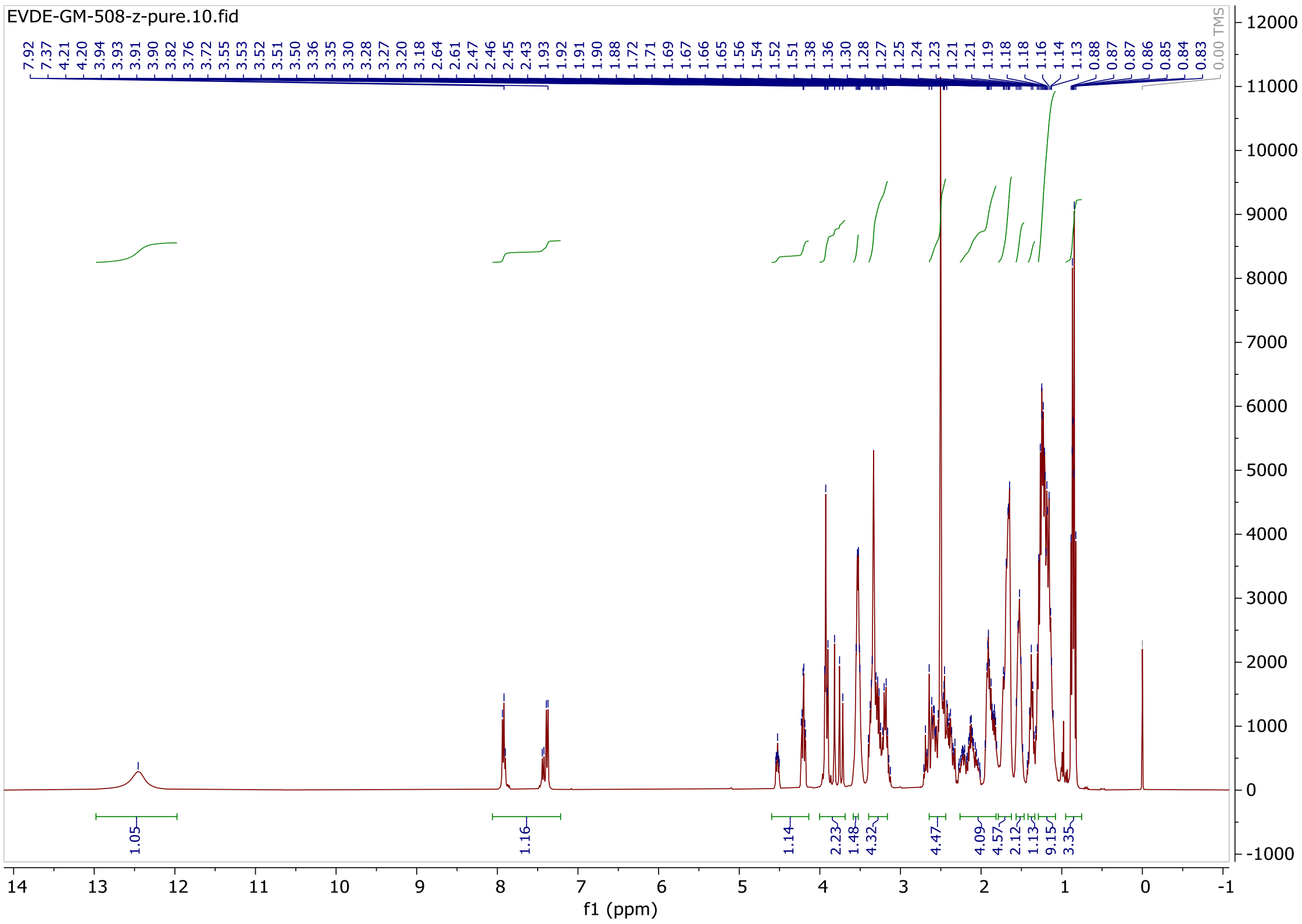

Figure S19. ^{1}H-NMR spectrum of compound **8**.

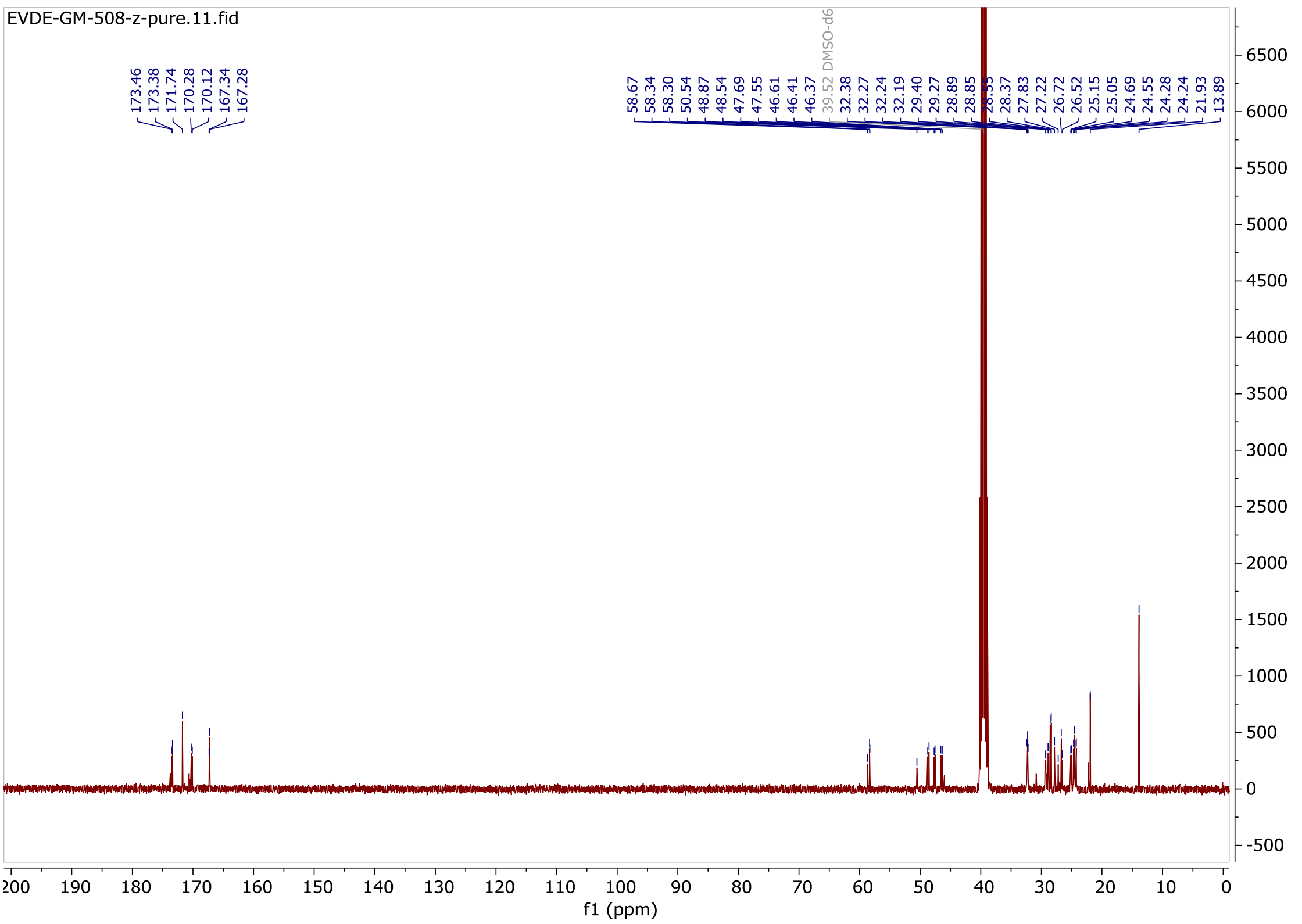

Figure S20. ^{13}C-NMR spectrum of compound **8**.

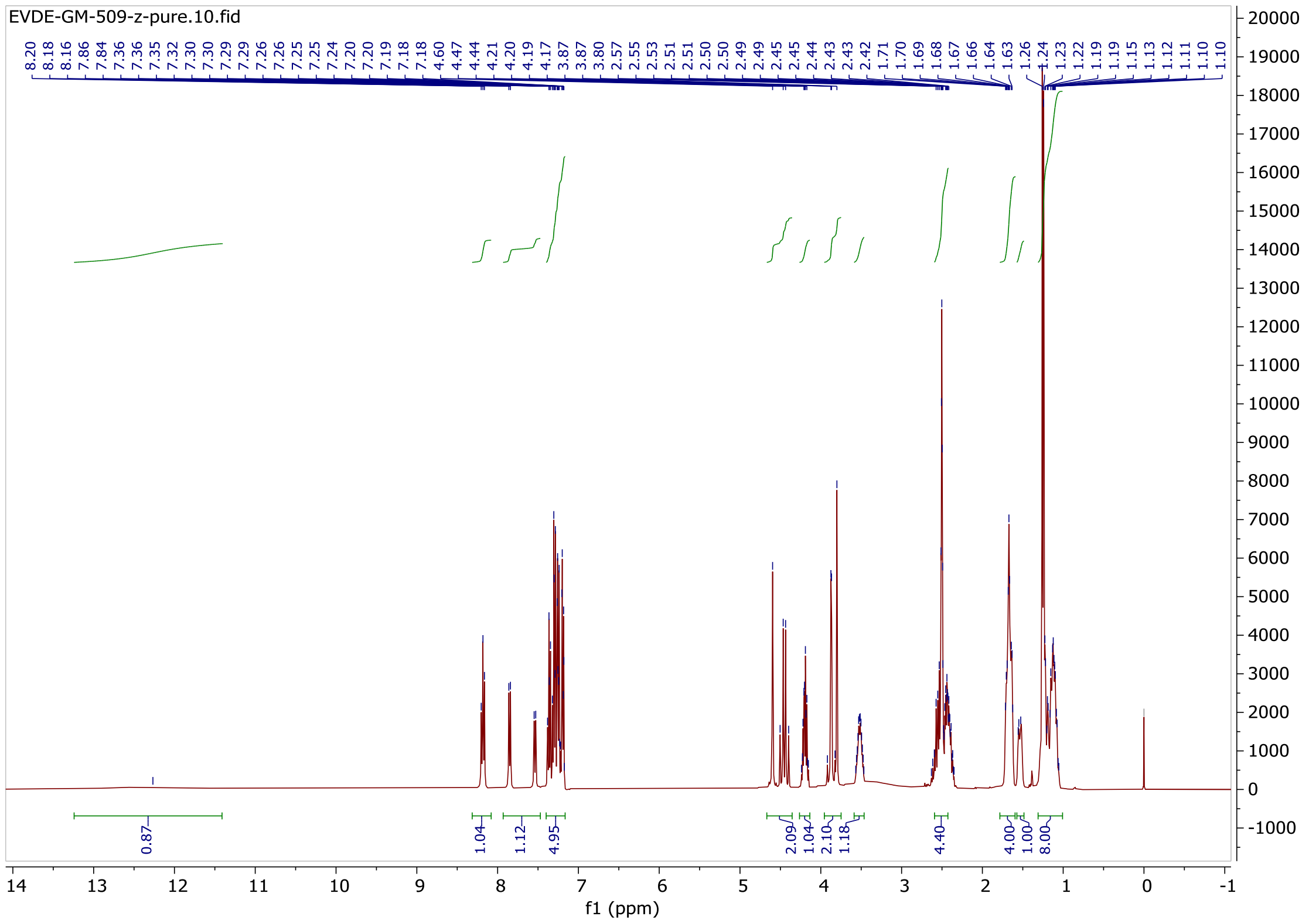

Figure S21. ^{1}H-NMR spectrum of compound **9**.

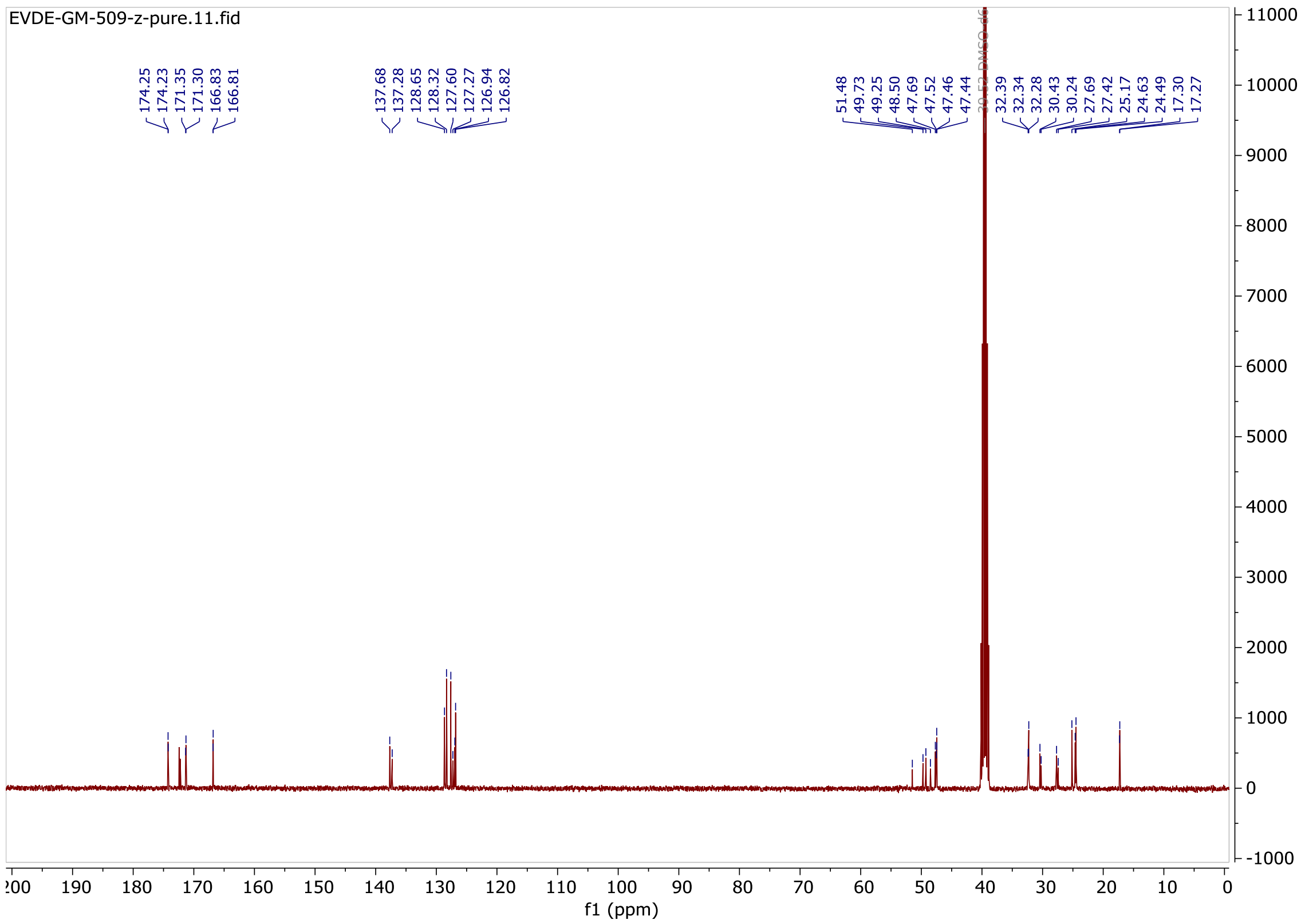

Figure S22. ^{13}C-NMR spectrum of compound **9**.

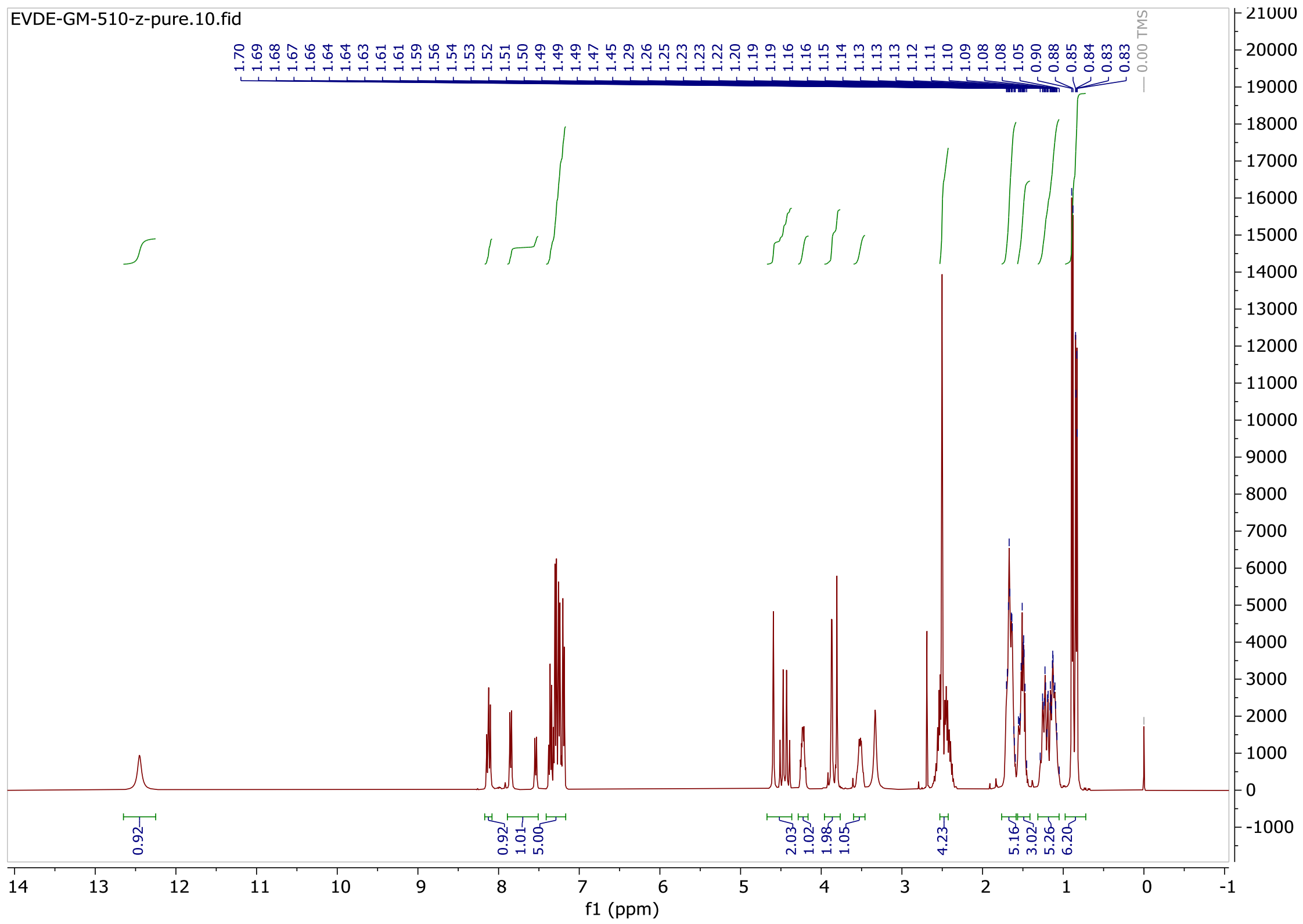

Figure S23. ^{1}H-NMR spectrum of compound **10**.

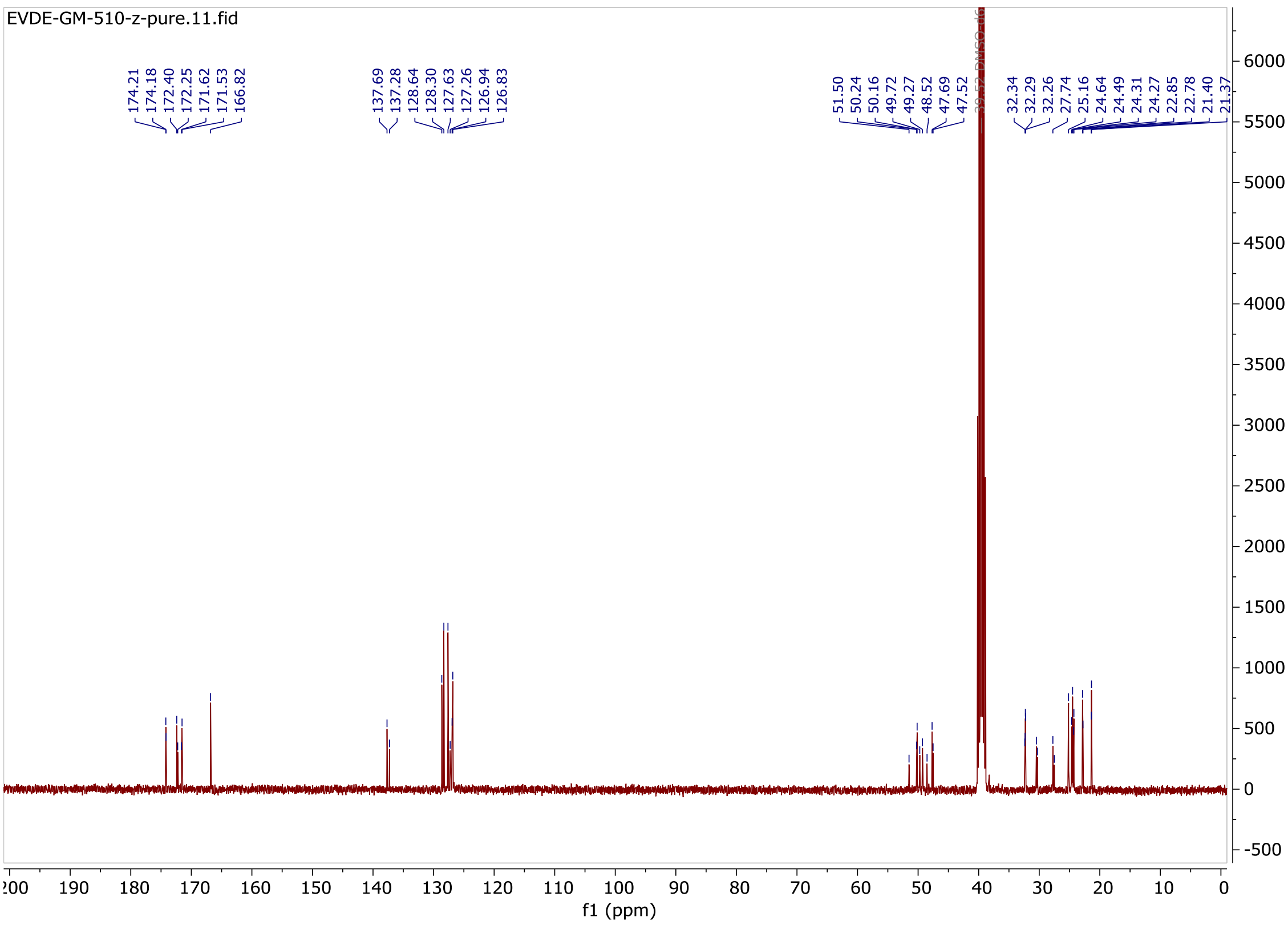

Figure S24. ^{13}C-NMR spectrum of compound **10**.

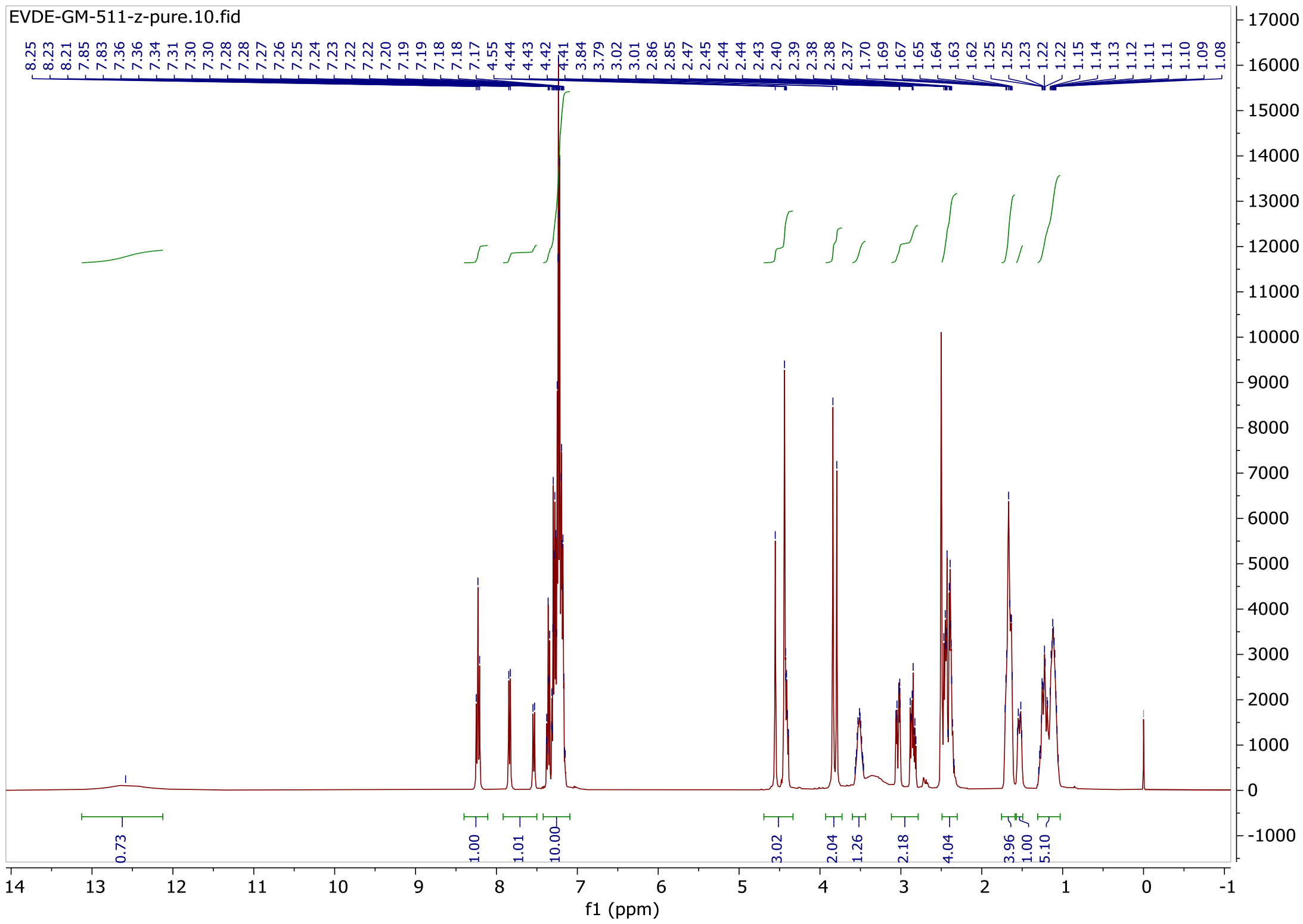

Figure S25. ^{1}H-NMR spectrum of compound **11**.

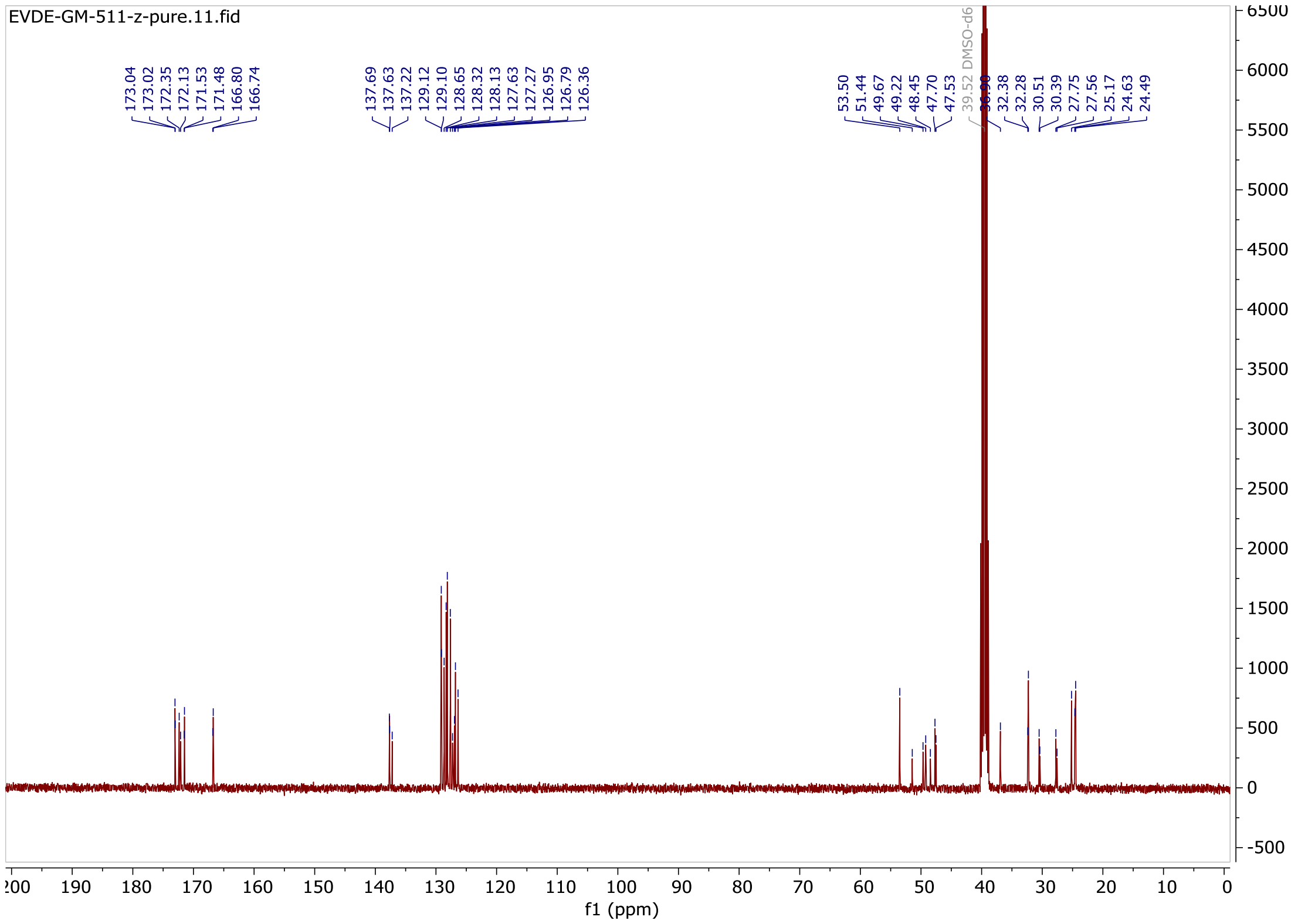

Figure S26. ^{13}C-NMR spectrum of compound **11**.

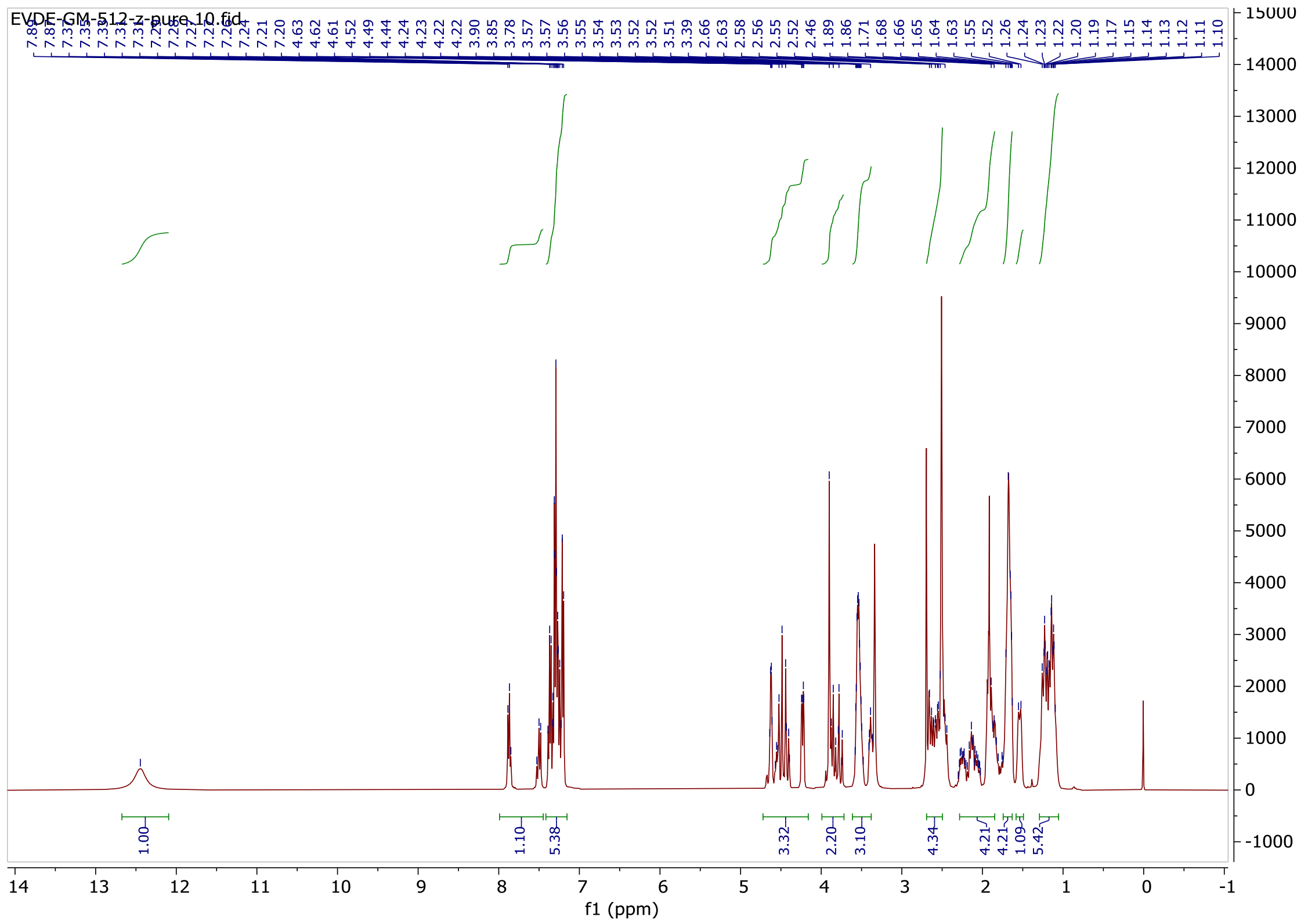

Figure S27. ^{1}H-NMR spectrum of compound **12**.

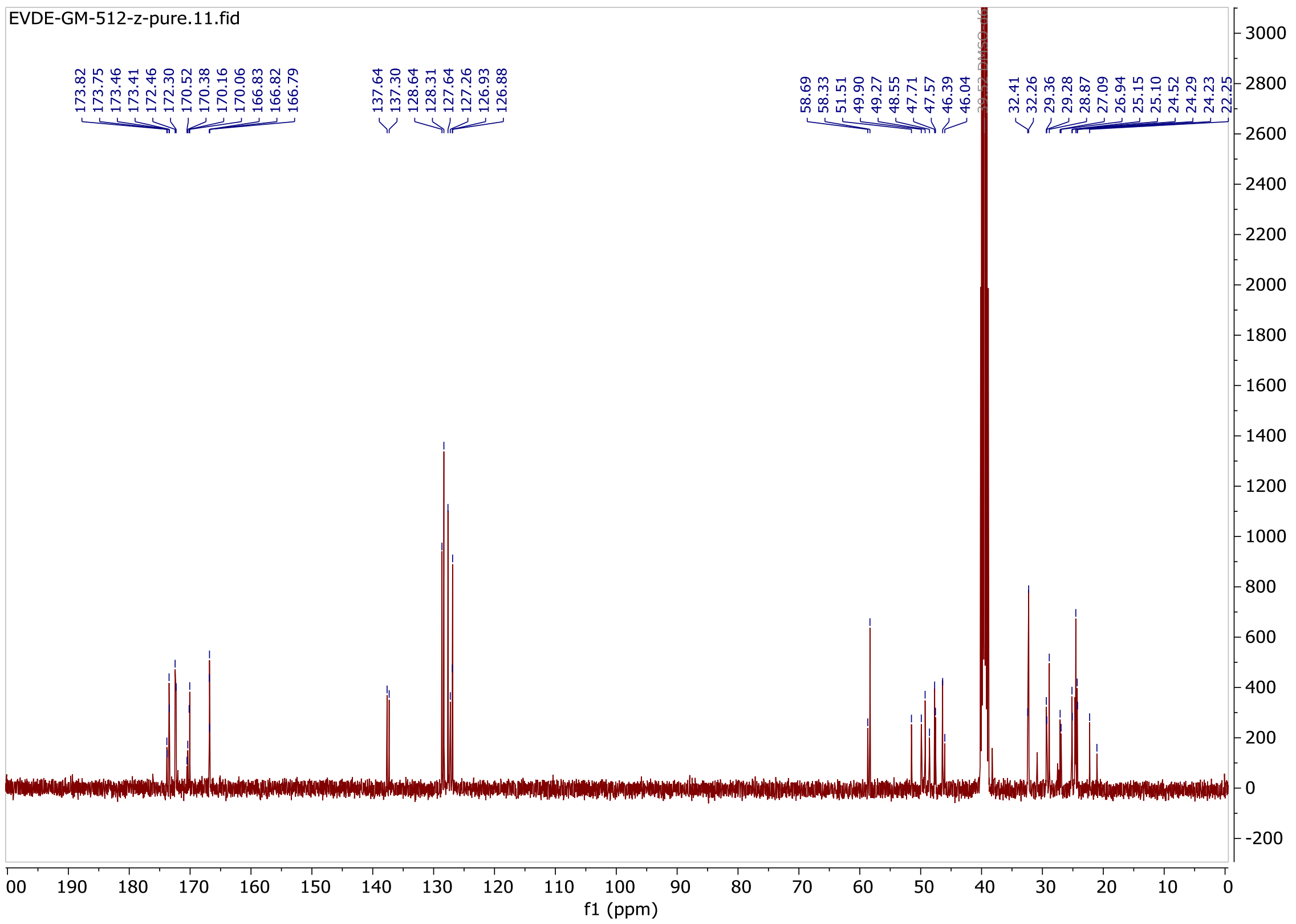

Figure S28. ^{13}C-NMR spectrum of compound **12**.

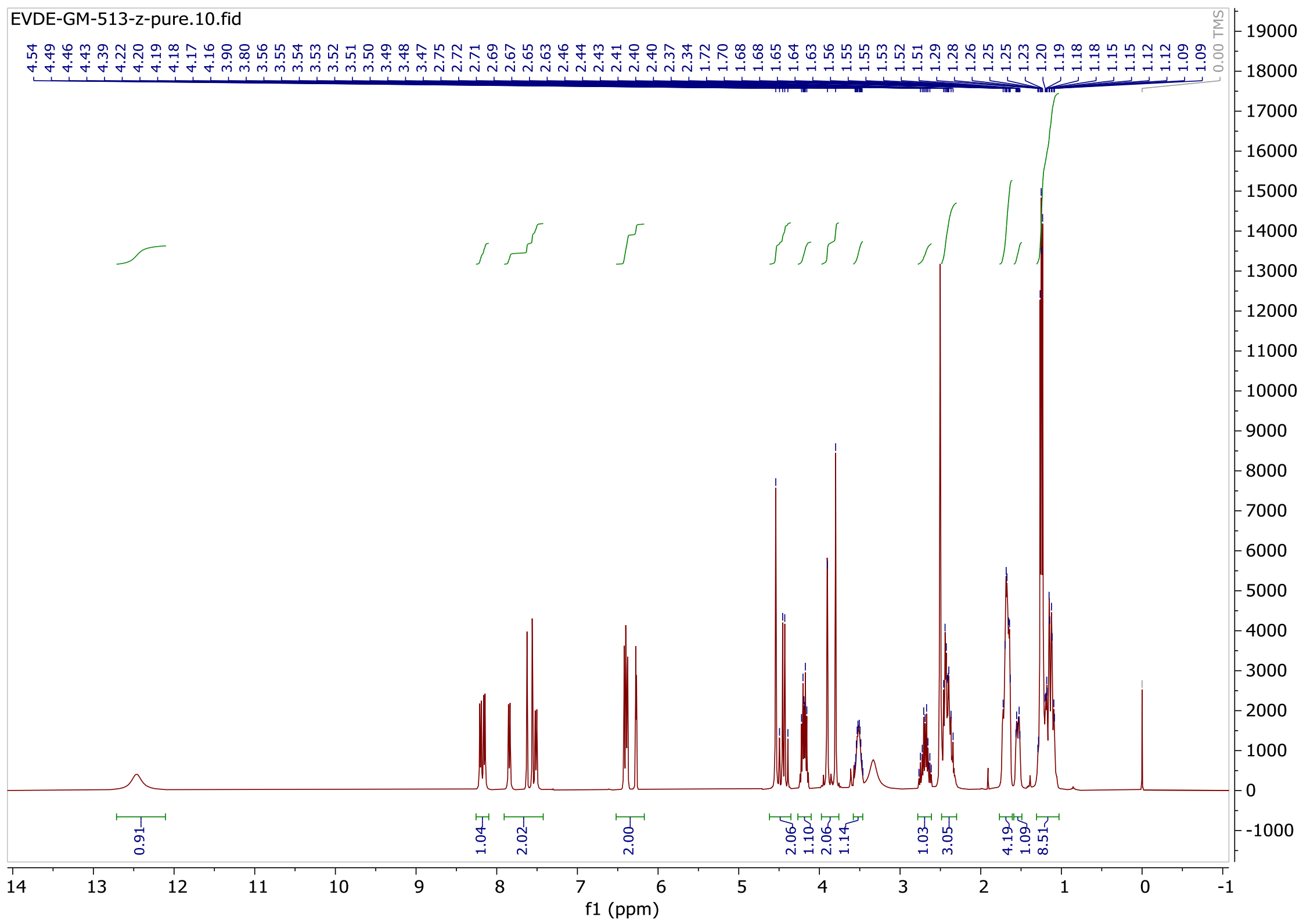

Figure S29. ^{1}H-NMR spectrum of compound **13**.

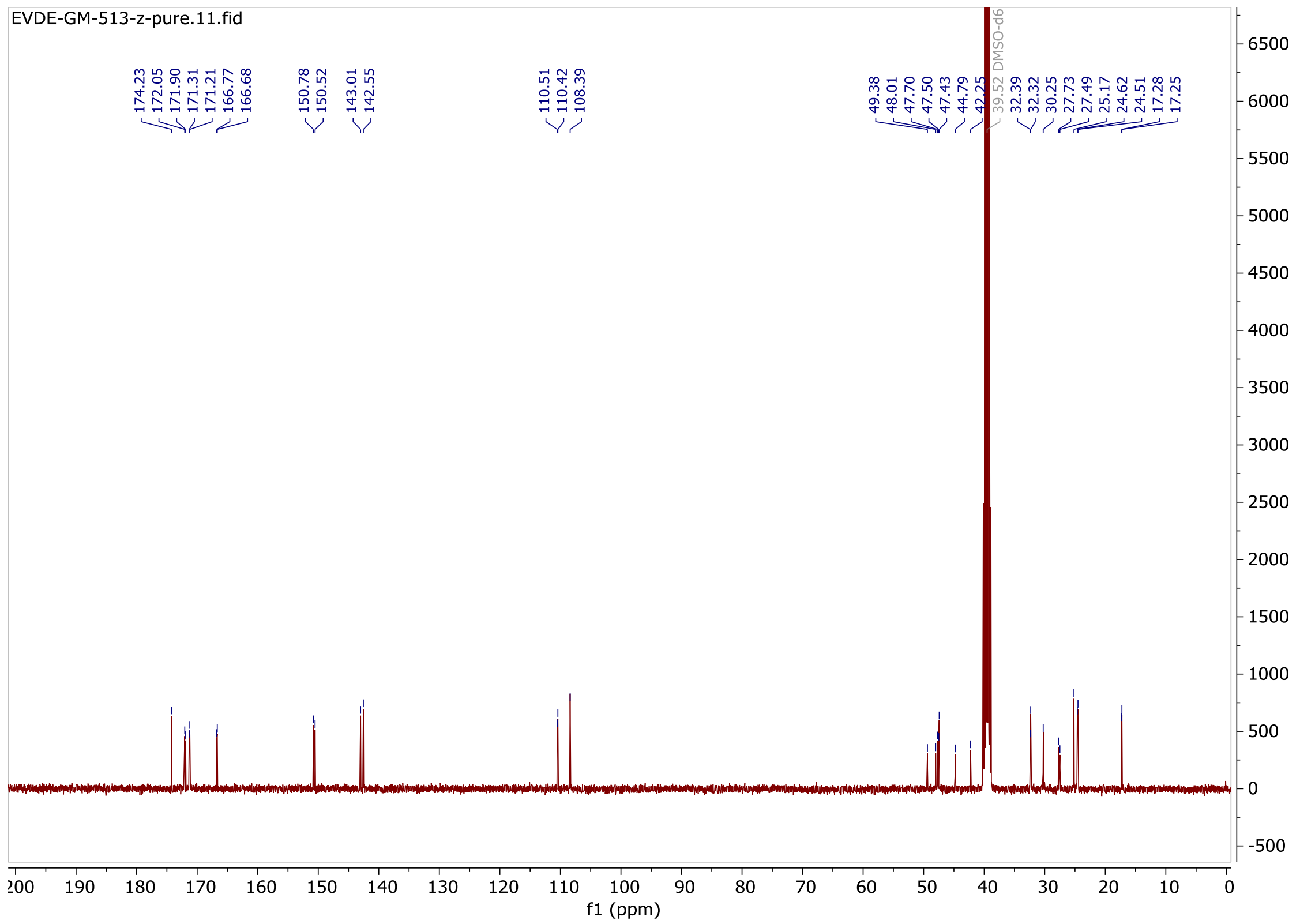

Figure S30. ^{13}C-NMR spectrum of compound **13**.

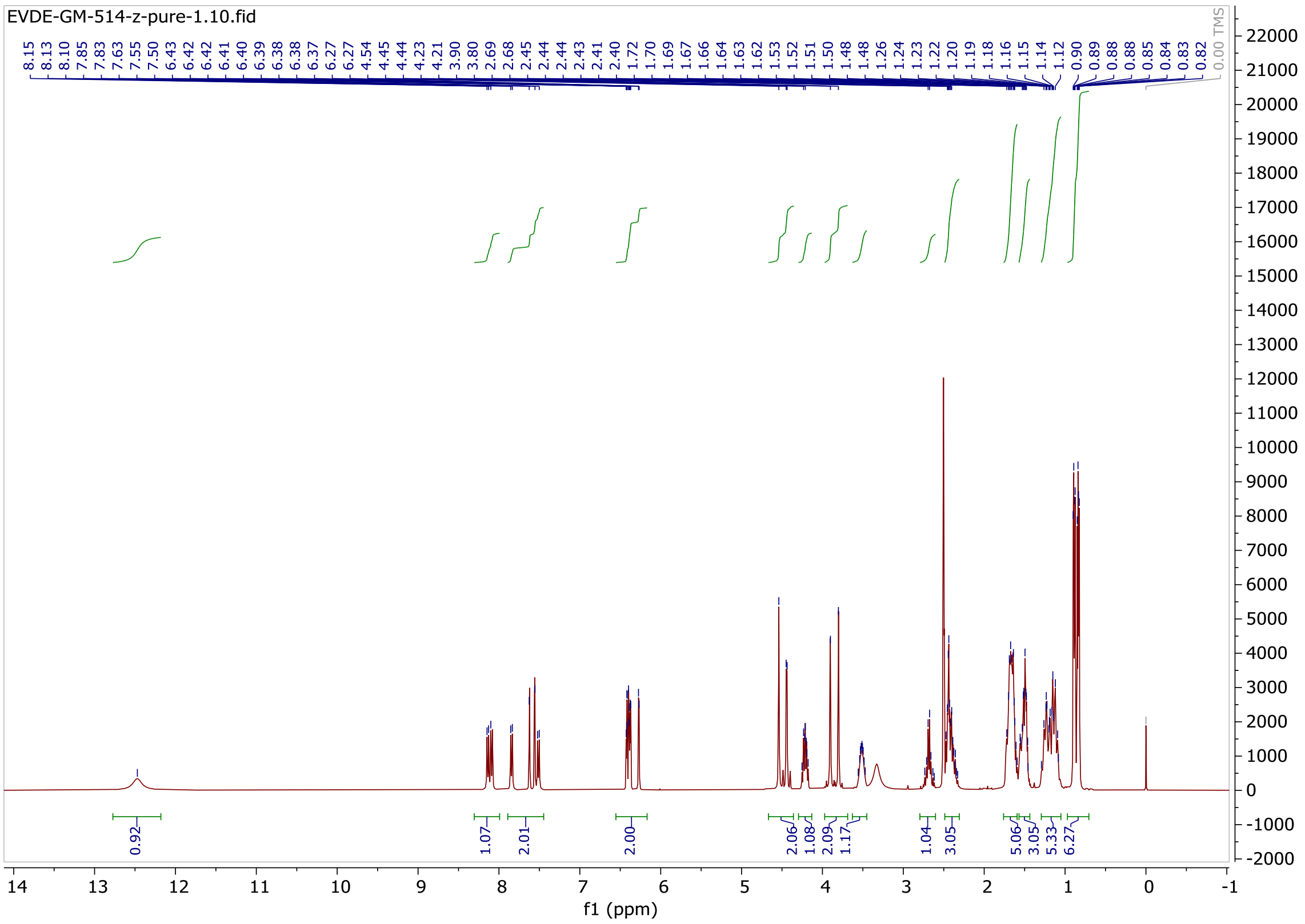

Figure S31. ^{1}H-NMR spectrum of compound **14**.

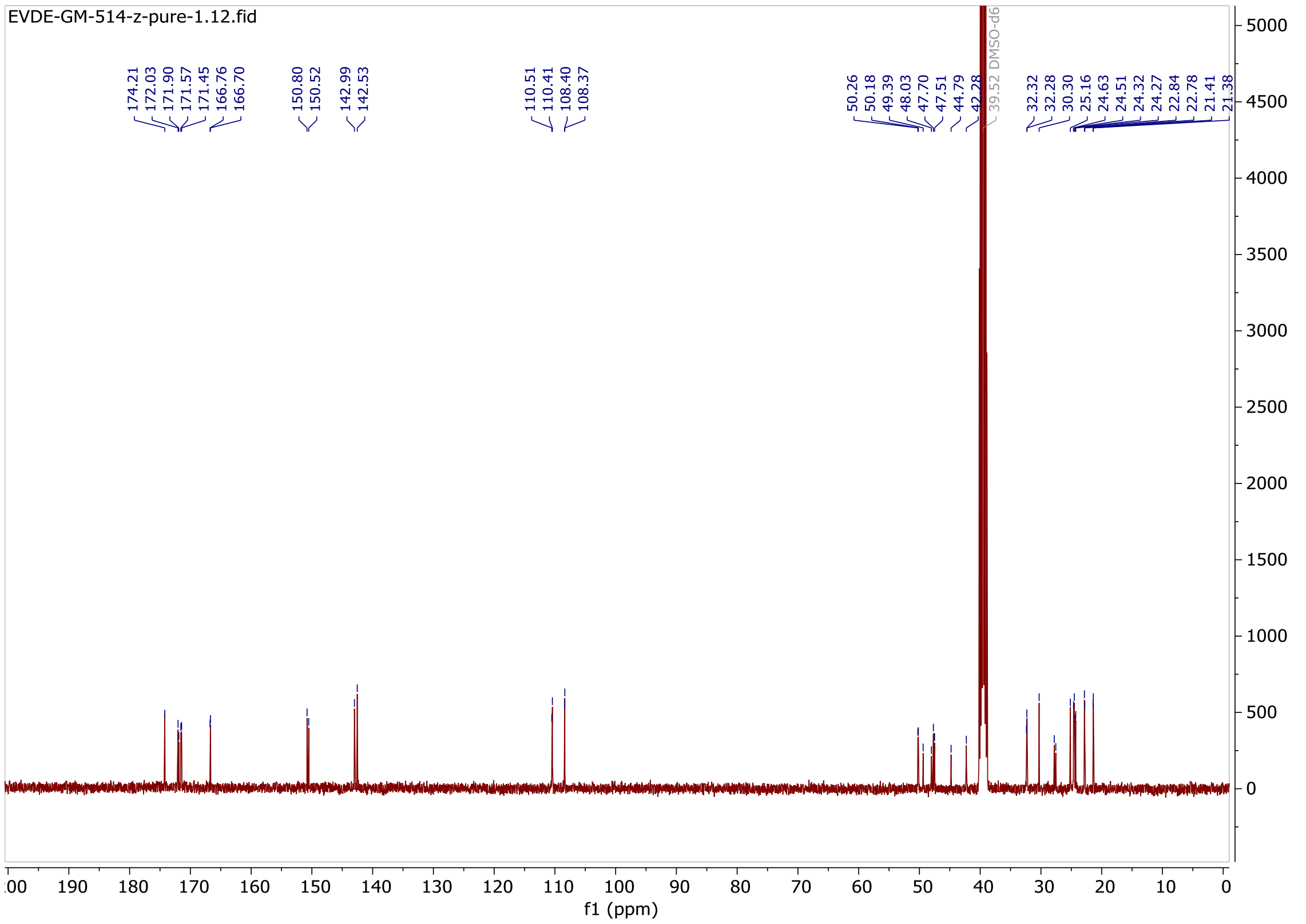

Figure S32. ^{13}C-NMR spectrum of compound **14**.

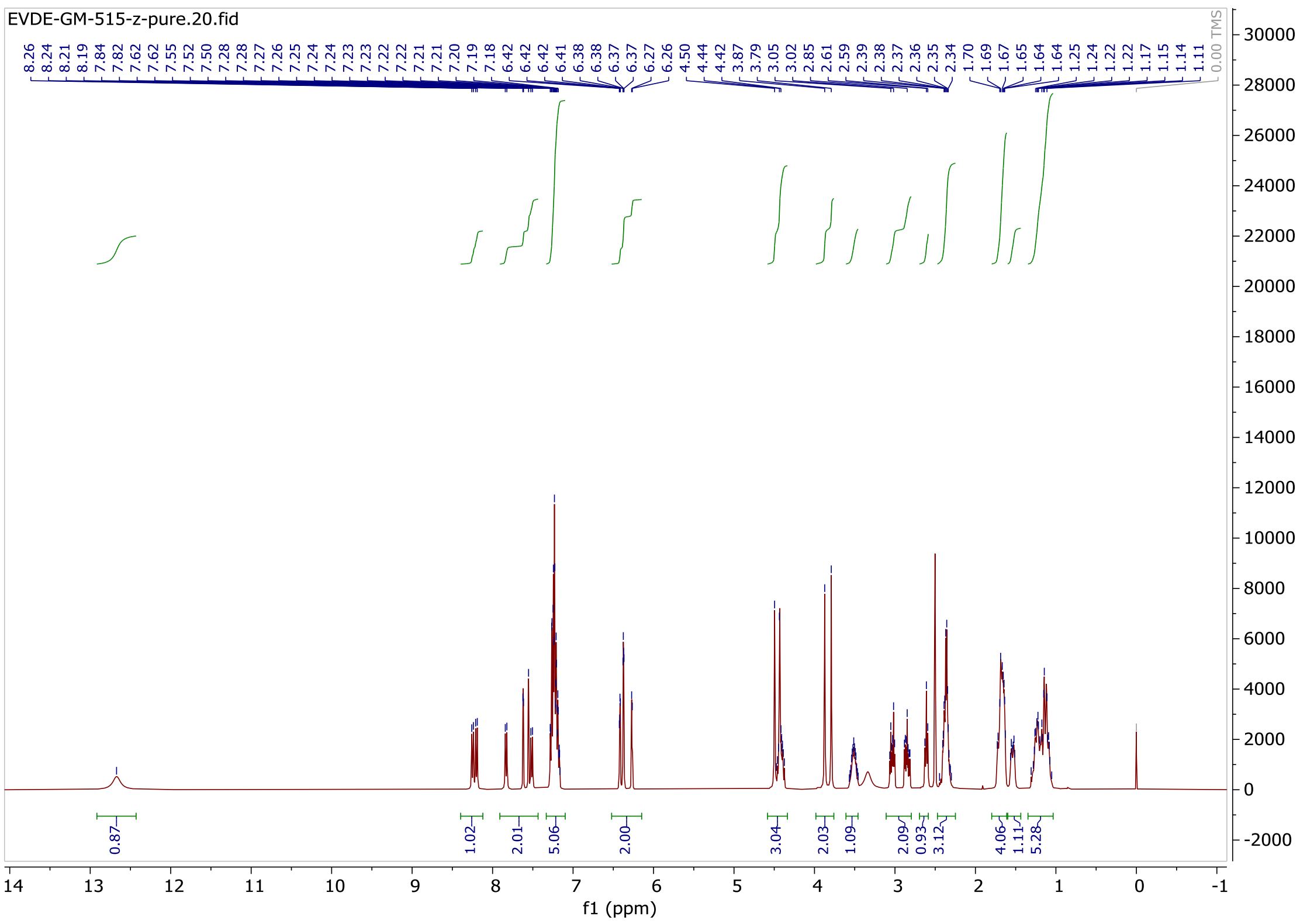

Figure S33. ^{1}H-NMR spectrum of compound **15**.

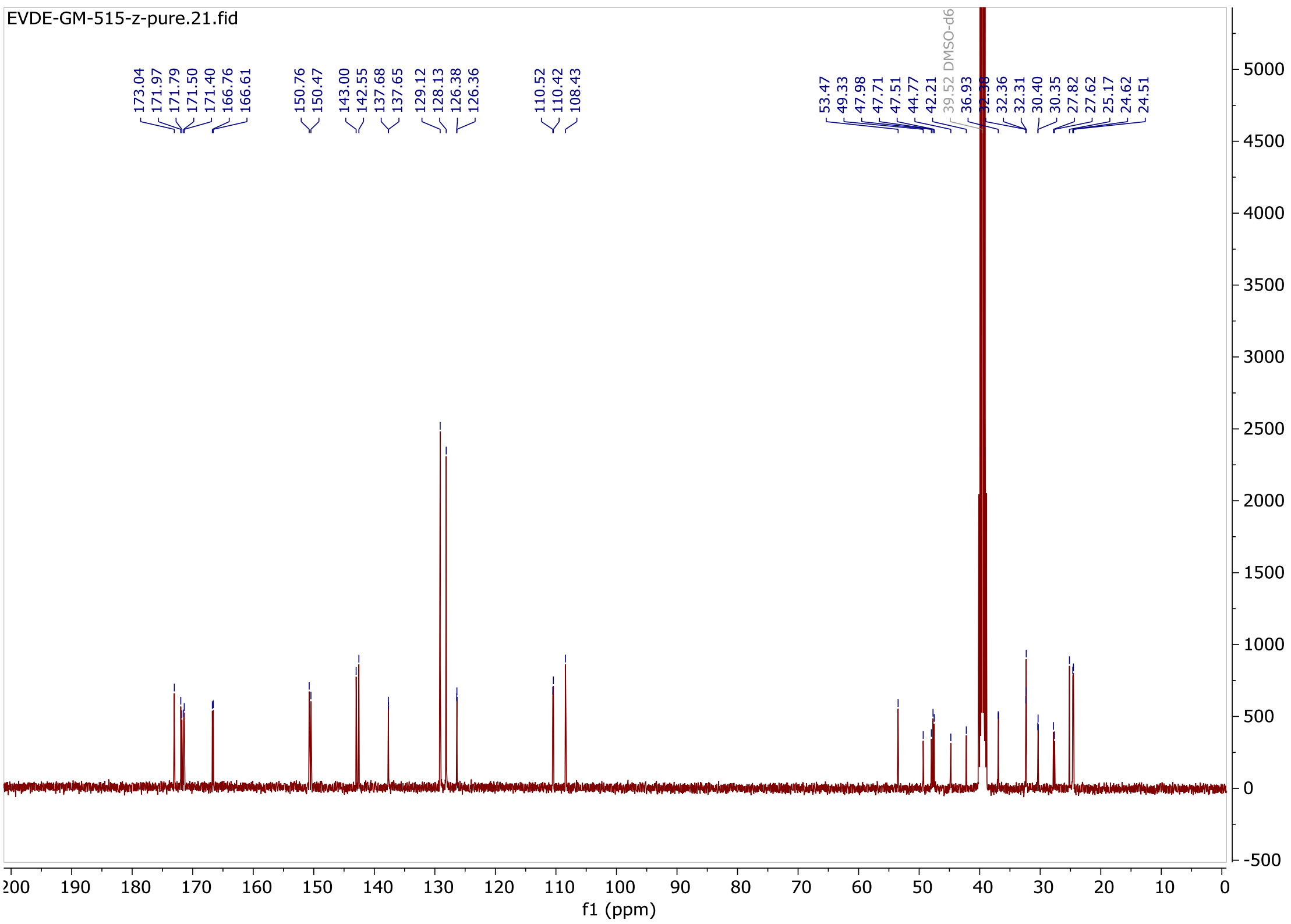

Figure S34. ^{13}C-NMR spectrum of compound **15**.

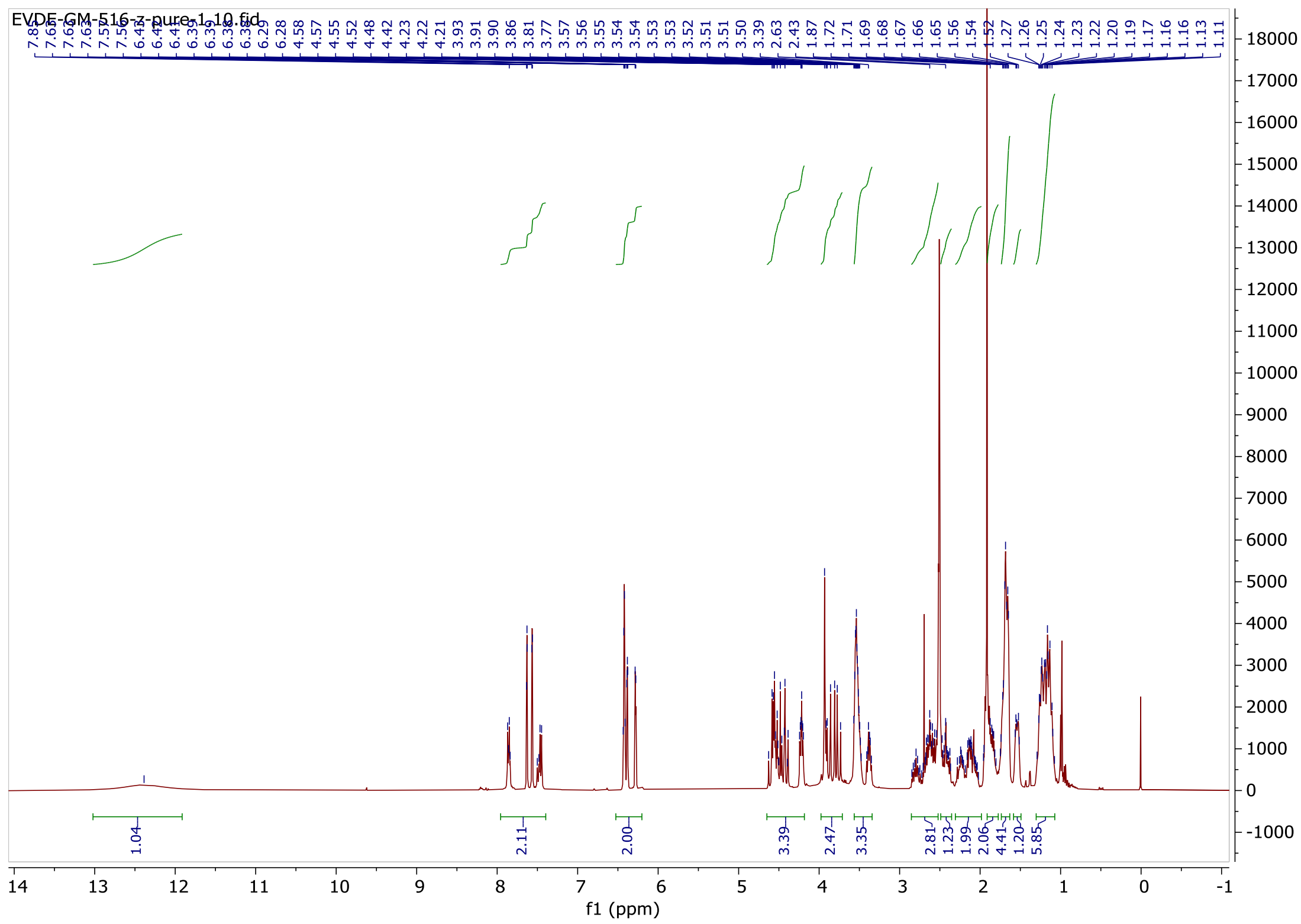

Figure S35. ^{1}H-NMR spectrum of compound **16**.

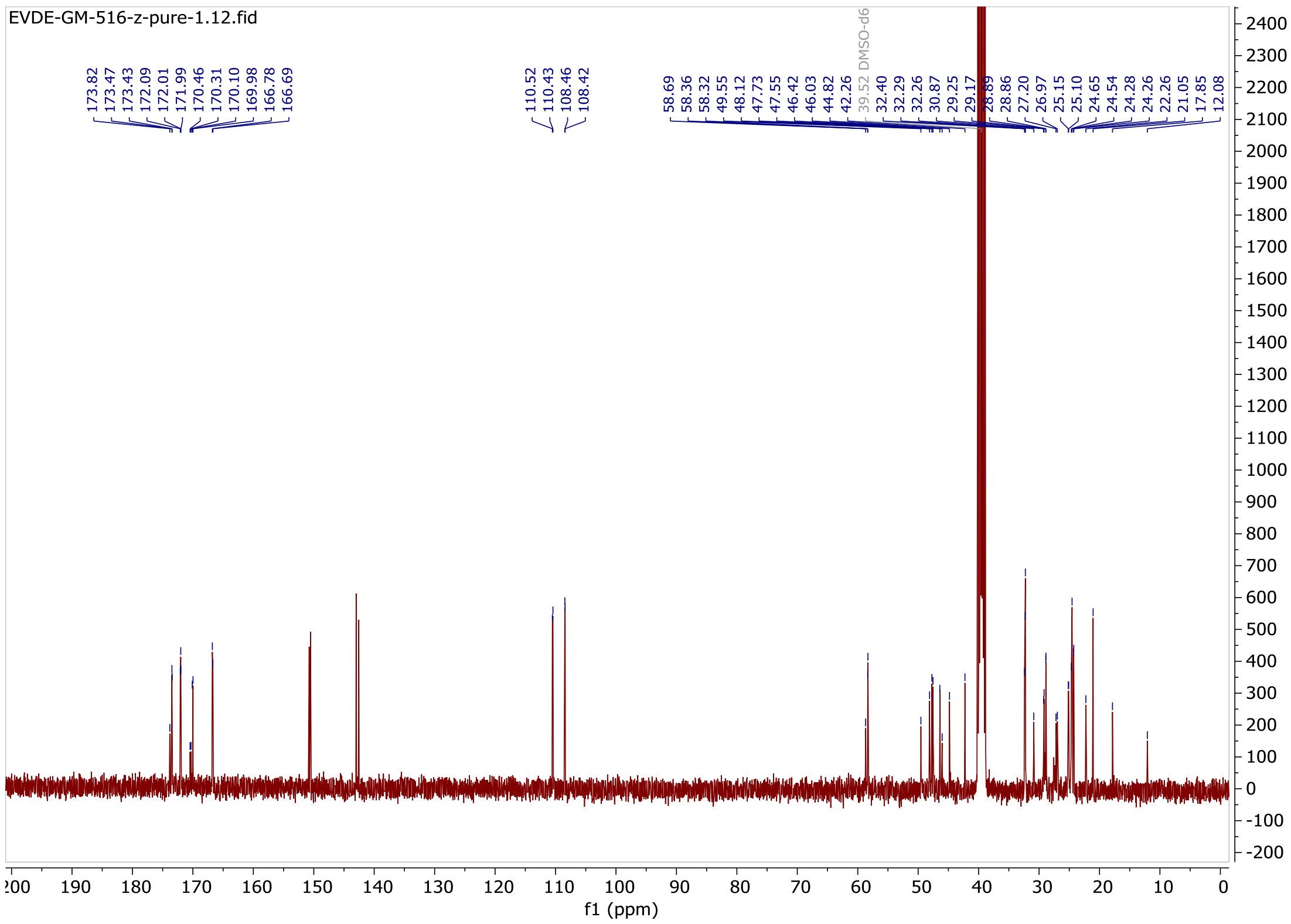

Figure S36. ^{13}C-NMR spectrum of compound **16**.

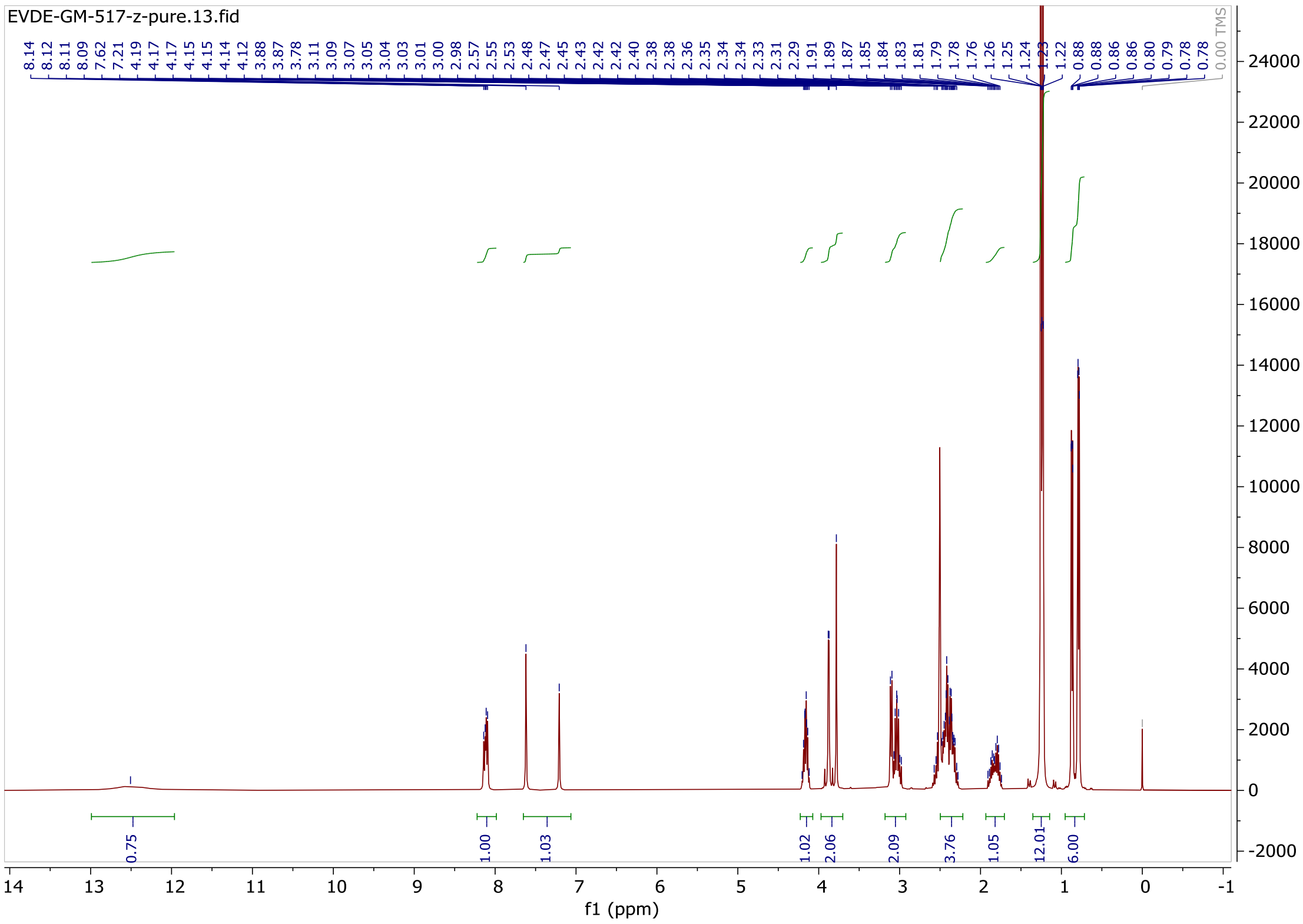

Figure S37. ^{1}H-NMR spectrum of compound **17**.

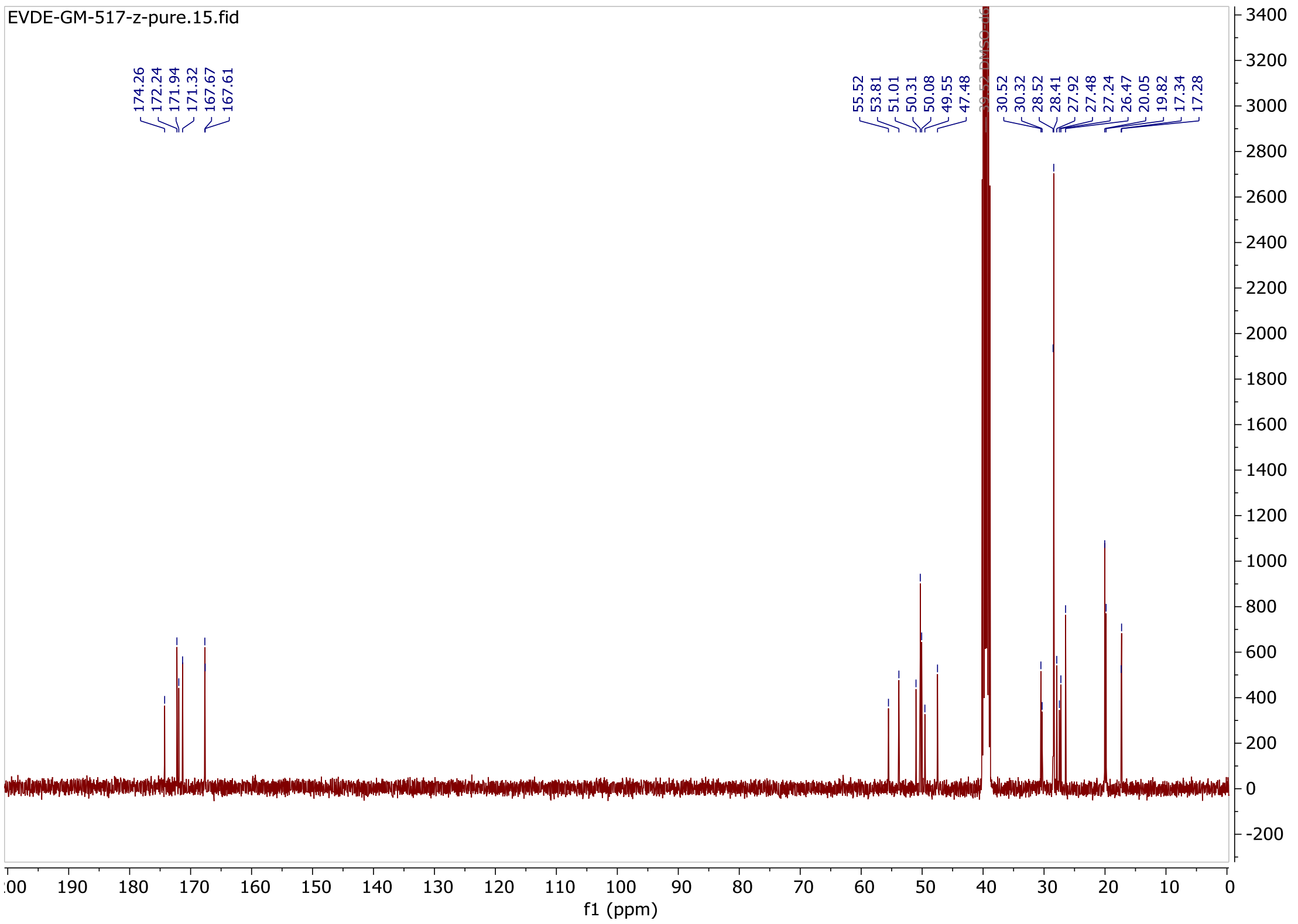

Figure S38. ^{13}C-NMR spectrum of compound **17**.

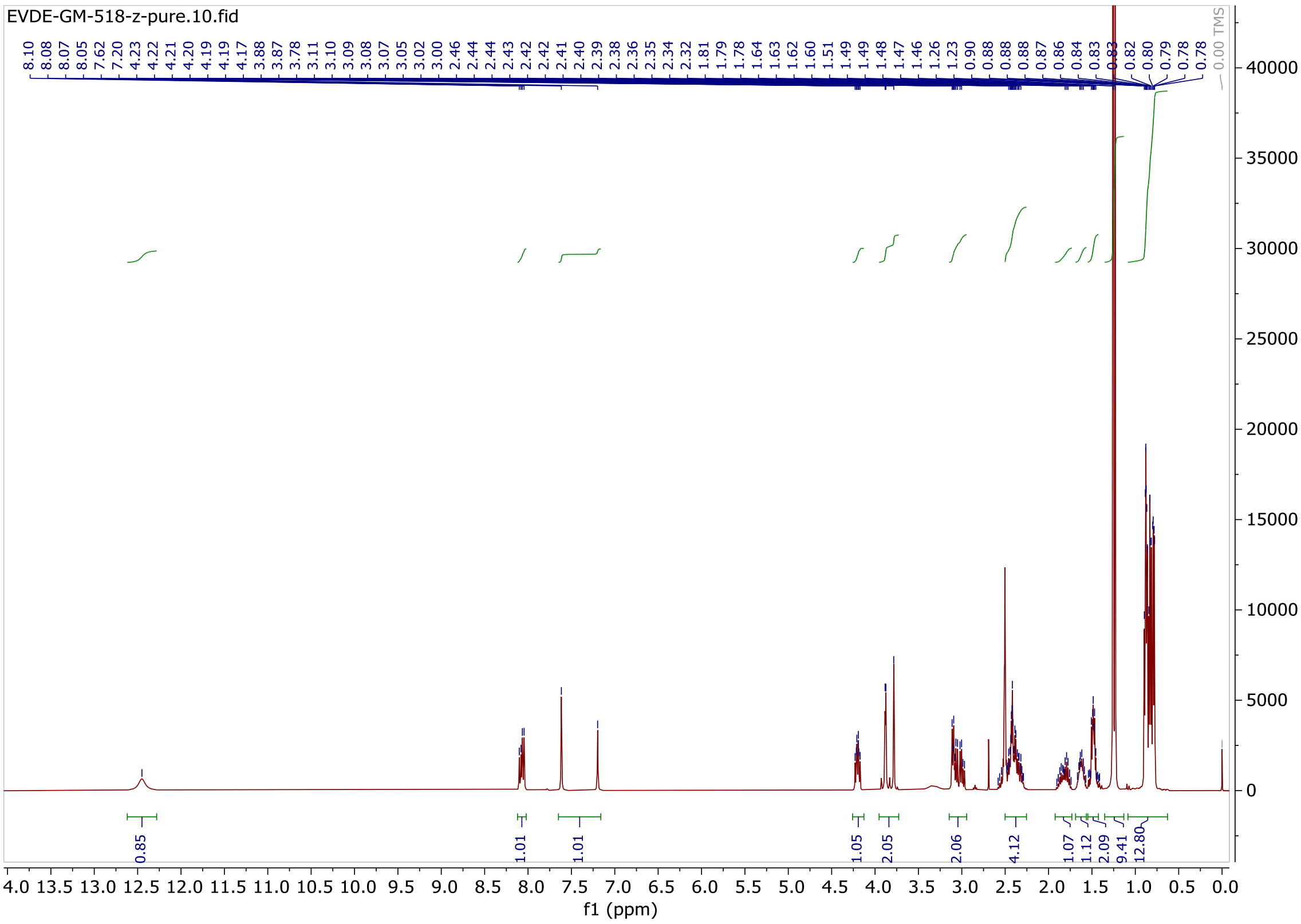

Figure S39. ^{1}H-NMR spectrum of compound **18**.

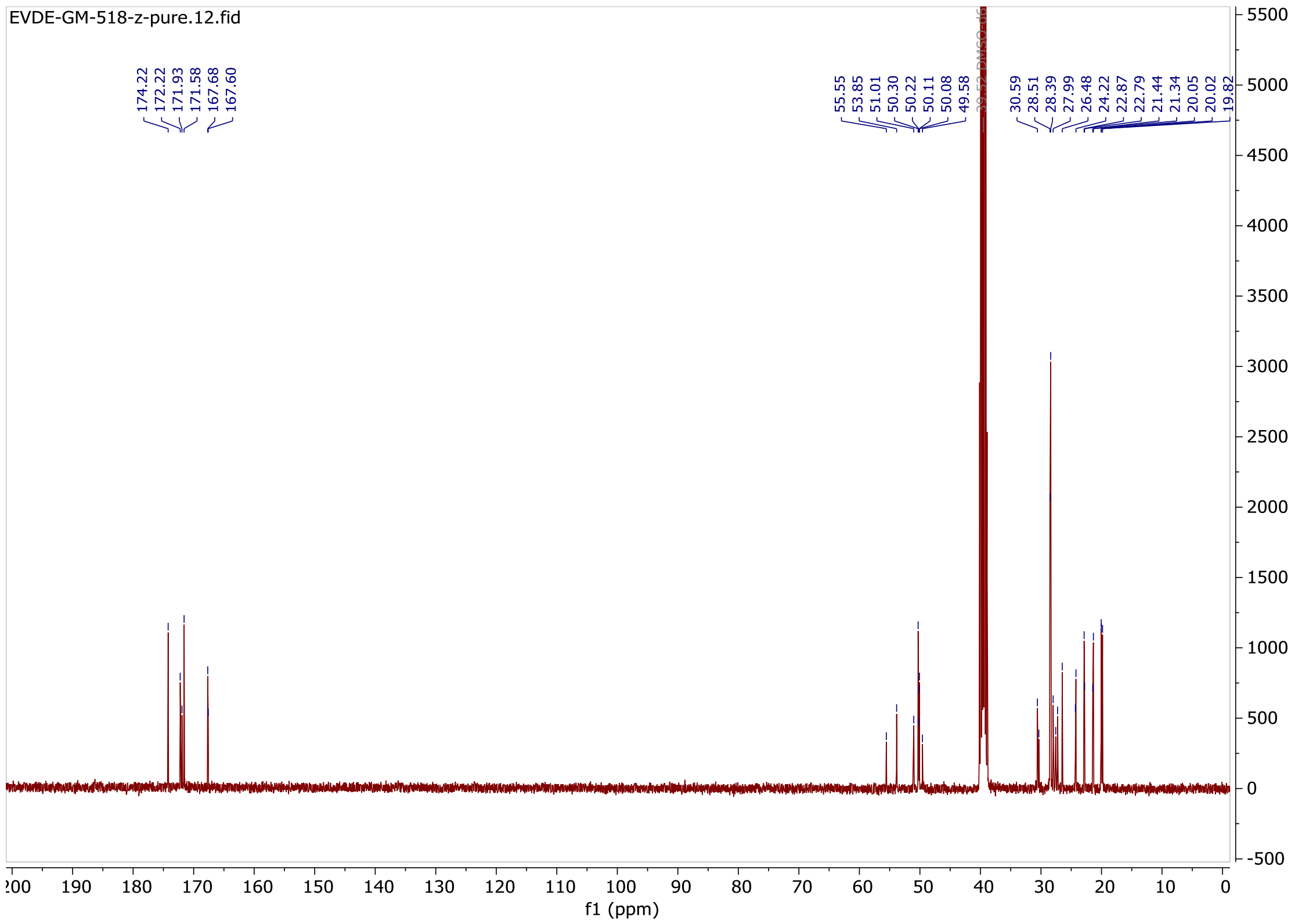

Figure S40. ^{13}C-NMR spectrum of compound **18**.

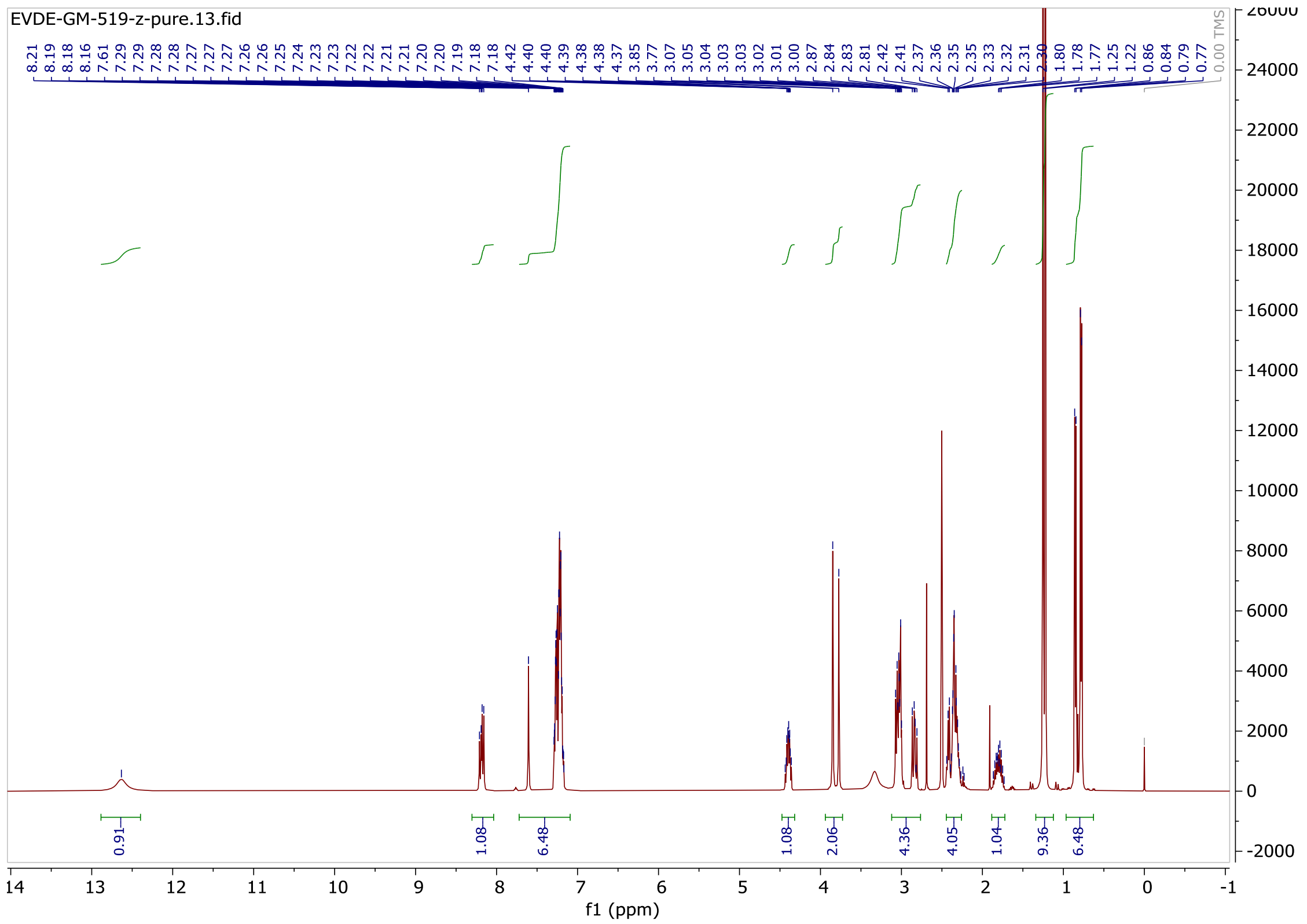

Figure S41. ^{1}H-NMR spectrum of compound **19**.

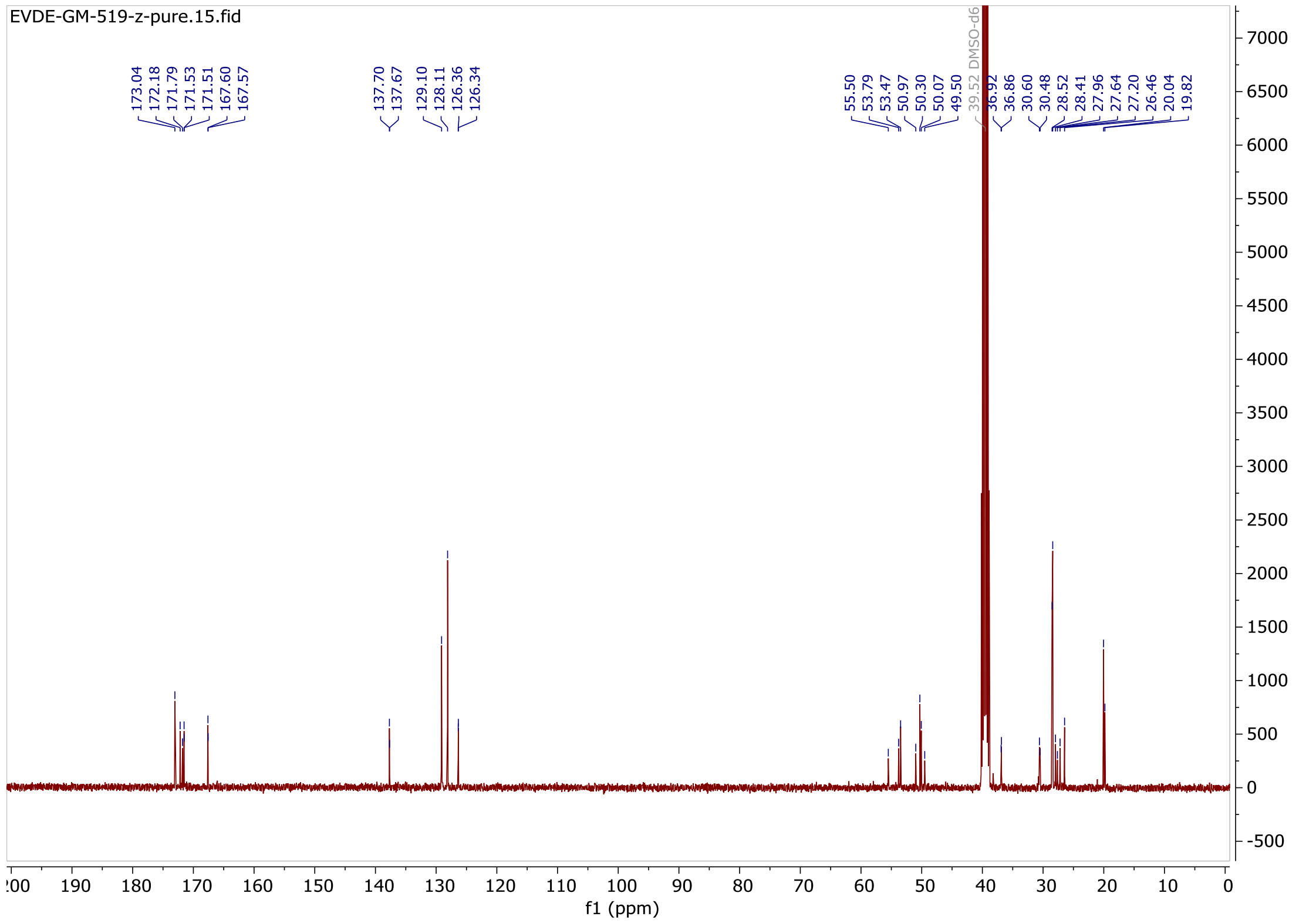

Figure S42. ^{1}H-NMR spectrum of compound **19**.

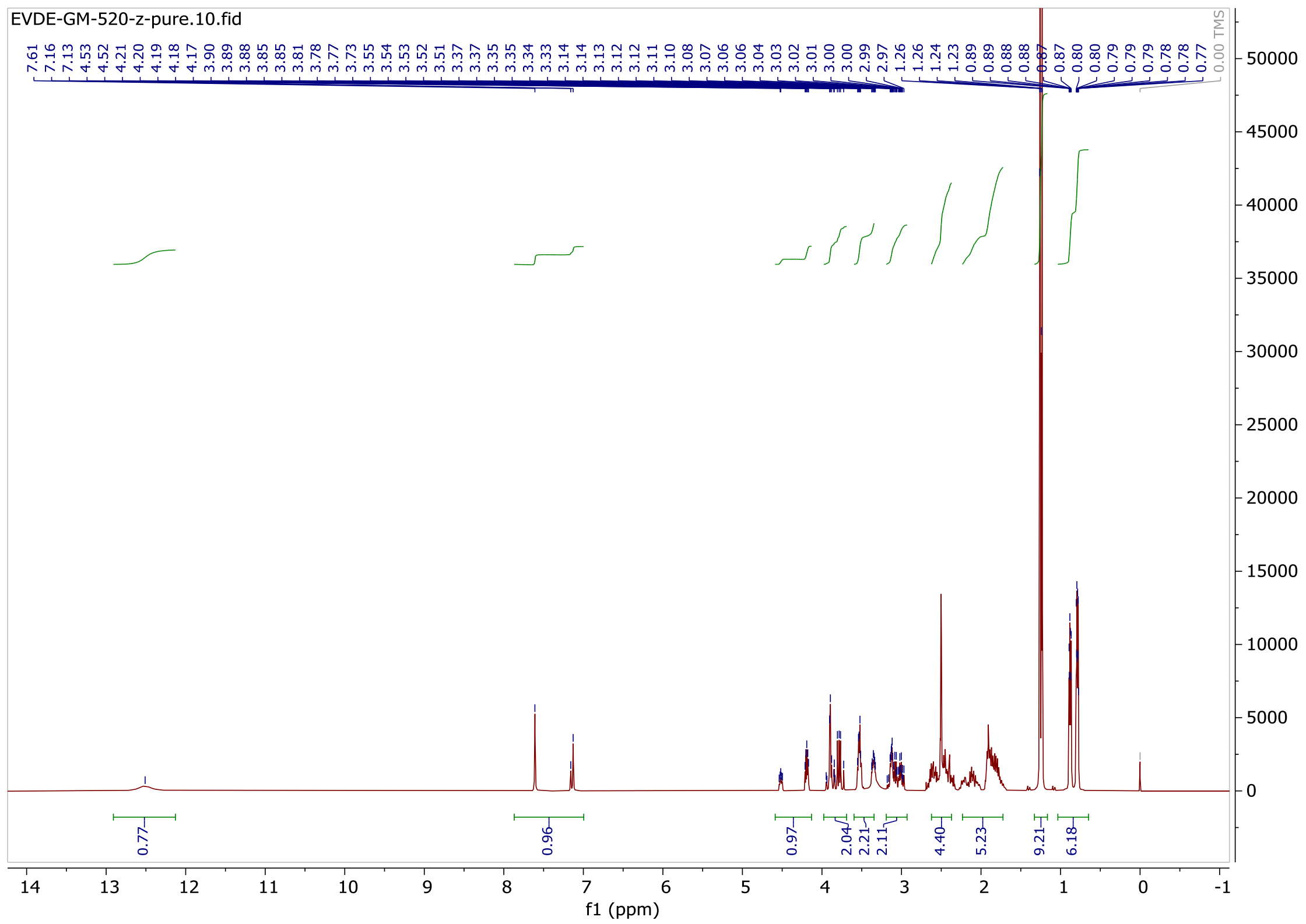

Figure S43. ^{1}H-NMR spectrum of compound **20**.

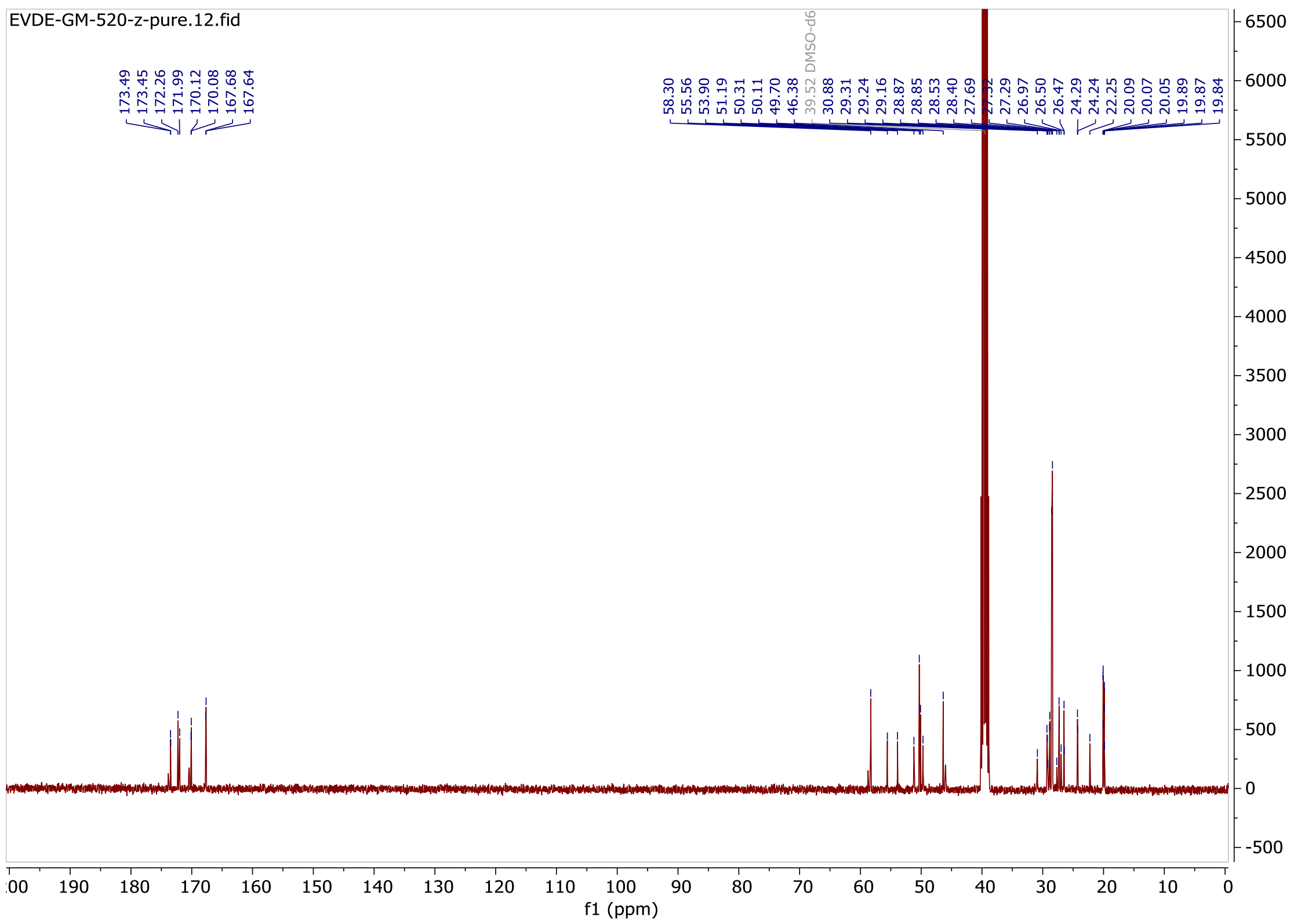

Figure S44. ^{13}C-NMR spectrum of compound **20**.

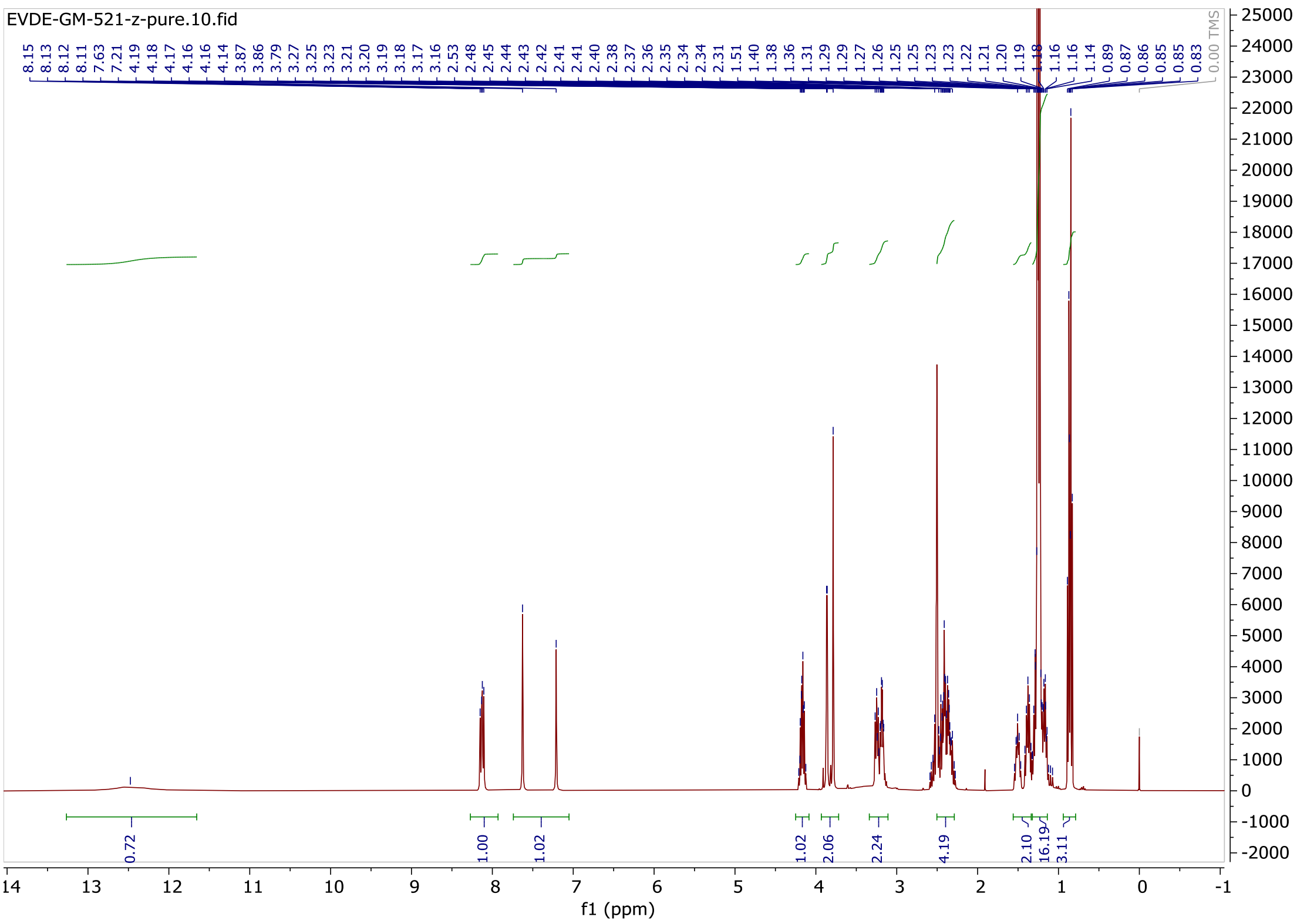

Figure S45. ^{1}H-NMR spectrum of compound **21**.

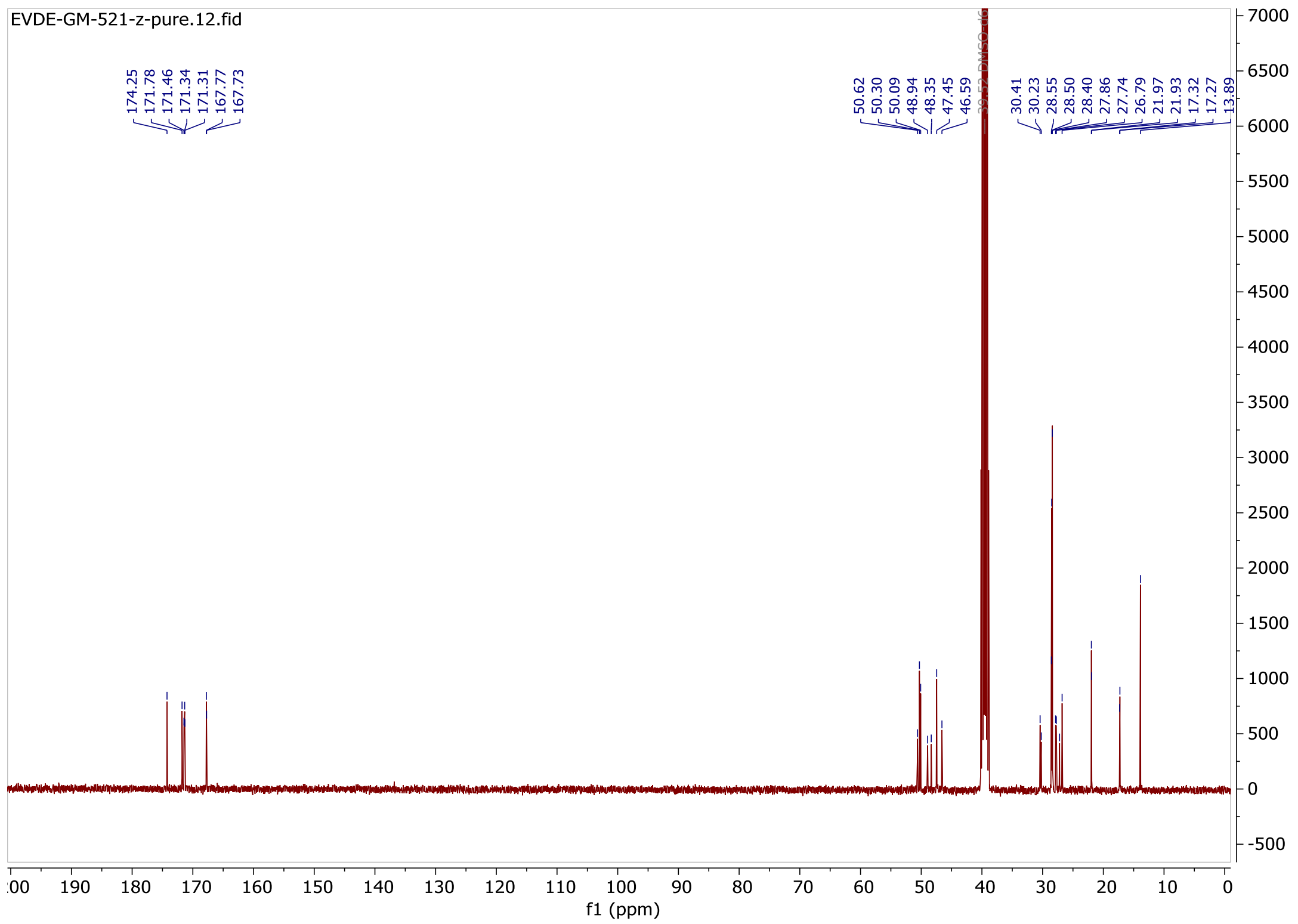

Figure S46. ^{13}C-NMR spectrum of compound **21**

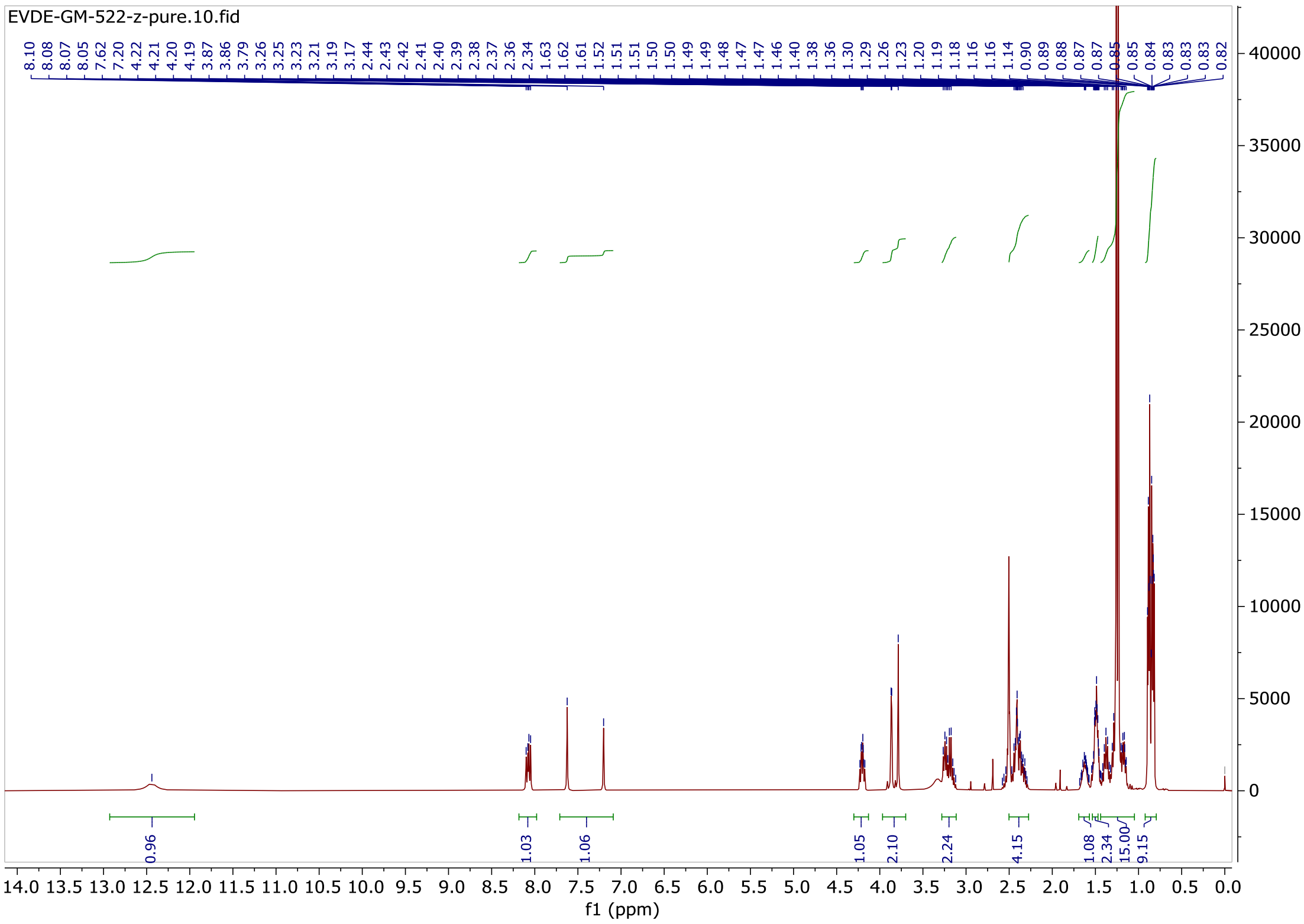

Figure S47. ^{1}H-NMR spectrum of compound **22.**

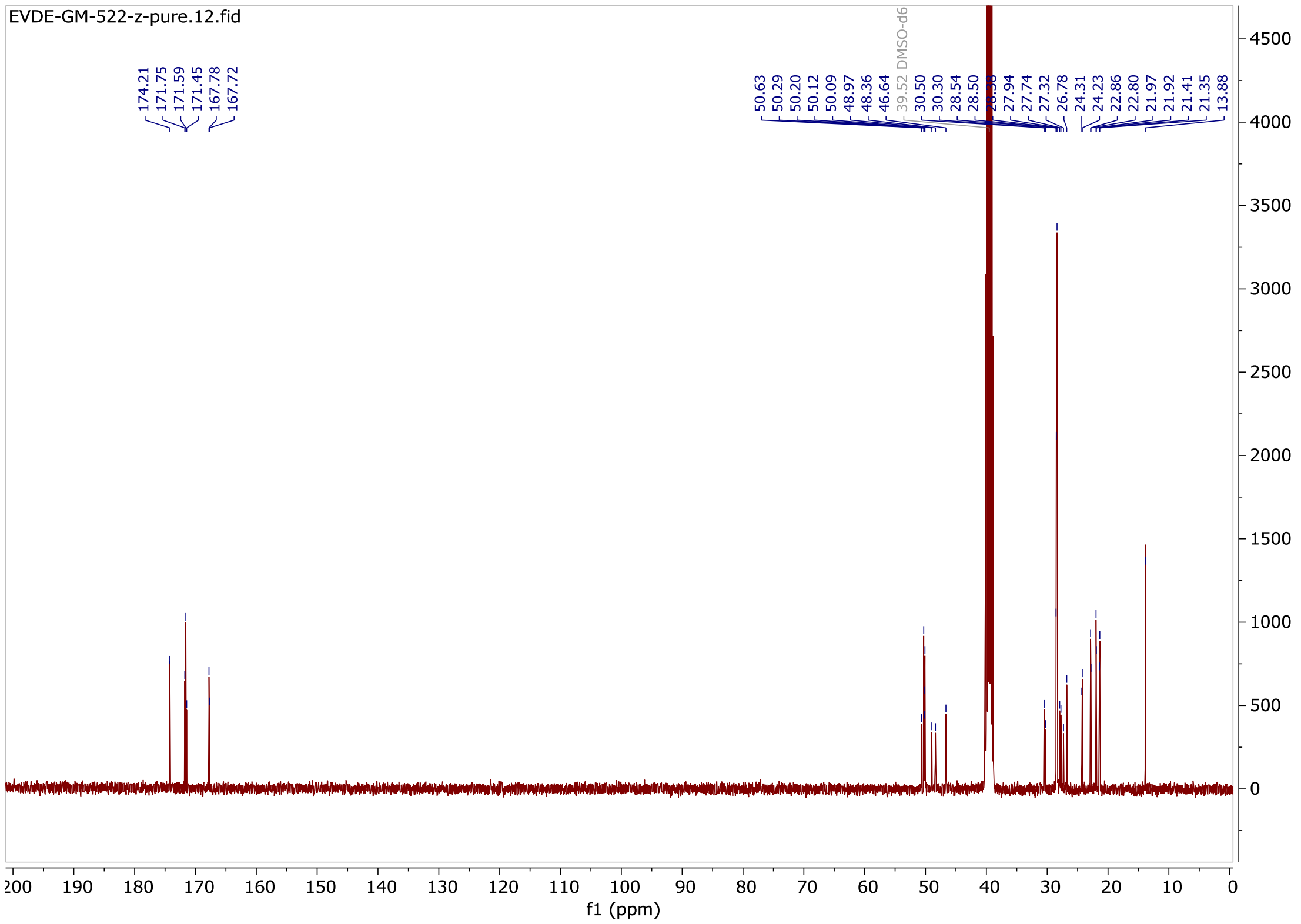

Figure S48. ^{13}C-NMR spectrum of compound **22.**

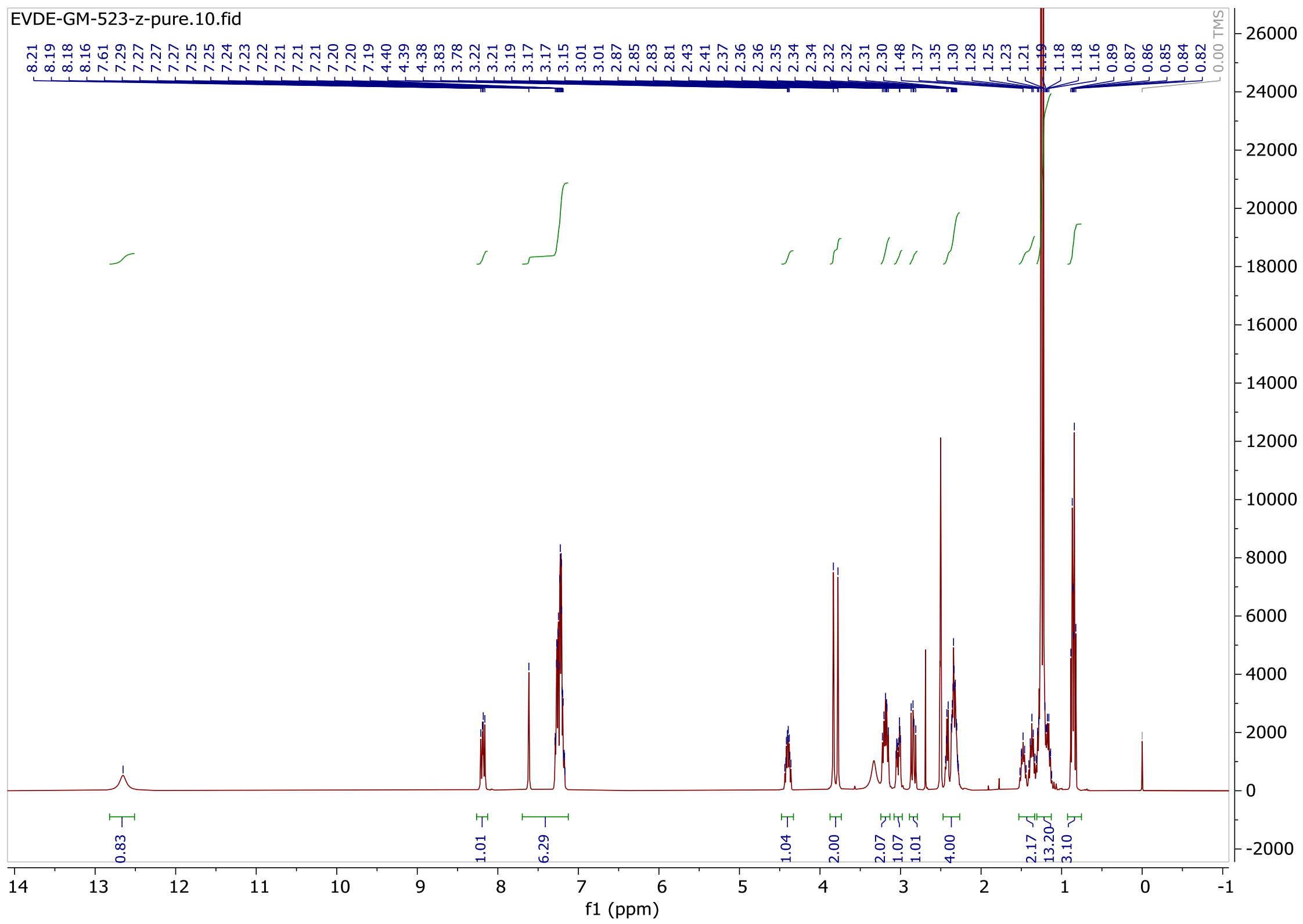

Figure S49. ^{1}H-NMR spectrum of compound **23.**

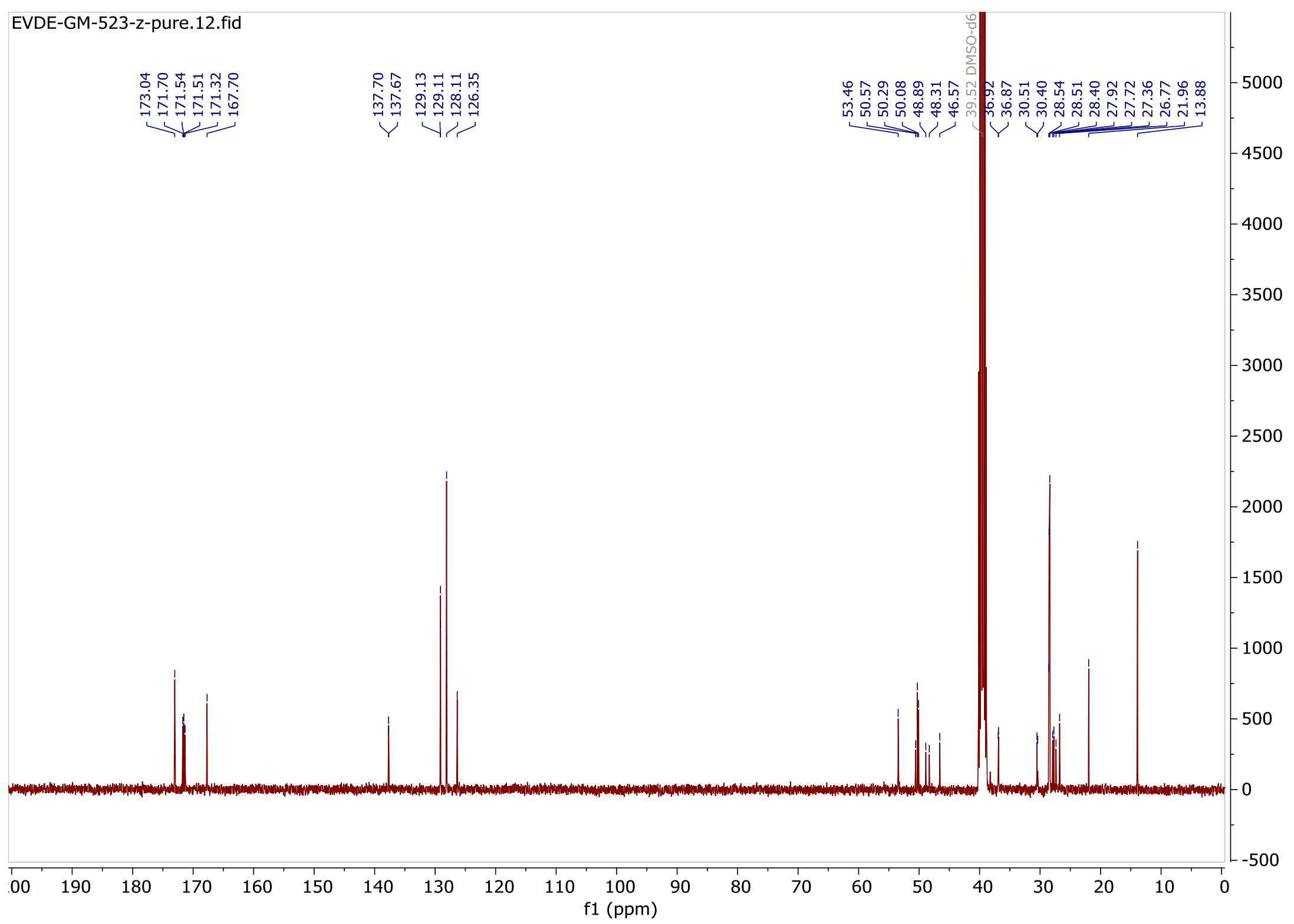

Figure S50. ^{13}C-NMR spectrum of compound **23**.

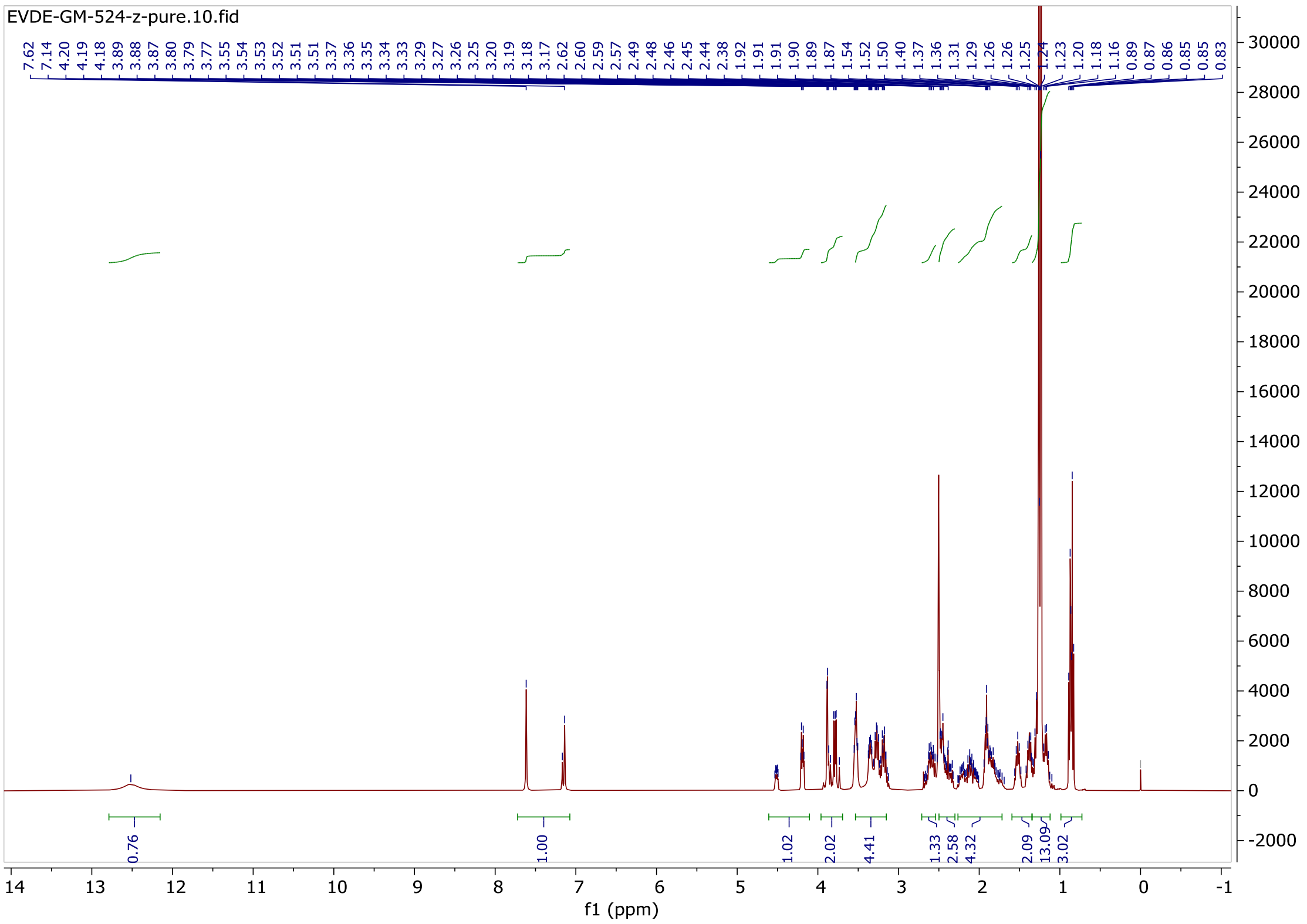

Figure S51. ^{1}H-NMR spectrum of compound **24.**

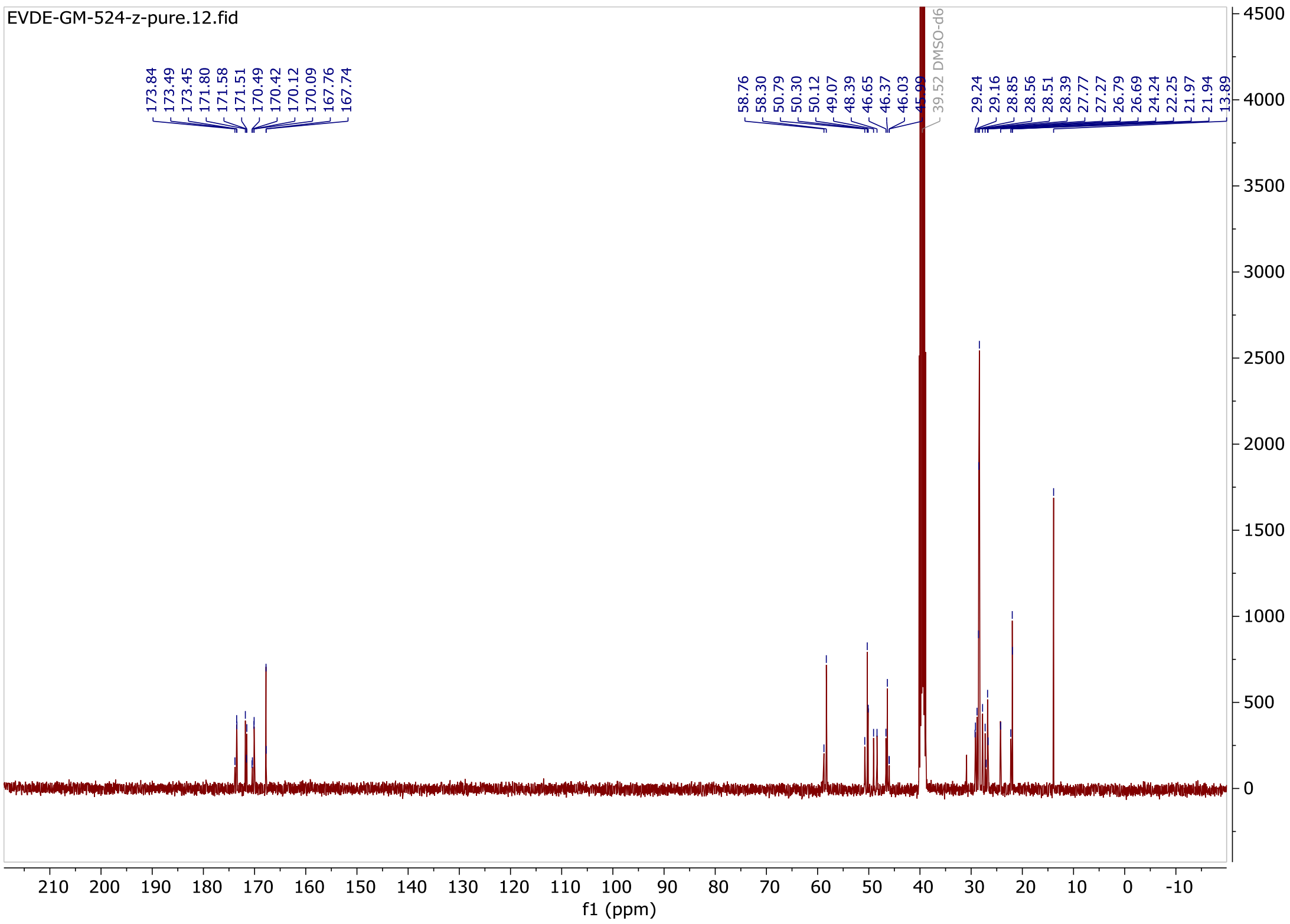

Figure S52. ^{13}C-NMR spectrum of compound **24.**

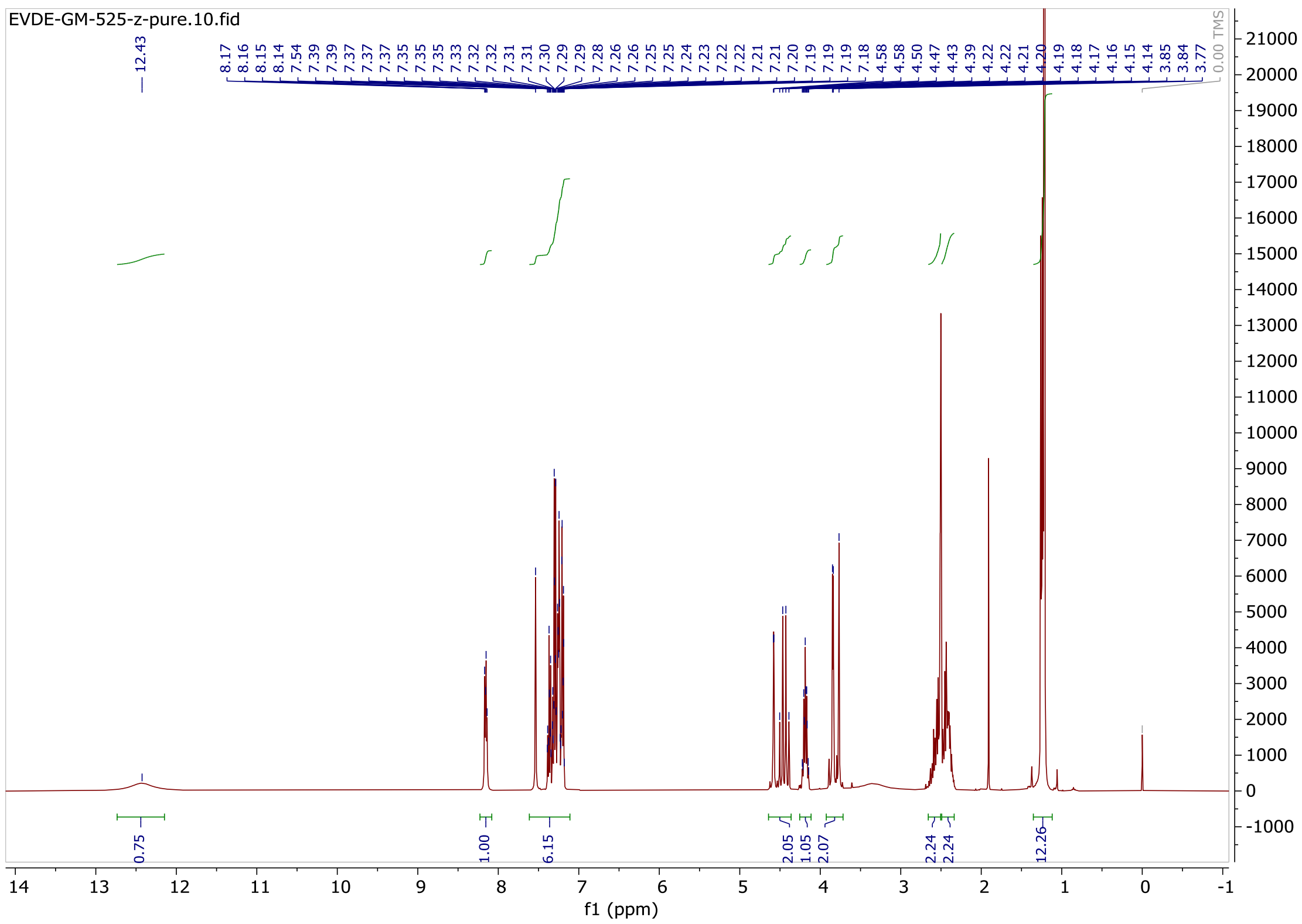

Figure S53. ^{1}H-NMR spectrum of compound **25.**

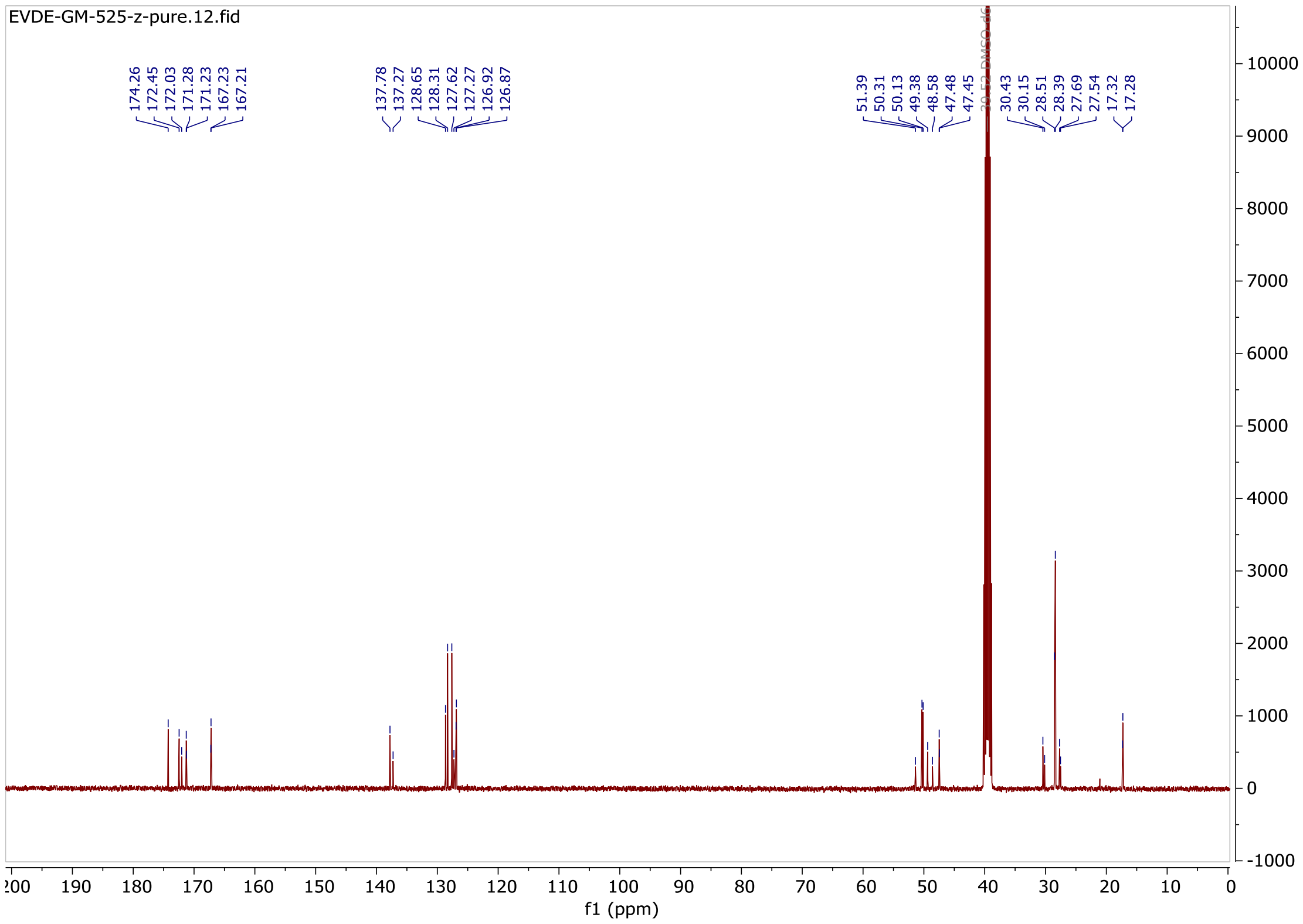

Figure S54. ^{13}C-NMR spectrum of compound **25.**

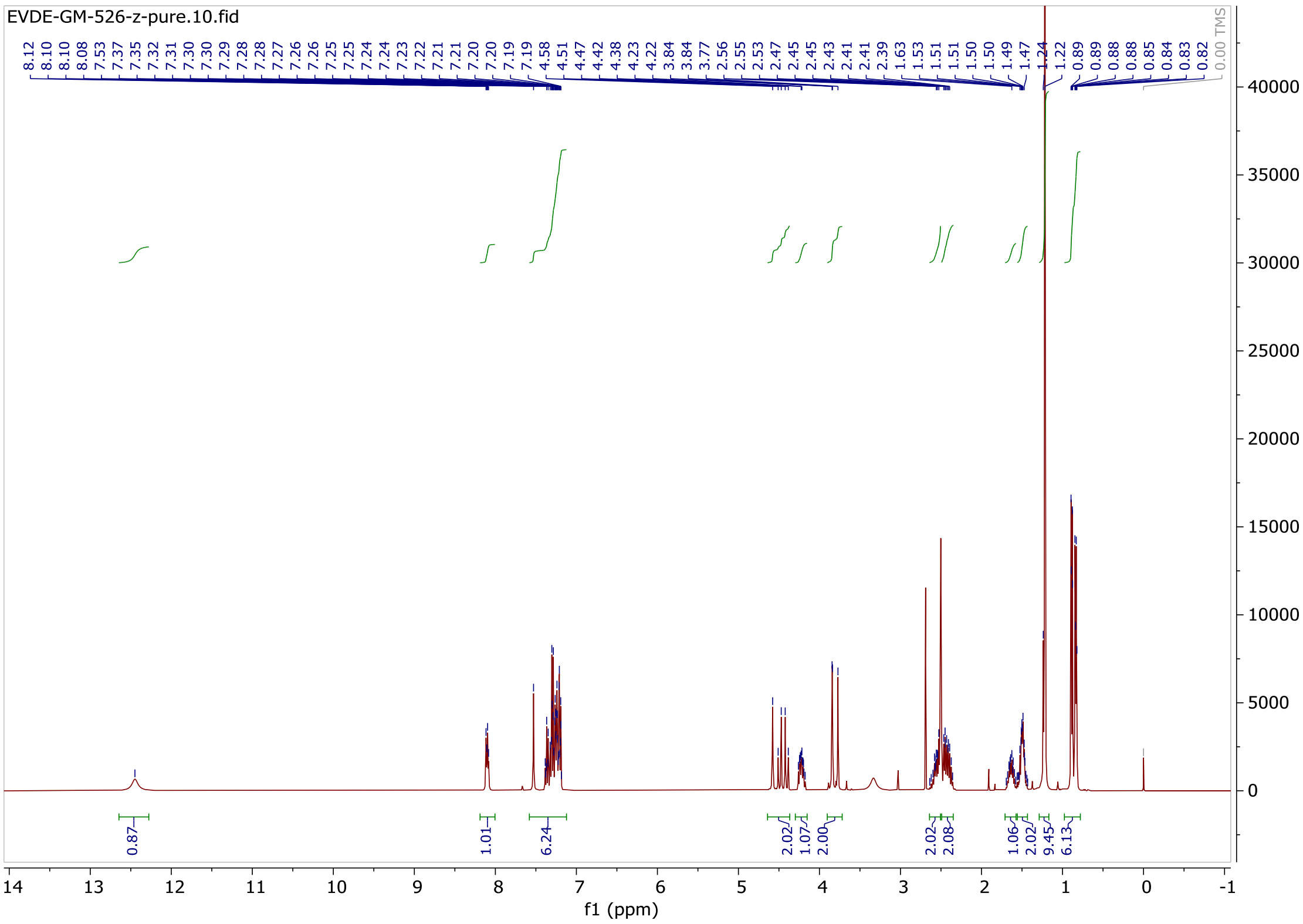

Figure S55. ^{1}H-NMR spectrum of compound **26.**

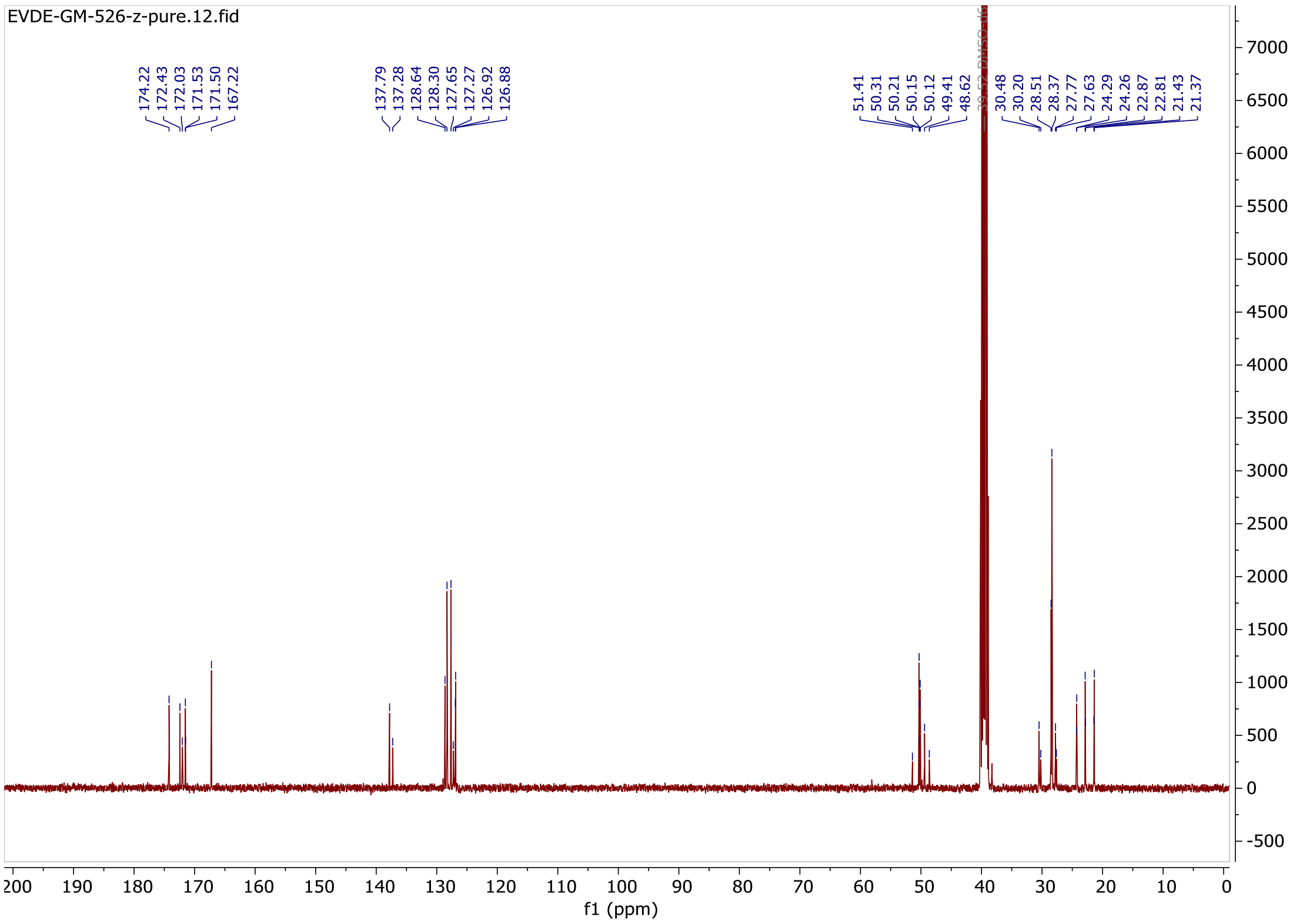

Figure S56. ^{13}C-NMR spectrum of compound **26.**

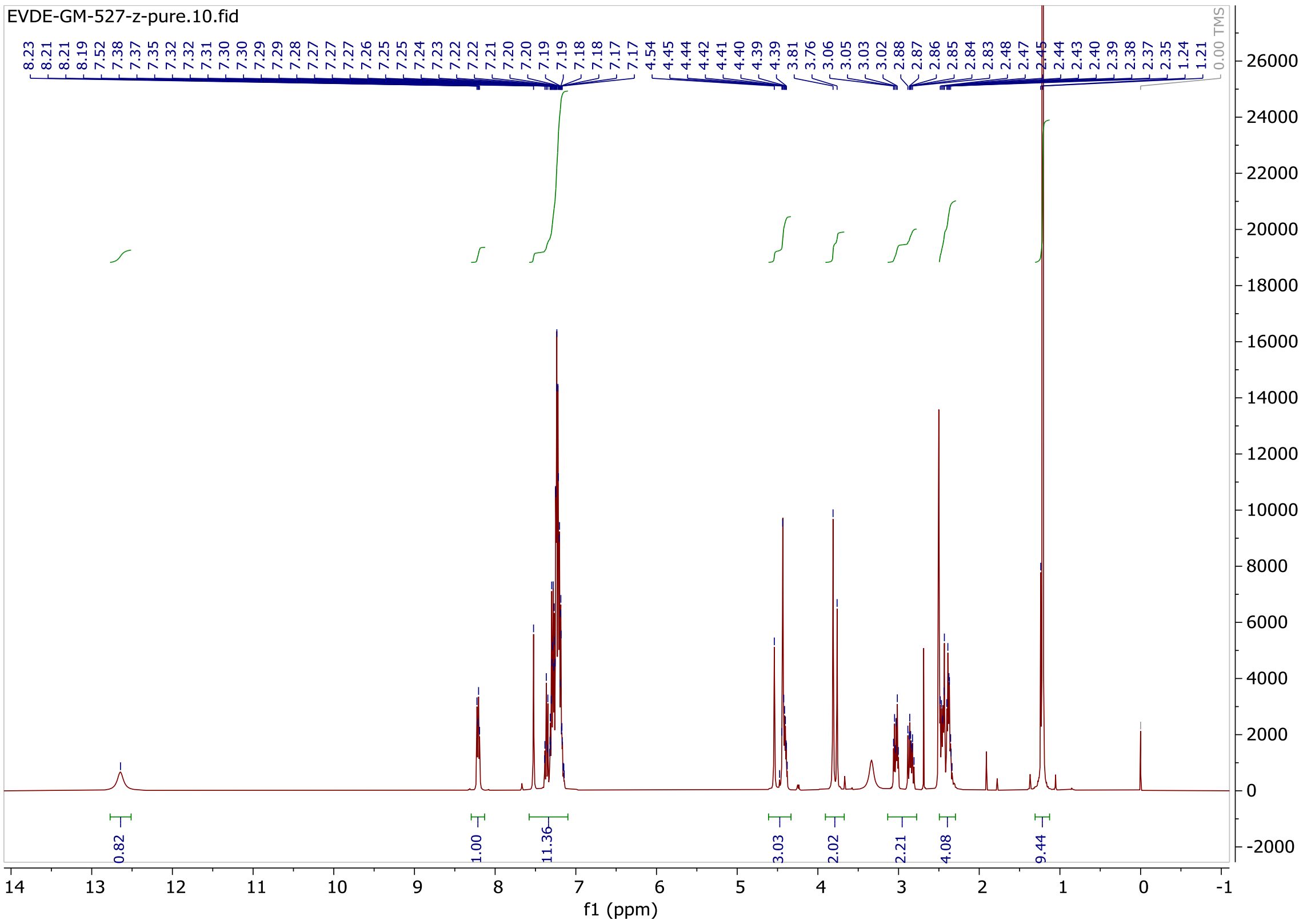

Figure S57. ^{1}H-NMR spectrum of compound **27.**

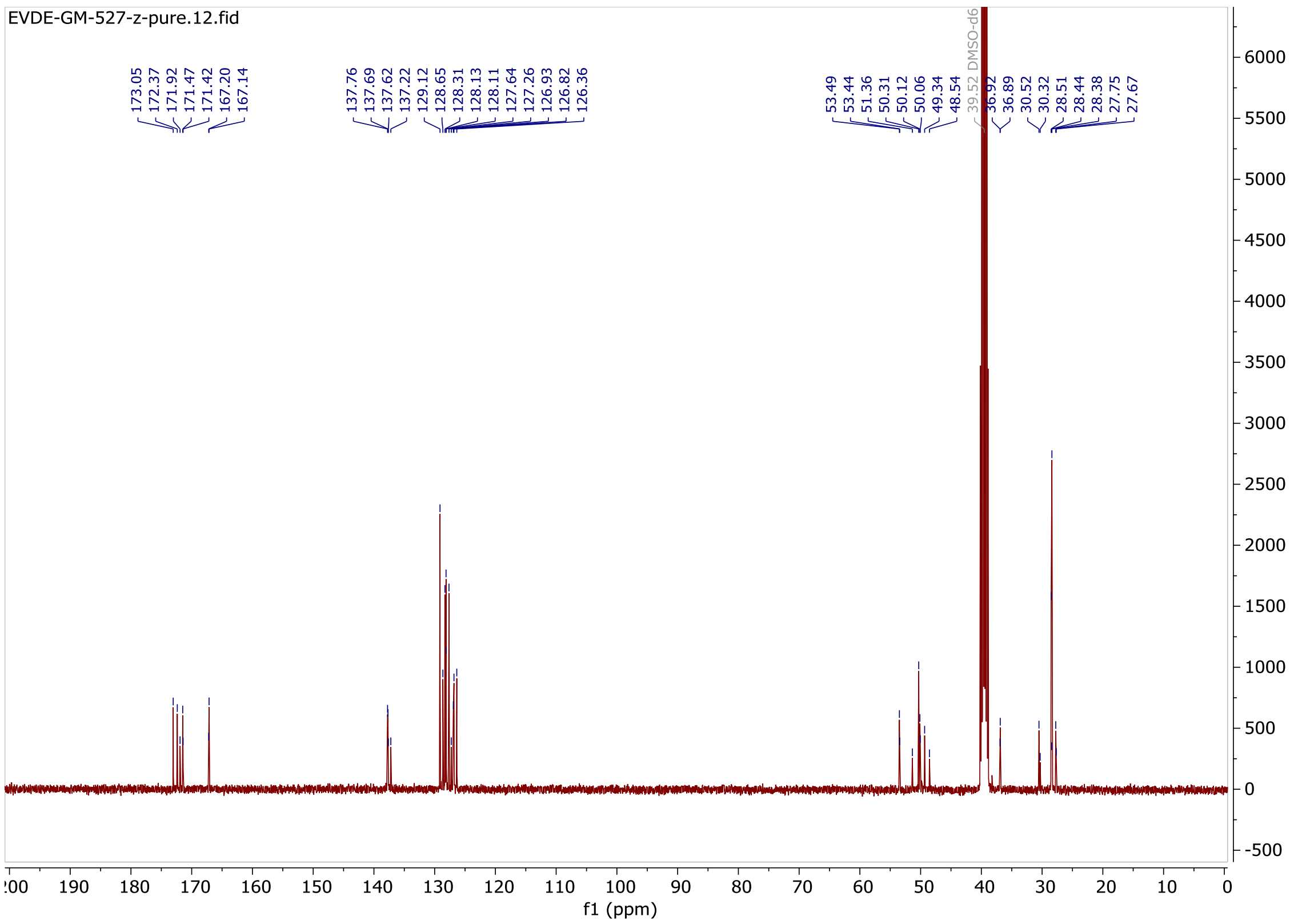

Figure S58. ^{13}C-NMR spectrum of compound **27.**

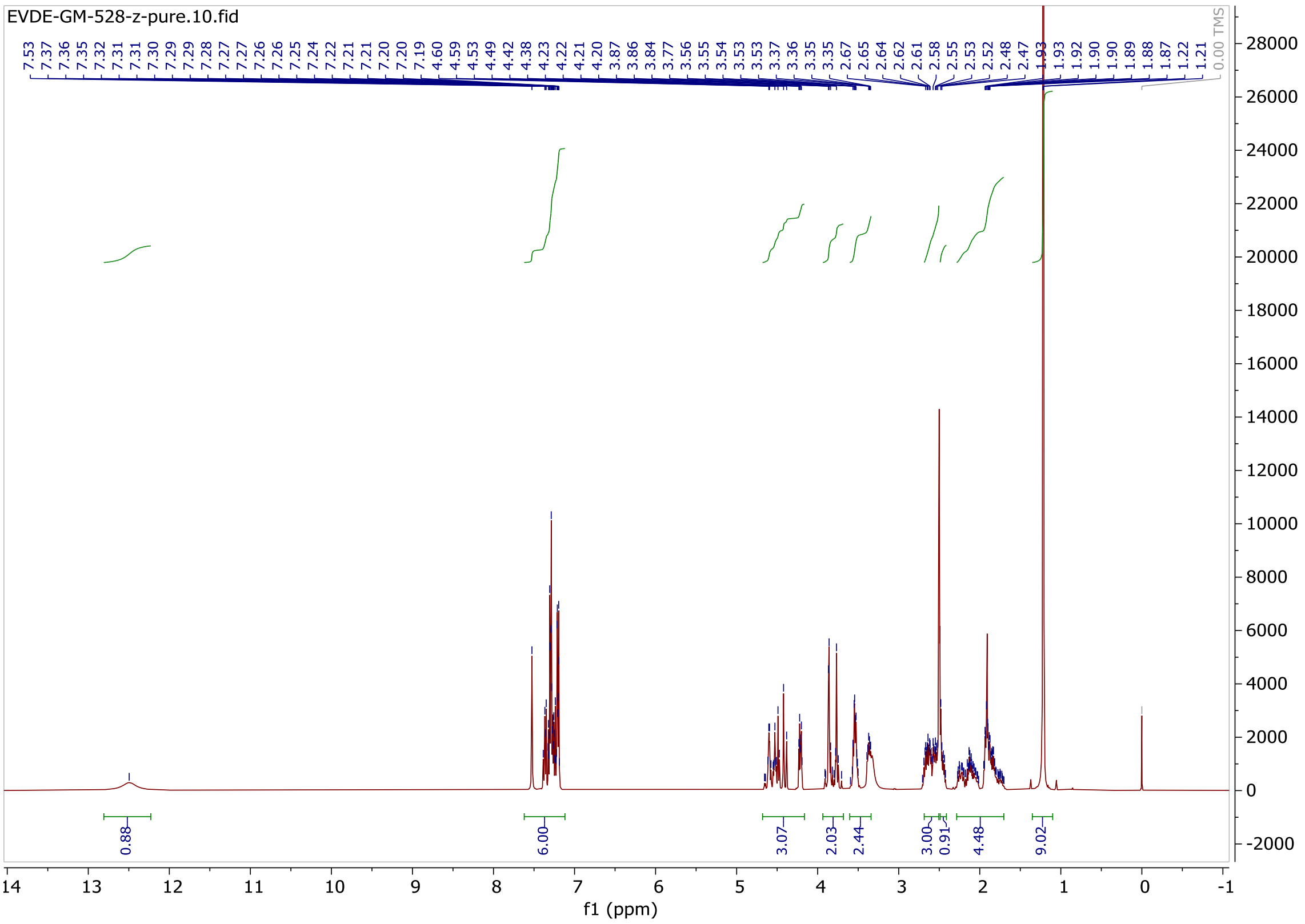

Figure S59. ^{1}H-NMR spectrum of compound **28.**

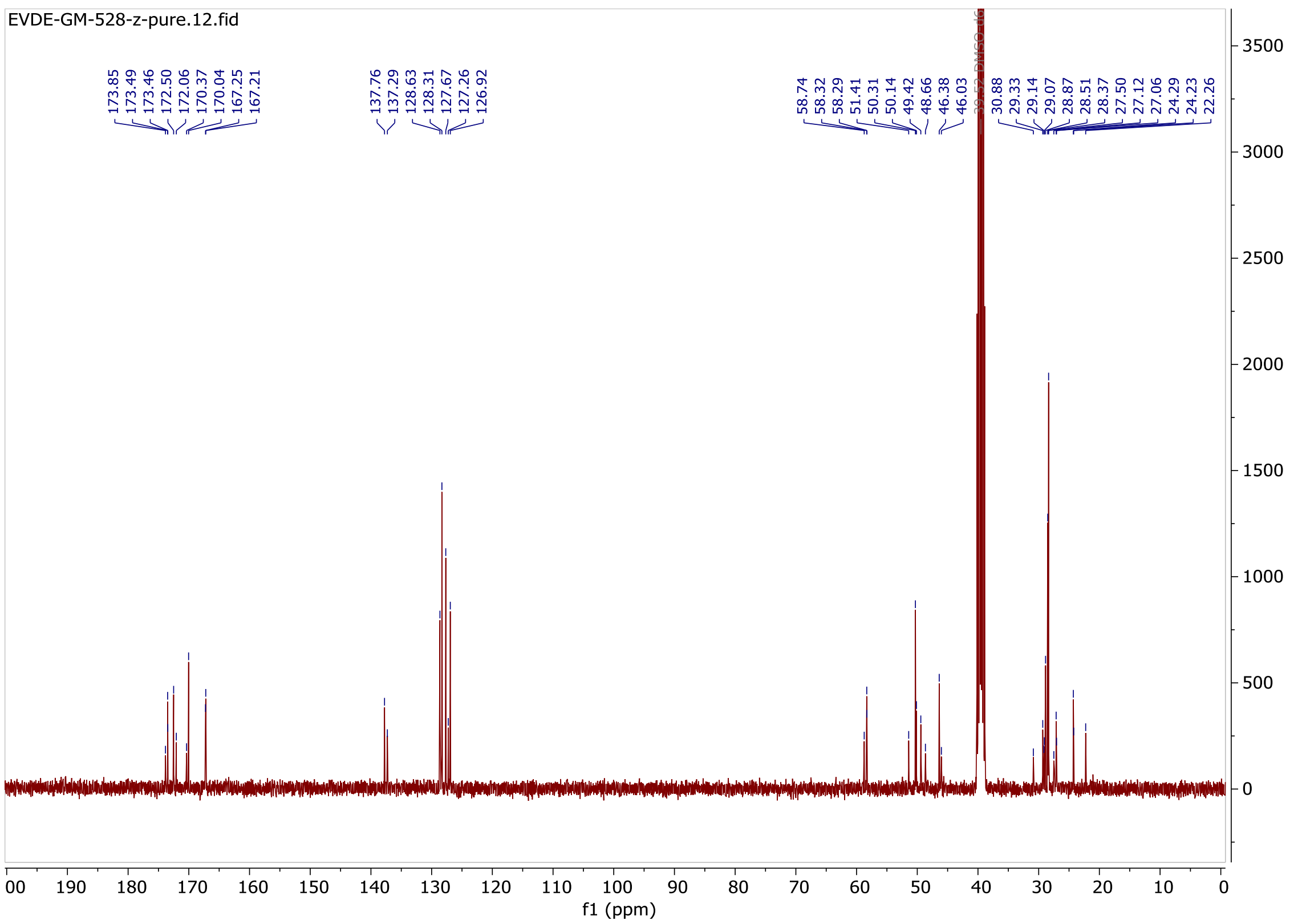

Figure S60. ^{13}C-NMR spectrum of compound **28.**

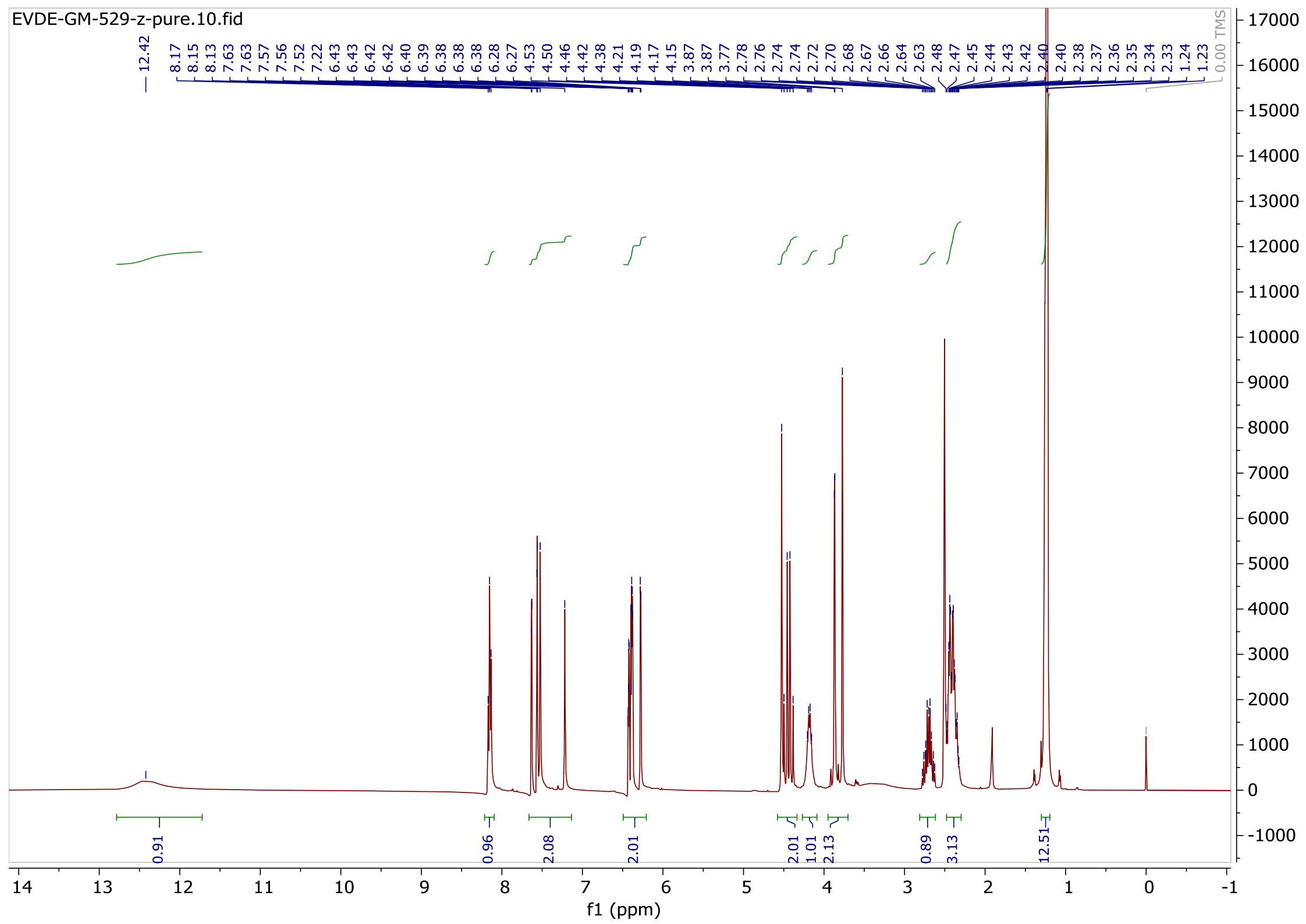

Figure S61. ^{1}H-NMR spectrum of compound **29.**

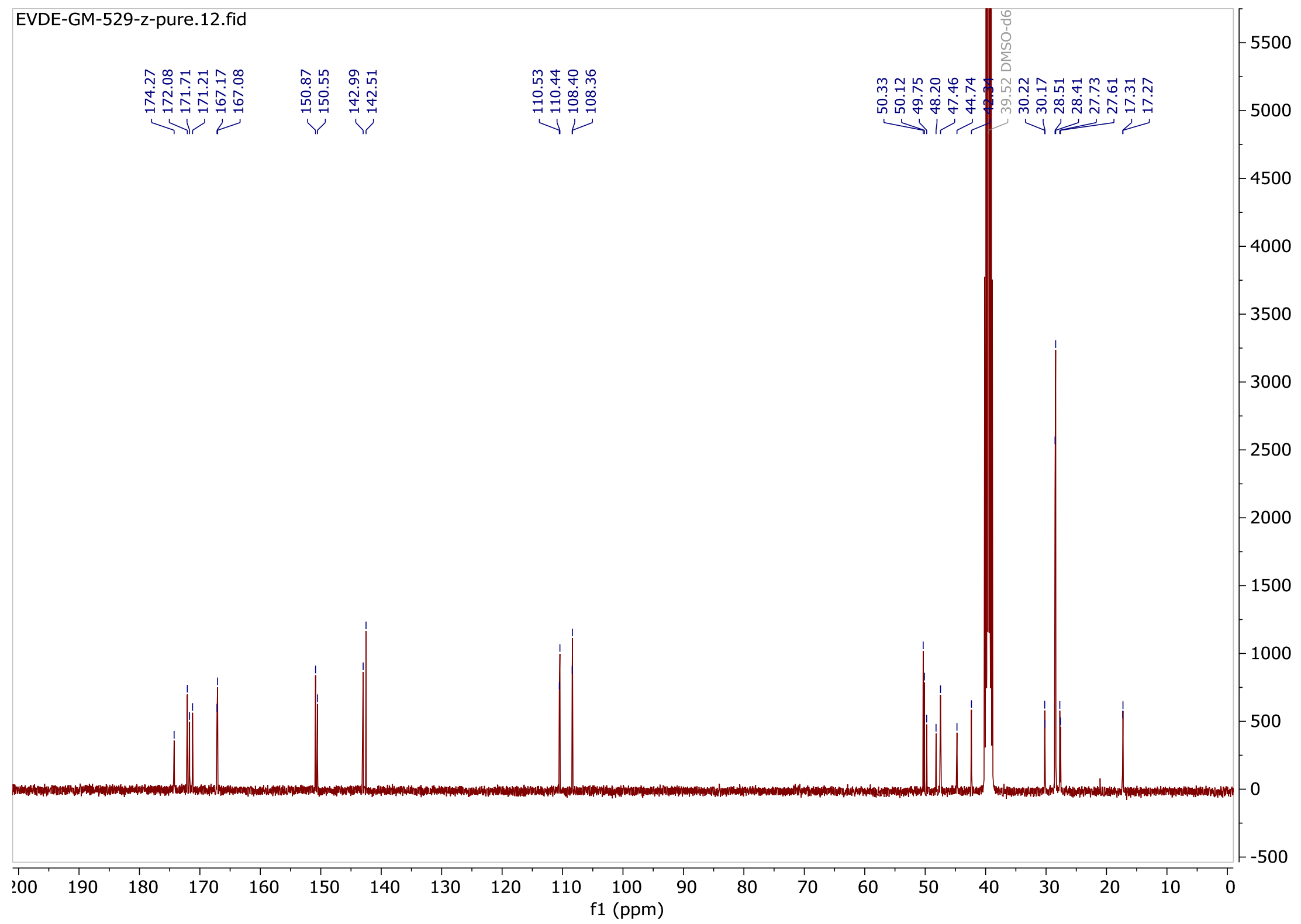

Figure S62. ^{13}C-NMR spectrum of compound **29.**

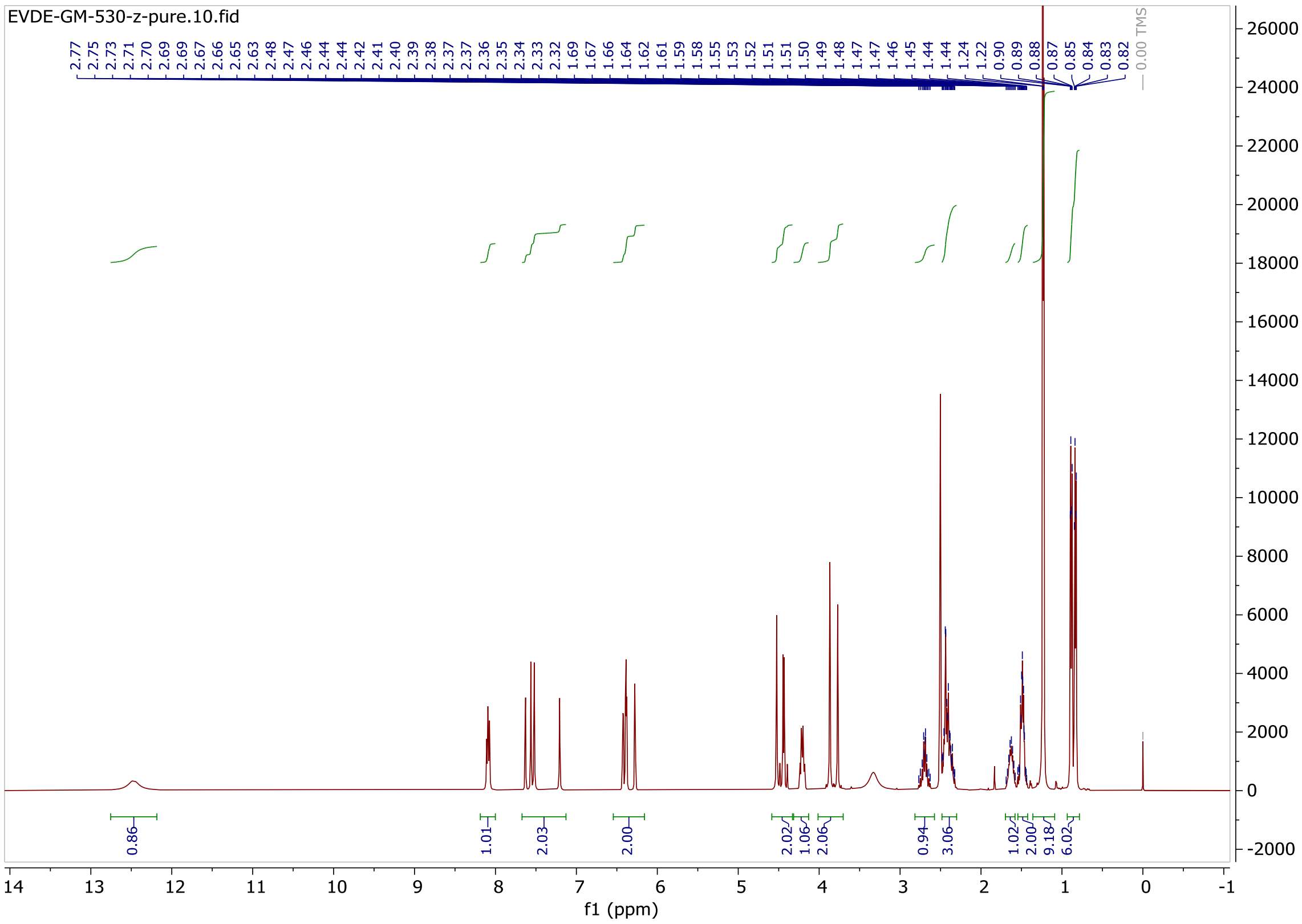

Figure S63. ^{1}H-NMR spectrum of compound **30.**

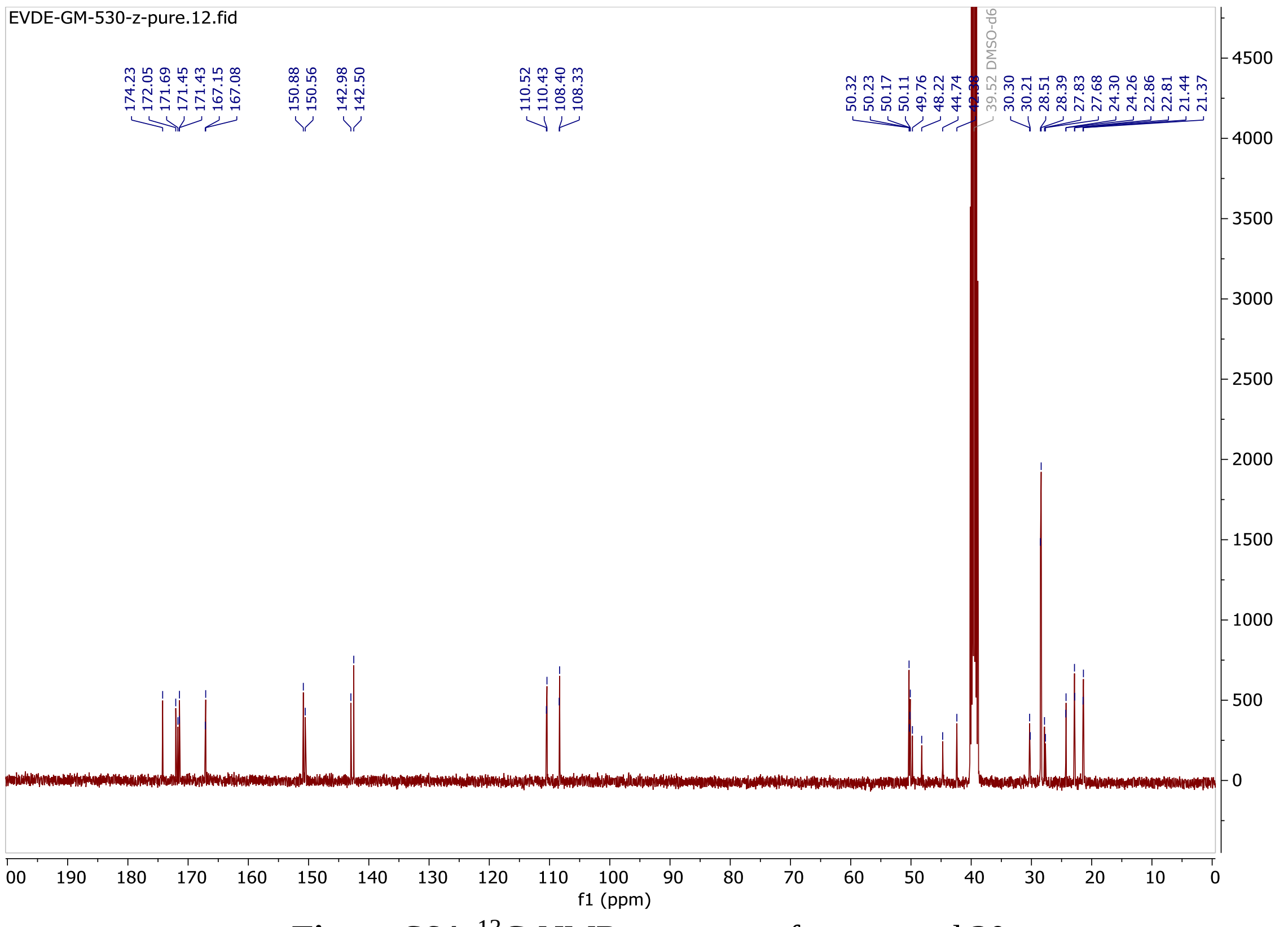

Figure S64. ^{13}C-NMR spectrum of compound **30.**

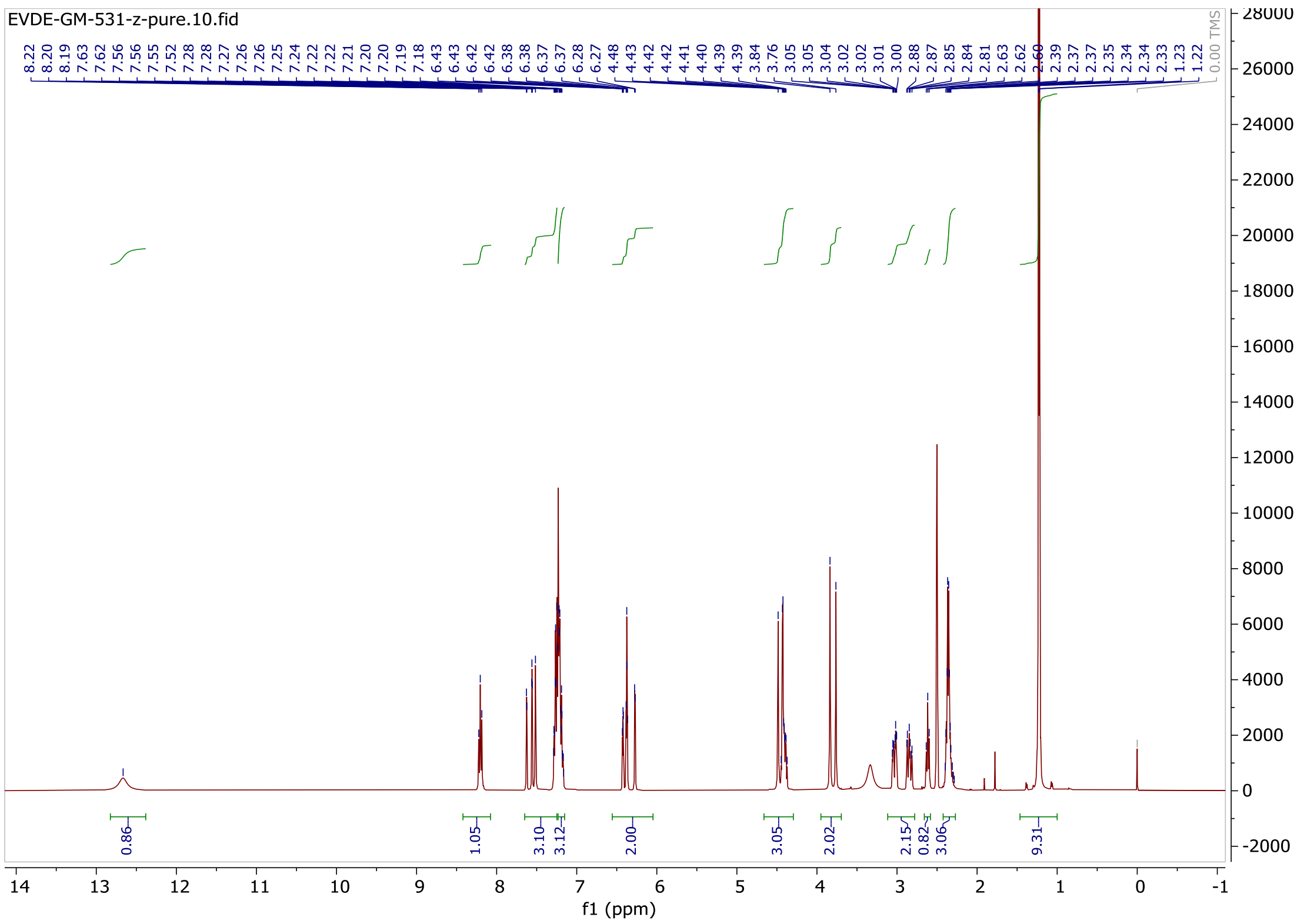

Figure S65. ^{1}H-NMR spectrum of compound **31.**

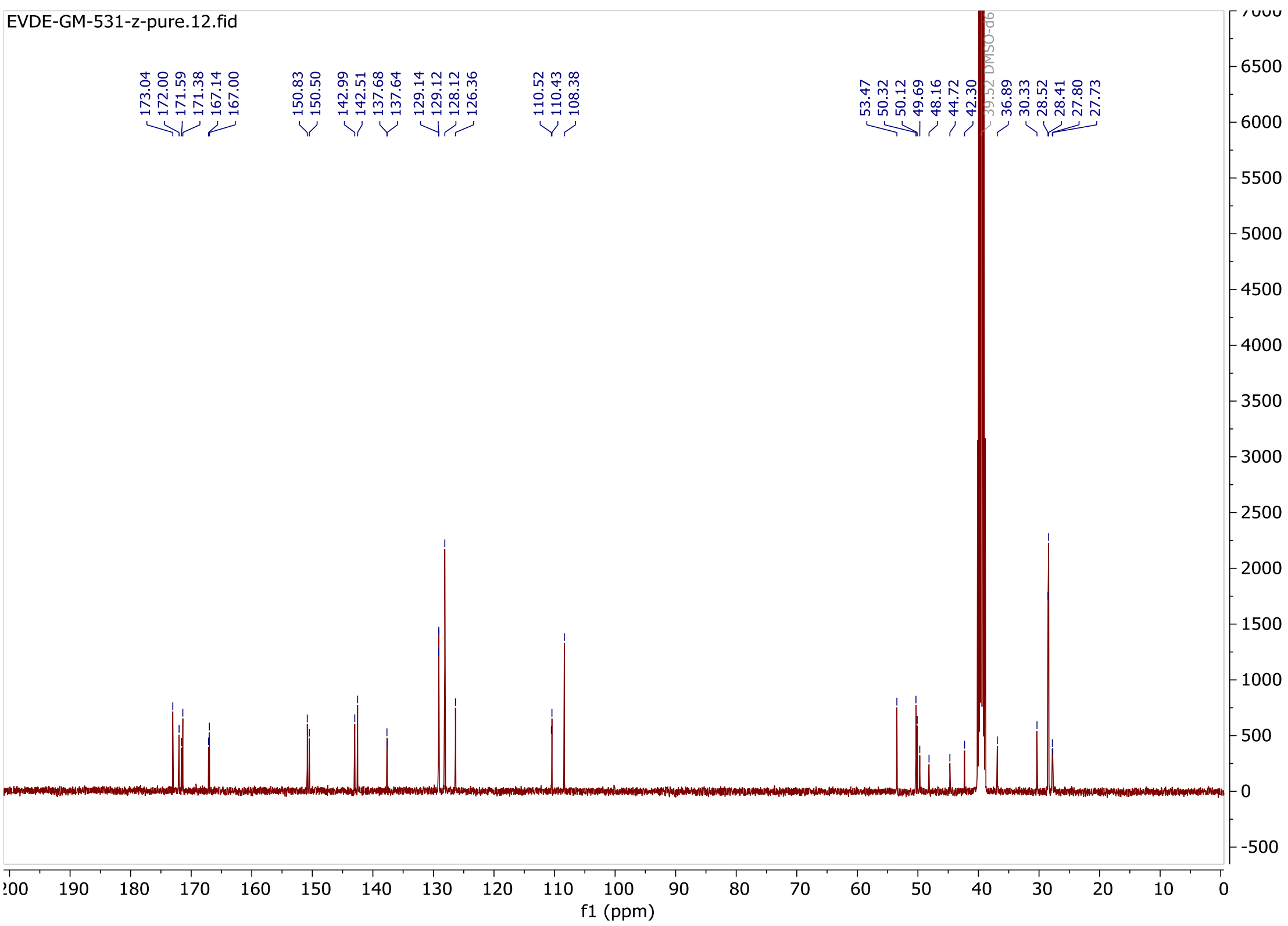

Figure S66. ^{13}C-NMR spectrum of compound **31.**

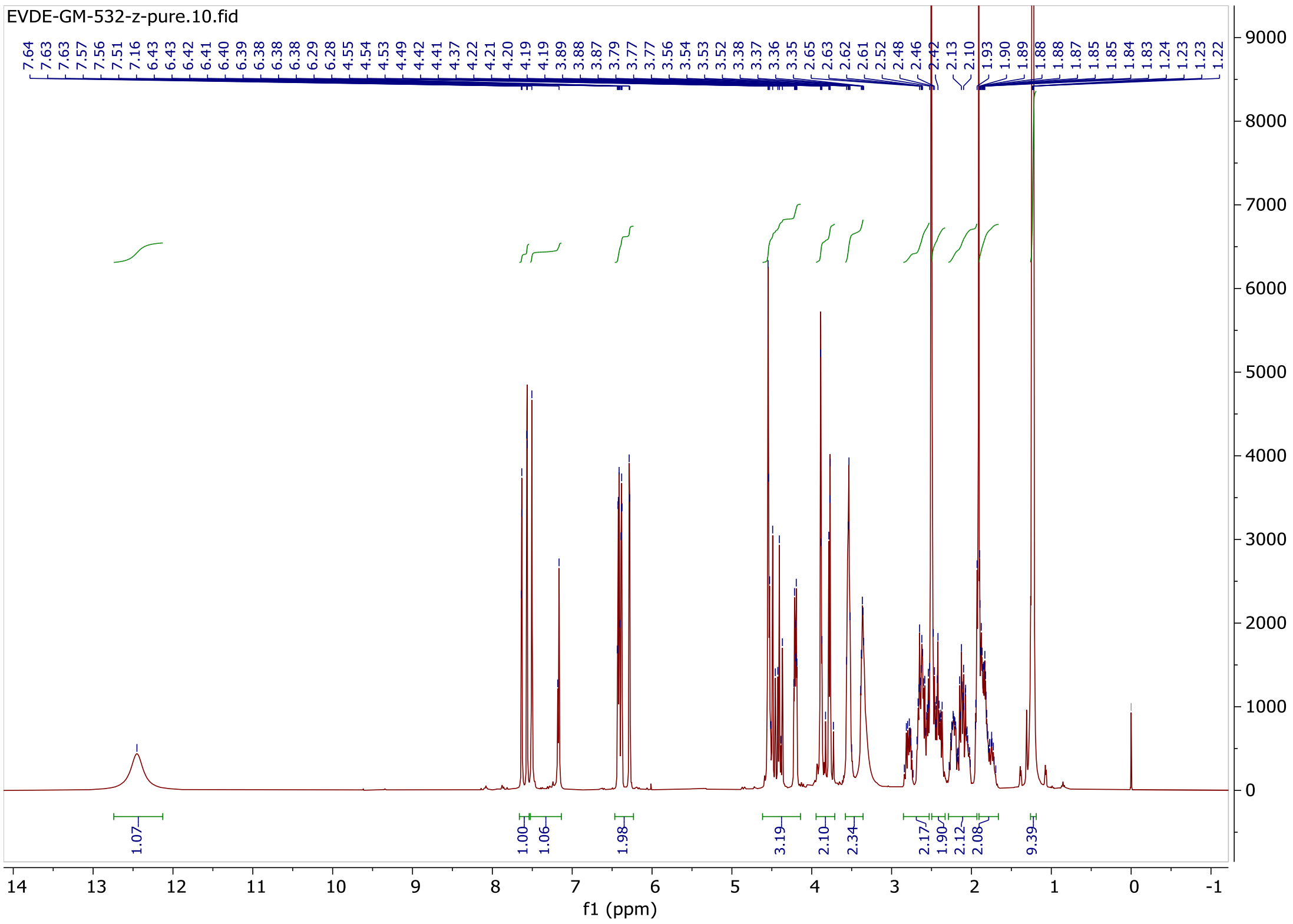

Figure S67. ^{1}H-NMR spectrum of compound **32.**

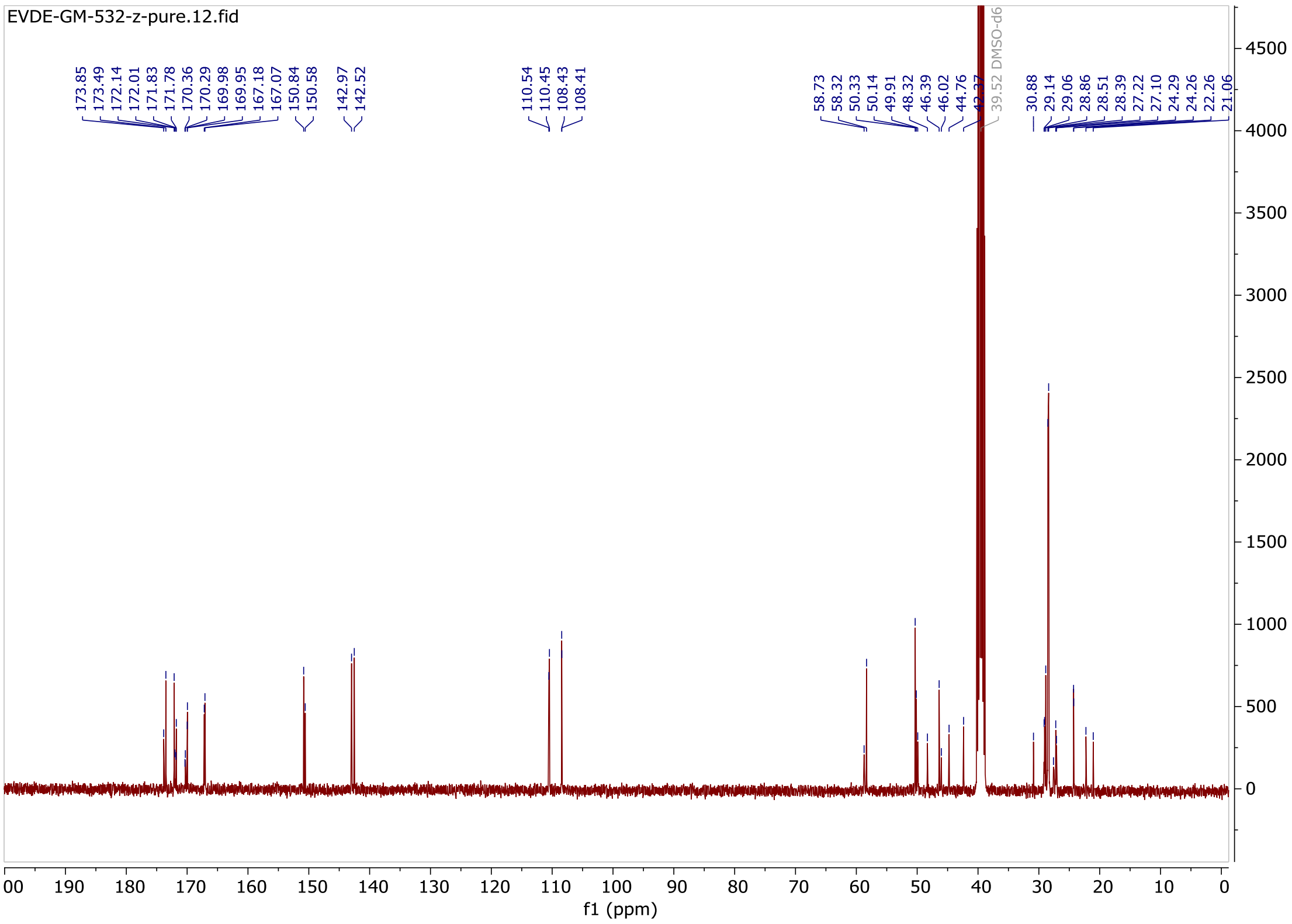

Figure S68. ^{13}C-NMR spectrum of compound **32.**

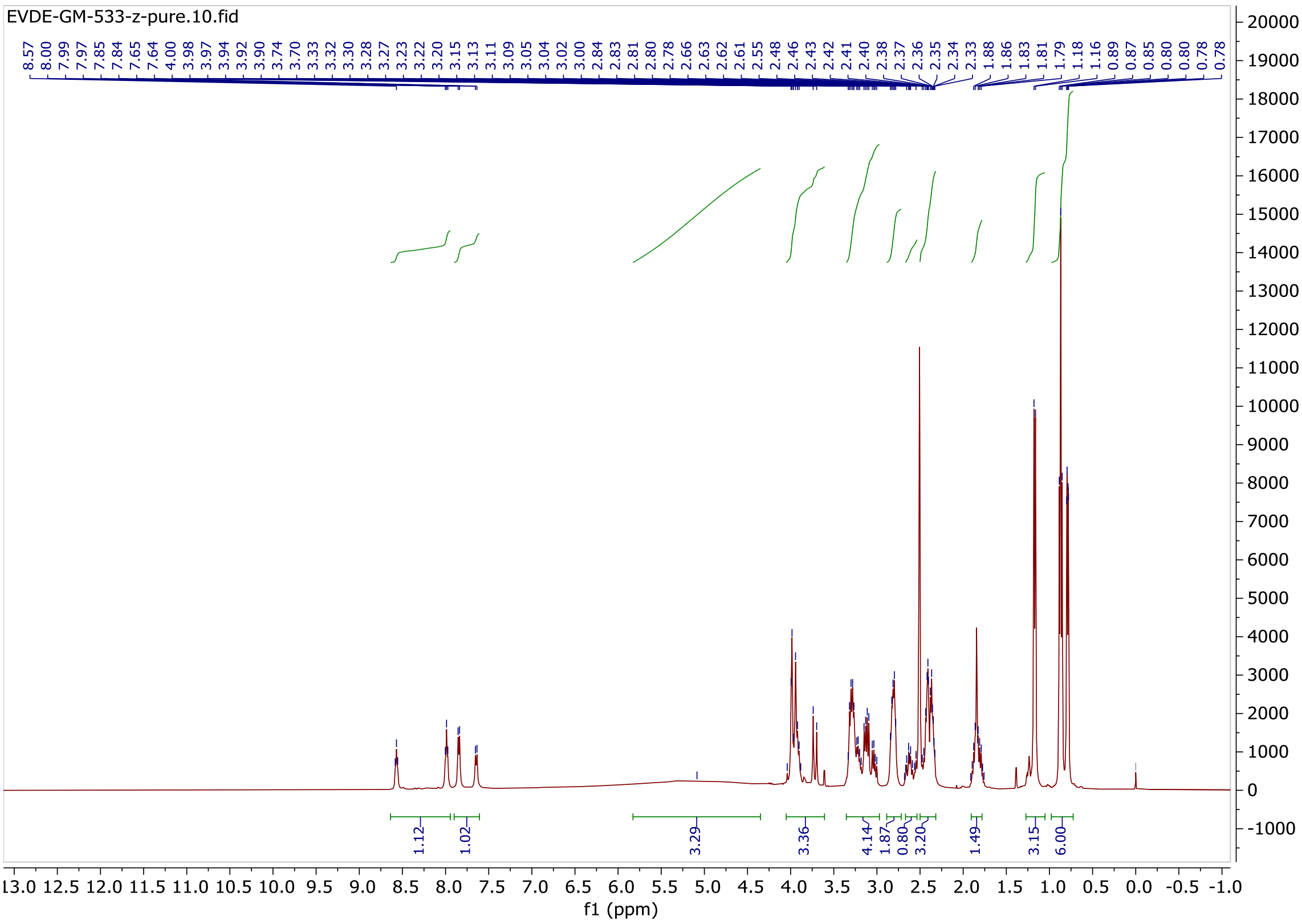

Figure S69. ^{1}H-NMR spectrum of compound **33.**

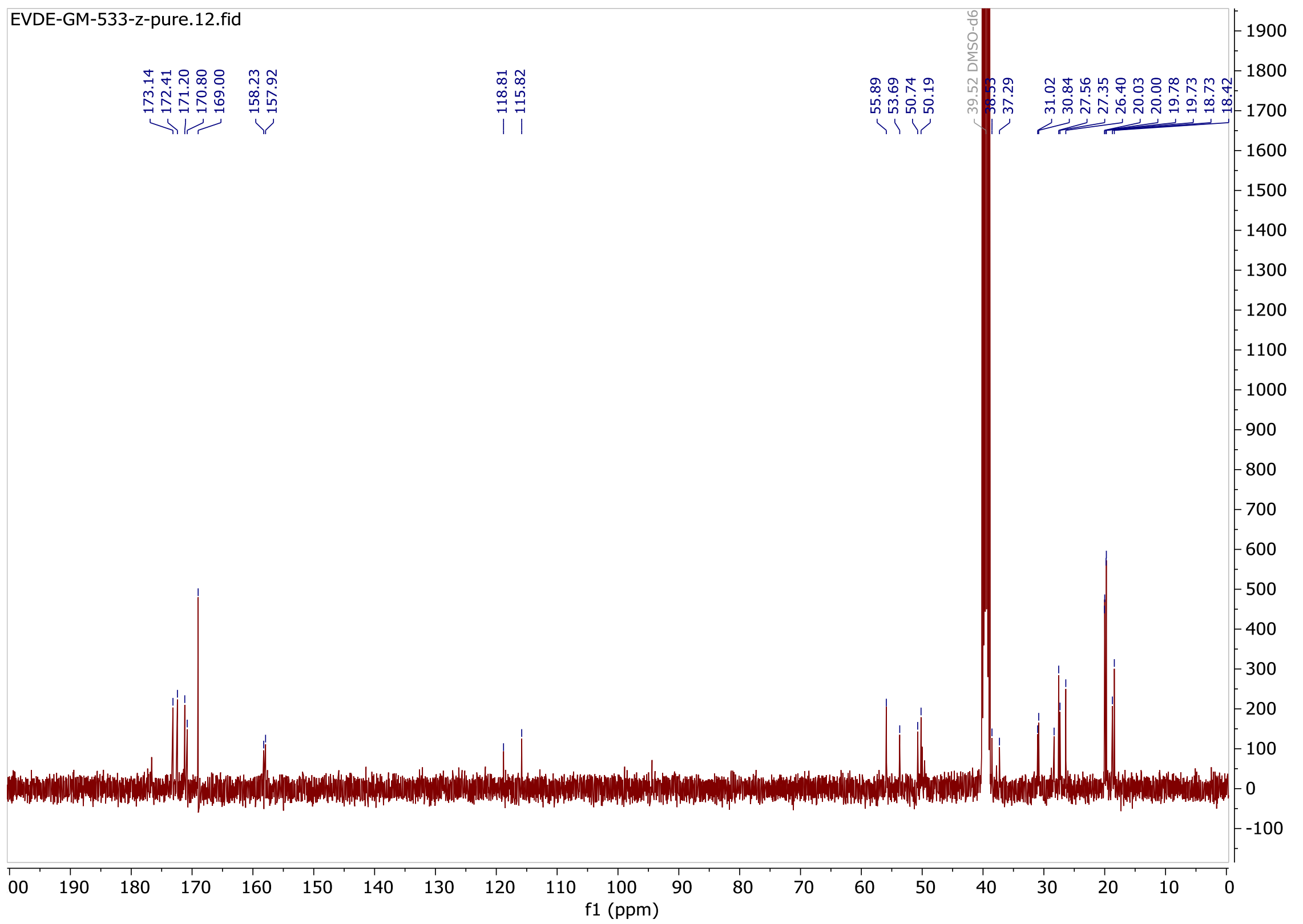

Figure S70. ^{13}C-NMR spectrum of compound **33.**

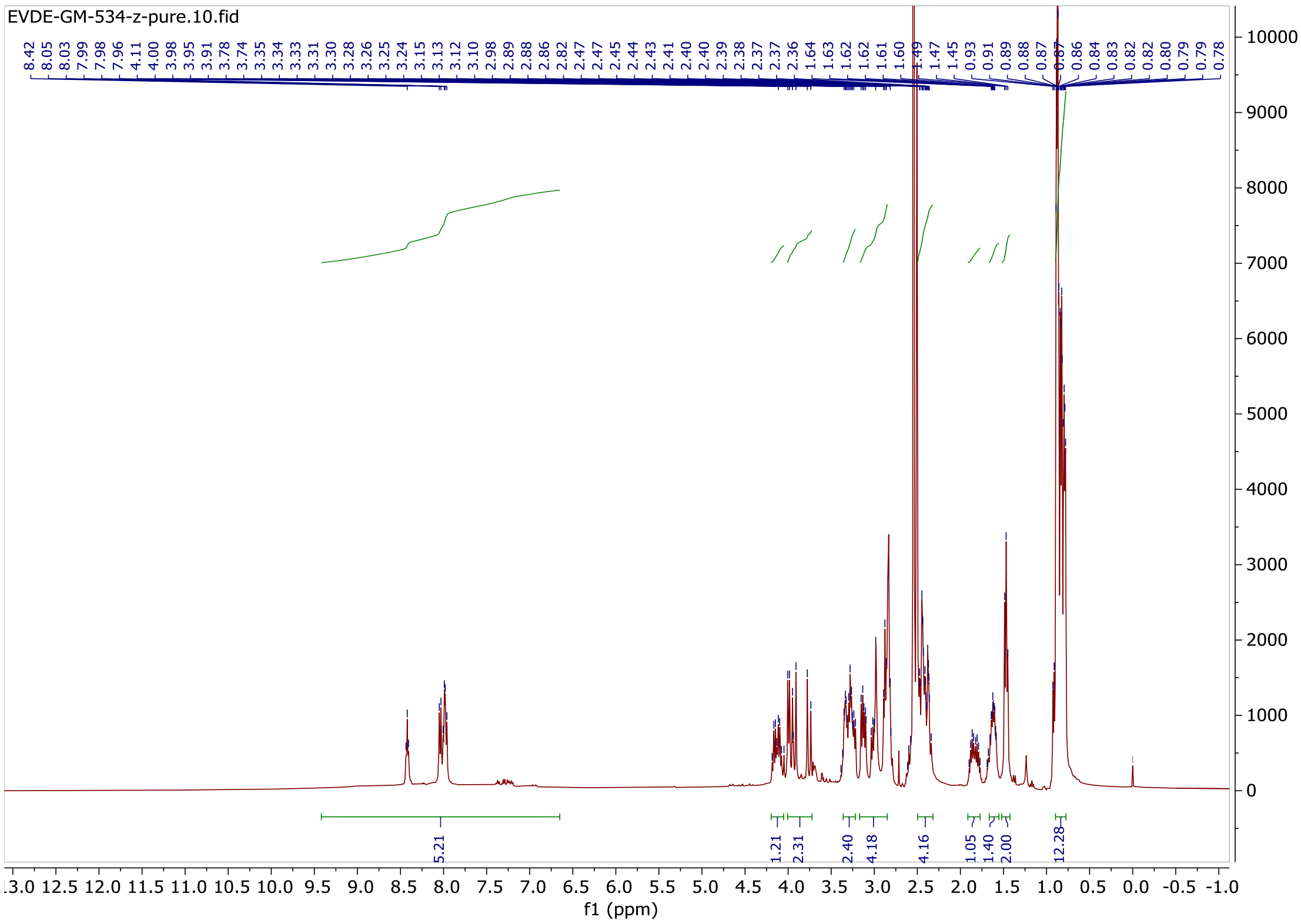

Figure S71. ^{1}H-NMR spectrum of compound **34.**

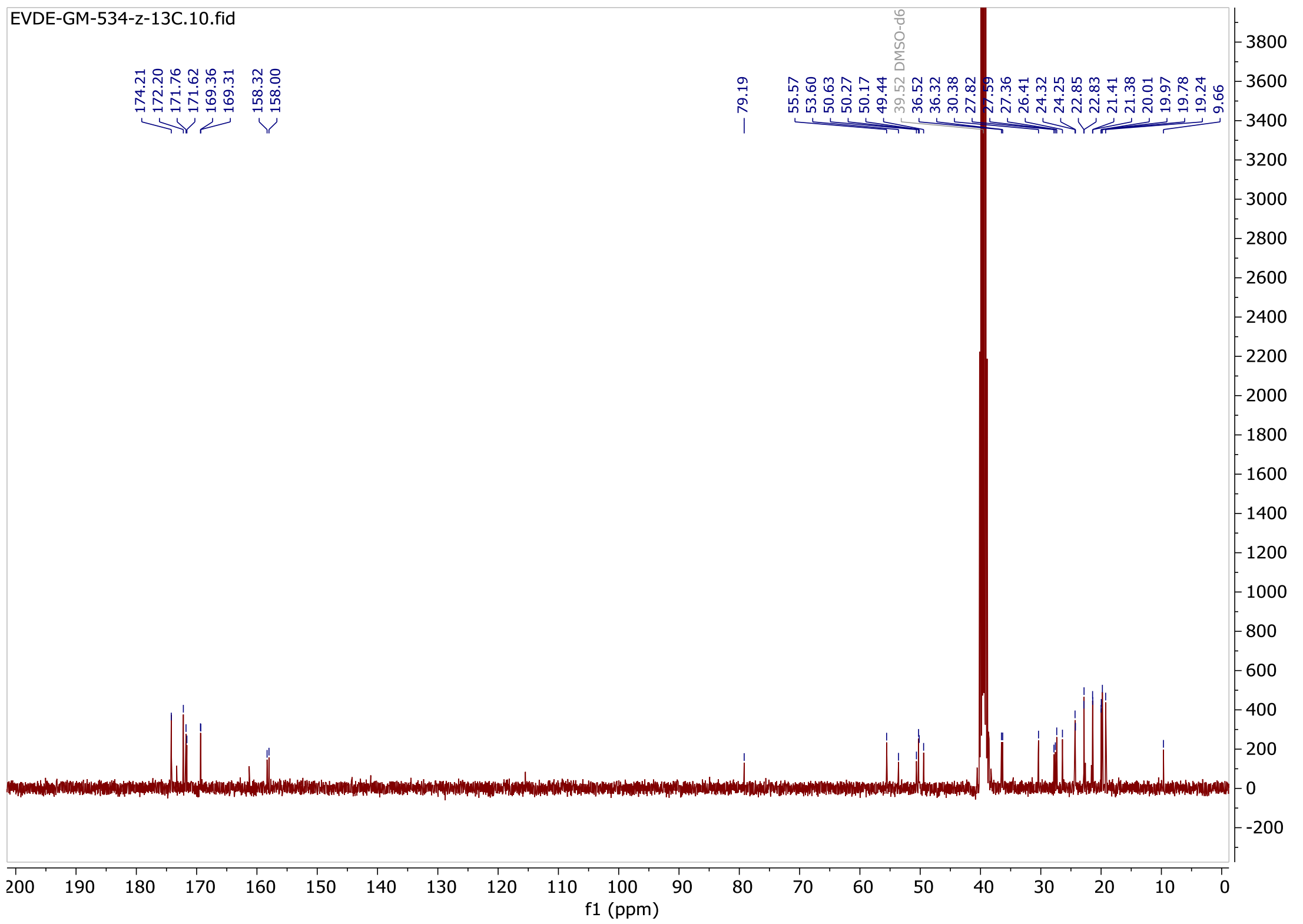

Figure S72. ^{13}C-NMR spectrum of compound **34.**

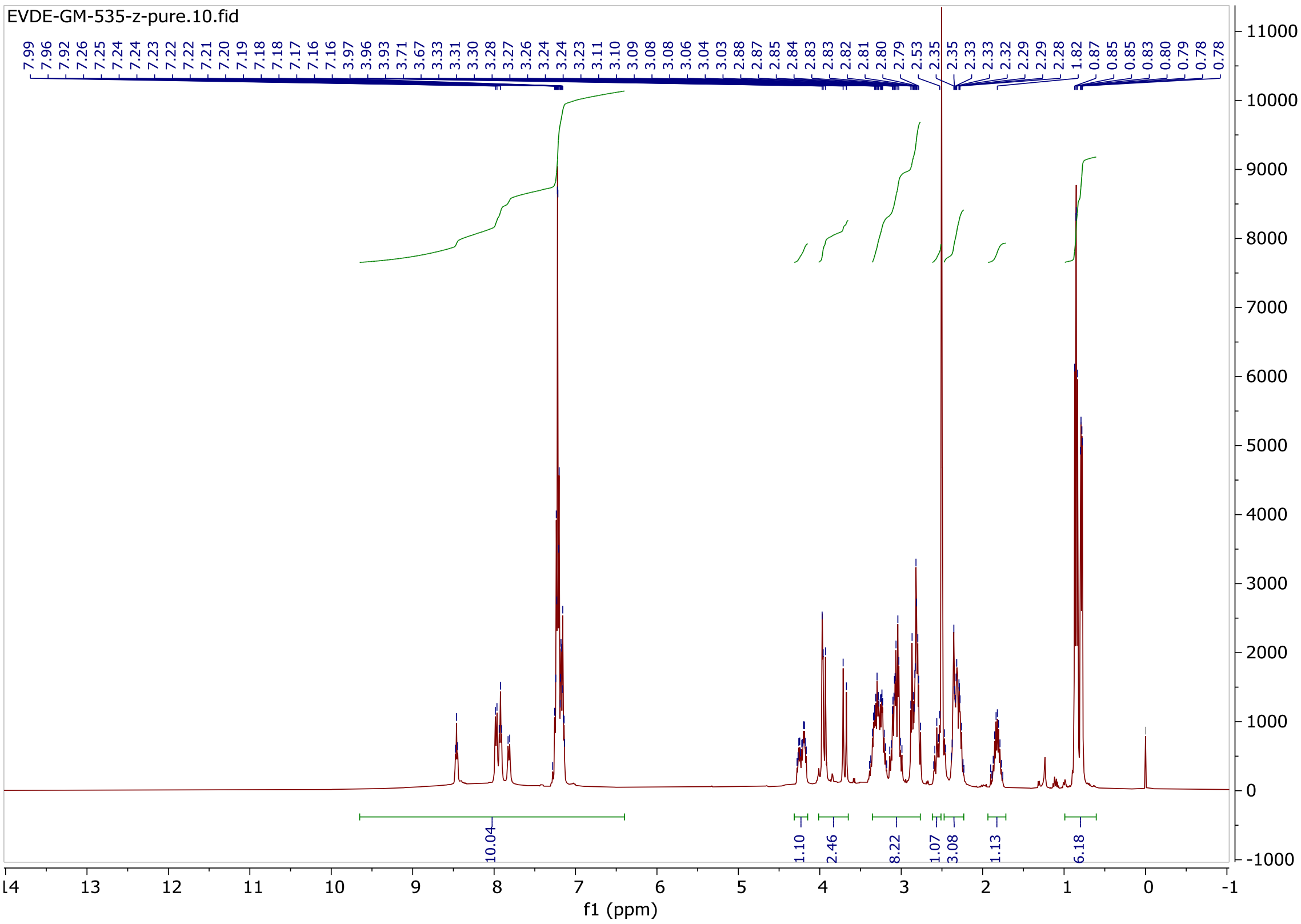

Figure S73. ^{1}H-NMR spectrum of compound **35.**

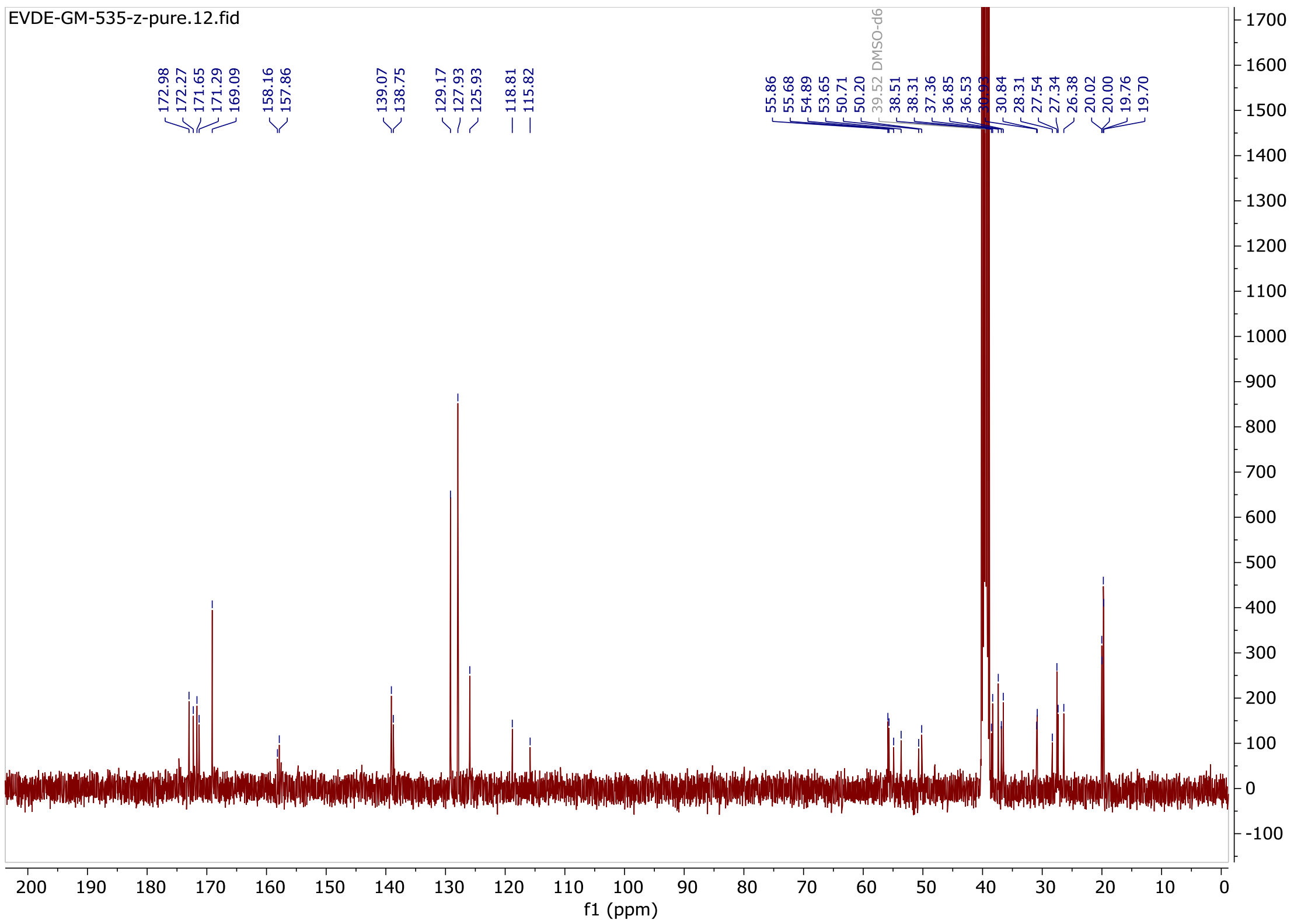

Figure S74. ^{13}C-NMR spectrum of compound **35.**

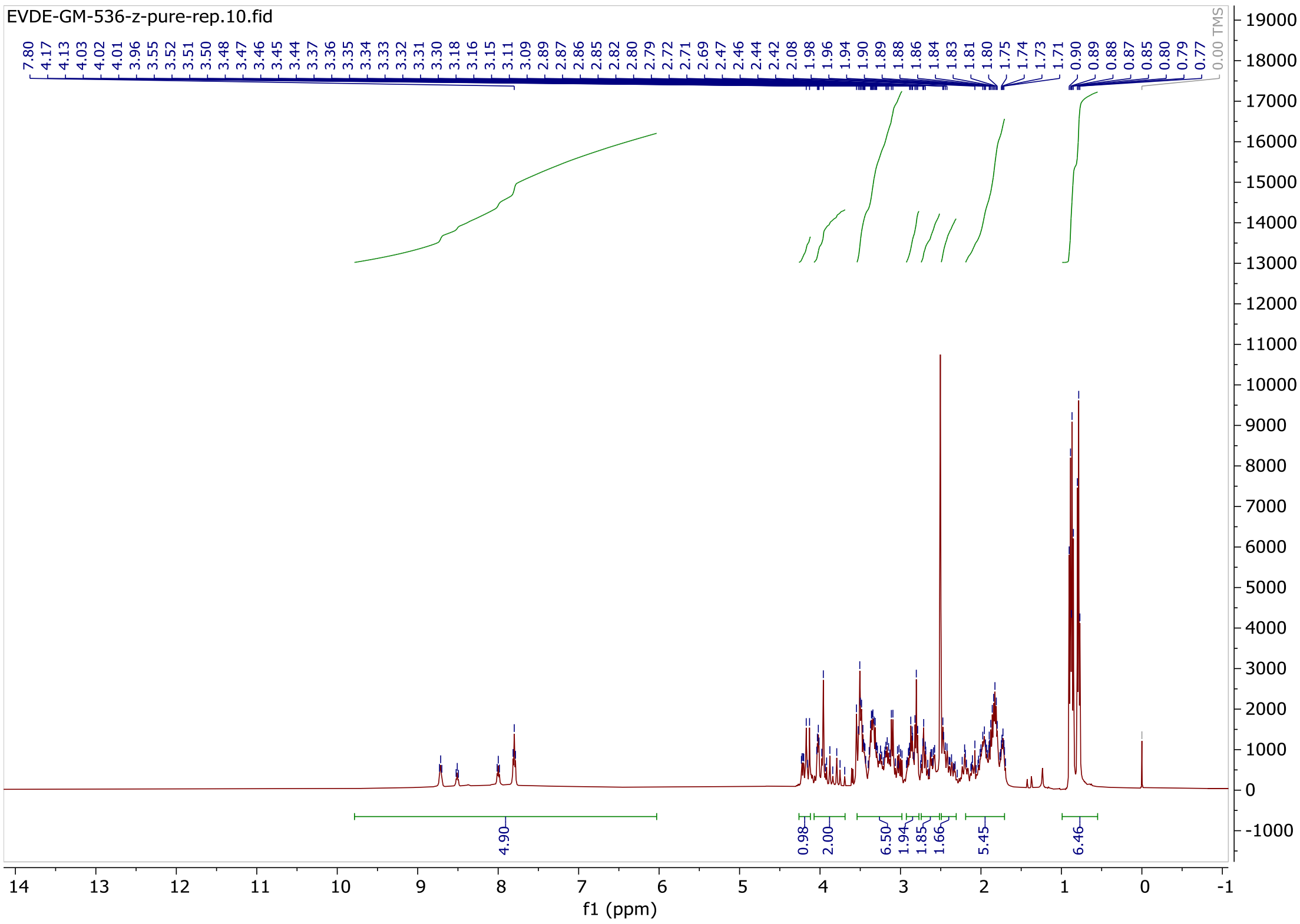

Figure S75. ^{1}H-NMR spectrum of compound **36.**

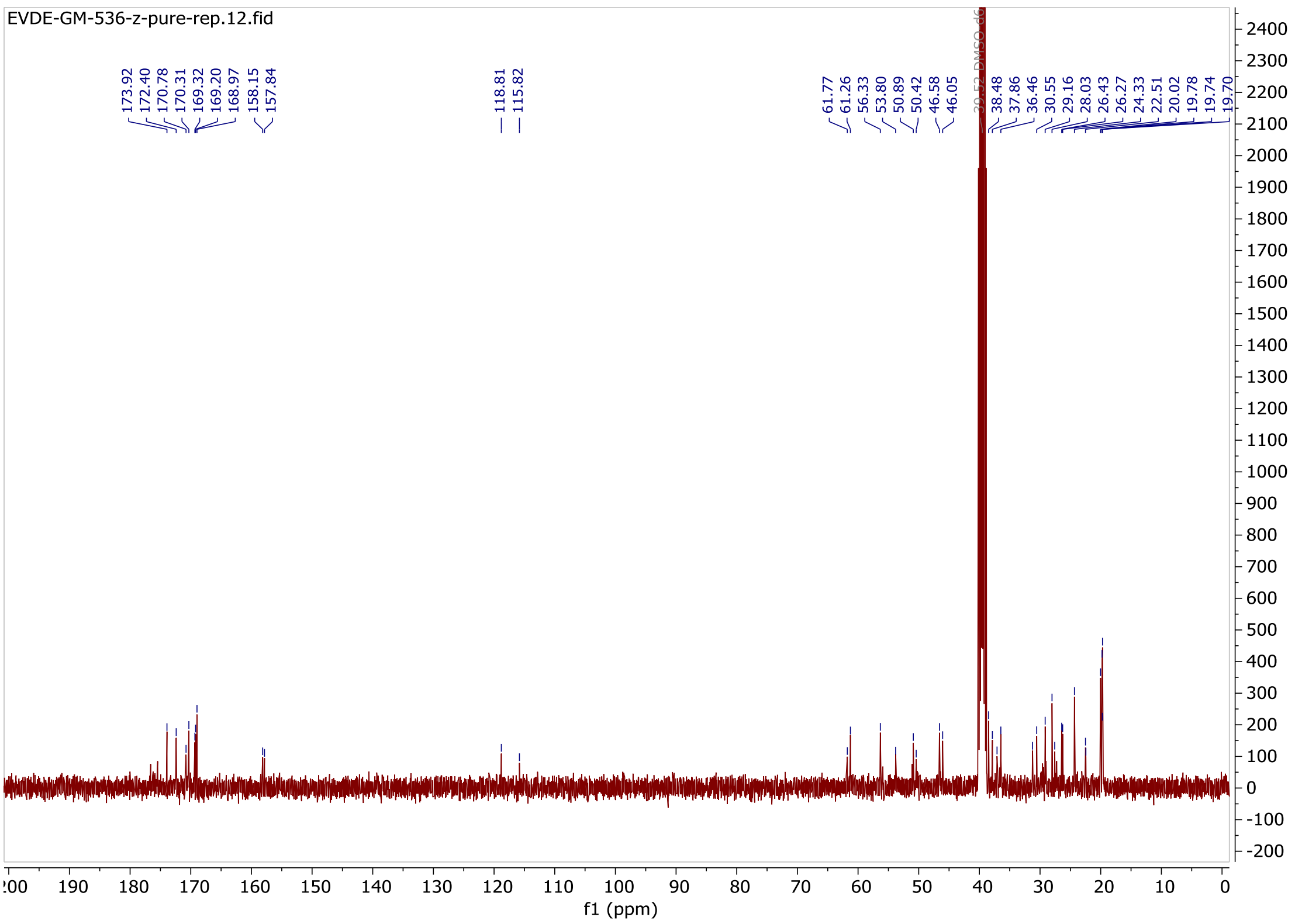

Figure S76. ^{13}C-NMR spectrum of compound **36.**

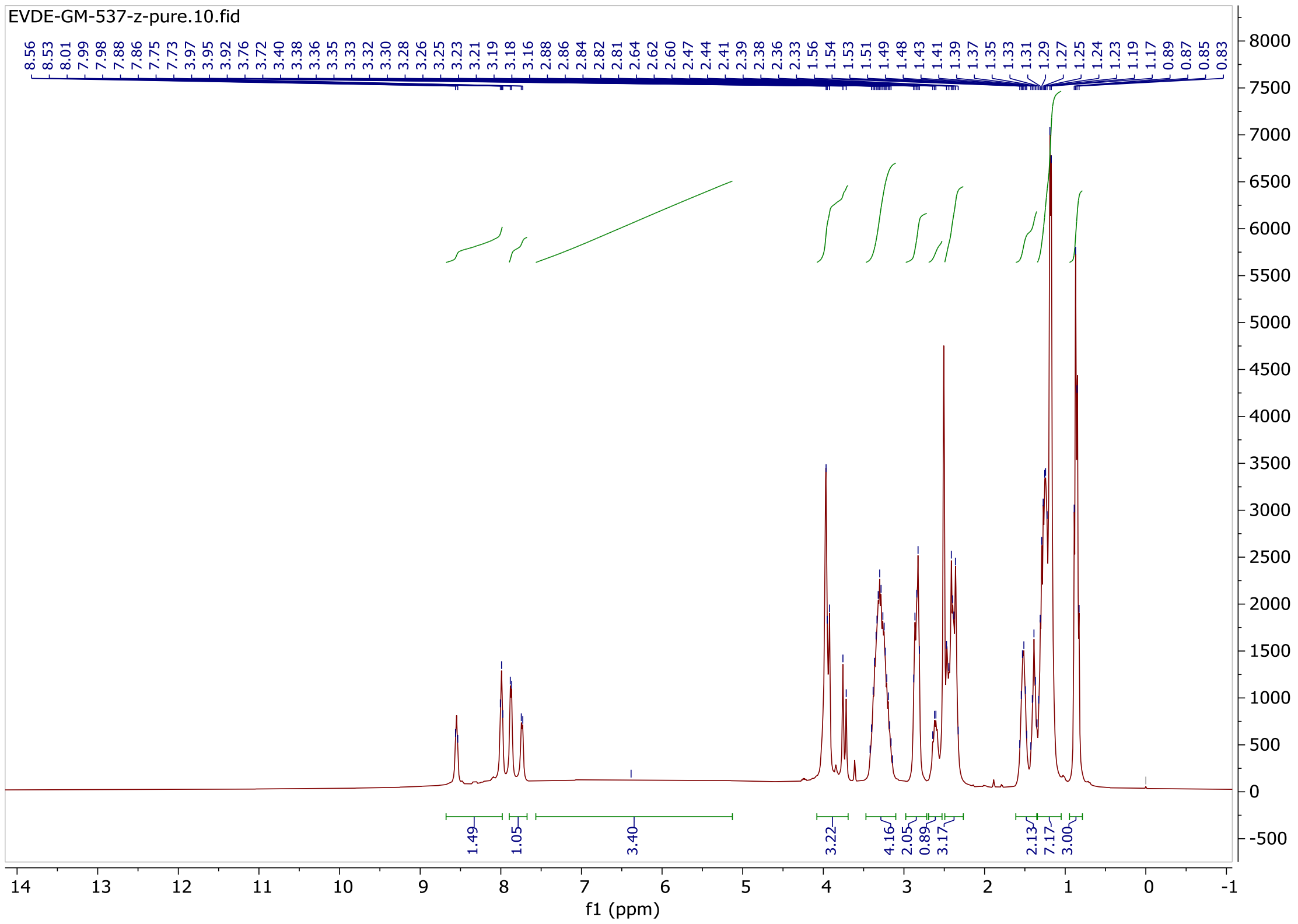

Figure S77. ^{1}H-NMR spectrum of compound **37.**

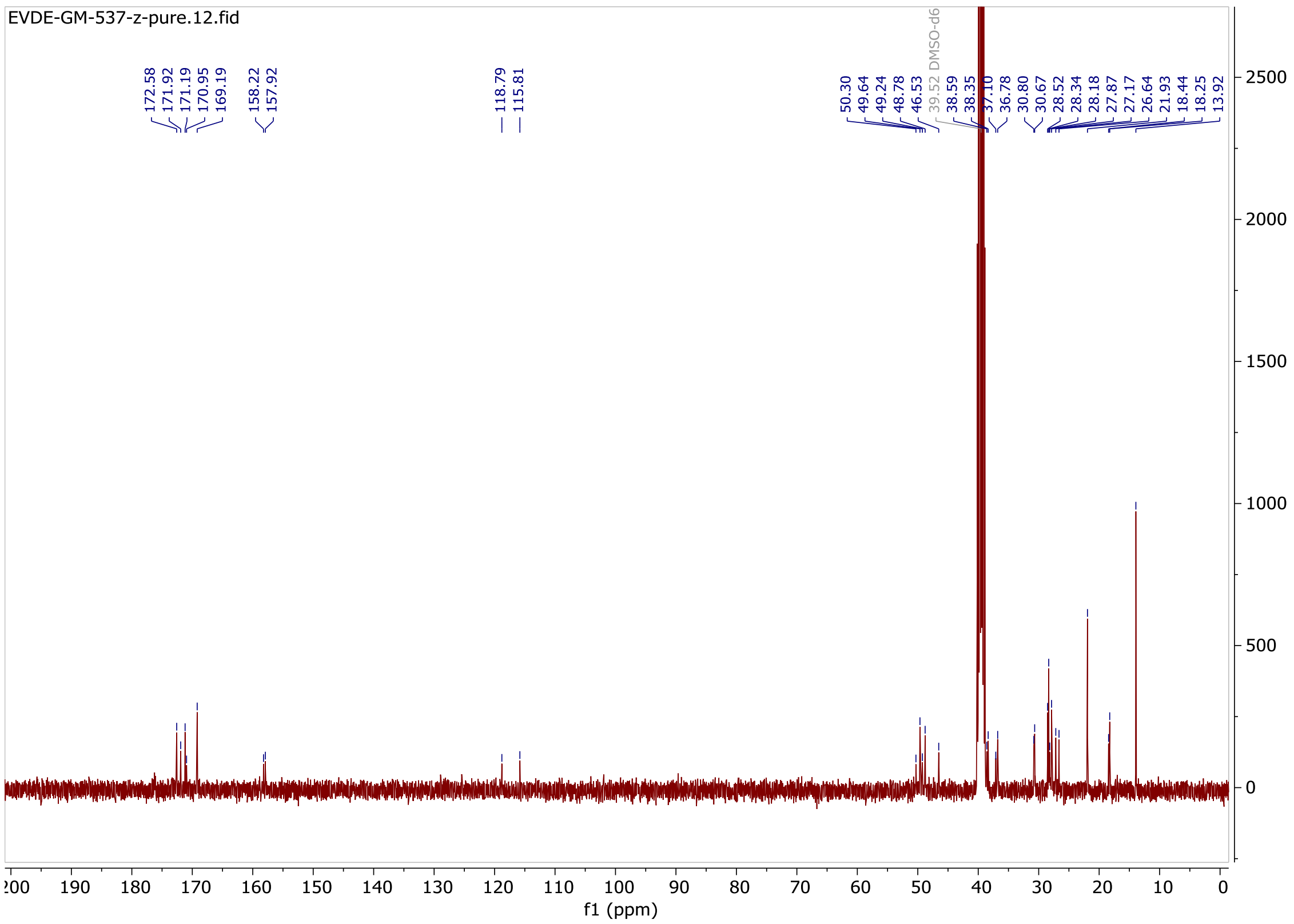

Figure S78. ^{13}C-NMR spectrum of compound **37.**

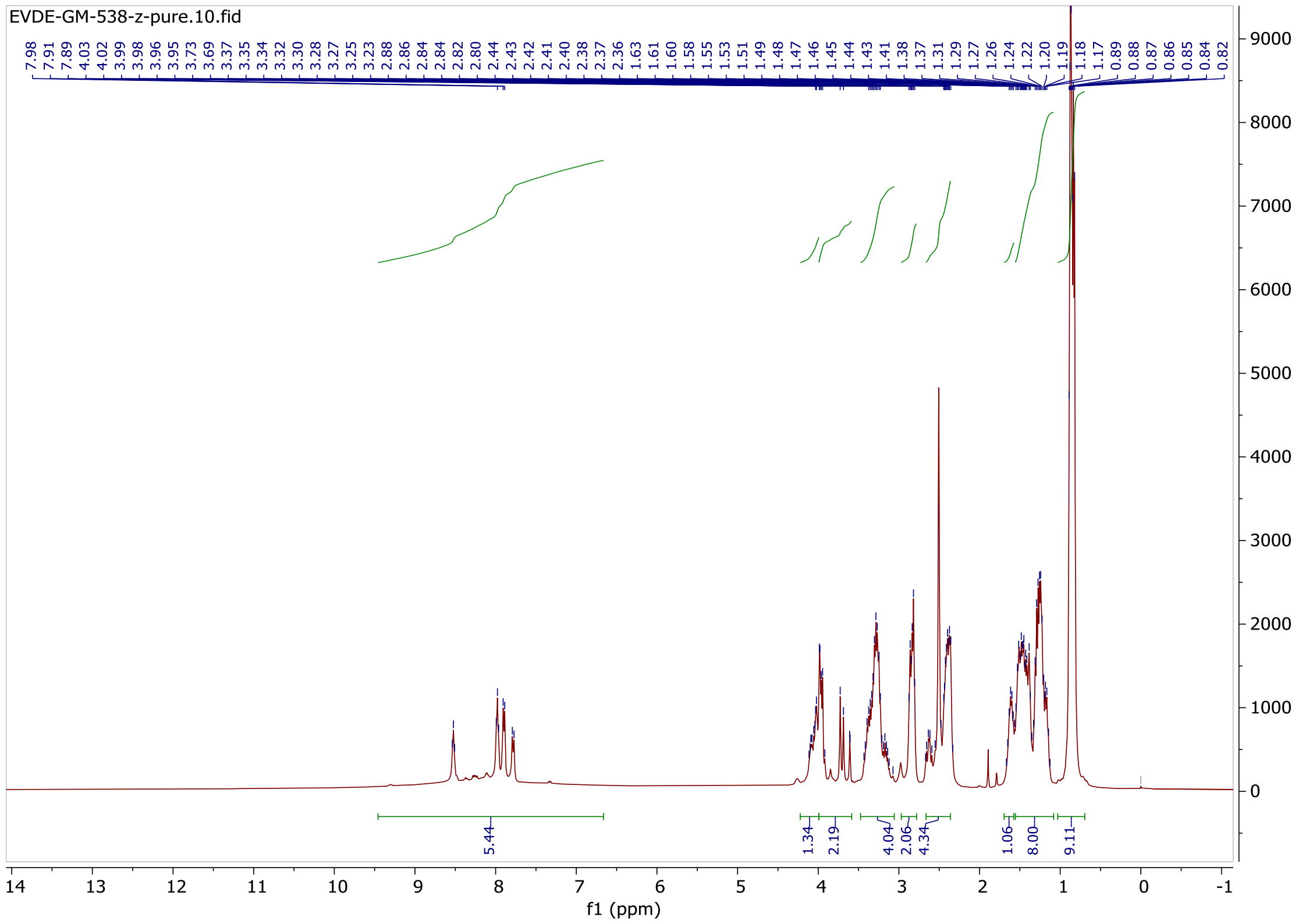

Figure S79. ^{1}H-NMR spectrum of compound **38.**

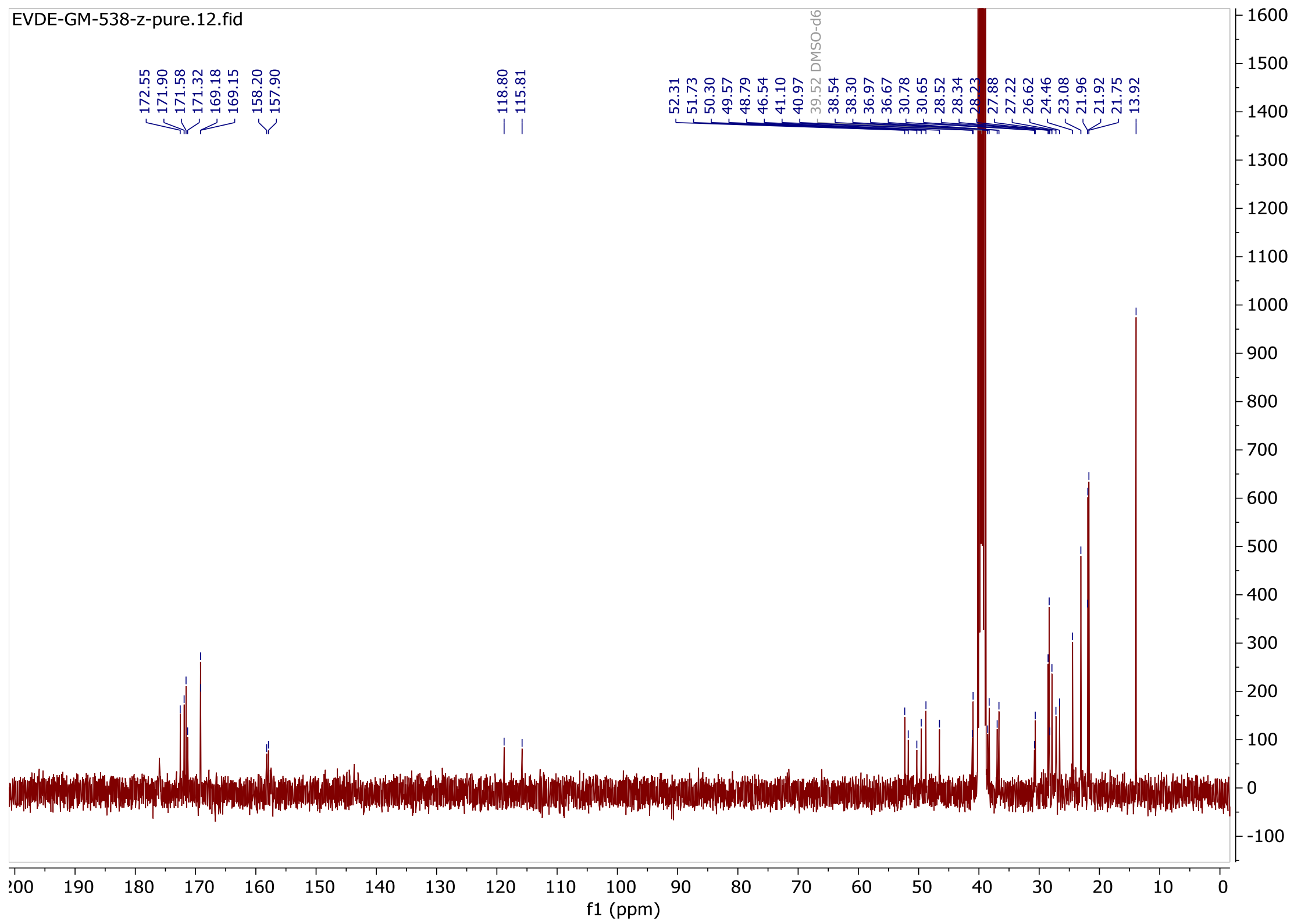

Figure S80. ^{13}C-NMR spectrum of compound **38.**

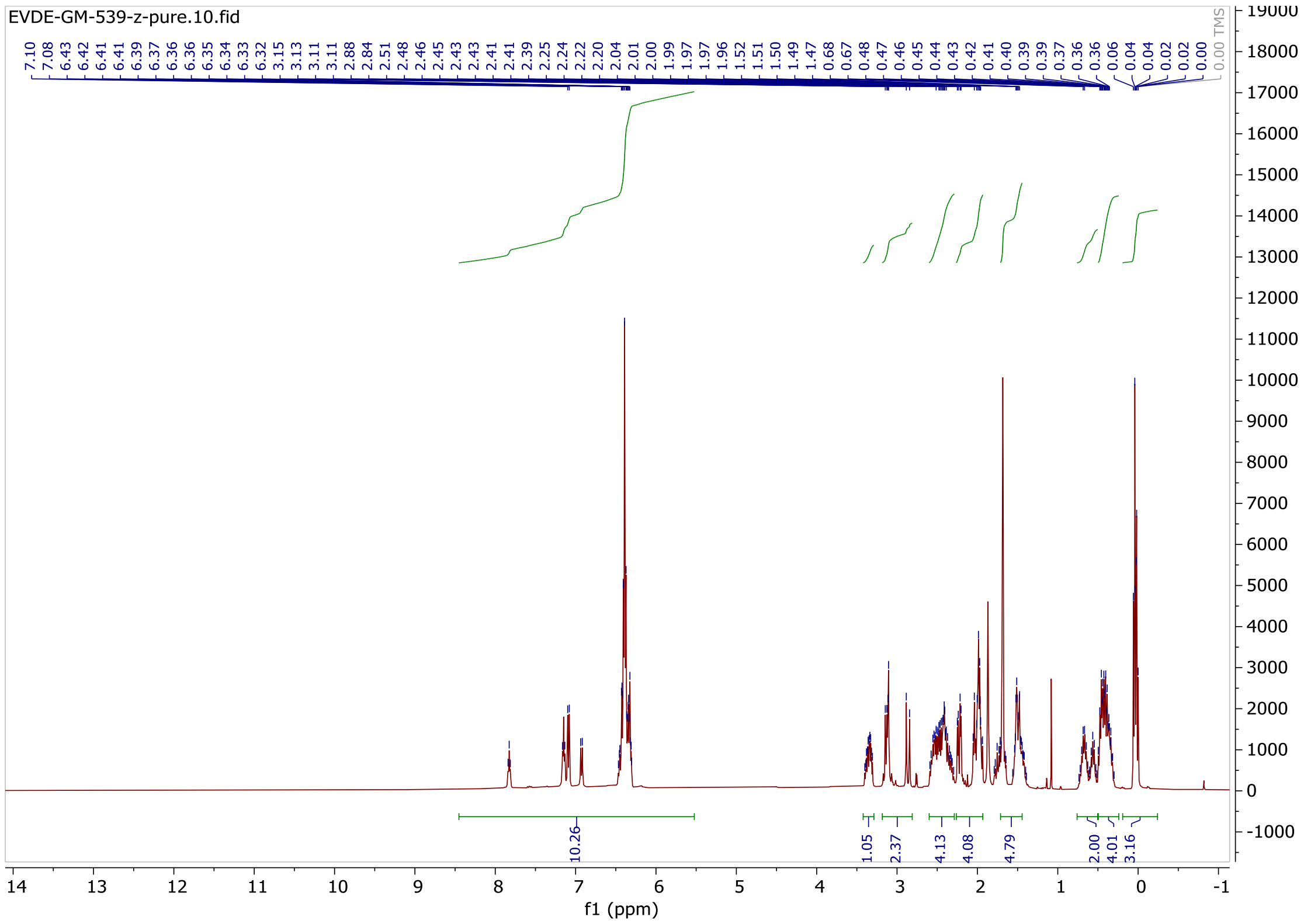

Figure S81. ^{1}H-NMR spectrum of compound **39.**

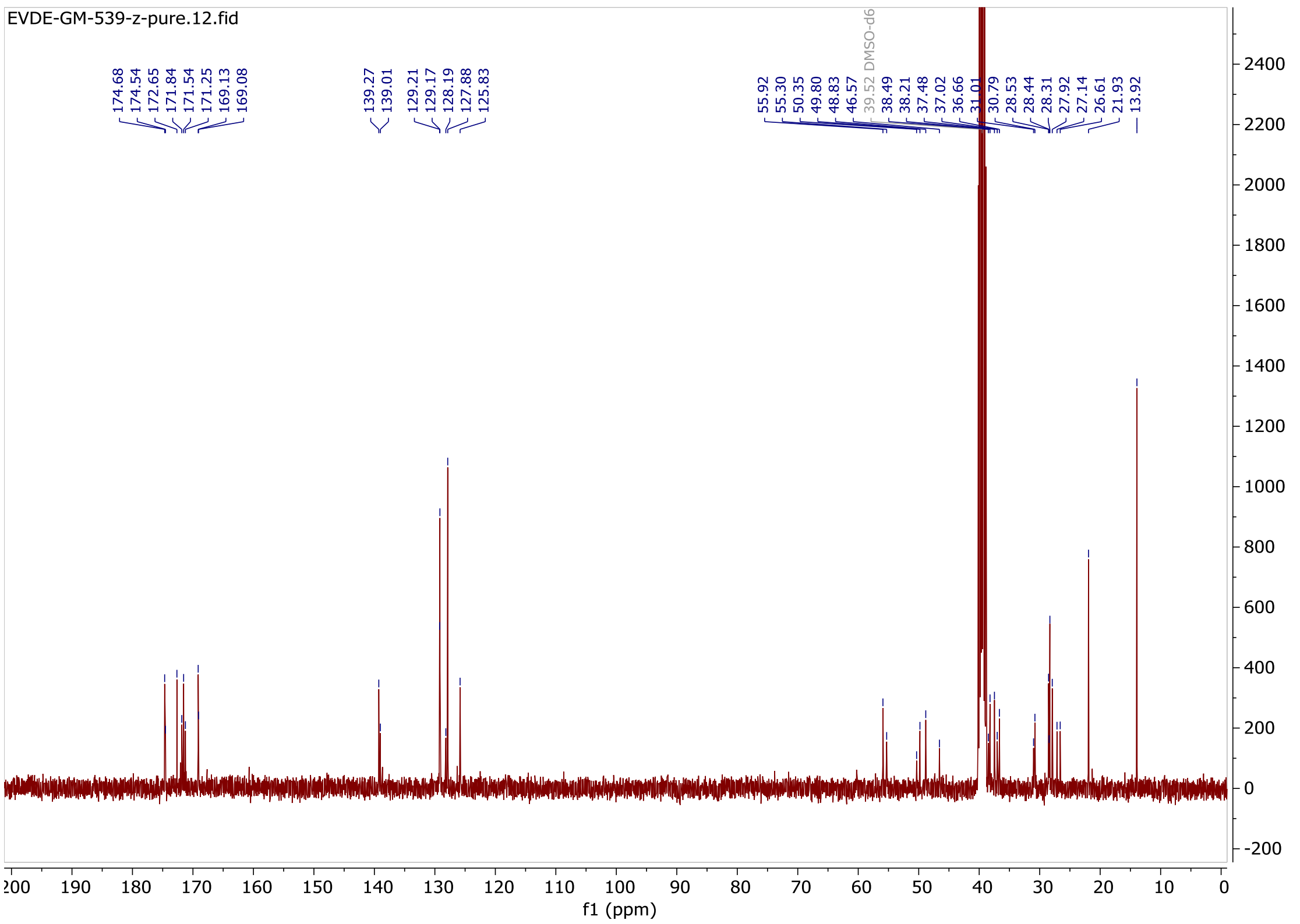

Figure S82. ^{13}C-NMR spectrum of compound **39.**

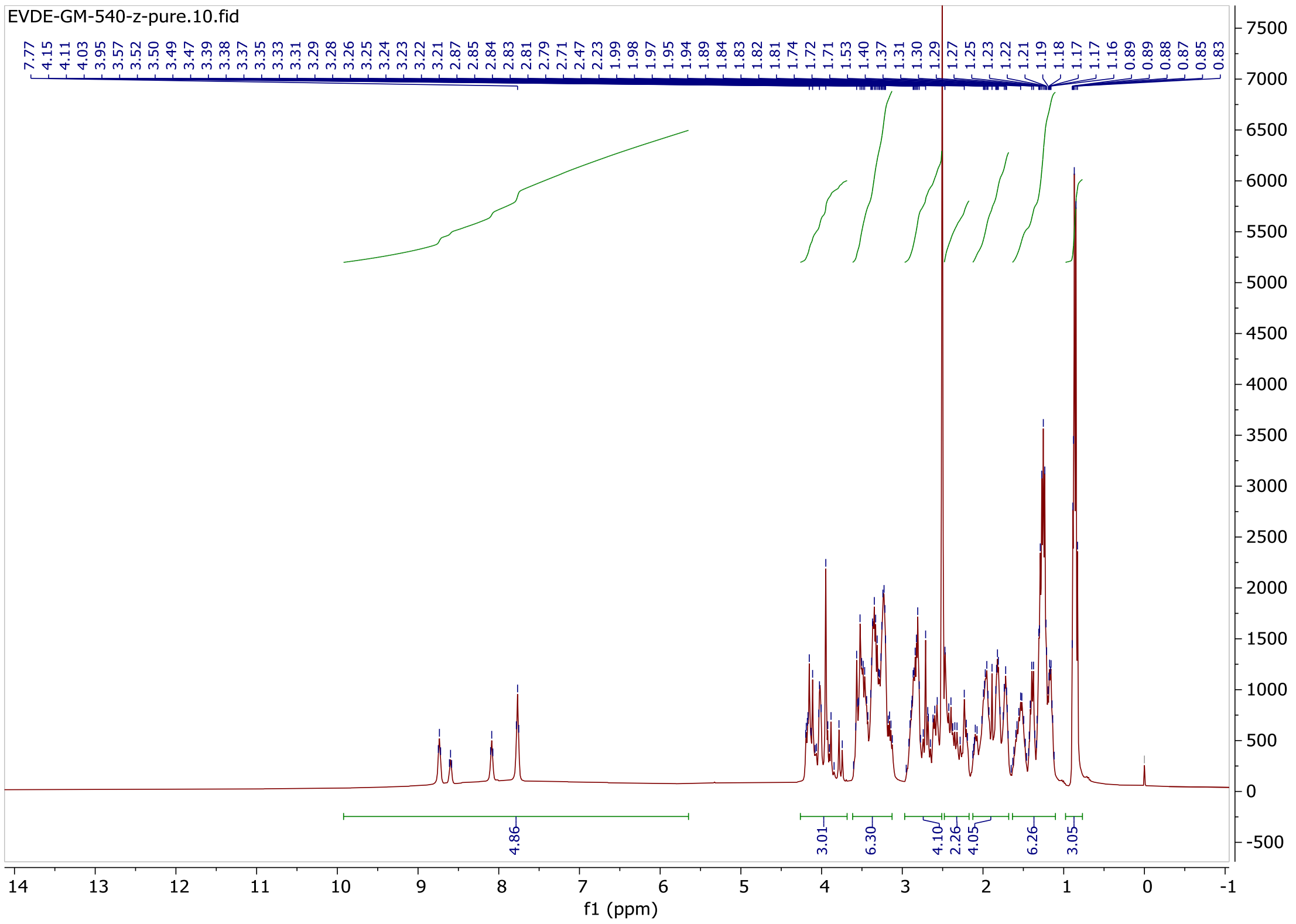

Figure S83. ^{1}H-NMR spectrum of compound **40.**

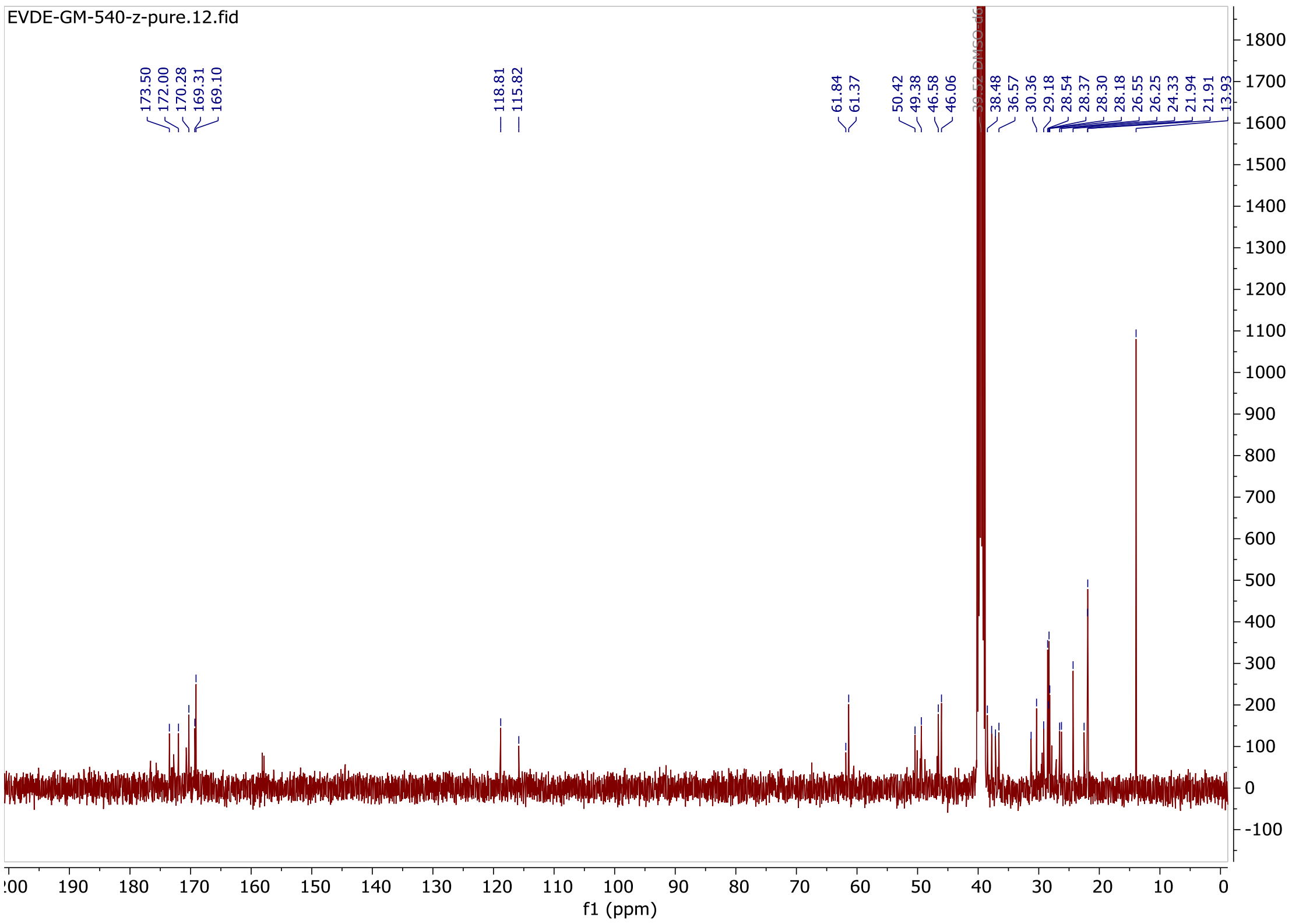

Figure S84. ^{13}C-NMR spectrum of compound **40.**

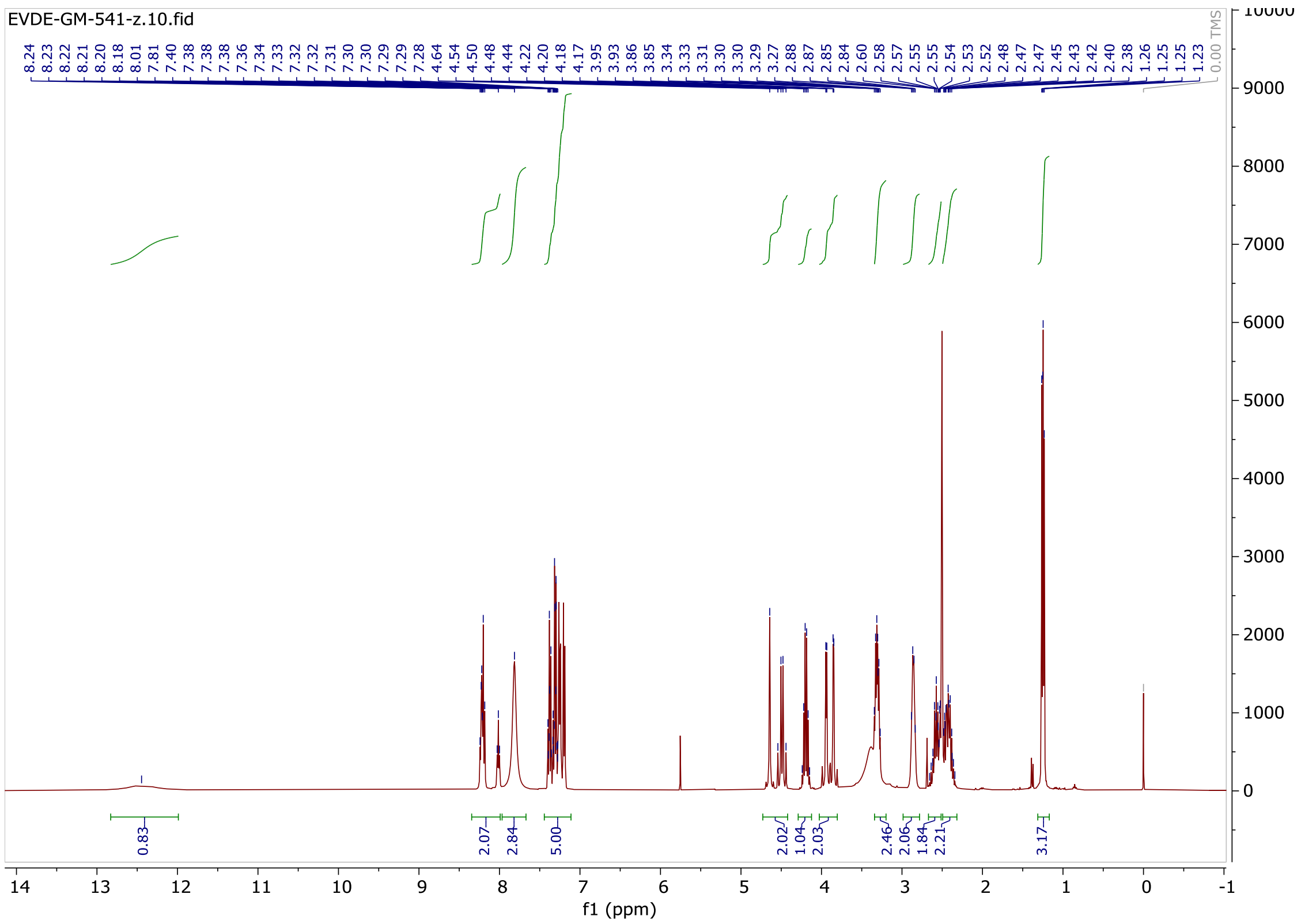

Figure S85. ^{1}H-NMR spectrum of compound **41.**

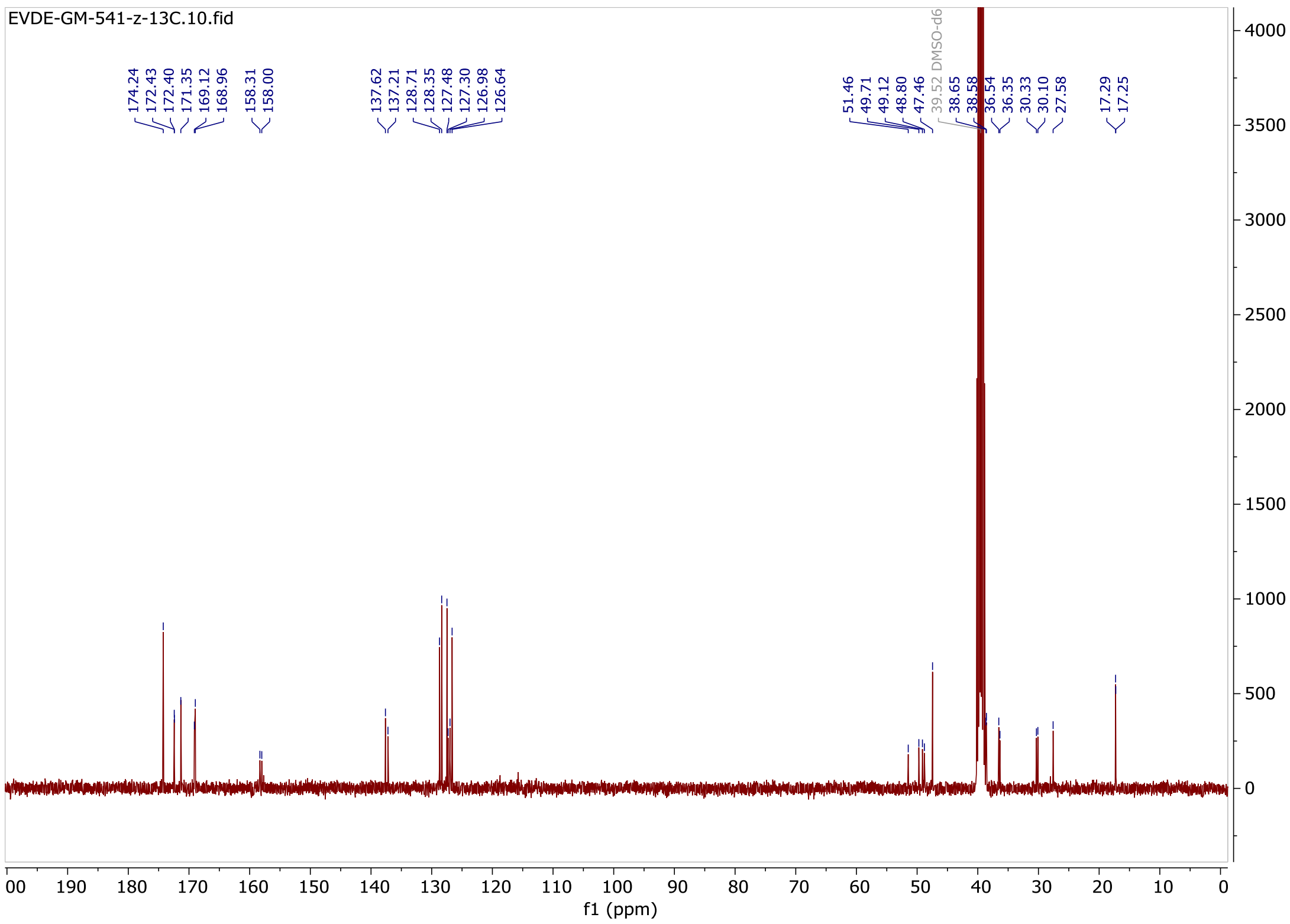

Figure S86. ^{13}C-NMR spectrum of compound **41.**

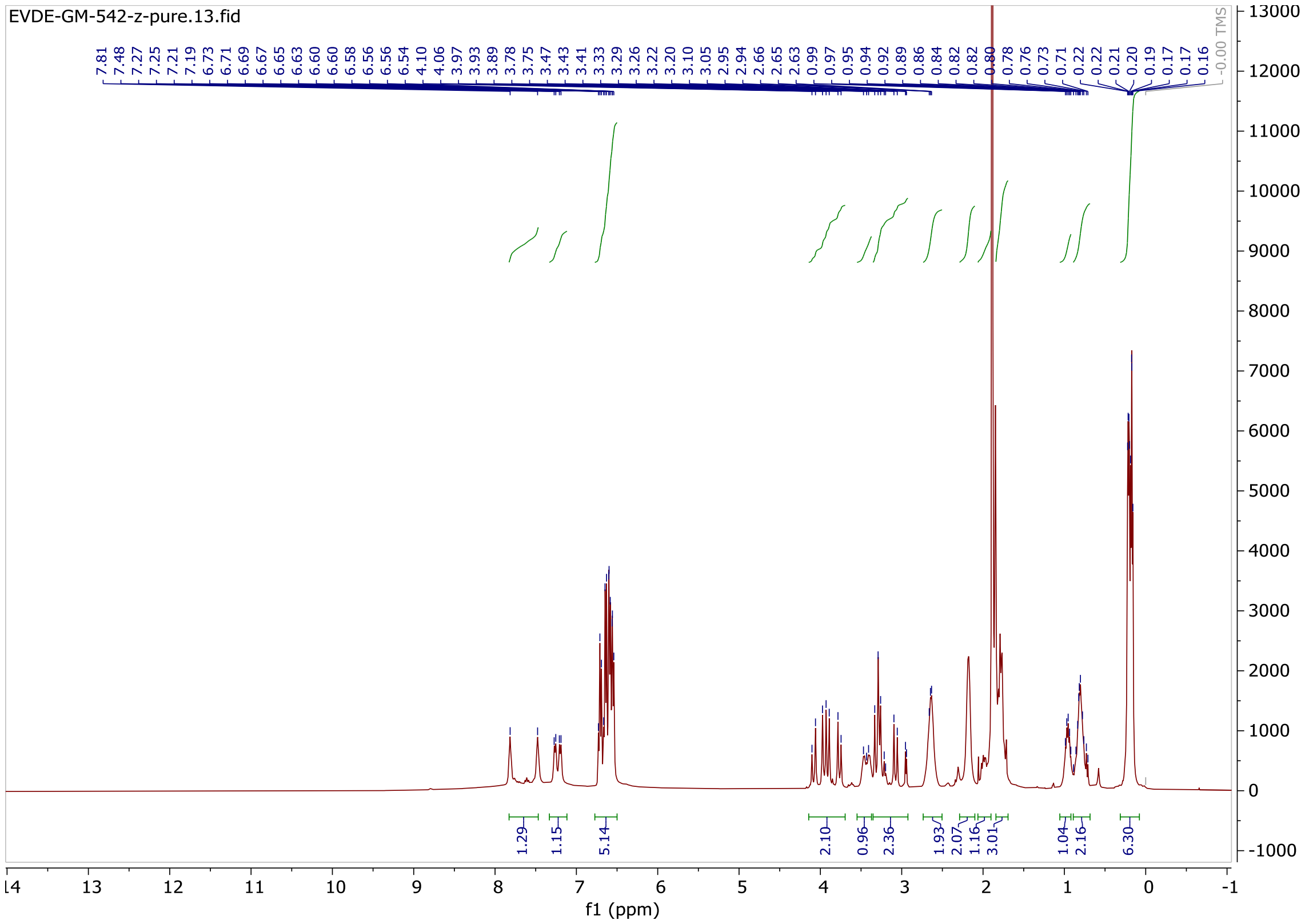

Figure S87. ^{1}H-NMR spectrum of compound **42.**

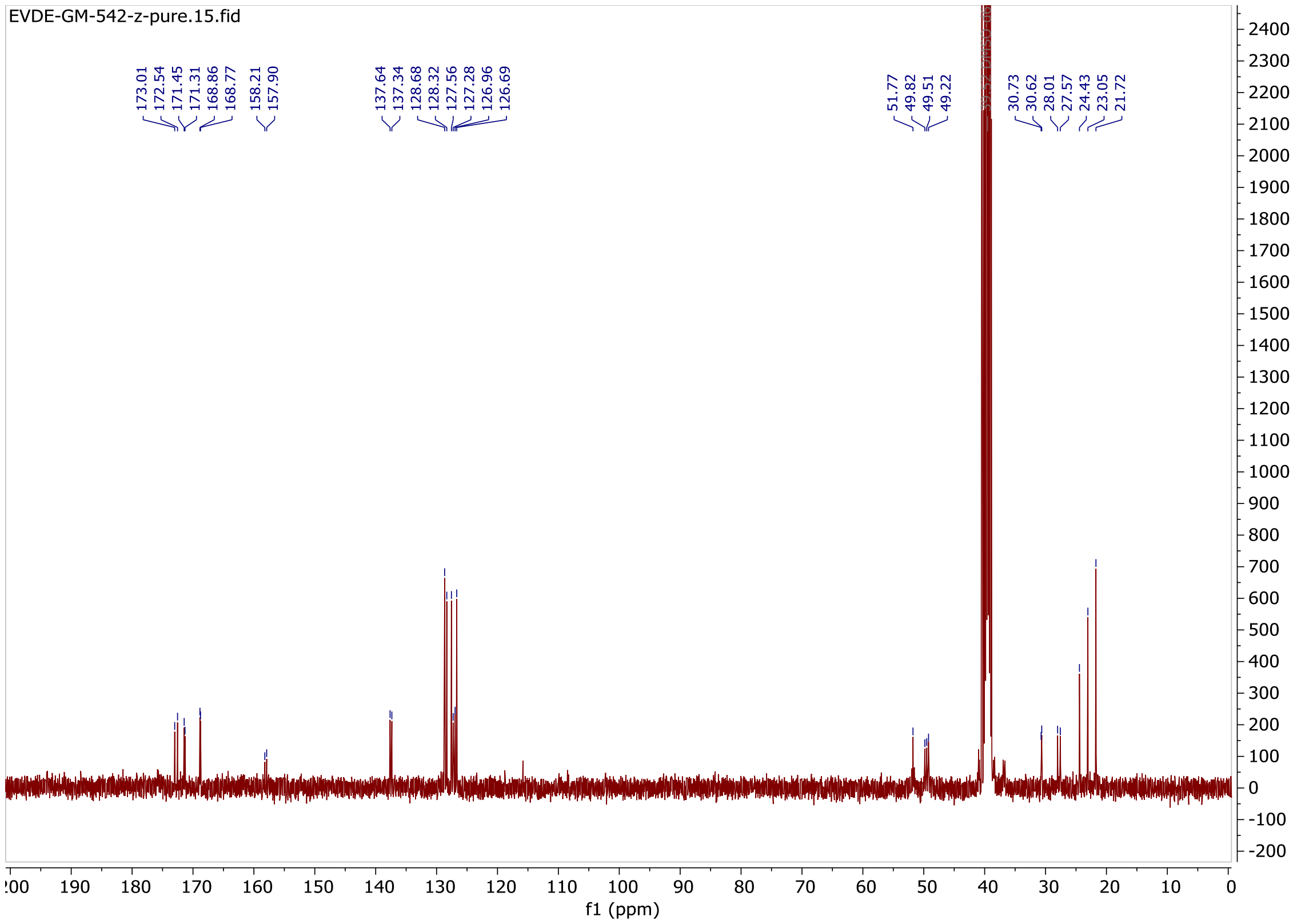

Figure S88. ^{13}C-NMR spectrum of compound **42.**

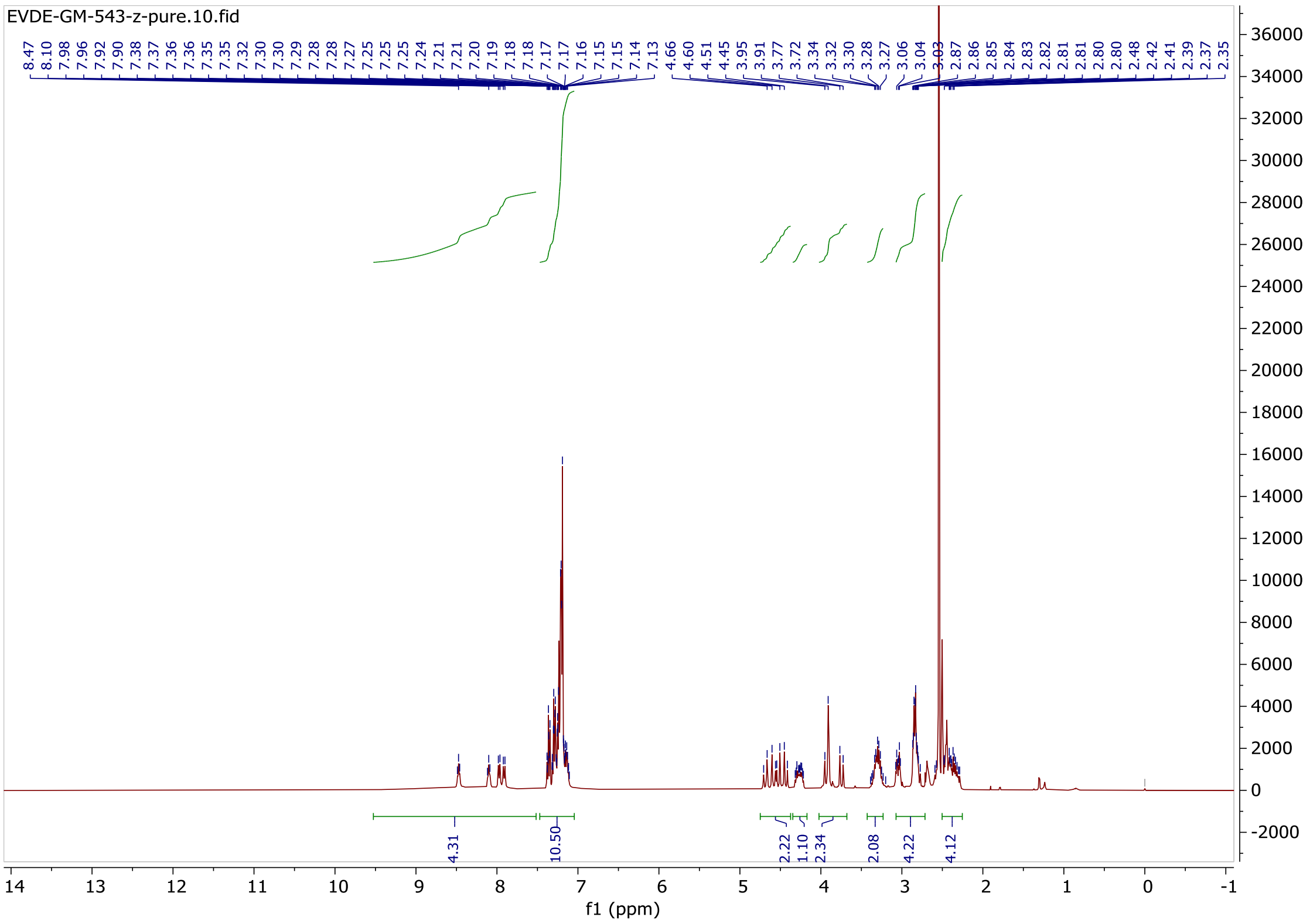

Figure S89. ^{1}H-NMR spectrum of compound **43.**

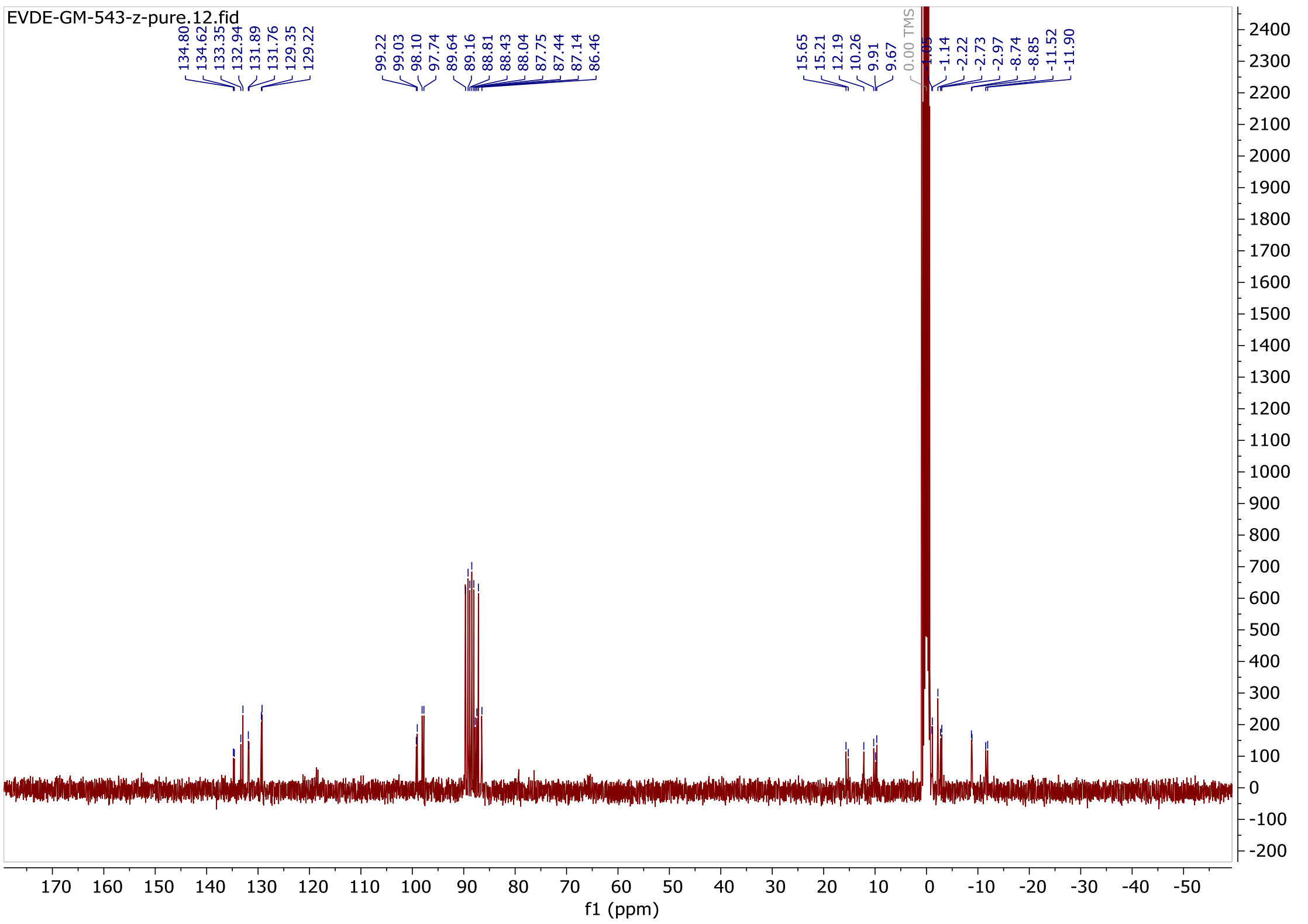

Figure S90. ^{13}C-NMR spectrum of compound **43.**

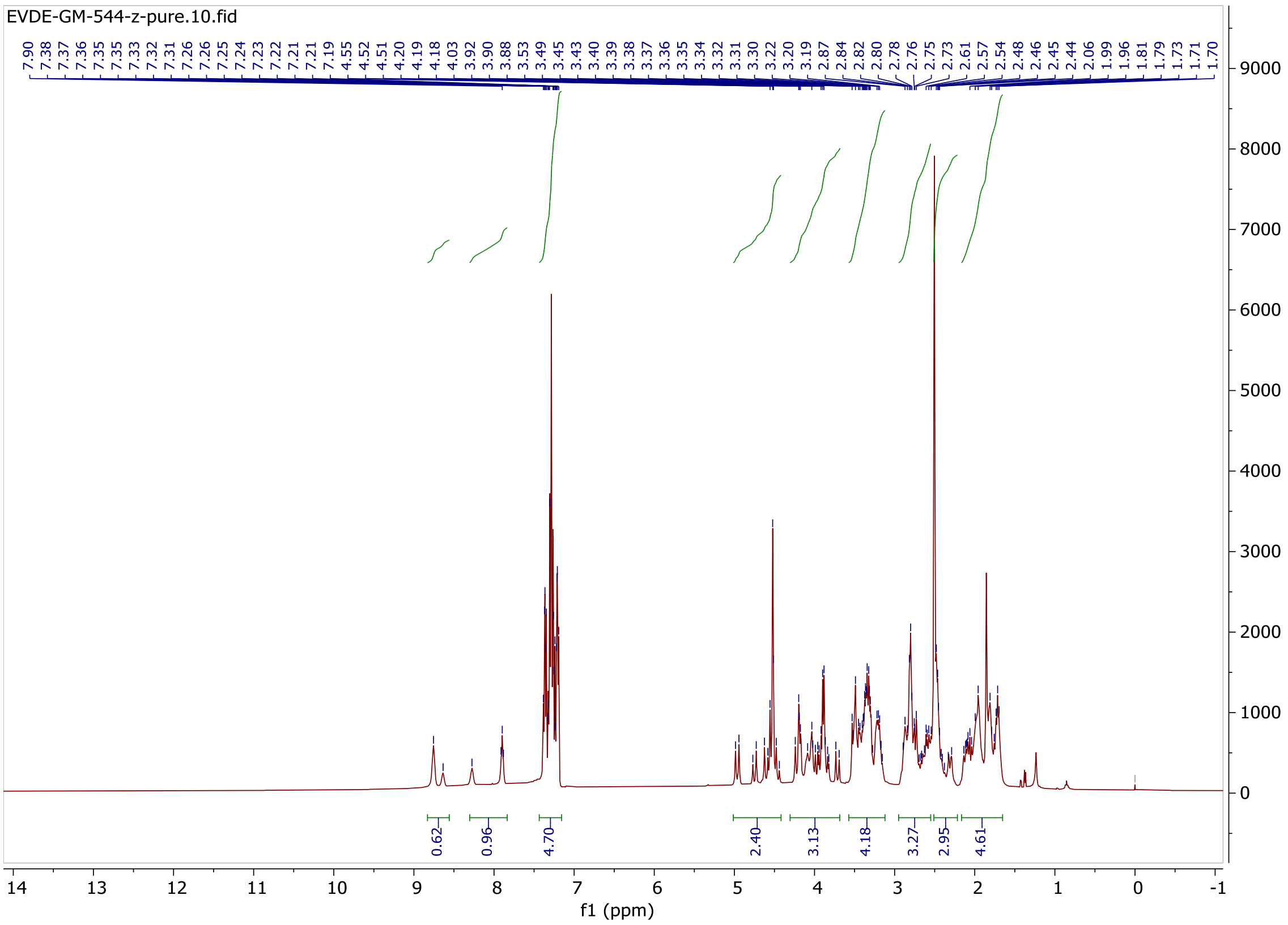

Figure S91. ^{1}H-NMR spectrum of compound **44.**

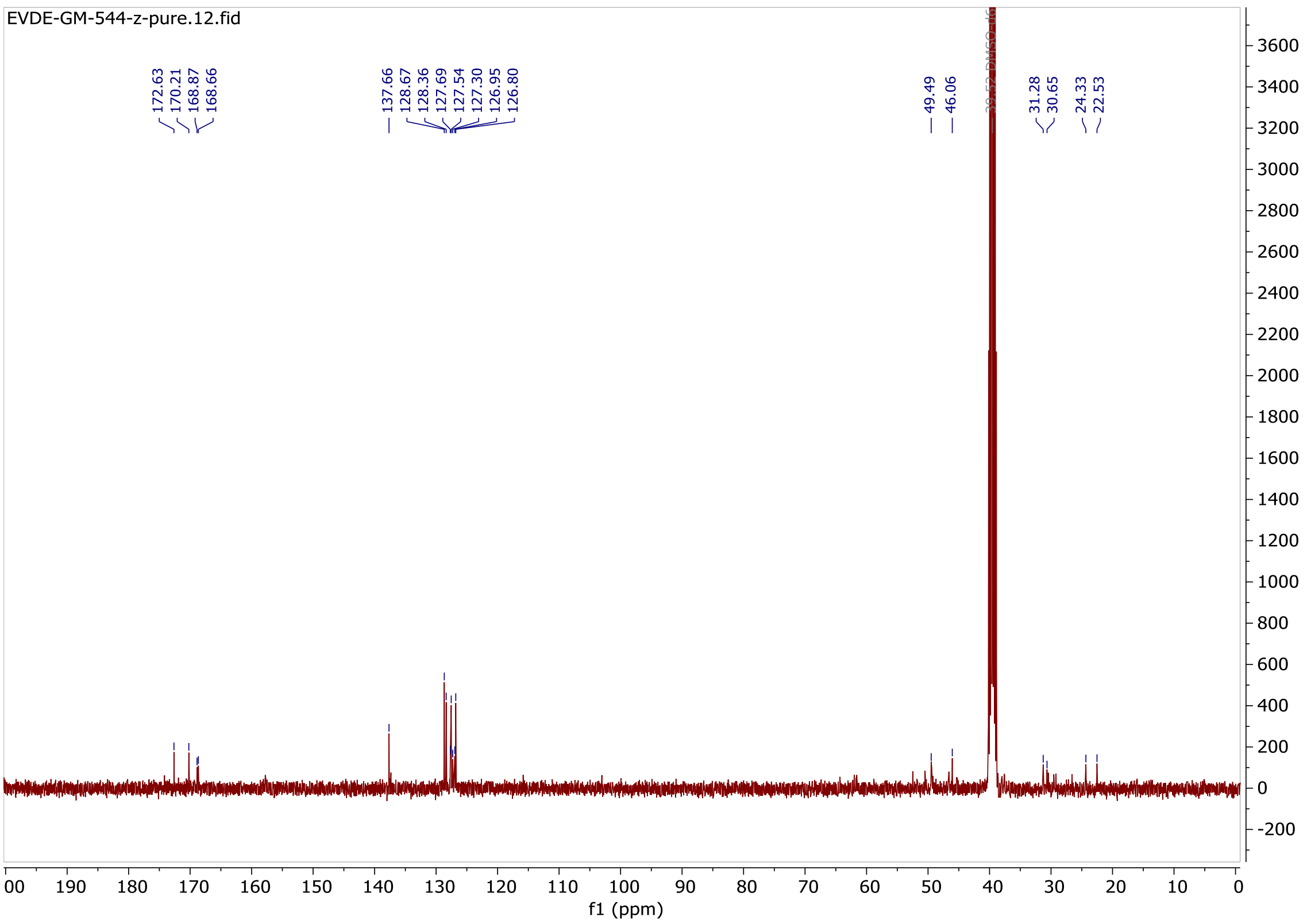

Figure S92. ^{13}C-NMR spectrum of compound **44.**

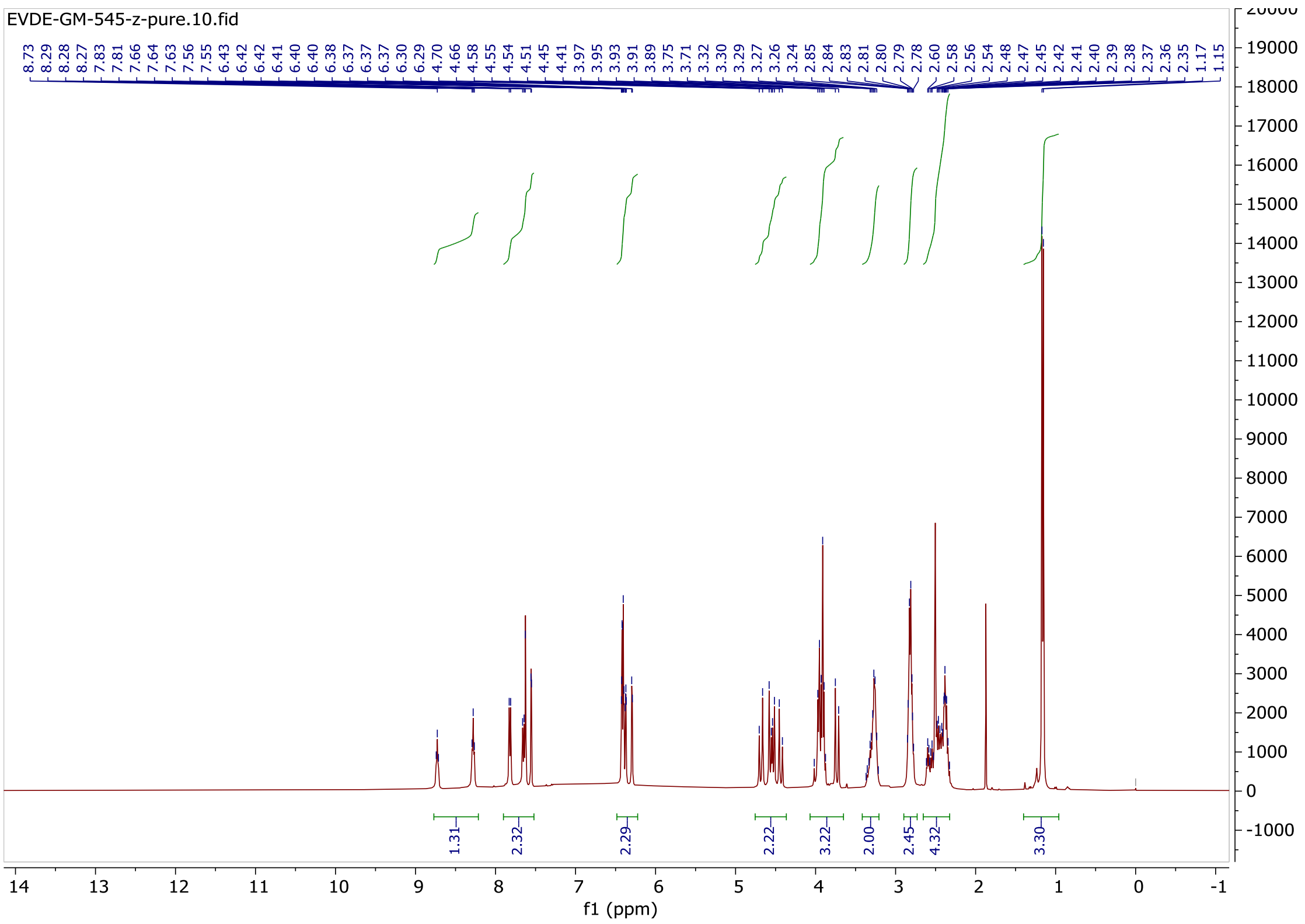

Figure S93. ^{1}H-NMR spectrum of compound **45.**

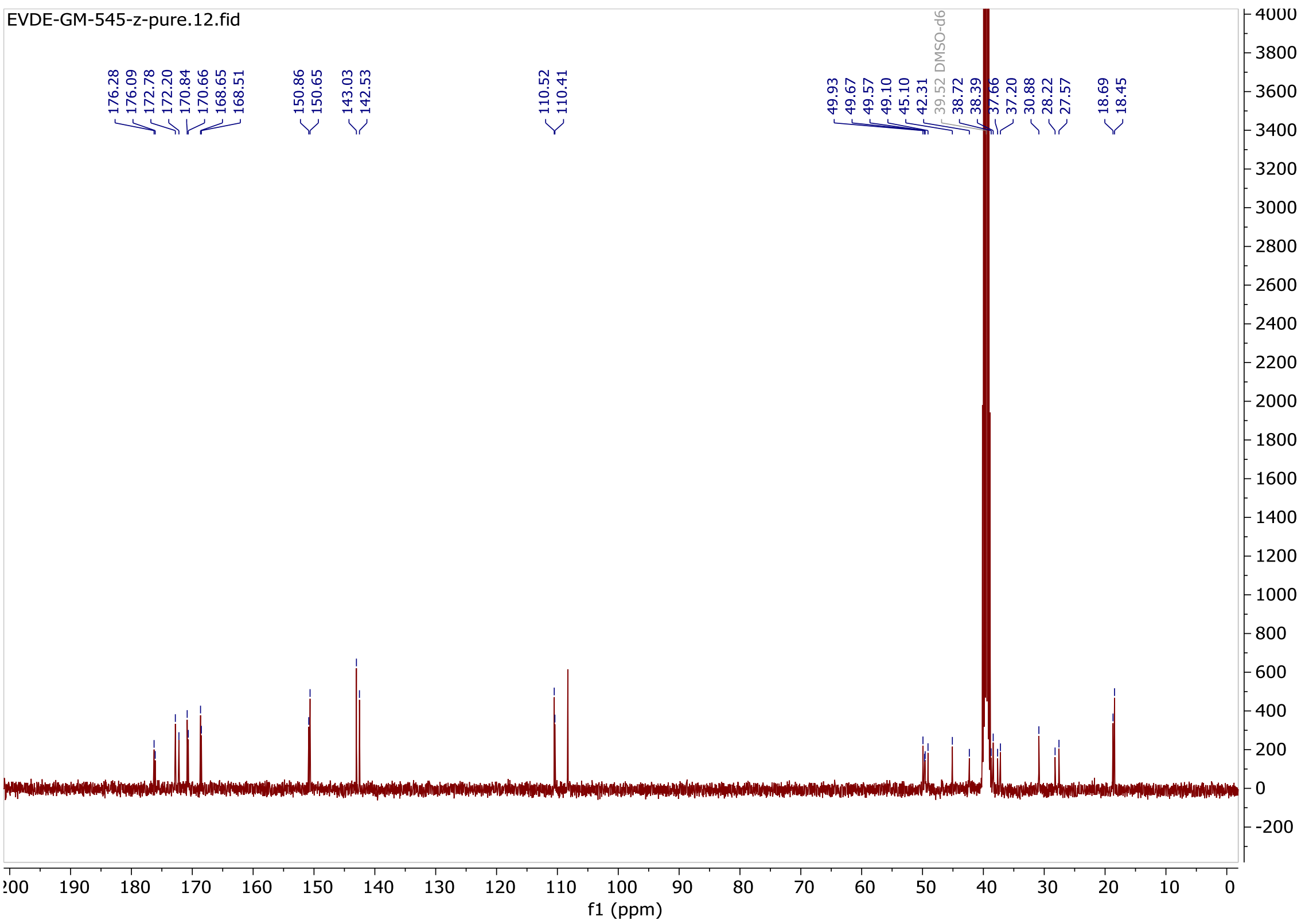

Figure S94. ^{13}C-NMR spectrum of compound **45.**

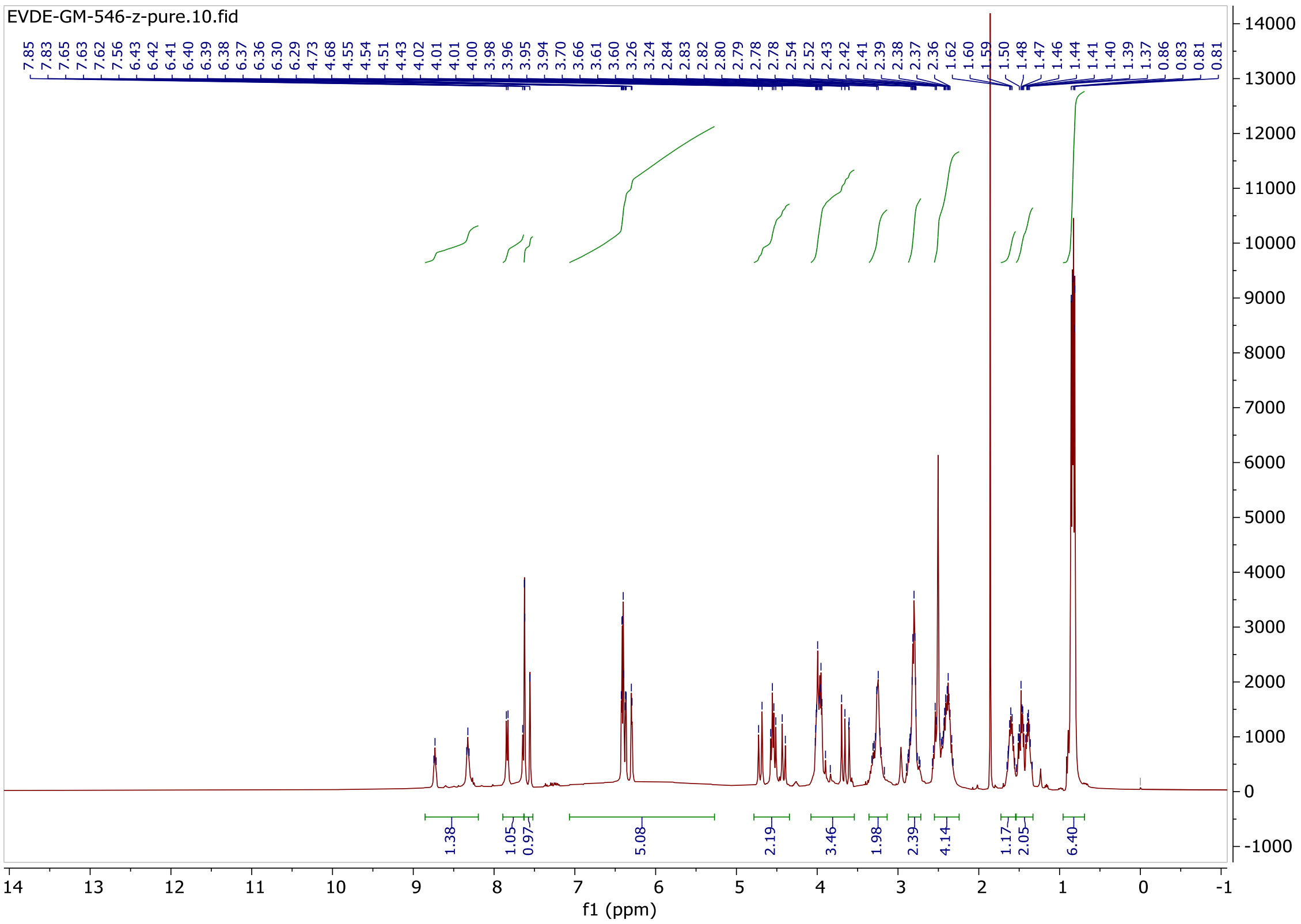

Figure S95. ^{1}H-NMR spectrum of compound **46.**

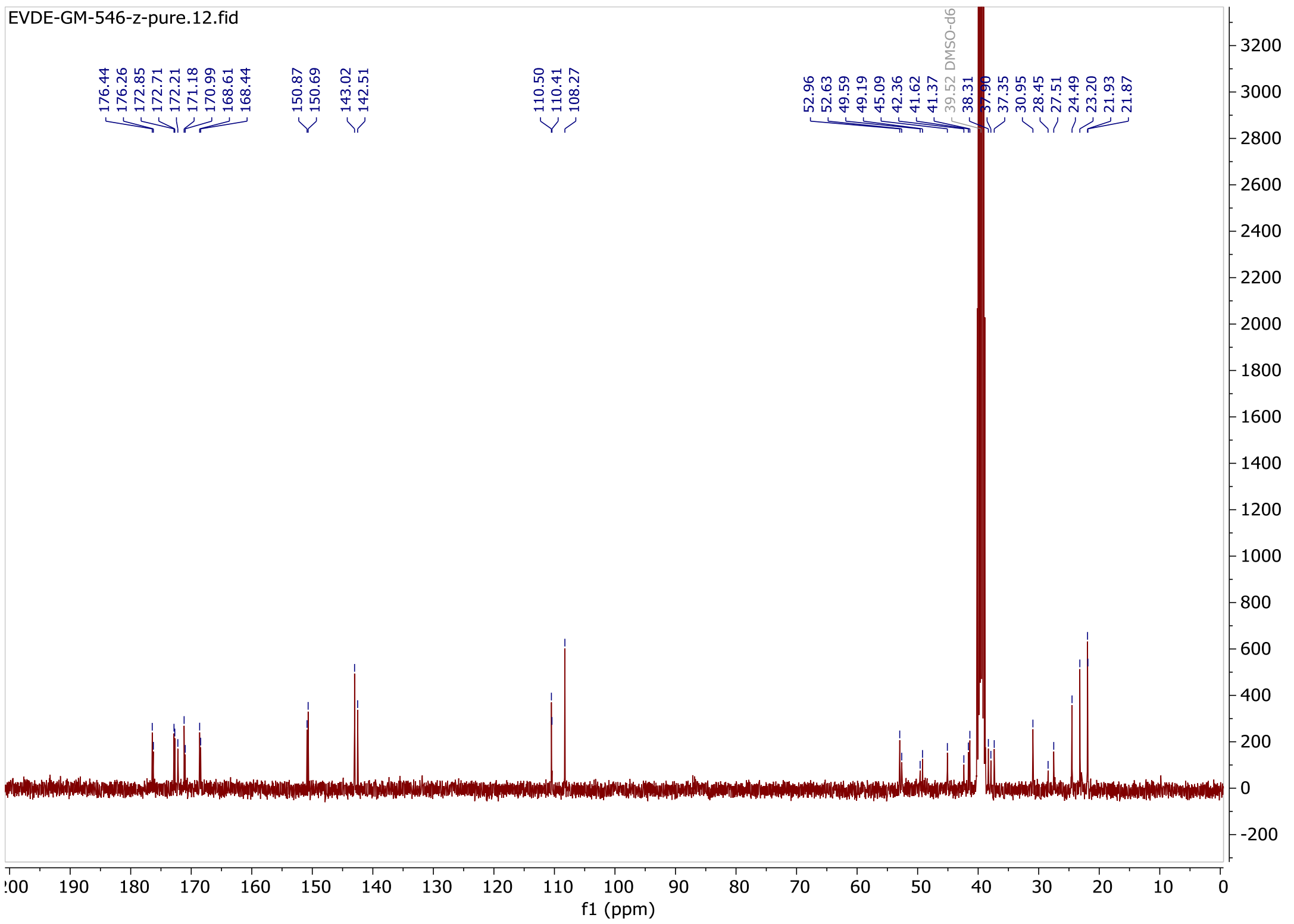

Figure S96. ^{13}C-NMR spectrum of compound **46.**

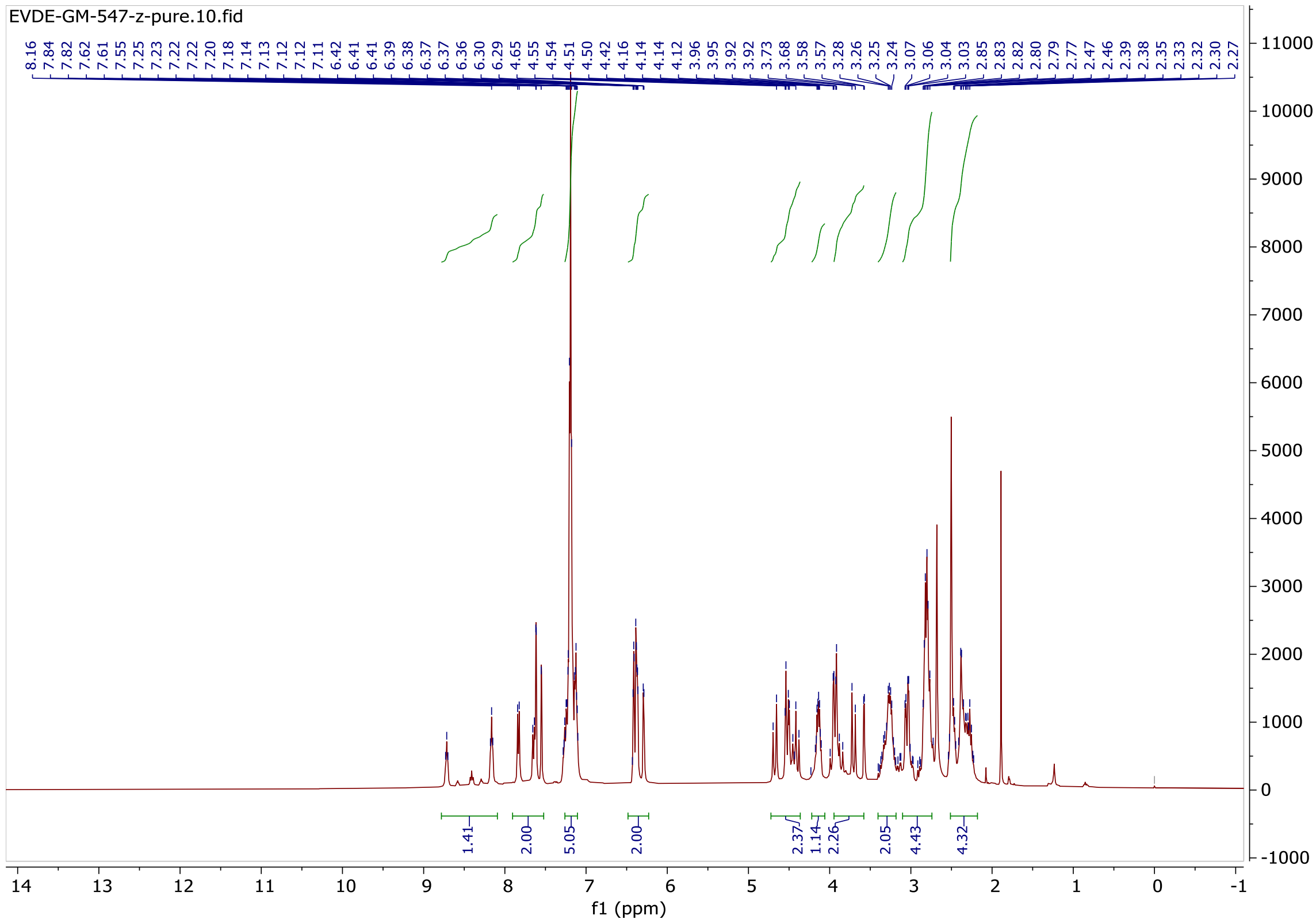

Figure S97. ^{1}H-NMR spectrum of compound **47.**

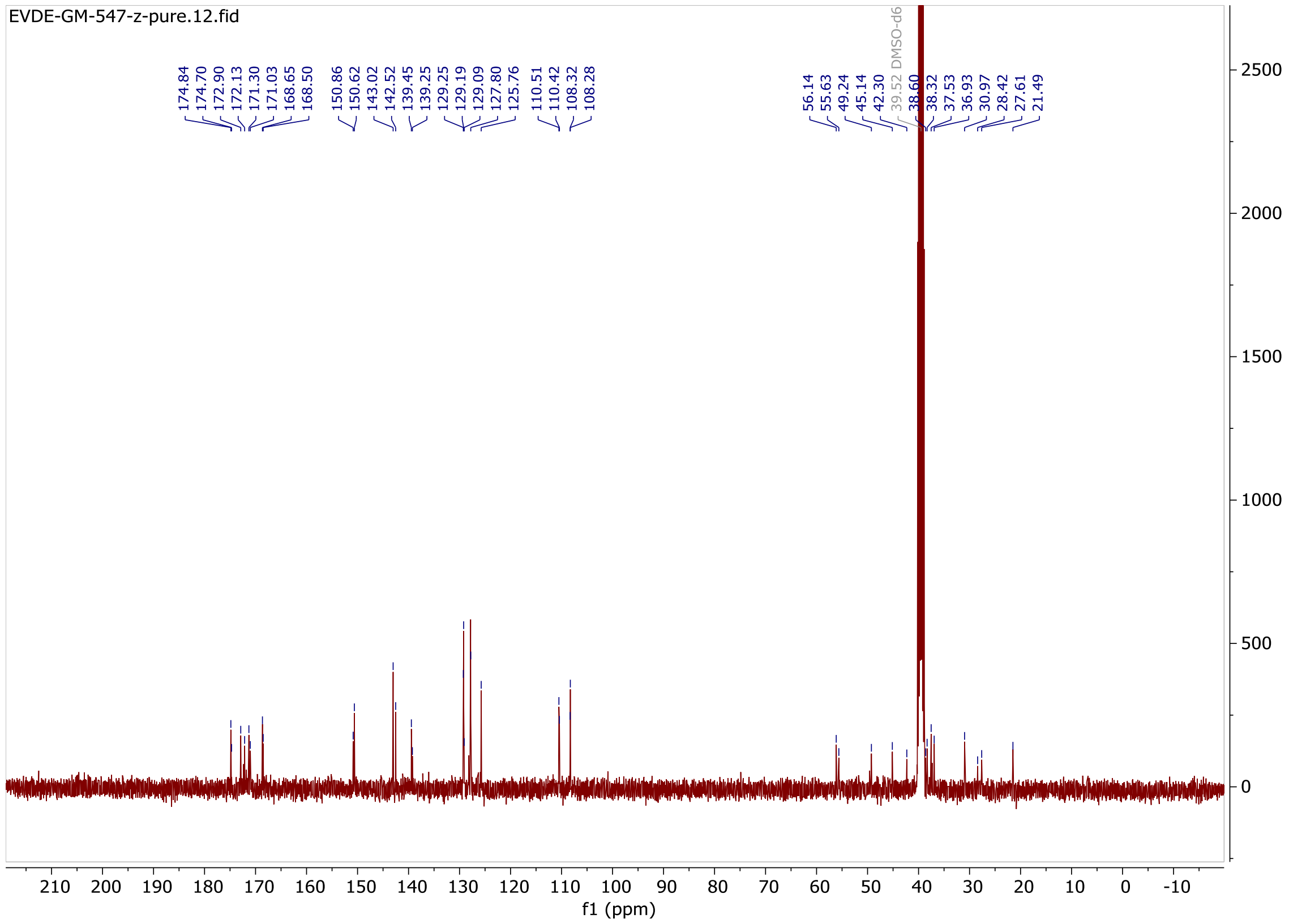

Figure S98. ^{13}C-NMR spectrum of compound **47.**

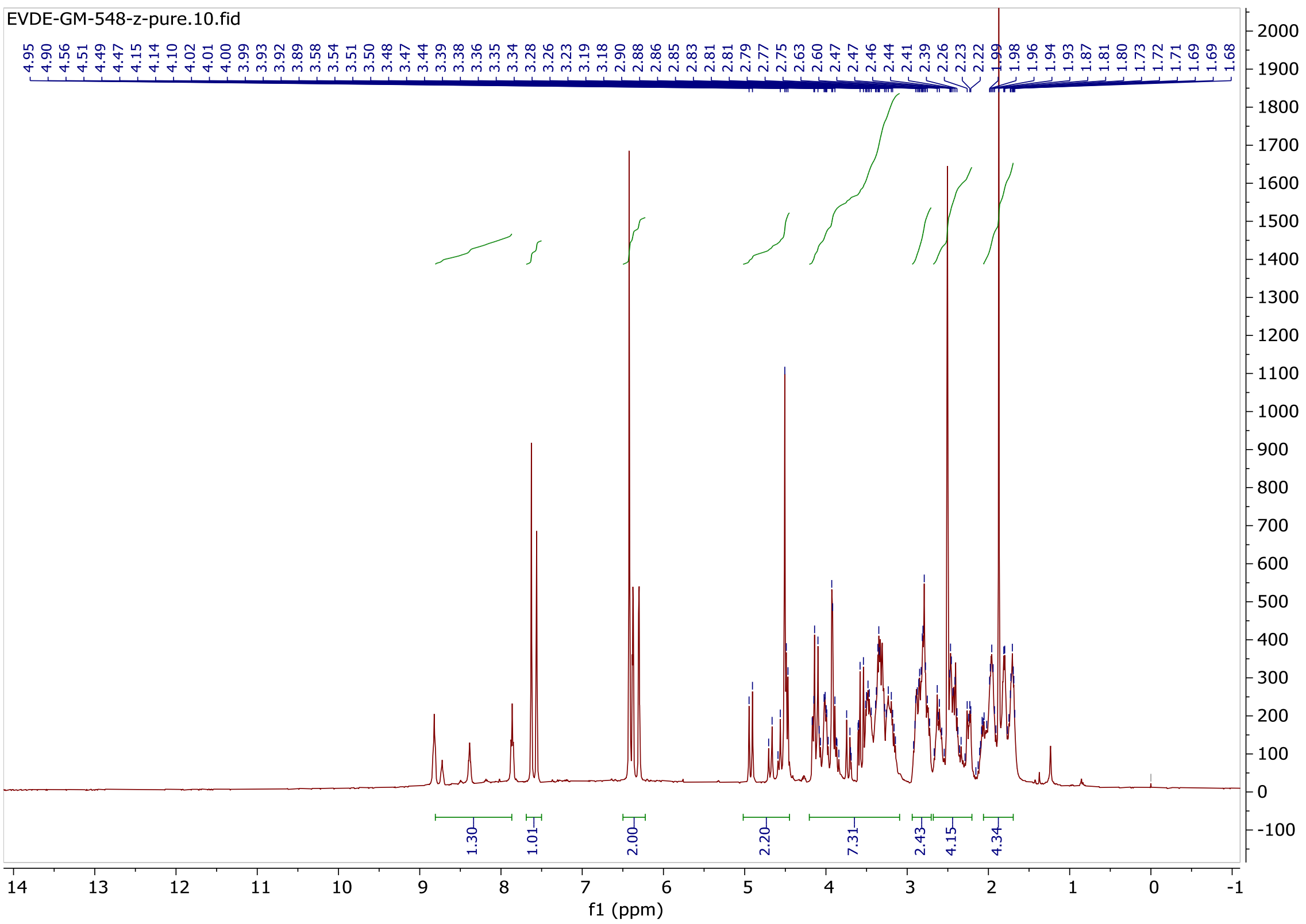

Figure S99. ^{1}H-NMR spectrum of compound **48.**

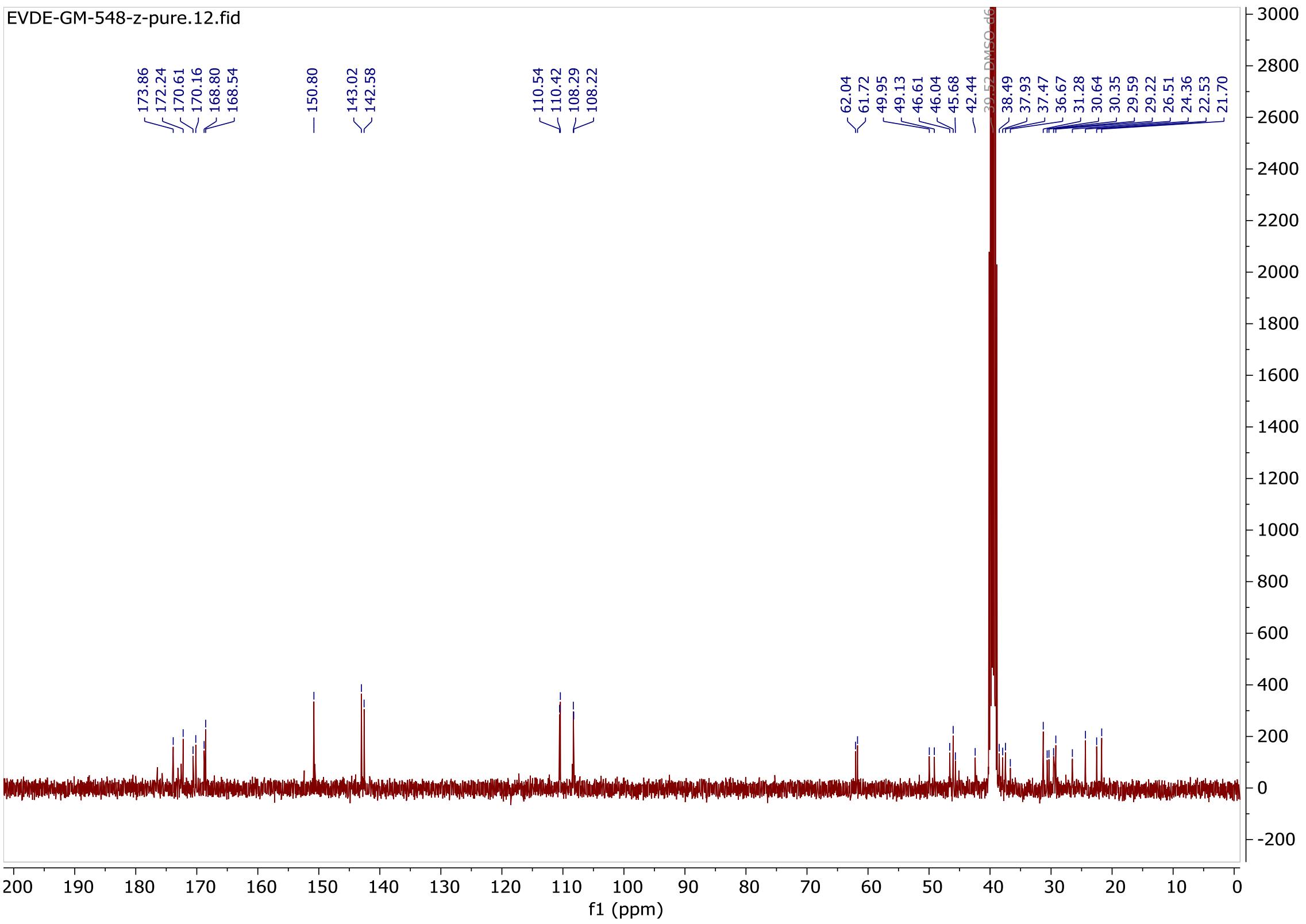

Figure S100. ^{13}C-NMR spectrum of compound **48.**

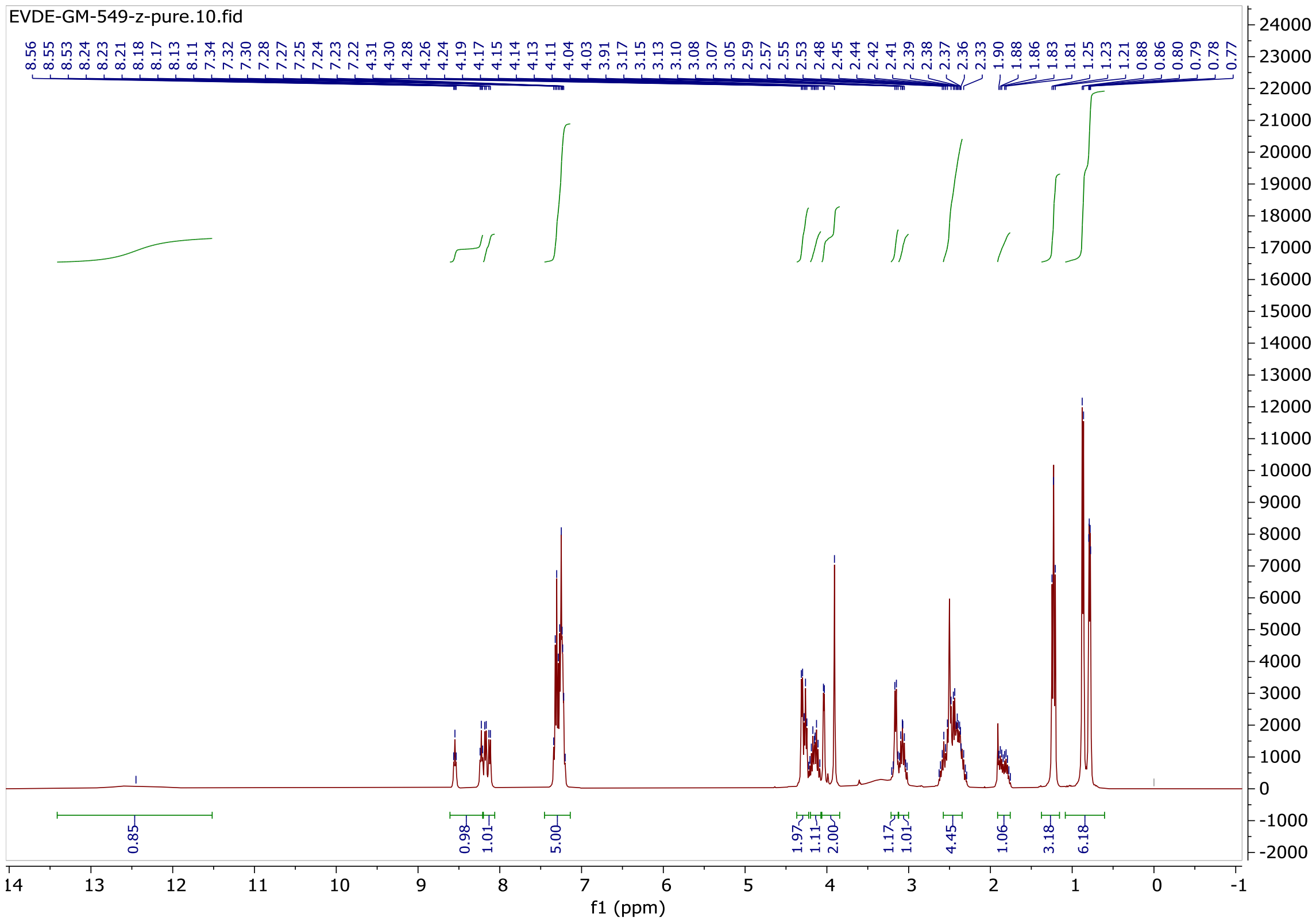

Figure S101. ^{1}H-NMR spectrum of compound **49.**

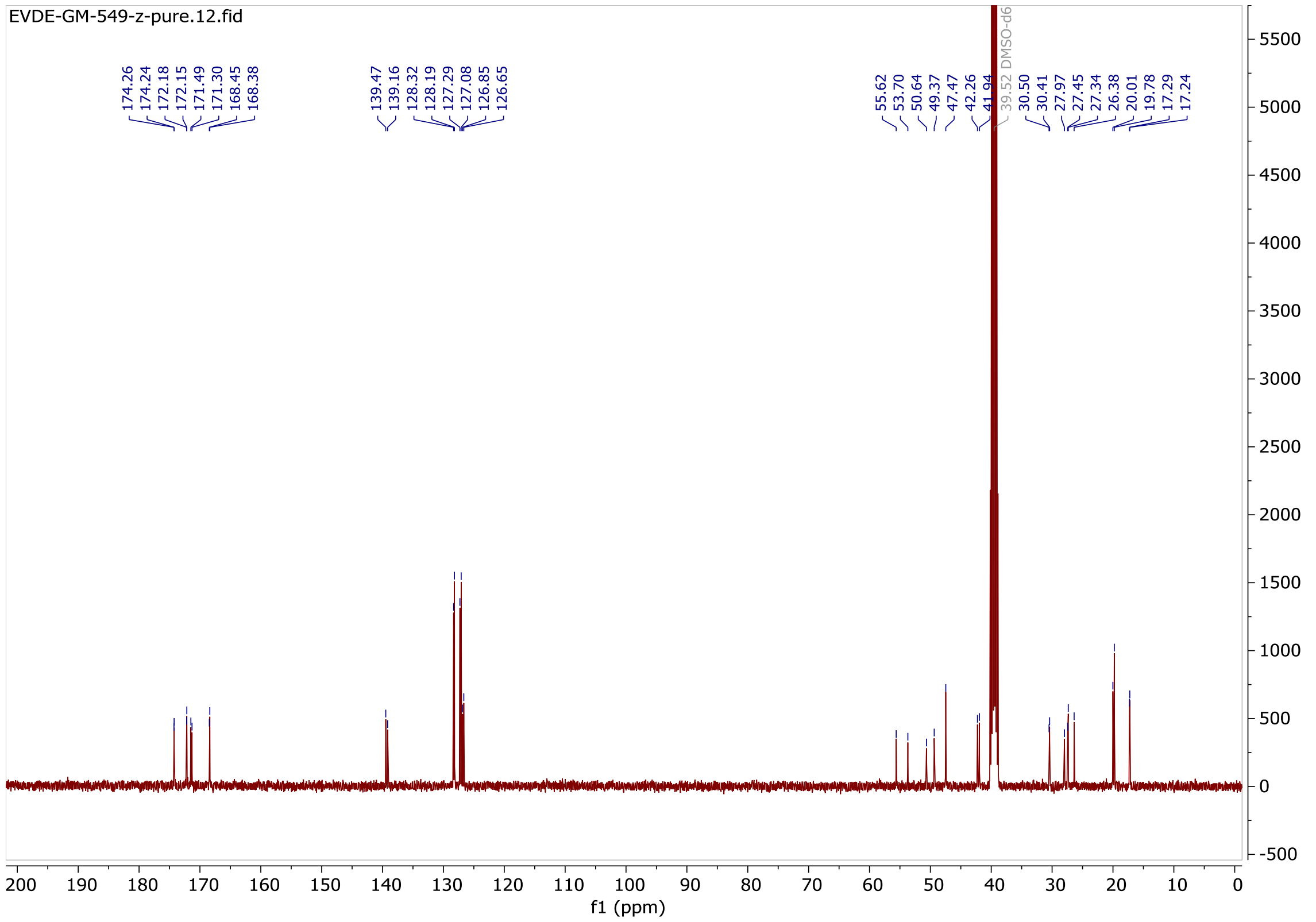

Figure S102. ^{13}C-NMR spectrum of compound **49.**

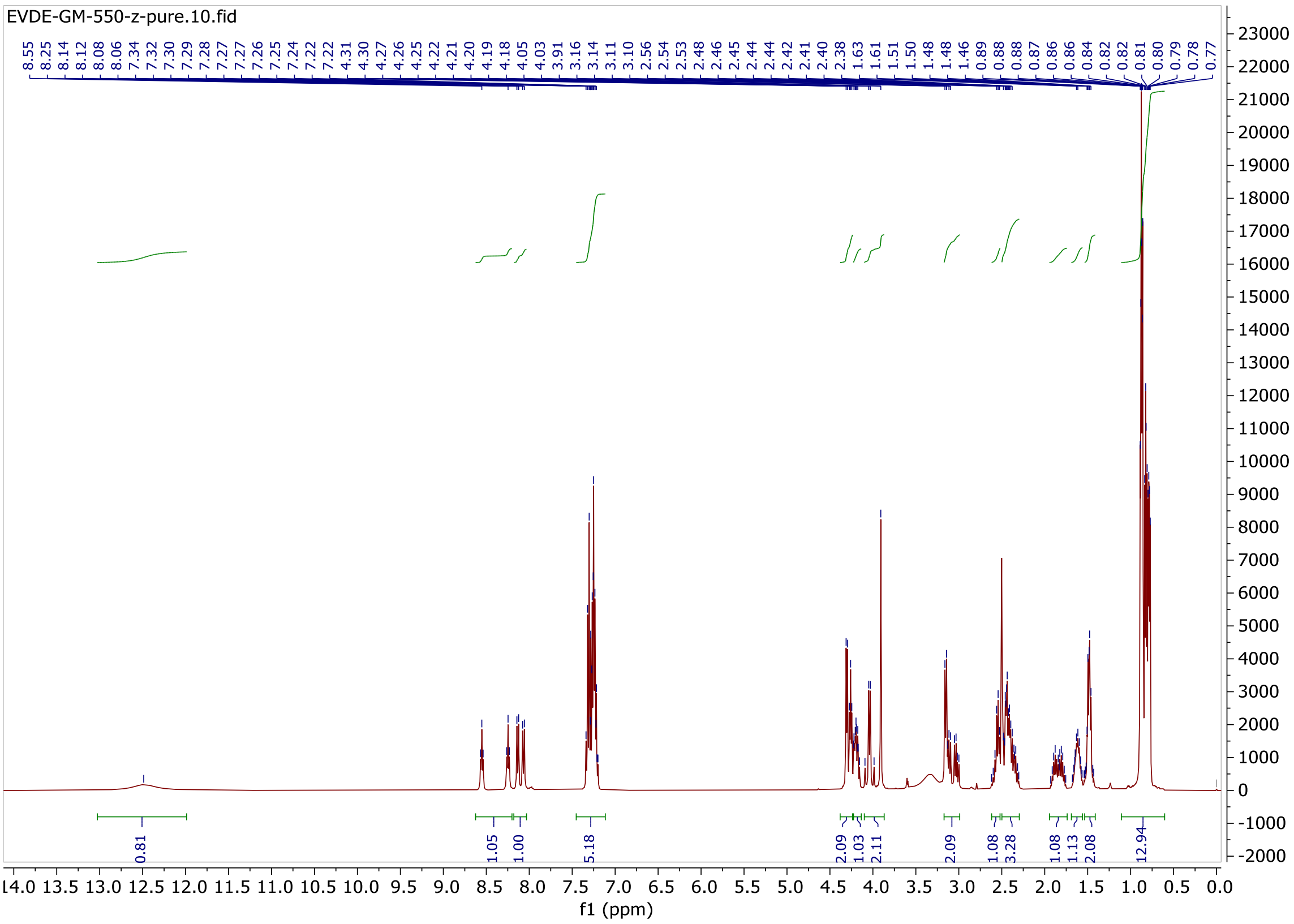

Figure S103. ^{1}H-NMR spectrum of compound **50.**

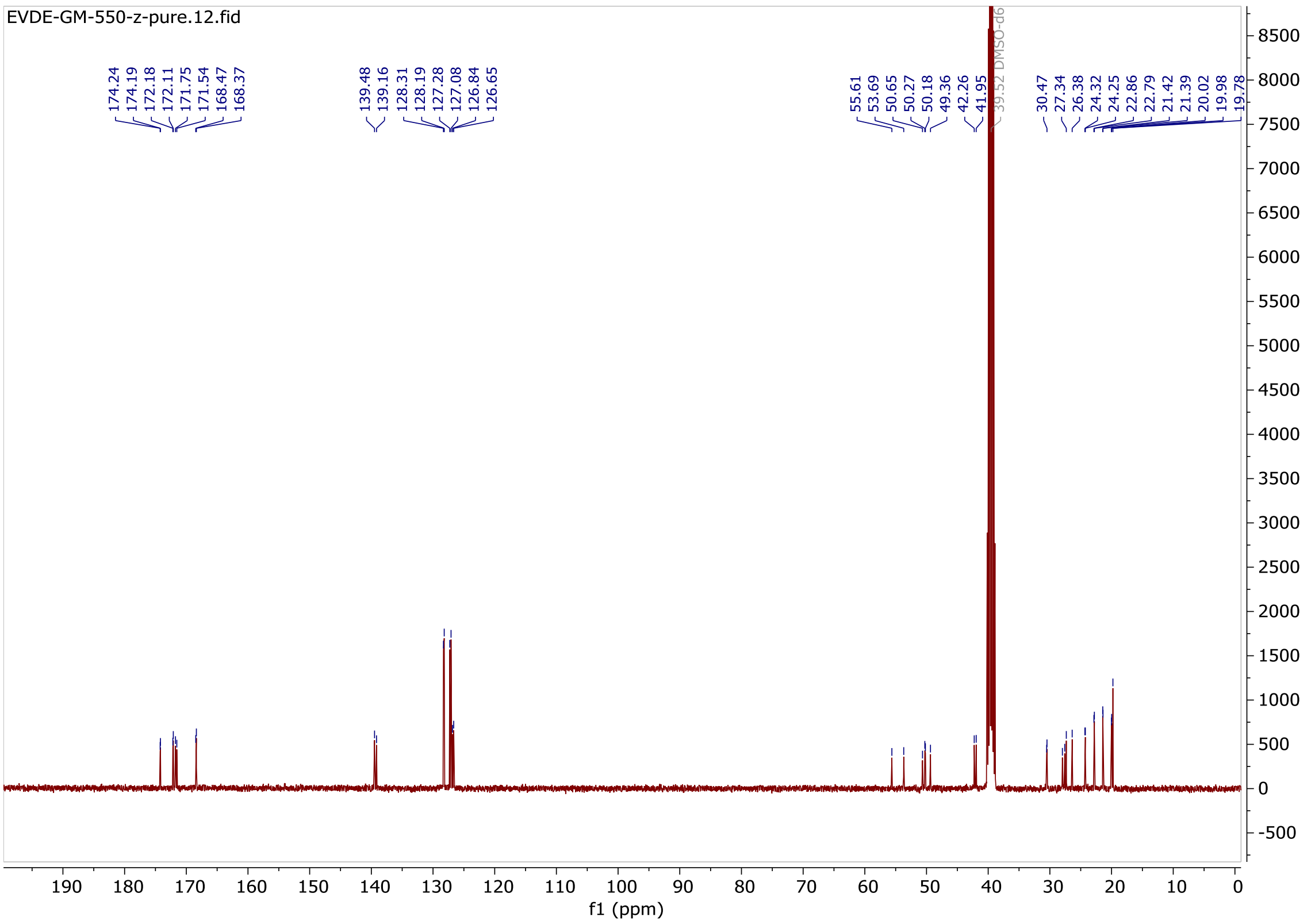

Figure S104. ^{13}C-NMR spectrum of compound **50.**

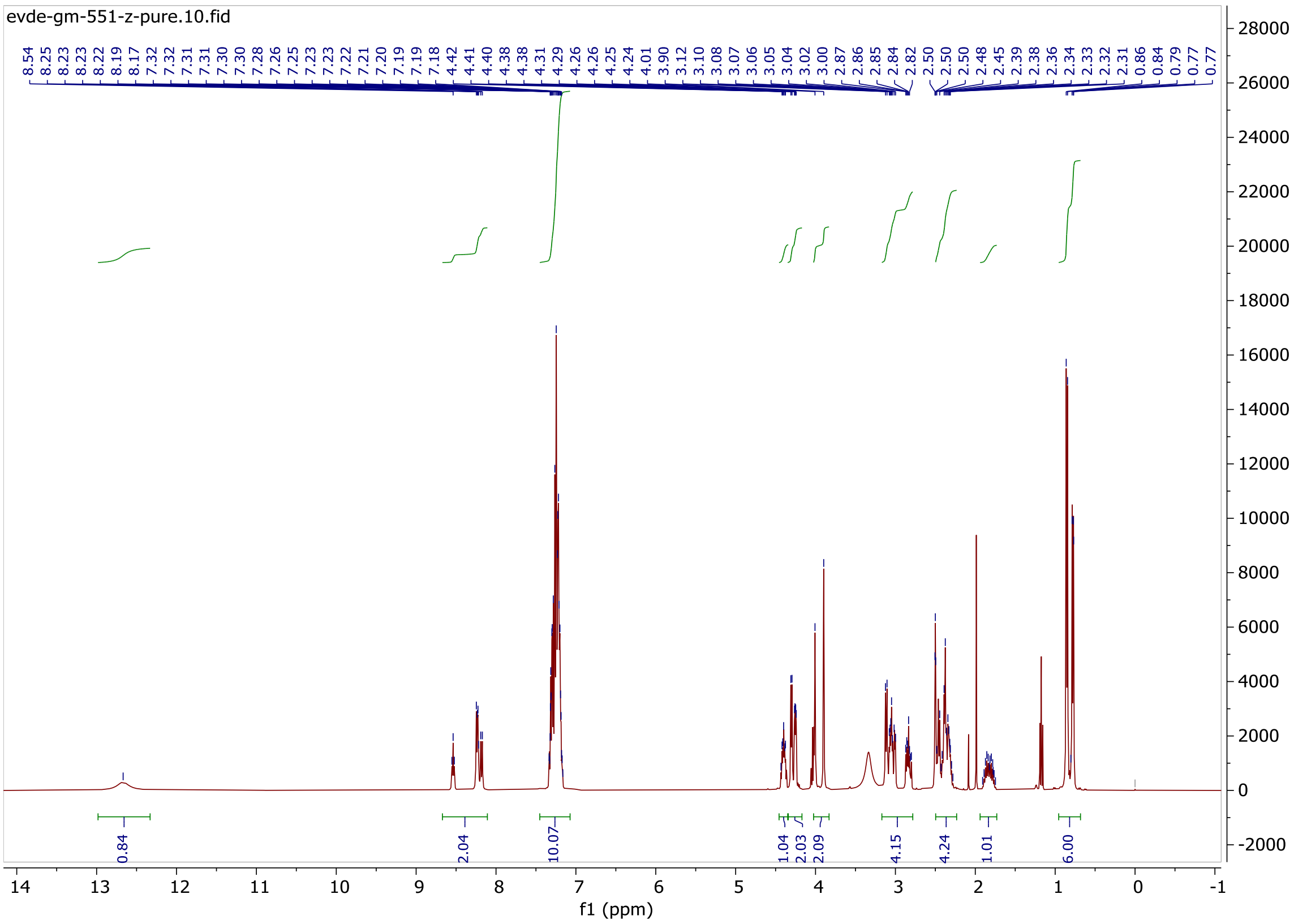

Figure S105. ^{1}H-NMR spectrum of compound **51.**

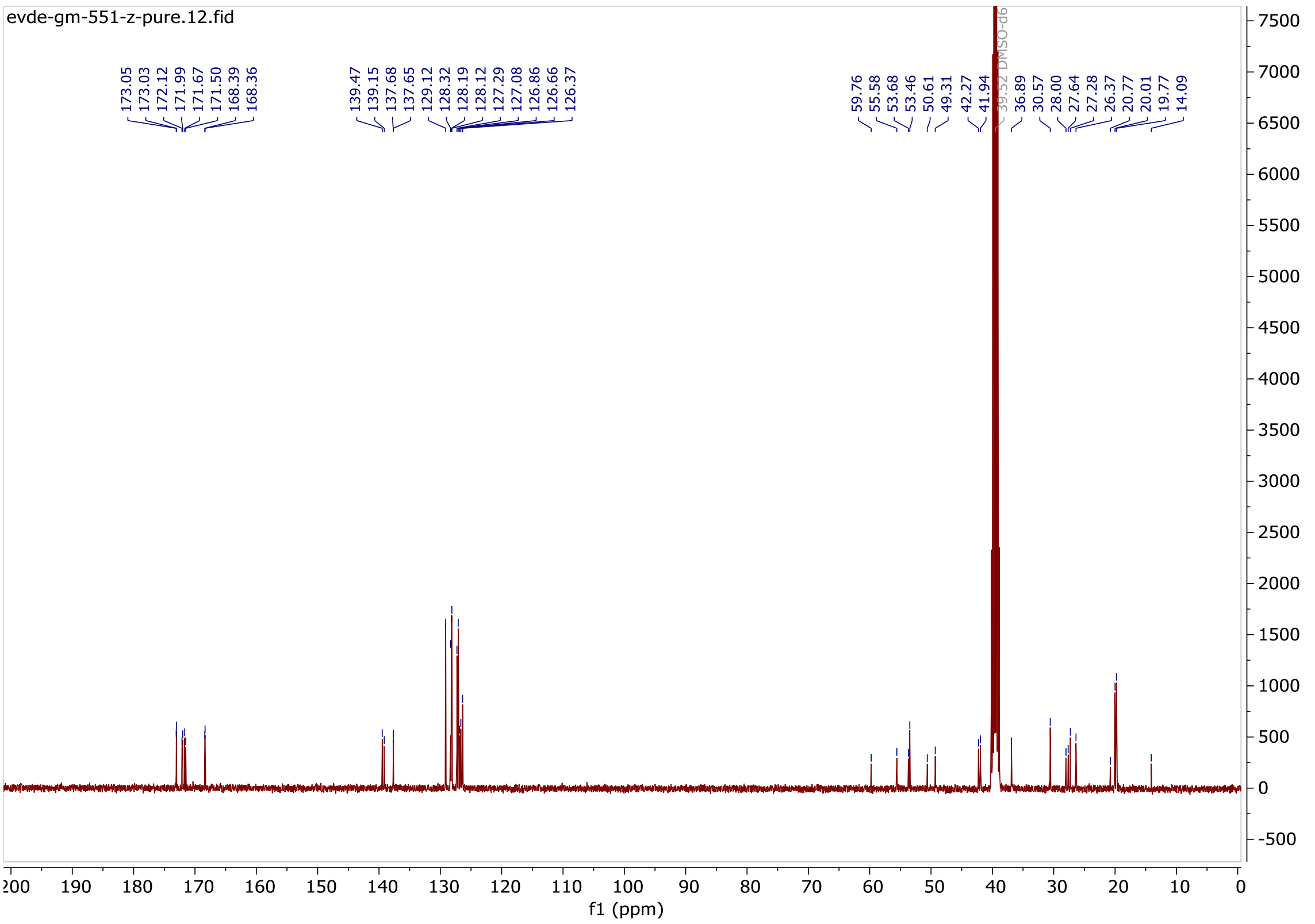

Figure S106. ^{13}C-NMR spectrum of compound **51.**

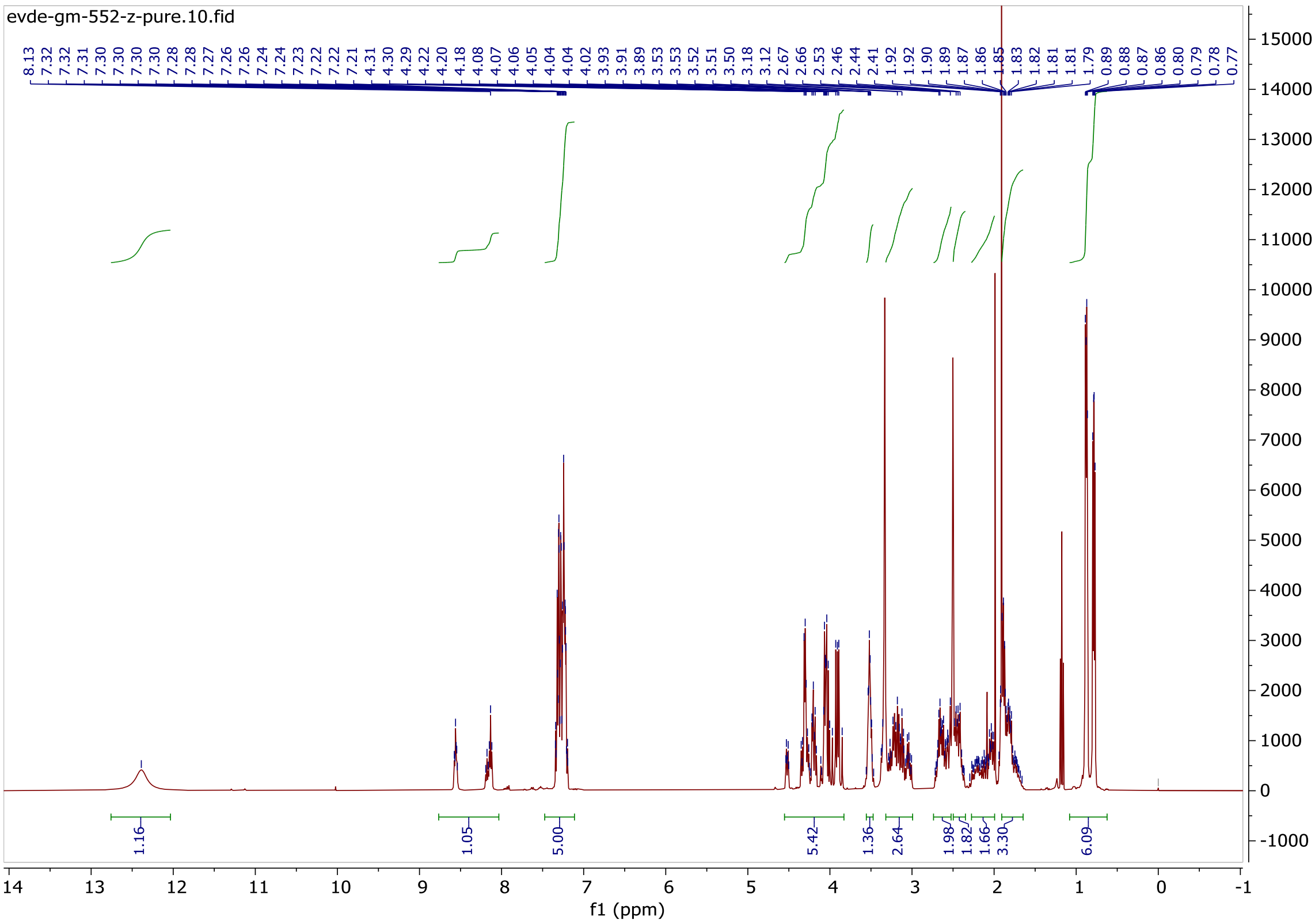

Figure S107. ^{1}H-NMR spectrum of compound **52.**

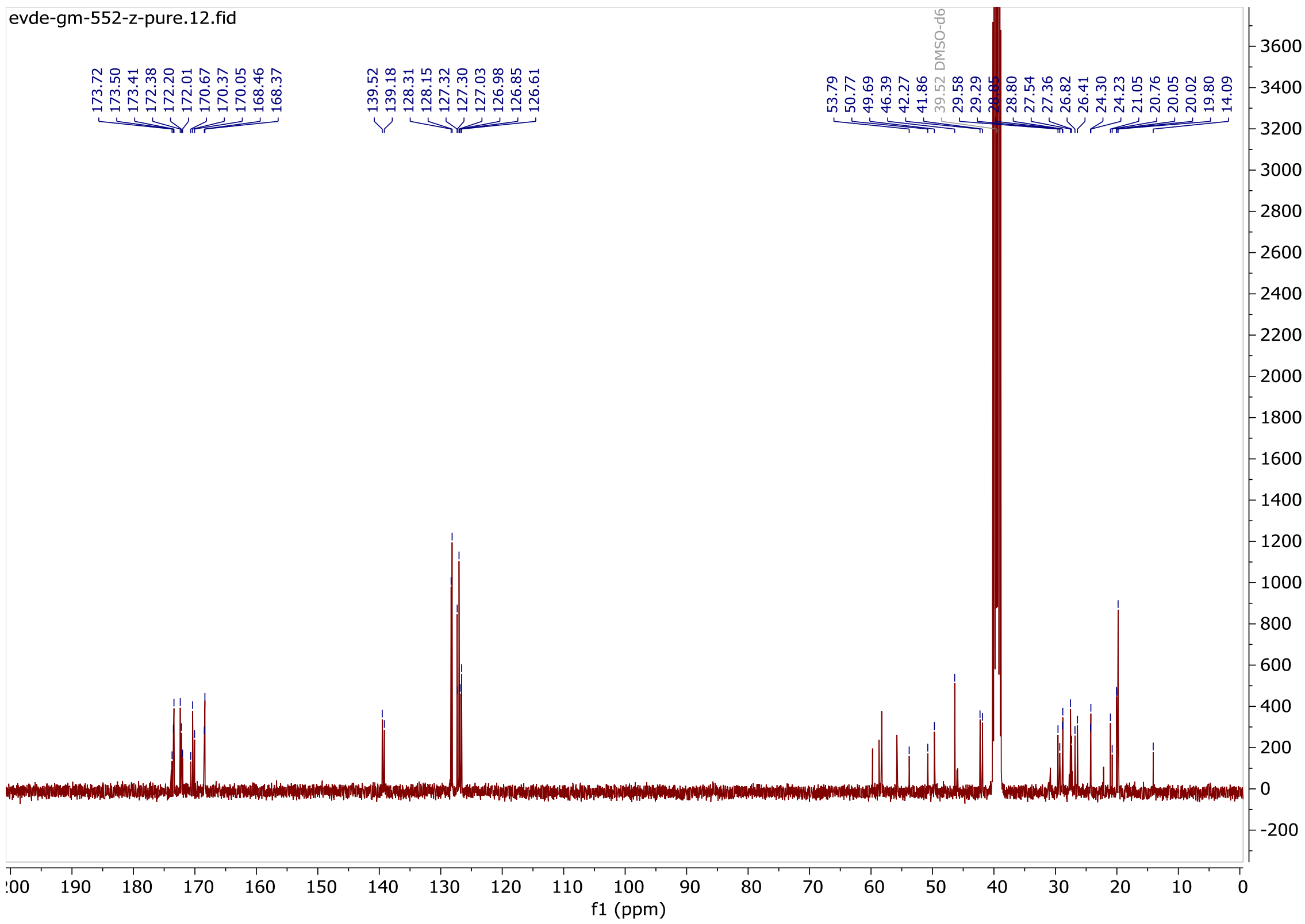

Figure S108. ^{13}C-NMR spectrum of compound **52.**

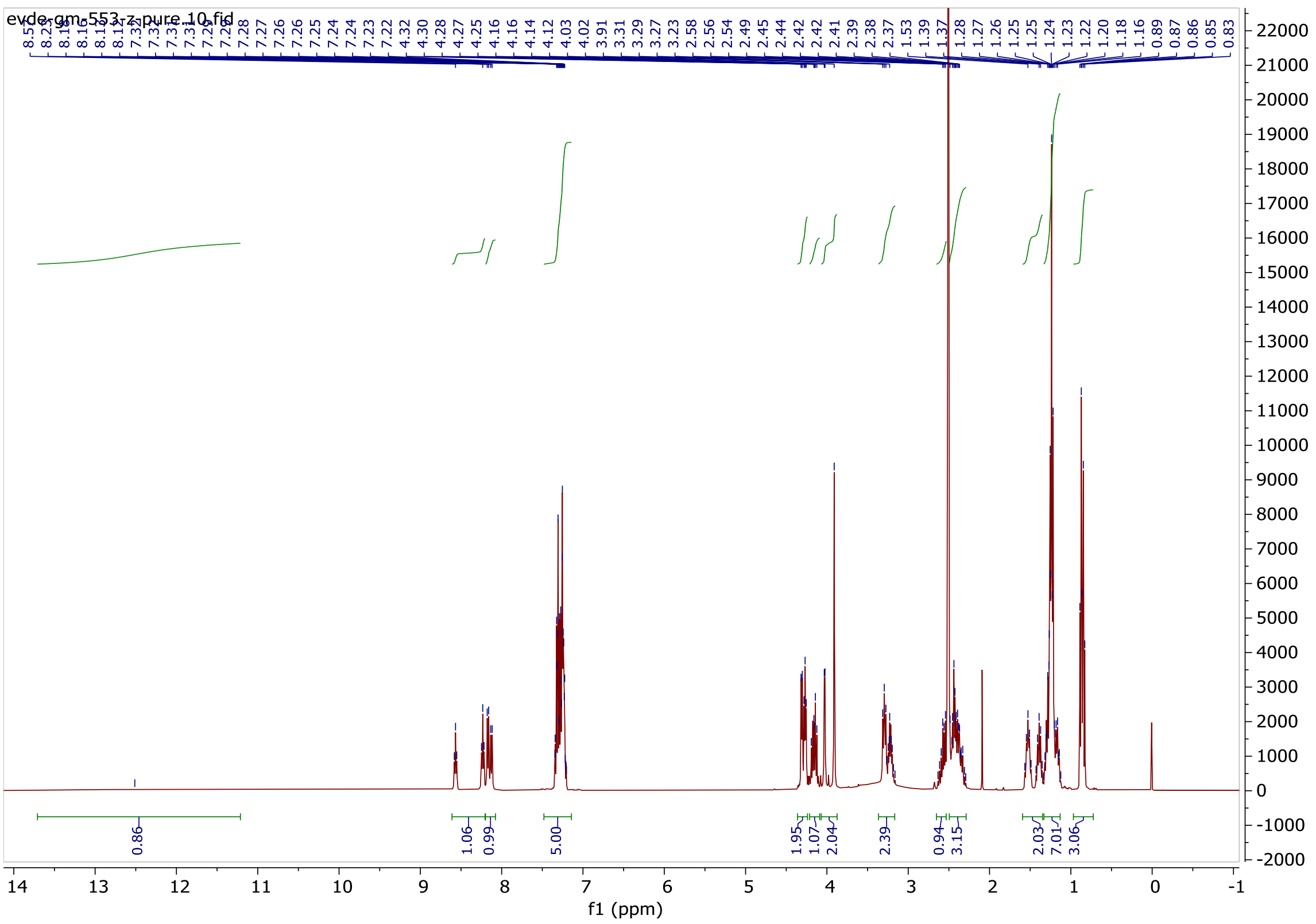

Figure S109. ^{1}H-NMR spectrum of compound **53.**

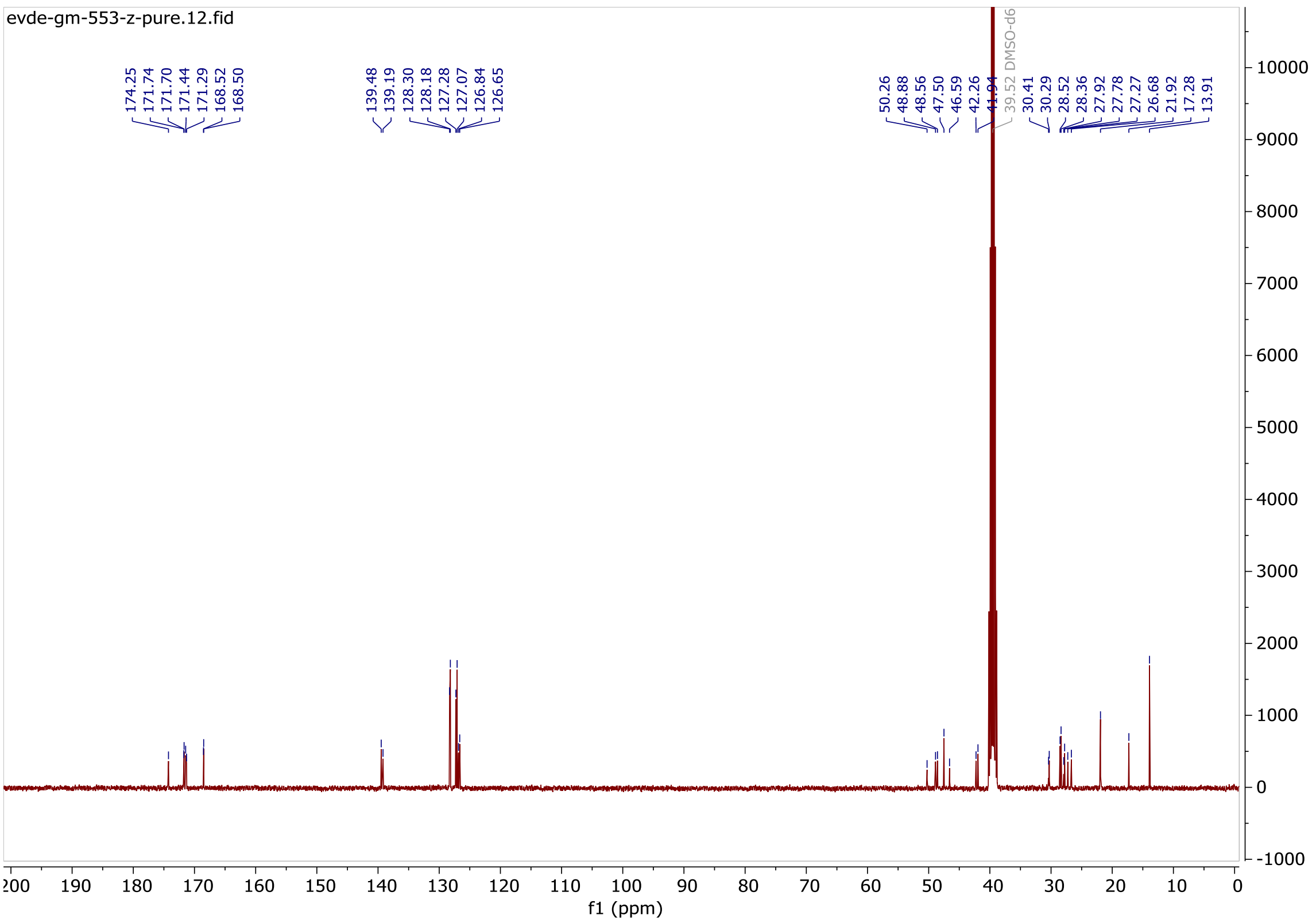

Figure S110. ^{13}C-NMR spectrum of compound **53.**

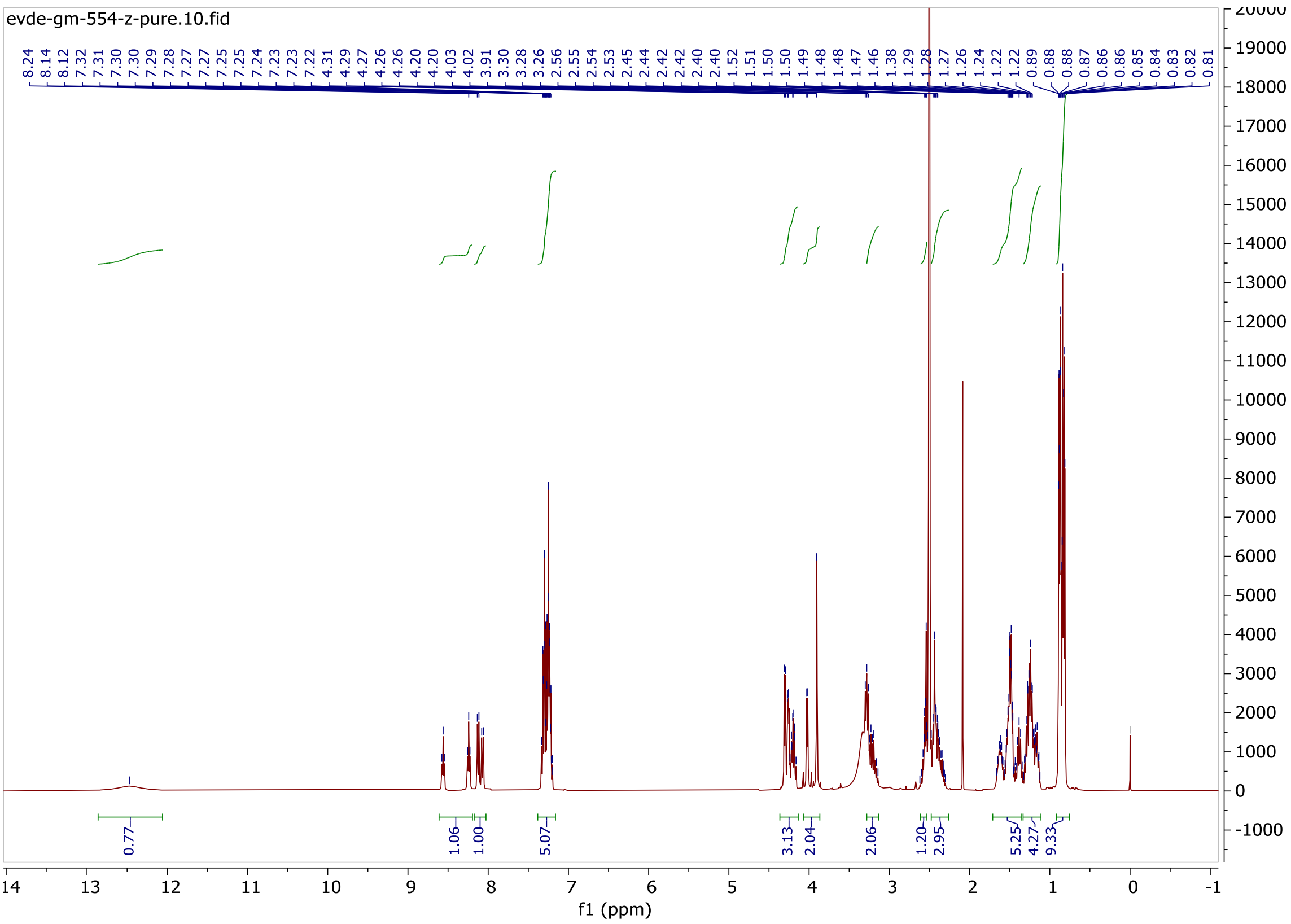

Figure S111. ^{1}H-NMR spectrum of compound **54.**

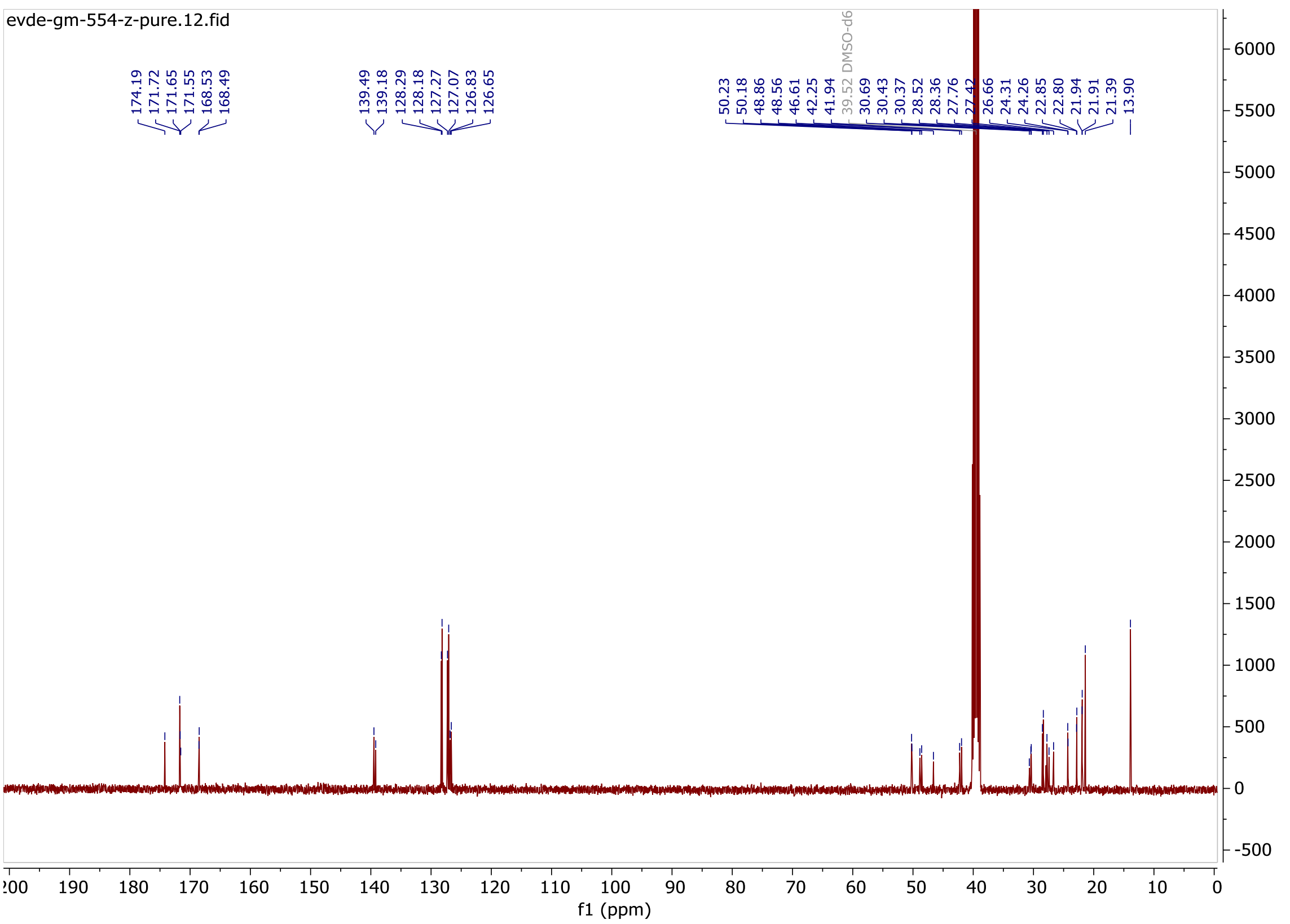

Figure S112. ^{13}C-NMR spectrum of compound **54.**

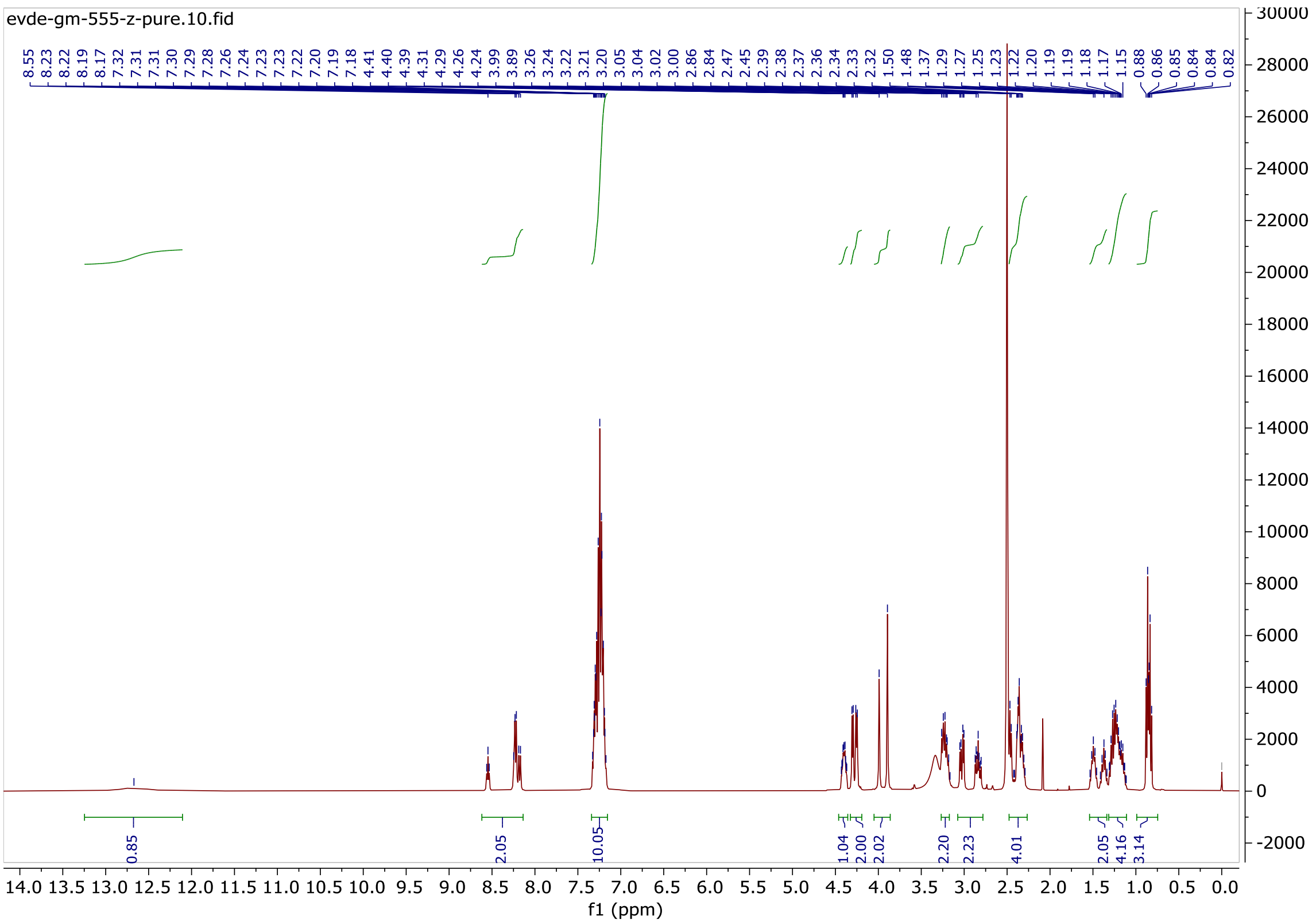

Figure S113. ^{1}H-NMR spectrum of compound **55.**

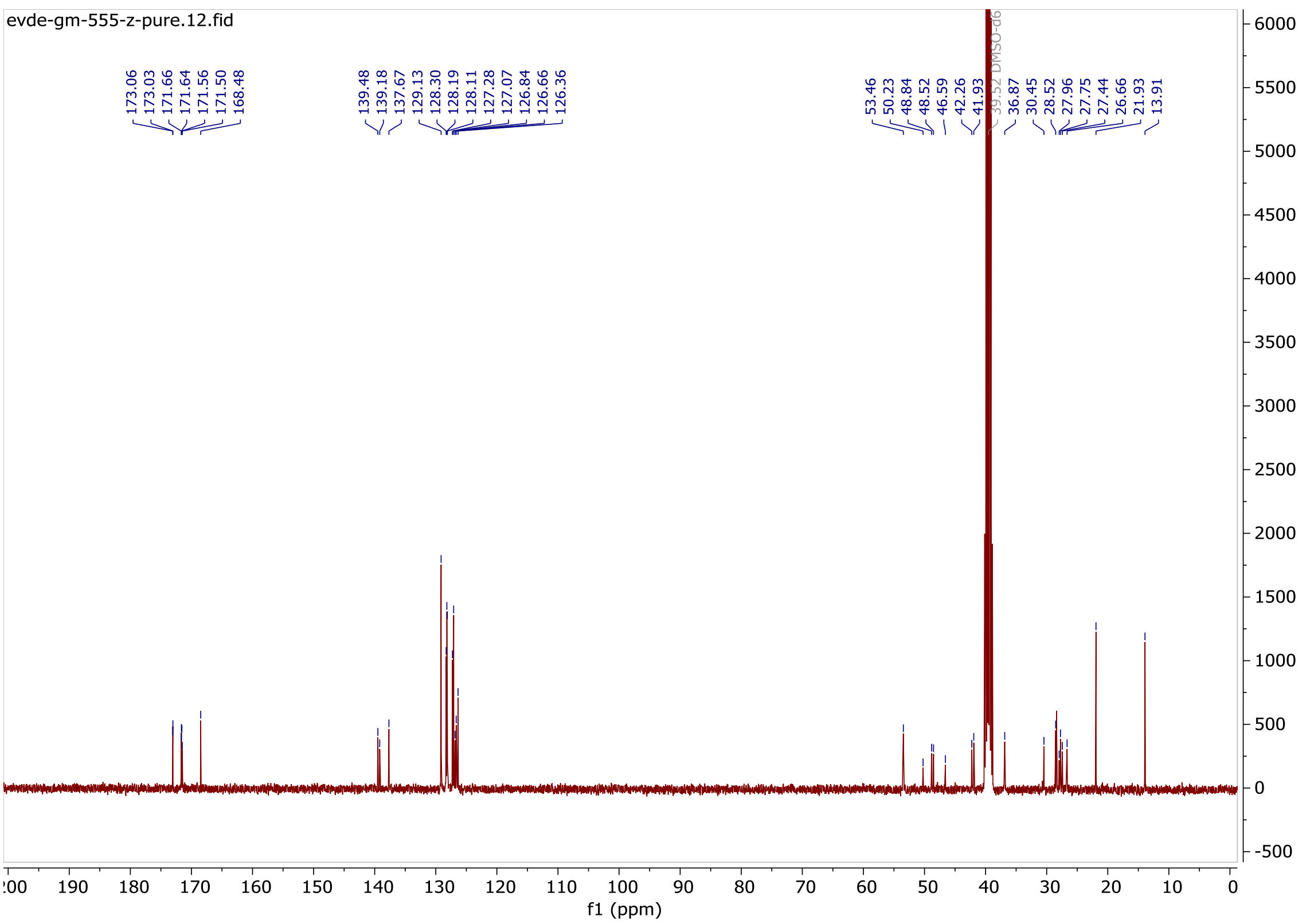

Figure S114. ^{13}C-NMR spectrum of compound **55.**

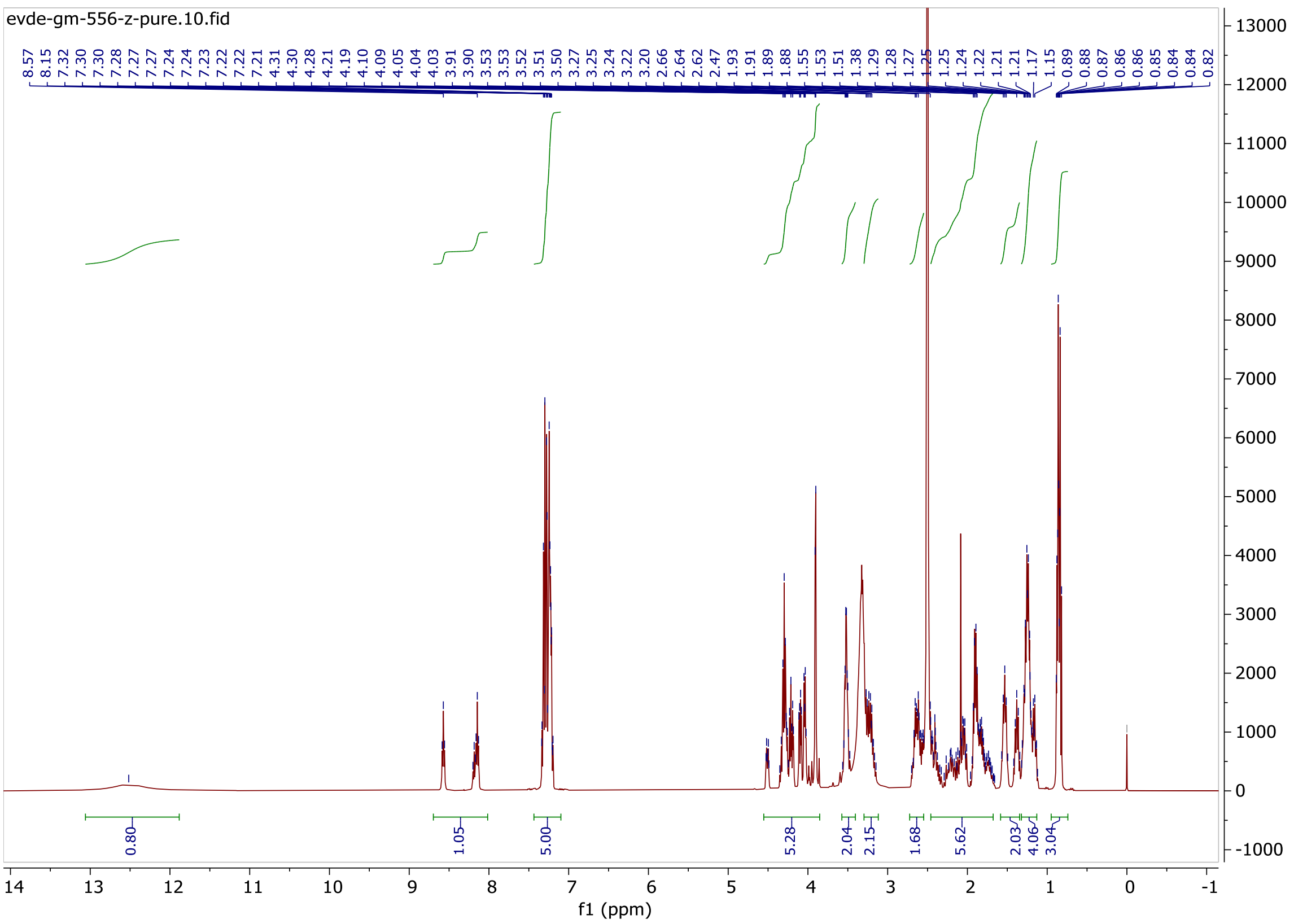

Figure S115. ^{1}H-NMR spectrum of compound **56.**

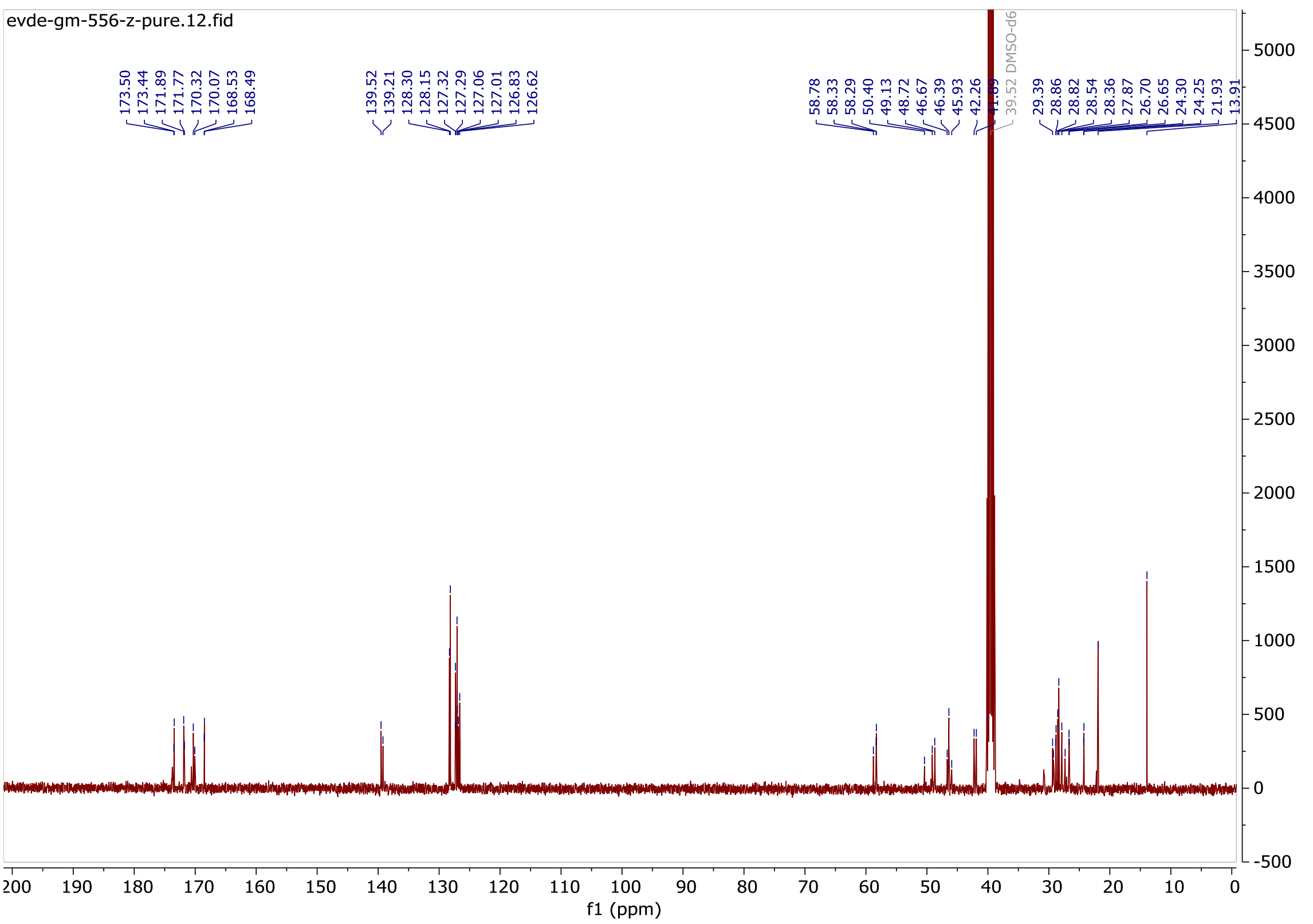

Figure S116. ^{13}C-NMR spectrum of compound **56.**

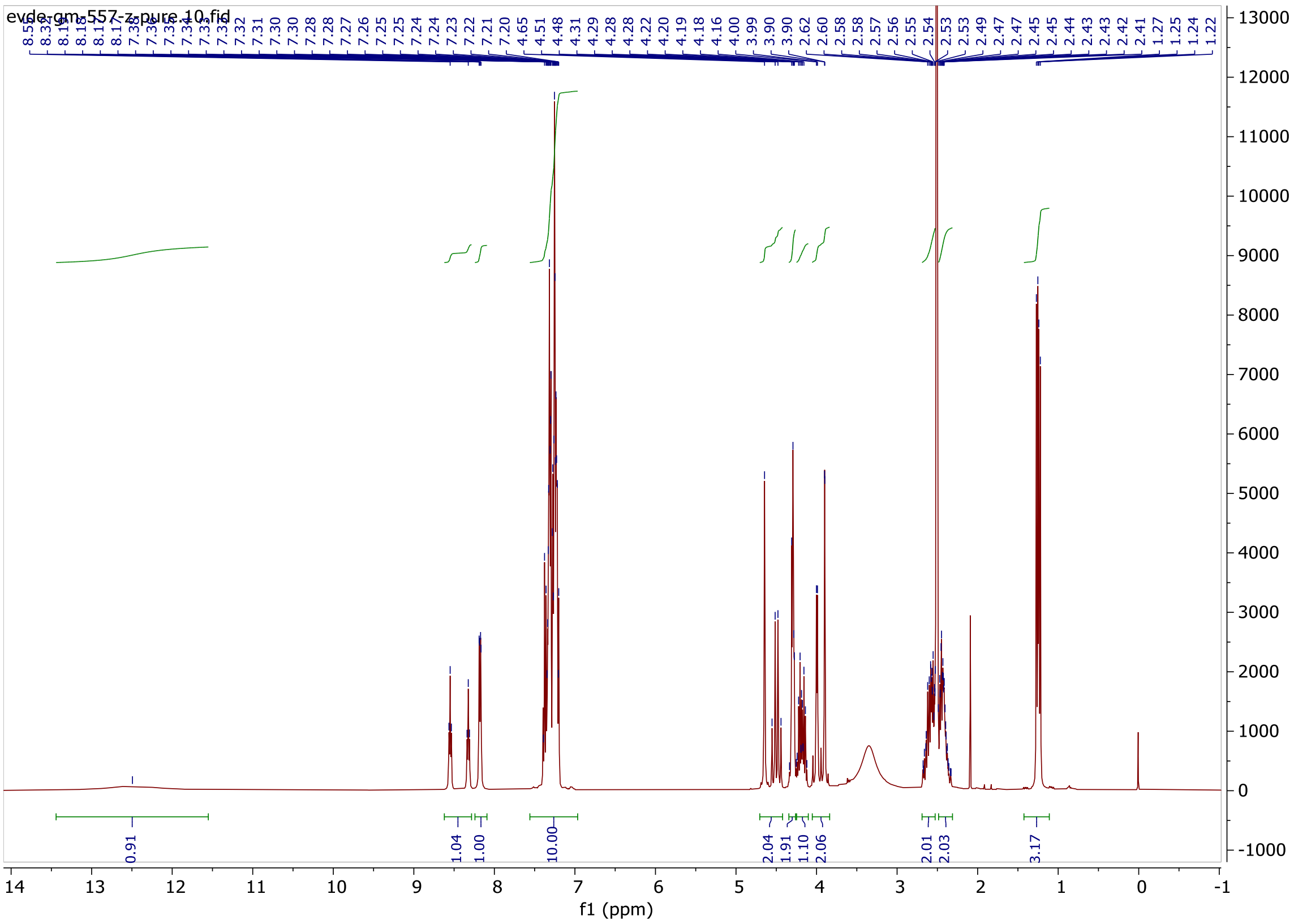

Figure S117. ^{1}H-NMR spectrum of compound **57.**

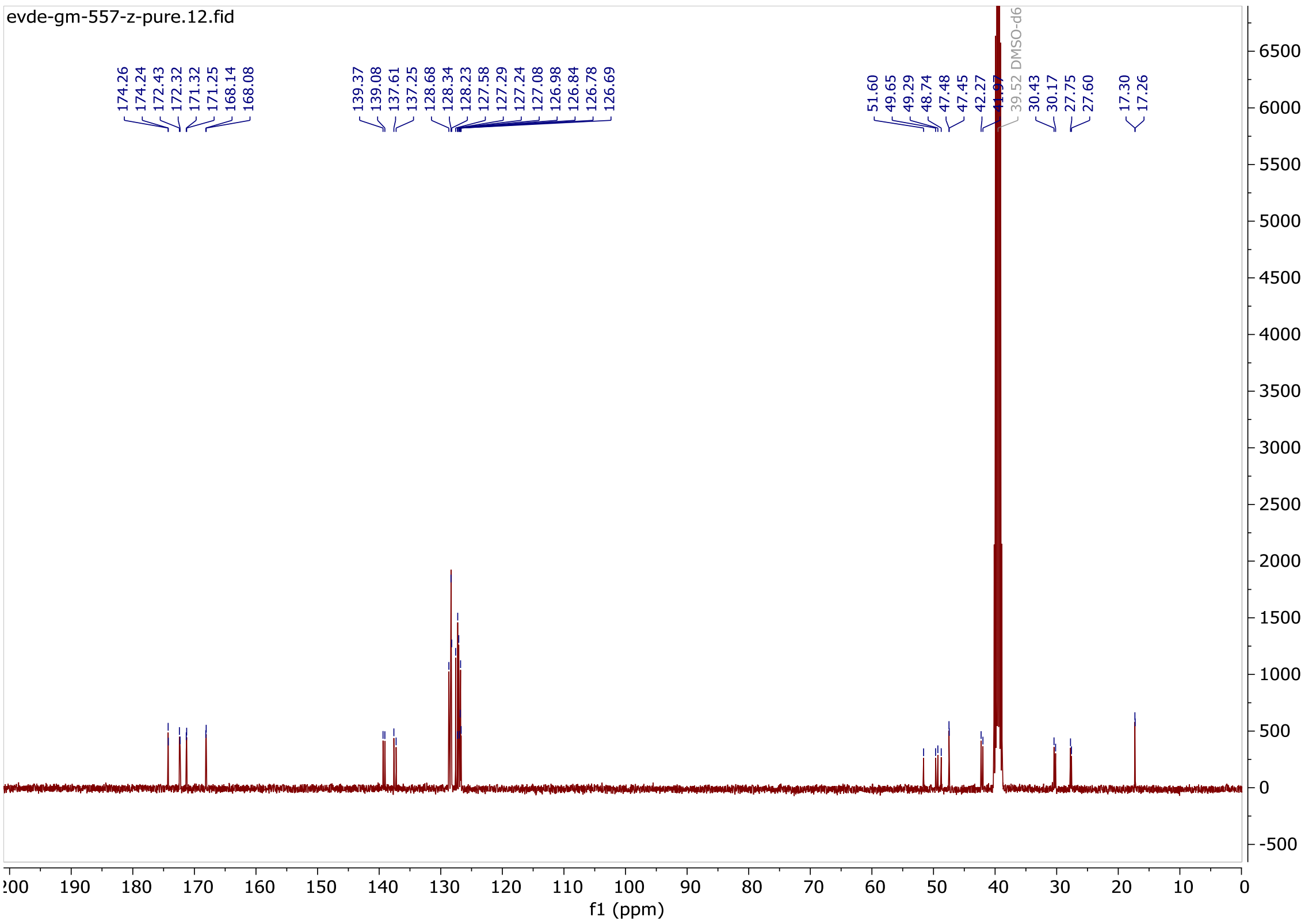

Figure S118. ^{13}C-NMR spectrum of compound **57.**

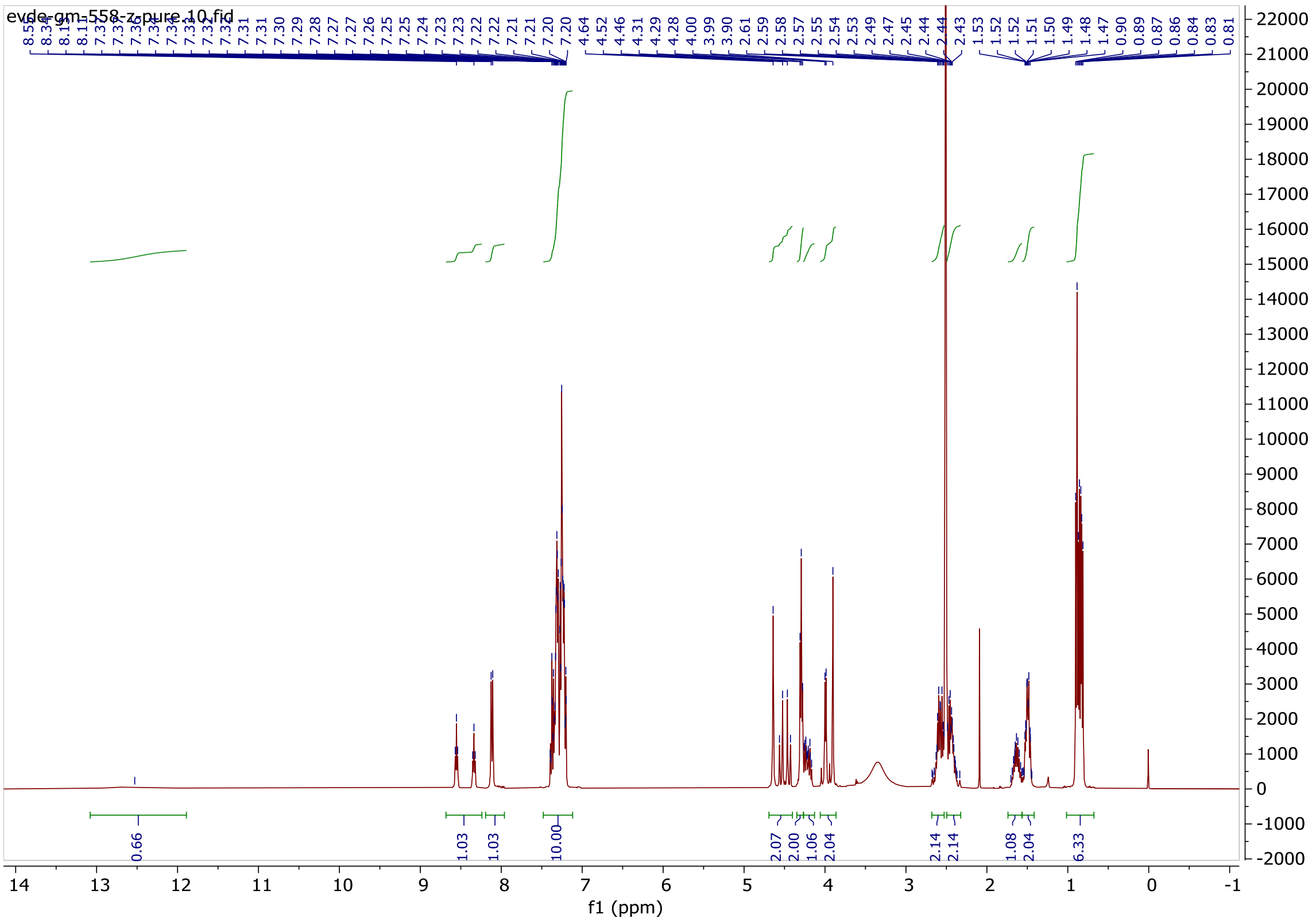

Figure S119. ^{1}H-NMR spectrum of compound **58.**

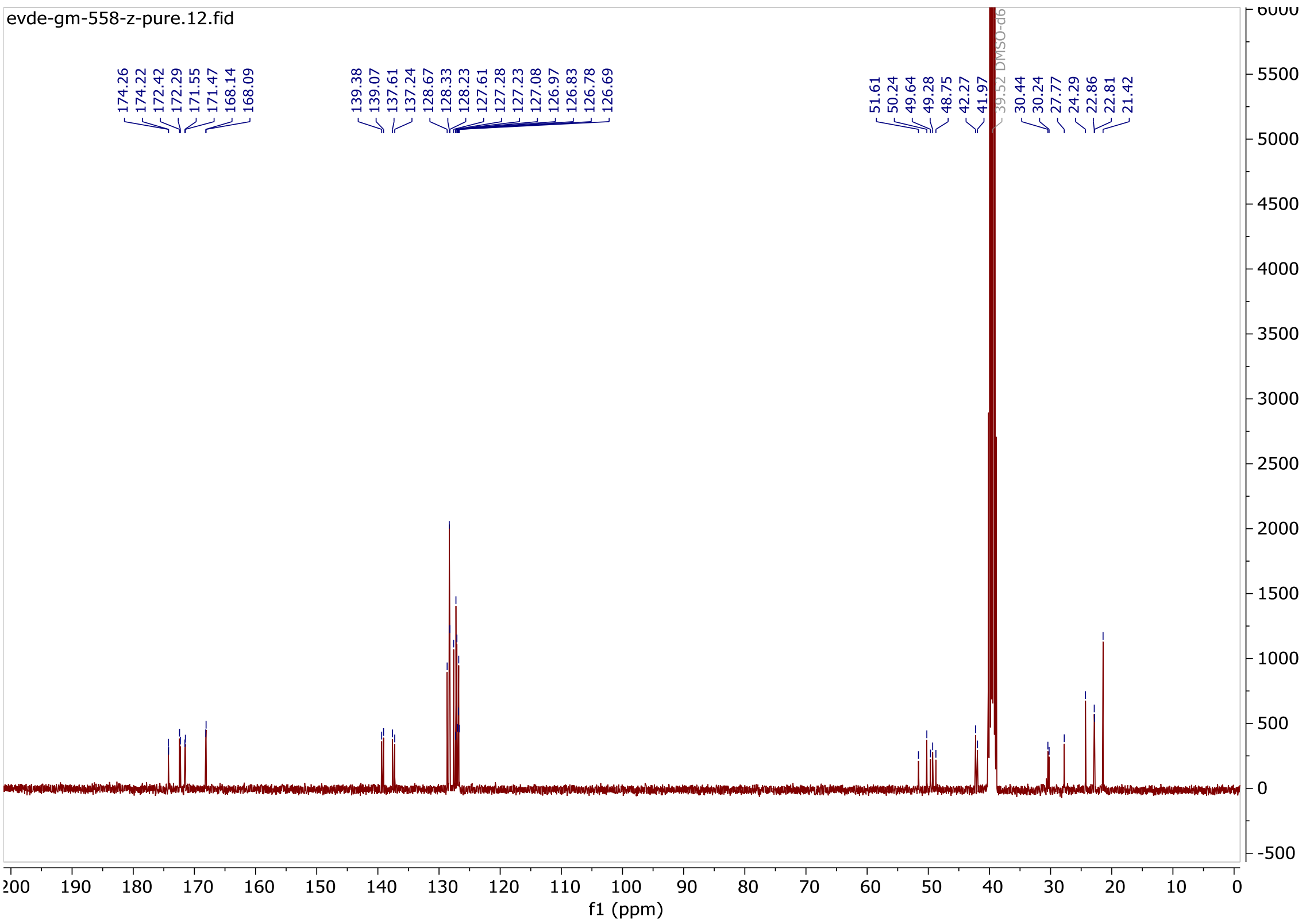

Figure S120. ^{13}C-NMR spectrum of compound **58.**

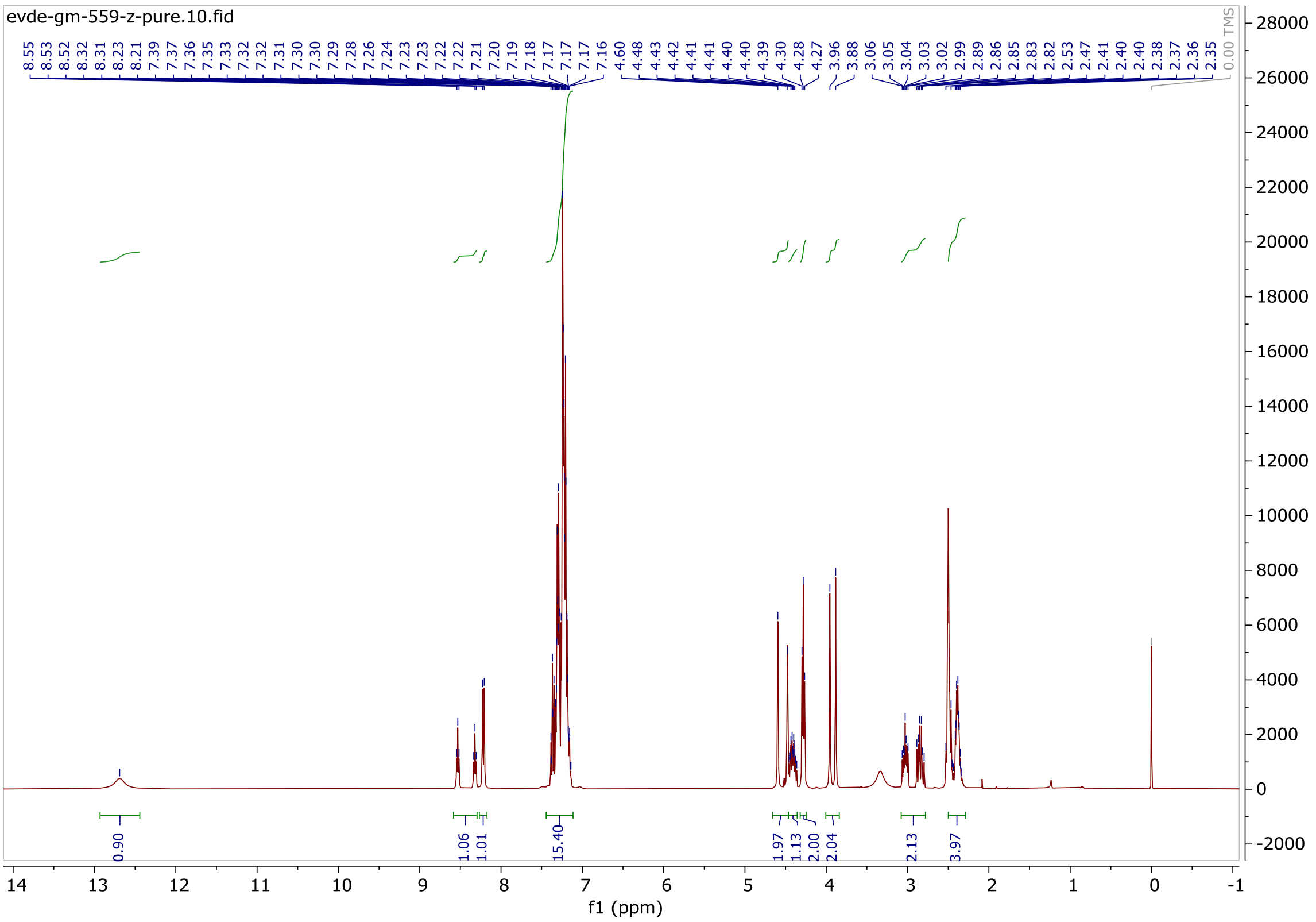

Figure S121. ^{1}H-NMR spectrum of compound **59.**

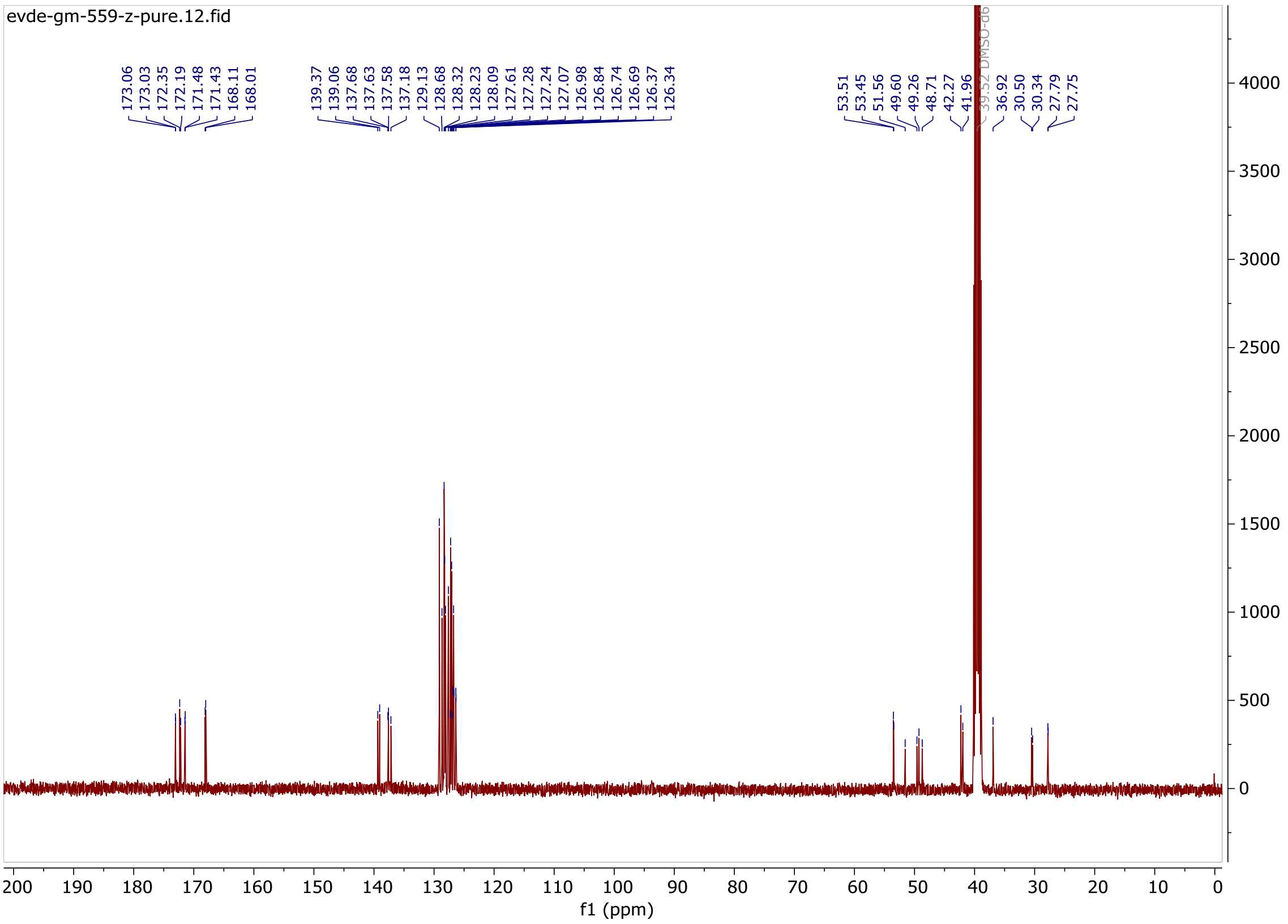

Figure S122. ^{13}C-NMR spectrum of compound **59.**

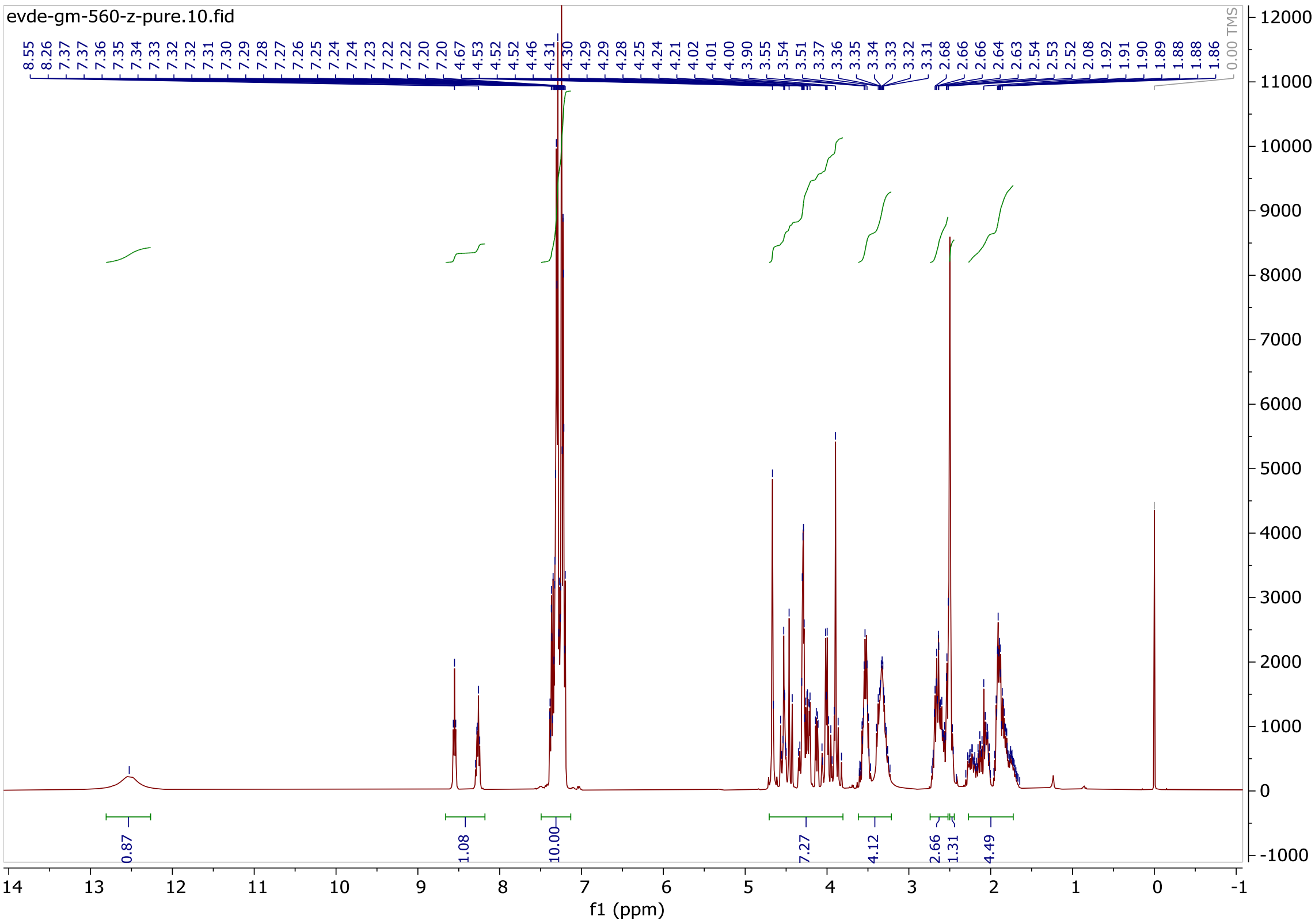

Figure S123. ^{1}H-NMR spectrum of compound **60.**

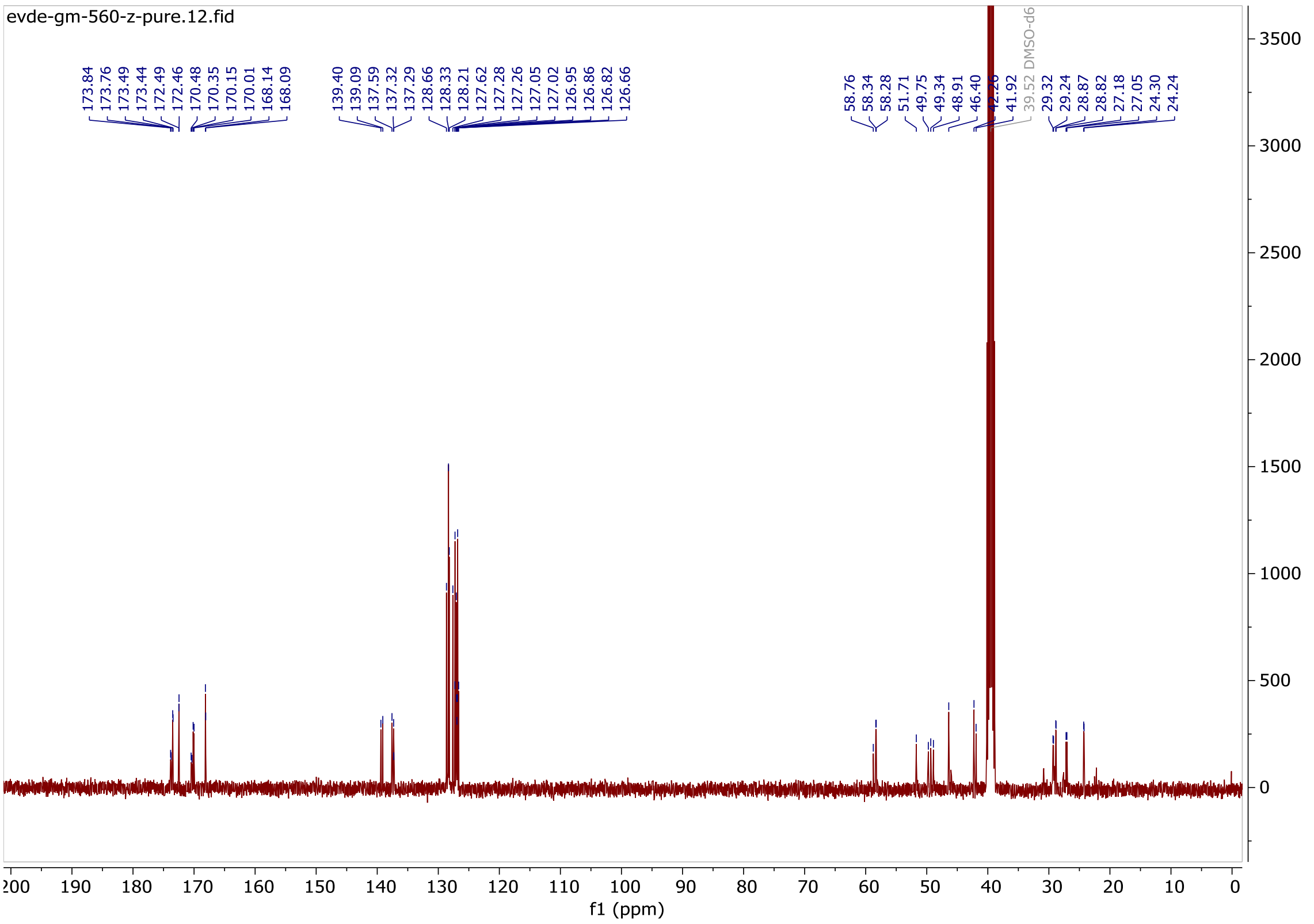

Figure S124. ^{13}C-NMR spectrum of compound **60.**

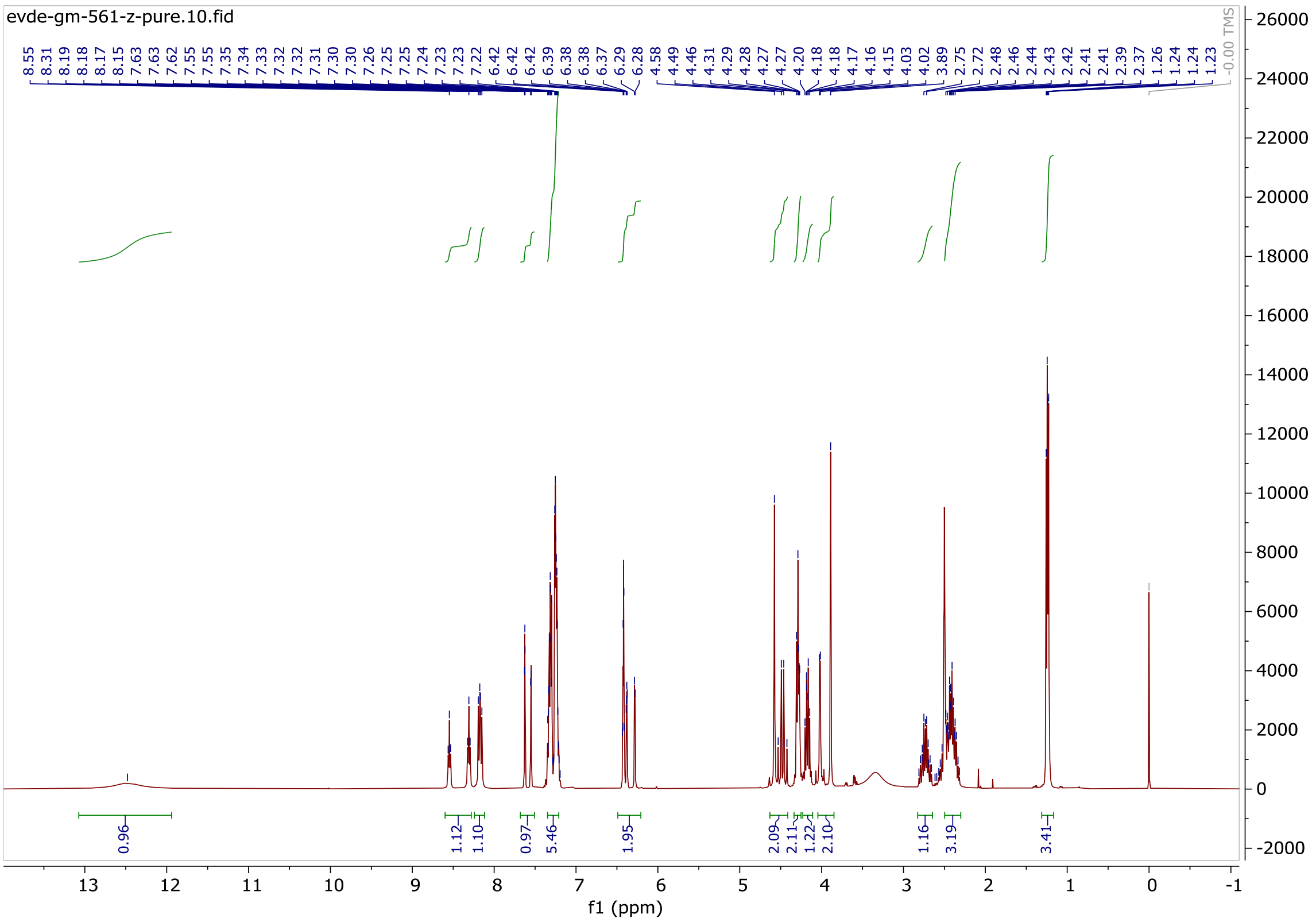

Figure S125. ^{1}H-NMR spectrum of compound **61.**

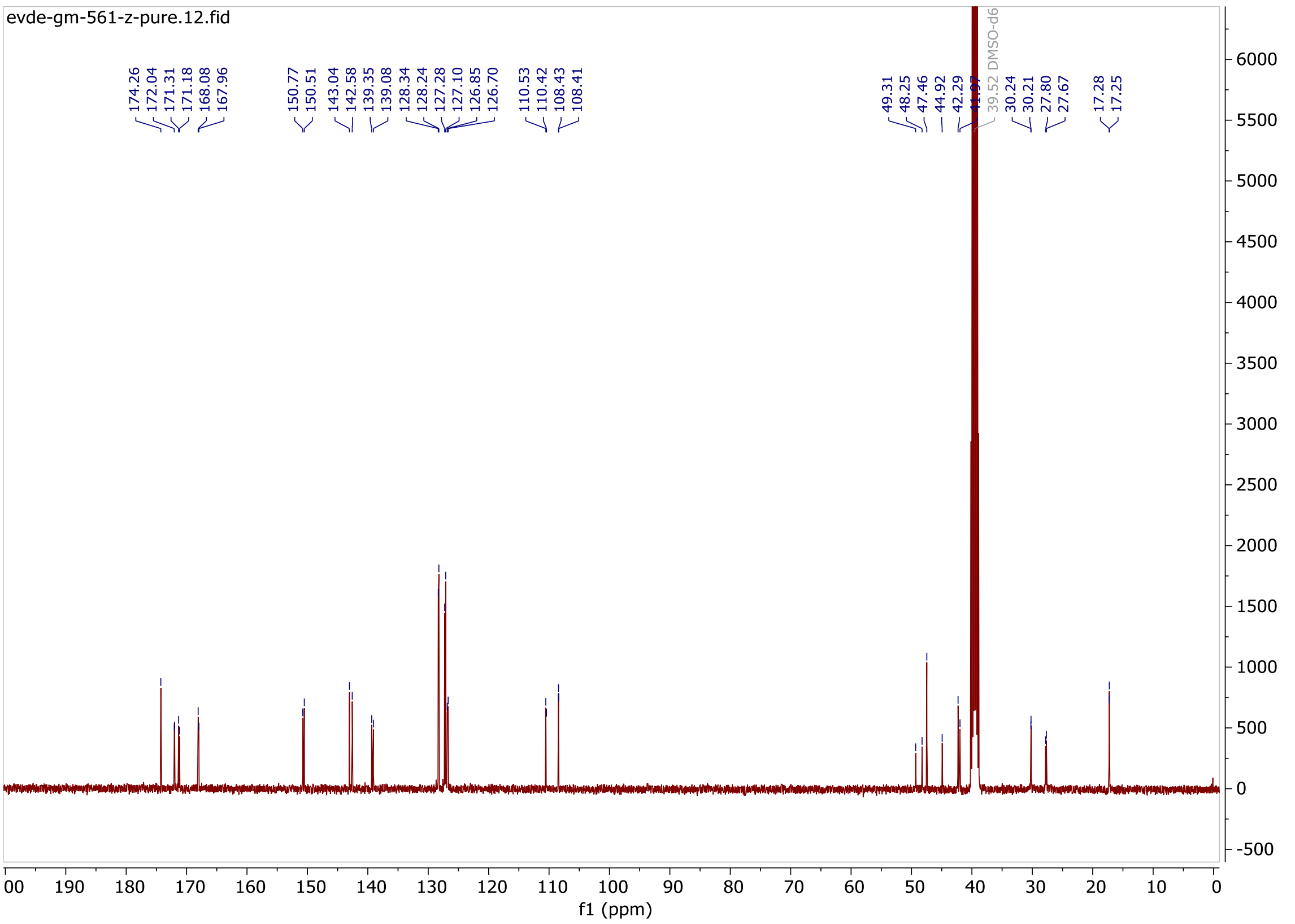

Figure S126. ^{13}C-NMR spectrum of compound **61.**

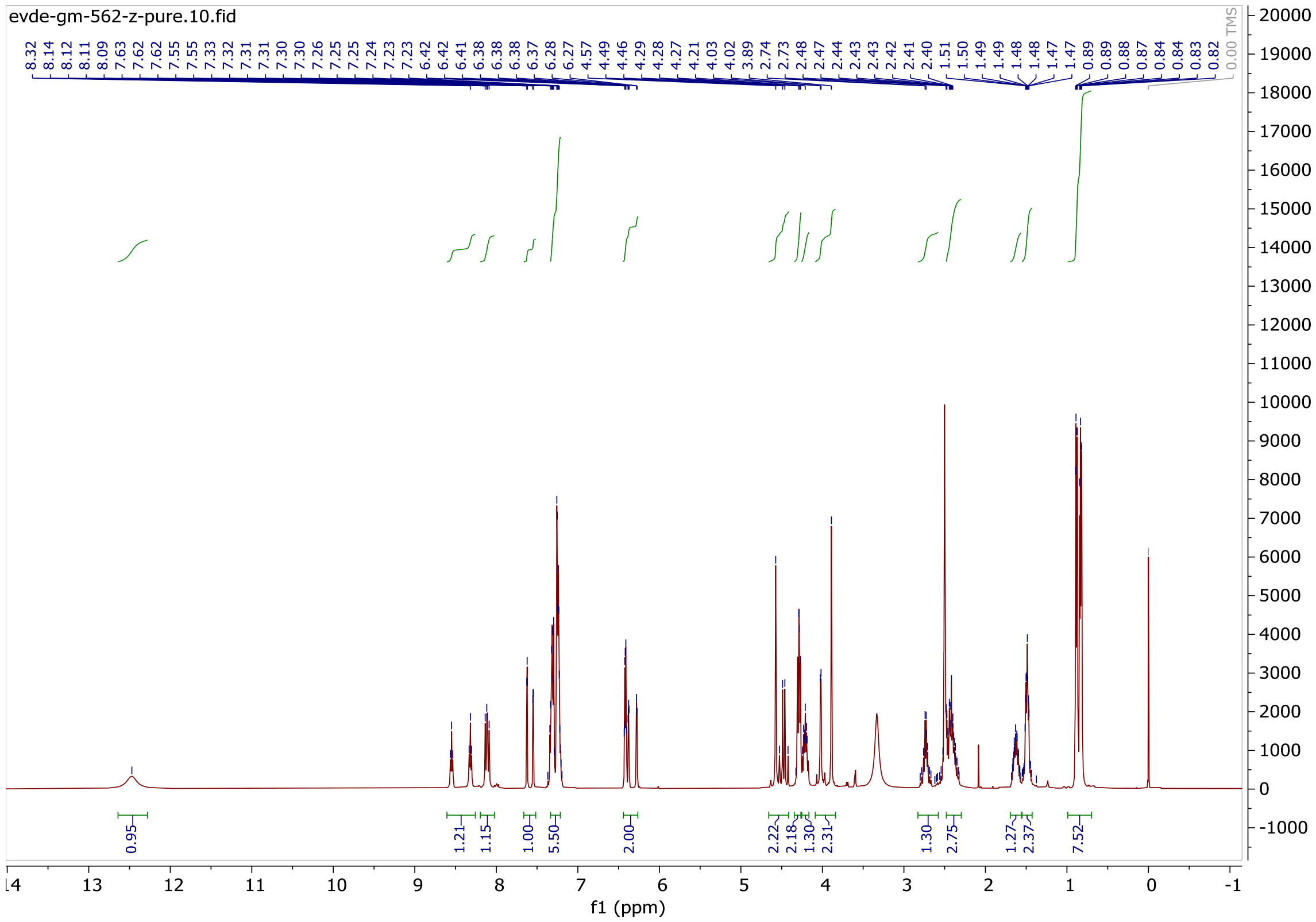

Figure S127. ^{1}H-NMR spectrum of compound **62.**

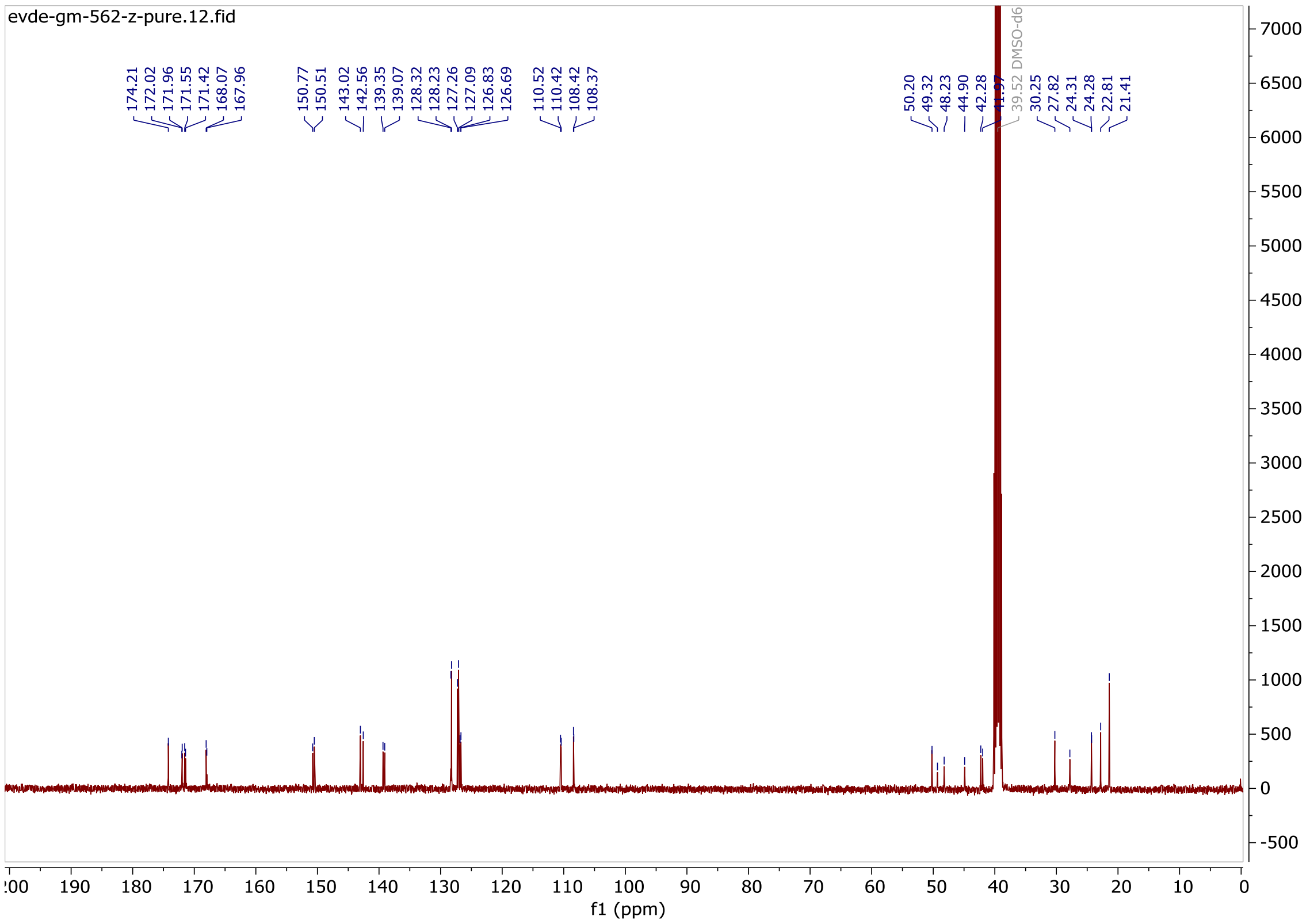

Figure S128. ^{13}C-NMR spectrum of compound **62.**

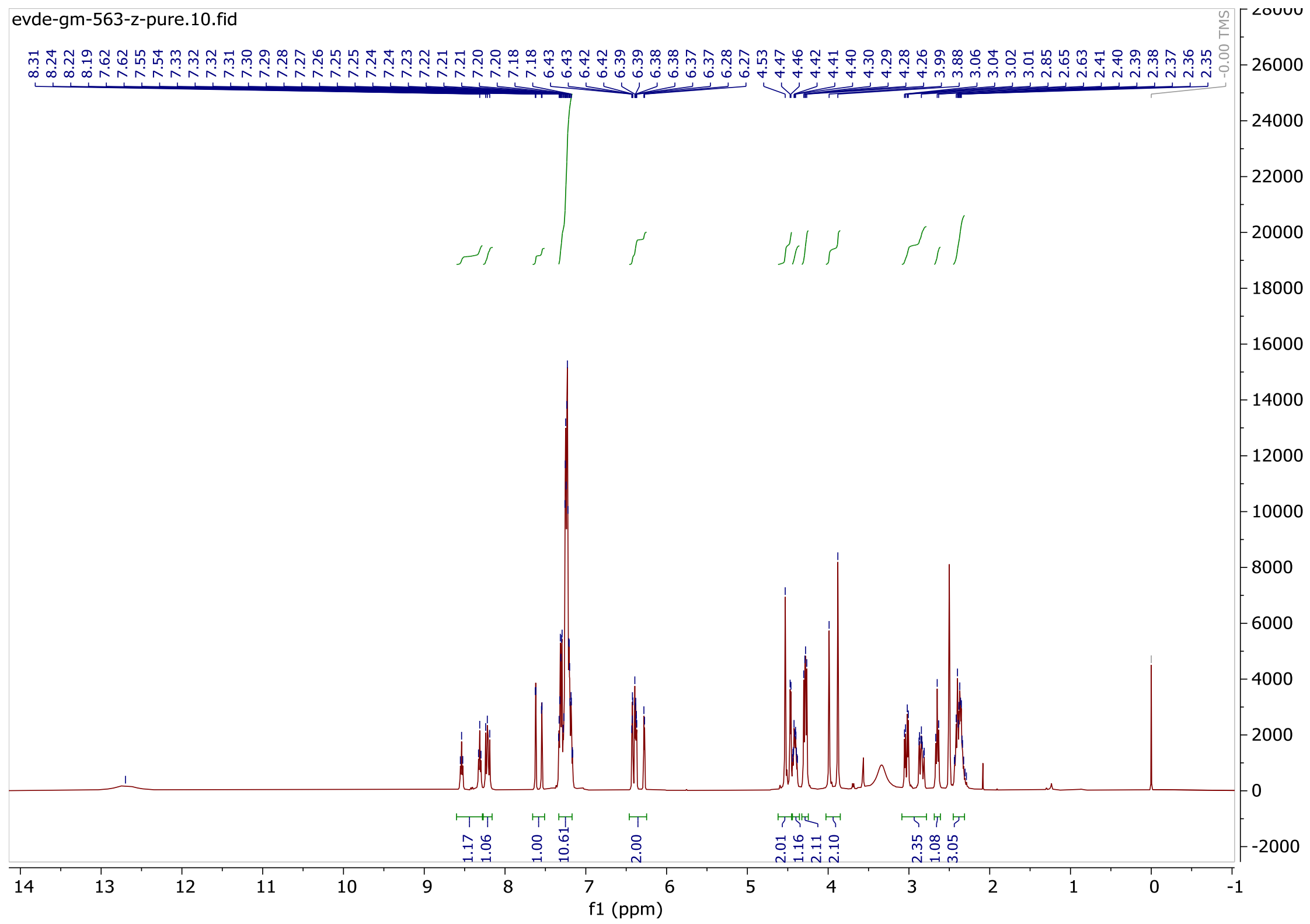

Figure S129. ^{1}H-NMR spectrum of compound **63.**

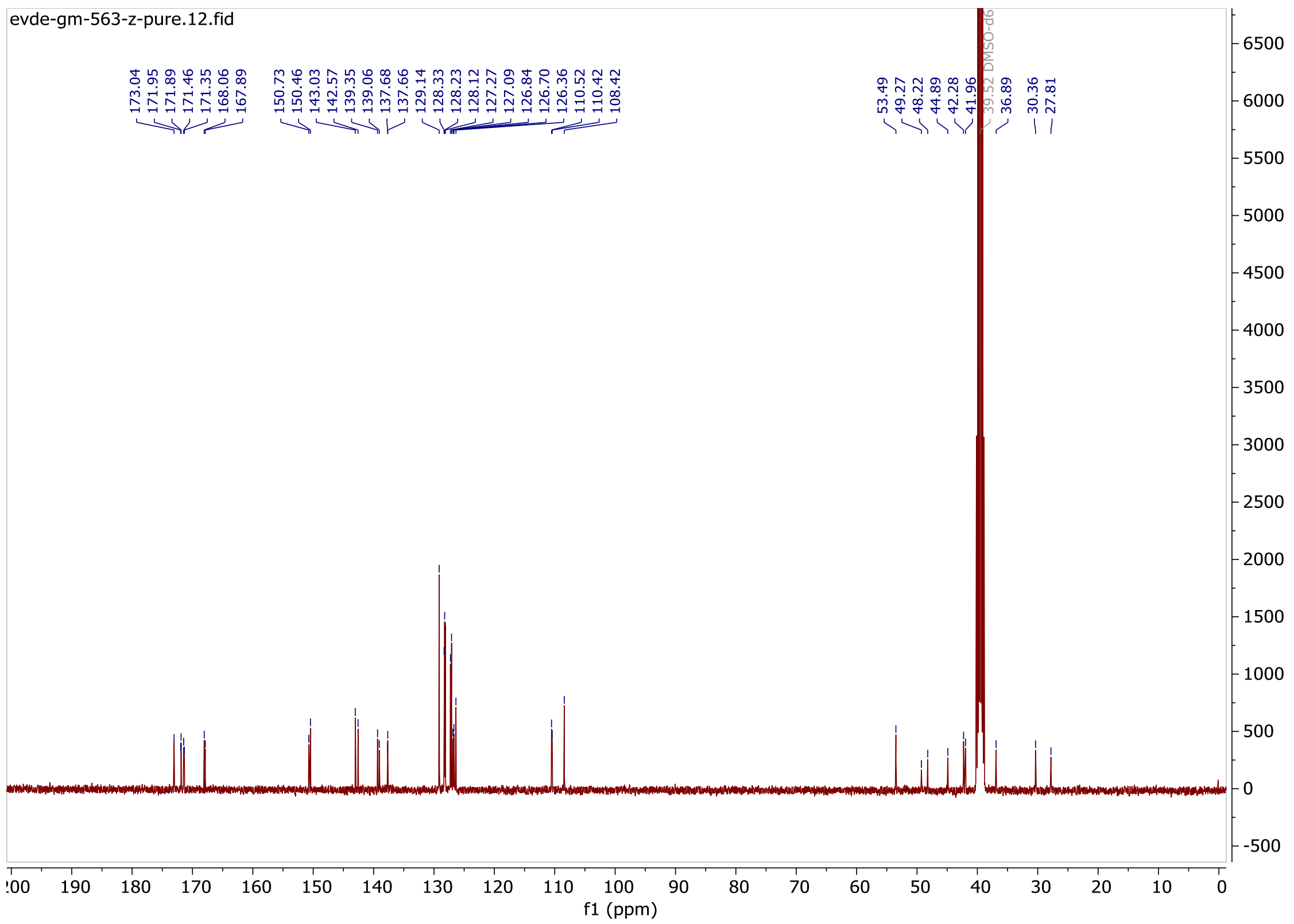

Figure S130. ^{13}C-NMR spectrum of compound **63.**

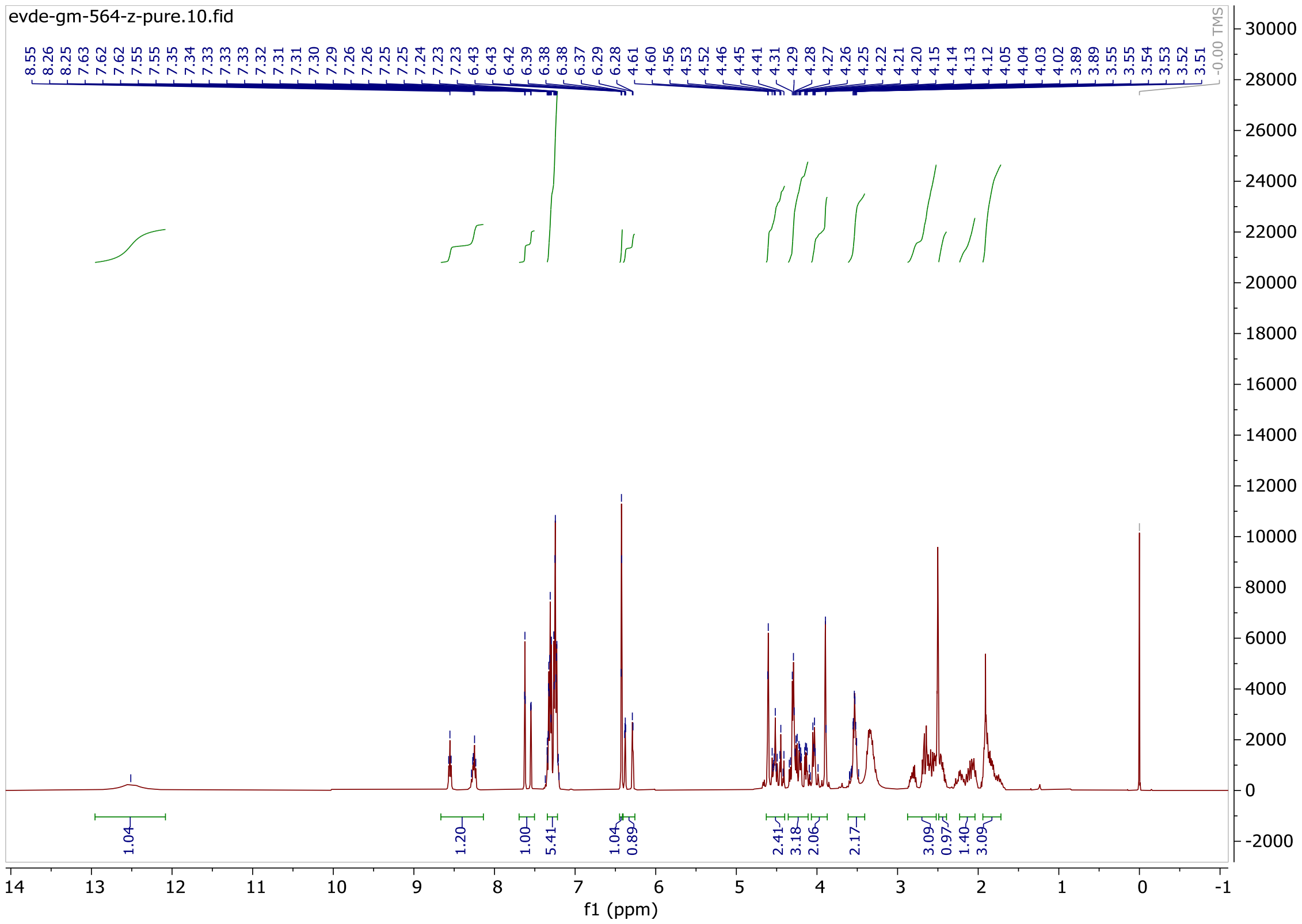

Figure S131. ^{1}H-NMR spectrum of compound **64.**

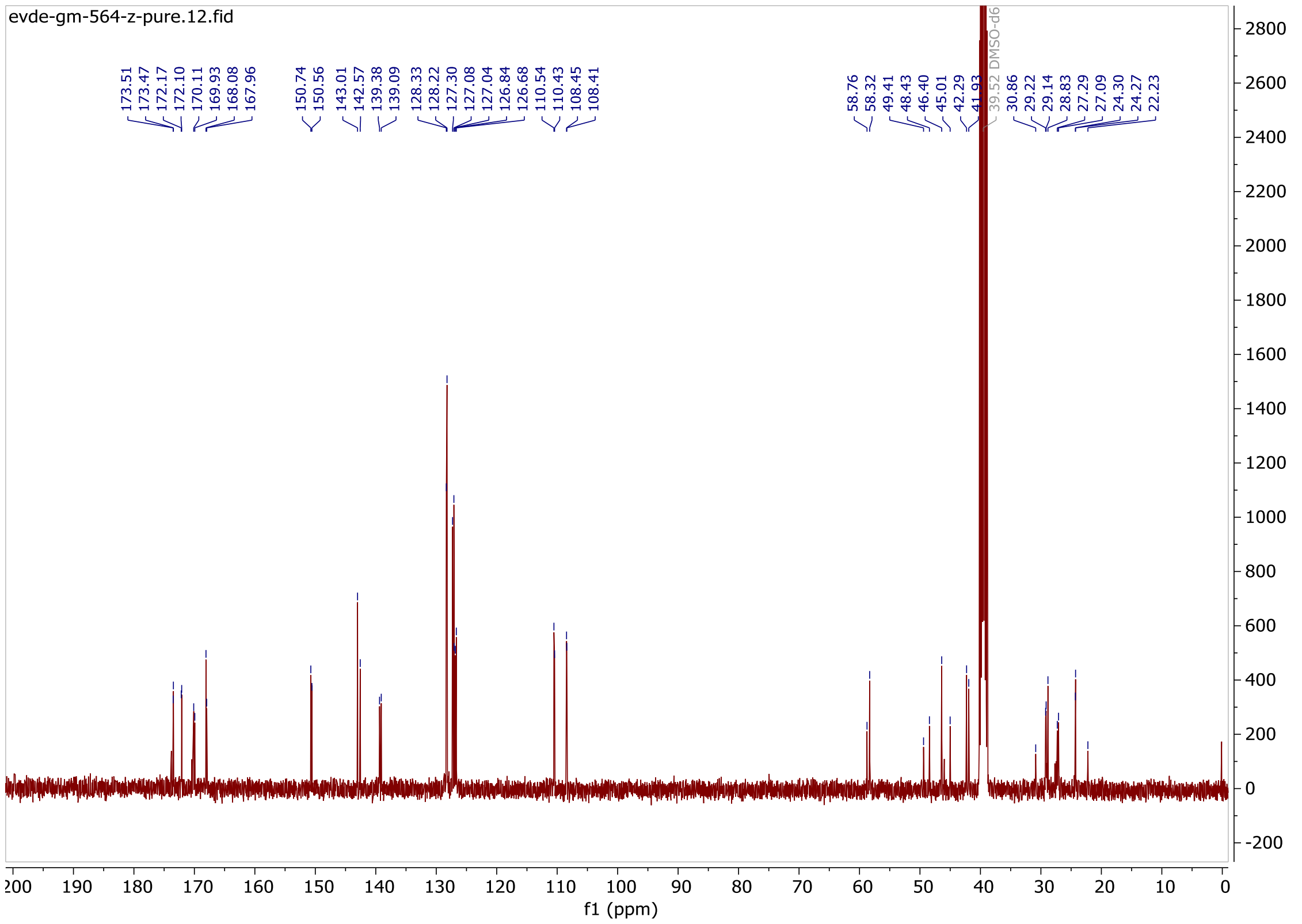

Figure S132. ^{13}C-NMR spectrum of compound **64.**

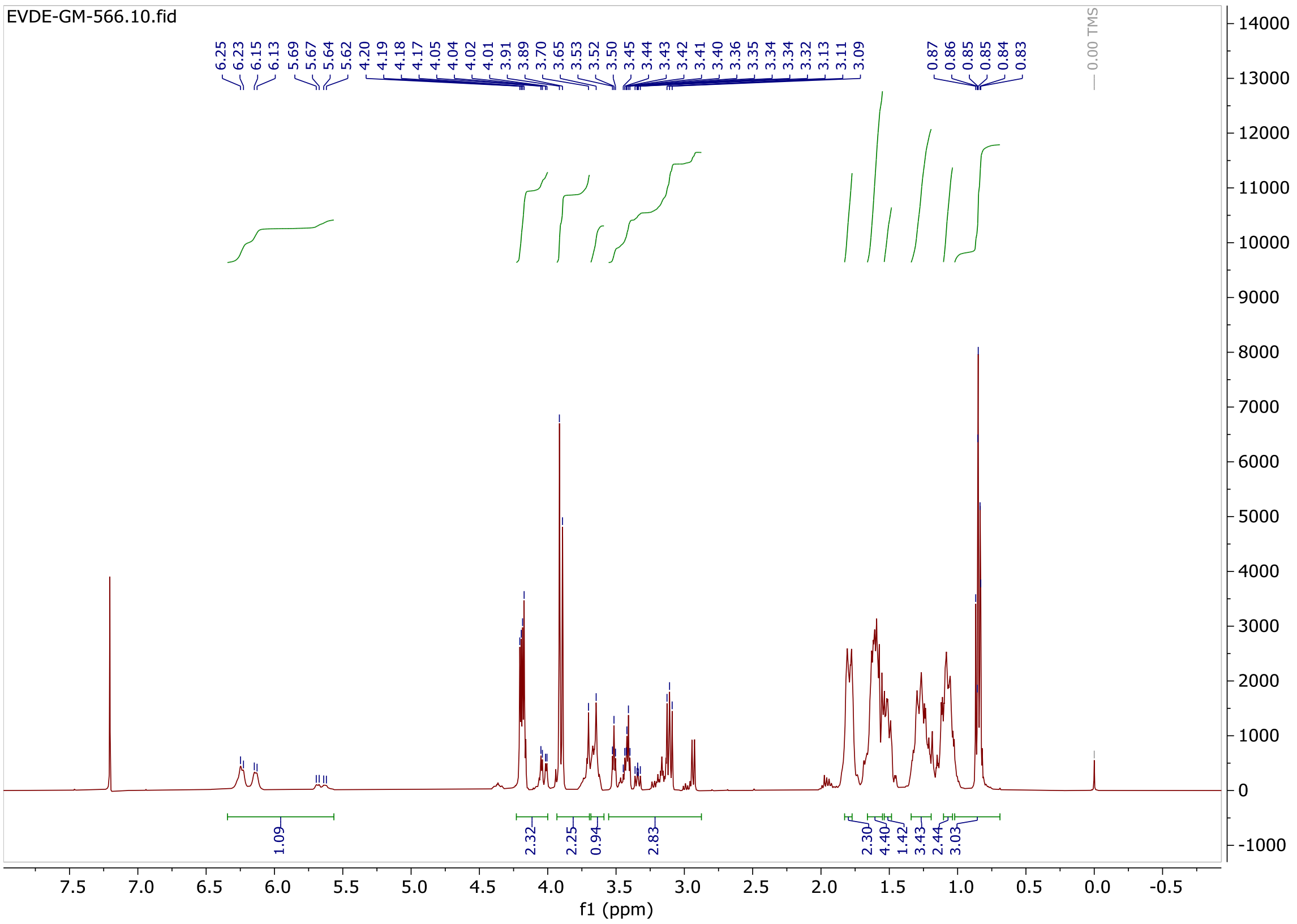

Figure S133. ^{1}H-NMR spectrum of compound **65.**

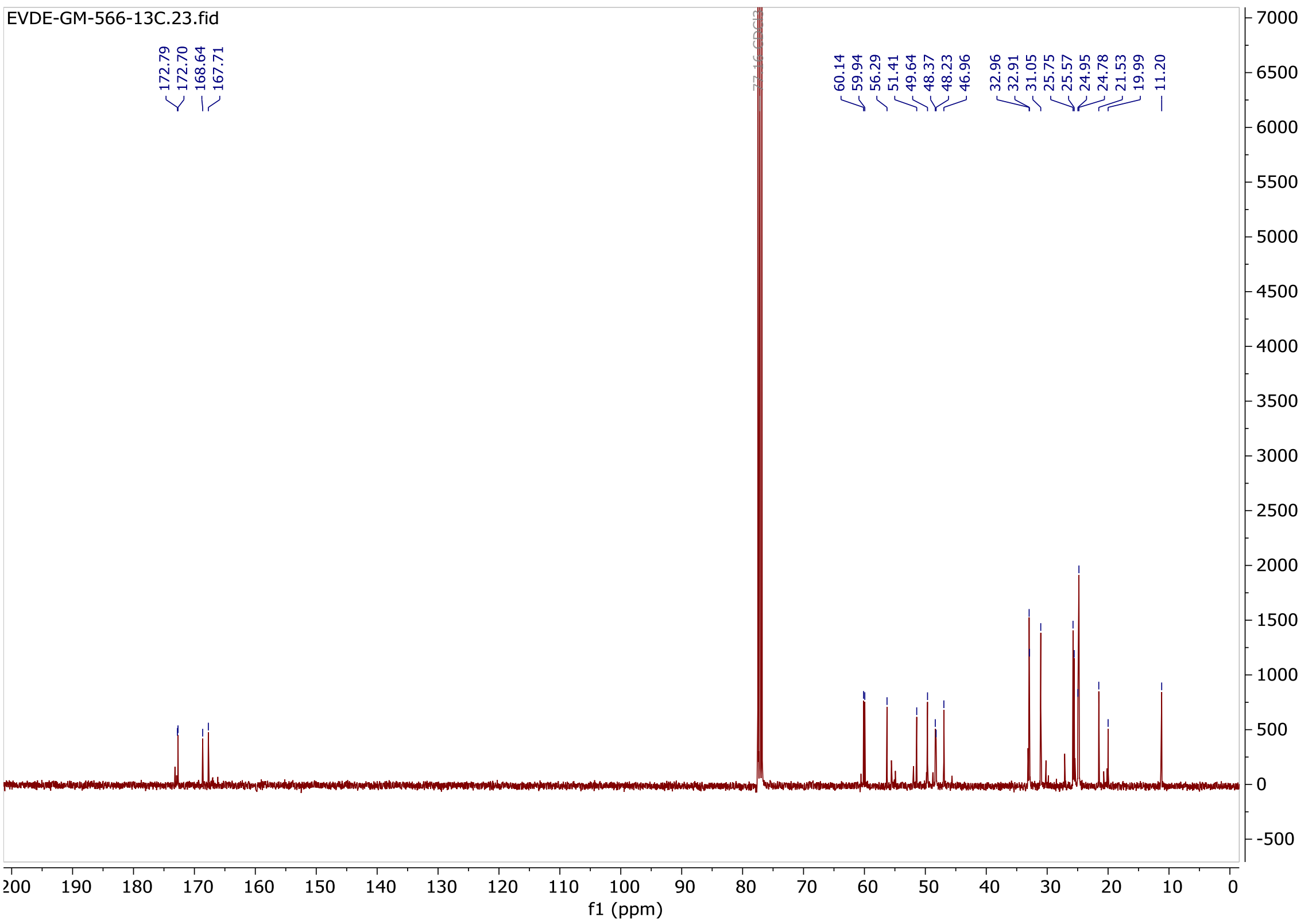

Figure S134. ^{13}C-NMR spectrum of compound **65.**

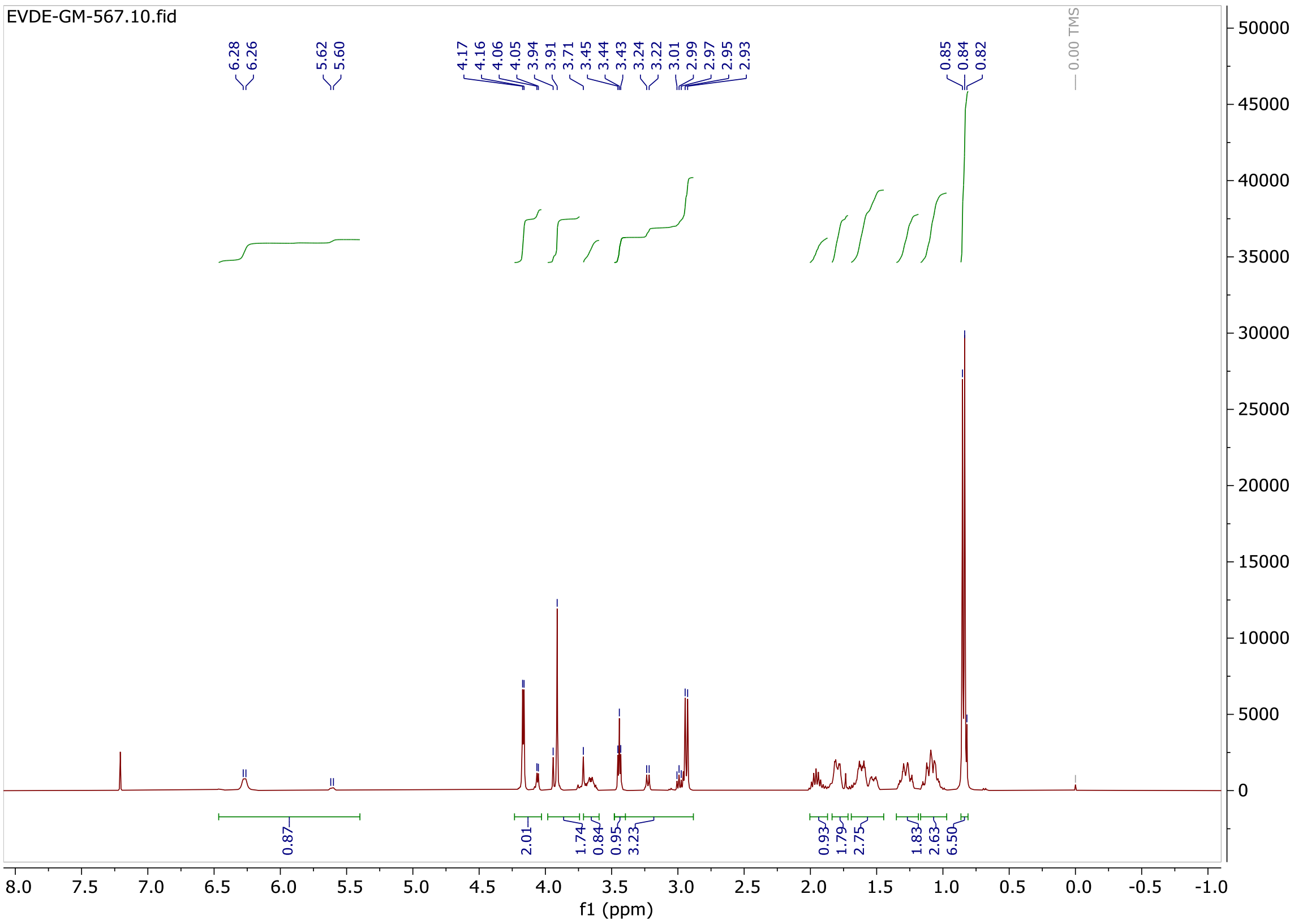

Figure S135. ^{1}H-NMR spectrum of compound **66.**

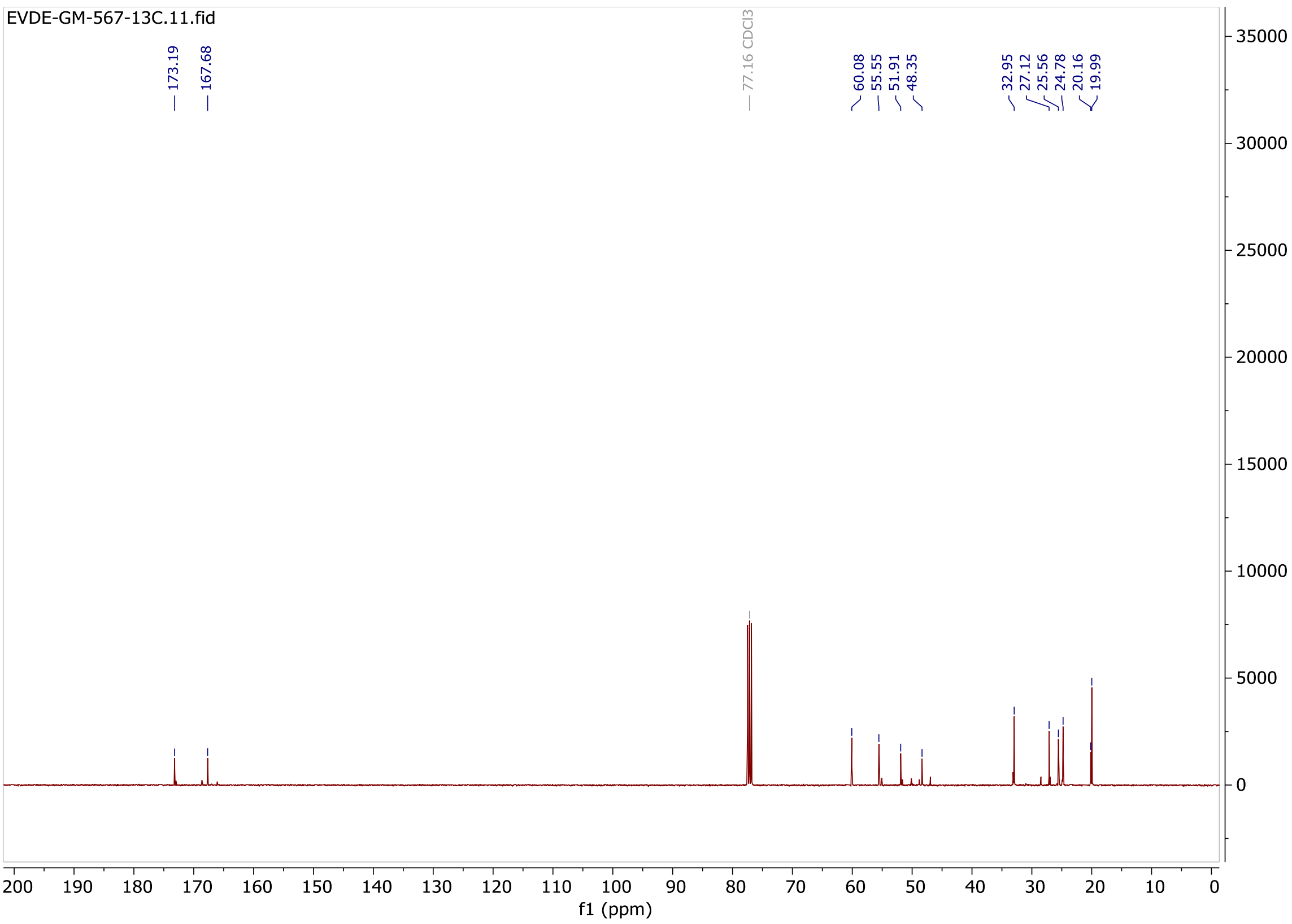

Figure S134. ^{13}C-NMR spectrum of compound **66.**

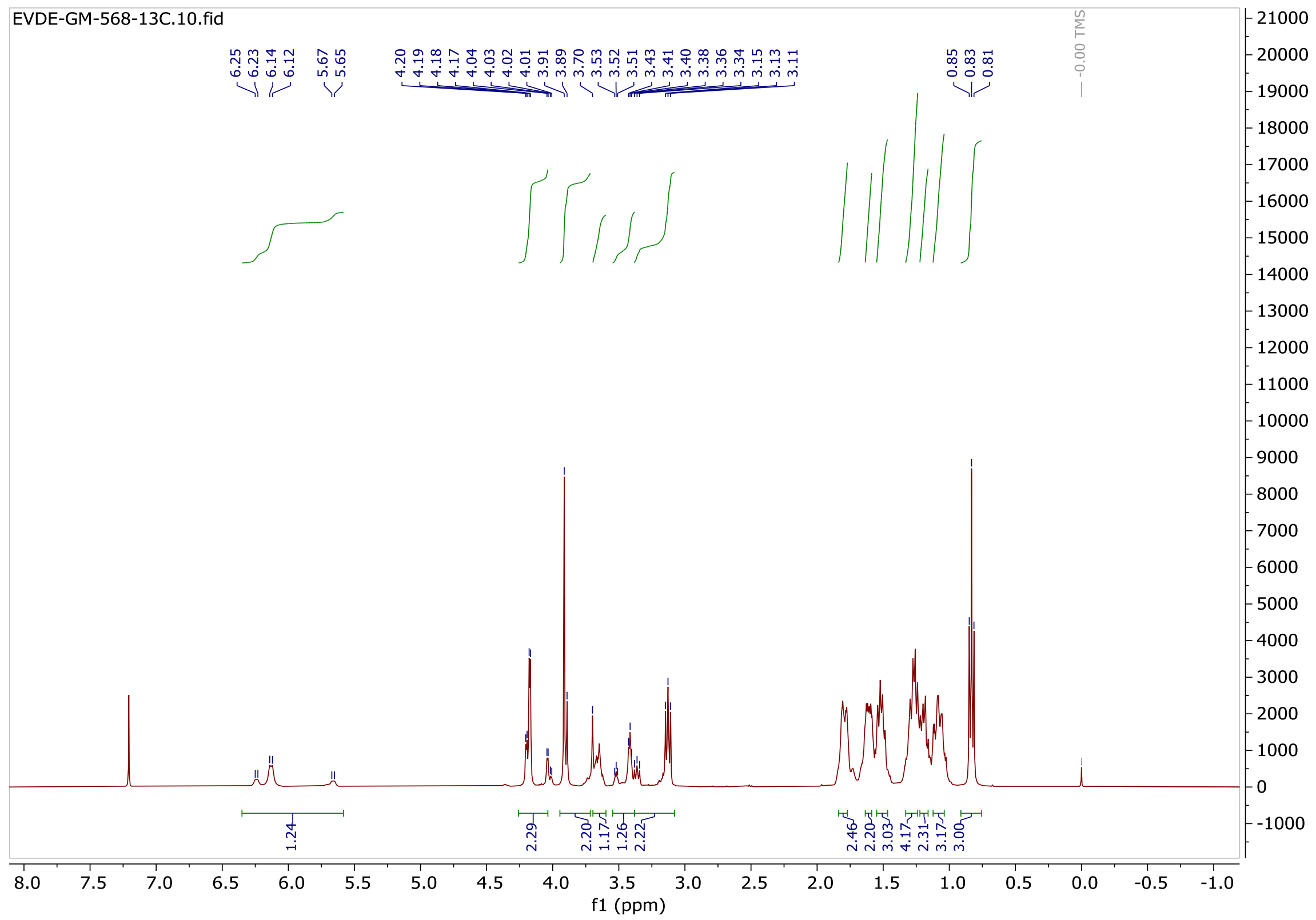

Figure S135. ^{1}H-NMR spectrum of compound **67.**

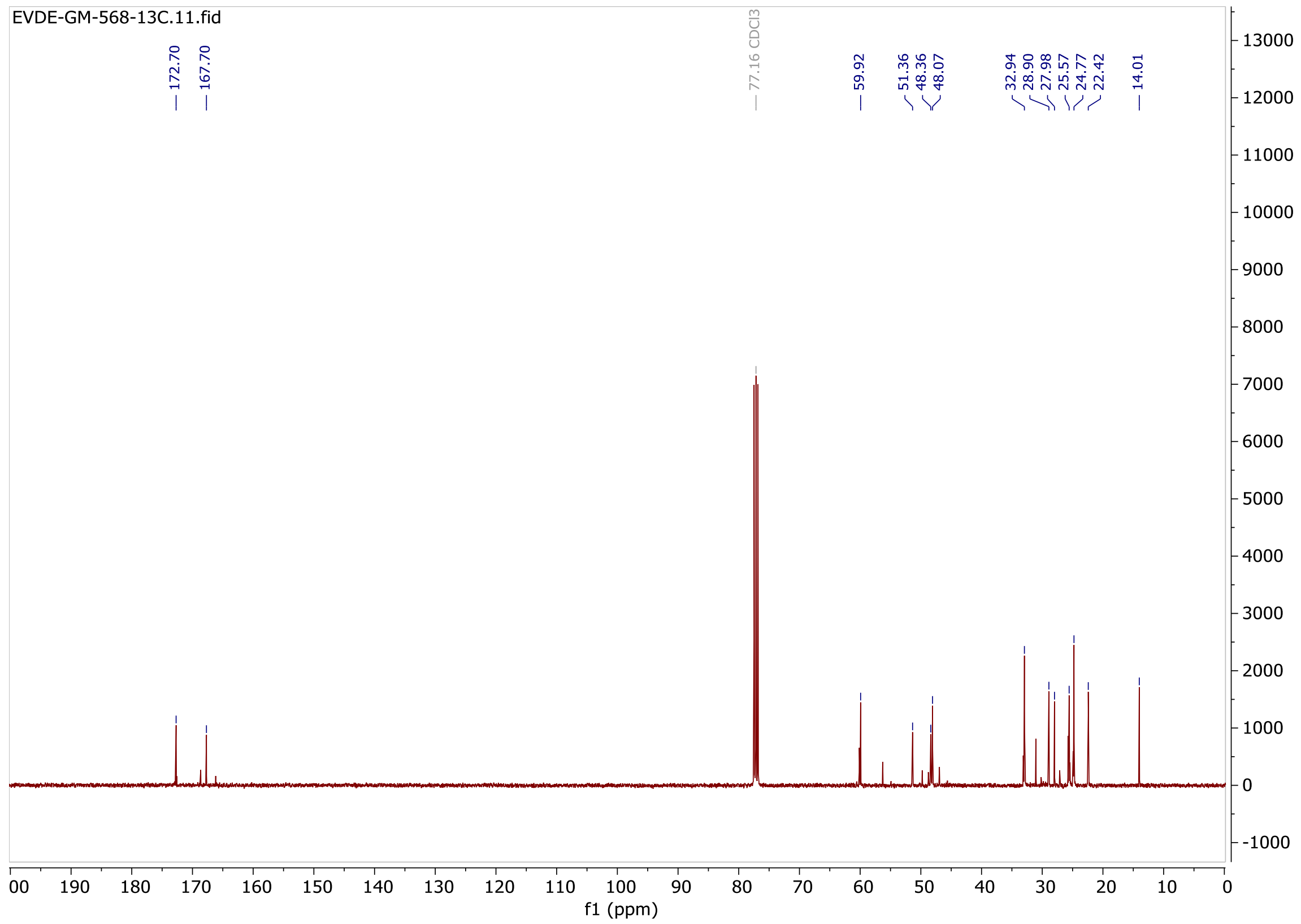

Figure S136. ^{13}C-NMR spectrum of compound **67.**

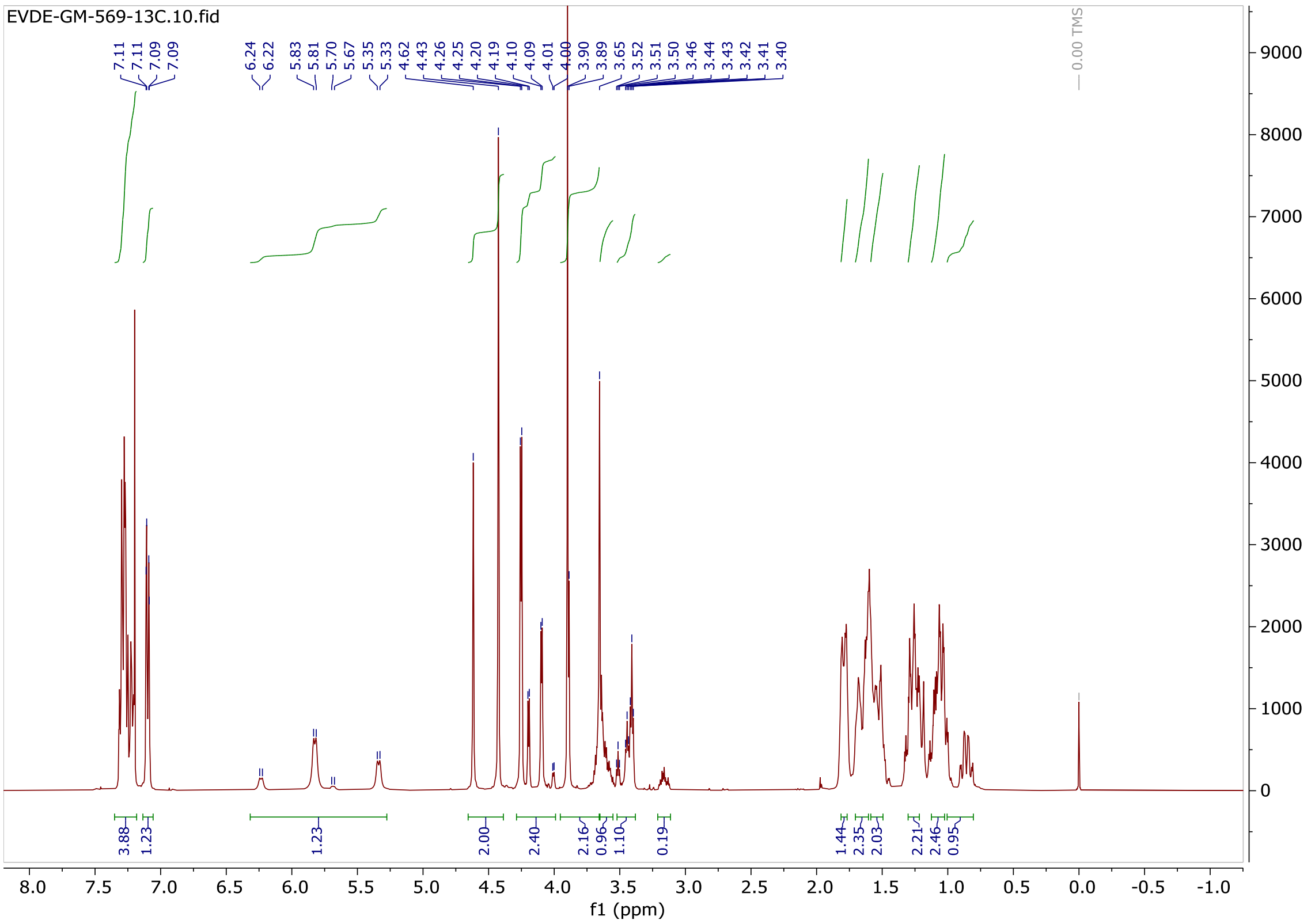

Figure S137. ^{1}H-NMR spectrum of compound **68.**

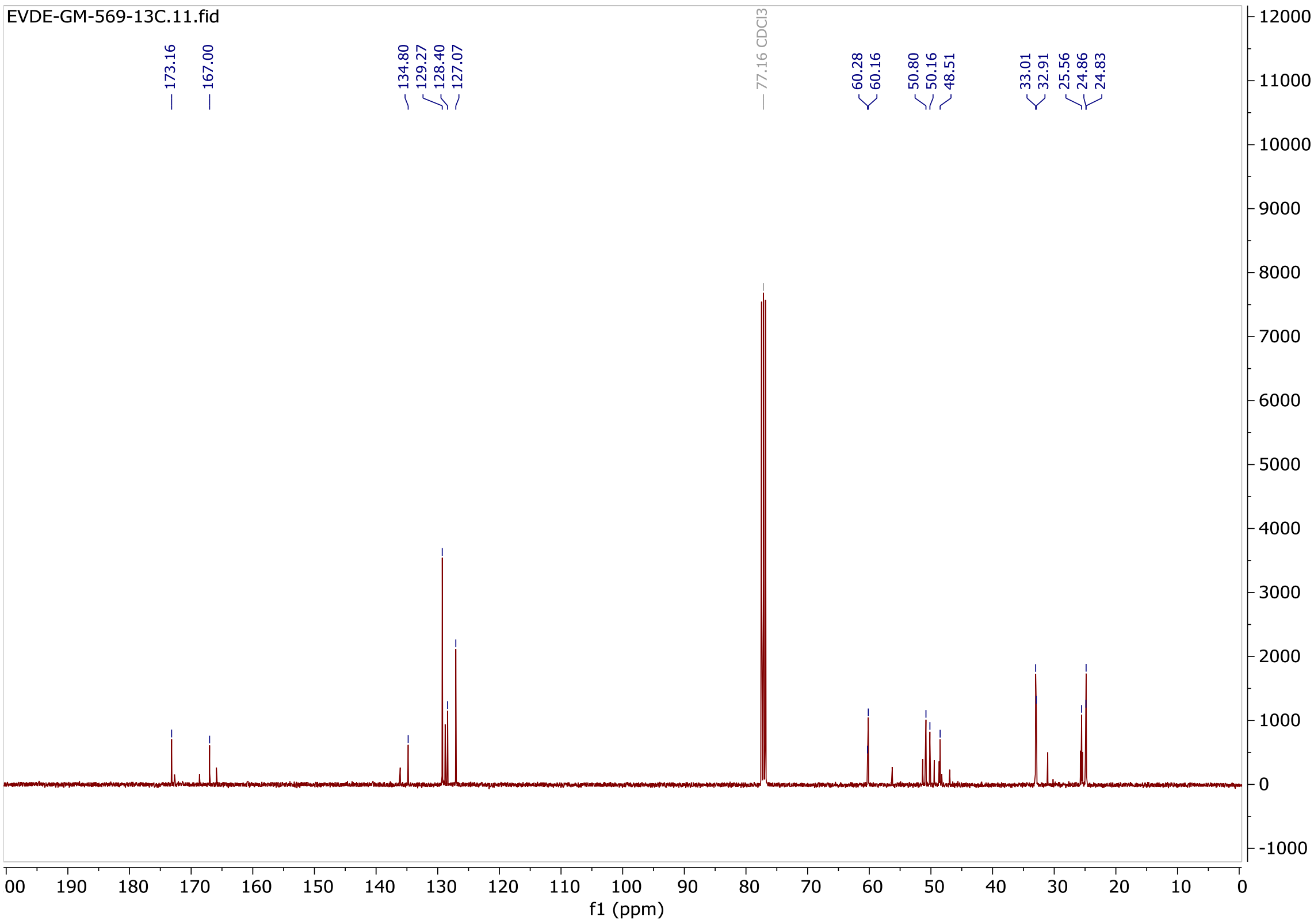

Figure S138. ^{13}C-NMR spectrum of compound **68.**

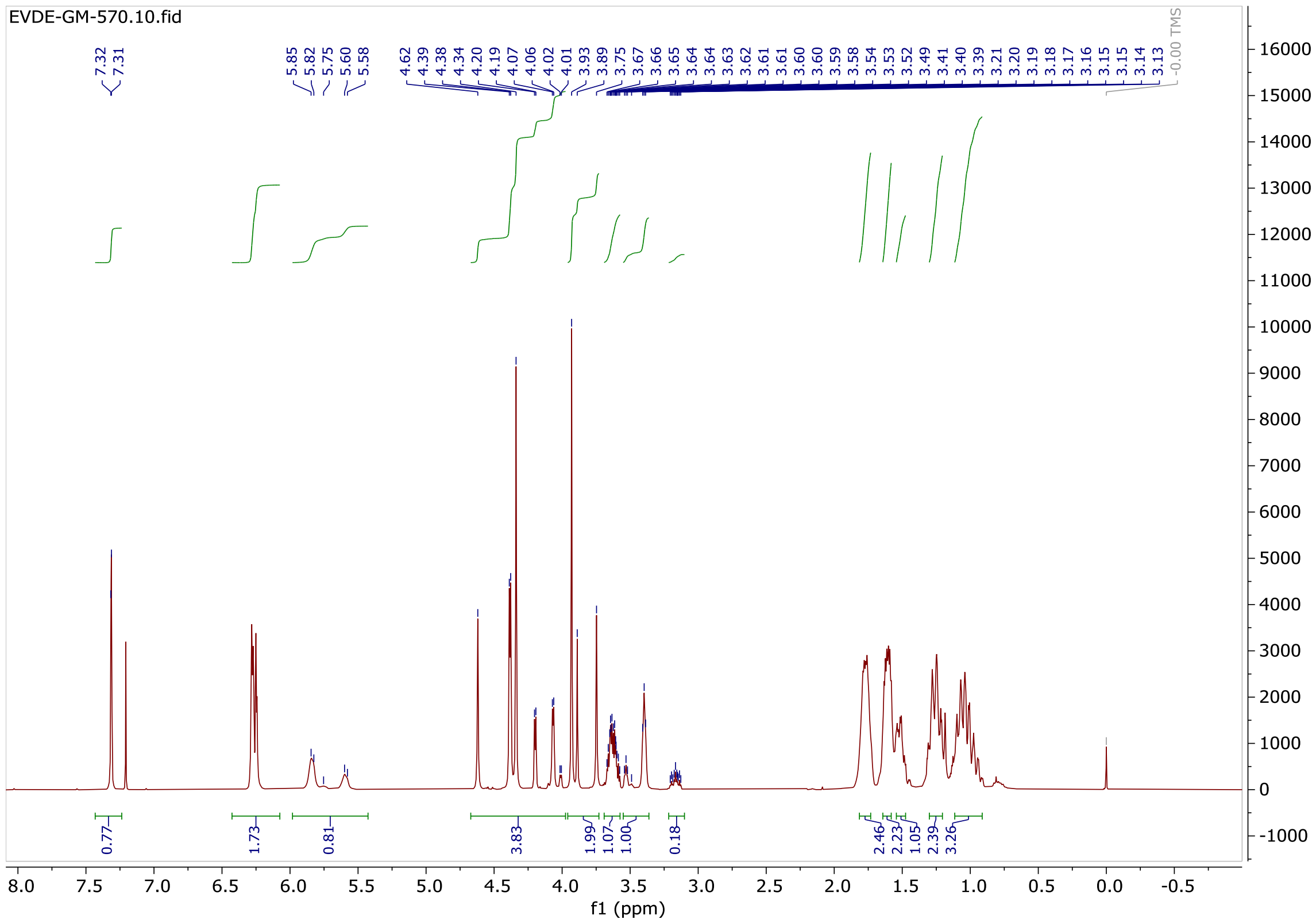

Figure S139. ^{1}H-NMR spectrum of compound **69.**

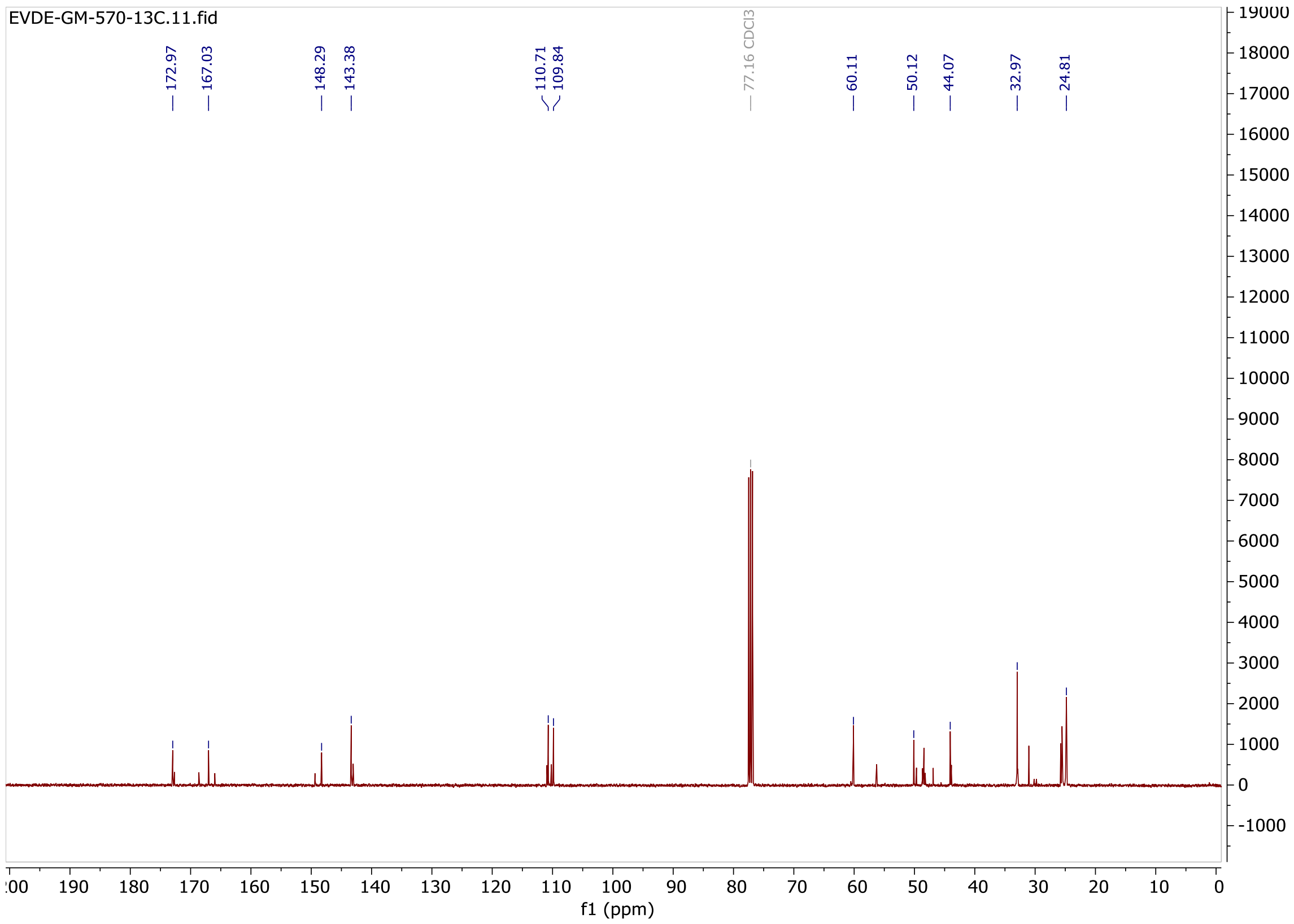

Figure S140. ^{13}C-NMR spectrum of compound **69.**

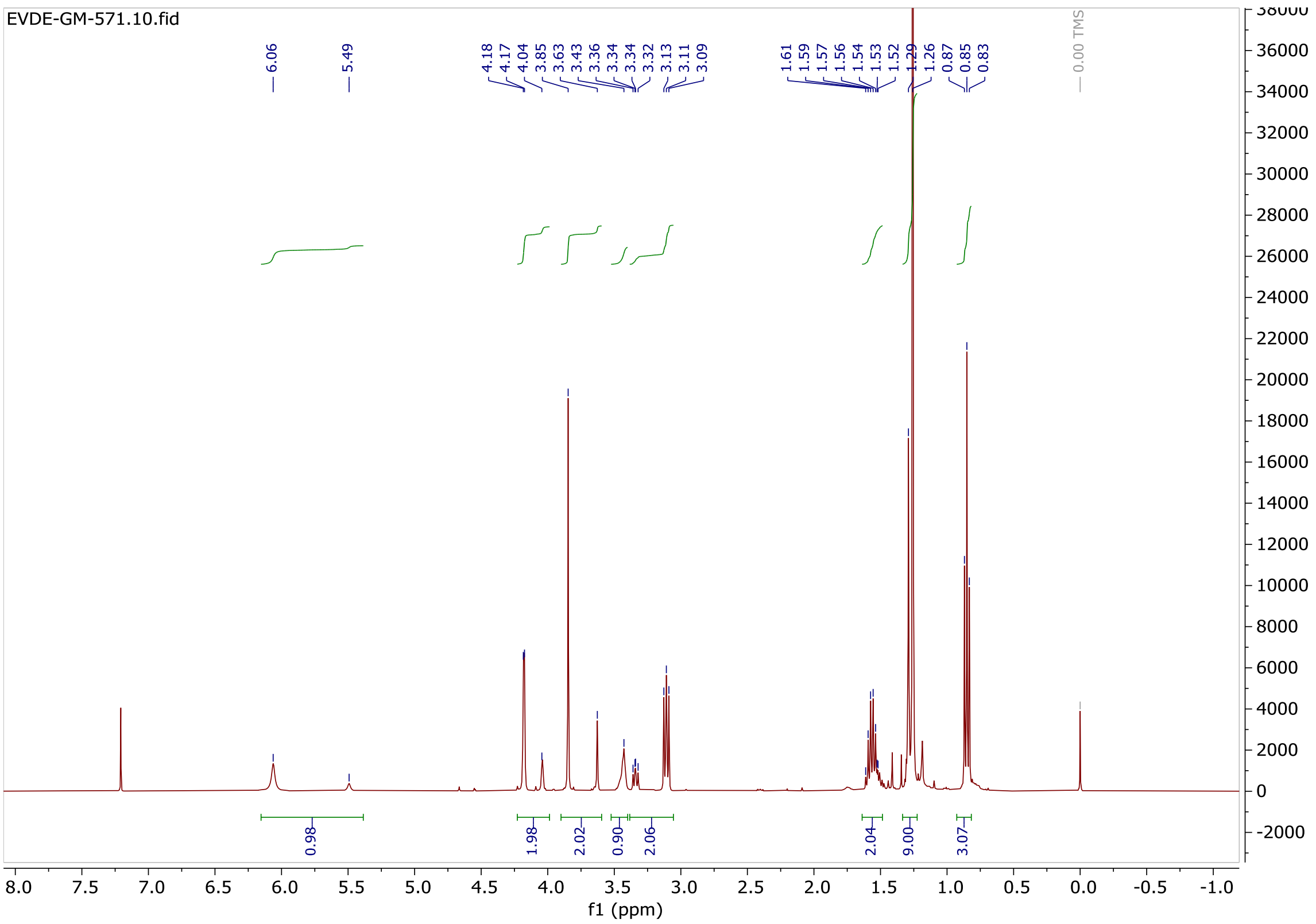

Figure S141. ^{1}H-NMR spectrum of compound **70.**

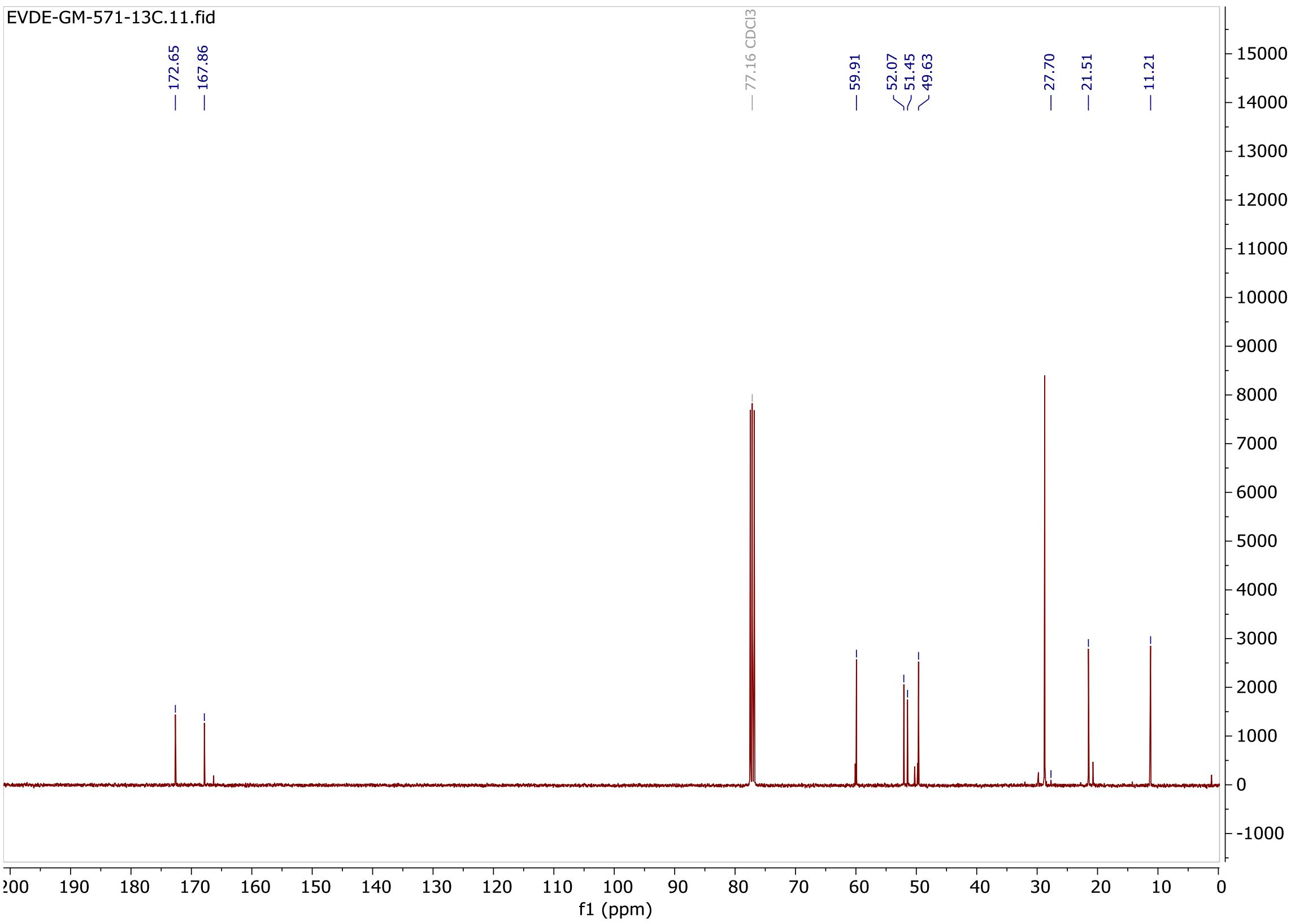

Figure S141. ^{13}C-NMR spectrum of compound **70.**

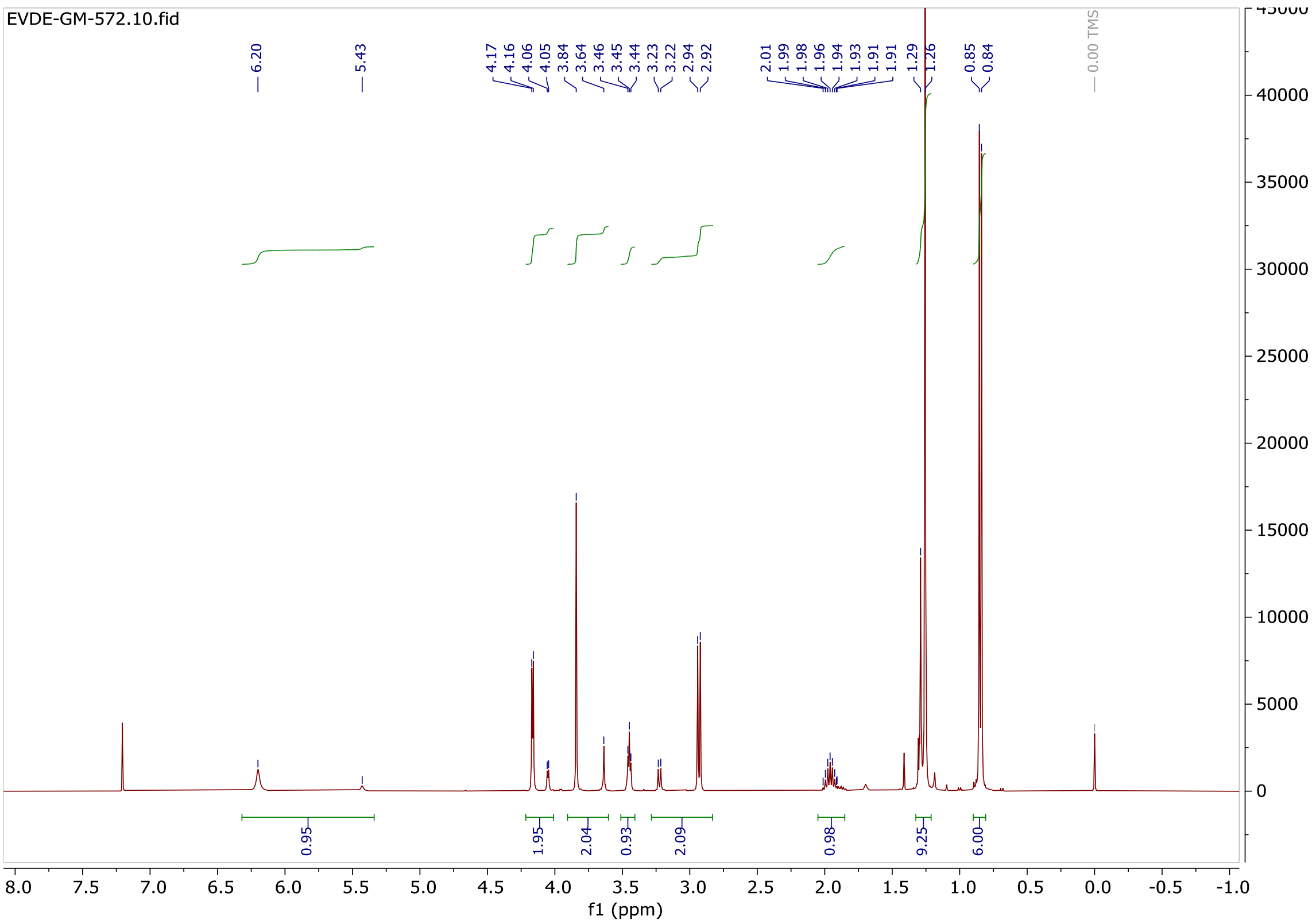

Figure S143. ^{1}H-NMR spectrum of compound **71.**

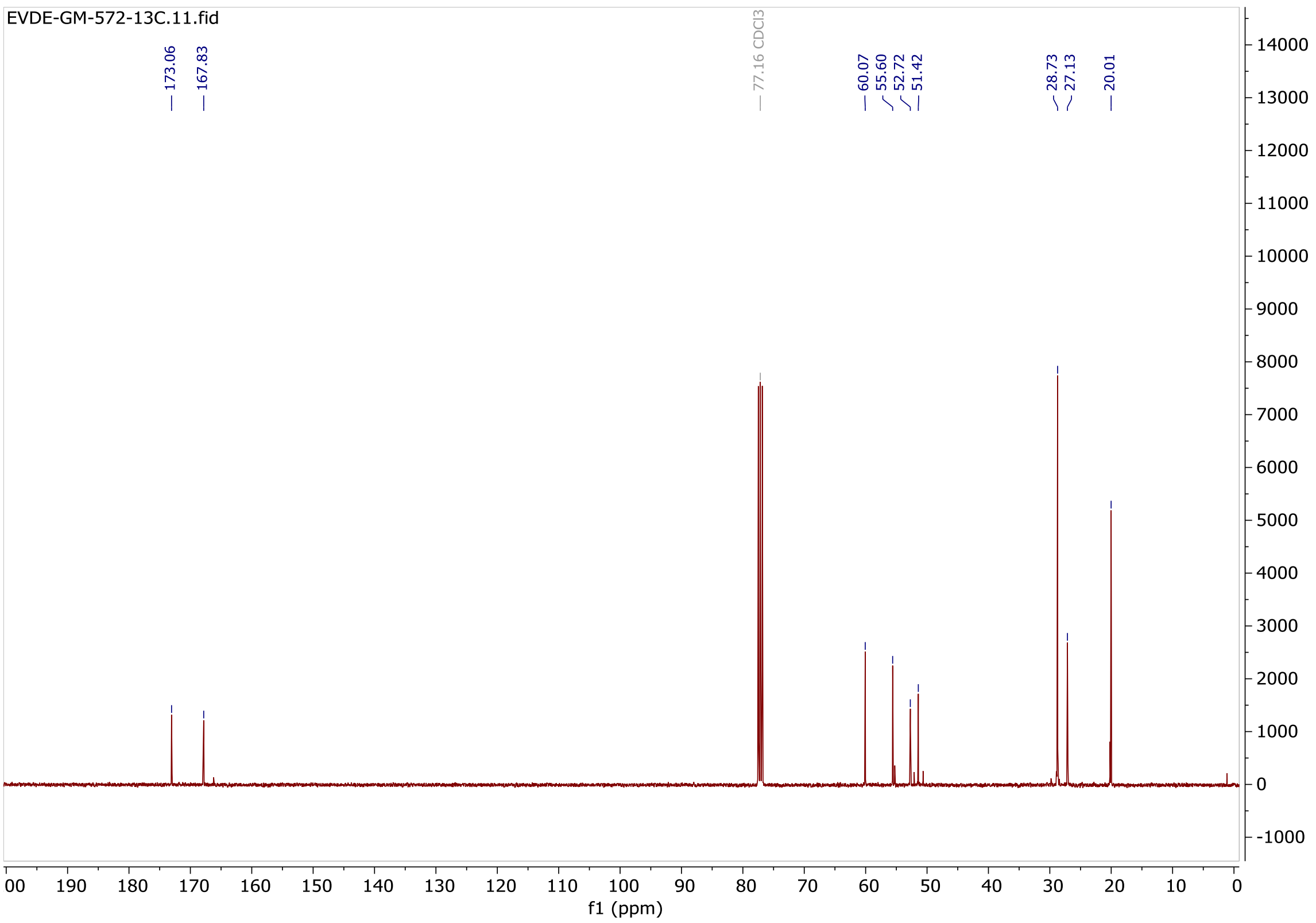

Figure S144. ^{13}C-NMR spectrum of compound **71.**

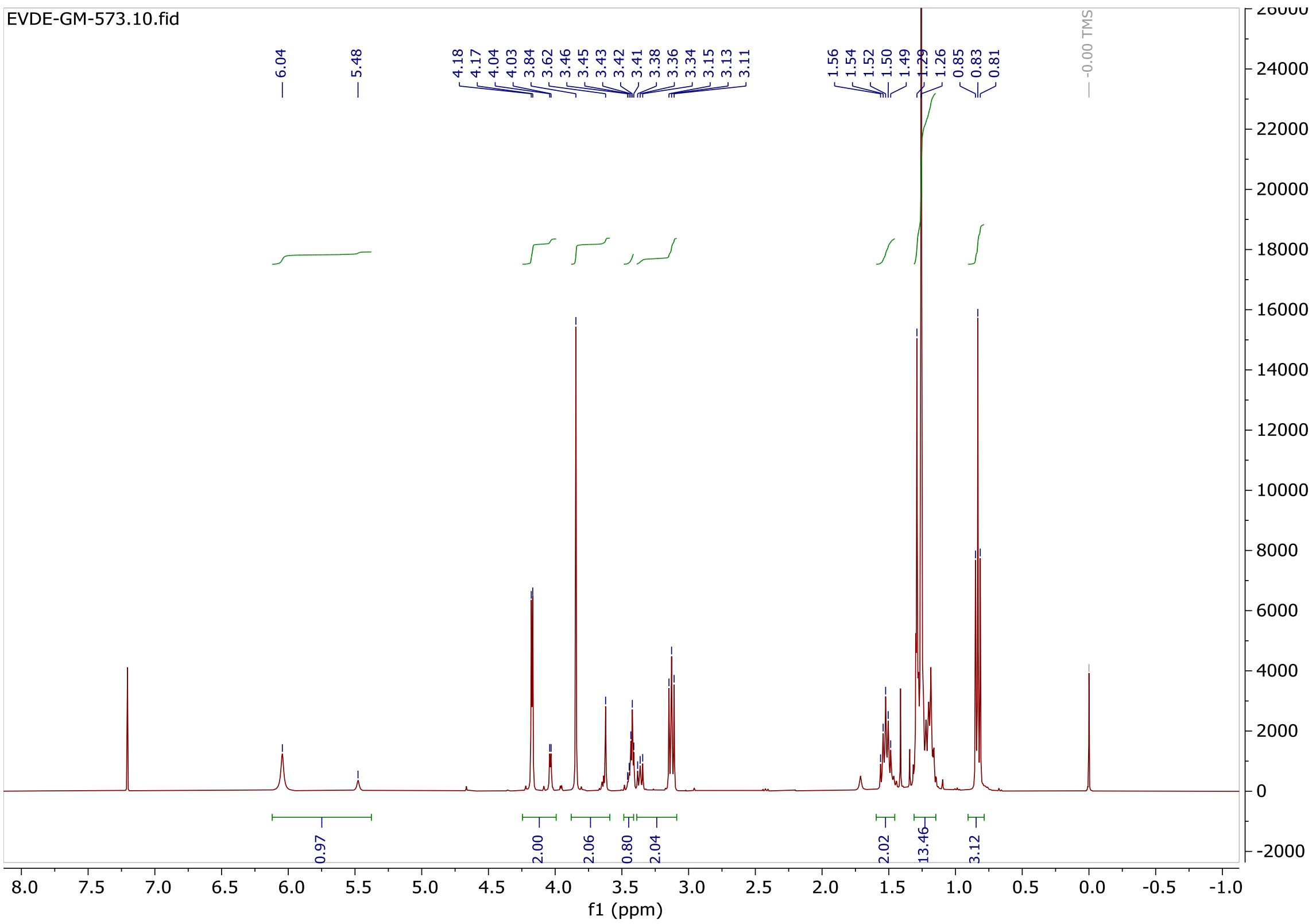

Figure S145. ^{1}H-NMR spectrum of compound **72.**

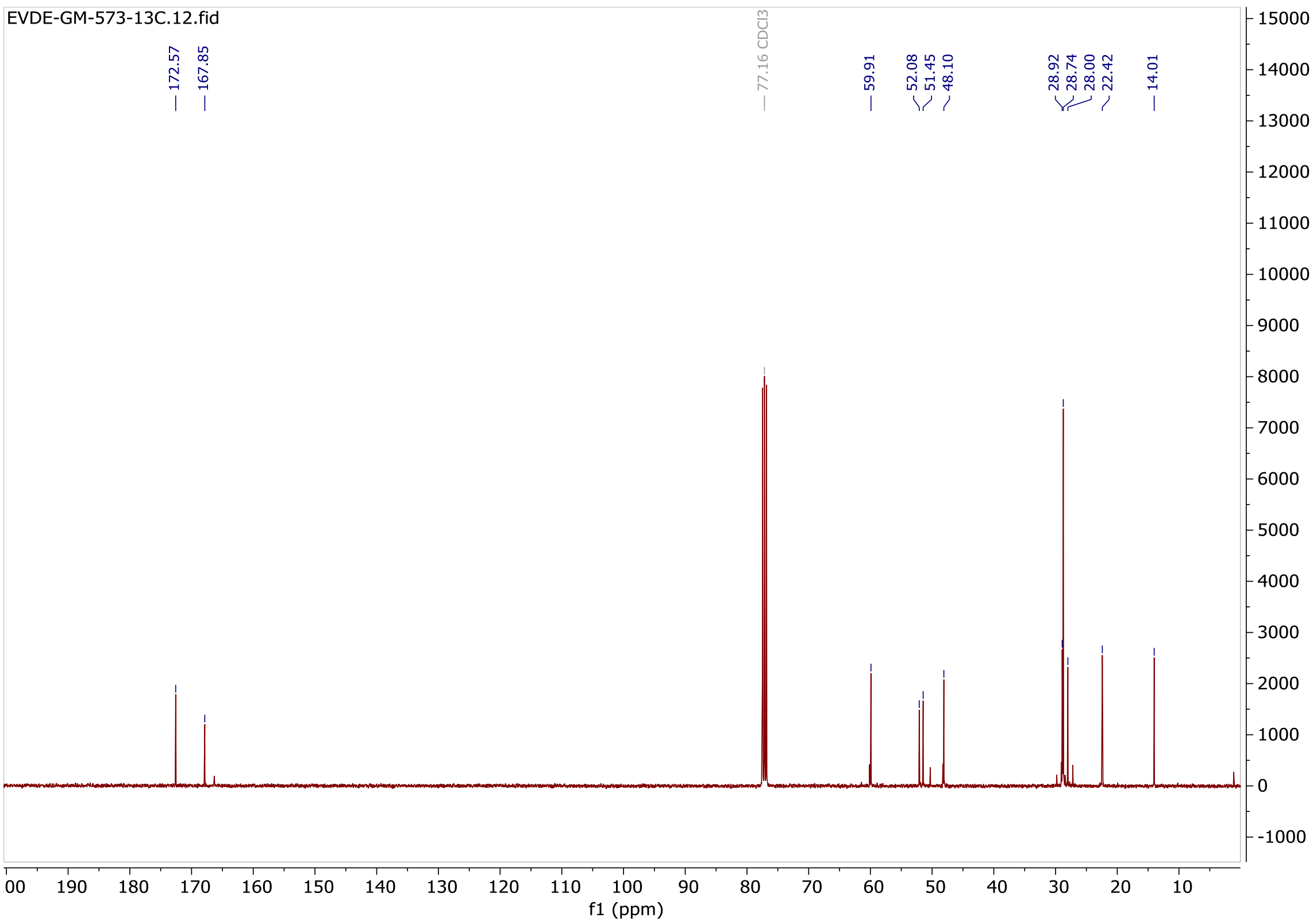

Figure S146. ^{13}C-NMR spectrum of compound **72.**

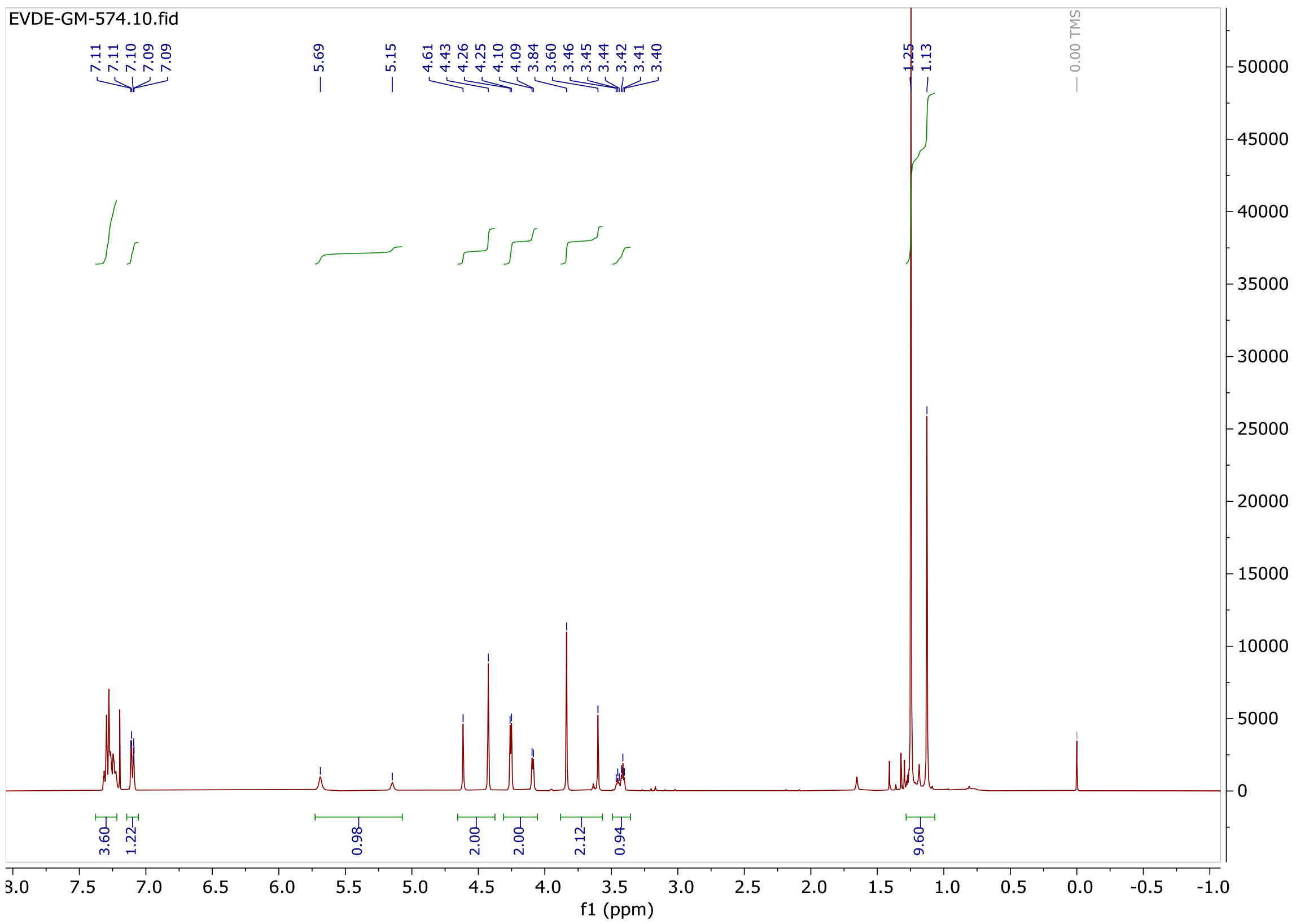

Figure S147. ^{1}H-NMR spectrum of compound **73.**

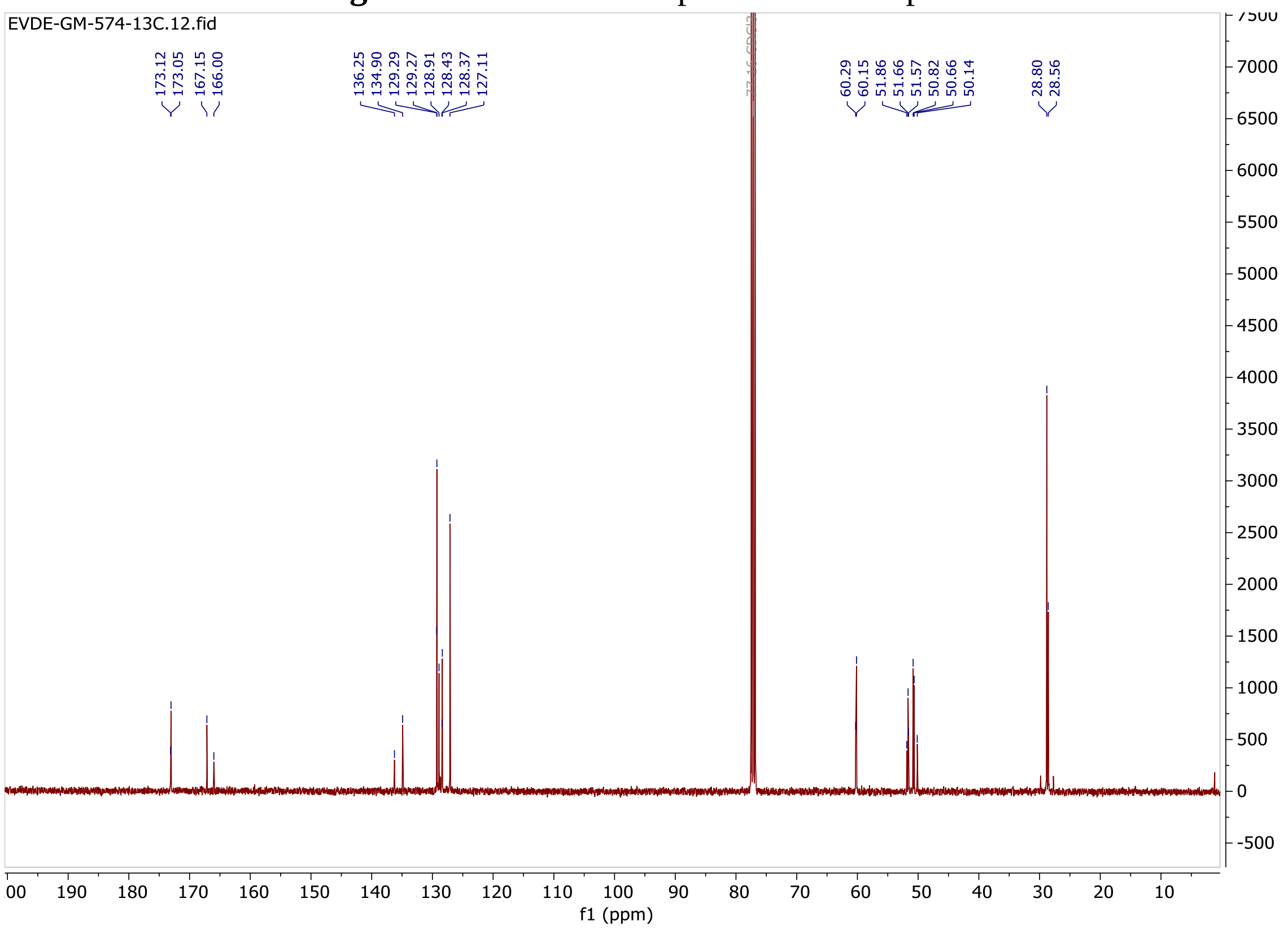

Figure S148. ^{13}C-NMR spectrum of compound **73.**

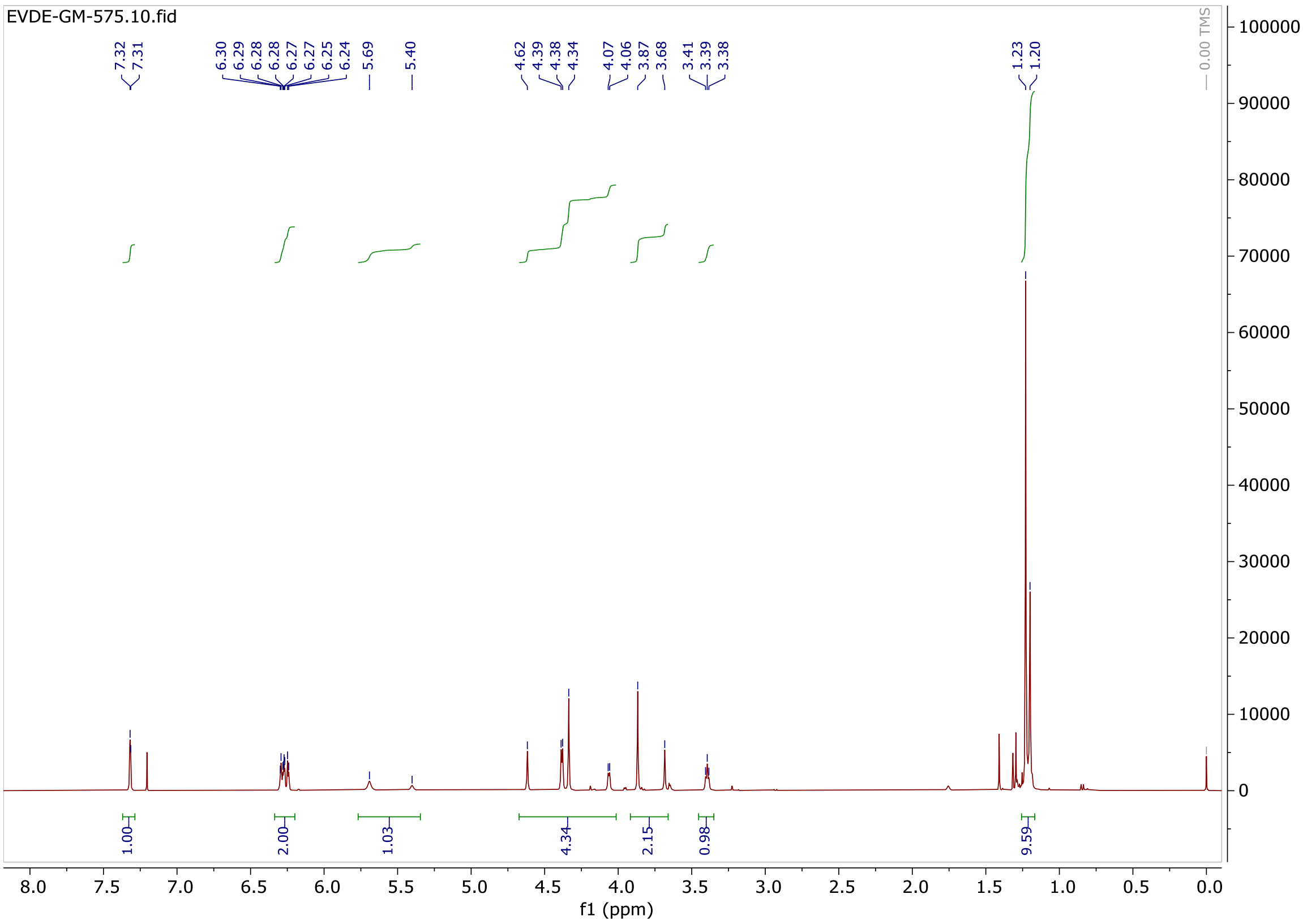

Figure S149. ^{1}H-NMR spectrum of compound **74.**

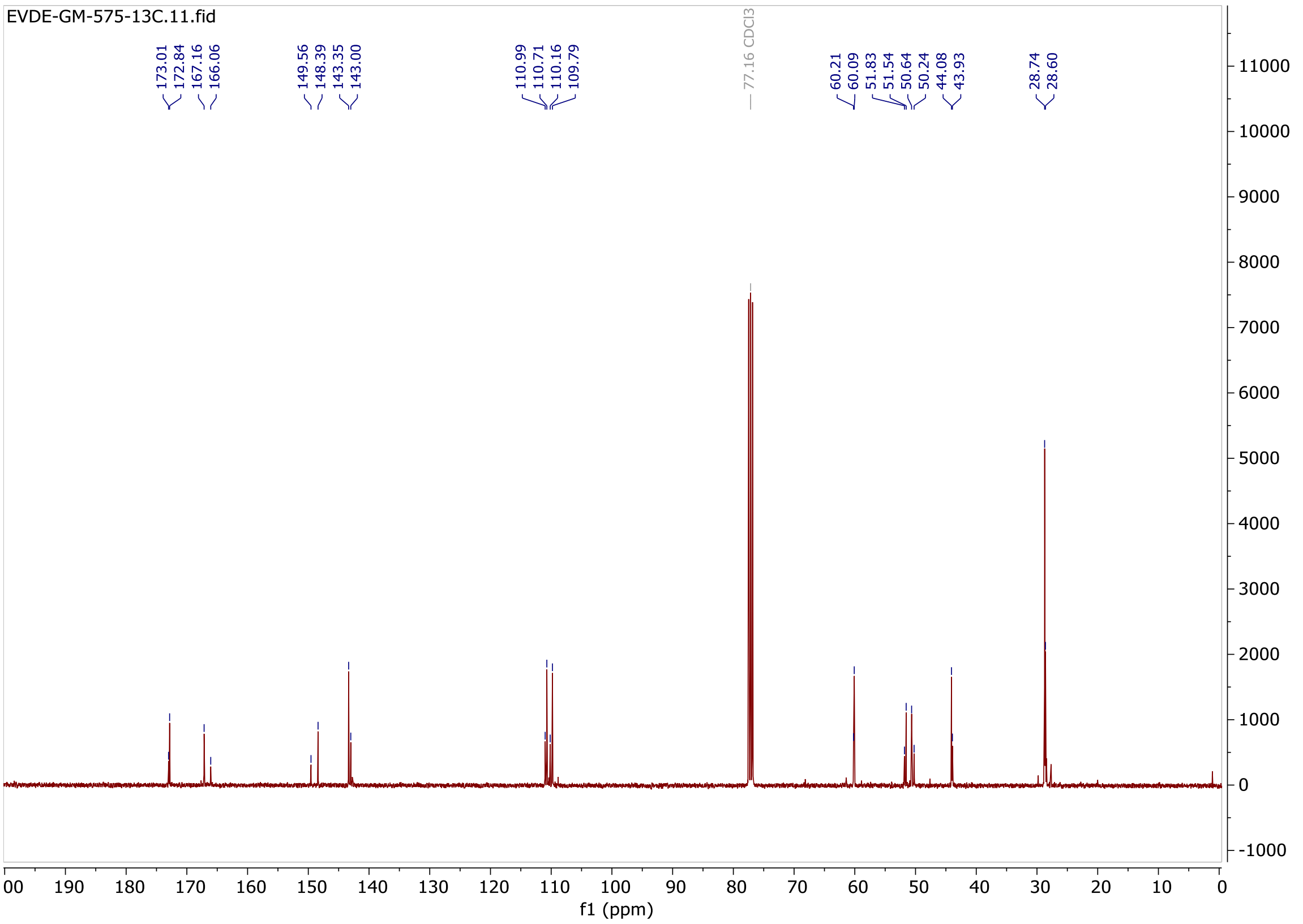

Figure S150. ^{13}C-NMR spectrum of compound **74.**

3. References

[1] G. M. Ojeda-Carralero, Multicomponent and Catalytic Methods for the Modification of Peptides and Peptidomimetics; KU Leuven: Belgium, 2022 (PhD Dissertation, Supervisors: E. V. Van der Eycken, D. G. Rivera, Defended September 21, 2022).

[2] J. González-Bacerio, J. Osuna, A. Ponce, R. Fando, K. Figarella, Y. Méndez, J-L. Charli, M. Á. Chávez, *Protein Expr. Purif.* **2014**, 104, 103-114.

[3] M. Izquierdo, M. E. Aguado, M. Zoltner, J. González-Bacerio, *Protein J.* **2019**, 38, 167-180.

[4] M. E. Aguado, M. González-Matos, M. Izquierdo, J. Quintana, M. C. Field, J. González-Bacerio, *Protein Expr. Purif.* **2021**, 183, 105877. doi: 10.1016/j.pep.2021.105877

[5] J. González-Bacerio, S. E. C. Maluf, Y. Méndez, I. Pascual, I. Florent, P. M. S. Melo, A. Budu, J. C. Ferreira, E. Moreno, A. K. Carmona, D. G. Rivera, M. Alonso del Rivero, M. L. Gazarini, *Bioorg. Med. Chem.* **2017**, 25, 4628-4636.

[6] M. Izquierdo, D. Lin, S. O'Neill, M. Zoltner, L. Webster, A. Hope, D. W. Gray, M. C. Field, J. González-Bacerio, *SLAS Discov.***2020**, 25, 1064-1071.

[7] J. González-Bacerio, A. K. Carmona, M. L. Gazarini, M. Á. Chávez, M. Alonso del Rivero, Kinetic characterization of recombinant PfAM1, a M1-aminopeptidase from *Plasmodium falciparum* (Aconoidasida: Plasmodiidae), using fluorogenic peptide substrates. *Rev. Cub. Cienc. Biol*. **2015**, 4, 40-48.

[8] R. A. Copeland, *Enzymes: A practical introduction to structure, mechanism, and data analysis*, Wiley-VCH, Inc., New York, **2000**, p. 266.

[9] D. Alpízar-Pedraza, A. N. Veulens, Y. M. Á. Ginarte, J. Piloto-Ferrer, Á. Sánchez-Lamar, Xanthatin and 8-epi-xanthatin as new potential colchicine binding site inhibitors: a computational study. *J. Mol. Model*. **2023,** 29, 36. doi: 10.1007/s00894-022-05428-w

[10] G. M. Morris, R. Huey, W. Lindstrom, M. F. Sanner, R. K. Belew, D. S. Goodsell, A. J. Olson, AutoDock4 and AutoDockTools4: Automated docking with selective receptor flexibility. *J. Comput. Chem*. **2009**, 30, 2785-2791.

[11] S. McGowan, C. J. Porter, J. Lowther, C. M. Stack, S. J. Golding, T. S. Skinner-Adams, K. R. Trenholme, F. Teuscher, S. M. Donnelly, J. Grembecka, A. Mucha, P. Kafarski, R. DeGori, A. M. Bucle, D. L. Gardiner, J. C. Whisstock, J. P. Dalton, *Proc. Natl. Acad. Sci. USA* **2009**, 106, 2537-2542.

[12] J. Timm, M. Valente, D. García-Caballero, K. S. Wilson, D. González-Pacanowska, Structural characterization of acidic M17 leucine aminopeptidases from the TriTryps and evaluation of their role in nutrient starvation in *Trypanosoma brucei*. *MSphere* **2017**, 2, e00226-17.
